# Similarities, variations, and evolution of cytochrome P450s in *Streptomyces* versus *Mycobacterium*

**DOI:** 10.1038/s41598-019-40646-y

**Published:** 2019-03-08

**Authors:** Louisa Moshoeshoe Senate, Martin Phalane Tjatji, Kayla Pillay, Wanping Chen, Ntokozo Minenhle Zondo, Puleng Rosinah Syed, Fanele Cabangile Mnguni, Zinhle Edith Chiliza, Hans Denis Bamal, Rajshekhar Karpoormath, Thandeka Khoza, Samson Sitheni Mashele, Jonathan Michael Blackburn, Jae-Hyuk Yu, David R. Nelson, Khajamohiddin Syed

**Affiliations:** 1 Unit for Drug Discovery Research, Department of Health Sciences, Faculty of Health and Environmental Sciences, Central University of Technology, Bloemfontein, 9300 Free State South Africa; 2 Department of Biochemistry and Microbiology, Faculty of Science and Agriculture, University of Zululand, KwaDlangezwa, 3886 KwaZulu-Natal South Africa; 3 College of Food Science and Technology, Huazhong Agricultural University, Wuhan, Hubei Province China; 4 Department of Pharmaceutical Chemistry, College of Health Sciences, University of KwaZulu-Natal, Durban, 4000 KwaZulu-Natal South Africa; 5 Department of Biochemistry, School of Life Sciences, University of KwaZulu-Natal (Pietermaritzburg campus), Scottsville, 3209 KwaZulu-Natal South Africa; 6 Institute of Infectious Disease & Molecular Medicine; Department of Integrative Biomedical Sciences, Faculty of Health Sciences, University of Cape Town, Cape Town, 7925 South Africa; 7 Department of Bacteriology, University of Wisconsin-Madison, 3155 MSB, 1550 Linden Drive, Madison, WI 53706 USA; 8 Department of Systems Biotechnology, Konkuk University, Seoul, Republic of Korea; 9 Department of Microbiology, Immunology and Biochemistry, University of Tennessee Health Science Center, Memphis, TN 38163 USA

## Abstract

Cytochrome P450 monooxygenases (P450s) found in all domains of life are known for their catalytic versatility and stereo- and regio-specific activity. While the impact of lifestyle on P450 evolution was reported in many eukaryotes, this remains to be addressed in bacteria. In this report, *Streptomyces* and *Mycobacterium*, belonging to the phylum *Actinobacteria*, were studied owing to their contrasting lifestyles and impacts on human. Analyses of all P450s and those predicted to be associated with secondary metabolism have revealed that different lifestyles have affected the evolution of P450s in these bacterial genera. We have found that while species in both genera have essentially the same number of P450s in the genome, *Streptomyces* P450s are much more diverse than those of *Mycobacterium*. Moreover, despite both belonging to *Actinobacteria*, only 21 P450 families were common, and 123 and 56 families were found to be unique to *Streptomyces* and *Mycobacterium*, respectively. The presence of a large and diverse number of P450s in *Streptomyces* secondary metabolism contributes to antibiotic diversity, helping to secure the niche. Conversely, based on the currently available functional data, types of secondary metabolic pathways and associated P450s, mycobacterial P450s seem to play a role in utilization or synthesis of lipids.

## Introduction

Cytochrome P450 monooxygenases (P450s/CYPs) are ubiquitously distributed in all domains of life, and even found in some non-living entities such as viruses^1,2^. P450s are catalytically versatile and cause stereo- and regio-specific enzymatic reactions^3,4^. Because of this unique nature, P450s have been in focus for more than five decades^5^. Whole genome sequencing of various organisms belonging to different biological domains and kingdoms have resulted in identification of more than 300,000 P450s^2^. Analyses of P450 evolutionary patterns with respect to species and their ecological niches is gaining a great momentum. This type of study has been reported involving eukaryotes; animals^6^, plants^7^, fungi^8–14^ and oomycetes^15^. In the reported studies researchers observed blooming of certain P450 families, the presence of unique P450 families or changes in the number of P450s with respect to species adaptation to specific ecological niches or lifestyles. However, to date, this type of evolutionary analysis has not been reported for bacteria. To address this research gap, we selected two genera, *Streptomyces* and *Mycobacterium*, belonging to the phylum *Actinobacteria*^15^, and assessed the impact of lifestyle on the evolution of P450s.

*Streptomyces* is the largest genus in the phylum *Actinobacteria*^16^. *Streptomyces* species are spore-forming filamentous bacteria and well known to produce various secondary metabolites including antibiotics^17^. It is estimated that more than 50% of commercially available antibiotics are produced by *Streptomyces* species^17^. A survey on the characteristics of *Streptomyces* species revealed that most of the species belonging to this genus produce chemically diverse secondary metabolites that are used in human medicine as antibiotics, anti-infectives, anti-fibrotic, antitumor and immunosuppressant drugs (Table S1). Much of the chemical diversity of secondary metabolites produced by *Streptomyces* species has been attributed to their symbiotic lifestyle, apart from their well-known saprophytic lifestyle^18^. Symbiosis of *Streptomyces* species with plants, fungi, and animals has been reported and in some cases parasitic symbiosis was identified^18^. *Streptomyces* species are known to produce geosmin, a volatile metabolite that is responsible for their characteristic “earthy” smell, and P450s were found to be involved in the production of this metabolite^19^.

On the other hand, the *Mycobacterium* genus includes pathogens known to cause serious diseases^16^. Generally, mycobacterial species are rod-shaped and some species show pleomorphism^20^. The *Mycobacterium* genus is well known for its human and animal pathogens, especially *M*. *tuberculosis*, a deadly human pathogen that is responsible for the death of millions of people across the world^21^. In a recent study mycobacterial species were classified into six different categories based on their lifestyles, site of infection, and other characteristics^22^. While most of the mycobacterial species are pathogens, some are saprophytes with potential biotechnological applications, such as bioremediation. Comprehensive comparative analysis of P450s in the genus *Mycobacterium* revealed that progression from soil mycobacteria into human pathogens, such as those living in human blood and ultimately adapted as a lung pathogen, resulted in gradual loss of a considerable number of P450s^22^. Furthermore, species belonging to each of the six categories were found to have category-specific P450s that can be used as a diagnostic marker in the detection and distinction of these species^22^.

However, to date, comprehensive analysis of P450s in the genus *Streptomyces* has not been reported. Furthermore, despite the contrast in lifestyles and copious dissimilarities between the species of *Streptomyces* and *Mycobacterium* (as mentioned above), striking analogies were observed in the developmental and morphological hallmarks of their life cycles^20^. Thus, we carried out genome-wide P450 analyses of the genus *Streptomyces*, along with comprehensive comparative analyses with mycobacterial P450s to assess the impact of lifestyle on the evolution of P450s, if any, between the two genera. Based on comprehensive comparative analyses of P450s and secondary metabolite biosynthetic gene clusters (BGCs) profiling, a logical conclusion on the evolution of P450s in both genera is provided.

## Methods

### *Streptomyces* species and genome databases

Forty-eight *Streptomyces* species genomes that are available for public use at Integrated Microbial Genomes & Microbiomes from the Joint Genome Institute (https://img.jgi.doe.gov/) and Kyoto Encyclopedia of Genes and Genomes (KEGG)^23^ were used in this study. Detailed information on species used in this study, along with their genome database links and genome IDs, are listed in Supplementary Table S2.

### Genome data-mining and identification of P450s

*Streptomyces* species genomes that are publicly available as listed in Table S2 were mined for P450s following the method described elsewhere^14,22,24^. Briefly, for each bacterium, whole proteomes were downloaded and grouped into different protein families using the National Centre for Biotechnology and Information (NCBI) Conserved Domain Database: NCBI Batch Web CD-search tool^25^. The hit proteins grouped under the cytochrome P450 monooxygenases superfamily were selected for further study. The bacterial genome available at Integrated Microbial Genomes & Microbiomes from the Joint Genome Institute was mined for P450s using InterPro code “IPR001128”. The hit protein sequences were downloaded and subjected to the NCBI Batch Web CD-Search Tool^25^. Proteins that grouped under the P450 superfamily were selected for further analysis. The selected proteins were searched for the presence of P450 characteristic motifs such as EXXR and CXG. Proteins having one of the motifs were considered as fragment/pseudo P450s. For each organism, P450s identified at KEGG and Integrated Microbial Genomes & Microbiomes were compared. A final total count is presented by deleting the same P450s found in both genome databases.

### Assigning P450 family and P450 subfamily to orphan P450s

The above selected P450s were subjected to Basic Local Alignment Search Tool analysis against all named bacterial sequences on the Cytochrome P450 Homepage^26^ to identify the closest named homolog P450. Based on the percentage identity to the named homolog P450, i.e. >40% amino acid identity and >55% amino acid identity, P450s were grouped under the same family and same subfamily^27^. P450s that had less than 40% and 55% amino acid identity to the named homolog P450s were assigned to new P450 families and new P450 subfamilies. Some *Streptomyces* species P450s were annotated and made available at the Cytochrome P450 Homepage^26^. In this case, the same nomenclature for P450s was continued. The *Streptomyces* P450 protein sequences along with their names were listed in Supplementary Dataset 1. For comparative analysis the P450 data for mycobacterial species were retrieved from published literature^22^.

### Construction of the P450s phylogenetic tree

The phylogenetic tree of P450s was constructed as described previously^28^. First, the P450 protein sequences were aligned using the HMMER package 3.1b2 (http://hmmer.org/) by adjusting them to the P450 profile hidden Markov model PF00067 from the Pfam database (http://pfam.xfam.org/)^29^. Then, the phylogenetic tree from P450s alignments was generated by FastTree 2.1.10 using the maximum-likelihood method (http://www.microbesonline.org/fasttree/)^30^. Finally, the phylogenetic tree was displayed by iTOL (http://itol.embl.de/upload.cgi)^31^.

### Analysis of P450 diversity percentage

The percentage contribution of the number of P450 families in the total number of P450s in an organism is considered the P450 diversity percentage^15,22^. This formula was employed previously to measure the P450 diversity percentage among species in a genus^15,22^. However, in order to compare two genera of a phylum where the number of species will be different, a new formula (shown below) has been formulated to obtain an average P450 diversity percentage per species.

$${\rm{P}}450\,{\rm{diversity}}\,{\rm{percentage}}=\frac{100\times {\rm{Total}}\,{\rm{number}}\,{\rm{of}}\,{\rm{P}}450\,{\rm{families}}}{{\rm{Total}}\,{\rm{number}}\,{\rm{of}}\,{\rm{P}}450{\rm{s}}\times {\rm{number}}\,{\rm{of}}\,{\rm{species}}}$$

The above formula will nullify the number of species used and will give an accurate P450 diversity percentage comparison between the genera within a phylum; previously this was not employed^15,22^. A point to be noted is that this formula is useful only when the number of species shared between the genera is relatively similar. For comparative analysis the P450 diversity percentage data for mycobacterial species was retrieved from published literature^22^.

### Generation of P450 profile heatmaps

The presence or absence of P450s in *Streptomyces* species was shown with heatmaps generated using P450 family data following the method described elsewhere^24^. Briefly, the data was represented as −3 for family presence (green) and 3 for family absence (red). A tab-delimited file was imported into Multi-experiment Viewer (Mev)^32^. Hierarchical clustering using a Euclidean distance metric was used to cluster the data. Forty-eight *Streptomyces* species formed the horizontal axis and CYP family numbers formed the vertical axis (see Supplementary Dataset 2).

### Secondary metabolite BGCs analysis

Secondary metabolite BGCs analysis was carried out following the method described elsewhere^24^. Briefly, individual *Streptomyces* species and mycobacterial species genome IDs from NCBI (Tables S2 and S3) were submitted to antibiotics & Secondary Metabolite Analysis Shell (anti-SMASH)^33^ for identification of secondary metabolite BGCs. Results from anti-SMASH were downloaded both in the form of gene cluster sequences and Excel spreadsheets representing species-wise cluster information, and finally, P450s that were part of a specific gene cluster were identified. Standard gene cluster abbreviation terminology available on the anti-SMASH database^33^ was maintained in this study.

### Functional analysis of P450s

Considering the large number of P450s identified in this study and the availability of functional data for some P450s^34^, a literature survey on the functional analysis of *Streptomyces* species P450s was carried out and used in this study. The functional role of P450s in *Streptomyces* physiology is presented at P450 family level and subfamily level. Furthermore, functional analysis of some *Streptomyces* species P450 was predicted based on the characterized homologous P450s from other organisms.

## Results and Discussion

### Identification of *Streptomyces* P450s

**Figure 1:**
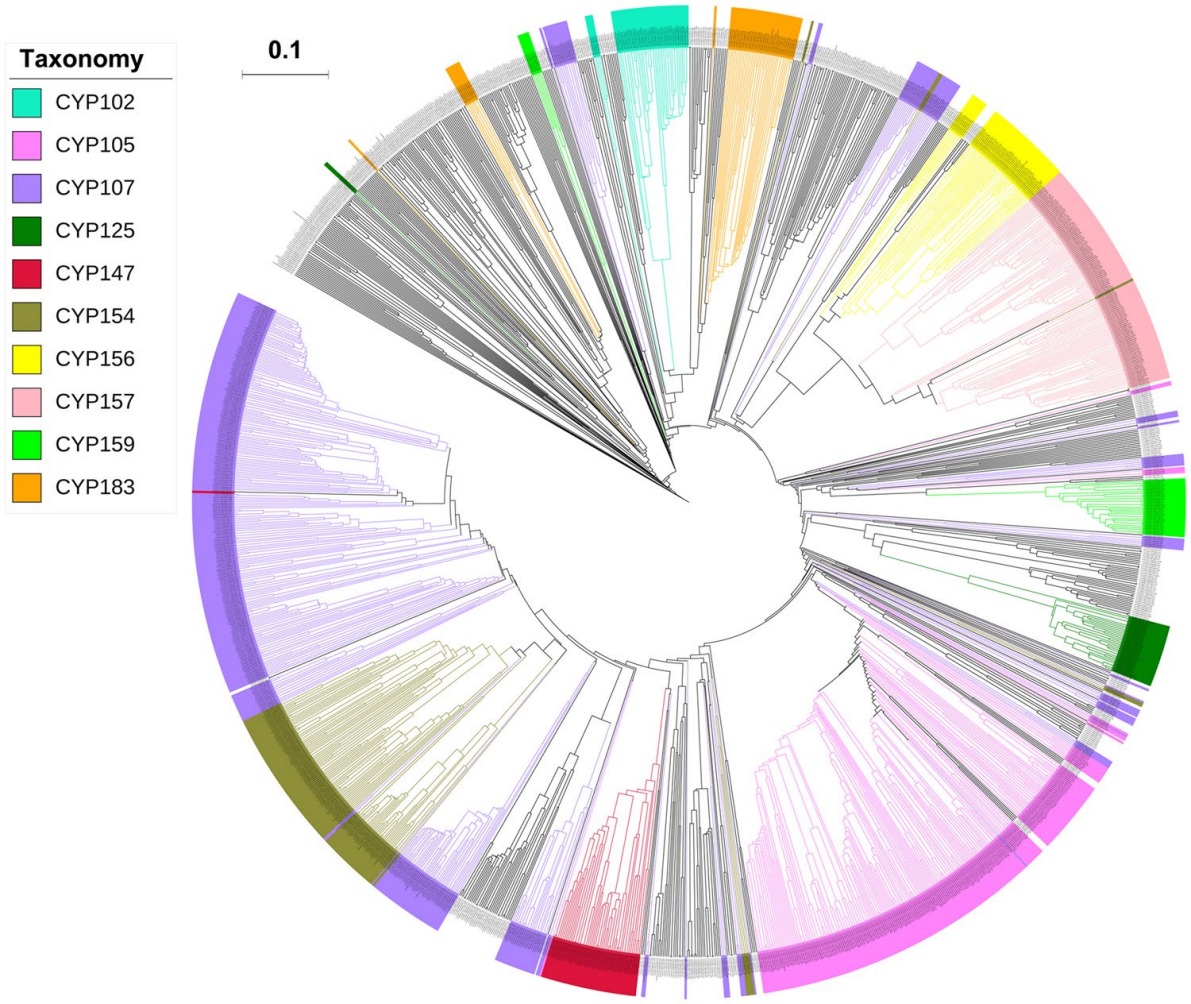
Phylogenetic analysis of *Streptomyces* P450s. P450 families that are dominant in *Streptomyces* species are highlighted in different colors. A high-quality figure is presented as Supplementary Dataset 3.

**Figure 2:**
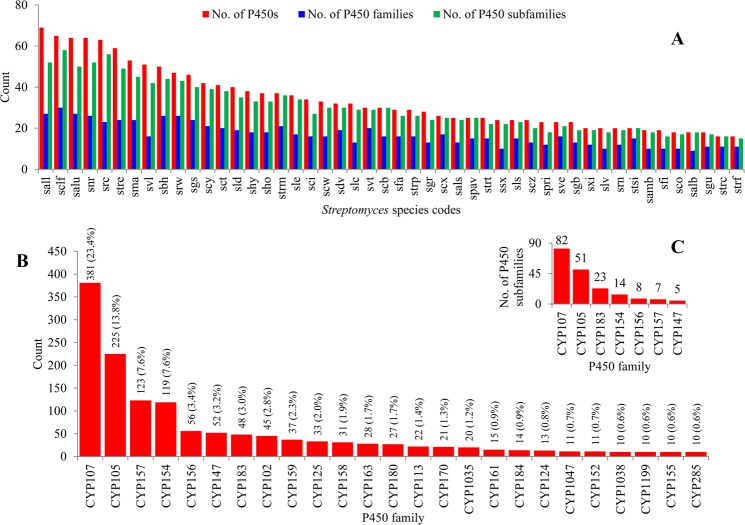
Comparative analysis of P450s in 48 *Streptomyces* species. (**A**) Comparative analysis of the number of P450s, P450 families and P450 subfamilies in 48 *Streptomyces* species. Each *Streptomyces* species is presented with its code (for details see Table S4). (**B**) P450 family level comparative analysis in *Streptomyces* species. P450 families that are dominant in *Streptomyces* species are presented in the figure. The numbers next to the family bar indicate the total number of P450s and percentage contribution (parenthesis) by a particular family to the total number of P450s (for details see Table S6). (**C**) P450 subfamily level comparative analysis among seven dominant P450 families. The numbers next to bars indicate the number of P450 subfamilies in that family.

Genome-wide data mining and annotation of P450s in 48 *Streptomyces* species revealed the presence of 1625 P450s in their genomes (Fig. 1 and Table S4). Among these 1625 P450s, all had characteristic glutamic acid and cysteine at EXXR and CXG motifs, respectively; however, thirty four P450s lacked one or both P450 characteristic motifs, EXXR and CXG, owing to short amino acid sequences and were thus regarded as fragment/pseudo P450s (see Supplementary Dataset 1). The presence of short P450s/pseudo P450s is common in organisms. Two false positive P450 fragments were identified in *S*. *ambofaciens* ATCC 23877 and one in *Streptomyces* sp. CdTB01. These P450 fragments were not included in the final count. The P450 count in the *Streptomyces* species ranged from 16–69 P450s, with an average of 34 P450s (Fig. 2A and Table S4). Among the *Streptomyces* species selected for the study, *Streptomyces* sp. CNQ-509 and *Streptomyces* sp. 4F have the lowest number of P450s (16 P450s) and *Streptomyces albulus* ZPM the highest number of P450s (69 P450s) in their genomes (Fig. 2A). The percentage coverage of P450s in the *Streptomyces* species ranged from 0.2% to 1.1% in (Table S4). Comparison of P450s revealed that species belonging to the genera *Streptomyces* and *Mycobacterium* had almost the same patterns in terms of the average number of P450s in their genomes, the highest average percentage contribution of P450s in the genome (≥1%) and highest number of P450s for a species (*S*. *albulus* ZPM has 69 P450s and *M*. *rhodesiae* NBB3 has 70 P450s).

### *Streptomyces* species have more diverse P450s than mycobacterial species

As per International P450 Nomenclature Committee Rules^27^, all 1625 P450 identified in 48 *Streptomyces* species were grouped into 144 P450 families and 377 P450 subfamilies (Fig. 2A and Supplementary Dataset 2). Among the families and subfamilies, 66 new P450 families and 144 new P450 subfamilies were identified in *Streptomyces* species (Table S5). Most of the new P450 subfamilies were identified in the P450 families CYP107 (62 new subfamilies) and CYP105 (38 new subfamilies) (Table S5). A detailed list of newly identified P450 families and P450 subfamilies in *Streptomyces* species is presented in Table S5. An interesting feature observed during phylogenetic analysis of *Streptomyces* P450s is that some P450 family members are not grouped together (Fig. 1 and Supplementary Dataset 3), despite being annotated according to the rules set by the International P450 Nomenclature Committee^27^, suggesting that sometimes the phylogenetic-based annotation of P450s could be detecting similarity cues beyond a simple percentage identity cutoff. It is especially difficult to assign subfamily membership in large families such as CYP105 and CYP107.

**Figure 3:**
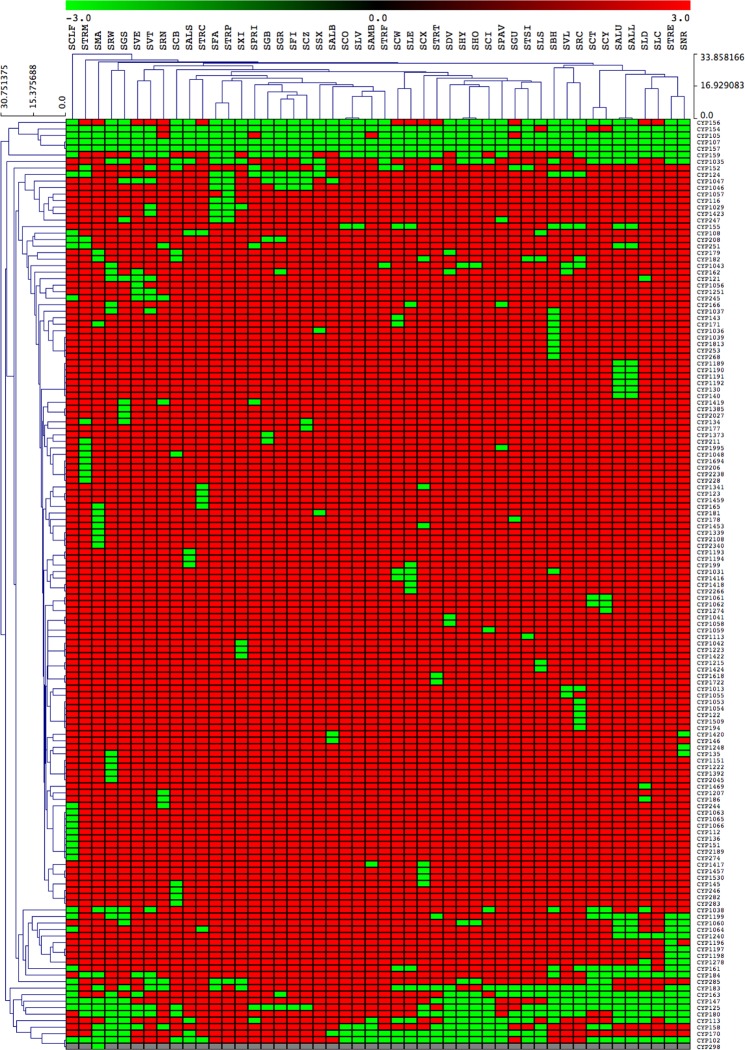
Heatmap of the presence or absence of P450 families in 48 *Streptomyces* species. Forty-eight *Streptomyces* species form the horizontal axis (see Table S2 for species codes) and P450 families (right side) and the phylogenetic relationship among P450 families (left side) form the vertical axis. The data have been represented as −3 for family presence (green) and 3 for family absence (red). The data used in the generation of the figure are presented in Supplementary Dataset-sheet 2.

Analysis of P450 family conservation across 48 *Streptomyces* species revealed that among 144 P450 families identified, only two P450 families, the CYP107 and CYP157 P450 families, are conserved across all *Streptomyces* species (Fig. 3 and Supplementary Dataset 2). Certain P450 families, such as CYP105 and CYP156, tend to co-occur in some but not all *Streptomyces* species (top of the heatmap).

The P450 family number ranged from nine to 30; *S*. *clavuligerus* has the highest number of P450 families (Fig. 2A and Table S4). P450 subfamilies ranged from 15 to 48, with *Streptomyces* sp. 4F and *S*. *clavuligerus* having the lowest and highest number of P450 subfamilies (Fig. 2A and Table S4). Analysis of P450 families revealed that 25 P450 families were highly populated and contributed 85% of all the P450s identified in 48 *Streptomyces* species (Fig. 2B and Table S6). This indicates that these P450 families play an important role in *Streptomyces* species’ physiology and are thus maintained in high numbers. It is noteworthy that P450s possibly provide important functions, as most of the bacteria lack P450s. This phenomenon of maintaining the highest number of certain P450 family members is not new and has been observed in microorganisms such as fungi^9,12^. However, unlike fungal species where only specific subfamilies are populated for a dominant P450 family^9,12^, *Streptomyces* species’ dominant P450 family analysis revealed high diversity in terms of the number of subfamilies (Fig. 2C): the CYP107 family has 82 subfamilies, followed by CYP105 with 51 subfamilies, CYP183 with 23 subfamilies, CYP154 with 14 subfamilies, CYP156 with eight subfamilies, CYP157 with seven subfamilies and CYP147 with five subfamilies (Fig. 2C).

**Figure 4:**
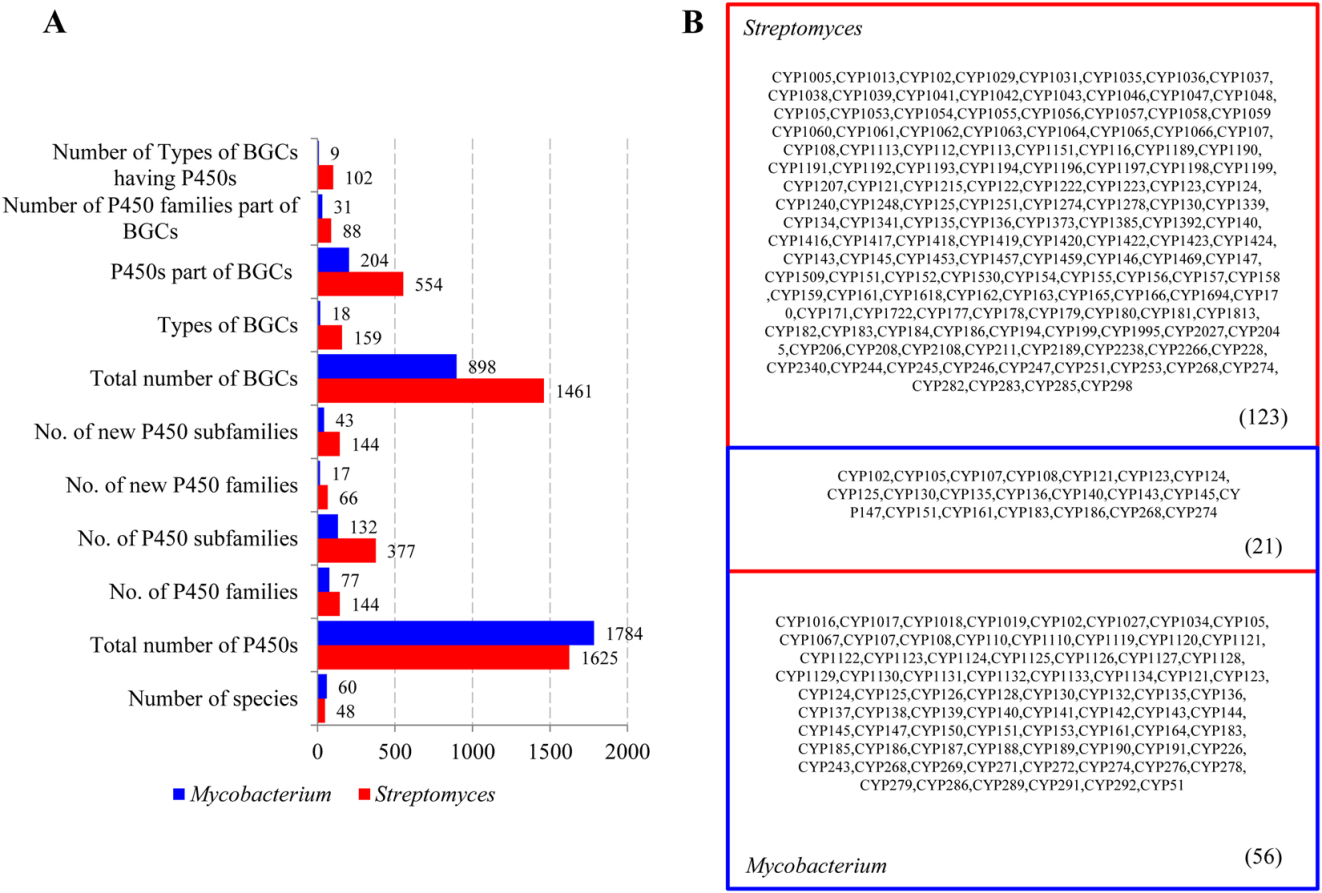
Different characteristics of P450s between *Streptomyces* and *Mycobacterium*. (**A**) Comparative analysis of key features of P450s between the genera *Streptomyces* and *Mycobacterium*. Y-axis indicates the count for each of the key features. (**B**) Comparative analysis of P450 families between *Streptomyces* and *Mycobacterium*. The numbers in parenthesis indicate the number of P450 families that are common and those unique to each genus.

Comparative analysis of P450 family dynamics between the genera *Streptomyces* and *Mycobacterium* revealed the presence of the highest number of P450 families and P450 subfamilies in *Streptomyces* species (Fig. 4A). *Streptomyces* species have 144 P450 families and 377 P450 subfamilies compared to 77 P450 families and 132 P450 subfamilies in mycobacterial species (Fig. 4A). *Streptomyces* species also have the highest number of new P450 families (66 families) and new P450 subfamilies (144 subfamilies) in their genomes compared to mycobacterial species (Fig. 4A). Interestingly, only two P450 families (CYP107 and CYP157) conserved in *Streptomyces* compared to mycobacterial species where 10 P450 families, namely CYP51, CYP123, CYP125, CYP130, CYP135, CYP136, CYP138, CYP140, CYP144 and CYP1128, were conserved^22^. Furthermore, P450 diversity percentage analysis between two genera revealed that *Streptomyces* species had almost double the P450 diversity percentage (0.18%) than mycobacterial species (0.07%). The factors responsible for the highest P450 diversity in *Streptomyces* are discussed in the next section.

### *Streptomyces* and *Mycobacterium* species show contrasting P450 profiles

**Figure 5:**
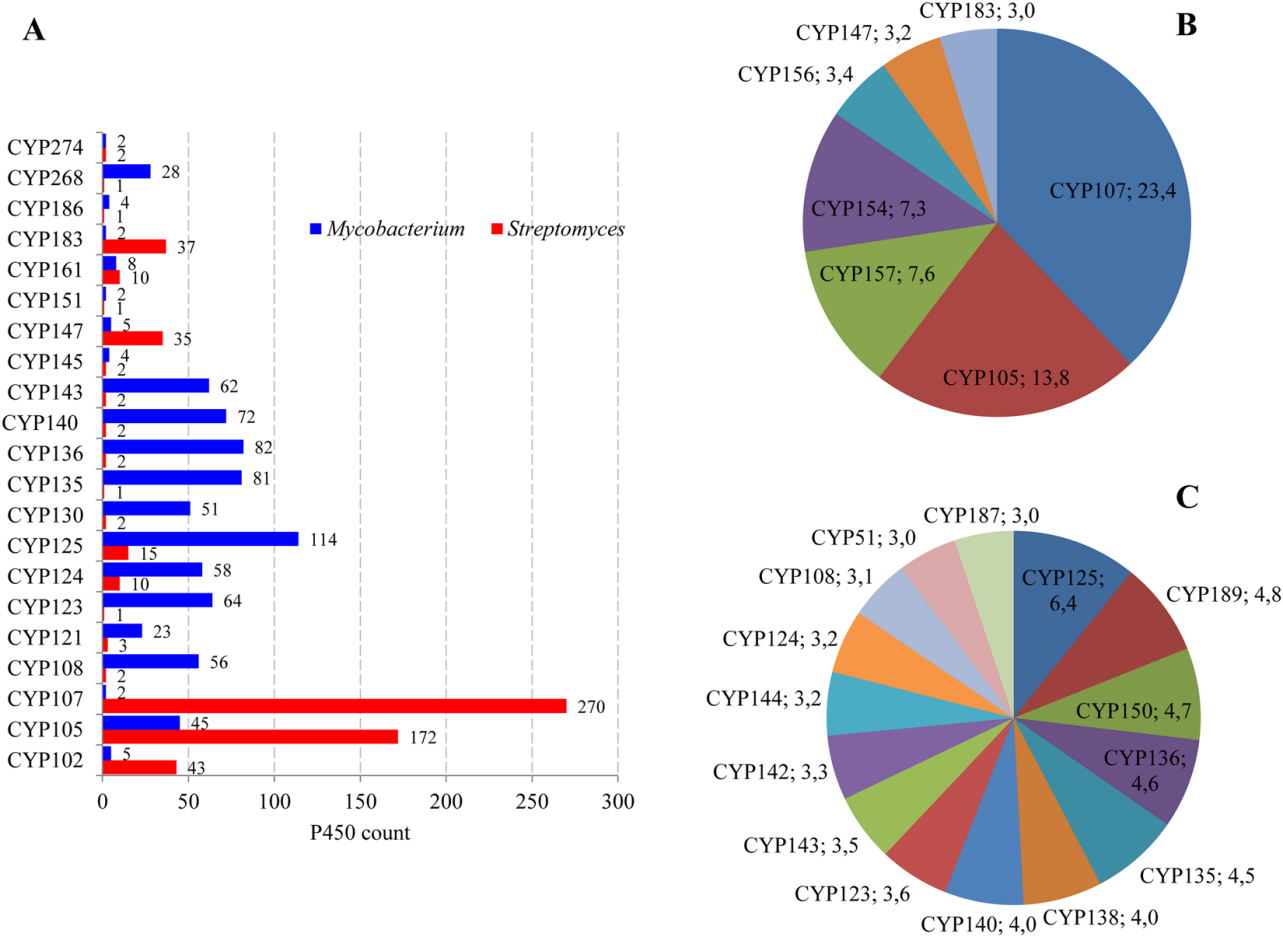
Comparative analysis P450 families between the genera *Streptomyces* and *Mycobacterium*. (**A**) Comparative analysis of member P450s in P450 families common between the genera *Streptomyces* and *Mycobacterium*. The numbers next to bars represent the number of P450s in the P450 family. Comparative analysis of dominant P450 families between the genera *Streptomyces* (**B**) and *Mycobacterium* (**C**). P450 families that are dominant in a genus are presented along with their names and percentage contribution to the total number of P450s in a genus. A detailed analysis of the percentage contribution of each P450 family in *Streptomyces* species is presented in Table S6.

Comparative analysis of P450 profiles between the genera *Streptomyces* and *Mycobacterium* revealed that species belonging to these genera have different P450 profiles with few similarities (Fig. 4B). Despite both genera belonging to the same phylum, *Actinobacteria*, only 21 P450 families were found to be common and quite a large number of P450 families were found to be unique to *Streptomyces* (123 P450 families) and *Mycobacterium* (56 P450 families) (Fig. 4B). In the 21 P450 families commonly found between the two genera, an interesting feature was observed in terms of the number of member P450s (Fig. 5A). A significant difference in the number of member P450s in the commonly shared P450 families was observed between *Streptomyces* and *Mycobacterium* (Fig. 5A). The P450 families CYP102, CYP105, CYP107, CYP147, CYP161 and CYP183 were highly populated in *Streptomyces* species, whereas the P450 families CYP108, CYP121, CYP123-CYP125, CYP130, CYP135, CYP136, CYP140, CYP143 and CYP268 were highly populated in mycobacterial species (Fig. 5A).

Differences were also observed in the number of dominant P450 families in the two genera (Fig. 5B,C). Only seven P450 families, namely CYP107, CYP105, CYP157, CYP154, CYP156, CYP147 and CYP183, contributed 62% of all P450s in *Streptomyces* species, whereas 15 P450 families, namely CYP125, CYP189, CYP150, CYP136, CYP135, CYP138, CYP140, CYP123, CYP143, CYP142, CYP144, CYP124, CYP108, CYP51 and CYP187, contributed 60% of all P450s in *Mycobacterium* (Fig. 5B,C). An interesting feature was that the percentage contribution of families was highest in *Streptomyces*, i.e. 23.4% by CYP107 and 13.8% by CYP105, compared to *Mycobacterium* P450 families, where the highest contribution was 6.4% by CYP125 (Fig. 5B,C). Furthermore, differences in P450 profiles between the two genera were observed in terms of type of dominant P450 families (Fig. 5B,C). A comparison of the dominant P450 families between the two genera revealed that none of the dominant P450 families was common between them (Figs 4B and 5B,C).

### *Streptomyces* species have a large and diverse number of secondary metabolite BGCs

**Figure 6:**
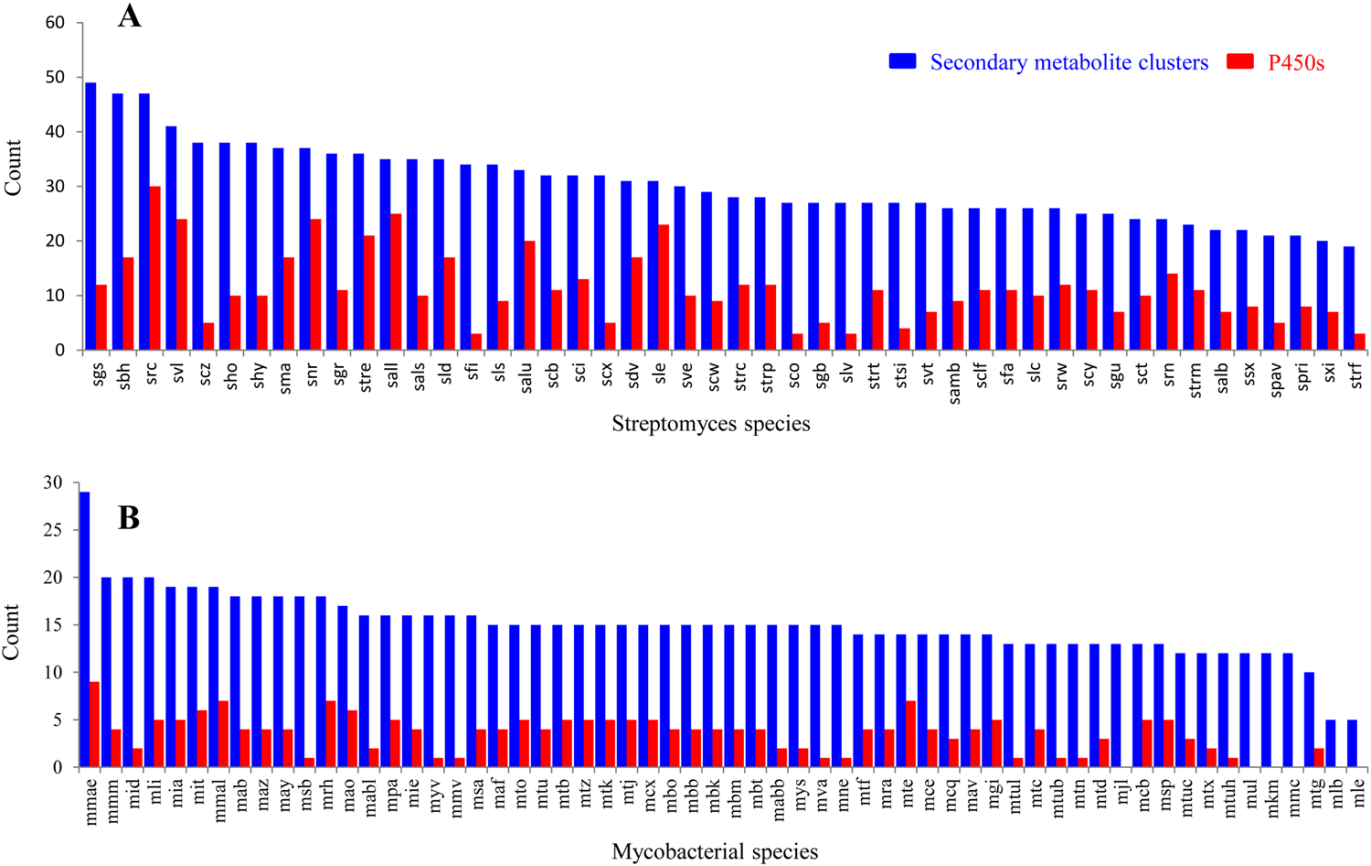
Comparative analysis of secondary metabolite BGCs and P450s associated with secondary metabolite BGCs in *Streptomyces* species (**A**) and mycobacterial species. (**B**) Detailed information on secondary metabolite BGCs in *Streptomyces* species and mycobacterial species is presented in Supplementary Dataset 4.

**Figure 7:**
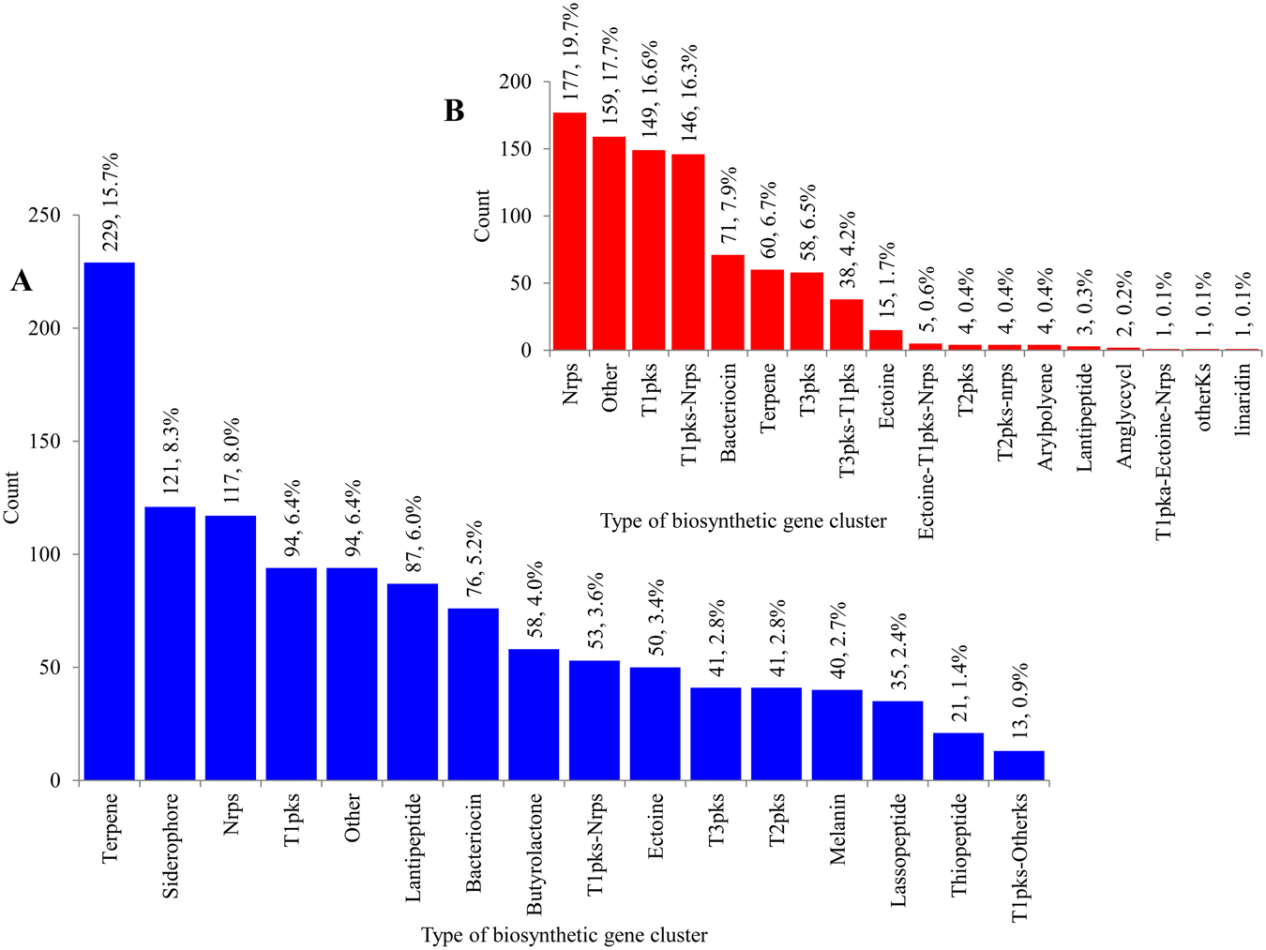
Comparative analysis of types of BGCs between *Streptomyces* species (**A**) and mycobacterial species. (**B**) The numbers next to bars represent the number of secondary metabolite BGCs and their percentage in the total number of BGCs found in 48 *Streptomyces* species (**A**) or 60 mycobacterial species. (**B**) Comparative analysis of types of secondary metabolite BGCs in *Streptomyces* species and mycobacterial species were presented in Supplementary Datasets 5 and 6, respectively. Furthermore, comprehensive comparative analysis of different types of secondary metabolite BGCs between *Streptomyces* and *Mycobacterium* is presented in Table S7.

*Streptomyces* species are well known for producing chemically diverse secondary metabolites (Table S1). Because of this ability one can expect the presence of a large number of secondary metabolite BGCs in *Streptomyces* species. As anticipated, genome-wide analysis revealed the presence of a large and diverse number of secondary metabolite BGCs in *Streptomyces* species compared to mycobacteria species (Figs 4A, 6 and 7). Almost double the secondary metabolite BGCs and eight times the types of BGCs were found in 48 *Streptomyces* species compared to mycobacterial species (Fig. 4A). In total 1 461 secondary metabolite BGCs belonging to 159 types were found in 48 *Streptomyces* species compared to 898 secondary metabolite BGCs belonging to only 18 types found in 60 mycobacterial species (Fig. 4A). The average number of secondary metabolite BGCs was found to be double in *Streptomyces* species (30) compared to mycobacterial species (15) (Fig. 6). Among *Streptomyces* species, *S*. *griseochromogenes* has the highest number of secondary metabolite BGCs (49) and *Streptomyces* sp. 4F has the lowest number of secondary metabolite BGCs (19) in their genomes (Fig. 6A). *M*. *marinum* has the highest number of secondary metabolite BGCs (29) and two strains of *M*. *leprae* have the lowest number of secondary metabolite BGCs (five each) in their genomes (Fig. 6B). Detailed information on secondary metabolite BGCs found in each of the species belonging to the genera *Streptomyces* and *Mycobacterium* is presented in Supplementary Dataset 4.

**Figure 8:**
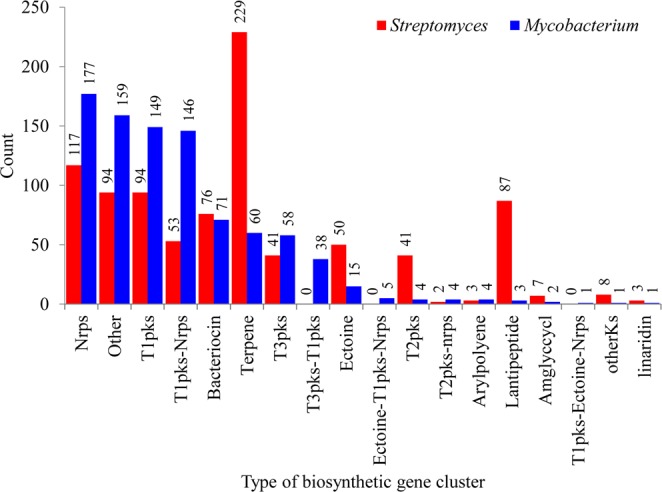
Comparative analysis of secondary metabolite BGCs commonly found in both *Streptomyces* and mycobacterial species. The number on top of each bar represents the number of types of BGCs present in 48 *Streptomyces* and 60 mycobacterial species.

As mentioned earlier, quite a big difference was observed with respect to the types of secondary metabolite BGCs between *Streptomyces* and mycobacterial species (Fig. 7 and Table S7). Among 159 types of secondary metabolite BGCs found in *Streptomyces* species, only 13 types of BGCs contributed 80% to the total percentage of BGCs (Fig. 7A), suggesting that the secondary metabolites produced by these BGCs were highly important in *Streptomyces* species physiology. The secondary metabolite BGC Terpene was dominant, followed by Siderophore, Nrps and T1pks in *Streptomyces* species (Fig. 7A and Supplementary Dataset 5). Among 18 types of secondary metabolite BGCs identified in mycobacterial species, Nrps was the dominant secondary metabolite BGC, followed by Other, T1pks and T1pks-Nrps (Fig. 7B and Supplementary Dataset 6). Despite the presence of 15 common types of secondary metabolite BGCs between the genera *Streptomyces* and *Mycobacterium*, large differences were observed in terms of the number of BGCs (Fig. 8), indicating that the selective enrichment of particular secondary metabolite BGCs in both genera is possibly due to the different lifestyle, as discussed in the subsequent section.

### *Streptomyces* species have a large and diverse number of P450s in secondary metabolite BGCs

**Figure 9:**
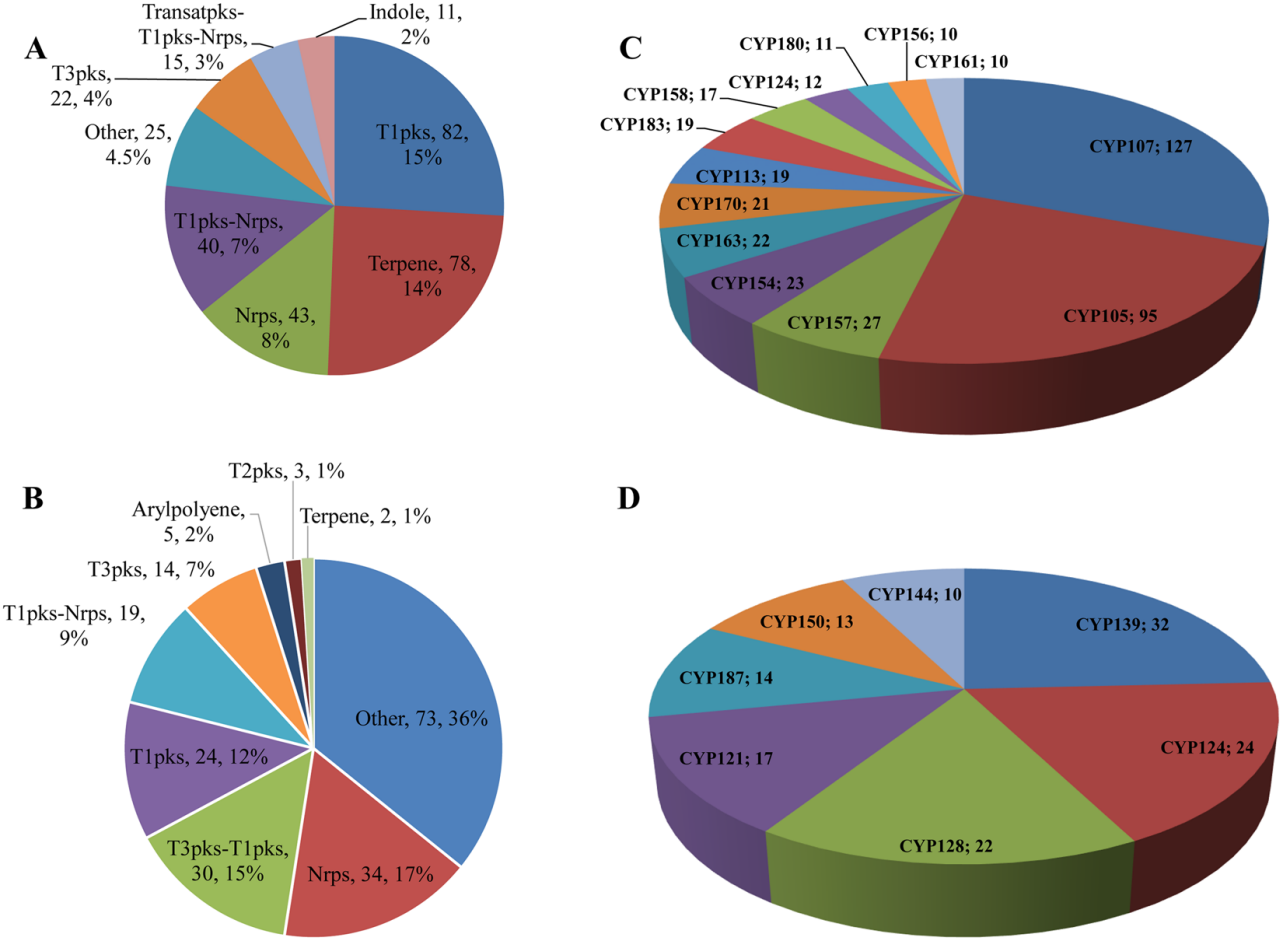
Comparative analysis of P450s associated with secondary metabolism. Comparative analysis of BGCs containing P450s in 48 *Streptomyces* species (**A**) and 60 mycobacterial species. (**B**) Numbers next to BGCs indicate the number of BGCs and their percentage in the total number of BGCs containing P450s (for details see Tables S8 and S9). Comparative analysis of P450 families that are part of secondary metabolite BGCs in *Streptomyces* species (**C**) and mycobacterial species. (**D**) The P450 families that are dominantly present in different BGCs are presented in the figure. The number after the P450 name indicates the number of member P450s. Detailed information on P450s that are part of BGCs is presented in Table S10.

Comparative analysis of P450s that are part of secondary metabolite BGCs revealed the presence of a large number of diverse P450s as part of these secondary metabolite BGCs in *Streptomyces*, compared to *Mycobacterium* (Figs 4A and 9; Tables S8 and S9). Not all secondary metabolite BGCs found in *Streptomyces* and *Mycobacterium* have P450s. Among 1 461 in *Streptomyces* species, only 554 secondary metabolite BGCs were found to have P450s, whereas in mycobacterial species, among 898 only 204 secondary metabolite BGCs have P450s (Fig. 4A). Moreover, not all types of secondary metabolite BGCs have P450s: 64% and 50% of types of secondary metabolite BGCs of *Streptomyces* and *Mycobacterium* were found to have P450s (Figs 4A and 9; Tables S8 and S9). Furthermore, a large difference was observed in the number of different P450 families that are part of secondary metabolite BGCs between the genera *Streptomyces* and *Mycobacterium*; the former genus has 88 P450 families and the latter only 31 P450 families, strongly indicating the possibility that diverse P450s are involved in the generation of diverse secondary metabolites in *Streptomyces* compared to *Mycobacterium*. Overall, 554 and 204 P450s were found to be part of secondary metabolite BGCs in *Streptomyces* and mycobacterial species (Fig. 4A and Tables S8 and S9). An interesting pattern was observed when comparing the number of P450s that are part of different types of secondary metabolite BGCs, revealing that dominant secondary metabolite BGCs are not necessarily dominant in terms of having P450s (Fig. 9A and B). In *Streptomyces* species, the secondary metabolite BGC, T1pks, the fourth dominant BGC (Fig. 7A), has the highest number of P450s (82 P450s) (Fig. 9A). Terpene, despite the dominant BGC in *Streptomyces* (Fig. 7A), has the second highest number of P450s (Fig. 9A). The same pattern was observed in mycobacterial species (Fig. 9B) where Others, the second dominant BGC (Fig. 7B), have the highest number of P450s compared to Nrps, the dominant BGC (Fig. 7B), which has the second highest number of P450s (Fig. 9B).

Among 88 P450 families that are part of different secondary metabolite BGCs, some P450 families are highly dominant in *Streptomyces* species (Fig. 9C and Table S8), indicating their key role in the synthesis of different secondary metabolites. These P450 families (Fig. 9C) were found to be the same that are highly populated in *Streptomyces* species (Fig. 2B), with some exceptions. This strongly indicates that these P450 families provide an advantage to *Streptomyces* species by generating diverse secondary metabolites, thus letting these species thrive in nature. Despite P450s families such as CYP102, CYP159, CYP125, CYP1035, CYP1038 and CYP155 being highly populated in *Streptomyces* species (Fig. 2B), their percentage contribution as part of secondary metabolite BGCs is quite negligible (Table S10), indicating further selectivity in terms of which P450s should be part of secondary metabolite BGCs. Mycobacterial species’ secondary metabolite BGC P450 profiles (Fig. 9D) are contrasted with *Streptomyces* species, where the dominant P450 families are not necessarily dominant as part of secondary metabolite BGCs. Ten P450 families, namely CYP51, CYP123, CYP125, CYP130, CYP135, CYP136, CYP138, CYP140, CYP144 and CYP1128, are conserved and highly populated across 60 mycobacterial species^22^. However, none of these P450 families is dominant as part of different secondary metabolite BGCs (Fig. 9D and Table S10). In fact, P450 families such as CYP123, CYP130 and CYP1128 are not found to be part of any secondary metabolite BGCs and P450 families such as CYP136, CYP125, CYP138 and CYP140 are rarely present as part of different secondary metabolite BGCs (Table S9). Their percentage contribution as part of secondary metabolite BGCs is thus very low (Fig. 9D and Table S10). The CYP139 P450 family was found to be the dominant family as part of secondary metabolite BGCs (Fig. 9D). Although eight P450 families, namely CYP124, CYP121, CYP105, CYP125, CYP102, CYP147, CYP136 and CYP161, which are part of secondary metabolite BGCs, were commonly found between the genera *Streptomyces* and *Mycobacterium*, the number of P450s in these families was found to be different (Table S10).

### Predicted functions of *Streptomyces* P450s

Functional analysis P450s based on characterized homolog P450s from other microorganisms and P450s from *Streptomyces* species^34^ revealed that most of the *Streptomyces* P450s were involved in secondary metabolite production (Table S11). This strongly supports the concept that these *Streptomyces* P450s play a key role in the production of chemically diverse secondary metabolites, as a large number of P450 families were found in 48 *Streptomyces* species. Detailed analysis of P450 functions according to general functions and specific functions at P450 family and P450 subfamily level is presented in Tables S11 and S12, respectively. The P450 family CYP180 was found to be part of a gene cluster that produces geosmin^19^. Among the P450 families involved in secondary metabolite production, 88 P450 families are uniquely present in *Streptomyces* species compared to mycobacterial species (Fig. 4B and Table S10). P450 families, namely CYP105, CYP107, CYP161, and CYP183, which are highly populated in *Streptomyces* species compared to mycobacterial species (Fig. 5B and C), were found to be involved in secondary metabolite production (Fig. 9C). This strongly suggests that these P450 families have been populated in *Streptomyces* species owing to their importance and necessity in secondary metabolite production. Therefore, the diversity among these P450 families, judged by the large number of P450 subfamilies, presumably serves to increase the resultant chemical diversity further across different *Streptomyces* species. It is well established that P450s are one of the key enzymes contributing to the diversity of secondary metabolites in organisms^35^. In contrast to the P450 families highly populated in *Streptomyces* species, P450 families that are highly populated in mycobacterial species such as CYP125, CYP124, CYP108, CYP140 and CYP268 (Fig. 5C) are involved in steroid (cholesterol) and hydrocarbon (lipids, alkenes, long chain acetate and ketone) hydroxylation (Fig. 5C and Table S11), suggesting that these P450 families possibly help mycobacterial species to assimilate the host compounds. It is noteworthy that results from this study revealed that some of these P450 families are rarely (CYP125 and CYP140) or not at all (CYP268) part of secondary metabolite BGCs in mycobacterial species (Table S10).

### Impact of lifestyle on the evolution of P450s in *Streptomyces* and *Mycobacterium*

Adaptation is key for the survival of an organism. Organisms adapt to different ecological niches by changing their gene pool and thus changing their physiology to make them suitable for survival in the new environment. The effect of ecological niches or lifestyle on P450s’ evolution in organisms such as animals^6^, plants^7^, fungi^8–14^ and oomycetes^15^ has been observed. In this study, for the first time, we present the influence of lifestyle on the evolution of P450s in a bacterial population. We present ample evidence of the impact of lifestyle on shaping the P450 profile in species belonging to the genera *Streptomyces* and *Mycobacterium*.

*Streptomyces* species are generally saprophytes living in soil or decaying vegetation, where the rule of survival of the fittest applies. Some studies reported symbiosis in *Streptomyces*, including parasitism^18^. In response to this type of ecological niche, *Streptomyces* adapted to produce different secondary metabolites (Table S1), which are harmful to other bacteria and thus *Streptomyces* species can survive and utilize the readily available carbon sources in their environment. In contrast to *Streptomyces* species, mycobacterial species are well-known pathogens of humans and other animals, despite some saprophytes being present in this genus^16,22^. The pathogenic nature of mycobacterial species forced them to adapt to lifestyles such as living in a host where evading the host’s immune system and utilizing host carbon sources for survival are the prime tasks. It is clear that different lifestyles influenced the P450 profiles in *Streptomyces* and *Mycobacterium*, hence the differences observed between the two genera in terms of number of P450s, P450 family and subfamily diversity, type of dominant and unique P450 families, differences in number of P450s in common P450 families, differences in number and type of secondary metabolite BGCs and P450s that are associated with secondary metabolite BGCs. Furthermore, functional analysis of P450s suggests that in *Streptomyces*, P450s are destined for secondary metabolite production, whereas in *Mycobacterium* they are destined for utilization of host lipids or synthesis of novel lipids. The presence of quite a large number of polyketide synthase biosynthetic gene clusters (identified in this study) (Fig. 7B) that are involved in the production of unique lipids and glycolipid conjugates^36^ further strengthens the argument that *Mycobacterium* P450s are involved in lipid metabolism. The saprophytic and symbiotic lifestyle of *Streptomyces* resulted in the highest diversity of secondary metabolite BGCs and P450s, thus helping these organisms to generate chemically diverse secondary metabolites to adapt to different ecological niches. For this reason, *Streptomyces* species have been found to have large and diverse secondary metabolite BGCs and P450s compared to mycobacterial species. Based on the evidence presented in this article, we hereby propose that lifestyle or ecological niches play a key role in the evolution of P450 profiles in species belonging to the genera *Streptomyces* and *Mycobacterium*.

## Supplementary information

Supplementary Information

Dataset 1

Dataset 2

Dataset 3

Dataset 4

Dataset 5

Dataset 6
