# Supplementary material for: Similarities, variations, and evolution of cytochrome P450s in Streptomyces versus Mycobacterium
Source: Sci Rep. 2019 Mar 8;9:3962. doi: 10.1038/s41598-019-40646-y (PMC6408508; doi:10.1038/s41598-019-40646-y)
Supplement: Supplementary file 1 — Supplementary Information [file 41598_2019_40646_MOESM1_ESM.docx]

**Similarities, variations, and evolution of cytochrome P450s in *Streptomyces* versus *Mycobacterium***

Louisa Moshoeshoe Senate^1@^, Martin Phalane Tjatji^1@^, Kayla Pillay^2@^, Wanping Chen^3@^, Ntokozo Minenhle Zondo^2^, Puleng Rosinah Syed^4^, Fanele Cabangile Mnguni^2^, Zinhle Edith Chiliza^2^, Hans Denis Bamal^1^, Rajshekhar Karpoormath^4^, Thandeka Khoza^5^, Samson Sitheni Mashele^1^, Jonathan Michael Blackburn^6^, Jae-Hyuk Yu^7,8^, David R Nelson^9*^, Khajamohiddin Syed^2*^

^1^ Unit for Drug Discovery Research, Department of Health Sciences, Faculty of Health and Environmental Sciences, Central University of Technology, Bloemfontein 9300, Free State, South Africa

^2^Department of Biochemistry and Microbiology, Faculty of Science and Agriculture, University of Zululand, KwaDlangezwa 3886, KwaZulu-Natal, South Africa

^3^ College of Food Science and Technology, Huazhong Agricultural University, Wuhan, Hubei Province, China

^4^ Department of Pharmaceutical Chemistry, College of Health Sciences, University of KwaZulu-Natal, Durban 4000, KwaZulu-Natal, South Africa

^5^Department of Biochemistry, School of Life Sciences, University of KwaZulu-Natal (Pietermaritzburg campus), Scottsville, 3209, KwaZulu-Natal, South Africa

^6^Institute of Infectious Disease & Molecular Medicine; Department of Integrative Biomedical Sciences, Faculty of Health Sciences, University of Cape Town, Cape Town 7925, South Africa

^7^Department of Bacteriology, University of Wisconsin-Madison, 3155 MSB, 1550 Linden Drive, Madison WI 53706, USA

^8^ Department of Systems Biotechnology, Konkuk University, Seoul, Republic or Korea

^9^ Department of Microbiology, Immunology and Biochemistry, University of Tennessee Health Science Center, Memphis, TN 38163, USA

 @ Authors contributed equally to the work

* Corresponding authors’ email:

[drnelson1@gmail.com](mailto:drnelson1@gmail.com) & [khajamohiddinsyed@gmail.com](mailto:khajamohiddinsyed@gmail.com)

**Supplementary Dataset 1: *Streptomyces* species P450s.** P450s were represented with their name following protein ID (in parenthesis) and species code. P450s that have one of the P450 characteristic motifs suc as EXXR and CXG and short in amino acid lengths were named as seudo/fragment P450s were listed in a tabular format after the sequences. All the P450s have invariantly conserved glutamic acid and cysteine at EXXR and CXG motifs except CYP157 which is well known not to have glutamic acid at EXXR motif (Rupasinghe et al., 2006).

>CYP147F21(2581179344)SALU

MNAKTTHGAREETVVDLAAYGAQFVENPYPVYAELRAKGPVHRVRVPGQERDFWLVVRNE

EGRRILADERLSKDWRSQGVWPADALPINENMVESDPPKHTRLRALVTRAFTARRIEALA

PRVHTLTADLLDAMSKAPNGRADLVAALAFPLSMTVICELLGVPDLDRQSFRQWTNEIVA

SSSPEATAEAVRAVNAYLTGLIEQKRAEPRDDLLSALLRTTDEEGDRLSPEEVVGMAFLL

LAAGHETTVGLISNTVLALLRHPDQLALLKADFSLIGNAVEETLRYDSPTENSTYRFATE

AMDFCGARFEKGDPVLVSLAAAGRDGERFEDGERFDITRSARGHLSFGHGIHYCLGAPLA

RLEAGVAVRALLERCPDLRLDTSEPLVYIPGMLVRGVRRLPVRWTG

>CYP1190A1(2581179455)SALU

MTTTDAHPPTSRTAAEGPPAFPFDDWGQRISPAYARLREAPAPACRVVTVTGDQVWLVTR

YDLARRLLADPRLSLTAALEADAPRQEPLRPRATGARGDGMATLQERGLRGILADALSPR

AIRAHHAWTRLRARALFDELSEQGPPADLQQGLARPLTFAVARRVLLGELTEDEGQVLNA

WCDTVLVWRDRTRDEIQAALDAMYGFFLRRAPELAAAPGSDVVKRAAAACTRDGGRLGAD

GLAEVANLMLIAGYRTAASFVANALVMMLSHPTALAALRDRPALLPSMVEEVLRHTPMST

GGVKRVATDDVPLDGLTIKAGECVLVSLESGNHDPHAYPEPDRFAPDRFAADRGPVDGTS

SEARRPRSRPHLGFGHGKHHCPGNALARMQIAVVLQTLADHTPALRLAVPAGELRWRPDV

AFRIPETIPVTW

>CYP154D13(2581179489)SALU

MDIAPVGPPHRMDPSGGCPHADNARLLARGAVAPVVLPGEIEGMAVLGHAALKEFLGHPD

VAKDARHFTALSEGRIPEGWPLRTFATVRSMTTADGEDHRRLRSLVSRSFTARRVAELQP

RVEELTDSLLDDLAGAARAGGGVADLRRHFALPLPMGVISELLGVDLAHRDRLHELSVEV

VTTDIGSQRAIAANHEFAAVIGEFVAAKARHPGDDLTSALIAARDDDGDQLSGPELIGTL

LLMIVAGHDTTLNLITNAVRALCGHRDQLELALSERVTWGDVVEETLRWDAPVSYFPFRY

PVRDLTLHGTVIPKGTPVLAGYSAAGRDPAAHGPDADRFDVTRPGRPDAVRHLSLGHGAH

YCLGAPLARLEAETALQRLFHRFPDLELAVPEDALPRHAGFVANSVGSLPVRLWPS

>CYP105A6(2581179626)SALU

MTDTATTPQTTDAPAFPSNRSCPYQLPDGYAQLRDTPGPLHRVTLYDGRQAWVVTKHEVA

RKLLGDPRLSSNRADTNFPATSPRFEAIRERPQAFIGLDPPEHGTRRRMTISEFTVKRIK

NMRPEVEEIVHGFLDEMLAAGPTADLVSQFALPVPSMVICRLLGVPYADHEFFQDASSRL

VQSTDAQSALTARNDLAGYLDGLITQFQTESGAGLVGALVADQLANGEIDREELISTAML

LLIAGHETTASMTSLSVITLLDHPEQFAALRADRSLVPGAVEELLRYLAIADTAGGRVAT

ADIEVEGQTIRAGEGVIVVNSIANRDGTVYEDPDALDIHRSARHHLAFGFGVHQCLGQNL

ARLELEVILNALMDRVPTLRLAVPVEQLVLRPGTTIQGVNELPVTW

>CYP105B37(2581179630)SALU

MPDATGPTPTPTGSAEPHHPSDVPEFPMPRAAGCPFDPPPTLTAQQQQGPLTKVRLWDGS

TPWLVTRYADQRALLADPRVSADVTRPGYPSAAPVSGNTIGFILMDDPEHARQRRMVTAP

FAVKRVEALRPRVQQIVDERIEALLGGPRPVDLVEAFALPVPSLVICELLGVPYADHDFF

QENSRILINRNVTPEERTAAHGRLSDYLDDLVGEKLARPMDDLLSQLAQRVADGELTRLD

AARMGVLLLIAGHETTANMIALGSLALLEHPGQLAALRASDDPKRVANAVEELLRYLHIT

HSGRRRVATADIELAGRTIRAGDGLIFPNDIANRDPDAFPDPDRLDLQRAARHHVAFGFG

VHQCLGQTLARLELQVVYGTLYRRIPTLRLAVPLADVPFKHDGSVYGVYELPVTW

>CYP107AM10(2581179632)SALU

MSHDDGTPAPAYPLTAPGALEAPAEWRELRTTCPVAPVTLPSGDRAALLTRYDDVKQVLS

DPRCTRQLDAEGAARISADPSGGVFNSAMAASLNGAGQQRWRRMLTKWFTAKRMNALRPA

IEAMAEQLVDEMVDRGHPADLKASVGFPLPVWVICDLLGVPAADRDRFSRWSDMLLNLTR

YGRDEIDTAQRDFHAYLTEHLEAKRAEPGEDLLSSLITATDVDGGRLTDDQLAATGQALL

IAGHETTANMIGKMMALLLADRRRWQQLVADPALVRTAVEEVLRYDANAGFGMPRYVTQD

IDVAGTVLPRGATLVCSMAAANRDGAVFAAADDLRLERSPNPHLAFGAGPHSCLGQALAR

TELQVVLDVLLRRLPSLELAVPVSELRRIEGLVVGGLCDVPVRW

>CYP140C3(2581179655)SALU

MSDLPYAQRLFARDVRWALGHALPRLATDRAARQGDLHGQLVALSRSPRPHGDTADVERR

LMDRIRAEGPVHRSRFGFVTASHPAVREVLSSNDFRTGALPVTTGPLGRLAAWAGADAPV

GPLKPPSLLVTEPPDHTRYRKLVTRVFSVRAVEQLRTRAEEIAEELLDDLQRRPPGADDV

DLVSAYCGLLPVTVIAEILGVPHAERHRVLRFGTGAAPSLDFGLPRRRFLAVERSLRDFD

AWLAQHIERIRRQPGANLLSQLVTARDDDGRGLTGTELRATAGLVLAAGFETTVNLLGNG

IALLDRHPDQRAALHDDPTLWPNAVDEMLRFDPPVFLTGRAATRDTSIGGRPVPRGALVT

LLLAGANRDAALFTAPHRFDVTRPNAKEHLSFSGGRHYCLGAALARMEAEVGLQALHRRF

PHLTLHAGARRRTTRILRGYVHLPARLGTPVPV

>CYP159A15(2581179714)SALU

MTSIPQAPDILSAQFATDPYPAYRVLREHYPLLHHEGTGSYLLSRYDDVERAFREPVFTS

DNYIWQLEPAHGGRTLPQLSGREHAVRRALVAPAFRGRELRETFQPLIERNARELIDAFR

DDDEVDLVAQFATRLPINVIVDMLGLDRADHDRFHDWYTAVVGFIANLAQDPAIADAGRR

AGEELAAYLHPIIQERRAAPGDDLLSRLCTAEVEGVRMTDQDITAFVSLLLSAGGETTDK

AIALVVRNLLAHPEQLAAVRADRSLVPAAFAETLRHTPPVQMIMRQPAEDVTVSGGTVPA

GATVTCLIGAANRDADRYAHPDAFDILRSDLTPDTAFSAAARHIAFGLGRHFCVGALLAK

AEVETSVNQLLDAFPDLAFADGVAPPDAGVFTRGPRQLRLRLRDRER

>CYP1060A2(2581179841)SALU

MGRANEPPRLPGGSFAAWSRDRLALASRGADECGDVWQLEPGVYVAARAGVCEAVLHRAQ

DFPKPSSPLFPPFKRAGGAPMPQERAHARAARMRGLRPQAVAARIGEIAAGTDRIADQWP

TGRDVEILPLVRPVLAEIGVRYLFSEDAPVLLPFAWQLFVAREVLVRPSRWVWPRWVPTP

ARRFRTRRQVAFTNALRPIIRRRRTSQRLGDDVLGQMLQPSSRYGPLAEEAVLDTLPGIT

VATFETPSRAAGWILLHLARYPRAADRVAAEAALLPASPASTTSTHFDNLHYTQALVREV

LRLHPPSWLLTRRAPRRTQLADYTIDAGSTVLVCPYTAHRDAREHPEPDRFRPERWLDDA

GSPTKPGVFLAFGTGPHGCEGAALAMAMLTLMTAQTARRYHLSEPPGAEPGYRITTFEGL

ATAGLCLRATLRG

>CYP147F20(2581179844)SALU

MISGDNLRAFKRDGRIVVVGASLAGLRAAEALRGGGFTGSLTMIGDELGEPYDRPPLSKQ

VLTGWVPADNTTLPRRRDVDAEWLLGVPASGLDLTTNHVLLADGREVPFDRVLISTGVRA

RPWSVESEAALDGVFVVRTREHAEGLRRALAARPSRVLVIGAGFTGSEIASVCRERDISV

TVAELAPAPLVGALGAMIGEVAADMQRAHGVDLRCRVEVTQLEGDARGRFRRAHFDDGSA

VDADVAVVALGSIRNTEWLRDSGLAADEWGITCDTSCRALDVNGRVTDDVFAAGDVARCP

NPLYEHRLISLEHWANAVEQAEIAAHNMVSAQADLRPHLSVPVFWSIQFGVNIKSVGVPT

FADEVVVTQGSLEDRRFVTAYGYRGRVTAAVGFNNGKRLHHYRRLIELAAPFPPACPTPD

QPADRKPVPVDLPGPALLAQGATAVVTGHDLGERRVTAAPQHRQEQGRTSTTGTPGTLQR

IFDYSARADPYPLYAELRTTPVIRQEDGSYVVSTYREITDVLNDPHLSSDLRNLSRPMPQ

ADEGATSSFIRMDSPEHDRLRRMAMRHFGPPHAPGLVTGLEGFLTATVGSLIDNLAGKEQ

IDVVDDFASPFSVTVTCALLGVPREDEPRFHVWVNDLMNSIDYNPETDPKEKLDKGVRAR

KDVRQCLGELVERSHSRPGDGLLSQLANDDGPDGRMTDAEIVATARLLLIAGHETIVNLI

TNGMLTLLRHPPVFQRLCGEPDLIVPLVEELLRYEPPVHIIPWRVAYSDITVADTLIPKG

SQIMLMLASGNRDPNRFHEPDRFDPDRRDNQHLGFGSGIHLCFGGPLARRETQIALTELV

HRLDHPRLVADPPPYRRSPVLRGPLHLDIEQGCG

>CYP1192A1(2581179873)SALU

MRLRERPAGYDRARHGVHLLRTPEVIADPSVYIDAIAELGPLFFDEVGGMWVCSGYAEAV

EILRDHRTFSSVREHDQDAFQELGLHASASLSTMVHEQMLFMDPPQHKAIRSALAEQFTG

TRVRSRENDLRHIAARALEELPRAGVLDLVADFAAKLPSALVAQLLGMPGREAELTRWAE

AYERLLGSLSALPAAPDREVDAVLTDALSVLQNEARSRLHAPGDDVISSLTAPLVDRSPT

GEELFAVAANCIVLVGGGYQTLTHLVTSALLALHDDPGLEKQLRELPELIPPAVAEFMRI

NGSSQYVARKATTDVKIQGTLITGGESVLVHLAAANLDPRTFSAPRALDLTRHGPKHLGF

GSGRHTCPGAGYAERLAGFAIEGFLAKYPSYAPESEPRAISWGLHGNTRCLEHARVRVDA

EVIPAATVDIPAAETHDSTGGNGLSLPPAATTAAACWHEVFERQALLTPDAPAVQGPDDL

ISYRELDHWANALAHRLRHQGAQPGALVGIVMERSVEFVLTVLAVAKTGAAFLLADVSCP

RERLRTMLVEAEARLVVTDGSLPSSAFPVQIVGVGAKDFRPDAPLTGVSPGDTAYVVFTS

GSTGAPKAIAISHEATVNLHLAQHQIFGLEPRDRVLQFLSPNFDGCIADLTLALLSGAAL

IVAPSNQLTVGPPLVRLLASQRVTTAILTPSVWMTLPDQPLPELRIAAAAGERLPAAWAR

RWAAPGRRLLNLYGPAETAVLATWHECSPSEDPPPIGRPVANKRAYLLDHHLRNVPPGQR

GELWLGGLGVGRYLNQPDLMEERFIRNPHTTTDPASLLYRTGDICRQRPDGTLEYIGRRD

RQVKIRGQRVELDEVERVLESAPGVTACAVHEQDGRIEALAVPAGPQLDETAIRTYLASR

LHSAMLPSVFTAVTELPRTVNGKADHRHEPPGEPTVQSSRPSGPALPPDDHERRLSRITW

EVAQNFAQVLNLPLRQVQADSDFFTAGGDSITMAAFLARLESLAGAPVDTAALITAPTPE

QITTLLLNAGAPK

>CYP107EA1(2581179902)SALU

MTVRRSAVTGWQPGAAGLKEETQAAARGLPSAIIAVSPFPAPGQDHADRSHADGSADPST

TAPQTPEPVTMNRPATELPPRSAAPGRPDPASAADVHRPFDAGFFRDPYPVYARLRTLGP

VLKVVLPDGSHAWLVTREEHVRAAFTDPRLSVNKARSRNGYQGFSLPPALDANLLNIDPD

DHLRLRRLVSRGFTPRHVERLRDRVGTAAAHYADRLAERLAEHGTADLLAEFANPLPLVV

IGHLLDVPEADGRAFSRWVAAMHAPARPGDTAEAIEHIHRYLLELIRARRAAPGDDLLSS

LIAARDADDRLSEDELVSLAFLLLMAGTENVQHLISGGVLTLLRHPEHLAALRSRPELMP

DAVEELLRHAHPNQMAIRRFPTTSVEIAGVRIPAGDTVLLGLASAHRDPDRYPEPELFDI

HRADKSHLALGHGLHYCLGASLARMEIGVALGTLLDRFPGLRLAVSDGELEWRQSFRSHA

LRRLPVAVSAPAG

>CYP147B7(2581179978)SALU

MSTETLLERINAYASRPDPYPLYTELREHGVARQDDGSYLVGTYHEIAALLHDPRISSDV

RHRTHPDLRDRPEDLPPSFIAVDDPEHDRLRRLAMRHFGPPHSPGRIDALHDDIVDVARE

LVDGLRGRDRIDLVDDFAYPLPVTVICRLLGVPREDMPEIRGWTNTIIASLDRSPDEDPD

GRLRAAAQARVSMAGHLGALAQRRRERPADDMISALVHDDGPEGRLTDPELTTTLTLLTI

AGHETTVNLITNGMLTLLRRPEALERLRREPELMPSAVEELLRYEPPVQMLPQRTPLTDV

EVGGVTIPQGVPLFLVLASGNRDPLRFEDADHFDPARRDNQHFGFGSGVHNCFGAPLARL

ETQVALTALLHGLDAPRLVEDPPPYRHSPILRGPRHLLVTGDVAAGAGA

>CYP107L41(2581180070)SALU

MPENPQAPQDGRTPRASEDTTDPPAPRGSDIYTSPEARASAPAPAPGAPPLVDLRAYGQD

FVTNPYPYYAKLRAQGPVHPVLTPYDQAAWLVVGHEAVRTALADPRLGKDWSSANLPRTD

GDGVPLFTNMLDVDPPHHTRLRKLVAKEFTSRRVEALRPRVQRLTDELLDAMLTASDDRA

DLIEALAFPLPMTVICELLGVPSMDRDAFRGWSHELVSPTSPEAAQQAVEAMSGYLTTLI

ASLRREAGDGLLADLIRTSDEDGDRLSPEEVIGTAFLLLVAGHETTVNLIANGVRALLEH

PAQLAALRADDSLLDNAVEEMLRYDGPVETATWRCAAEPVELGGTVIPAGSAVLISLASA

SRDPERFADADDFDLTRDPRGHAAFGHGIHFCLGAPLARLEGRIALRSLLDRCPGLALDD

AAEPATWRPGLLMRGTDRLPVRWDR

>CYP1191A1(2581180088)SALU

MTGPDPHTTAPRPCAEAALPPLGSPDPWPGYAALLACPGLHHEQAEHTYFAARHSDVHHG

LRHPDLAVGFPFRATRQLFGPTAIDLDAPRHRPARQQVSWFTTRHMPTWNRTAVIPVIDD

LIERAATDTPLDVIKTFAEPLPTRVICRILGLPDHEWPWVWKQLRPVIRHIADPRTGMQA

ALASRDILADRLRHAVRTGVPHGSLLQRLCANAPSDSAVDRTAEPIRTALLLLAAGTETT

AAAVGNLLWCLQRRPHTWDEVAAGTIPAEAVVTESLRLHPPLHSTVRFARRDLTLGDTAI

PKGARVQLLLAAANRDPARIGASPSWDPHRPPQAHHAFGGGPHACVGAQLALTEMKFLLT

ALTQRFELAGPTHPAGRFRAGPFHYPTELPMRLIPRTTPA

>CYP147F15(2581180150)SALU

MTLGTLPAQITDYANRADPYPLYAELRRTPVRRESDGTYLVSTYYEVRSLANDPRLSNDT

SHRSPGYARIGQPDEETGLPPSFIFTDPPLHDRLRDTINRPFGPPHSPRFLDDLRGDLAK

VVTELLDAFEGKDEVDIVEDFSYPLPVTAICKVLGVPREDEPRFHGWADALASSLDPQAG

EDGLEKAQRARQELGSYLTDLIETKRRHPGPGMLSALAPEMTPADLEATAVLLLVAGHET

TVNAITNTTLTLLRHPDVLARFQREPALAVPLIEEVLRYEPPVQFVPWTTALADIDVADT

TIPEGSPVWLMLAAANRDPKRFPDPDRFDPDRKDNEHLGFYTGIHYCFGAPLARMELHVA

VPELFRRVTFSRLLEDPPPYRANAVLRGPRHLPVAIEGLTA

>CYP107CJ2(2581180204)SALU

MTIQEPPLPAAEQKPRPEPLPDPVPLTGCPYKSNPYPLYERMREAGPVHRVLFPSGVQAW

LVTGYDAAHAALNDDRLGKNHDRGNERWRARASIMPEPQHSQLQVHLLHQDPPRHTRMRR

FVTDAFTPRRIESLRPRFQELADALIDALPESGPADLVAGFAAHFPFQVLAEVIGLPHHL

AARFDRDWGKVVQPVGPTDPGRPLYEARLHGLQSYIAEVVAHKREHWDDDLLSRLVVARD

RRELSQEELDSMIFQLLVAGQEPVTNQITTALIALFRHPDQLARLRDEPDLLPRAVEELL

RYDSAFELTTWRFLDQDDNLHGTDIPAGDSVIVSLCAANRDPRRFPDPDSLDLDRTPNAH

LAFGHGIHFCPGAALARAELQVALDALLTRLPGLHLAIRDEDIEWIPAVLGRGTNHLPVG

YDRRR

>CYP107EB1(2581180323)SALU

MDPALIADPYGGFDRLREEAPLVLGRSADSTPTWYATRYDDVRAVLADPRFVVDPELTPG

TEAVDNRNRMLDMLELPKEFHPYLSESILDVDGDRHTRLRGLATRAFTARRVNALRPRVE

AITASLLDGFGESVELISEFAYPLPIAVICELVGIPEEDRGLWRTAGSALTSIAPGSKGA

AAHELIAYTHALVDQRRAAPADDLISDLVKVQDEDGDRLSDVELVTMLLGLATAGHETTA

NLVGNGALALLSHPDQLEALRRTPELWPTAVDELVRSCGSILITQLRYATEDIDVGGQTI

KAGEAVQPVVVSANRDPREFSRPECLDVTRRSVKPGDGHVGFGLGAHYCLGAALARQECE

VALRGLFDRFPRVALTTDEHTWVPVPGLRQLASLPLTLY

>CYP163B9(2581180394)SALU

MTTPALDVVDLGDPATFADHDLDAFWRTLRDTYPVYWNPPVDGRRGFWVLSRYDDIMAAY

RDDVHFTSERGNVLVTLLGGGDAGAGRMLAVTDGHRHHELRKILQRVLSPRVLSEVAAAV

RVNTRQLIREAVEAGGCDFAEQIASRIPMTTISNLLGVPEQDRDHLLAQTKAALSTDAED

VDEVDSEMARNEILMYFMDMVEERRESPGDDVISMLVASSIDGVPLSDEDIVLNCYSLII

GGDETSRLTMIDGVHTLAAQPEQWRRLKHGEVAIDTAVDEVLRWASPTMHFGRSVVGETE

LHGVQLRPGEIVTLWHASGNRDERIFDRPGTFDLGRTPNKHLAFGYGPHFCIGSYLAKVE

IAELLMALRDFTTGFETTGEALRIRSNFLTGFSSLPVRWRPDHSGMKEVD

>CYP105AA13(2581180404)SALU

MRQTVPVPHGLPMDRDANPFDPPRALTRLRAARPVSPLVFPDGHEGWLVTGYEAVRQLMA

DTRFSSRLDLGVVHVPYETPGMPVPTEPSPQIPGVFIAMDPPDHTRLRRRLTGAFTVKRM

KQLEEHIVEVTERQLDAMARLAPPVDLVREFALPVPSLVICELLGVPYEDRDTFQSNSAK

FLVKEQTLEEKMAAYGALTTYLAELVTSKRAAPGEDILSDLARHDDLTVEELTGIAFLLL

LAGHETTANMLALGTFALLEHPDQLAELRADPDLLPDAVEELLRYLAVGDVFYRYATEDI

ELGGETIGKGSTVVVSLLAANHDPRRFEHPDTLDVHRKARGHLSFGHGVHQCLGQQLARV

EMRAGFAGLLRRFPTLELAIPANEVKLRTDMNIYGVHELPVTWTETAG

>CYP154A17(2581180466)SALU

MPQQSPHVLDPAGRDRRTEDASLRARGPMARVDVLGEEAWAVTDPELLRSLLLDDRVSKD

PHRHWDRFPDRTADWPLNLWVAVENMFTAYGAEHRRLRRLIAPAFAARTITALEPDIERF

TRELLDDLATARPGEAVDLRERFAAPLPIRVITHLMGLPAHLLADFRRAVNGVFATDVTA

EAAAANARDLYAALDALLALKQERPGDDLTTRLLQARDPEGQGHGLTPQEVRDTLLLVIS

AGYETTVNLIDQALVALLSHLEHLAAARSGALPWGDVVEETLRWQAPVPLLPMRFATTDI

PVADGIVIRKGQAILAAYSAANRHPALHGPDADRFDPTRPDKTHLSFGHGVHLCLGATLA

RLEATTALRMLTARFPELRLAVPADALVPLPSFLTNGHVNLPALVGPEAGA

>CYP107EC1(2581180474)SALU

MSDLASFDVTNPLFKANPHDFYAGLRESGPAHMVHLPGYGDVWLVTGYADARAALADPGL

SKAPANVPASLRNAMVEAGERDGFQLMAHMLNSDPPDHTRLRKLVVRTFTARRVRALRPR

IQEITDALLDEVVAKGSADLMDDLAFPLPITVICELLGVPAEDRDDFRRWTSILVSEDEA

LRPQLQDTFAQLNAYLTALVRHKHAAPDDGLLSALVSVAEDGDSLDDDEVVWMAFLVLVA

GHETTENLIGNGMLALLSNPEQLAALRADASLLPTAVEEMLRYEGPVETTTWRFTTRPVE

IGGVTIPADQTVVVVLASANRDPSRFADPGRFDISREDNQHVAFGHGIHYCLGAPLARLE

GQIAIGTVLRRLPDVRLAVDEGDLQWRLGIVMRGLHNLPVRFTPSGPEH

>CYP147F17(2581180478)SALU

MTSVALLRQVLDYANRPDPYPLYAELRRTPVVRDEAGPYLVSTYWAVKGLLHDPRISSDA

RNLTPEAAEATGQEQDPNLPPSFLRLDPPEHDRLRRLAMRPFGPPHTPRRVFEMHGELAG

IVTGLIDGLRGRDAIDLVDDVSYPFPVTVICRILGVPREDETHFHEWADTIAAGLDPVGT

PEERAAKMQDVQQARRDLAMYLAGLIEERRRSPRDDMLSALATEHGPDGQMSPVEMITTS

VLLLIAGHETTVNLITNGMLTLLRHPDVLQRLRTEPALAGPLVEELLRFEPPVQMLPQRT

TLAEIEVAGTVIPKGAAVYLMVASGNRDPQRFTDPDRFVPDRPDNQHLGFGHGLHSCFGA

PLARLEAQLALTELVRRLDAPQLVEDPPPYRQNAVLRGPRHLPLTIGGIRD

>CYP1064A3(2581180493)SALU

MTSGTGRQTGGAARALPGPEPRADGGAGAIAAAGGLHAYQLRLHDAYGPVVRFQLPGTEL

AVSISDPVLLEATAGLDERPVRPFAFLEPLCEAGNLQVLPAAEHGPWRHLLLSVLAGRPS

HERHFGQFTALTTALADRWAEQADRQPVALQRDLTALSLRMICAYAFGGEVTDPEGVVTA

FEEVLTEHLGRLYEVPGAVGPAERAERAERAAAALARLRATVDRVVAAHRRAGRTDRSDL

IGALVAAGERPARIRDTVMMIMLAAHHTTGVAVSWTLYLLGRHPEVAARVTEEVDRVLGD

RAAPEYADLRRLTYLGMTLKEAMRRYPPGPYGARETAEDLAVGDYLIPAGATVFYPFWAV

HMNPKYWPEPEKFVPERFTPEAVAGRPRLAHVPFGFGPRSCEGAALAVVEAELVLAVLLK

RFRFRPVAGDEVTPVERFVLWAADDIRMLVSPRTPG

>CYP105H9(2581180554)SALU

MTHSDPVVVDFPTRKPGVPFPPPDYDGYRDHQGLVLSRLPNGARAWLVTRHEDVRAVLTD

SRISSNPSHKGFPNVGTVGVPTQEQIPGWFVGLDSPEHDRFRKALIPEFTVRRIRGLRPA

IERTVEERLDAMLAAGNSADLVADYALPVPSLVISTLLGVPPSDRDFFESRTRTLVSLRA

STDAQRETAVKELLRYIKRLVGIKAKWPGDDLISRLLAAGSIAPHELSGVLMLLLIAGHE

TTANNIALGVVTLLRNPQWIGDDRAVEETLRFHSVADIVSLRVAVEDVEIGGQLIKAGDG

IVPLIAAANHDTSAFECPHMFDPSRSARHHVAFGYGIHQCLGQNLVRVEMEIAYRKLFER

IPDIRLAVPDEGLSIKYDGVLYGLEQLPVRW

>CYP161A7(2581180560)SALU

MSSPHRDLPSLDLETPALLRVSPLLRDLQERGPVCRVRTPAGDEGWLVTRHSVLKQLLND

ERIGHSHPDPANAAQYVRNPFLDLMIADTDAETARRTHTESRRLLAPMFSARRVREMEPR

VAAVVDAVLDDFTAQEPPGDLHGGVSVPVARTVLCDIIGVPPQNREHLTALLSQTAVLGD

REGVQRTQRDLYAFVGGLVEHKRGEPGQDIITRLTEGGLSDERVTHLAVGLLFAGLDSVV

TIMDHGVVLLATHPEQRAAALADPDVMTHAVEEVLRAAKAGGSILPRYATEDLTVGGETI

RAGDLVLFDFSLPNFDERAFDEPERFDVTRSPNQHLTFAHGMWHCIGAPLARIELNTVFT

QLFTRLPDLRLALPAGELAENEGRLSGGLSELPVTW

>CYP105AC9(2581180595)SALU

MPHDSSTPPVPSPAPSPAPSTPGAPAPAGTPRPEPEPLAALPVTRPAGCPFSPPAAFAEL

RAEQPLRRMRYPDGHLGWLATGHSVVRAVAADPRFSSRYELAHLPFPGMADVTLPPAPVG

DLTGIDPPHHTRYRRLLMGKFTVRRMRELTSRVEQITAEHLDAMERQGPPVDLVAAFAHP

VPALMICELLGVPYADRDRFQHHAMAASGTGGSLEDQYAAMAALQEFVRELVQVKRARPT

DDLLSDLTTSDLSDEELAGIGSFLLGAGLDTTSNMIGLGTFALLSHPEQADALRADPGLA

DQAVEELMRYLTITHTGIRVALEDVALDGQLIRAGESVTLAVQAANRDPARFPDPDRLDL

RRRATGHLAFGHGIHQCLGQQLARVEMRVAFPALLTRFPSLRLAIPPAEVPLRTDLTMHG

VQRLPVAWDA

>CYP107E14(2581180604)SALU

MTTAKTAPLSYPFNIAESLDLSAEYEKARNRPGLLKVQMTYGEPAWLVTRYAEARFVLGD

QRFSRAEGIRHDEPRQSEGSRNSGILSMDPPDHTRLRTLVAKAFTVRQVEKLRPQVKELT

RELLDELEAAGPPADLVDRYALPIPVAVICRLLGVPTEDRPKFRTWSDAALSTSSLTAEE

FDANREELRAYMGNLIEQHRREPQDDLMTALIDARDVNDRLTELELVDLCVGILVAGHET

TATQIPNFVLALLDHPDQLAVLREQPDLIGGAVEELLRFVPLGSGAGQPRYATEDIDVGG

TLVRAGEPVLVAMGAANRDALRFDGPGKLDIRRTGNQHLGFGHGVHHCLGAPLARLELQE

ALSALITRFPGLHVAGDVEWKTEMLVRGPRVLPVGW

>CYP184A8(2581181325)SALU

MSLTNSPTPADPEAERAFRQPLPTAIPGPAGLPVIGSLLDLRRDSLGAFLKAQREHGDVV

RLEAGPPGLRSVFYAVFAPEGVQQVLGTQAANFRKDHPLYEEVRQSFGNGLLTSQDDDYL

RQRRLVQPLFTKRRVDGYAAAVTTEADAVTARWRSVEGDVVDLVAEMNRLALRTVARILF

GLDVEAAVETIHRCAPVINDYVVRRAYTPLKVPRDWPTPRNLTHRKVTTELNALCDRIIA

ERRTASATGTTVTPDHNDLLSLLVAAGNEEDGTLDATEVREQVLIFLLAGHETTATSMAF

ALHLLARHPAEQTRIRAELTRVLGDRTPTAADLDRLPRLTQAFKEAMRLYPAAAVVGRRA

VEATEVAGHRLPAGADVVVAPWVTHRHPGLWEDPERFDPDRFAPEREAERHRYAWFPFGG

GPRACIGQHFSMLESVLALATLLRSHELTAVDQDVPVAAGITLQATGPARVRLRAL

>CYP125A33(2581181976)SALU

MHCPALPEGFDFTDPDVYQSRVPLPEFARLRQTAPVWWNAQPHGIAGFDDDGYWVVTRHQ

DVKEVSTKPEVFSANLNTSIIRFNQGISRDQIEVQKLIMLNMDPPEHTRVRQIVQRGFTP

RAVRALEAALRRRAEQIVEEARHKGSGDFVTDVACELPLQAIAELIGIPQEDRARIFDWS

NKMIAYDDPELAITEEVGANAAMELISYAMNLAAARKECPAQDIVSRLVAAEHDGNLGSD

EFGFFVLLLAVAGNETTRNAITHGMHAFLTHPDQWELYKRERPATAAEEIVRWATPVVSF

QRTATQDTELGGARIKKGQRVGIFYSSANHDPEVFDRPEVFDITRDPNPHLGFGGGGPHF

CLGKSLAVLEINLIFNALADAVPGISLAGDPRRLRSAWLNGVKELQVHYR

>CYP107EA3(2581182153)SALU

MTGQSVLKPFVGEFFANPYATYARLREVSPICRVELPDASPAWLVLTESDVRKALIDDRF

SVDRTHSRASGYQGFSLPSALDANLLNLDGDTHLRLRRLVMKAYTRPRINEMRQGVVQAA

EELADRIEGASTFDLVSEFANLLPIRVIGDMFDVPEEHRRPFSQWVGAMFGSQGPQQVRD

AINSIHEFLLHLVAERRREPGQDLLSALIAARDDHDKLTEDELVSLAFLILSAGSENVQH

IISNGIHTLLQHPDQLAQLRCEPSLLPAAVEELLRFAHPNQMAIRRFPTEPVSVRDVTIP

AGDTVLLCIASANRDPARYPDPDRFEIRREDKTHLGLGHGVHFCLGASLARMEAEAALGA

LLNRFPHLSYATPVEELQWRPSFRSHALKALPLSPK

>CYP1240B1(2581182228)SALU

MTSPYEPGAAPPPSACPGQAVPPSEAAAPPPGCPAHAGPQRAAAHPEAHAPVKLYGPDFA

ADPHRIYARLRQYGAVAPVEIAPEVPALLVTDYRAALELLNDDATWSKDSRAWMQTVPAD

SPVMPMLHWRPNVFYSDGPAHVRYRDAIVDSFKLVEPHELRARVHHAADTLIRRFGDRGE

ADLIADYARLIPLLMFNTLFGLPDSYSDRLIAAIAGMMEGNSPEEATAANEAYTQYIMEL

VGAKKAQRGPDLTSWMMDHANDLSDEELIHNIILVMGAGNEPLANLIGNALARMLSDDRY

YNTVSGGALTVHDAINEVLWNDPPMANYSAHFPVRDVFFHGTWVRAGQLVMVSYAAANSQ

FDSTGAHGPESGSGSHLAWAAGPHACPVKRHALLIAITAIERLTAWLSDIELAVAPAELT

WRNGAFHRALAALPARFTPITPDQAGATPWQNSDRSPSSSTRPAPTSTAKETASAH

>CYP154A21(2581182229)SALU

MTGHALLKELLTDDRISKNPRDHWPEWQRPEIRGSWLQSWIGVTNMFTAYGADHRRLRKL

IAPAFTARRTDAMVPRVTQIVGDLLDGLAARPAGEVVDLRESFNHPLPMQVICELFGYPE

GAPRGELARVVSEIMDTTATPEQATATQAAVAELLGSLVATKRAQPADDLTSLLVAARDD

EGQGMTERELLDTLLLVIGAGHETTVDLLGNAVFALLTHPEQLKLVRDGAVSWHDVIEET

LRWTPSIASLPLRFAVQDVALPHGEVIRKGEALLPMYAAAGRDPEQHGPAAATFDVQRAS

QDHLAFGHGVHHCIGAPLARLEARTALPALFERFPDMQLAVPAEELQPAGGFIAGGLASL

PVRLTA

>CYP107U17(2581182726)SALU

MTCCVAWGGCGTGYGREVQQTSDARQENRSDSHPEARPEARPAGDPPTGCPAAAGPSPAL

FSWEFAADPYPAYAWLREHAPVHRTRLPSGVEAWLVTRYPDARQALADARLSKNPVHHSE

AAHGKGKTGIPGERGANLMTHLLNIDPPDHTRLRRLVSKAFTPRRVAAFAPRIQELTDRL

IDAMIEKQHGGQRGSADLIHEFAFPLPIYAICDLLGVPPEDQDDFRDWAGMMIRHGGGPR

GGVARSVKKMRAYLAELIHRKRADLGDDLISGLIRASDHGEHLTENEAAAMAFILLFAGF

ETTVNLIGNGAYTLLRHPAQRELLQKSLAAGDTELLGTAVEELLRYDGPVELATWRYATR

ALTLGGQRIAEGDPVLVVLAAADRDPARFDEPDVLDLTRRDNPHLGYGHGIHYCLGAPLA

RLEGQTALATLLTRLPDFRLAVEPDDLRWRGGLIMRGLRTLPVEFTPEPSRPEAIP

>CYP130A10(2581182790)SALU

MAAQEAPQAPVSGPAAFTPCAGEGWRAPWDMYAALRDRDPLHHVDDGDYWVLSRYADVLA

AARDTERFSSAGGLTFTYGERERLGITDAAPMVMLDPPEHTDFRRLITRGYTPRRVAAIE

PDVRAFVRDRLDRIAGLGAGCDIVAELFKPLPSFVVGRYLGVPEADRGRFDGWTHAIVEA

NALGDPLAAVEAVGGLFGYFTELVARRRAEPADDTVSDLVRLLPDDDTALLRILGFAFTM

VAGGNDTTTGLLGGAAELLTADPDRRRALLDSPARLPAAVEELLRLTSPVQCLARTVTAD

TTLHGRTVPAGRKVLLLYGAANRDPRAFGPDADRLDLTRDGPQHLAFTHGPHHCLGAAAA

RLVARVALAELLARFPDFAVDAAGGTFADGHYVRRYATLPFVTGRG

>CYP105B1(2581183615)SALU

MTTAERTAPPDALTVPASRAPGCPFDPAPDVTEAARTEPVTRATLWDGSSCWLVTRHQDV

RAVLGDPRFSADAHRTGFPFLTAGGREIIGTNPTFLRMDDPEHARLRRMLTADFVVKKVE

AMRPEVQRLADDLVDRMTTGRTSADLVTDFALPLPSLVICLLLGVPYEDHAFFQERSRVL

LTLRSTPEEVRAAQDELLEYLARLARTKRERPDDAIISRLVARGELDDTQIATMGRLLLV

AGHETTANMTALSTLVLLRNPDQLARLRAEPALVKGAVEELLRYLTIVHNGVPRIATEDV

VIGGRTIAAGEGVLCMISSANRDAEVFPGGDDLDVARDARRHVAFGFGVHQCLGQPLARV

ELQIAIETLLRRLPDLRLAVPHEEIPFRGDMAIYGVHSLPIAW

>CYP107DW1(2581183840)SALU

MSEVIDLAELAATADLERELARLAAQHGIIRTRQLNQQETWTVLGAGLTRELLSDPRLSN

DVHTHAPHGALVPGLQVMLLEQDDPGHARYRRLVSAAFASKAVRQLEPRIVEISRQLLDK

LGDSGTADFIDAFTYPMPLEVICDLLGVPGEDRDPFRKWAMDISAAPSLEAMQTSAGELF

AYCIGLIGAKREQPTEDLLSELIAARFEDGTGLSDEELSSFAAVLLIAGHDTVTNLLANA

LHDLLTHPEQLAALRADRSLVNQAVEEALRFRGSAMTTVNRVALEDIEAGGVTIRKGELV

RFLLNAANRDVEVREDGHAFDIGRATAQHVAFGMGPHFCLGQRLARQEATIALNEILDRF

PKLELGVSRAEVRWLASDAIRGLEELPLRYARETA

>CYP157B17(2581184031)SALU

MNDPQHSTTGGCPLRAGASGTDAVPLYGAGLSGDPARLYREMRARHGTVAPILLDGDVPG

WFVLGYRELHQVTSNPELFARDSRRWHAWDQIPADWPLLPFVGHQPSVMFAEGPEHRRRA

GAISDALTAIDQFELRQICERLADGLIDAFAGSGEADLMARYAARLPLLVIAELFGFPHT

EVPELAADIAASLNEDEGAIAAHQRVAERMQRLVKAKRSAPGADVPSRLLAHPAGLAEGE

VVIDLLVVMAAAQQPTANWIGNTLRLMLTDDRFALSLSGGRRSVGQALNEVLWHDTPTQN

FIGRWAVRDTQLGGHRIRTGDLVILGLAAANTDPQVRPDFSDAGSDGNQAHMSFSHGEHS

CPYPAPEIAEVIAKAAVEVLLDRLPDVMLAVPTEDLVWHPSLWMRGLVSLPVEFTPAYTP

IPAMGTAPARRS

>CYP159A12(2581184032)SALU

MNREAPDILSPEFAADPYSAYRVLRDDFPLLHHEATQSYVISRHEDVERAFKDPVFTTDN

YDWQLEPVHGRTILQMSGREHAVRRALVAPAFRGNTLEQSFLPVIERNARELIDAFRGTG

SADLVADFATRFPVNVIADMLGLDRADHDRFHRWYTAIIGFLGNLAQDPDVTEAGLRTRD

EFAAYLLPIIRERRTAPGDDLLSTLCAAEIDGTRMSDEDIKAFCSLLLTAGGETTDKAIS

SLVHNLLRHPEQLAAVRADHALIPRAFAETLRHTPPVHMIMRQAAEDVTIGGGLVPKGAT

LTCLIGAAGRDERRYTDPDVFDIHRTDLSTTTAFSAAADHLAFALGRHFCVGALLAKAEV

EVGVGQLLDALPDLTLAPDAHPREQGVYTRGLAALPVRFTPAG

>CYP105AK8(2581184660)SALU

MTTLRSRIFAWAGRLYLARTRKKGFDLSRMSFLPDSVLMPLRRDGLDPVPDLADVREHEP

ISKLPVPIASNVWLVTGYDEVKAVLGKADAFSSDFTNLIGKAGAGAEQNPGGLGFADPPV

HTRLRRLLTPEFTMRRLGRLTPRIHDIVEERLDAMERAGRNGDPVDLVAHFALPIPSLVI

CELLGVPYEDRADFERLSAARFDLFSGANASFGAISESLSYFRDVVKKQRENPGDGLLGM

IVREHGDSVSDEELAGLADGVLTGGFETTASMLALGALVLLQDPQHFAALKDGDDVVDRY

VEELLRYLTVVQVAFPRFAREELEIGGVQIAAGDVVLCSLSGADRDGELGPEMEQFDPHR

AKVPSHLAFGYGIHRCVGAELARMELRAAYPALVRRFPTMRLATRPEDLAFRKLSIVYGL

DSLPVRLDA

>CYP107B6(2581184688)SALU

MSNSSLPHADAFTAESLRDPHALYAKMRDEAPVQKVVLPQGLAVWLLTRYDDVRAALSDP

RLRSDKSDVDGVLRNHLVSQEARESWVDELSGNLLNTDPPDHTRLRRLVNRAFTPRTVAA

MRSRIEEVTDELLDALPRGSEVDLLASFALPLPIIVICDLLGVPPEDRGVFTDWSNALLS

SADAAETAEAGQKMFAFLGALLAEKRARPADDLLSGLVQVRDEEDRLSEEELISMALLLL

VAGNESTVNLIGNSVLALLRHPDQLAALRADPALLPGAIEEFLRYDGPINTATFRSTAEP

VTFSGVTIPAGELVVVSLLAANRDAGRFADPDRLDVTRPAGGHLGFGHGVHFCLGAPLAR

MEGEIALGRLLARFPDIKPALALDELTYRFSTIIHGLEKLPVIV

>CYP1189A1(2581184711)SALU

MTTTEQIAAFADVPGPQGSGVAGVAPEFLRDPIAVLARAHRDHGDLVAFPFGPRKGPLGK

VVVAAYHPDAARQVLTETERTIGRGPSSTQVLDDMIGRNLMTTDGAEWRRQRRTLQPLFT

PKRVAQYTDLMAAEAARIVAEDVPAGTTADRVDLHRLMLRYSLRVVGRALFSGDIDYTAP

ELHKLIPLTNELIIGRTTQLLKPPLALPTPRNRAFLRTKAQLYALIDRILARSDAEAGGA

ERDDIVTRLRTARDPETGAGLSDAEIRDQTLLMMMAGHETTATALTFALHLLGRHAEVQQ

AVADEALEYTRGGGTPAEFAQQRESLARASLLESMRLYPPVYMTEHLATADIVLGGYRVP

AGTAVFLSPWVTHRHPEFWPEPERFDPHRFVGEHDRPRYAYLPFGGGPHVCIGEHFALLA

ATVLLEAVVRKFRIESLEESISYQQTGNLRPDKPVWAALTAR

>CYP1189A2(2581184712)SALU

MSTTAQLRPFHDIPGPKGHALAGLLPDFNADPLGFLTRGFQEHGDLVAYRFGPRKGPLGK

TILAVYHPDLVHQLLMDTERTFGRDTDGFRATYELVGRGLMTTEGPYWRRRRHILQPLFT

PKRVARYTELMAAEAERIIAEHEQFEGTEIDLHQAMMRYSLRVVGRALFGGDLDDAEAEL

HALIPDANRGIMARTTQIPKLPLKFPSPTNRMFVRTRDSLYDLIQRVIERSGSGDAASSE

DNIVSRLREARDPESDEPLTEQEVRNEALLLFMAGHETTAMGLTFGLHQIGRHGDVQKAI

AAEIDAHHAAGGTGAEYAQNRDTLGRAALNEGLRLFPSVHMTERVANEDLELNGYHVPKG

TSVFLVPWVTHRHPEFWPDPERFDPDRFVGKQADRPRYAYFPFGGGPRVCIGEHFALLAS

SILMEALLRKYQITSHDEQISMKVLNSIRPDRDVRTTFTRR

>CYP107F9(2581185164)SALU

MAKEADPTVWNCPFDYAEALEFDPTLRRIMTEEPVARIRLPYGEGEAWLVTKYDDVRTVT

TDRRFSRHAIVGRDFPRMTPEPIVQDEAINVMDPPASSRLRSLVAKAFAPKQVERMRSRT

QHVVDELLDRMVENGAPGDLMENLASPLPLTTICEVLDIPEGERAQLRGYARTMMNTSLA

NKDNAIRAKADMREYFTELTARRRRDPGDDLISALATARVGDEVLDAKELTVMAMVLLIT

GQDTTTYQIGNLSYTLLTRPKDLAMLRERPEALPQAMEEMLRFIPFRKGVGIPRVALEDV

ELSGVTIRAGDIVHVSYLTANRDSEKFERPDELDLSREATGHMTFGWGAHHCLGAPLALT

ELQVALSTLLQRFPDLKLAKPAEELRWNTTAIWRYPLALPVVW

>CYP163C3(2581185203)SALU

MTAMTSRPEPAPDALGDVDLADPLLHARHDLGPLWRRLRTEAPVHWQPESGTRPGFWVIS

RHADVVGVLNDPQTFTSERGNVLDTLLAGGDSAAGKMLAVTDGGPHKALRSALLKPFSPR

ALDVVVDSVRRGTRALVEEAVARGTVDFAADVAAHIPLAAICDLLGVPLADRRQIIELTS

SALSSADGVPTEEATWSSRNGLLFYFSELAAARREKPLDDVISLLVTKEIDGRPLSHEEI

VFNCYSIIMGGHETTRFAMVGGVQALMERPEQWHALKTGQVSTASAVEEVLRWTTPALHS

GRTATQDVFLGGQFVEEGDIVTAWMASANGDERVFDRPDTFDLARTPNKHLSFAHGSHFC

LGAFLARAELAALLESLCDLVAVAEPAGAPGRVFSNFLSGFSSLPVTLVPEGRQAT

>CYP105AC10(2581185377)SALU

MTDPVTANMAEPVHTVSVLPTARQSGYPFDPPEELIQARRHGPISRYPFPGGRQGWLATG

YDLVRSVLADSRFSSRKELMLHPYMDIGDIRIPPAAPGEFVLMDEPQHGRYRKLLAGKFT

VRRMRSLTEHIGQVTADHLDAMESVGPPTDLVTAFAKPIPAIMICELLGVPYRDRGSFQE

QAATIFGGDTSAEDQMDAYTAIQEYLAQLVAAKRAHPTDDILSDLTGSDLTDEELMGVGL

LLLAAGLDTTTNMLALGTFALLSHPEQFAALHANPAIADKAVEELMRYLSVAKTFTRTAL

EDVELGGQIIEAGTTVILSFHTANRDPQRFTDPDTLDIQRQATGHLGFGHGIHQCLGQQL

ARVEMQVAFPALVKRFPTLRLDVPAEEVDLRPETADVFGVKSLPVTWDA

>CYP105D23(2581185704)SALU

MTGTIPFPQDRSCPYHPPTNYRPLRESGPLSHVSFYNGRKVWAVTGHAEARTLLVDPRLS

SDRQNPAFPIPVERFEAVRRVRTPLIGVDDPEHNTQRRMLIPSFSVKRTAALRPQIQQIV

DGLLDRMLEQGPPAELVSAFALPVPSMVICSLLGVPYADHEFFEDRSRRILRGGTAEESE

QARRELEGYLADLMARKETDPGDGLLDELIAERLRAGTLQHQELVRLAMVLLVAGHETTA

NMISLGTFALLEHPDQLAQLRSDESLMPGAVEELLRFLSIADGMLRVATADIEIAGHTIR

TGDGVVFSTSLINRDATAYPSPDELHVDRSARHHVAFGFGIHQCLGQNLARAELEIALRS

LFRRVPDLRLAVPAAEIPFKPGETLQGMIELPLIW

>CYP113D6(2581185809)SALU

MTNASTQWTFHEDQFWMRGELPPGRVCYDEKKGLWNVYGYAECLQVLGDAETFSSDLSIL

APEGKRQIFPGNLTTMDPPEHTKMRKIVSGVFTRGVVAALEPRIKAITHELLDQVEPGDA

FDLVEVLAHPLPVIVIAELLGIPAGDRHVFREWVSKLLENNQSFSTGEDTEELRKQREET

FVQINNLSTYLREHVEQRIAEPRDDLLTKLVQAEVDGERLSTAEIVNFAFVLLVAGHITT

TMLLGNTILCLDAHPTALKSVRTDRGRIPAAIEESLRLFAPLAALRRVTRKPARIGDVEI

PELQVVMVWTAAASRDLDQFRDPNTFDLDRGNNPHLSFGRGSHFCMGAPLARLEGLLALD

ILFDRFPALRCDPDQAPVFMPGANVMGVESLHLLT

>CYP157C28(2581185819)SALU

MTAFPSHQPGTTSASGPPQGCPAHARAGGTDHLARLFGPEVAHDAPGFFERLRREHGPVA

PVLVDGDLPAWLVLGYRENLDVLRTPTRFSHDSRIWHCFREGRVPADSPLMPALAWQPVC

LFMDGTEHERLRLAINESMARFDRRGIRRCVTRSANQLIDAFVADGRADLVRQFAEQLPM

LVLTQLLGMPDEAGPRLVEATRDLLKGSETAVESNAFLMAALEQLVARKRDAPGPDFTSW

LMSHPTRLTDEEVAQHLRIVALAANENTTNLTANTLRMVLTDPRFRASLTGGSMTLPDAL

EQMLWDEPPTSVLPARWATGDTELGGQSVRAGDMLLLGLAPGNGDPVIRPDRSVPMHGNR

SHLAFSSGPHECPGQDIGRAIVDTGIDVLLMRLPDIDLAVPEGDLTWVSHWIARHLRALP

VKFTPGVPASADASGDGADGPHDLRESAGGGPLVAGGVDASTPPAGAPRSRPSWWARLTR

WLSGQ

>CYP107X11(2581186015)SALU

MQNTAETGPAAPLDTTPLLDDPYAAYAALREAGPVHRITGTDGQPAWLVTRYDDVRSALA

DPRLSLDRRHATPGNYRGFSLPPALDANLLNMDPPDHTRIRRLVVKAFTPGRIEALRAPV

QRIADDLLDAMAARGRAELVTDYAGPLPITVICDLLGIPVERRRDFLAWSDALITPDPSR

PQAMKEAIGAMLEFYTGLIAAKRAEPGDDLLSDLIAVRDETADDPAGDRLSEDELTSLAF

LILFAGYENTVQLIGNAVLALLDHPERLAALRRNPAELAPAVEEFLRYDTPASLAIRRFP

VEDLDIGGVRIPAGESVLLSIASANRDPERFPDPDRLDPTRELSGHLALGHGIHYCLGAP

LARMEAEIAIGALISRFPELRLDVARDEVRHRRTIRARGLISLPVAW

>CYP105B33(2581186057)SALU

MTEMIDTTVPAAPAPLPVEPPSGCPFDPPAEFGALRTEEPISKISLPDGSWAWLATRYAD

IRAILGDTRFSSDTTLHGYPLSGMTGGGNQQNRGFIRMDPPEHTRLRRMVTREFMVKRVE

ALRPEIQRLTDELCDAMERRAGEPVDLIEALALPVPSLVISLLLGVPYDDHDVFQRLTGK

LLSRTIAEPEREAARGELREYLDALVTAKEKEPGDDILGRLIVEQQRTGEITHDDVAAFA

ALLLIAGHETTANMIGLSALTLMQDPDSAERLRQDPKLIRGAVEELLRFHSIIRNGPRRV

ATTDIEIDGQLIRAGEGVVVAVPSANRDATVFADPDRLDVGRANAQHHVAFGYGIHQCLG

QALARVELQVVIATLLRRFPAMRPAVPVEEIPFRTDMAIYGCHALPVTW

>CYP102G12(2581186071)SALU

MTDTIAPQTGDRASAVGVPVADLTATGISSTPLQQAMDLARIHGPAYVRKFGARETLFLS

SVDLVTEVSDETRFAKGVSVVLENVREFAGDGLFTAYNDEPNWAKAHELLMPAFALGSMR

TYHPAMLKVARRVTASWDRRMAEGRPVAVAEDMTRMTLDTIGLAGFGFDFESFSRDTPHP

FVEAMVRCLEWSMTKFARQPDADHTAADAAFRADADYLASVVDEVIAARAASGERRDDDL

LGLMLAAGEGEAAHQGPALDLANIRNQVITFLIAGHETTSGALSFALYHLLKDPVALRLV

QREADELWGDEADPDPTFEDIGKLAFTRQVLNETLRLWPTAAAFTRQARTDTVLGGRYPV

AAGSLVTVLTPMLHRDPVWGDNPEAFDPFRFTPEAEAARSPHAYKPFGTGERACIGRQFA

LHEATMLLALLVHRYRLVDHADYRLRVKETLTLKPDGFTLALAPRTPADRAAVRSGLAVL

PGGPAGAAAGDATDAAADEGLPTRVRQGTGLLLLHGTNYGTCREFAERLADEATALGFAT

EVAPLNAHAGSLPTDRPVVLVAASYNGQPTDDAAAFTAWLGTAPEGAAAGVHYAVLGVGD

RNWAATYQQVPTFLDDRLAALGGERLLPRAEADASGELAGAVRKFGAALRTELLIRHGDP

ASIGDRGADGADTGYAVTALTGGPLDALTARHDLVPMTVTEAYDLTADGWSRPKRFLRLV

LPDGVTYRTADHLAVLPVNTPQAVARTAAALGVDPDSVLALRPPTGRPVRDTLPIDRPLT

VRQLLTHHLELGMRPTPEQRALLAAHNPCPPERHALENLPDDDPRSLVELIEAHPALRGA

LPWPVVLELLPPLRTRHYSLSSSPAADPRHADLMVSLLPGGTGSTYLHAVRPGDTVLARV

QPCREAFRLDPDDDTPVILVAAGTGLAPFRGAVADRVAAGRTTPARLYFGCDDPDGDYLH

AAEFAAAERAGAVAVRPVFSARPENGHRFVQHRIAAEATEVWELLRAGARVYVCGDGSRM

APGVRDAFRAVHRERTGASEQESQAWLRELTAAGRYVEDVYAAG

>CYP155A6(2581186159)SALU

MDGEDRDYGRRKIERETPATGCPVSRAGDGSWQVRGYDEARAVLRSTTTVQAGLGIESVE

GLPSRIRRPVLYRDGPEHREHRRQTARFFTPRHVDQHYRDVMSRATRTQLDRLTANGRAT

LSDLAFNVAIEVACAVIGLTESRPGIKQRLERFFPEEFGTPGLRSLRGIYWTIRQSTNWL

RVHLGDVRPAVRARRRRRRDDLISHLLDEGCSTSEILGECLTFAAAGMVTTREFISAAAW

HLFSDTELLTHYQAVDEAGRIAVLNEILRIDPVIGRLSRRTTGPLDVPRNGDEPLTVPAG

ERIDILLDHTNLDERTVGPRPQRIRPGRTMREGARSPGLSFGDGPHKCPGTHLALLETDI

FLTSLFALPGLRMATPPTIGFIDGIASYELRHCVVEVDTVP

>CYP107Z13(2581186177)SALU

MTELTDSPFSEFVGKHPGEPNVMEPALLTDPFAGYGALREQGPVVRGRFVDDTPVWFITR

FEEAREVLRDQRFANSPAHSAGGGSADTPIDRLLEIMGLPEHYRAYLSGTILNMDAPDHT

RLRRLVSRAFTARKITDLRPRVADIAEDALRRLPEHAVDGVVDLIPHFAYPLPITVICEL

VGIPEADRPQWREWSTHLVSLRPELHPETFPEMIDHIHALIRERRTALTDDLLSELIRVH

DDDGSRLSDVEMVTLVLTLVLAGHETTAHLITNGVAALLTHPDQLQLLKSEPALLPRAVH

ELMRWCGPVHLTQMRYATEDVELAGVRIKKGEAVTPVLVAANHDPRHFADPDRLDLTRQP

AGRAENHVGFGHGMHYCLGATLARQEAEVAFGKLLAHYPDVALAVAPEDLQRVPLPGSWR

LASLPLRLN

>CYP156B17(2581186366)SALU

MQSHPEFQTPPPGCPAHAEGIKTSLHGTEFAAAPHAVYEQLRKHGPTAPVELSPGVEAEL

VTDYATALQILQNPDSFARDPRRWRALNEGRVPLDSPVLPMMMYRPNSMFSDGATHLRLR

QVVTDSLAKVDMHRVARHVDRVATYLVDQFSIRGKADLIGDFAQVVPLLVFNDLFGCPAE

LGDRLIVAISSLFDGIDVERANEEMAGALFELVALKRALPDEDMTSWMMQHPAKLSDEEM

VHQLALLIGAGTEPVQNVIGSSLLLLLSEDQFADGQHGGGVLVEDAINEVLWNSPPIANY

ATHYPVRDVEVSGSKLPAGAPVLISFAAANADPTLSATRQTFSKRAHLAWGAGPHACPAK

DPALLISVRAIEKLLNTLPDIELGVPQETLTWRPGPFHRALNALPSRFTPIRSDRRSSAG

YAAQDAAQPGGGAQDQGRRKRDGWWSGFLNWWKV

>CYP1035A12(2581186367)SALU

MEPALAAASVDRRSVVALFSRLRSARGQSNPLPFYTELQSMGEMVPAPWGGHLATSYRLC

HQVLRSRDWRVPDSGWRAAQADAVRWHAPASQQMGATMPMLNPPHHTQMRRPLGNVFDRA

ALQQMERSVERNAESLVDTFFEQLSGGTADFCALVGDELPVITVGEWLGLPSADFAVLRS

LTHDQVHTQELFPTPSQLAISDSATRNLRKYFTDLIRERRKAPGDDPVSRWLRMWDEFEP

DQDAADEAVYALALFMLLAALETTSHLLTTMMWLLLEHPRQVDWLRTHPEHIPGAIDETL

RYDAPIHMISRIAPEDTELAGVPVREGEMVQLMVGAAHHDPEQYAAPEVFDVRRKAPHLS

FGGGIHYCLGNALARMEATCVLTSLLRRLPVQGLRVAGTPTWAPRVAFRRLMELPVVRT

>CYP107AE9(2581186512)SALU

MNDPMESAEFSRDPYPLLAALRARGPVQRVRTGKGRTTWVVTGWAEARAALADRRLSKDT

ARYFANRPSGRDLAPAVSQSMLATDPPDHGRLRKLAMAAFTPAAVGRLEPRIREIAEGLA

AELGRSAGAAGGPVDLVEGFAVPLPIAVICELLAVPEADRAAVRRWSDDLFAAADPGTAD

RASHALAGYVAELIAARRAAPGDDVLSGLIAARDAGDRLSERELVSLAVLLVVAGHETTT

HLIGNGTLALLRDDALHARLRDDPGLLPAAVEEFLRYEAPVTLATFRYATEAFDLGGVRI

GAGDVVLVSPGGANRDPARFDEPDAVRLARSGAGGHLSFGHGPHHCLGAPLARAEARIAF

EVLLARFPGLRLAAGGGQGAGGSDGPDGVTWRRTRLMRGPAQLPVLLGPLREAPEGRG

>CYP251G1(2581186720)SALU

MTGAGVPGDDPRGGSPRGAAGVPGRVPLVGHAVPLRRDPLRFLCGLRQRGPVTKIYIGPR

PVHVVNSSDQVRELLTVQARSFDKGAMFDALRVPLGDGLITAAGDRHLRHRRLVQPAFHH

ERIARYARTMAERSLARSADWAPGTTRDLVPDINRLTLDVLLRTLFAAPHEPGLDAAVQD

WLTVKYHSMRLALSPLHAWAERVPLLPGWRPPDAGPLRRLVDVQLRIIDGYRADGRDRGD

LLSMLLLAGGPEGALTDAEVTDELITLFLAGTGTVSASLAWALHEISRRPDVQRRIHDEL

DTVLAGRPPACEDLPALVYTRQVLTEVLRLHPPSWLLMRRAVRPVSLGGVRLAPGDEVFF

SPYALHRDPHLYEDPEDFAPDRWPADAAAKAPRHTFLPFGAGSRLCIGEDYAWTELTLAV

AAFTAGRRLEPAGTAPVRTLVGTVLRPDRLPLTARPRPA

>CYP147F14(2581186856)SALU

MTTTQTPDTLRRILDYSSRADPYPLYAELRETPVARQEDGSFVISTYREISDILHNPHLS

SDTRNLSCPTEGMRAEGTPAFINLDPPEHDRLRRLAMRHFGPPHTPGLVTGMEGALTTAV

GHLIDDFAGKERIDVVDDFAYPFPVAVICHLLGVPREDEPRFHRWVDDLINSIDYNPKTD

PKEKLDKGVQARKDLRQYLGGLLERRHGHPGDDLLSRLANDDGPDGRMTDEEIVATANLL

LIAGHETTVNLITNGMLTLLRHPHVLQRLCEEPDLVVPLVEELLRYEPPVHIIPWRAAYS

DITVAGTTIPKGAQIMLMLASGSRDPNRFHDPDRFDPDRRDNQHLGFGSGIHLCFGGPLA

RREAQIALGQLVRHLDRPRLVADPPPYRRSPVLRGPIHLYVEQGAA

>CYP180A10(2581186884)SALU

MTGSTGGAGREPARSPDVPDVFDPRRYAAGLPHDAYRLLRDHHPVAWQDEPEVLGWPAGP

GFWAVTRHRDVVRVLKDARTFSSRLGATQIRDPDPADLPFIRRMMLNQDPPQHNRLRRLV

SRAFTPRRVDRFEDAVRDRARTLLASAVDAARAADGVCDLVGTVTDDYALRNLADLLGVP

PAERGLLLDWTRRVIGYQDPDEAGPAVTGASGRPVDPRSPAMLRDMFAFARELAAHKRRH

PGDDVMTVLAADRELAVPELEMFFFLLTVAGNDTVRSAAPGGLLALAGHPDAYAALRTGA

VPMGPAVEELLRWHPPVLSFRRTAAVDTELAGRRIRAGEKVVVFHASANHDERVFAEPGR

LDLGRAPNPHVSFGDGPHVCLGAHLARLQLRVLYEETCALLPAVAVAGPPPRLVSNFING

LKSLPLRVAT

>CYP1199A1(2581186937)SALU

MLQRSTASRGGRQVRFAPRIDALLRQHTGQDLFRLEPTTVGVGGADLMDALLRSRPANAE

ERPTFKPVLGRHVSRADAATYMQAVAADVRKALQRPLEAPVDLTGPWPQVPHAYLRDLVF

GRELLRFRVLVDRRLELTPKLTWSAVTSGAALLRRPDSTEPLSKLAALVLGATGFPDRRY

AMYLYRRVAAPICFTVAALVTNAVWLGAPFDDSVPNRHLLAEALRLLPPSWNILRVASPE

FTVLDTRIGPADDVLLLPLLSHRDPALWEEPDAFRPERWADLDPDDHPGYLPFGHANERC

WGRHMVLPLAERLLDLVRRDGLTVRPAQTVGRVELDGLMEVAEVRVTRG

>CYP161A6(2581187107)SALU

MSTPTAPPSLAAEARTVLRLSPLLRDLQSRAPVCKVRTPAGDEGWLVTRHSELKQLLHDE

RLARAHTDPANAPRYVRNPLLDLLVTDDVDGARAVHAEMRALLTPQFSARRVLDLAPKVE

ALAEQALAHLTAQGPPADLHDHFSMPFSLSVLCTLIGVPAAEQGQLIAALAKLGEIDDPP

RVQEAQDELFGLLSGLARRKRTEPEDDVISRLSRKVPSDDRIGPIVAGLLFAGLDSVASH

IDLGTVLFTQYPDQLAAALADEQLMRSGVEEILRSAKAGGSVLPRYATADVPVGDVTIRT

GDLVLLDFTLVNFDRTVFDEPELFDIRRAPNPHLTFGHGMWHCIGAPLARVQLRTAYTLL

FTRLPGLRLARPIEELGYSSGQLSAGLRQLSVTW

>CYP105H1(2581187109)SALU

MSTGAEAPTPAPQCPVAFPLRQPGRPFPPPEYAQYRAGPGPVRTELPSGTVWLVTRHEDV

RAVLTDPRISADPSRPGFPKASRTGGAPSQYEVPGWFVAMDPPEHGRFRKTLIPEFTVRK

VRELRPAIQQIVDERIDAMLAAGTSADLVESFALPVPSLVISSLLGVPRADRDFFEDRTR

VLVRLSSTDEERDKATQALLRYLGRLIQIKQRRPGDDLISRLIAAGTLSRQELSGVSMLL

LIAGHETTANNIGLGVVQLLANPQWIGDDRIVEELLRYYSVADLVAFRVAVEDVEVGGRL

IRAGEGIVPLLAAANHDDTVFAAPREFDPERSARSHVAFGYGVHQCLGQNLVRVEMEIAY

RTLFARMPSLALAVPVEELSLKYDGVLFGLHELPVTWK

>CYP105B22(2581187324)SALU

MPHTTIPDFPLDRAPGCPFDPPPHYAALRAQAPLVRVRIWDGQTPWLVTRHEDQRAVLAD

PRFSADPSRPGFPAPTAGFKAQGREEVQALSMQDDPEHARQRRMLIGRFTVKQVTAMTPR

LVQIIDDLLDRMEAAGPPTDLVAAFALPMPSLVISELLGVPQQDHALFQRTAGTLISRES

TVQEFAAARTELADFLGDLIRRKDDDPGDDLLSSLVVTRMRTGELTPALLVETAMTLLVA

GHETTTNQLALGTLVLLRNPDQLAVVRDSDDPARVASAVEELLRYLSITQNGLSRVATED

VEIAGQLVRAGEGVIVPNASGNRDAAAFRDPDRFDVGRPDVRGHLAFGYGTHQCLGQNLA

RKELQLAYPALLRRFPGLRTTLPDEDIRFKHDMIAYGVHELPVTW

>CYP107T3(2640830897)SALS

MAAPTLEQLAPAGHDMAADPYPVWAELRARRPVHRVTVPESGECWLVLTHEAARTALTDP

RLRNDLRHSSSWGSDGGHAIGRNMLQSDPPQHTRLRRLVAVHFTPGRITALRPRIEALCA

ELLSALPRQGTVDLVSRYALPLPVTVICELLGVPDVDRALFHIWSNSLVLPASQESAAAA

AAGLTGYLTELIDRVSASPDGTLLSDLAEAARVPAPLGAEASGPGSAPGLDREELLGMAF

LILVAGHETTVNLISATVHSLLSHPDQLALLRADPALMESAVEESLRLNSPVHASAFRFA

AEPLELAGTRIPAGDAVLVSLAAASRDPARFPGPDRFDLRRRAQGHLGFGHGLHHCLGAP

LARAETALALRGLLADRPELDFATDPAELPWRSSTLLRGLTELPVVVG

>CYP107P21(2640831082)SALS

MSVFAPESPQFVADPYPAYAELRAAGRVHWYEGSGQWLVPRHADVSALLRDRRLGRTYQH

RFSHEEFGRTPPPPEHAPFHLLNDNGLLDLEAPAHTRIRRLVAKAFTPRTVERLVPYVHR

LADELVEELVESGGGDLVSALAEPLPVAVIAEMLGIPEADRHLLRPWSAEICGMYELNPG

PETARRAVRASAEFSSYLRELIAERRGRPGEDLISALIAAHDEHLDDGGEERRAAPGRLS

EQEMISTCALLLNAGHEATVNTTAGGWWTLLRHPGELARLRAEPALLPTAIEELLRHDTP

LQLFERWVLEDIEIGGTVLERGSEVALLFGSANRDPEVFAAPDRLDLGRRHNPHLSFGAG

IHYCLGAPLARIELTASFGALLRRAPRLSLAAPPVRRPGFVIRGLSELRVAV

>CYP107U15(2640831538)SALS

VSEQVGRNGQAGEPGGPDARAAAPGSCPVAGAESPPDLFTWEFAADPYPAYAWLRTHAPV

HRTRLPSGVEAWLVTRYADARAALADPRLSKNPAHHDEPAHAKGKTGIPGERKAELMTHL

LNIDPPDHTRLRRLVSKAFTPRRVAEFAPRVQELTDSLIDSFAARGSADLIHEFAFPLPI

YAICELLGVPREDQDDFRDWAGMMIRHGGGPRGGVARSVKQMRAYLAELIHRKREDLGEE

SARDDLISGLIRASDHGEHLTENEVAAMAFILLFAGFETTVNLIGNGTYTLLRNPDRRAE

FQRAVAEEDTALLASSVEELLRYDGPVELATWRFATRPLEIGGQRIASGDPVLVVLAAAD

RDPARFPAPDTLDLARTDNQHLGYGHGIHYCLGAPLARLEGQYALGTLLRRLPDLRLAAE

PEELRWRGGLIMRGLRTLPVSFTPR

>CYP170B5(2640832088)SALS

MSVESALRSGSAPREHLPPPPLVPGGAPLLGHAWNLVRDPLDFLSRLRDHDEVVRLRLGP

RTAYAVTAPELVGALLKSPDYEVGGPLWETLEVLLGKGVATSNGQPHRRQRRMMQPAFRP

ERIADYAAVMAEEARATAARWEDGATVDVGAEMFRTAVRIVSRSLLEVDSIGEKADRISE

SLHTVFEGLYRRMVLSAGPLHRVPTPANRRFAGALADLHALVDEIIEERRAAGSGHEDLL

AVLLDATDESGAPLSHAEIHDHVVSLVVAGAENVASTLSWTFWLLTQHPVQESRLVEEVE

SVAPEGRPAFADLKKLVHTRNVITEAMRIRPAAWIFTRRSVAETSLGGYAIPAGADVVYS

VYAMQRDPRSFEQHLEFDPDRWLPERASEVPQFAMMPFSVGNRKCPGDHFSMTELALILA

TVAPRWRLTPVEGTDPRTRIGITLHPKRLLLRAERR

>CYP107DU1(2640833188)SALS

VSLPLMDEEFLTNPYPAYDRLQAGGPVHRMLLPSGLKVWAVVSYEESRKALAHPSLSKDI

DASVQLGYFDREMSEGGTPRLEHSSVGQHMLNMDPPDHTRLRKLVNRALTARSVKRLEPV

IEEISTALLDAVELRAAESADGSVDLVEEYTAKFPVEVLGRLLGITEEHFPVLVSLTAAI

MSNDNENAGANMARFGQHLGEMIRLKRADPGEDLVSALIQARDEDAKLSDIELISTVFLL

VVAGYETTVNMIGHGLRALLNHPGQLAELRADPSLIPGANEEFLRYEGPGNRATLRFATE

PFELGGVTIGTGEYVSVLLGSANRDGAQFACPHQLDVKRSTTGHLGFGHGIHYCVGAPLA

RLEMEIALRHLLTRFPDLELAVPDAELRWRKSFMMQGLERLPVRLHKG

>CYP105DB1(2640833404)SALS

MKTRPDPPVIPSDRGGCPFDPPAAYARLRAEDPVSPVTFQVAPGDPNGWLVTRHDLVREI

LADERFSHRNELLAHVVAPPFPMDEYKPVPSAPGSFAKMDAPEHTKYRRLLAGHFTLRRI

QAYEPELERLVDETLEEMAAKGSPADLVTDWAEVFSLRSVCSLMDVTPELMDGIAEHFGA

LMRLTYTLEEFIHHIESMDALIRPMVAERMAEQGEDFFGRLSATGELTEDELVNLAVLTL

GGSLDTTPNMLALSTFALLEHPEQLALLRERPELYEAGAVDELLRYLTISQMGSSRCAVQ

DVELGGRTIKAGQTVVLSLPAANRDPEVFTDPDRLDVTRIPRKHLALGFGAHQCLGQHLA

RSSMRIGLRKLFDRFPSLRLAKPAAEVPLRDRSVHYGVDELPVAWD

>CYP113Y1(2640833405)SALS

MSEVLDPAQAAAGLPDRAPTVEPDGGDTLLAWAARMRAEQPVWRDGSGIVHVFRHADVQR

ILADPGTFSSDTVGRLSGGERQAPRGTLLLLDPPLHGKMRRLISRAFTPGLIAGLEPAIT

ELTAELLDRAEGDRYDLVDVLANPLPVTVIARMLGVPGEDRMLFQGWADQLLNTDPDDPE

SVRAMEETAVGITAYLQTFIDERRRSPRGDLLGILVEAELEGERLDDEDIASFATLLLMA

GHITTSVLLGNTLMSLDRDPGLFAQVRADRSLVPAVIEETLRLRPPFTRIERVTTTEVTL

GGVDIPENGLVYLWLLSANRDEDVFEEADSFRLHRANGKQAAFGHGVHYCIGAPLARLEG

RIALDALLDRYARIEVDPEVRLSWHGTNVFGARRLPLRLERS

>CYP107L51(2640833517)SALS

VPFAVDADLLALGEDFLRDPFPVYAALRARGPVHRIRMPEGAEAWLVVGHEEGRAALADP

RLSKEWASAGAHLPVKAIASGRSLLSSDPPDHTRLRALLTREFTPRRVAALAPRVQEMTD

ALLDTMLAAPDRRADLVDALSFPLPLGVICELLGVPGLDREPFRRWSNAAVSGLTAEERE

PATASMRAYLTQLIADKRASGGDDLLSALIRTSDEDGDRLSGHELLGMAWILLVAGHETT

VNLISNGVLALLTHPEQLAALRADFDGLLDRTVEEVLRYEGPVETPTYRFTTEPFDIGGT

TLPGGGELVLIALADANRDPRRFEDPARFDIHRDPRGHLAFGHGIHYCLGAPLARLEARI

VLRSLLERCPALTLDTALNRLDWRTGMLIRGPERLPVRW

>CYP107EJ1(2640833810)SALS

MPASKSSCPVAHGPVRDFPFTPVDGLVIEPVLAELRREEPISRIRLPYGGEAWLATRYQD

VRNALADPRLSRAAVVNADVPRRQEMQVGPEGIMYMDPPDHSRLRRLLTKAFTRRRVEML

RPRVAEIAAELAGDLAAQGSGGDLVSTFSWPLPTRVICELLGVPYEDREIFSRGADALMQ

GDTLPHAEFTSRINELCLYLAKLIAQRRAQPTEDMLTALIQARDEDDRLSEQELISISVV

LLAGGHETTANQITNFLYTLLRHPGQLALLREKPELMPQAVDELMRYIPLGTGHHAAMIA

TEDLEIGGQRIAEGESVYVHIHSANRDGEFFENPDELDLGREDNHHMGFGHGVHFCVGAQ

LARMELAVAVESVLDRFPTLELDIEDEAIRWRSSLLVRGPLALPVRW

>CYP105BK3(2640833932)SALS

MSTHRPLANDPLTSAWAAGLRSSLRAFARTHLPEAMVPAHFMVLPDLPHLPNGKVDRAAL

PALSADTSPERAYLAPRTPVEKHLARIWQDLLGLPQVGVETSFFDLGGDSLTVLQMAAQI

RESYDIRLDLRRMFEDPTVARLARMIGSQADPAVTGADNPRGIDAEAMRADAVLPPDITP

HPEALPATQGPFHHILLTGGTGYTGAFLLRELLDRTPATATVHVLVRAQTPRQATDRVRA

NLAEYGLLTDTDMDRVAAVPGDTGRPYLGLTKDTYLRLAADTELVIHNAAVSSWIVPYPQ

LKPVNVFGALEVLRLACRTRIKAVHFVSTIGVYPGHPGERTWHEEALTESAHVVGGYRQS

KWVADTLMLQARERGIPTHVHRLGAVTGSQKSGACSPDTFINHLIKGCIQLGAYLDLDLL

LDLVPVDHCAAAVSHIALSARSEHAVFNLPSARPTAMNDIFELITTYGYRLRRLDYRSWY

RELAAAVERGEDNELARYLPLFGTDQPAEEVGYQGSRPAFTTTHLDAALEGTGIRPHPVD

RELFDLYLDHFVATGFLPSPDEAAARHLRPTTVETPTMTTTPAPAPAPVFPLPRTHPLDP

PPAYRELAAEQPVFQVRTPRGEHVWLVTRHEDARTVLTDLRFSSDPKTPGYPSYISGDTP

IPPGFFLNQDAPDHTRLRRLVTREFLITQMEAKRPRMKAILDDILAAMAARGTTADLVRD

LAFPMAATVMCELLDVPYEDHHIFVTLTDTILDRSSTPAQAEGAARELMAYFDKVVTARE

QQPTDDMLGRLVAQEEAGKLSHDEFVGLAALLMLSGYDTMAQMIGLGTATLLEHPEQLAA

LRADPALYPQAVEELLRYLSINHAGLPRAATEDLTLAGTRISKGDGLLVMINAANRDATV

FTEPDTFDIHRPDPQSHVAFGHGFHKCIGLTLARVELSTVFAGLFEKFPTLQPAAPLEEL

PFRHDMVLYGVRALPVTW

>CYP199R1(2640834023)SALS

MYPDREQNMTTTTEPTGVPIVDADPFDPETLLDPYPLHERLREAGPVAFLPKYQHYAYAR

YEQVQAALADWETYSSAAGVGLQDPRDPGYFRTPGLLLEHDPPDHTKFRKVFAPVLAAPA

VRKLRTESAATAAQLVDSVVRKGSVDIVRDLAQVFPLQVLPDAVGLPVPGREHLISFGEL

VFNGFGPQNDLFTRALERSGPARDWVAACMRRENLSDTGMGATIYASADAGVITHDEAGT

LVRNFLSAGIDTTVSAISGALYLLATNPDQWARLRADPSLARAAFEEAVRLVSPVQQFVR

SSTREVEVDGAVIPPGSRFLIFHSAANRDPRQWAEPDRFDIGRRPAGHVGFGTGIHACLG

QVIARLEGELILSALVERVERIELTGQSRWHLNNSVRSLASLPVHLHPAAQEPGEHHAG

>CYP154A20(2640834238)SALS

VPEQQPLVLDPTGSNHHAEDDALRARGPATLVDILGVTVWSVSDPTLLTYLLSSAEVSKD

PRHWPAGGQVAPTWPLALWITVDNMSTADEINHWRLRRPLAPTFGAHRVQAMAPAVENIV

TTLLDALDAVPPGTTADLRAHLAAPLPLAVISQLMGIGERQRSAFALVVDGLVDTTLTPT

QTKAATARLYELLDELIAAKRREPGHDMTSQLLATRDNDGGRALVGDEVRDTLLLMISAG

YAPTVNAIDQAITALLTDPTQLAVLQRGDADWGDVVEETLRHEPPIRHLPLRYVLADIPL

PDGQTIRTGEAILASYSAAGRHPDWHGDSADRFDATRKAKDHLAFGHGVHFCLGAPLARL

EVATALRMLFDRFPHLELAVPPIELQPLASLISNGHRALPVRLRPTHT

>CYP1194A1(2640834359)SALS

MTTTVRPPEGGACASGHGAPLPFAGKKGLLGHAKELQQDTIGALHRLSETAENGVLGFRI

GSSPAVVVSSAATAREVLIDRADDFGRGKRQTRALTPLMGRGLLTSEGELHSRQRRLVLP

HFSPRRVPKHADAIVAVAEETVRRWSEGVDVDLVAEMNALTMDIVTKLLFSTSTRDNQTI

ASAITEAFEWEMHAITSVFALPMWAPTPRNRRARAAIETFRNWIAGFIRERRTADELPED

ILSDLMGARYEDGGTMTEDLLLDEVLTAWGAAQETSADAQAWTLYLLARHPEAMARVHEE

VDSVLGGRSVTFEDLARLPYCLQVFKEAMRLFPPAAVIPRQALKDTVIGGYTVPAGTMVF

LNTFSLHRRPEVFPDPERFDPDRFTREREKAQPKGGYLPFGTGGNICPGSHLAMMEGHLL

TALLNQRMRYELLPQGAEVRPELLVNLRPDPGVRAHVSPR

>CYP108N11(2640834423)SALS

MSIIEAPGAVDLPDEIARRVVLPEGHSEDEPLFEAYRWLRENNPLGLAKVEGYDPVWLVS

KHADIMEIERQPQIFTSGGGEQPGSHNPILQNQAGDEFTKGLTGGSLRVLDTVTYLDPPE

HTAVRGIAADWFRPANLKQWEERIRTLAREAVEKRLHSGTNHLDFVQDFALFYPLHVIMS

LFGVPEEDEPRMMALTQEFFGTADPDAQRADIEPLGPEAAAQQWSATIQDFYAYFDVLVE

ERRKSPQDDLATIIAGARREDGEYYPKTFAYGWFVAIATAGHDTTSSTMAGTIEQLALHP

ELLAKVKEEPKLVPDLINEGLRWSSPVKQFTRQATEDYTLRGRRIGKGDRFMLLYQSANR

DAEVFDEPDTFRVDRRPNKQIAFGYGPHMCIGQHLAKLELRIMLEELLPRIQSIEVAGER

KVVQTNFVGGLRKLPLRLTLS

>CYP108N4(2640834443)SALS

MSVIETESNPVLPDDVARQIVLPEGHRDEVRLFEAYRWLREHNPLGQARVEGYDDLWLVS

KHADIMEVERQPHIFTSGGGDEPGTHNPILGNQAGDEFTKQLTGGSLRILEALPYLDPPE

HTEVKDIAAEWFRPANLKKWEDSIRSLAREAIERFLKSGQNEIDFVKQFSVFYPLHVIMT

LFGVPEEDEPRMMALTQDFFGTADPETQREDIEALSQEAAAQQWAAAIQDFFAYFDSLVE

ARRAEPKDDLATIIACAKTADGEFYPKISAYGWFIAIATAGHDTTSSTLAGTIEQLAHHP

DQLEKVRQDPAQIPNLINEGLRWSSPVKQFTRRATQDYTLRGRQIKKGDRLVLLYQSANR

DEDIFDAPDDFRFDRRPNKQIAFGYGPHMCIGQHLAKQELRIMLEELLPRIAHLDTTGER

KVLQTNFVGGLQNLPVRLELR

>CYP105B40(2640834592)SALS

MAVPAPQTLTDVPAPRRSGCPFDPAPAYEQARREEPVSRIRLWDGSWCWLVTRHQDVRTV

LRDQRFSADSSRPGFPFLSPGRRVLGTGRTSFIRQDDPEHARLRRMLTADFMVRRTEELR

PRIQELADALLDTMTAHGRREADLVPEFALPLPSVVICLLLGVPYADHDFFQHLSSVMLR

TASSPAEISAAQDELQGYLADLAGRRRGQPDEGILSRLAAEGELDDEEIASVGRLLLIAG

HETTANMTALSVLALLRHPDQLARLRAGGPQERKAAVEELLRYLTIVQDGVVRLATEDVS

LGGTGIAAGEGVLCMLSSANRDEAAFPGEAGLDLGRDARRHLAFGFGVHQCLGQALARVE

LQVALGTLLSRLPELRLAVPFEEIRFREDMLVYGVHALPVTW

>CYP157C24(2640835027)SALS

VTTPSPYRPGTDDTALAPPPGCPAHGRGPGGAHRLYGPEAEDLAAVYERMRAEHGAVAPA

LLHDDLPIWAVLGHGENLQMVRNSSHFTRDSRHWRAVQDGSAGPDHPLAPVFTWQPMCSF

VEGAEHRRLRGAVTGAMATIDHRGVRRHINRFSQRLVNEFCEDGRADLVSRFAEHLPMMV

MCQVIGMPEEYDERMVQAARDMIQGTETAIASNAYVMAALTRLVRRRRERPVEDFTSALI

THESRLSDEEVAQHLRLVLIASYEATANLIANVLRMVLTDPRFRAQLNGGQMTVPEAVEQ

SLWDEPPFSAMVGYFATQDTELGGKHIRAGDGLILGIAPGNVDPAVRPDPEAPMQGNRAH

LAFSGGPHECPGQDLGRAIADTGVDALFMRLPDVELAVPESELRWRSSVLSRHLVELPVQ

FAPRPPQEPAGQAVTVPAPRGNWEVSSLTANSPATAATSAPAPASAPAPASGPAPASAPA

SEPAAPAPATAPGAARHAQNERRGGLGRRLLRWWRGQGR

>CYP1193A1(2640836132)SALS

VTTDTTTAATAESLLDETTFVTDGYAEVFARHRRDNPVARDEKYGRWMLFGYDDVRQAYL

DQRLSSKDGTIIGGSLNGQSDSASNRMLICSDNPSHQRLRKIHSPLFFRSMLKATDTATR

QRLAEASQEFAAGGGGDLLSAVLQELPVSFLSLAYAIEPADAARLVRLSERLIGYADPRI

GDAERPAARRAEAHLELLTAIMKLNARGAAAPGCPVAAPPQRADALAEMKEQLQQDEYLY

NFLNVTVGGNDTTPYTAASFTEYVLGRDTEELAESWARDRTGFLAEVFRWTSTNAYVQRR

ALCDLEIGGQEIKQGEFVVLWNYSANFDEAYFGADFAPEVPHPRPHLSFGVGAHRCIGAQ

VAAVEIECYLDWFFSRAGAMTLPGPFERLHSTFMRGYTSAEVGMAG

>CYP107KW1(2640836267)SALS

MAETTKAEQPAAHEPKYTYPIPRTSPNEPPCQYAQLRAEEPVCPIALPTGHPAWLVTRYE

DVKTVLADPRFSREALFRDGAPRAQLVEPDSSSLISMDPPRHSVLRNLTNREFSPRRAEA

MRPRIQEIVDRLLDDMAEMTGPVDLNATLARPLALQVICELLGIPYEDQHLFGAWCDHFM

SLTKYTAEEIVRSNTEMRNYIADLAAQKREKPGEDLLSKLVHSMDNEQTITHEELISTGV

MFLLAGHDTTVTVTGGGAVLLLRNPEQLALIQQDMSLMDQACEEIIRMVTPGDGTFIRIT

LEDVELSGTTIPANSAVIAPISAANRDDSVFEDPDTFDVHRKDNKHIGFAYGTHFCAGSA

LARAEIQIAMKSLFTRFPTLRLHVDAEDLRWRMTAALGGYEEIPVIW

>CYP154L2(2640836688)SALS

VHAHPEAHWIYNWCGVENMFTAYGPDHTRLRKLVAPAFTARRTQEMQPAVDRITGELLDA

LGTRAPGEVVDLREQFAHPLPMRVICELFGLDEAERTDVAALINTFVDTSLPPEVAGQVL

ANGRETLARLVARKREHLGEDLTSALIEARDGGDRLNETELVDTLILVLGAGHETTVNLI

GNAVVALLDNPDQLALVLDGTVPWSQVVEETLRWAPSLAHLPLRFATEDIPVADVVIGAG

EPILATFGAVGWDPEFHGPRAHLFDVTRAPSRHLAFGHGVHRCIGAPLARAEALTALPRF

FEAFPEVALAKDQARAPFPSFIAHGHQAPKAVLRP

>CYP156D2(2640836689)SALS

MSFPPPPYYRLHTEDFAQRRHEYYADMRRHYGDLAPIELVDGVFGYLVVSHRAAADVLHD

TETWTKDSTDWAAQWPEDHPILGMLGPRPNPMFTDGADHARHRGVTADAFAMIEPHHLRD

MVRDVADQLIDTFAADGEADLVARYARPLPSYVFNRLFGRDDFFAPRLVDGLAKMMEGGR

EADAGKKAFEAYLMELIAEKRENPGRNLTTWMLQHPAGLDPVEIVHQVVLTFGAGQEPTT

NLIASPLARLLRDDELYNQVVSGTLDVIPAIDRVLADQPPISNYGAHYPRQDLTFHGRGI

PAHTLVMIGYEAIGTDPRGPGCAASGRGSHLAWSMGPHACPVPGTAMSIAETALTQLIHR

LPDLELAVPETELRLRKGPFHHALASLPARFSPVRVATRGEIPWPSNPFTPGASTPSTST

APTSPAKPHGYESWGTWLPSSCPAGYGPGHPPATASSNPS

>CYP156B14(2640836698)SALS

MDSQAGASPQTAPPAGCPMHQPQSAPLYGEDFAAHPQEIYARLRAQGPVAAVELAPGVDA

DLVIAHDAALRVLQNPSLFARDARRWAALNEGRVPADSPVLGMMGYRPNAMFSDGAEHLR

LRKAITESLARINTARLSRDVERVADYLIDQFSERGSADLLNDYAKLLPLLLFNQLFGCT

GDIGDRLTHYMSMLFDGQDVVRSNEELNACLMELVALKRRQPGEDMTSWLIQHPAGLRDE

ELKDQLVLLMGAGVEPERNLIGNALLLLLSEEAPAPGSRGGGLLVEDALDDVLWNHSPIA

NYGTHFPLQDVDLDGTVLPANSPVLISFAAANSDPSLTDARQTLSKGAHLAWGAGPHACP

AKSPAQLIALTAIERILNALPDIALAAPARELAWRPGPFHRALVSLPVRFTPTAARRAAA

PRPVAQAVPVPPTQALPGEQTPKKAKGWWSSFLDVFRV

>CYP1035A10(2640836699)SALS

VTSPAGDIAVDRRAVVPLLGRLRAPEGLAEPFPVWNEIRSLGEAVPTPWGGWLLTGFDVC

NQVLRGRDWLVPDFAWQARQPDTERWQAPATQEMSRTLSRLNPPMHTHQRRGIGNLFDRA

ALTALGPMVAGHTTHLLDELAERFARGPADFVRPVGEMLPIATIGDWLGIPPEDHRYLVE

LTHDQAHAQELLPNKSQLALSEEATRQLRAYFTGLIAERRKAPGADVLSSWIRTWDELEP

DRESADEILYTLTMFVTVAALETTTTLLSTVVWRLLQDPARWDWLRRNPEHIDSALDEAL

RYDPPIQLNTRIAAVDTELAGIPLAKDTMVHVMYGAANHDPRANENPGDFDILRGGNHLT

FGGGIHYCLGAQLARLEARTLLGQLLERFPTLHTPGAPHYASRMVFRRIISLELAV

>CYP105D27(2640837030)SALS

MTEPVAFPQDRTCPYHPPAAYDPLRGGPPLKRVTFFDGRSVWAVTGHAAARELLGDPRLS

SDSQNPAWPVPSARYVGLLNRRTPLLQVDDPEHRRQRRLLIPSFTLKRVQSLRPGIEETA

DGLLDALLSRGNQAELVGEFALPMPSKVICRLLGVPFADHDFFEEQARRLLRGPELSDVE

DARDRLEGYLRQIIEEKQVRPGEGLLDEIIREQADVEGEFDPQELAELAFILLIGGHETT

ANMISLSVFTLLQHPERLAELREDRALLPTAVEELMRHLSIVEGMLRVATEDIEIAGETI

RKDEGVVFSLSLVNRDESVYSHPDDLDWQRPARHHIGFGYGIHQCLGQNLARAELEIALD

RLLTRLPGLRLAVPAEEIPHKPGDTIQGIVQLPVTW

>CYP102B23(2640837069)SALS

MAQTTEPAGAAGRVSGFRSAELGWPRLERIPHPPRRLPLLGDVLGVHPRTPVQDSVAMAA

RLGPIFRRRAFGKEFVFVWGAGLTADLADESRFAKHVGVGVANLRPVAGDGLFTAYNHEP

NWQLAHDVLAPGFSREAMGGYHPLMLDVARQLLAHWDEGERAGTAVDVPGDMTKLTLETI

ARTGFGHDFGSFERDRPHPFVTAMVGTLAFAQRRNTVPVALAPLLLRGAERRNAADTALL

NSTVDEVVAARARDGGGQGDLLDRMLEVAHPATGERLSAENIRRQVITFLVAGHETTSGA

LSFALHYLAQHPAVLARAQEEVDRVWGPTGEPGYEQVAKLRYVRRVLDESLRLWPTAPGF

AREAREDTVLGGVHPMRRGAWALVLAGALHRDPEAWGEHAEEFNPDRFAPQAVRSRPAHV

FKPFGTGARACIGRQFALHEATLVLGLLLRRYDLLPEPGYRLKVAERLTLMPQGLRLRLN

RRSAGAGA

>CYP107BX2(2541755515)SALB

MSAATPAPAGAVPPLAPLHRRAPAEPGPPRPCTLPDGSPGWLVDRYADVRQVLSDSRFGR

AGLYLQDGPSRSQAAGLVDDPELMFNQDGVEHLRLRRTLRRAFTPRAVARWRPWIASIVD

QLLDDLSARSGPVDAVAEFTLPLPVAVISRLMGLDASVRGRMRHWSEHAFSDGTRPKDEV

DAALAEFTAFGARLLAQRRRAPGDDLVSSLVRAADAEGGIPEDRLVSLVCGLVAGGHDST

MTMLGNSLLYLLAERPEEWPRLADEPSAELAAARLIHLIPLGDDPGSTRCATEDAEVGGV

LIPAGAVVLADSTTANRDPSVFPAAQTEALFTPLPAPTLAFGAGPHYCLGTWLARLELHL

ALHRLAVRFPGLRLAEPEKPVRWRPAGTSRSPERLPVTW

>CYP107L2(2541755874)SALB

MTDVVDLAAYGERFTADPYPVYAELRERGPVHRVRLPRADGPVEAWLVVGYEEAREALAD

PRLSKDPTTLGITIPEGELIGRHMLVADPPEHTRLRRLVAREFTARRVQALAPLVQRITD

ELLDAMVPLGRADLVQSFAFPLPLTVISELLGVPVADRAAFRRMSGEVVAPSGKVPPEAN

LAELGTFLDALIEEKRRSGTTGDLVGDLIRTADDEGDQLSSSELRAMAYLLLIAGHETTV

NLIASGVHTLLRHPDQLAALRADPTLTEGAVEEILRYEGPIETATWRHTAEEVEFGGVRM

GKNEPVLVSLASAGHDPARFPDPERFDIRRRTQGHLAFGHGLHFCLGAPLARLEGRIALT

SLLARCPELAADGEPSGWAPGLLIRGVQDLPLRW

>CYP1420A1(2541755917)SALB

MLPTEQRLRRRTQLSHGLVWLAAAQGDPYAALLRGLDTDPRPYWRLLSEERLSRSATGAW

VTARHAVARQVAAEPALRALPPVEGVDAPASGTGASEAAHHVEEGWERALASAAREPDLV

TLARDGALGTLSRAWGLDSAGERTLRVAVELTGAALDDPFYPQSLAATRGIAEGVAALRS

LPEMGRRGEELLAAAGVPMVTSLVVNTLTPHPSADRRPDLPGLWDQLRAEPGRAASAVRE

TLRWAGPVQLHATVADAACDLAGRRIEEGEQVVVALGAANRDPGVFTHPDRYDPDRPADE

LSAVLPPGLEWSPVVAFAVAVAEEGLRRLTAGGGGAAPAGPVVRRAAAPVSWAPAVRLSE

TI

>CYP154T1(2541756125)SALB

MTTTPDAAVPEITLDPYGSDHHGEAARLRALGPVVRVRLPGDVRAWAVTRHDLLAELVAD

PRMSKDWRNWNAIRRGEIEDGWPLIGMVKVTNMVTADGQEHRRLRKLVMQTFTPRRVAEL

RPRVEAIVAGLLDELPSHADADGVVDLRRHYANPVPMRVICELFGVPGHEQPRLKELMDN

IFRSDLGPEEVTASQVEQYQLLARVVAARRAEPGPDLTSALIAAREADPDALSEEELVGT

LLLMLSAGQETTLSLVTNAVRALLTHPDQRALAEAGGEEVWEAVVEETLRWDAPIGNFPF

RYPLEDVEIAGVPIGRGEAIMAPYSAVGRDRAQHGEDADRFDLTREQNRHLAFSHGPHFC

LGAPLARLEAALAIPAVFARYPGLSLAVDPATLTPVPSMFSNSSATLPVRLAHDT

>CYP157J1(2541756126)SALB

MTAAPPPGCPAHASTGLVPLTAATGAPDHREVYRRLRAEWGDVARVELEPGVPAWLVMGY

REMLTLTRNEQLFSRDARNWRDLREGVVSPDSGLLPMMGWRANVIGADGPEHRRLRAPLD

DGVARIDQRRARRQVEALCEELIAEFAARGSADLVGEYATIVPMLALASLFGLDSAGGHE

LLRALIALFGSADDSQAGNRQFEEILLDTLRERRARPADDLTTAFLDHPDLHNEAEHLQS

VVVMISAGNETVTAWIAHTLRLLLCDPRFASRLHGGRLGIDDALDEALWRDPPMNNMPAR

YALRDTELGGHRIRQGDCLILGIAAANDDPLIRPEDELTEPGNRAHLAFSAGPHVCPAQV

PARLITRTAVKTVLHRLPGLRLTVPAEEIGWRPSPWTRVPTALPVAFPPAPRPSKENR

>CYP146A3(2541756303)SALB

MTIDLANPDLYTTDARFEMWEEYIRSDAKVWSDPGISPSGFWSVFSHRDVSAVLSPKAPF

TSEYGMMIGFDAEHADHSGGRMLVVSEGNWHSVLKRLIGPFLSRLRAPELRSVLHEEVRE

IVERLRSQETTDIALTVGPRLPAAIVCEVIGVPLSEREHLIQLTNHAFGGEESSFDKMTP

AEAHTEILFYFHELIERRQKEPGTDLVSALLADGRLSTEDVLINCDNVLIGGNETTRHSI

TGAFHAFQAFPGALDTLRADPELADRAVEEVVRWTSPAAHVLRVATEDCEIGGQQIREGQ

AVVAWLAAANRDERLFAHPHRFVVDRAPNRHLGFGTGPHHCLGAALARLELKELLTQLAA

EAKAVEPREEVRWMRSNLVQGYSGLEVSMRWR

>CYP170B5(2541756894)SALB

MSAESTTERPAAPGPRTPPLVAGSVPVLGHAPSLVRDPLAFLTGLRDHGDLVRVKLGPKT

AYAVCAPELVGALLRNSQEFQVGGPLWENLEVLLGKGVATSNGADHRRQRRMMQPAFRPE

RIASYATVMEEEARAMADRWRDGQVVDIGAETFSCAVRVVARSLLEVDSIGAKADRISAS

LHTVFSGLYRRMILSFGPLHRLPTPANRRFERALADLHHLVDEVIAERHAQGKGADDLLD

ILLTSEDGEGRPLGDQEIHDHLVSLIVAGAENVASTLAWCFQLLTEHPAEEQRVAEEALS

VAPDRPVNFGDLGSLDHTRNVIVESMRIRPAAWIFTRRAVADTELGGYRIPAGADIIYSA

YAMQHDPRSFDRPDVFDPDRWIPERAEKVPQYAMMPFSTGNRKCPGDHFSMVEATLMLAT

VLPRWRLVPVPETDPAPRIGITLQPKRAVFRVESR

>CYP107U11(2541757323)SALB

MSETQAAGRPELFGWEFAADPYPAYAWLRTHEPVHRTRLPSGVEAWLVTRYADARQTLAD

PRLSKNPVHHAADEAGRSRTGIPGERSAGLMTHLLNIDPPDHTRLRRLVSKAFTPRRVAQ

FAPRVQELTDGLIDGFAGRGEADLIHEFAFPLPIYAICDLLGVPREDQDDFRDWAGMMIR

HGGGPRGGVARSVKKMRNYLAELIHRKRADLGDDLISGLIRASDHGEHLTEEEAAAMCFV

LLFAGFETTVNLIGNGTYALLRNPAERARLQRALAEGDEALLDTGIEELLRYDGPVEMAT

WRYATEPLRIGGADIAAGDPVLVVLAAADRDPERFDGPDRLDLARTDNQHLGYGHGIHYC

IGAPLARLEGKAALATLLTRLPDLRLGTEPEELRWRGGLIMRGLRTLPVEFTPERDEAAK

KV

>CYP157A13(2541757603)SALB

MTPPDIPSATADAPQAATPAAPATGGCPVAHGAGGAEPAPLLLGGDRFQSDPIGLYRDLR

RDHGPVAPVVLPGKLPAWLVIGYRELHQVTSDPVLYSRDSDLWNQWDRVPENWPLLPMIG

KQESILYTVGERHRRRAAVMEGGLEAVEAHELRATTERLADSLIDDFCGSGDADVIADYA

MMLPALVLFQLFGLPESEGRGLAHAINDMINGGERALAGQQHIRECVGRMVADLHVHPRD

HVTSRMLRLGGHLPGEDRAVITDEFSAAEIIQDVLVMVVAGHQPTADWIGNTLRLMLTDT

RFAASLFGGRHSIAEAMNEVLWEDTPSQNIAGRWATRDTHIGGRRIREGDLLILSFAGAN

YDPLVRTDQSALTGGNNAFFSFGHGEHRCPFPAREIAEIVARTAIEVILDRLPDIDLAVA

AEELTRRPSPWLRGLTRLPVRFTPVPAG

>CYP154C12(2541757604)SALB

MSLSETSATQTPATPPPGCPAHAHGAVDPEAYIALDPLVRDLDGESARLRAAGPLAKVML

IGDVPVYSVTHHAEARKLLTDSRLVKDINHWNAWNNGEIPADWPLMGLANPGRSMLTADG

PEHRRLRNFVAQALTVRRVQKLRPGIEALCARMLDTMEQAADENGVVDLKAHYAHPVPMT

VITELFGMPSHHIPRLKELFDIFFSTVVPREQVPPMMAELDDIFQAFVQSKRDEPGDDLT

SGLLEAAADGDTLTNEEIVNTLKIIVTAGHETTISLIVNAVRALSAHPEQRARVLAGEIP

WSQVIEETLRYNTPTSHLLIRFPTEDIEVGDQILPKGEGLIVSFSAIGRDENQHGPTAGA

FDATRDPIRHIAFGHGPHVCPGASLSRVEAEVALPALYARFPELTLAVDDSELRNKPILT

QNDLFDLPVRLHG

>CYP154A1(2541757634)SALB

MPSSAPPEQEPLVLDPTGADPHAEHRALHARGPATRVDVLGVPAWSVSDPALLRALLTSP

DVSKDGRAHWPAFAETVQSWPLALWVAVRNMFTSYGADHRRLRRIIAPAFSARRVEAVRP

VVERITAGLLDDLAALPPDAPVDLREKLAYPLPVAVIGHLMGVPEERYAHFRAVVDGVFD

TTLGREEAAANTDALYVVLDELIATRRAEPGDDLTPLLLATPDEEGAEPALSHEELRDTL

LLVISAGYETTVHVIDQAVHALLTVDGQLDLVRSGQVGWGDVVEETLRHESAVKHLPLRY

AVRDIPLPDGRTIARGEAILASYAAANRHPGWHDDADVFDAGRLSKEHLAFGHGVHFCVG

APLARLEVEVCLRLLFARFPGLALDLPAEGLPPFPSLISNGHRNLPVWLHGGALGEGPG

>CYP156B11(2541757655)SALB

MESHPPYASATGRCPMHDANFAADPQALYEQLREQGPAGPVELAPGVDATLVVGYETALR

VLQNPTSFPRDARRWAALNEGRVPMDSPVLPMMAYRPNALFTDGAVHLRLRKAVTDSLAK

LNITRIRRDVEPIADYLIDQFSERGRADLLAEYAKLLPLLLFNKLFGCPADIGDTLTTEM

SAMFDGKDPVRTKARLDACLMQLIAIKRREPADDVISWLIQHPAGLTDEELKDQLVMLMG

AGIEPERNLIGNALLELLSGGAGGRGAGMMVEEAVDHVLWHRTPIANYAAHYPAQDTDFG

NGVIAPAGSPVLISFAGANSDPALAEARRSAGRGAHLAWGAGSHACPAKDPAQVIAVTAV

EKILNALPDLTLGVPEEDLQWRPGPFHRALVSLPVAFSPTPATRMAAALQNRGIPQQQAA

PATPQPQHTAPASSDGGGRRKGFWSSFLDIFRV

>CYP105AK8(2541758533)SALB

MPIATPIRTVRTARSSIAGWLTRRYLSRLRRKGTTLDLNALSKLPEPALLPLRRNGLDPV

PEIGALRDREPVSRLPVPAVPVWLVTGYDEAKEVLGDARAFSNDFAHLVGTNGVAEHHEP

GGLGFADPPDHTRLRRLLTPEFTMRRLNRLTPLIHSIIEERLDALEAAADADGRVDLVEH

FALPVPALVICELLGVPYEERDAFQQFSVARFDVLGGLGASFGAISQSREYLRGVIAQQR

REPGDGLLGMIIREHGDAVTDEELTGLADGVLTGGLETTASMLALGTLVMLQDRTHFTAI

REAEDPGAVATPFVDELLRHLTVVQTAFPRFARENTVVGGQAISAGDIVIVSLSAADRDK

RLGPEMDAFDPSRPPASSHLAFGYGIHRCVGAELGRMELRAAFPLLVERFPALRLAVEPQ

ELEFRKLSIVYGVDSLPVRLK

>CYP1047A4(2541760513)SALB

MSAGTGAREQGRQGEVPVVVPGPKGVPLLGSLPEFGKDPLAFFERLRGHGDVVSWNFGGK

PSLFIGDPDLVGELLREVESTFDQPDLGVAFRAVLGNGVTVARGRDWRRKRSLVQPSVRP

KQVKSYAATMASCAVDTAGGWRDGQRIDIKREMAALTQRIAVRTIFGTDAEGDVEAIGRA

MDIAQREIGAEFSGIGAVLPDWVPTPGRRRVKKAAAVIDREVGRVVAAHREDGERPDLLS

RLLTAQDETGNRLSEEEIRDETVTLYIGGHETTSSTLVWAWYLLARNPRVRAALDEELDR

VLGDREPGFDDFARLPYAQAVVQETLRLYPILWLLTGIAKEGASLGGLPVAPGTRVWTSQ

WAVHRDPRWYGDAEVFRPERWLEGAEESIPEYAWFPFGGGPRVCIGARFATVEAVLILAV

LGRRYDLDVDPGEIRPMTTLTLQPDRDMLATVRARGGGA

>CYP157C24(2541760518)SALB

MTTPKAPHSFPGTPAGPPPGCPAHGADPVGPFGAGGLRRLYGPEAERDAPGLFEKLRAEH

GPVAPALLHDDVPIWVVLGHSENLHMLRTHSVYTRNSRRWRLVQDGTLGPDYPLTPLFAW

QPICSFAEGAERERLRGAVNNAMQQIDYRGVRRAINRHSNRLVNEFGQDGRADLVSQFTD

HLPMLVMLDVLGLPEEYNEQMVDAARDMLQGTETANASNAAIMGILERHVARRRAQPDDD

FTSSLLEDEARLTDDEVAQHLRLVLIAAYEATSNLTANVLRMVLTDPRFRAQLNGGQMTV

PEAVEQTLWDQPPFSNMLGYFAVQDAELGGQQIRKGDALLLNIAAANVDPVVRPDLEANM

QGNRAHLAFGGGVYECPGDDLGRAIADTGVDALLMRLPDVELAVPETDLHWTNSLISSHL

KELPVVFSPRQPLELSSSPGQTGASRTDWEISSPAPRPPAAGPVPPQAAPHVPAQPAVDG

SVATVPAQRRGAWRRLVEWVRGG

>CYP107AM6(2541761021)SALB

MARSADTPPLDYPLTSKAALEPPEEWAGLREGCPVARVTLPSGDEATLLTRYDDVRAALS

DPRLSREGLASPDAARVAAGDTAGIFASPMARALNDEGHERWRRMVGRWFTARRMSALRP

GMEELTARLIGRMREHGGPADLVAHLAFPLPVLVICSMLGVPESDRDAFKGWSDTFLNTT

RYTKAETEAAHRDFAAYMSGLVDAKRAAPGDDLLSHLLAGADSEGEPMSEAGLVATGQAL

LLAGHETTAGFIAMMTAHLLSDRTRWERLVADPSLVRRSVEELLRFDPNGSGFGMLRYVH

EDTEFSGGTVPRGTTVVCSMAAANRDERAWQDAAAMDLDRSPNPHLAFGVGPHSCLGQPL

ARTELQAVLTVLLRELPTLRLAVDPRELRRHEGLLTSPLRELPVTW

>CYP107F4(2541761124)SALB

MSTEAVPAAPDSAEVPTCPFDFAQGLDFDPALLAMLRSEERVARVRMPYGEGDAWLVTRY

EDVRTVTTDRRFSRNAVTGKDFPRMTPEPIVQTGAINLMDPPESSRLRRLVRQSFAPRHL

ERMRGRTQSVVDGLLDAMAESGSPTDLFAHLALPLPLVTICEVLDIPEADRHWLRAHAMT

MMNMKPAGKEAAVRAKGELREYFARLTAERRRSPGADMISTLATAREGGEMLGEDELTVM

AMVLLITGQDTTTYQLGNISYLLLTRDDVREQLRREPGSLPRVLEELLRHIPFRKGVGIP

RIATEDVELGGALIRAGDTVHVSYLTANRDGEKFERPDEIDLDRPSVPHMTFGWGSHHCL

GSPLAVMELEIALSTLLRRFPDLALAVEPGEVEWNATSIWRYPLALPVTW

>CYP105H3(2541761180)SALB

MTTSPGPTVVDFPRRTPREPLPLSQYAEHRKQNGLVQTHLPNGRPIWLVTRHEDVRAVLT

HPRISANPDNEGFPNVGETMGVPKQEQIPGWFVGLDSPEHDRFRKVLIPEFTVRRVRELR

PAIERTVDERIDAMLAGGNTADLVNDFALPVPSLVISALLGVPSADRDFFESRTRTLVAI

RTSTDEERAEATRQLLRYINRLIVIKKKWRGEDLISRLLSTGKLSDEELSGVLLLLLIAG

HETTANNIGLGVVTLLSHREWIGDDRLVEELLRLHSVADMVALRVAVDDVEIAGQTIRKG

EGIVPLLASANHDTEAFGCPHAFNPERTERRHVAFGYGVHQCLGQNLVRVEMEIAYRKLF

ERIPELRLAVPEDQLAYKYDGILFGLHELPVRW

>CYP130A10(2641167467)SALL

MMWVLQLTCKSARDAGRSGRRRAPPSAPASPLTLVPHSRTLPAMAAQEAPQAPVSGPAAF

TPCAGEGWRAPWDMYAALRDRDPLHHVDDGDYWVLSRYADVLAAARDTERFSSAGGLTFT

YGERERLGITDAAPMVMLDPPEHTDFRRLITRGYTPRRVAAIEPDVRAFVRDRLDRIAGL

GAGCDIVAELFKPLPSFVVGRYLGVPEADRGRFDGWTHAIVEANALGDPLAAVEAVGGLF

GYFTELVARRRAEPADDTVSDLVRLLPDDDTALLRILGFAFTMVAGGNDTTTGLLGGAAE

LLTADPDRRRALLDSPARLPAAVEELLRLTSPVQCLARTVTADTTLHGRTVPAGRKVLLL

YGAANRDPRAFGPDADRLDLTRDGPQHLAFTHGPHHCLGAAAARLVARVALAELLARFPD

FAVDAAGGTFADGHYVRRYATLPFVTGRG

>CYP107U17(2641167531)SALL

VTCCVAWGGCGTGYGREVQQTSDARQENRSDSHPEARPEARPAGDPPTGCPAAASATAAG

PSPALFSWEFAADPYPAYAWLREHAPVHRTRLPSGVEAWLVTRYPDARQALADARLSKNP

VHHSEAAHGKGKTGIPGERGANLMTHLLNIDPPDHTRLRRLVSKAFTPRRVAAFAPRIQE

LTDRLIDAMIEKPHGGQRGSADLIHEFAFPLPIYAICDLLGVPPEDQDDFRDWAGMMIRH

GGGPRGGVARSVKKMRAYLAELIHRKRADLGDDLISGLIRASDHGEHLTENEAAAMAFIL

LFAGFETTVNLIGNGAYTLLRHPAQRELLQKSLAAGDTELLGTAVEELLRYDGPVELATW

RYATRALTLGGQRIAEGDPVLVVLAAADRDPARFDEPDVLDLTRRDNPHLGYGHGIHYCL

GAPLARLEGQTALATLLTRLPDFRLAVEPDDLRWRGGLIMRGLRTLPVEFTPEPSRPEAI

P

>CYP154A21(2641168034)SALL

VTGHALLKELLTDDRISKNPRDHWPEWQRPEIRGSWLQSWIGVTNMFTAYGADHRRLRKL

IAPAFTARRTDAMVPRVTQIVGDLLDGLAARPAGEVVDLRESFNHPLPMQVICELFGYPE

GAPRGELARVVSEIMDTTATPEQATATQAAVAELLGSLVATKRAQPADDLTSLLVAARDD

EGQGMTERELLDTLLLVIGAGHETTVDLLGNAVFALLTHPEQLKLVRDGAVSWHDVIEET

LRWTPSIASLPLRFAVQDVALPHGEVIRKGEALLPMYAAAGRDPEQHGPAAATFDVQRAS

QDHLAFGHGVHHCIGAPLARLEARTALPALFERFPDMQLAVPAEELQPAGGFIAGGLASL

PVRLTA

>CYP1240B1(2641168035)SALL

VTSPYEPGAAPPPSACPGQAVPPSEAAAPPPGCPAHAGPQRAAAHPEAHAPVKLYGPDFA

ADPHRIYARLRQYGAVAPVEIAPEVPALLVTDYRAALELLNDDATWSKDSRAWMQTVPAD

SPVMPMLHWRPNVFYSDGPAHVRYRDAIVDSFKLVEPHELRARVHHAADTLIRRFGDRGE

ADLIADYARLIPLLMFNTLFGLPDSYSDRLIAAIAGMMEGNSPEEATAANEAYTQYIMEL

VGAKKAQRGPDLTSWMMDHANDLSDEELIHNIILVMGAGNEPLANLIGNALARMLSDDRY

YNTVSGGALTVHDAINEVLWNDPPMANYSAHFPVRDVFFHGTWVRAGQLVMVSYAAANSQ

FDSTGAHGPESGSGSHLAWAAGPHACPVKRHALLIAITAIERLTAWLSDIELAVAPAELT

WRNGAFHRALAALPARFTPITPDQAGATPWQNSDRSPSSSTRPAPTSTAKETASAH

>CYP125A33(2641168217)SALL

MHCPALPEGFDFTDPDVYQSRVPLPEFARLRQTAPVWWNAQPHGIAGFDDDGYWVVTRHQ

DVKEVSTKPEVFSANLNTSIIRFNQGISRDQIEVQKLIMLNMDPPEHTRVRQIVQRGFTP

RAVRALEAALRRRAEQIVEEARHKGSGDFVTDVACELPLQAIAELIGIPQEDRARIFDWS

NKMIAYDDPELAITEEVGANAAMELISYAMNLAAARKECPAQDIVSRLVAAEHDGNLGSD

EFGFFVLLLAVAGNETTRNAITHGMHAFLTHPDQWELYKRERPATAAEEIVRWATPVVSF

QRTATQDTELGGARIKKGQRVGIFYSSANHDPEVFDRPEVFDITRDPNPHLGFGGGGPHF

CLGKSLAVLEINLIFNALADAVPGISLAGDPRRLRSAWLNGVKELQVHYR

>CYP184A8(2641169037)SALL

MSLTNSPTPADPEAERASRQPLPTAIPGPAGLPVIGSLLDLRRDSLGAFLKAQREHGDVV

RLEAGPPGLRSVFYAVFAPEGVQQVLGTQAANFRKDHPLYEEVRQSFGNGLLTSQDDDYL

RQRRLVQPLFTKRRVDGYATAVTTEADAVTARWRSVEGDVVDLVAEMNRLALRTVARILF

GLDVEAAVETIHRCAPVINDYVVRRAYTPLKVPRDWPTPRNLTHRKVTTELNALCDRIIA

ERRTASATGTTVTPDHNDLLSLLVAAGNEEDGTLDATEVREQVLIFLLAGHETTATSMAF

ALHLLARHPAEQTRIRAELTRVLGDRTPTAADLDRLPRLTQAFKEAMRLYPAAAVVGRRA

VEATEVAGHRLPAGADVVVAPWVTHRHPGLWEDPERFDPDRFAPEREAERHRYAWFPFGG

GPRACIGQHFSMLESVLALATLLRSHELTAVDQDVPVAAGITLQATGPARVRLRAL

>CYP107E14(2641169767)SALL

VTTAKTAPLSYPFNIAESLDLSAEYEKARNRPGLLKVQMTYGEPAWLVTRYAEARFVLGD

QRFSRAEGIRHDEPRQSEGSRNSGILSMDPPDHTRLRTLVAKAFTVRQVEKLRPQVKELT

RELLDELEAAGPPADLVDRYALPIPVAVICRLLGVPTEDRPKFRTWSDAALSTSSLTAEE

FDANREELRAYMGNLIEQHRREPQDDLMTALIDARDVNDRLTELELVDLCVGILVAGHET

TATQIPNFVLALLDHPDQLAVLREQPDLIGGAVEELLRFVPLGSGAGQPRYATEDIDVGG

TLVRAGEPVLVAMGAANRDALRFDGPGKLDIRRTGNQHLGFGHGVHHCLGAPLARLELQE

ALSALITRFPGLHVAGDVEWKTEMLVRGPRVLPVGW

>CYP105AC9(2641169777)SALL

MPHDSSTPPVPSPAPSPAPSTPGAPAPAGTPRPEPEPLAALPVTRPAGCPFSPPAAFAEL

RAEQPLRRMRYPDGHLGWLATGHSVVRAVAADPRFSSRYELAHLPFPGMADVTLPPAPVG

DLTGIDPPHHTRYRRLLMGKFTVRRMRELTSRVEQITAEHLDAMERQGPPVDLVAAFAHP

VPALMICELLGVPYADRDRFQHHAMAASGTGGSLEDQYAAMAALQEFVREQVQVKRARPT

DDLLSDLTTSDLSDEELAGIGSFLLGAGLDTTSNMIGLGTFALLSHPEQADALRADPGLA

DQAVEELMRYLTITHTGIRVALEDVALDGQLIRAGESVTLAVQAANRDPARFPDPDRLDL

RRRATGHLAFGHGIHQCLGQQLARVEMRVAFPALLTRFPSLRLAIPPAEVPLRTDLTMHG

VQRLPVAWDA

>CYP161A7(2641169819)SALL

MSSPHRDLPSLDLETPALLRVSPLLRDLQERGPVCRVRTPAGDEGWLVTRHSVLKQLLND

ERIGHSHPDPANAAQYVRNPFLDLMIADTDAETARRTHTESRRLLAPMFSARRVREMEPR

VAAVVDAVLDDFTAQEPPGDLHGGVSVPVARTVLCDIIGVPPQNREHLTALLSQTAVLGD

REGVQRTQRDLYAFVGGLVEHKRGEPGQDIITRLTEGGLSDERVTHLAVGLLFAGLDSVV

TIMDHGVVLLATHPEQRAAALADPDVMTHAVEEVLRAAKAGGSILPRYATEDLTVGGETI

RAGDLVLFDFSLPNFDERAFDEPERFDVTRSPNQHLTFAHGMWHCIGAPLARIELNTVFT

QLFTRLPDLRLALAAGELAENEGRLSGGLSELPVTW

>CYP105H9(2641169833)SALL

MTHSDPVVVDFPTRKPGVPFPPPDYDGYRDHEGLVLSRLPNGARAWLVTRHEDVRAVLTD

SRISSNPSHKGFPNVGTVGVPTQEQIPGWFVGLDSPEHDRFRKALIPEFTVRRIRGLRPA

IERTVEERLDAMLAAGNSADLVADYALPVPSLVISTLLGVPPSDRDFFESRTRTLVSLRA

STDAQRETAVKELLRYIKRLVGIKAKWPGDDLISRLLAAGSIAPHELSGVLMLLLIAGHE

TTANNIALGVVTLLRNPQWIGDDRAVEETLRFHSVADIVSLRVAVEDVEIGGQLIKAGDG

IVPLIAAANHDTSAFECPHMFDPSRSARHHVAFGYGIHQCLGQNLVRVEMEIAYRKLFER

IPDIRLAVPDEGLSIKYDGVLYGLEQLPVRW

>CYP1064A3(2641169899)SALL

VTSGTGRQTGGAARALPGPEPRADGGAGAIAAAGGLHAYQLRLHDAYGPIVRFQLPGTEL

AVSISDPVLLEATAGLDERPVRPFAFLEPLCEAGNLQVLPAAEHGPWRHLLLSVLAGRPS

HERHFGQFTALTTALADRWAEQADRQPVALQRDLTALSLRMICAYAFGGEVTDPEGVVTA

FEEVLTEHLGRLYEVPGAVGPAERAERAERAAAALARLRATVDRVVAAHRRAGRTDRSDL

IGALVAAGERPARIRDTVMMIMLAAHHTTGVAVSWTLYLLGRHPEVAARVTEEVDRVLGD

RAAPEYADLRRLTYLGMTLKEAMRRYPPGPYGARETAEDLAVGDYLIPAGATVFYPFWAV

HMNPKYWPEPEKFVPERFTPEAVAGRPRLAHVPFGFGPRSCEGAALAVVEAELVLAVLLK

RFRFRPVAGDEVTPVERFVLWAADDIRMLVSPRTPG

>CYP147F17(2641169914)SALL

MTSVALLRQVLDYANRPDPYPLYAELRRTPVVRDEAGPYLVSTYWAVKGLLHDPRISSDA

RNLTPEAAEATGQEQDPNLPPSFLRLDPPEHDRLRRLAMRPFGPPHTPRRVFEMHGELAG

IVTGLIDGLRGRDAIDLVDDVSYPFPVTVICRILGVPREDETHFHEWADTIAAGLDPVGT

PEERAAKTQDVQQARRDLAMYLAGLIEERRRSPRDDMLSALATEHGPDGQMSPVEMITTS

VLLLIAGHETTVNLITNGMLTLLRHPDVLQRLRTEPALAGPLVEELLRFEPPVQMLPQRT

TLAEIEVAGTVIPKGAAVYLMVASGNRDPQRFTDPDRFVPDRPDNQHLGFGHGLHSCFGA

PLARLEAQLALTELVRRLDAPQLVEDPPPYRQNAVLRGPRHLPLTIGGIRD

>CYP107EC1(2641169918)SALL

VSDLASFDVTNPLFKANPHDFYAGLRESGPAHMVHLPGYGDVWLVTGYADARAALADPGL

SKAPANVPASLRNAMVEAGERDGFQLMAHMLNSDPPDHTRLRKLVVRTFTARRVRALRPR

IQEITDALLDEVVAKGSADLMDDLAFPLPITVICELLGVPAEDRDDFRRWTSILVSEDEA

LRPQLQDTFAQLNAYLTALVRHKHAAPDDGLLSALVSVAEDGDSLDDDEVVWMAFLLLVA

GHETTENLIGNGMLALLSNPEQLAALRADASLLPTAVEEMLRYEGPVETTTWRFTTRPVE

IGGVTIPADQTVVVVLASANRDPSRFADPGRFDISREDNQHVAFGHGIHYCLGAPLARLE

GQIAIGTVLRRLPDVRLAVDEGDLQWRLGIVMRGLHNLPVRFTPSGPEH

>CYP154A17(2641169926)SALL

MPQQSPHVLDPAGRARRTEDASLRARGPMARVDVLGEEAWAVTDPELLRSLLLDDRVSKD

PHRHWDRFPDRTGDWPLNLWVAVENMFTAYGAEHRRLRRLIAPAFAARTITALEPDIERF

TRELLDDLATARPGEAVDLRERFAAPLPIRVITHLMGLPAHLLADFRRAVNGVFATDVTA

EAAAANARDLYAALDALLALKQERPGDDLTTRLLQARDPEGQGHGLTPQEVRDTLLLVIS

AGYETTVNLIDQALVALLSHLEHLAAARSGALPWGDVVEETLRWQAPVPLLPMRFATTDI

PVADGIVIRKGQAILAAYSAANRHPALHGPDADRFDPTRPDKTHLSFGHGVHLCLGATLA

RLEATTALRMLTARFPELRLAVPADALVPLPSFLTNGHVNLPALVGPEAGA

>CYP105AA13(2641169989)SALL

MRQTVPVPHGLPMDRDANPFDPPRALTRLRAARPVSPLVFPDGHEGWLVTGYEAVRQLMA

DTRFSSRLDLGVVHVPYETPGMPVPTEPSPQIPGVFIAMDPPDHTRLRRRLTGAFTVKRM

KQLEEHIVEVTERQLDAMARLAPPVDLVREFALPVPSLVICELLGVPYEDRDTFQSNSAK

FLVKEQTLEEKMAAYGALTTYLAELVTSKRAAPGEDILSDLARHDDLTVEELTGIAFLLL

LAGHETTANMLALGTFALLEHPDQLAELRADPDLLPDAVEELLRYLAVGDVFYRYATEDI

ELGGETIGKGSTVVVSLLAANHDPRRFEHPDTLDVHRKARGHLSFGHGVHQCLGQQLARV

EMRAGFAGLLRRFPTLELAIPANEVKLRTDMNIYGVHELPVTWTETAG

>CYP163B9(2641169999)SALL

VTVTTPALDVVDLGDPATFADHDLDAFWRTLRDTYPVYWNPPLDGRRGFWVLSRYDDIMA

AYRDDVHFTSERGNVLVTLLGGGDAGAGRMLAVTDGHRHHELRKILQRVLSPRVLSEVAA

AVRVNTRQLIREAVEAGGCDFAEQIASRIPMTTISNLLGVPEQDRDHLLAQTKAALSTDA

EDVDEVDSEMARNEILMYFMDMVEERRESPGDDVISMLVASSIDGVPLSDEDIVLNCYSL

IIGGDETSRLTMIDGVHTLAAQPEQWRRLKHGEVAIDTAVDEVLRWASPTMHFGRSVVGE

TELHGVQLRPGEIVTLWHASGNRDERIFDRPGTFDLGRTPNKHLAFGYGPHFCIGSYLAK

VEIAELLMALRDFTTGFETTGEALRIRSNFLTGFSSLPVRWRPDHSGMKEVD

>CYP107EB1(2641170070)SALL

MDPALIADPYGGFDRLREEAPLVLGRSADSTPTWYATRYDDVRAVLADPRFVVDPELTPG

TEAVDNRNRMLDMLELPKEFHPYLSESILDVDGDRHTRLRGLATRAFTARRVNALRPRVE

AITASLLDGFGESVELISEFAYPLPIAVICELVGIPEEDRGLWRTAGSALTSIAPGSKGA

AAHELIAYTHALVDQRRAAPADDLISDLVKVQDEDGDRLSDVELVTMLLGLATAGHETTA

NLVGNGALALLSHPDQLEALRRTPELWPTAVDELVRSCGSILITQLRYATEDIDVGGQTI

KAGEAVQPVVVSANRDPREFSRPECLDVTRRSVKPGDGHVGFGLGAHYCLGAALARQECE

VALRGLFDRFPRVALTTDEHTWVPVPGLRQLASLPLTLY

>CYP107CJ2(2641170189)SALL

MTIQEPPLPAAEQKPRPQPLPDPVPLTGCPYKSNPYPLYERMREAGPVHRVLFPSGVQAW

LVTGYDAAHAALNDDRLGKNHDRGNERWRARASIMPEPQHSQLQVHLLHQDPPRHTRMRR

FVTDAFTPRRIESLRPRFQELADALIDALPESGPADLVAGFAAHFPFQVLAEVIGLPHHL

AARFDRDWGKVVQPVGPTDPGRPLYEARLHGLQSYIAEVVAHKREHWDDDLLSRLVVARD

RRELSQEELDSMIFQLLVAGQEPVTNQITTALIALFRHPDQLARLRDEPDLLPRAVEELL

RYDSAFELTTWRFLDQDDNLHGTDIPAGDSVIVSLCAANRDPRRFPDPDSLDLDRTPNPH

LAFGHGIHFCPGAALARAELQVALDALLTRLPGLHLAIRDEDIEWIPAVLGRGTNHLPVG

YDRRR

>CYP147F15(2641170247)SALL

MTLGTLPAQITDYANRADPYPLYAELRRTPVRRESDGTYLVSTYYEVRSLANDPRLSNDT

SHRSPGYARIGQPDEETGLPPSFIFTDPPLHDRLRDTINRPFGPPHSPRFLDDLRGDLAK

VVTELLDAFEGKDEVDIVEDFSYPLPVTAICKVLGVPREDEPRFHGWADALASSLDPQAG

EDGLEKAQRARQELGSYLTDLIETKRRHPGPGMLSALAPEMTPADLEATAVLLLVAGHET

TVNAITNTTLTLLRHPDVLARFQREPALAVPLIEEVLRYEPPVQFVPWTTALADIDVADT

TIPEGSPVWLMLAAANRDPKRFPDPDRFDPDRKDNEHLGFYTGIHYCFGAPLARMELHVA

VPELFRRVTFSRLLEDPPPYRANAVLRGPRHLPVAIEGLTA

>CYP1191A1(2641170310)SALL

MTGPDPHTTAPRPCAEAALPPLGSPDPWPGYAALLACPGLHHEQAEHTYFAARHSDVHHG

LRHPDLAVGFPFRATRQLFGPTAIDLDAPRHRPARQQVSWFTTRHMPTWNRTAVIPVIDD

LIERAATDTPLDVIKTFAEPLPTRVICRILGLPDHEWPWVWKQLRPVIGHIADPRTGMQA

ALASRDILADRLRHAVRTGVPHGSLLQRLCANAPSDSAVDRTAEPIRTALLLLAAGTETT

AAAVGNLLWCLQRRPHTWDEVAAGTIPAEAVVTESLRLHPPLHSTVRFARRDLTLGDTAI

PKGARVQLLLAAANRDPARIGASPSWDPHRPPQAHHAFGGGPHACVGAQLALTEMKFLLT

ALTQRFELAGPTHPAGRFRAGPFHYPTELPVRLIPRTTPA

>CYP107L41(2641170328)SALL

MPENPQAPQDGRTPRASEDTTNPPAPRGSDIYTSPEARASAPAPAPGAPPLVDLRAYGQD

FVTNPYPYYAKLRAQGPVHPVLTPYDQAAWLVVGHEAVRTALADPRLGKDWSSANLPRTD

GDGVPLFTNMLDVDPPHHTRLRKLVAKEFTSRRVEALRPRVQRITDELLDAMLTASDDRA

DLIEALAFPLPMTVICELLGVPSMDRDAFRGWSHELVSPTSPEAAQQAVEAMSGYLTTLI

ASLRREAGDGLLADLIRTSDEDGDRLSPEEVIGTAFLLLVAGHETTVNLIANGVRALLEH

PAQLAALRADDSLLDNAVEEMLRYDGPVETATWRCAAEPVELGGTVIPAGSAVLISLASA

SRDPERFADADDFDLTRDPRGHAAFGHGIHFCLGAPLARLEGRIALRSLLDRCPGLALDD

AAEPATWRPGLLMRGTDRLPVRWDR

>CYP163D-fragment(2641170384)SALL

VPDGTPSGGSGEGRDATTGTFRSVLLVVHASDGPARPGTASDAWTGVGVEPTDANTAYLL

YTSGTTGRPKGVCVTRGALAAHVDDMAERLELVPEDRVLWFAAPHVDVAWEQALTPLRVG

ATVVTRGPGVPTFGELADLVERHAVTVANLPGGYWNGWALALTEQQRTQRRALRLMISGS

ERMSARAAVNWQRILPDVPLLNAYGPTEAVITSTLFRVPAGLAARDEIPIGTACGSRQLQ

VLNAELAPVVRGQVGELYVGGGPLAREYLGRPSMTAARFVPDPYADSRGAVMYRTGDLVR

ENADGDLEFVGRIDDQVKVRGFRVEPAEVRLALERHPAVRHCAVLGRTAPGGPTSLVADP

AFWAGDDSLPILRELRQRAPLWHLESATEGPLWCVLSHELAGEVLGDAARFSSERGSLLG

TGRDRAPAGAGKMMALTDPPRATGTCGTWCCRIRARLPRRAAAPPRGARLTRPCPAGGCP

PAHPGSPLPGLRCGGELRSCLASVTIGKGVSWAKACFRRGSVW

>CYP147B7(2641170412)SALL

MSTETLLERINAYTSRPDPYPLYTELREHGVARQDDGSYLVGTYHEIAALLHDPRISSDV

RHRTHPDLRDRPEDLPPSFIAVDDPEHDRLRRLAMRHFGPPHSPGRIDALHDDIVDAARE

LVDGLRGRDRIDLVDDFAYPLPVTVICRLLGVPREDMPEIRGWTNTIIASLDRSPDEDPD

GRLRAAAQARVSMAGYLGALAQRRRERPADDMISALVHDDGPEGRLTEAELTTTLTLLTI

AGHETTVNLITNGMLTLLRRPEALERLRREPELMPSAVEELLRYEPPVQMLPQRTPLTDV

EVGGVTIPQGVPLFLVLASGNRDPLRFEDADHFDPARRDNQHFGFGSGVHNCFGAPLARL

ETQVALTALLHGLDAPRLVEDPPPYRHSPILRGPRHLLVTGDVAAGAGA

>CYP107EA1(2641170487)SALL

LTVRRSAVTGWQPGAAGLKGETQAAARGLPSAIIAVGPFPAPGQDHADRSHADGSADPST

TAPQTPEPVTMNRPATELPPRSAAPGRPDPASAADVHRPFDAGFFRDPYPVYARLRTLGP

VLKVVLPDGSHAWLVTREEHVRAAFTDPRLSVNKARSRNGYQGFSLPPALDANLLNIDPD

DHLRLRRLVSRGFTPRHVERLRDRVGTAAAHYADRLAERLAEHGTADLLAEFANPLPLVV

IGHLLDVPEADGRAFSRWVAAMHAPARPGDTAEAIEHIHRYLLELIRARRAAPGDDLLSS

LIAARDADDRLSEDELVSLAFLLLMAGTENVQHLISGGVLTLLRHPEHLAALRSRPELMP

DAVEELLRHAHPNQMAIRRFPTTSVEIAGVRIPAGDTVLLGLASAHRDPDRYPEPELFDI

HRADKSHLALGHGLHYCLGASLARMEIGVALGTLLDRFPGLRLAVSDGELEWRQSFRSHA

LRRLPVAVSAPAG

>CYP1192A1(2641170512)SALL

MRLRERPAGYDRARHGVHLLRTPEVIADPSVYIDAIAELGPLFFDEVGGMWVCSGYAEAV

EILRDHRTFSSVREHDQDAFQELGLHASASLSTMVHEQMLFMDPPQHKAIRSALAEQFTG

TRVRSRENDLRHIAARALEELPRAGVLDLVADFAAKLPSALVAQLLGMPGREAELTRWAE

AYERLLGSLSALPAAPDREVDAVLTDALSVLQNEARSRLHAPGDDVISSLTAPLVDRSPT

GEELFAVAANCIVLVGGGYQTLTHLVTSALLALHDDPSLEKQLRELPELIPPAVAEFMRI

NGSSQYVARKATTDVKIQGTLITGGESVLVHLAAANLDPRTFSAPRALDLTRHGPKHLGF

GSGRHTCPGAGYAERLAGFAIEGFLAKYPSYAPESEPQAFSWGLHGNTRCLEHARVRVDA

EVIPAATVDIPAAETHDSTGGNGLSLPPAATTAAACWHEVFERQALLTPDAPAVQAPDDL

ISYRELDHWANALAHRLRHQGAQPGALVGIVMERSVEFVLTVLAVAKTGAAFLLADISCP

RERLRTMLVEAEARLVVTDGSLPSSAFPVQIVGVGAKDFRPDAPLTGVSPGDTAYVVFTS

GSTGAPKAIAISHEATVNLHLAQHQIFGLEPRDRVLQFLSPNFDGCIADLTLALLSGAAL

IVAPSNQLTVGPPLVRLLASQRVTTAILTPSVWMTLPDQPLPELRIAAAAGERLPAAWAR

RWAAPGRRLLNLYGPAETAVLATWHECSPSEDPPPIGRPVANKRAYLLDHHLRNVPPGQR

GELWLGGLGVGRYLNQPDLMEERFIRNPHTTTDPASLLYRTGDICRQRPDGTLEYIGRRD

RQVKIRGQRVELDEVERVLESAPGVTACAVHEQDGRIEALAVPAGPQLDETAIRTYLASR

LHSAMLPSVFTTVTELPRTVNGKADHRHEPPDEPTVQSSRPSGPALPPDDHERRLSRITW

EVAQNFAQVLNLPLRQVQADSDFFTAGGDSITMAAFLARLESLAGAPVDTAALITAPTPE

QITTLLLNAGAPK

>CYP1060A2(2641170520)SALL

MGRANEPPRLPGGSFAAWSRDRLALASRGADECGDVWQLEPGVYVAARAGVCEAVLHRAQ

DFPKPSSPLFPPFKRAGGAPMPQERAHARAARMRGLRPQAVAARIGEIAAGTDRIADQWP

TGRDVEILPLVRPVLAEIGVRYLFSEDAPVLLPFAWQLFVAREVLVRPSRWVWPRWVPTP

ARRFRTRRQVAFTNALRPIIRRRRTSQRLGDDVLGQMLQPSSRYGPLAEEAVLDTLPGIT

VATFETPSRAAGWILLHLARYPQAADRVAAEAALLPASPASTTSTHFDNLHYTQALVREV

LRLHPPSWLLTRRAPRRTQLADYTIDAGSTVLVCPYTAHRDAREHPEPDRFRPERWLDDA

GSPTKPGVFLAFGTGPHGCEGAALAMAMLTLMTAQTARRYHLSEPPGAEPGYRITTFEGL

ATAGLCLRATLRG

>CYP147F21(2641170523)SALL

MISGDNLRAFKRDGRIVVVGASLAGLRAAEALRGGGFTGSLTMIGDELGEPYDRPPLSKQ

VLTGWVPADNTTLPRRRDVDAEWLLGVPASGLDLTTNHVLLADGREVPFDRVLISTGVRA

RPWSVESEAALDGVFVVRTREHAEGLRRALAARPSRVLVIGAGFTGSEIASVCRERDISV

TVAELAPAPLVGALGAMIGEVAADMQRAHGVDLRCGVEVTQLEGDARGRFRRAHFDDGSA

VDADVAVVALGSIRNTEWLRDSGLATDGWGITCDTSCRALDVNGRVTDDVFAAGDVARCP

NPLYEHRLISLEHWANAVEQAEIAAHNMVSAQADLRPHLSVPVFWSIQFGVNIKSVGVPT

FADEVVVTQGSLEDRRFVTAYGYRGRVTAAVSFNNGKRLHHYRRLIELAAPFPPPCPTPD

QPADRKPVPVDLPGPALLAQGATAVVTGHDLGERRVTAAPQHRQEQGRTTTTGTPGTLQR

IFDYSARADPYPLYAELRTTPVIRQEDGSYVVSTYREITDVLNDPHLSSDLRNLSRPMPP

ADEGATSSFIRMDSPEHDRLRRMAMRHFGPPHAPGLVTGLEGFLTATVGSLIDNLAGKEQ

IDVVDDFASPFSVTVTCDLLGVPREDEPRFHVWVNDLMNSIDYNPETDPKEKLDKGVRAR

KDVRQCLGELVERSHSRPGDGLLSQLANDDGPDGRMTDAEIVATARLLLIAGHETIVNLI

TNGMLTLLRHPPVFQRLCGEPDLIVPLVEELLRYEPPVHIIPWRVAYSDITVADTLIPKG

SQIMLMLASGNRDPNRFHEPDRFDPDRRDNQHLGFGSGIHLCFGGPLARRETQIALTELV

HRLDHPRLVADPPPYRRSPVLRGPLHLDIEQGCG

>CYP159A15(2641170687)SALL

VTSIPQAPDILSAQFATDPYPAYRVLREHYPLLHHEGTGSYLLSRYDDVERAFREPVFTS

DNYIWQLEPAHGGRTLPQLSGREHAVRRALVAPAFRGRELRETFQPLIERNARELIDTFR

DDDEVDLVAQFATRLPINVIVDMLGLDRADHDRFHDWYTAVVGFIANLAQDPAIADAGRR

AGEELAAYLHPIIQERRAAPGDDLLSRLCTAEVEGVRMTDQDITAFVSLLLSAGGETTDK

AIALVVRNLLAHPEQLAAVRADRSLVPAAFAETLRHTPPVQMIMRQPAEDVTVSGGTVPA

GATVTCLIGAANRDADRYAHPDAFDILRSDLTPDTAFSAAARHIAFGLGRHFCVGALLAK

AEVETSVNQLLDAFPDLAFADGVAPPDAGVFTRGPRQLRLRLRDRER

>CYP140C3(2641170748)SALL

MSDLPYAQRLFARDVRWALGHALPRLATDRAARQGDLHGQLVALSRSPRPHGDTADVERR

LMDRIRAEGPVHRSRFGFVTASHPAVREVLSSNDFRTGALPVTTGPLGRLAAWAGADAPV

GPLKPPSLLVTEPPDHTRYRKLVTRVFSVRAVEQLRTRAEEIAEELLDDLQRRPPGADDV

DLVSAYCGLLPVTVIAEILGVPHAERHRVLRFGTGAAPSLDFGLPRRRFLAVERSLRDFD

AWLAQHIERIRRQPGANLLSQLVTARDDDGRGLTGTELRATAGLVLAAGFETTVNLLGNG

IALLDRHPDQRAALHDDPTLWPNAVDEMLRFDPPVFLTGRAATRDTSIGGRPVPRGALVT

LLLAGANRDAALFTAPHRFDVTRPNAKEHLSFSGGRHYCLGAALARMEAEVGLQALHRRF

PHLTLHAGARRRTTRILRGYVHLPARLGTPVPV

>CYP107AM10(2641170771)SALL

VSHDDGTPAPAYPLTAPGALEAPAEWRELRTTCPVAPVTLPSGDRAALLTRYDDVKQVLS

DPRCTRQLDAEGAARISADPSGGVFNSAMAASLNGAGQQRWRRMLTKWFTAKRMNALRPA

IEAMAEQLVDEMVDRGHPADLKASVGFPLPVWVICDLLGVPAADRDRFSRWSDMLLNLTR

YGRDEIDTAQRDFHAYLTEHLEAKRAEPGEDLLSSLITATDADGGRLTDDQLAATGQALL

IAGHETTANMIGKMMALLLADRRRWQQLVADPALVRTAVEEVLRYDANAGFGMPRYVTQD

IDVAGTVLPRGATLVCSMAAANRDGAVFAAADDLRLERSPNPHLAFGAGPHSCLGQALAR

TELQVVLDVLLRRLPSLELAVPVSELRRIEGLVVGGLCDVPVRW

>CYP105B37(2641170773)SALL

VPDATGPTPTPTGSAEPHHPSDVPEFPMPRAAGCPFDPPPTLTAQQQQGPLTKVRLWDGS

TPWLVTRYADQRALLADPRVSADVTRPGYPSAAPVSGNTIGFILMDDPEHARQRRMVTAP

FAVKRVEALRPRVQQIVDERIEALLGGPRPVDLVEAFALPVPSLVICELLGVPYADHDFF

QENSRILINRNVTPEERTTAHGRLSDYLDDLVGEKLARPTDDLLSELAQRVADGELTRLD

AARMGVLLLIAGHETTANMIALGTLALLEHPGQLAALRASDDPKHVANAVEELLRYLHIT

HSGRRRVATADIELAGRTIRAGDGLIFPNDIANRDPDAFPDPDRLDLQRAARHHVAFGFG

VHQCLGQTLARLELQVVYGTLYRRIPTLRLAVPLADVPFKHDGSVYGVYELPVTW

>CYP105A6(2641170777)SALL

MTDTATTPQTTDAPAFPSNRSCPYQLPDGYAQLRDTPGPLHRVTLYDGRQAWVVTKHEVA

RKLLGDPRLSSNRADTNFPATSPRFEAIRERPQAFIGLDPPEHGTRRRMTISEFTVKRIK

NMRPEVEEIVHGFLDEMLAAGPTADLVSQFALPVPSMVICRLLGVPYADHEFFQDASSRL

VQSTDAQSALTARNDLAGYLDGLITQFQTESGAGLVGALVADQLANGEIDREELISTAML

LLIAGHETTASMTSLSVITLLDHPEQFAALRADRSLVPGAVEELLRYLAIADTAGGRVAT

ADIEVEGQTIRAGEGVIVVNSIANRDGTVYEDPDALDIHRSARHHLAFGFGVHQCLGQNL

ARLELEVILNALMDRVPTLRLAVPVEQLVLRPGTTIQGVNELPVTW

>CYP154D13(2641170917)SALL

MTGQSKRSHQVEDIAPVGPPHRMDPSGGCPHADNARLLARGAVAPVVLPGEIEGMAVLGH

AALKEFLGHPDVAKDARHFTALSEGRIPEGWPLRTFATVRSMTTADGEDHRRLRSLVSRS

FTARRVAELQPRVEELTDSLLDDLAGAARAGGGVADLRRHFALPLPMGVISELLGVDLAH

RDRLHELSVEVVTTDIGSQRAIAANHEFAAVIGEFVAAKARHPGDDLTSALIAARDDDGD

QLSGPELIGTLLLMIVAGHDTTLNLITNAVRALCGHRDQLELALSERVTWGDVVEETLRW

DAPVSYFPFRYPVRDLTLHGTVIPKGTPVLAGYSAAGRDPAAHGPDADRFDVTRPGRPDA

VRHLSLGHGAHYCLGAPLARLEAETALQRLFRRFPDLELAVPEDALPRHAGFVANSVGSL

PVRLWPS

>CYP1190A1(2641170951)SALL

MTTTDAHPPTSRTAAEGPPAFPFDDWGQRISPAYARLREAPAPACRVVTVTGDQVWLVTR

YGLARRLLADPRLSLTAALEADAPRQEPLRPRATGARGDGMATLQERGLRGILADALSPR

AIRAHHAWTRLRARALFDELSEQGPPADLQQGLARPLTFAVARRVLLGELTEDEGQVLNA

WCDTVLVWRDRTRDEIQAALDAMYGFFLRRAPELAAAPGSDVVKRAAAACTRDGGRLGAD

GLAEVANLMLIAGYRTAASFVANALVMMLSHPTALAALRDRPALLPSMVEEVLRHTPMST

GGVKRVATDDVPLDGLTIKAGECVLVSLESGNHDPHAYPEPDRFAPDRFAADRGPVDGTS

SEAGRPRSRPHLGFGHGKHHCPGNALARMQIAVVLQTLADHTPALRLAVPAGELRWRPDV

AFRIPETIPVTW

>CYP107L43(2641171063)SALL

MNAKTTHGAREETVVDLAAYGAQFVENPYPVYAELRAKGPVHRVRVPGQERDFWLVVRNE

EGRRILADERLSKDWRSQGVWPADALPINENMVESDPPKHTRLRALVTRAFTARRIEALA

PRVHTLTADLLDAMSKAPNGRADLVAALAFPLSMTVICELLGVPDLDRQSFRQWTNEIVA

SSSPEATAEAVRAVNAYLTGLIEQKRAEPRDDLLSALLRTTDEEGDRLSPEEVVGMAFLL

LAAGHETTVGLISNTVLALLRHPDQLALLKADFSLIGNAVEETLRYDSPTENSTYRFATE

AMDFCGARFEKGDPVLVSLAAAGRDGERFEDGERFDITRSARGHLSFGHGIHYCLGAPLA

RLEAGVAVRALLERCPDLRLDTSEPLVYIPGMLVRGVRRLPVRWTG

>CYP107EA2(2641171298)SALL

VIDQRGLLKPFRPEFFANPYAAYARLREDSPVCRVELPDGTPAWLVLREADVRSALADTR

LSVDRSCSRNSGYKGFSLPPALDANLLNLDGDTHIRLRRLVMRAFTYRRIGDMRNDVIKA

AERLSDKLDSSSTCDLVTDFATPLPLQVIGDMFDVPEEHRRPFAAWVGTMFALERPQQVR

DSIDNIHQFLLRLVAERRREPGQDLLSALIAARDDHDRLTEDELVSLAFLLLSAGVQNVQ

HLISNGIHTLLQHPEQLAELRSEPSLLTSAVEELMRFAHPNQMSIRRFPTEPVQLGGVTI

PAGDTVMLCVASANRDPARYPDPDTFDIRREDKSHLALGHGVHFCLGASLARMETEATIG

TLLRRFPNLSYAAPVEELQWRSTFRSRSLKALPLRLK

>CYP163B8(2641171305)SALL

VTPSLPDDISSVDLTDPKTFEYYDLRDYWQQLRNTRPLYWHPPTASGPGFWVVSRHADVM

ALYRDNKRLTSERGNVLVTLLAGGDSAGGKMLAVTDGERHRDLRNVMLKAFSPQALRPIV

DQVRVNTTRLVVEAVRRGECDFAADVAERIPMNTISDLLGVPAEDRDALLSLTKSALSSD

EEDHSANDAWLARNEILVYFSDLVAERRAEPTDDIISVLANSTVNGEPLSEELIVLNCYS

LIIGGDETSRLSMIESVRALAQHPEQWQLLRDEKVLLESATEEILRWATPAMHFGRRSVT

DFELHGQVIAAGDIVTLWNSSANRDERVFGDPYVFDLNRSPNKHITFGYGPHFCLGAYLG

RAEIRAILDALRTFSTAFEINGRPQAIHSNFLSGLCSLPVRFYPDDAALDAYLDRNRVTG

>CYP107EL1(2641171329)SALL

MVHAHMPSPARVGRGGDPDPYPNYAWLRKEAPVSALYSPDGDGRSWLVTSYEFARACLED

SRLSNDDREAAGDSERVSEELWSTARGLLNLDGPEHMRLRKVVSGAFSPRTSAQFRPMME

RVCQEAIDSFASRGSADLAAEYALRVPVAIIHEVLGVPESVRKDPARCFDLFYRTGLART

TDPTCFEELVEYADELADYKRSHPGDDIGTLLLRSVDSGQLQGQRELRSMILSVLGAGHV

TTVQSVGCAVLRLLENPDQLASIFAGSPSWAQSVNEMLRYDSPIQATVNRYATEDMRIGE

VDVAKGDVVLISIAAANRDPERFEDPERFDVTRPSRSNLAFGHGTHLCLGAHLARIEGEV

ALEMLFRQLKDLRLAIDPAEVVWSYGPMLRGPRELPVTFSR

>CYP107EL1(2641171370)SALL

MVHAHMPSPARVGRGGDPDPYPNYAWLRKEAPVSALYSPDGDGRSWLVTSYEFARACLED

SRLSNDDREAAGDSERVSEELWSTARGLLNLDGPEHMRLRKVVSGAFSPRTSAQFRPMME

RVCQEAIDSFASRGSADLAAEYALRVPVAIIHEVLGVPESVRKDPARCFDLFYRTGLART

TDPTCFEELVEYADELADYKRSHPGDDIGTLLLRSVDSGQLQGQRELRSMILSVLGAGHV

TTVQSVGCAVLRLLENPDQLASIFAGSPSWAQSVNEMLRYDSPIQATVNRYATEDMRIGE

VDVAKGDVVLISIAAANRDPERFEDPERFDVTRPSRSNLAFGHGTHLCLGAHLARIEGEV

ALEMLFRQLKDLRLAIDPAEVVWSYGPMLRGPRELPVTFSR

>CYP163B8(2641171394)SALL

VTPSLPDDISSVDLTDPKTFEYYDLRDYWQQLRNTRPLYWHPPTASGPGFWVVSRHADVM

ALYRDNKRLTSERGNVLVTLLAGGDSAGGKMLAVTDGERHRDLRNVMLKAFSPQALRPIV

DQVRVNTTRLVVEAVRRGECDFAADVAERIPMNTISDLLGVPAEDRDALLSLTKSALSSD

EEDHSANDAWLARNEILVYFSDLVAERRAEPTDDIISVLANSTVNGEPLSEELIVLNCYS

LIIGGDETSRLSMIESVRALAQHPEQWQLLRDEKVLLESATEEILRWATPAMHFGRRSVT

DFELHGQVIAAGDIVTLWNSSANRDERVFGDPYVFDLNRSPNKHITFGYGPHFCLGAYLG

RAEIRAILDALRTFSTAFEINGRPQAIHSNFLSGLCSLPVRFYPDDAALDAYLDRNRVTG

>CYP107EA2(2641171401)SALL

VIDQRGLLKPFRPEFFANPYAAYARLREDSPVCRVELPDGTPAWLVLREADVRSALADTR

LSVDRSCSRNSGYKGFSLPPALDANLLNLDGDTHIRLRRLVMRAFTYRRIGDMRNDVIKA

AERLSDKLDSSSTCDLVTDFATPLPLQVIGDMFDVPEEHRRPFAAWVGTMFALERPQQVR

DSIDNIHQFLLRLVAERRREPGQDLLSALIAARDDHDRLTEDELVSLAFLLLSAGVQNVQ

HLISNGIHTLLQHPEQLAELRSEPSLLTSAVEELMRFAHPNQMSIRRFPTEPVQLGGVTI

PAGDTVMLCVASANRDPARYPDPDTFDIRREDKSHLALGHGVHFCLGASLARMETEATIG

TLLRRFPNLSYAAPVEELQWRSTFRSRSLKALPLRLK

>CYP105B22(2641171512)SALL

VPHTTIPDFPLDRAPGCPFDPPPHYAALRAQAPLVRVRIWDGQTPWLVTRHEDQRAVLAD

PRFSADPSRPGFPAPTAGFKAQGREEVQALSMQDDPEHARQRRMLIGRFTVKQVTAMTPR

LVQIIDDLLDRMEAAGPPTDLVAAFALPMPSLVISELLGVPQQDHALFQRTAGTLISRES

TVQEFAAARTELADFLGDLIRRKDDDPGDDLLSSLVVTRMRTGELTPALLVETAMTLLVA

GHETTTNQLALGTLVLLRNPDQLAVVRDSDDPARVASAVEELLRYLSITQNGLSRVATED

VEIAGQLVRAGEGVIVPNASGNRDAAAFRDPDRFDVGRPDVRGHLAFGYGTHQCLGQNLA

RKELQLAYPALLRRFPGLRTTLPDEDIRFKHDMIAYGVHELPVTW

>CYP105H1(2641171745)SALL

MSTGAEAPTPAPQCPVAFPLRQPGRPFPPPEYAQYRAGPGPVRTELPSGTVWLVTRHEDV

RAVLTDPRISADPSRPGFPKASRTGGAPSQYEVPGWFVAMDPPEHGRFRKTLIPEFTVRK

VRELRPAIQQIVDERIDAMLAAGTSADLVESFALPVPSLVISSLLGVPRADRDFFEDRTR

VLVRLSSTDEERDKATQALLRYLGRLIQIKQRRPGDDLISRLIAAGTLSRQELSGVSMLL

LIAGHETTANNIGLGVVQLLANPQWIGDDRIVEELLRYYSVADLVAFRVAVEDVEVGGRL

IRAGEGIVPLLAAANHDDTVFAAPREFDPERSARSHVAFGYGVHQCLGQNLVRVEMEIAY

RTLFARMPSLALAVPVEELSLKYDGVLFGLHELPVTWK

>CYP161A6(2641171747)SALL

MSTPTAPPSLAAEARTVLRLSPLLRDLQSRAPVCKVRTPAGDEGWLVTRHSELKQLLHDE

RLARAHTDPANAPRYVRNPLLDLLVTDDVDGARAVHAEMRALLTPQFSARRVLDLAPKVE

ALAEQALAHLTAQGPPADLHDHFSMPFSLSVLCTLIGVPAAEQGQLIAALAKLGEIDDPP

RVQEAQDELFGLLSGLARRKRTEPEDDVISRLSRKVPSDDRIGPIVAGLLFAGLDSVASH

IDLGTVLFTQYPDQLAAALADEQLMRSGVEEILRSAKAGGSVLPRYATTDVPVGDVTIRT

GDLVLLDFTLVNFDRTVFDEPELFDIRRAPNPHLTFGHGMWHCIGAPLARVQLRTAYTLL

FTRLPGLRLARPIEELGYSSGQLSAGLRQLSVTW

>CYP1199A1(2641171923)SALL

MPQRSTASRGGRQVRFAPRIDALLRQHTGQDLFRLEPTTVGVGGADLMDALLRSRPANAE

ERPTFKPVLGRHVSRADAATYMQAVAADVRKALQRPLEAPVDLTGPWPQVPHAYLRDLVF

GRELLRFRVLVDRRLELTPKLTWSAVTSGAALLRRPDSTEPLSKLAALVLGATGFPDRRY

AMYLYRRVAAPICFTVAALVTNAVWLGAPFDDSVPNRHLLAEALRLLPPSWNILRMASPE

FTVLDTRIGPADDVLLLPLLSHRDPALWEEPDAFRPERWADLDPDDHPGYLPFGHANERC

WGRHMVLPLAERLLDLVRRDGLTVRPAQTVGRVELDGLMEVAEVRVMRG

>CYP180A10(2641171979)SALL

VTGSTGGAGREPARSPDVPDVFDPRRYAAGLPHDAYRLLRDHHPVAWQDEPEVLGWPAGP

GFWAVTRHRDVVRVLKNARTFSSRLGATQIRDPDPADLPFIRRMMLNQDPPQHNRLRRLV

SRAFTPRRVDRFEDAVRDRARTLLASAVDAARAADGVCDLVGTVTDDYALRNLADLLGVP

PAERGLLLDWTRRVIGYQDPDEAGPAVTGASGRPVDPRSPAMLRDMFAFARELAAHKRRH

PGDDLMTVLAADRELAVPELEMFFFLLTVAGNDTVRSAAPGGLLALAGHPDAYAALRTGA

VPMGPAVEEMLRWHPPVLSFRRTAAVDTELAGRRIRAGEKVVVFHASANHDERVFAEPGR

LDLGRAPNPHVSFGDGPHVCLGAHLARLQLRVLYEETCALLPAVAVAGPPPRLVSNFING

LKSLPLRVAT

>CYP147F14(2641172007)SALL

MTTTQTPDTLRRILDYSSRADPYPLYAELRETPVARQEDGSFVISTYREISDILHNPHLS

SDTRNLSCPTEGMQAEGTPAFINLDPPEHDRLRRLAMRHFGPPHTPGLVTGMEGALTTAV

GHLIDDFAGKERIDVVDDFAYPFPVAVICHLLGVPREDEPRFHRWVDDLINSIDYNPKTD

PKEKLDKGVQARKDLRQYLGGLLEQRHGHPGDDLLSRLANDDGPDGRMTDEEIVATANLL

LIAGHETTVNLITNGMLTLLRHPHVLQRLCKEPDLVVPLVEELLRYEPPVHIIPWRAAYS

DITVAGTTIPKGAQIMLMLASGSRDPNRFHDPDRFDPDRRDNQHLGFGSGIHLCFGGPLA

RREAQIALGQLVRHLDRPRLVADPPPYRRSPVLRGPIHLYVEQGAA

>CYP251G1(2641172145)SALL

VTGAGVPGDDPRGGSPRGAAGVPGRVPLVGHAVPLRRDPLRFLCGLRQRGPVTKIYIGPR

PVHVVNSSDQVRELLTVQARSFDKGAMFDALRVPLGDGLITAAGDRHLRHRRLVQPAFHH

ERIARYARTMAERSLARSADWAPGTTRDLVPDINRLTLDVLLRTLFAAPHEPGLDAAVQD

WLTVKYHSMRLALSPLHAWAERVPLLPGWRPPDAGPLRRLVDVQLRIIDGYRADGRDRGD

LLSMLLLAGGPEGALTDAEVTDELITLFLAGTGTVSASLAWALHEISRRPDVQRRIHDEL

DTVLAGRPPACEDLPALVYTRQVLTEVLRLHPPSWLLMRRAVRPVTLGGVRLAPGDEVFF

SPYALHRDPHLYEDPEDFAPDRWPADAAAKAPRHTFLPFGAGSRLCIGEDYAWTELTLAV

AAFTAGRRLEPAGTAPVRALVGTVLRPDRLPLTARPRPA

>CYP107AE9(2641172338)SALL

VNDPMESAEFSRDPYPLLAALRARGPVQRVRTGKGRTTWVVTGWAEARAALADRRLSKDT

ARYFANRPSGRDLAPAVSQSMLATDPPDHGRLRKLAMAAFTPAAVGRLEPRIREIAEGLA

AELGRSAGAAGGPVDLVEGFAVPLPIAVICELLAVPEADRAAVRRWSDDLFAAADPGTAD

RASHALAGYVAELIAARRAAPGDDVLSGLIAARDAGDRLSERELVSLAVLLVVAGHETTT

HLIGNGTLALLRDDALHARLRDDPGLLPAAVEEFLRYEAPVTLATFRYATEAFDLGGVRI

GAGDVVLVSPGGANRDPARFDEPDAVRLARSGAGGHLSFGHGPHHCLGAPLARAEARIAF

EVLLARFPGLRLAAGGGQGAGGSDGPDGVTWRRTRLMRGPAQLPVLLGPLREAPEGRG

>CYP1035A12(2641172493)SALL

VEPALAAASVDRRSVVALFSRLRSARGQSNPLPFYTELQSMGEMVPAPWGGHLATSYRLC

HQVLRSRDWRVPDSGWRAAQADAVRWHAPASQQMGATMPMLNPPHHTQMRRPLGNVFDRA

ALQQMERSVERNAEFLVDTFFEQLSGGTADFCALVGDELPVITVGEWLGLPSADFAVLRS

LTHDQVHTQELFPTPSQLAISDSATRNLRKYFTDLIRERRKAPGDDPVSRWLRMWDEFEP

DQDAADEAVYALALFMLLAALETTSHLLTTMMWLLLEHPRQVDWLRTHPEHIPGAIDETL

RYDAPIHMISRIAPEDTELAGVPVREGEMVQLMVGAAHHDPEQYAAPEVFDVRRKAPHLS

FGGGIHYCLGNALARMEATCVLTSLLRRLPVQGLRVAGTPTWAPRVAFRRLMELPVVRT

>CYP156B17(2641172494)SALL

MQSHPEFQTPPPGCPAHAEGIKTSLHGTEFAAAPHAVYEQLRKHGPTAPVELSPGVEAEL

VTDYATALQILQNPDSFARDPRRWRALNEGRVPLDSPVLPMMMYRPNSMFSDGATHLRLR

QVVTDSLAKVDMHRVARHVDRVATYLVDQFSIRGKADLIGDFAQVVPLLVFNDLFGCPAE

LGDRLIVAISSLFDGIDVERANEEMAGALFELVALKRALPDEDMTSWMMQHPAKLSDEEM

VHQLALLIGAGTEPVQNVIGSSLLLLLSEDQFADGQHGGGVLVEDAINEVLWNSPPIANY

ATHYPVRDVEVSGSKLPAGAPVLISFAAANADPTLSATRQTFSKRAHLAWGAGPHACPAK

DPALLISVRAIEKLLNTLPDIELGVPQETLTWRPGPFHRALNALPSRFTPIRSDRRSSAG

YAAQDAAQPGGGAQDQGRRKRDGWWSGFLNWWKV

>CYP107Z13(2641172683)SALL

MTELTDSPFSEFVGKHPGEPNVMEPALLTDPFAGYGALREQGPVVRGRFVDDTPVWFITR

FEEAREVLRDQRFANSPAHSAGGGSADTPIDRLLEIMGLPEHYRAYLSGTILNMDAPDHT

RLRRLVSRAFTARKITDLRPRVADIAEDALRRLPEHAVDGVVDLIPHFAYPLPITVICEL

VGIPEADRPQWREWSTHLVSLRPELHPETFPEMIDHIHALIRERRTALTDDLLSELIRVH

DDDGSRLSDVEMVTLVLTLVLAGHETTAHLITNGVAALLTHPDQLQLLKSEPALLPRAVH

ELMRWCGPVHLTQMRYATEDVELAGVRIKKGEAVTPVLVAANHDPRHFADPDRLDLTRQP

AGRAENHVGFGHGMHYCLGATLARQEAEVAFGKLLAHYPDVALAVAPEDLQRVPLPGSWR

LASLPLRLN

>CYP155A6(2641172701)SALL

VDGEDRDYGRRKIERETPTTGCPVSRAGDGSWQVRGYDEARAVLRSTTTVQAGLGIESVE

GLPSRIRRPVLYRDGPEHREHRRQTARFFTPRHVDQHYRDVMSRATRTQLDRLTANGRAT

LSDLAFNVAIEVACAVIGLTESRPGIKQRLERFFPEEFGTPGLRSLRGIYWTIRQSTNWL

RVHLGDVRPAVRARRRRRRDDLISHLLDEGCSTSEILGECLTFAAAGMVTTREFISAAAW

HLFSDTELLTHYQAVDEAGRIAVLNEILRIDPVIGRLSRRTTGPLDVPRNGDDPLTVPAG

ERIDILLDHTNLDERTVGPRPQRIRPGRTMREGARSPGLSFGDGPHKCPGTHLALLETDI

FLTSLFALPGLRMATPPTIGFIDGIASYELRHCVVEVDTVP

>CYP102G12(2641172789)SALL

MTDTIAPQTGDRASAVGVPVADLTATGISSTPLQQAMDLARIHGPAYVRKFGARETLFLS

SVDLVTEVSDETRFAKGVSVVLENVREFAGDGLFTAYNDEPNWAKAHELLMPAFALGSMR

TYHPAMLKVARRVTASWDRRMAEGRPVAVAEDMTRMTLDTIGLAGFGFDFESFSRDTPHP

FVEAMVRCLEWSMTKFARQPDADHTAADAAFRADADYLASVVDEVIAARAASGERRDDDL

LGLMLAAGEGEAAHQGPALDLANIRNQVITFLIAGHETTSGALSFALYHLLKDPVALRLV

QREADELWGDEADPDPTFEDIGKLAFTRQVLNETLRLWPTAAAFTRQARTDTVLGGRYPV

AAGSLVTVLTPMLHRDPVWGDNPEAFDPFRFTPEAEAARSPHAYKPFGTGERACIGRQFA

LHEATMLLASLVHRYRLVDHADYRLRVKETLTLKPDGFTLALAPRTPADRAAVRSALAVL

PGGPAGAAAGDATDAAADEGLPTRVRQGTGLLLLHGTNYGTCREFAERLADEATALGFAT

EVAPLNAHAGSLPTDRPVVLVAASYNGQPTDDAAAFTAWLGTAPEGAAAGVHYAVLGVGD

RNWAATYQQVPTFLDDRLAALGGERMLPRAEADASGELAGAVRKFGAALRTELLIRHGDP

ASIGDRGADGADTGYAVTALTGGPLDALTARHDLVPMTVTEAYDLTADGWSRPKRFLRLA

LPDGVTYRTADHLAVLPVNTPQAVARTAAALGVDPDSVLALRPPTGRPVRDTLPIDRPLT

VRQLLTHHLELGMRPTSEQRALLAAHNPCPPERHALENLPDDDPRSLVELIEAHPALRGA

LPWPVVLELLPPLRTRHYSLSSSPAADPRHADLMVSLLPGGTGSTYLHAVRPGDTVLARV

QPCREAFRLDPDDDTPVILVAAGTGLAPFRGAVADRVAAGRTTPARLYFGCDDPDGDYLH

AAEFAAAERAGAVAVRPVFSARPENGHRFVQHRIAAEATEVWELLRAGARVYVCGDGSRM

APGVRDAFRAVHRERTGASEQESQAWLRELTAAGRYVEDVYAAG

>CYP105B33(2641172806)SALL

MTEMIDTTVPAAPAPLPVEPPSGCPFDPPAEFGVLRTEEPISKISLPDGSWAWLATRYAD

IRAILGDTRFSSDTTLHGYPLSGMTGGGNQQNRGFIRMDPPEHTRLRRMVTREFMVKRVE

ALRPEIQRLTDELCDAMERRAGKPVDLIEALALPVPSLVISLLLGVPYDDHDVFQRLTGK

LLSRTIAEPEREAARGELREYLDALVTAKEKEPGDDILGRLIVEQQRTGEITHDDVAAFA

ALLLIAGHETTANMIGLSALTLMQDPDSAERLRQDPTLIRGAVEELLRFHSIIRNGPRRV

ATTDIEIDGQLIRAGEGVVVAVPSANRDATVFADPDRLDVGRANAQHHVAFGYGIHQCLG

QALARVELQVVIATLLRRFPAMRPAVPVEEIPFRTDMAIYGCHALPVTW

>CYP107X11(2641172849)SALL

MQNTAETGPAAPLDTTPLLDAPYAAYAALREAGPVHRITGTDGQPAWLVTRYDDVRSALA

DPRLSLDRRHATPGNYRGFSLPPALDANLLNMDPPDHTRIRRLVVKAFTPGRIEALRAPV

QRIADELLDAMAARGRAELVTDYAGPLPITVICDLLGIPVERRRDFLAWSDALITPDPSR

PQAMKEAIGAMLEFYTGLIAAKRAEPGDDLLSDLIAVRDDTADDPAGDRLSEDELTSLAF

LILFAGYENTVQLIGNAVLALLDHPERLAALRRNPAELAPAVEEFLRYDTPASLAIRRFP

VEDLDIGGVRIPAGESVLLSIASANRDPERFPDPDRLDPTRELSGHLALGHGIHYCLGAP

LARMEAEIAIGALISRFPELRLDVARDEVRHRRTIRARGLISLPVAW

>CYP157C28(2641173042)SALL

VTAFPSHQPGTTSASGPPQGCPAHARAGGTDHLARLFGPEVAHDAPGFFERLRREHGPVA

PVLVDGDLPAWLVLGYRENLDVLRTPTRFSHDSRIWHCFREGRVPADSPLMPALAWQPVC

LFMDGTEHERLRLAINESMARFDRRGIRRCVTRSANQLIDAFVADGRADLVRQFAEQLPM

LVLTQLLGMPDEAGPRLVEATRDLLKGSETAVESNAFLMAALEQLVARKRDAPGPDFTSW

LMSHPTRLTDEEVAQHLRIVALAANENTTNLTANTLRMVLTDPRFRASLTGGSMTLPDAL

EQMLWDEPPTSVLPARWATGDTELGGQSVRAGDMLLLGLAPGNGDPVIRPDRSVPMHGNR

SHLAFSSGPHECPGQDIGRAIVDTGIDVLLMRLPDIDLAVPEGDLTWVSHWIARHLRALP

VKFTPGVPASADASGDGADGPHDLRESAGGGPLVAGGVDASTPPAGAPRSRPSWWARLTR

WLSGQ

>CYP113D6(2641173052)SALL

VTNASTQWTFHEDQFWMRGELPPGRVCYDEKKGLWNVYGYAECLQVLGDAETFSSDLSIL

APEGKRQIFPGNLTTMDPPEHTKMRKIVSGVFTRGVVAALEPRIKAITHELLDQVEPGDA

FDLVEVLAHPLPVIVIAELLGIPAGDRHVFREWVSKLLENNQSFSTGEDTEELRKQREET

FVQINNLSTYLREHVEQRIAEPRDDLLTKLVQAEVDGERLSTAEIVNFAFVLLVAGHITT

TMLLGNTILCLDAHPTALKSVRTDRGRIPAAIEESLRLFAPLAALRRVTRKPARIGDVEI

PELQVVMVWTAAASRDLDQFRDPNTFDLDRGNNPHLSFGRGSHFCMGAPLARLEGLLALD

ILFDRFPALRCDPDQAPVFMPGANVMGVESLHLLT

>CYP105D23(2641173216)SALL

VTGTIPFPQDRSCPYHPPTNYRPLRESGPLSHVSFYNGRKVWAVTGHAEARTLLVDPRLS

SDRQNPAFPIPVERFEAVRRVRTPLIGVDDPEHNTQRRMLIPSFSVKRTAALRPQIQQIV

DGLLDRMLEQGPPAELVSAFALPVPSMVICSLLGVPYADHEFFEDRSRRILRGGTAEESE

QARRELEGYLADLMARKETDPGDGLLDELIAERLRAGTLQHQELVRLAMVLLVAGHETTA

NMISLGTFALLEHPDQLAQLRSDESLMPGAVEELLRFLSIADGMLRVATADIEIAGHTIR

TGDGVVFSTSLINRDATAYPSPDELHVDRSARHHVAFGFGIHQCLGQNLARAELEIALRS

LFRRVPDLRLAVPAAEIPFKPGETLQGMIELPLIW

>CYP163C3(2641173722)SALL

MTAMTSRPEPAPDALGDVDLADPLLHARHDLGPLWRRLRTEAPVHWQPESGTRPGFWVIS

RHADVVGVLNDPQTFTSERGNVLDTLLAGGDSAAGKMLAVTDGGPHKALRSALLKPFSPR

ALDVVVDSVRRGTRALVEEAVARGTVDFAADVAAHIPLAAICDLLGVPLADRRQIIELTS

SALSSADGVPTEEATWSSRNGLLFYFSELAAARREKPLDDVISLLVTKEIDGRPLSHEEI

VFNCYSIIMGGHETTRFAMVGGVQALMERPEQWHALKTGQVSTASAVEEVLRWTTPALHS

GRTATQDVFLGGQFVEEGDIVTAWMASANGDERVFDRPDTFDLARTPNKHLSFAHGSHFC

LGAFLARAELAALLESLCDLVAVAEPAGAPGRVFSNFLSGFSSLPVTLVPEGRQAT

>CYP107F9(2641173762)SALL

MAKEADPTVWNCPFDYAEALEFDPTLRRIMTEEPVARIRLPYGEGEAWLVTKYDDVRTVT

TDRRFSRHAIVGRDFPRMTPEPIVQDEAINVMDPPASSRLRSLVAKAFAPKQVERMRSRT

QHVVDELLDRMVENGAPGDLMENLASPLPLTTICEVLDIPEGERAQLRGYARTMMNTSLA

NKDNAIRAKADMREYFTELTARRRRDPGDDLISALATARVGDEVLDAKELTVMAMVLLIT

GQDTTTYQIGNLSYTLLTRPKDLAMLRERPEALPQAMEEMLRFIPFRKGVGIPRVALEDV

ELSGVTIRAGDIVHVSYLTANRDSEKFERPDELDLSREATGHMTFGWGAHHCLGAPLALT

ELQVALSTLLQRFPDLKLAKPAEELRWNTTAIWRYPLALPVVW

>CYP1189A2(2641174113)SALL

MSTTAQLRPFHDIPGPKGHALAGLLPDFNADPLGFLTRGFQEHGDLVAYRFGPRKGPLGK

TILAVYHPDLVHQLLMDTERTFGRDTDGFRATYELVGRGLMTTEGPYWRRRRHILQPLFT

PKRVARYTELMAAEAERIIAEHEQFEGTEIDLHQAMMRYSLRVVGRALFGGDLDDAEAEL

HALIPDANRGIMARTTQIPKLPLKFPSPTNRMFVRTRDSLYDLIQRVIERSGSGDAASSE

DNIVSRLREARDPESDEPLTEQEVRNEALLLFMAGHETTAMGLTFGLHQIGRHGDVQKAI

AAEIDAHHAAGGTGAEYAQNRDTLGRAALNEGLRLFPSVHMTERVANEDLELNGYHVPKG

TSVFLVPWVTHRHPEFWPDPERFDPDRFVGKQADRPRYAYFPFGGGPRVCIGEHFALLAS

SILMEALLRKYQITSHDEQISMKVLNSIRPDRDVRTTFTRR

>CYP1189A1(2641174114)SALL

MTTTEQIAAFADVPGPQGSGVAGVAPEFLRDPIAVLARAHRDHGDLVAFPFGPRKGPLGK

VVVAAYHPDAARQVLTETERTIGRGPSSTQVLDDMIGRNLMTTDGAEWRRQRRTLQPLFT

PKRVAQYTDLMAAEAARIVAEDVPAGTTADRVDLHRLMLRYSLRVVGRALFSGDIDYTAP

ELHKLIPLTNELIIGRTTQLLKPPLALPTPRNRAFLRTKAQLYALIDRILARSDAEAGGA

ERDDIVTRLRTARDPETGAGLSDAEIRDQTLLMMMAGHETTATALTFALHLLGRHAEVQQ

AVADEALEYTRGGGTPAEFAQQRESLARASLLESMRLYPPVYMTEHLATADIVLGGYRVP

AGTAVFLSPWVTHRHPEFWPDPERFDPHRFVGEHDRPRYAYLPFGGGPHVCIGEHFALLA

ATVLLEAVVRKFRIESLEESISYQQTGNLRPDKPVWAALTAR

>CYP107B6(2641174128)SALL

MSNSSLPHADAFTAESLRDPHALYAKMRDEAPVQKVVLPQGLAVWLLTRYDDVRAALSDP

RLRSDKSDVDGVLRNHLVSQEARESWVDELSGNLLNTDPPDHTRLRRLVNRAFTPRTVAA

MRSRIEEVTDELLDALPRGSEVDLLASFALPLPIIVICDLLGVPPEDRGVFTDWSNALLS

SADAAETAEAGQKMFAFLGALLAEKRARPADDLLSGLVQVRDEEDRLSEEELISMALLLL

VAGNESTVNLIGNSVLALLRHPDQLAALRADPALLPGAIEEFLRYDGPINTATFRSTAEP

VTFSGVTIPAGELVVVSLLAANRDAGRFADPDRLDVTRPAGGHLGFGHGVHFCLGAPLAR

MEGEIALGRLLARFPDIKPALALDELTYRFSTIIHGLEKLPVIV

>CYP105AK8(2641174156)SALL

MTTLRSRIFAWAGRLYLARTRKKGFDLSRMSFLPDSVLMPLRRDGLDPVPDLADVREREP

ISKLPVPIASNVWLVTGYDEVKAVLGKADAFSSDFTNLIGKAGAGAEQNPGGLGFADPPV

HTRLRRLLTPEFTMRRLGRLTPRIHDIVEERLDAMERAGRNGDPVDLVAHFALPIPSLVI

CELLGVPYEDRADFERLSAARFDLFSGANASFGAISESLSYFRDVVKKQRENPGDGLLGM

IVREHGDSVSDEELAGLADGVLTGGFETTASMLALGALVLLQDPQHFAALKDGDDVVDRY

VEELLRYLTVVQVAFPRFAREELEIGGVQIAAGDVVLCSLSGADRDGELGPEMEQFDPHR

AKVPSHLAFGYGIHRCVGAELARMELRAAYPALVRRFPTMRLATRPEDLAFRKLSIVYGL

DSLPVRLDA

>CYP159A12(2641174773)SALL

VNREAPDILSPEFAADPYSAYRVLRDDFPLLHHEATQSYVISRHEDVERAFKDPVFTTDN

YDWQLEPVHGRTILQMSGREHAVRRALVAPAFRGNTLEQSFLPVIERNARELIDAFRGTG

SADLVADFATRFPVNVIADMLGLDRADHDRFHRWYTAIIGFLGNLAQDPDVTEAGLRTRD

EFAAYLLPIIRERRTAPGDDLLSTLCAAEIDGTRMSDEDIKAFCSLLLTAGGETTDKAIS

SLVHNLLRHPEQLAAVRADHALIPRAFAETLRHTPPVHMIMRQAAEDVTIGGGLVPKGAT

LTCLIGAAGRDERRYTDPDVFDIHRTDLSTTTAFSAAADHLAFALGRHFCVGALLAKAEV

EVGVGQLLDALPDLTLAPDAHPREQGVYTRGLAALPVRFTPAG

>CYP157B17(2641174774)SALL

VNDPQHSTTGGCPLRAGASGTDAVPLYGAGLSGDPARLYREMRARHGTVAPILLDGDVPG

WFVLGYRELHQVTSNPELFARDSRRWHAWDQIPADWPLLPFVGHQPSVMFAEGPEHRRRA

GAISDALTAIDQFELRQICERLADGLIDAFAGSGEADLMARYAARLPLLVIAELFGFPHT

EVPELAADIAASLNEDEGAIAAHQRVAERMQRLVKAKRSAPGADVPSRLLAHPAGLAEGE

VVIDLLVVMAAAQQPTANWIGNTLRLMLTDDRFALSLSGGRRSVGQALNEVLWHDTPTQN

FIGRWAVRDTQLGGRRIRTGDLVILGLAAANTDPQVRPDFSDAGSDGNQAHMSFSHGEHS

CPYPAPEIAEVIAKAAVEVLLDRLPDVMLAVPTEDLVWHPSLWMRGLVSLPVEFTPAYTP

IPAMGTASARRS

>CYP107DW1(2641174983)SALL

MSEVIDLAELAATADLERELARLAAQHGIIRTRQLNQQETWTVLGAGLTRELLSDPRLSN

DVHTHAPHGALVPGLQVMLLEQDDPGHARYRRLVSAAFASKAVRQLEPRIVEISRQLLDK

LGDSGTADFIDAFTYPMPLEVICDLLGVPGEDRDPFRKWAMDISAAPSLEAMQTSAGELF

AYCIGLIGAKREQPTEDLLSELIAARFEDGTGLSDEELSSFAAVLLIAGHDTVTNLLANA

LHDLLTHPEQLAALRADRSLVNQAVEEALRFRGSAMTTVNRVALEDIEAGGVTIRKGELV

RFLLNAANRDVEVREDGHAFDIGRATAQHVAFGMGPHFCLGQRLARQEATIALNEILDRF

PKLELGVSRAEVRWLASDAIRGLEELPLRYARETA

>CYP105B1(2641175246)SALL

MTTAERTAPPDALTVPASRAPGCPFDPAPDVTEAARTEPVTRATLWDGSSCWLVTRHQDV

RAVLGDPRFSADAHRTGFPFLTAGGREIIGTNPTFLRMDDPEHARLRRMLTADFIVKKVE

AMRPEVQRLADGLVDRMTTGRTSADLVTDFALPLPSLVICLLLGVPYEDHAFFQERSRVL

LTLRSTPEEVRAAQDELLEYLARLARTKRERPDDAIISRLVARGELDDTQIATMGRLLLV

AGHETTANMTALSTLVLLRNPDQLARLRAEPALVKGAVEELLRYLTIVHNGVPRIATEDV

VIGGRTIAAGEGVLCMISSANRDAEVFPGGDDLDVARDARRHVAFGFGVHQCLGQPLARV

ELQIAIETLLRRLPDLRLAVPHEEIPFRGDMAIYGVHSLPIAW

>CYP107T2(2656589136)SAMB

MTAPTLDELAPAGHDPVADPYAVLAALRAEGAVHRIRVPGSGEAWLVVTRDAARAALTDP

RLRNDIRHSASWRTDGGHAIGRNMVQSDPPQHTRLRRLVAGHFTPGRIAALRPRVERVAE

ELLDALPRRGTADLVGRYALPLPVTVICDLLGVPEADREGFHTWSGELVAPTSAEAASTA

SEALTGYLTELTGRKRRAPDDTLLGELVVAADSGVLTAEELLGMVFLILVAGHETTVDLI

SATVHSLLTHPGLLDLLRADPGLTGSAVEESLRFNSPVHSTAFRYAAAPLELAGTRIAAG

DSVLVSLAAASRDPGHFPDPDRFDIGRRTAGHLGFGHGLHHCLGAPAARLEAAVAVRLLL

RRHPSLSLAADPATLTWRTGTLLRGLTELPVRLG

>CYP107P14(2656589229)SAMB

MTALSDLAFDPWDPAFVADPYPAFAELRSRGRVHYYEPSDQWLVPHHADVSALLRDRRLG

RTYRHRFTHEDFGRAPHPPEQEPFHTLNDHGMLDLEPPDHTRIRRLVSKAFTPRTVERLK

PYVCGLADDLVDRLVAAGGGDLLRDVAEPLPVAVIAEMLGIPESDRGPLRPWSAEICGMY

ELNPSEETAAKAVRASLDFSDYLRGLIAARRKEPGEDLISGLIAAHDEDDRLTEQEMIST

CVLLLNAGHEATVNATTNGWLALFRNPDQLAALRADHSLVPSAVEELMRYDTPLQLFERW

VLDEIELDGTVLPRGAEVALLFGSANHDPAVFTAPDRLDLTRRDNPHISFSAGIHYCIGA

PLARIELAASMRSLLERAPALRLAAEPRRRPHFVMRGLEELTVET

>CYP170A21(2656590210)SAMB

MTVQSSPESPASAGQSATASPAVRDEATPELRRPPVAGGGVPLLGHGWRLARDPLAFMSQ

LRDHGDIVRIKLGPKTVYAVTAPELTGALALSPDYHIAGPLWESLEGLLGKEGVATANGP

LHRRQRRTMQPAFRLDAIPAYGPIMEEEAHALAERWQPGRTVDATSESFRVAVRVAARCL

LRGQYMDERAERLCVALATVFRGMYRRMVVPLGPLYRLPLPANREFNNALADLHLLVDEI

IAERRASGQKPDDLLTALLEAKDDNGDPIGEQEIHDQVVAILTPGSETIASTIMWLLQAL

ADHPEHADRIHDEVRAVTGGRPVAFEDVRKLTHTNNVIVEAMRLRPAVWVLTRRTVVETE

LGGYRIPAGSDIIYSPYAIQRDAKSYADNLRFDPDRWIPERATDVPKYAMKPFSVGNRKC

PSDHFSMAQLTLITAALASKYRFEQVAGSNDAVRVGITLRPHDLLVRPVAR

>CYP113B4(2656590810)SAMB

MGEAVTGPMELSKDADARGLLEWFAYNRTRHPVFWDETRQAWQVFGYDDYVTVSNNPQFF

SSDFNMVMPTPPELEMIIGPGTIGALDPPAHGPMRKLVSQAFTPRRIARLEPRVRAITEE

LLDKVGQQDVVDAVGDLSYALPVIVIAELLGIPAGDRDLFREWVDTLLTNEGLEYPNLPD

NFTETIAPALKEMTDYLLKQIHAKRDAPADDLVSGLVQAEQDGRRLTDVEIVNIVALLLT

AGHVSSSTLLSNLFLVLEENPQALEDLRADRSLVPGAIEETLRYRSPFNNIFRFVKEDTT

VLGPLMEKGQMVIAWSQSANRDPRHFPDPDTFDIRRSDGTRHMAFGHGIHHCLGAALARL

EGKVMLELLLDRVQGFRIDHEHTVFYEADQLTPKYLPVRVDWN

>CYP102B16(2656591867)SAMB

MAGTTNGLAPDGLPKGFRSAELGWPELHRIPRPPYRLPLLGDVVGASRSTPMQDSLRYAR

RLGPIFRRRAFGNEFVFVWGAGLAADLADEERFAKHVGLGVANLRPVAGDGLFTAYNHEP

NWQLAHDVLAPGFSREAMAGYHVMMLDVAARLTDHWDRAGAAGRTVDVPGDMTKLTLETI

ARTGFGHDFGSFERSRPHPFVTAMVGTLTYAQRLNTVPAPLAPWLLRGASRRNTADIDHL

NRTVDDLVRARRAAGGRGGTGDLLDRMLETAHPETGERLSPENVRRQVITFLVAGHETTS

GALSFALHYLAQHPGIAARARAEVDRVWGDTEAPGYEQVAKLRYVRRVLDESLRLWPTAP

AFAREARTDTVLGGSYPMRRGAWALVLAGMLHRDPQVWGPDAEEFDPDRFDAKAVRSRAP

HTFKPFGTGARACIGRQFALHEATLVLGLLLRRYELRPEPEYRLRVTERLTLMPEGLRLR

LERRAGSGRAGTGPDRAAVAEDAASAPRCPVPRADD

>CYP157K5(2656591893)SAMB

MNDDQTPSGPTGGGCPVAHAGGVTRLYGPEAATDPHDIYARLRKEYGSVAPVLLEGDVPA

WLVLGYRENRRVLDNPLQFSRDSRIWRDFRDGRVEATSPLIQMVGWRPDCVSQDGEPHRR

LRGAVTDNLYAVAGRGIRRHVTHFANKQIDAFADTGRADLVADFAEYLPMLVLTRVFGLA

EREGRNLAESSKQVIKGGADALTHNERIMGILGELAARKRQEPGSDFTSGLIEHRAGLDE

DEIVNHLRLVLITAHTTTSNLLARTLQMVLTETSWLSGLVSGQLNLSAVVEEVMWNSPPL

AVLPGRFATADLELGGRPVKKGDLLVLGLAAGNHDPEVRPDIAAPVHGNASHLAFSAGPH

ECPGQNIGQAIIETAVDVLLHRLSGLRLAVPPAGLTSTASTWESRLDSLPVEFTV

>CYP159A1(2656592091)SAMB

MSAAQQVPDILSAEFAANPYPAYRAMREGAPLFWHEATRSWIVSRYEDVERVFKDRDGQF

TTENYDWQIEPVHGRTILQLSGREHAVRRALVAPAFRGTDLQERFLPVIERNSRELIDAF

RYTGRADLVSDYATRFPVNVIADMLGLDKADHDRFHGWYTSVIAFLGNLAGDPEIAAAGA

RTRVEFAEYMFPVIQERRENPGDDLLSTLCAAEVDGVRMSDEDIKAFCSLLLAAGGETTD

KAIASIFANLLAHPDQLAAVREDRTLIARAFAETLRHTPPVHMIMRQSATEVELSGGTVP

AGATVTCLIGSANRDESRYHDPDRFDILRDDLTTTTAFSAAADHVAFALGRHFCVGALLA

KAEVEIGVGQLLDAMPDLTPADGFDAVERGVFTRGPQSLPVRFTPRAD

>CYP107EF1(2656592246)SAMB

MSVLYDPNDPALAADRFAYYKRLREADPVHEIAAGYWLLTRYDDVEALLRSPHSSSAFPK

DPGWAMSRGGPTCPAMRSVSKWLLLQDGAEHRRLRKLIARVFTPRYIDRLRPRIGEIVDG

LLDSMGEGEVDLIRDLALPMPVAVVGELLGIPVEDRARCRDWTDKVGYILDPDVTPRRRI

AMNKAEPEFRAYLLDLMASRRGADPDHDLLSVLMTAEDDEQLTEEEVVANILLIFNAGHE

TTVNLIGNGMLALLRQPEALEALRADPGLMATAVDELSRFDPPVTLSSRIATAEMEFGGK

VIPPGSHVIGFLDAAGRDPERYPDPDRLDLSRTEPKTLAFSAGPHFCLGAVLGRLEAATV

FSKLLERYTKIELLREDLPVNSHFNLHGLLELPLRLSR

>CYP107EP1(2656592247)SAMB

MSELEAERRAHYQATVVQGALRTAAAGGDPYASILLVPDDPYPFYERVRERGTLHRSAVG

VWTTTSHRVANQILRDRRFGVRTPEGEKPPEFMPFDNSMLGLDPPDHTRLRRLATPSLNP

RRLAHWQPHVERFTDELIDEMLAGRGQVNFMRAFAQQLPLRVIGDLVGIPPHHRQSFFRL

SRRMAYLLDGVATAPAARGVVAAMAEMTVMFHEIIAERRADPREDLISDLLPAVEDGRMT

MDEMVPLCMFLPLAGTETTVNLIGNGLLALLEHPEQWDMLVADPSLADAVVRETLRYDPS

VQQYRRIAHTDLQMEGASIAAGEEVAICAGGANRDPEVYPDPGRFDITRDPGPENLAFSA

GIHFCLGAALARMEAETALAALAVRVPGIRRAGPVRRRGSFIVRGMLQFPVDLR

>CYP154K2(2656592567)SAMB

MVSVQGCPYAFDASGGDVQGEAARLRERGVVAAAVLPGGVGAWAVTGAEEIRALLTDGRV

SKDAYRHWPAWREGRVEQAWPLAIWVSVRNMVTAYGADHTRLRRLVASAFTVRRVEVLRG

RVEEITASLLDALQERPGGGPVDVRREFACLLPMQVLTELFGIPVAYRERLRRIILGFFD

TAVSLADAQRNAADLYQMMDDLVACKRRVPGDDLTSALIAVRDEDGSRLSERELVDNLIL

LYTAGYETTVNLLDNTIALLLAHPGQLELVRSGVAGWDDAVEEALRLEAPGANGILRFAV

EDVEVGGVVIPAGDPVVISYAGAGRDPVVHGEDAGRYDVTRATRRSHLSFGHGTHYCIGA

PLARMEAQIALSGLFTRFPGLQLAVAYEELRPLQSFISNGHRELPVLLGPAAAAGAGAGE

GRAGEQPAARPVPAATA

>CYP154K2(2656592960)SAMB

MVSVQGCPYAFDASGGDVQGEAARLRERGVVAAAVLPGGVGAWAVTGAEEIRALLTDGRV

SKDAYRHWPAWREGRVEQAWPLAIWVSVRNMVTAYGADHTRLRRLVASAFTVRRVEVLRG

RVEEITASLLDALQERPGGGPVDVRREFACLLPMQVLTELFGIPVAYRERLRRIILGFFD

TAVSLADAQRNAADLYQMMDDLVACKRRVPGDDLTSALIAVRDEDGSRLSERELVDNLIL

LYTAGYETTVNLLDNTIALLLAHPGQLELVRSGVAGWDDAVEEALRLEAPGANGILRFAV

EDVEVGGVVIPAGDPVVISYAGAGRDPVVHGEDAGRYDVTRATRRSHLSFGHGTHYCIGA

PLARMEAQIALSGLFTRFPGLQLAVAYEELRPLQSFISNGHRELPVLLGPAAAAGAGAGE

GRAGEQPAARPAARPVPAATA

>CYP1417A1(2656593308)SAMB

VTTPPFTPTPTAPGSLPLIGHAHRLARTPLPFMASLREHGSVVRIRIGTAPAYVVTDPAL

TRKVLVTDAADFTKGGKIIDALRVFFGDGLATVADGDVHLRNRRLMQPMFNKAHIATRGD

AMINQVQSMVDAWEDGEHRDVFADMNNLTLAAFLVALFGTDLPPHLQEEFTALMPAIMKG

TIRQTILPPWANRLPLPANRAHAARVARLRTLIDQAIDHHTSRLSTTSATTTQATGCPHA

DRPTGLFETLLTAPEPLSRQQLQDEAITLLTGAIETTGTTLAWSLYEISQHPHVEQRLRA

ELTSVCQDRPLRYTDVDQLHYARSVLQEAVRKYGPAWMVTRTATRDTKLGGHAIPAGADV

VWSPYLHQHDPEVFPDPERFDPDRWTRGEAAATRGSFLAFGDGRRKCIGENFAWAELQII

LATILQNCSHLQLTTRPPRPQAVVTVKPDRLTMSYSRPTTTRNEDDEGQAHCPTAQPSTP

>CYP156B15(2656593429)SAMB

MNAHDTFPVPPPGCPAHGSGARVPLHGPEFAADPQAFYEYLRHYGVAAPVELAPGVEATL

VTDYSAALQLLQDSGSFRKDARRWRAFNEGKVGPDSPVAPLLAYRPNCMFADGADHLRLR

QAVTDSMARVDTRRLTRSTEQISGYLISQFGGRGSADLLGDYARQLPLFVFNELFGCPAD

IGDRVLFGISGMFDGINADKATAVLFQAVGELVALKRSKPGDDVTSWLMRHEADLSDEEM

VHQLALLLGAGAEPLRNLIGNTLHRLLTHERYAREGGLIDEALDDTLWENPPMANYAPHY

PAADTELAGQQLHSGDLVLVSFAAANTGPALKASRQAGSNRAHLAWSAGPHACPSKEPAR

HITVTAIEHLFNELPDVELAVPEDSLTWRPGPFNRALATLPARFTPARTGRRSAAQPDPR

APEREQAPSPDRPAERGGMWSQFLNWLTR

>CYP157A1(2656593482)SAMB

VSTDATDAVPLSGPRFQTEPALLYRQMRREHGAVTPVLLDGDVPAWLVLGYRELHQVTGD

PVLFSRDSDLWNQWENIPGDWPLLPMIGRKQPSILYTVGERHRERAAMISDALEAVDPHV

LRGHAERFADELVDRLCAKGEADLVGDYAMLLPVRVLTRLYGFSDEEGPALVTALNDMID

GRERAIAGQTHLGTSMARLLADRKAAPADDVASRMLADDSGFSEEEIAQDLMVMMAAGHQ

PTADWIGNSLRLMLTDERFAASLFGGRNSVAEAMNEVLWEDTPTQNVAGRWASRDTQLGG

RRIRAGDLLLLGLQGANSDPQVRTDGSALTGGNNAHFSFGHGEHRCPFPAQEVAEVIART

GIEVVLDRLPDIDLAVPAGSLTRRPSPWLRGLTELPVRFTPTTALGGTSA

>CYP154C1(2656593483)SAMB

MTTGTEEARIPLDPFVTDLDGESARLRAAGPLAAVELPGGVPVWAVTHHAEAKALLTDPR

LVKDINVWGAWQRGEIPADWPLIGLANPGRSMLTVDGAEHRRLRTLVAQALTVRRVEHMR

KRITELTDRLLDELPADGGVVDLKGAFAYPLPMYVVADLMGIEEARLPRLKVLFEKFFST

QTPPEEVVATLTELAGIMADTVAAKRAAPGDDLTSALILASEDGDHLTDEEIVSTLQLMV

AAGHETTISLIVNAVVNLSTHPEQRDLVLSGEADWSAVVEETLRYSTPTSHVLIRFATED

VPVGDRVIPAGDALIVSYGALGRDERAHGPSAGEFDITRTSQNRHISFGHGPHVCPGAAL

SRLEAGVALPALYARFPRLDLAVPASELRNKPVVTQNDLFELPVRLV

>CYP154U4(2656593539)SAMB

VTVTDRIALDPFGADIPGESARLRALGPMVPVELPGGIPAWAPTRHDTLRELILDPRVSK

DPRLHWRLWPEIGEHPSWAWIIGWVGVVNMLSTYGPDHTRLRRLVAPSFTHRRTEAMRPR

VEAITTGLLDALEASRGDRTDLREAFAHPLPMRMICELFGVPEEMWEDTGRLIAAVMDTS

DPGPEHAASVQRQIGTVLPTLIAHKSEHPGDDLTTELIRVRDEDGDRLSDEELLYTLLLV

IGAGFETTVNLIGNAVVALLTHPAQLAAVRSGRIGWDAVVDETLRVHPSIASLPLRFAVT

DIAVGGVTVPAGDAIVTTYAAAGHDPAHHGPDADVFDAARGTDDHLAFGIGVHRCVGAPL

ARAEALTALPALFARFPEIRLAVDPGELRQVPSFIAYGWREIPVRLGG

>CYP158A17(2656593962)SAMB

VSEDTLDRAESAPPVRDWPANDLPGTDFDPVLRALMREGPVTRVSLPDGEGWAWLVTRHD

DVRLVATDPRFGREAVVDRQVTRLAPHFVPARGAVGFLDPPDHTRLRRSVAAAFTDRGVE

RVRGRSRGLLDELVDAMLEAGPPADLTEAVLAPFPVAVICELMGVPDSDRHSVHTWTRLV

LSSSHGAEVSQRAKHEMGAYFADLIGARSDATGEDVASLLGAAVGRNEITRDEAVGLALL

VQLGGEAVTNNSGQMFYLLLTRPELAERLRAEPEIRPRAVDELLRWIPHRNAVGLSRIAL

EDVEIKGVRIRAGDAVYVSYLAANRDPAVFPDPDTIDFARGPRPHASFGFGPHFCPGGAL

VRLESELLVGTVLDRVPGLRLAVAPQDVPFRKGTLIRGPEALPVTW

>CYP157C1(2656594353)SAMB

VTPERHSLTGTGDPLLEPPPGCPAHGLGPGGLHRLHEADDLEELYEKLREQHGPVAPALL

HDDVPMWVVLGHAENLHMVSTPTQFCRDSRIWTPLNEGMVKPDHPLMPHIAWQPICSHAE

GEEHKRLRGAVMGAMAGLDYRELRRHIKRYTQRLVNRFCEEGRADLVGQFAEHLPMGVMC

HLLGMPDEYNDRMVEAARDTLKGTDTAIASHAYLMESLGRLSAERRERPENDIAGRLVTH

PAGLTDDEVREHLRLVLLAAYEATVNLISNVLRVVLTHPGFRAQLSGGQMTVFEAVEQSL

WDEPPFSTVFAYFAKQDTELGGQRIRAGDGLLLGIAPGNVDPRIRPDLAASMQGNRAHLA

FGGGPHECPGQDIGRAIADAGIDALLMRLPDVQLDCDEDDLQWRSSIASRHLVELPVRFE

PKAQQDIRQQPSHAPAPAQRTAWHVGIPRPEERPAPQAPPRPPEPVSLAAARPQPVPEQP

RPRGAWQRFLLWWRGY

>CYP107U1(2656595775)SAMB

VTGHPPRPGAGGPVPELFTWEFASDPYPAYAWLREHAPVHRTRLPSGVEAWLVTRYADAR

QALADPRLSKNPAHHAEPAHARGKTGIPGERKAELMTHLLNIDPPDHTRLRRLVSKAFTP

RRVAEFAPRVQELADGLIDRFAATGSADLIHEFAFPLPIYAICDLLGVPREDQDDFRDWA

GMMIRHGGGPRGGVARSVKKMRGYLADLIHRKRAALTPEPGPGEDLISGLIRASDHGEHL

TENEAAAMAFILLFAGFETTVNLVGNGTYALLTHPEQRARLQSSLADGERALLETGVEEL

LRYDGPVELATWRFATRPLTLGGQDIAAGDPVLVVLAAADRDPERFTDPDTLDLSRSDSQ

HLGYGHGIHYCLGAPLARLEGQTALATLLTRLPDLSLAADPAQLRWRGGLIMRGLRTLPV

SFTPVAPMAGDVSTSPQK

>CYP171A1(2712577666)SMA

MSQSTSSIPEAPGAWPVVGHVPPLMRQPLEFLRSAADHGDLLKLRLGPKTAYLATHPDLV

RTMLVSSGSGDFTRSKGAQGASRFIGPILVAVSGETHRRQRRRMQPGFHRQRLESYVATM

AAAAQETADSWSAGQVVDVEQAACDLSLAMITKTLFFSDLGAKAEAALRKTGHDILKVAR

LSALAPTLYEVLPTAGKRSVGRTSATIREAITAYRADGRDHGDLLSTMLRATDAEGASMT

DQEVHDEVMGIAVAGIGGPAAITAWIFHELGQNAEIESRLHAELDTVLGGRLPTHEDLPR

LPYTQNLVKEALRKYPGWVGSRRTVRPVRLGGHDLPADVEVMYSAYAIQRDPRWYPEPER

LDPGRWETKGSSRGVPKGAWVPFALGTYKCIGDNFALLETAVTVAVVASHWRLHALPGDE

VRPKTKATHVFPNRLRMIAEPRSVVRLEEPAAMGA

>CYP298B1(2712577881)SMA

MMQQRGRSHKNWFQNDRNLNFHMLLQNGTSSMVIFDPLAPEMLEDPYSTYAILRSGDPVH

WHDGLKAWVLTGHRDCLYVLQNPDSFSCDFRKIGEVTPPEFLSIQTVDPPLHDSIRKRLI

SALRRMDTGSWLDGVIATAEKLTWEVDHDGFDFIEFLEQVSTHAMCTFLGIPVPADQAAT

RAAQRDLTLSMDAGLEPGRASAGRAARAFLSEMIDPYLDKSRPDSFIGHIDHDRHTDMRH

YLVNSLRAFFVAGSSSTSSTLGNITDTLLRHGLLAETEPMAIDAAAVNELVRYSGAVQAV

SRAVVREICLPSGHQIHEGDVVVAVVASANRDQEVFRDADELRLDRSPNPHLGFGRGVHA

CTGAHLALTLGSRMLTWLSHNFVMTPAGPPERRPTATMRGLDCLPVRLVRR

>CYP1339B1(2712577900)SMA

MPRRFPGPRGKWLVGNMAPYLADRIGWLRATQKEYGDIVRLGPDTLVVHDPELAHEILAR

TNDTYLLETSQLAGRRQRAAAQARLDTWMAVRQGMWHGFAGRLAEVHLARLMAQVGLLLD

LHAGDDKDLVERCRATTGRLIVDFCIGGDPRHAALCDEAVTRADLLFTSAQNALVQKETR

RRFHRRPLASAASTANAELLGFLHDSVAERRSRGYSGSPRDLLDSLLHASGPDDTNDLIV

SVLRMAMFASHGVPGAALSWIVLRLASEPGALQAVRKEAETCPADGALSIERLPLTTAFV

KETLRLHPPQWLLTRTAIRRTTVGAYQVRAGQEIFVSPYVMHRDGRFWPRPERFELERWT

GDRIPYPRHTYLPFGAGPRICPGSRLALAHLVILTMRLAADHVPQAPSIEDVNVTCDGLL

LPANVRGGWTYRTS

>CYP154A2(2712577936)SMA

VPLQKLVHKTLPQIHQAAGRIADLHAGDACVREALDTVLYRDPPLANVAMAYPPRPLDIG

GFLLPTGQPAVISCAACNNGPALGTQRPLGNRAHLAWGAGPHRCPAGAHACLIAEMPSMS

WTPWPRRTSTAGAPASPGAPAPSTARWSPCPSLSPRAERDLNVSEQQIIVLDPTGSDPDA

EHQALRERGSAALVDILGVRAWSVSDPALLKQLLTSKDVSKDGRAHWPAFAETVPTWPLA

LWVAVENMFTAYGDNHSRLRRMVAPALSARRTAALQADIEALVNSMLDGLDAFPAGEVAD

LRKHLAYPLPIAVIGKLMGVPADRRTEFRTVVDNVFATHLSAEEQAANTAALYALLDALI

EIRRAEPGQDMTSLLIAARDEEDGSAFSDAELRDTLLLMISAGYETTVNVIDQAITTLLT

DPCQLAHVREGRCTWQDVVEETLRHQPAVKHLPLRYARIDIPLPDGQTIKTGEAILASYA

AANRHPTWHEDADRFDATRPSKEHLAFGHGVHFCLGAPLARLEVATALRLLFERFPDARL

ATERTALEALPSLISNGHTSVPVHLRPTSSASS

>CYP182A1(2712578034)SMA

MRSGMEAQPYATAPAGSPRLESLRVEPLLTSEFDADPAGVYERLRRTYGPVAPVGLMGVP

VWLVLDYHEVLEVLRDDSQWRRDIRYWRARAQGQLPRDWPLLAGYEVRQTMFLDGDEHHA

ARLTHHSALRPFQDGHSPQGWELRAAVARYADELIALLAAESGSAGYADLGAQYTRPLLL

MVTGKLFGCPVGLGDELVMDLWRMLDGGPDAGPATDRAAAVMTRLAAHRRQRPGDDLTSY

LLLADPGMTDEQLGRELLMNAVYLNDITGNMVMNTLLEVLRGNATVRRSVSDGHLGETFH

RVALVNPPVANMCFRFAAGDVRLGAFWIRAGDVVSPSAAAAHQDLLTIGTSPLGDATAST

RAHLGWGAGQHQCPGAARELAGMIVTTAVGRIFDHFTKAELTLPADQLPWRSGPVVRGLR

LLPVRYELRGTPSESHPVPPPRHDGEAPDTAPTADHRSRGLLSALRRLMTGTGRAG

>CYP107W1(2712578125)SMA

MAEAPSEPIAFPFPDPPSVCELPPELAEIRDGQSVVEVKFPDGISGWMVTKHADVRKVLV

DSRFSSKVMATAAAAMSETETGKLMNESLVGMDAPEHTRLRKLVTKAFTARRVETLRPRI

TELVGQLLDELETLPRPVDLVKNFSVPLPVRVICELLGVPAGDQDTFHAWSNALLGDWQQ

VVEKEAATVSLVNYFGELIAVKRENPADDLISELIAISDGDSTLTEREIIALSIGILSAG

HETTANQISMFLVTLLHNPEELDKLRDNREAIPKAVDELLRFVPLTTTGGIIPRLTTAEV

ELSGGQVLPAGAVVLPAVATANRDPEVFEDGERLNVTRENNPHLAFGAGIHHCLGAQLAR

IELQEALGAILDRMPQVRLAVPESELRLKSASIIRGLESLPITW

>CYP1453B-fragment(2712578143)SMA

MWSPPLILVRRARASFDLGTTRLEKAQNYLVSPHMIHRDHRYWQQPDTFDPDRFLPGVPH

GPTDRSCYVPFGWAPKKCIGNDIGTTQLMGLCYLICTRYRLSVPNSDTLPMACRFAPVPQ

RFNGRLALAWN

>CYP107L56(2712578229)SMA

VPKDPRALTSDGAVTELLPELTPDPAQFTADPHTRYAQLRAEHPVQRVRLPHGAAAWLVT

GGDEVRTALTDPRLRNDIKHSGTWQADGGFAIGRNMLQVDPPDHTRLRRLVAGTFTHRRI

QAMRPRVQRITDDLLDRVVPLGSADLVEALSFPLPVTVICELLGVPEADREAFRAWSAHM

VAATDPQAATAAGQKMTEYLAGLIDDRRHATASAPDDNRNGPLSAHGAHRPVDGGEGGEG

GEGGHDVLTALVRARDEEHGALSADELLGMAFLLLVAGHETTANLISSAVFLLLRHPDQL

AALRADPSLMTGAVEETLRYEPPALAVAYRYAAERLTLGGVDIPKGDPLVLSVAAANRDP

AHFTEPDRFDIRRDPATTAAHLSFGYGIHHCLGAPLARLEASIALYTLLRRCPDLRMNTE

AGDPAWRPSLLRGLDRLPVRW

>CYP147F32(2712578293)SMA

MTQAILRQIIDYAHRADPYPLYEELRKTPVYHDADGPYVVSKYYDIRSLLHDPRISSEAR

NLKATAADPLADKEEETALPPSFLRLDPPEHDRLRRMTNRSFGPPHCPRRVYDMHGDLAD

IVSGLIDGIDTTERIDLVDQFSYPFPVTVICRLLGVPREDEARFHSWADTIAAGLDPDPG

ADPTERAKVSNNARTELGMYLAGLIEERRKKPGDDMLSELATAPGPDGVMTTMEVLSTAA

LLLIAGHETTVNLITNGMLTLLRNPDVLQRLRKDPKLSVPIVEELLRFEPPVQLVPQRTT

LADIEVRGVTIPKGASLWLVLAAGNRDPDRFEHPERFDPDRGDIQHLGFGSGIHGCFGAP

LARLEAQLALSELARRLENPRLLEDPPPYRQNAVLRGPRHLPIACDGIRP

>CYP158A26(2712578328)SMA

MTSQPRTERPPVHVWEVEDLPALEFDPMMTTLLRDEPVSRIRLPFAGDMDAWLVTRYEDV

KAVASDPRFSREALRDIKVTAISGHRVAGAAALNYTDPPYHTKLRKIVNKAFTGRHMKGL

RPMTQQTADELLDAMEEQGPPADLMTHLHGPLPLAVVSDLLGVPREERDKFTVWPDQILN

AGIGAEASMAAKAEVTAYVVELLRSRFGRAAEGTDDLAGVLAQAWEAGEIEQDEAVSLAT

AIVISGAHAVRYNSANMVYMLLTHPELMDRLRDDSGLVPQAVDELIRHIPHRNAVGIPRI

AMEDVEVGGHVIPAGDAVYVSYLAANRDPAVFENPDAVDFDRQGVSHLSFGHGVHHCMGA

MLARMESEVMVSSLLDRFPKLRLAGPPQDTVFQSKAFIRGPQTLVVTW

>CYP105P1(2712578694)SMA

MPEPTADAPTVPKARSCPFLPPDGIADIRAAAPVTRATFTSGHEAWLVTGYEEVRALLRD

SSFSVQVPHALHTQDGVVTQKPGRGSLLWQDEPEHTSDRKLLAKEFTVRRMQALRPNIQR

IVDEHLDAIEARGGPVDLVKTFANAVPSMVISDLFGVPVERRAEFQDIAEAMMRVDQDAA

ATEAAGMRLGGLLYQLVQERRANPGDDLISALITTEDPDGVVDDMFLMNAAGTLLIAAHD

TTACMIGLGTALLLDSPDQLALLREDPSLVGNAVEELLRYLTIGQFGGERVATRDVELGG

VRIAKGEQVVAHVLAADFDPAFVEEPERFDITRRPAPHLAFGFGAHQCIGQQLARIELQI

VFETLFRRLPGLRLAKPVEELRFRHDMVFYGVHELPVTW

>CYP105D6(2712578695)SMA

VSPDGRRAVPFTEAAVTERGDGSYSVARHASGVRSVTEYSDGLPVDRGAPRSPRQRPTCP

TKGPGPDGRVPTGPRTALLTDRNLFPVAHHAPMNAALPMFERHDMTETEIRLTGSPAPSF

PQDRTCPYQPPKAYEERRGESPLTQVTLFDGRPAWLITGHAEGRALLVDPRLSSDWGHPD

FPVVVRRTEDRGGLAFPLIGVDDPVHARQRRMLIPSFGVKRMNAIRPRLQSLVDRLLDDM

LAKGPGADLVSAFALPVPSVAICELLGVPYGDHDFFEECSRNFVGAATSAEADAAFGELY

TYLHGLVGRKQAEPEDGLLDELIARQLEEGDLDHDEVVMIALVLLVAGHETTVNAIALGA

LTLIQHPEQIDVLLRDPGAVSGVVEELLRFTSVSDHIVRMAKEDIEVGGATIKAGDAVLV

SITLMNRDAKAYENPDIFDARRNARHHVGFGHGIHQCLGQNLARAELEIALGGLFARIPG

LRLAVPLDEVPIKAGHDAQGPIELPVVW

>CYP107P2(2712579292)SMA

MAAAFDLAFDPWDPAFLADPYPAYADLRAKGRVHYYEPTNQWLVPHHADVSALLRDRRLG

RAYQHRYTHEDFGRTAPPAEHEPFHTLNDHGMLDLEPPDHTRIRRLVSKAFTPRTVEQLK

PYVAKLAGELVDRLVAAGGGDLLADVAEPLPVAVIAEMLGIPESDRAPLRPWSADICGMY

ELNPPKDVAAKAVRASVEFSDYLRELIAERRKEPGDDLISGLIAAHDEGDRLTEQEMIST

CVLLLNAGHEATVNATVNGWYALFRNPDQLAALRADHSLVPAAVEELMRYDTPLQLFERW

VLDEIEIDGTTVPRGAEIAMLFGSANHDPEVFRNPEKLDLTREDNPHISFSAGIHYCIGA

PLARIELAASMTALLEKAPTLGLVAEPKRKPNFVIRGLEGLSVAV

>CYP105D7(2712579549)SMA

MTEPGTSVSAPVAFPQDRTCPYDPPTAYDPLREGRPLSRVSLYDGRSVWVVTGHAAARAL

LSDQRLSSDRTLPRFPATTERFEAVRTRRVALLGVDDPEHRTQRRMLVPSFTLKRAAALR

PRIQETVDGLLDAMEAQGPPAELVSAFALPLPSMVICALLGVPYADHDFFESQSRRLLRG

PGIAEVQDARAQLDDYLYALIDRKRKEPGDGLLDDLIQEQLNRGTVDRAELVSLATLLLI

AGHETTANMISLGTFTLLRHPEQLAELRAEPGLMPAAVEELLRFLSIADGLLRVATEDIE

VAGTTIRADEGVVFATSVINRDAAGFAEPDALDWHRSARHHVAFGFGIHQCLGQNLARAE

MEIALGTLFERLPGLRLAAPADEIPFKPGDTIQGMLELPVTW

>CYP102B2(2712579593)SMA

MAQSTRTVIPKGFRSAELGWPELHRIPHPPHRLPVVGDVLGVNVRTPVQDSLRIGRRLGP

VFRRKAFGKEIVFVGGADLAAELADESRFAKHVGLGVANLRPVAGDGLFTAYNHEPNWQL

GHDVLAPGFSREAMAGYHPMMLAVTERLIDHWDREQTAGRAVDVPGDMTKLTLETIARTG

FGHDFGSFERARPHPFVTAMVGTLTYAQRRNVVPEPLAPLLLRTATRRNAADLAYLNRTV

DALVRARRTTSGEGDLLDRMLDTARPGTGERLAPENIRRQVITFLVAGHETTSGALSFAL

HYLSRHPDVAARARAEVDRVWGGTARPGYDQVAKLRYVRRVLDESLRLWPTAPAFAREAR

RDTVLGGVHPMREGAWALVLTAMLHRDPGVWGADAERFDPDRFDAQAVRSRAAHTFKPFG

TGARACIGRQFALHEATLVLGLLLRRYELRAEPGYRLRVAERLTLMPEGLRLRLDRRVPA

VEDVPVANPEVSSGPRCPVTGAGE

>CYP107W1(2712580055)SMA

MAEAPSEPIAFPFPDPPSVCELPPELAEIRDGQSVVEVKFPDGISGWMVTKHADVRKVLV

DSRFSSKVMATAAAAMSETETGKLMNESLVGMDAPEHTRLRKLVTKAFTARRVETLRPRI

TELVGQLLDELETLPRPVDLVKNFSVPLPVRVICELLGVPAGDQDTFHAWSNALLGDWQQ

VVEKEAATVSLVNYFGELIAVKRENPADDLISELIAISDGDSTLTEREIIALSIGILSAG

HETTANQISMFLVTLLHNPEELDKLRDNREAIPKAVDELLRFVPLTTTGGIIPRLTTAEV

ELSGGQVLPAGAVVLPAVATANRDPEVFEDGERLNVTRENNPHLAFGAGIHHCLGAQLAR

IELQEALGAILDRMPQVRLAVPESELRLKSASIIRGLESLPITW

>CYP184A1(2712580602)SMA

MAAVTAAGAAQAPIVAGHPLLGSMNDLLNDPLATYLRARRDHGDVVRFRAGPPGLRADIY

AVFSAEGAQQVLATESGNFRKDNVFYGELRDSVGNGLLTSQDATYLRQRRLVQPLFTRRR

VDGYAGQVADEAAGLAEAWRGIPGGGVELVGEMHRFALRVVGRILFGTDMETTFEVIERT

LPLLQEYALKRGLAPVRTPRTWPTPANRRAARTQAELFALCDGIIDSRRNRKNEGDGGEE

SGEDLVTLLVRAGNAEDGSLDAAELREQVLIFLLAGHETTATALAFALHLLARHPEQQRR

VRDEADRVLGGPGGRAPTAADMEALPYLTMVLKEAMRLYPSAPVIGRRAVADAEVDGVRI

PAGADLFVSPWVTHRHPDYWPDPERFDPERFTPEAEAGRPRYAWFPFGGGPRACIGQHLS

MLESVLGLAVLIREFEFEAVGEEEVPLGAGITLLAKGPARCRVIPRSSGPRSS

>CYP184A15(2712580753)SMA

MSATSQDGGLELIRKIRSLPAKRQRALIALLRQQGVDLSALDGIPILPRSADEPVQLSFT

QQRLWFLAQLDGSSPAYNVPVAMRLRGPLDRPALLRALDALVQRHESLRTRFVDRDGVPY

QHVGDGRDFTVREEEPANPADVALICAEEAAAPFDLERDPMIRVRLMRQAEDEHVLMVTM

HHGVSDGWSVGVFIRDLAALYEAFRAGLPSPLEPLPVQYADYAHWQRQWLVDDVQTRQVE

YWRKRLAGIDPRLTLPVDRERPAVKTYRGARERFRCPADLLDRLREVAARHDATLYMTLL

AAYSVVLHRHTHQSDIAVGTVVANRNRIEVEGLIGFFANTLVMCADLSGDPAFTELLAQV

KKTALEAYDHQDVPFEAVVDALQLERSLSHSPVFQTMFVLQEAETGQEFRPGRLEVSAIE

VDVDFTKFDLTLDLRETPDGLVGTVEYNTDLYDRETIQRFVGHYTELLAAIAADPTARTS

RLGMLGAAERHQVLKEWNDTDRPFSDDRCLHQLFEQAVERHPDRTALLEGDRSWTYAQLN

AWANRIGHALRRRGVGPDTQVGLCLERSAEMVAGIFGILKAGGSYMPIDPSYPAARIAEL

VKNSGTRVVLTQPHLDAEPLGGTVEVLTLHRDGRILDRDGQAAAYAETDIPVAELGLGPE

HLAYVIHTSGSTGRPKGVMIEHRAAVNRIEWMQNEYGLTADDVVLQKTPFSFDVSVWEFF

WPLLFGARLAVAEPGGHKDPGYLVEAIQQFGVTTLHFVPSMLRSMVAEPGWSQCTTVRQV

FCSGEALPPELCERHYARHTAPLHNLYGPTEAAVDVSHWTCPAARLPRTVPIGRPIQNIR

LYVLNDVLEPQGIGCVGELYIAGAGLARGYLHQPELTRERFVPNPFDDAPGARMYRTGDL

ARWLPDGTLEYLGRADDQVKVRGFRIELGEIEHRLAEHSSVRACAVVVREDQPGNPRLVA

YTVLDGEPELADHRAVLTGHLERSLPEYMVPSAFVVVDALPVTAHGKLDRKALPAPGIED

FAQGAYAAPGTETERFLASLWAELLGFDEERISTDDNFFALGGHSLLITVLVARLKAHGL

RATVRDVFSASTLAGLAAVIDRTEDGTDFTVPPNLIPPGCERITPDMLPLVDLSQEQIDS

IVTTVPRGASNVQDIYPLASTQEGILFHHLMDPDNDPYLVSTLYVADDETACTRFTEALQ

AVVDRHDAMRTAVVTDDLSEPVQVVYRTAAPTVERTRLDPDGDPEQQARALLDRFGRMAI

DRAPLLRLVIAEDPNSERRYLLLNAHHLIEDATSLRLTLEELGAHMAGRAELLPPPAPYR

DFVAHVLHQRDPDEAEAYFRGVLGDVTEPTTPFGLTDVRGDGRRVLQHRRSLAPGLTRDL

RAQAQRLHLSPACLFHAAWALVVGACSGRDDVVFGAVMSGRLQGVPGVERMLGNFINTLP

MRTRLAGRTVRELITDVDTALKDLITHEQSPLSLAQRCSDLDGDAPLFSAVVNVRHFEPG

HGEVSVAGINDCGVRFLTAGDAINYPVTVSLDDFGSEFSLDVQVDESVACEAVADYVETA

LSGIVDALATDGGEGTVALDVEVLPAAERRRLLTEWNGSAVDVTATTLLAPFEAQVERSP

DRTALVFEGETLSYAELNARANRLAHWLTEQGAGPERLVAVRMERSFDLVTAVYAVLKTG

AAYLPVEPDLPAERVEQMLADSKPLLVLEELPDTSGHPESNPGVTVSREHPAYVFYTSGS

TGRPKGVVISHRAGLNWLSWNQRRYRLRDSHRLLLKTSVGFDVSVPELFWALQVGASLVI

ARPDGHRDPAYLARLISEQDVTDVYFAPSMLAAFLAEPAAARCTGLRRVEAVGETLPVEL

AERLTRALPGVELYNGYGPTEAGAVTAWQYRAEPGATGVPIGTVVPNVQVYVLNPALRPV

PPGVAGELYIAGDGLAHGYLNRPELTAERFVANPFTPGARMYRTGDLAMRRPDGVLEYLG

RIDDQVKVRGFRVEPGEITNVLLSHPAVHGAVVVPQAVGDTRGLVAYVSPDQQWLETVGK

EYTEDLDRWQRHFEDEYTADAPDPAGRHSESGGGPTAGIGAREHVDATVRRIEELRPKRL

LEVGCGTGTLLFRYAPLCASVHALDLSSAALDAVHRGVARRGWSHVTLSQGDALTVSDLA

GQTFDTIVLDSVVQYFPNRHYLDEAIGRLLPLLDEGGSIVVGDVRNLDLFPALACAAERR

RTHGPVTAGALAAQVERARSQESELLVSPTYFAGLTERFPELGTVDLLLRHAAGDDGILA

ADRYDVILTKGVTGTAEALPWLEAATPAALRSLLDGGTPDRFGVGGLSNPRVTDEVRAWE

DLRRGSPSRTVQALPDGDRPSPWVAESVRDLAAVLRHAGRLGYQVSATWSQDRLDGLDLV

FSRHGSPRVRARARYGATRLTNMPQIGRLGPSTARMLKEHLSASLPHYMIPSAFVIMEEL

PVTPNGKIDKRALPVPDEGDVAKEAYVAPATGAQQTLCRIIAEVLGLTRVGLQDNFFDLG

GHSLLATRLILQVKKETDAELPLQLIFSGATVEDLADALEQDPAGPRSDEPDTAADVREP

GAAPLSLQQRDLWFLNRPEHLGTAHYNVQLAFRVEGALDRDAYARAVRSLVERHAVLRTS

YVRHEGVVTQRVNDSAGYEVSVLEAASEEAATEWLRAERARPFAADDTYMIRAYLLALSD

TRHLAVLTRPWGVFDGWSVNIVLAELVETYRALSQGGEPNLPALSLQYADFARRQHRMVD

AAELSRQEEYWRRQLDGLPACLSLRTDYRRPPVKSHQGSSVQLQVPFELLGRLRRVCQEQ

NVTLYMLLLSAYAVLVGGHAEDDDLAIGTAVTNRPGADLEQLVGYCVNLLVLRLDVAPDR

AFSDVLAQARRVTTEAHQNQDLPFAELAGSLAEPDPAHSPLFQVMFNLLPAPATEAGDGG

GATDLEISPLPTEAGTARYDLNLVVRETDSGLEGNLEYSTDLFARSTAEDMARSYEQLLR

RIAAAPESGVTRLRTVEAHTGDRPDGLGRHPTGRSAPADAPACPVTSGPPTSPRPAEEQP

AGTPTADDTPPAQDEPPRPTSKRRHTSMAPTPPTMAGHPVFGSLMDLQRDTLGTYLKAQR

EHGDVVRFTAGLPGMRGEFYAVFSASGAQQVLAASAQTFTKENRFLGELRASFGNGLLTS

MGDEYLRQRRLLQSLFTPRQVDNYGSEITHEAQLLAERWSTVPDATVDVAEEMTGHTLRT

ITRILFGTKSDVDTMVRTVQRNFPLINAYAVKRAFGPFHLSRKVPTPGNLRAAKAHQELY

DVCDAIIAARQAEESEAPTDRHDMLSLLARAQGEDGAPISAKETRDQVLIFLITGHESTA

TTLGMALHLLAAHPEALARAHHEVDTVLAGREPAAADMDDLAYLVRVIKETLRLYPAAPA

QGRVATENAQVGDYAIPKGANVVISSGVVHRHPDIWEDPDRFDPDRFLPEREAERPRYAW

FPFGGGPRACIGQHLATLEAVLTLAVLLQRYSFTPVDLDIPLNTGITLRPTGQVRCKLTP

RD

>CYP154A47(2712580760)SMA

MEAERCPFRIDPGGSDIHAENRLLYRDGPVAKVELPGGVEAWAVGSQQLVKQLLGDPRVS

RSARQHWDAFMRGEIPPDWSLMVWVAAENMFTAYGSEHRRLRRLASGAFTARRIEALRPQ

VEAVVTGLLDELETAPEGTATDLREGYAYAVPIQVIGTLLGVPEERMPDLRTCVKAIFNT

TISPEDARANGERFYGLLVELAVLKQKEPGDDLTSALIAVRDDEDGSALTQEEVVETLMA

LISAGLETTVNLLDNSIHLLLTHPDQLALVRSGHASWSDVVEESLRLQAPVANIPLRFAA

EDIQAGDVLIRKGEAILIGYAAAGRDEAVHGDTAAQFDVTRANKEHVSFGYGVKFCLGAA

LARLEASTALPALFERFPQMALDGNELTPLASFITNGHRTLPVVLRPAAG

>CYP147F33(2712580780)SMA

MTTTETPDTLRRILDYSSRADPYPLYAELRKTPVALQEDGSYVISTYRELAGILHDPHLS

SDVRNLSHPMAAVEGRTTPSFINLDPPEHDRLRRLAMRHFGPPHTPGLVSGMEPDLRAIV

SGLIDGFAGRQQIDIVDDFAYPFPVTVICHLLGVPREDEPRFHVWVNAIIESIDYNPKTD

PQEKLDNGVQATKDLRQYLGGLLEQRHGRPGDDLLTRLANDDGPDGRMTDEEIISTANLL

LIAGHETTVNLITNGMLTLLRHPDVLQRLRAEPDLVVPMVEELLRYEPPVHIIPWRAAYS

DIHVADTIIPKGSQIMLMLASGSRDPDRFDDPDRFDPDRRDNQHLGFGSGVHLCFGGPMA

RLETQIALTELVRRLDRPRLVADPPPYRPSPVLRGPIHLLLEQG

>CYP107F2(2712581251)SMA

MTPSDEAIMCPFDFSKGLEFDPSLAELMARDSITRIRLPYGDADAWLVTGFDAVRQVTTD

QRFSRAGIMGSDYPRLTPEPIVSPESINVVDPPHSSRLRRLVSQAFTKGHVERMRRRIVR

LADTLLDEMAAQGPPADLAHHLSNRLPQHTICDVLGIEQDDWPRMEQYVHQLLSTGPDSR

QSAATAKSELREYFGGLVEKRRSSPGNDLISALAAAKDGEDVLDDQELAVMALTLMLSGH

DTATCQISNICYLLLTRPELMKHLRSRPDTLTAVLNEMLRFIPFRKGVGIPRVAVEDAEV

DGVKIRAGDFVHVSYLTANRDPERYPDPDAIDPDRPSLPHMTFGWGGHRCIAVPLAMAEL

EVAIGRLLERFPELRLAVPPEEVRWDTETIRRFPIELPVAW

>CYP154D1(2712581388)SMA

LDTSSGTQPHRMDPAGGCPHADNARLLARGAVAPVLLPGDIEGMAVLGHDALKEFLAHPE

VAKNARHFTALQEGRTAPGWPLLTFATVQGMTTADGEDHRRLRSLVSKAFTARRVERLRP

WIEELTSALLDGLGRAAEEGGQVADLRAHFALPLPMGVIGELLGVDAEHRDRLHHLSNQV

VATDIGPEQAIAANRELVAVLSTVAAARAERPGDDLTSALIAARDEGGDRLSQQELIGTL

VLMIIAGHETTLNLITNAVRALCGNRDQLELVRKGDAGWPDVVEETLRWDAPVSYFPFRY

PVRDLTVDGTVIPAGTPVLAGYSAAGRDPAAHGPDADRFDVTRPARSETTRHLSLGHGAH

YCLGAPLARMEATIALERLFTRFPDLELAVPEAELARHSSFVGNSVRALPVRPGVPRS

>CYP157C2(2712582201)SMA

VTPEPHPSTGTDDPTFGPPPGCPAHDPGLGGVRRLYGPEAEDLGDLYEKLRAEHGAVAPV

LLHNDVPMWVVLGHTENLHMVRSPSQYTRDSRVWTPVLDGTAGPDHPLMPHIAWQPICSH

AEGDEHLRLRGAVSGAISTIDHRGIRRYINRSSQALVNRFCEEGRADLVGQFAEHLPMAV

MCEILGMPDEYNDRMVQAARDMLKGTETAIASNAYVMDALMRLTARRRAHPEGDFTSHLI

NHPAQLSDDEVGQHLRLVLIAAYEATTNLLANVLRMVLTDPRFRAQLNGGQMTVPEAVEQ

SLWDEPPFSTVLGYFAKQETELGGQLIRKGDGLLLGIAPGNVDPHVRPDLSANMQGNRSH

LAFGGGPHECPGQDVGRAIADVGVDALLMRLPDVQLDCAEDELRWTASISSRHLVELPVR

FAPKEPQDVSQRPSLRPLPQPVPSRPVRTQQPQATTPPPAPPAPPAAAPERGPGAWQRFL

RWWRGY

>CYP105R1(2712582743)SMA

MTQTAAVTTAWPLHRTCPMSQPPALAAFRDGPPRQVLLRGDQPAWLITRYADVRQALADP

RLSVNDQHPNWPNRLLFPVPPRAVSFWRMDPPEHGAYRKMVAAEFTAHRTQALRPLLQSI

TDELLDEMAAMPKPVDFHSVFALPLPCIAIARIFGVPDEDMSEFKENTSALLNQKEPEKA

VQAFLATTAYLDGLARAKEREPKDDLLSRLVVNFVRPGQLSHDDLVAMVRLMLVAGHETT

ANQIALSIFTLLDRPGVLAELRADPRLLTPVVDELLRYWSIAQDNVVRTATEDLSVGDAR

IAAGDAVVISVPGANHDEAVFPDAADFDIHRDNSRHLAFGHGPHFCPGGPLARTELEIAI

TSVFRRFPDLRLAIGRDQVPVHTDTLVYGLECLPVTW

>CYP158A3(2712582803)SMA

MTEKTITEALPPVRHWPALNLTGVEFDPVLSQLMSEGPVSRIQLPNGEGWAWLVTRYDDV

RMVANDPRFSRAAVMGRQVTRLAPHFIPTAGAVGFLDPPDHTRLRRSVAAAFTARGVERV

REKSRRMLDELIDELLRGGPPADLVETVLSPFPIAVVCELMGVPAADRHSMHTWTQLILS

SAHGAEVSEKARNDMGAHFERLIGERRGSTGEDVTSLLGAAVGSGEITLDEAVGLAVLIQ

IGGEAVTNNSGQLFYILLTRPDLAERLRAEPKIRPQGIDELLRYIPHRNAVGLSRIATED

IEIRGVRIREGDTVYVSYLAANRDPDVFPDPERIDLTRSPNPHVSFGFGPHYCVGGMLAR

LESELLVEALLDRVPGLRLAVPPGLVPFKKGALIRGPEALPVTW

>CYP105Q1(2712583161)SMA

MADALAGRAPDATPPVAAYPMPRAVSCPLAPPPALQPLRDEQPITKVRIWNGSTPWLITR

HADQRALLTDPRVSNDDRDPGFPYVNAHRAEIAHATPRLITNTDAPEHTRLRRTVNAPFL

IKRIEAMRPAVQRIVDGLIDDMLDGPNPADLLTALALPVPSLVIAQLLGVPYADHEFFQR

NSNLVLDNSVPAEEARAASGALAAYLDTLLAEKTAAPDADVLSEMGGRIKAGEMTHREAV

HMGVAMLIAGHETTATMISLGTLALLEHPEQLAVLRDAEDPKVIAAAVEELLRYLTIVHS

GIRRVAKEDIEIGDRVIGAGDGLLFDLHAANWDSEAFPEADRLDLSRPARHHQAFGYGPH

QCLGQSLARLELQVVYGTLYRRFPTLRLAAPIDRLAFHHTGTTYGVRCLPVTW

>CYP107L2(2712583555)SMA

MGNVIDLGEYGARFTEDPYPVYAELRERGPVHWVRTPPPEAFEGWLVVGHEEARAALADP

RLSKDGTKKGLTSLDVELMGPYLLVVDPPEHTRLRSLVARAFTMRRVEALRPRIQEITDG

LLDEMLPRGRADLVDSFAYPLPITVICELLGVPDIDRVTFRALSNEIVAPTGGDAELAAY

ERLAAYLDELIDDKRSTAPADDLLGDLIRTRAEDDDRLSGEELRAMAFILLVAGHETTVN

LITNGVHTLLTHPDQLAALRADMTLLDGAVEEVLRFEGPVETATYRYAAESMEIGGTAIA

EGDPVMIGLDAAGRDPARHPDPHVFDIHRAPQGHLAFGHGIHYCLGAPLARLEARVALRS

LLERCPDLALDGPPGARPPGMLIRGVRRLPVRW

>CYP179A1(2712583628)SMA

MAYRPAHLYGTMGPTRIRGRRAPPVDLELHGRLDELQRDPYPHYARARRFTALEHVPELD

AWLVARDADVREVLRRPDDFSSANALRPDVMPAPAALAVLGGGFGGRPVVVTADGARHQE

LRAPIVRGLSPARVAAVLPYAAERATALVDRFLSGAESGRVELMAAYARRLPGDVIGRIV

GLDPADVPAVVYGGHRAEELLFRPLTEDEQVVAAQDVVAMQHLLDAFVRDRRADPREDLA

SEIIASVAPGDGELTLDQRHDIVAHLQNLLLAGHLTTTALIGTTVLNLLRHPRQWELLCA

EPERIPAAIEEAARYDSALQGFRRVTTRPVTLAGTELPAGASVFVAFGGANRDGGRHPRP

DEFDITRTPRRHLAFGFGVHGCPGAQLAREQLRLTLEQLTRRLPGLRPADDRPVTMRPTL

IHRSPRSLHLVW

>CYP180A1(2712583734)SMA

VSVREQAPPVPDVFDPRRYAAGVPHAAYRTLRDHHPVARQAEPEVLGWPAGPGFWAVTRH

ADVVRVLKDSTTYSSRFGATQIRDPDPDDLPFIRDMMLNQDPPDHGRLRRLVSRAFTPRR

VDRFESLARDRARTLLAAALAGARAGDGSCDLVATVTDDYALLNLADLLGVPESDRGLLL

HWTRRVIGYQDPDEAGEPLLDAAGKPVDPRSPGALRDMFEYARQLASYKREHPADDIMTT

LATDPELTGPELAMFFFLLTVAGNDTVRGAAPGGLLALAEHPEAYARLRARPQQLPSAVE

ELLRWHPPVLTFRRTAVRDTDLAGQRIRAGDKVVVFHASANRDGRVFTAPDRLDLSRSPN

PHVSFGDGPHVCLGAHFARLQLRVLYAQTLRALPAPRVAGPPGRLVSNFINGIKSLPLQV

A

>CYP107Y1(2712583947)SMA

MDFGADPYPEYAWLRAEEPVRQVLEGRGLYGLLVTRYEDVRKLLSDPRMSKDPRNAPLDW

QEAGKGRPLEDRTGLGTHLLTTDAPEHTRLRRLVSTAFTARRVEGLRAQVQHITDGLLDT

IVPRGQAELIGDFAFPLAITVICELLGVPKADQDVFRQWTKDFRRWTNTDSAQADRGDAR

PVGLRDLLEYLTRLVDKRRQDPADGLVDALIAARDDDDRLNEAELLSMMSLLLVGGFETT

VNLIGNGTLALLRHPDQLALLRERPELVDSALEEMLRYDGSFETATWRFPLEPIEVAGTR

IEKGHPVLLSLASANRDGAKFPAPDDFDVTRADPAHVAFGRGAHFCLGAPLARLEGRIAF

HGLLRRLPGLALSVPPEQLRWQRSLTVRGLEALPVTFDA

>CYP181A1(2712583955)SMA

MTKLPTHPIRRQLPGVLPAEYETLRERGIGQVELPTGKLVWMVVRPEYARIVLSDPRFSS

DKTDDRFPKLTPNSLMKLRYCAPFMINLDGPEHLKKKKSIMDEFSPEGLARLLPRLRVAV

EEKIDDMLRQPTKPVDLVKELAFPIAWQLQEMFLGIPAAELETMRDNVWKLLLGTTTEAE

EREAADRLNGHAEEVLKEKAKHLGDDMMSRLIVQEREKHGEVNWYELAPLMLSNAQGIHN

SVSTMISLGVLTLLNHPEQRPTLLAHPDRMTTAVDEMLRYFSVNDGTPMRLATEDMLIGD

TLVKAGDGVAVPTLPVNRDPSVCPYPHQLDIMREEPARHLAFGHGPHKCPADRLVPSLLE

IVYTTLFERVPTLALAVPEAELTYKYHSIQAFGPAEMPVTW

>CYP107LG1(2712584311)SMA

VNAEEVNDAVACPMPPGHRDSGTLTDLVDTRGYIDAPYALLAQLRAEKPVRRVVFAGVPV

WLVTRHEDIRTASKDPRLSNDPRRGNRTVRTAPWVFATETYTVTRNMLRCDPPDHTRLRE

LVAGEFTPGRIEALRPRIQQITDELLSPFLPLGRLDVITDFARRLPLTVISEVLGVPAQG

RDTFARLASVYVEMSEGDHGRMPRAASEMRDYLTGLLKAKRQSSARVDDLLGRLLAADHQ

EDEVIGMSFLLLVAGFETTANLIGSGVLALLEHPEQLELLRGKPHLVGQAIEEVLRWNGP

IKMAPVLRFTTCDMGIGDVTVPGGGEAVLLSYGAGNRDPARFAEPDRFDILRDSRGHLAF

GHGIHHCLGAPLGKAEAAIALRALLERCDGLALASDPSELAWGHSRFMRGLTSLPVTFRP

VPRGSTP

>CYP2108A1(2712584313)SMA

MGTIGSTDALTDVHAGDPYPVYEWLRETEPVYRCRTGAYLLTRYPDCRRVLTEREYFLAP

GGPRPSAGAAGRCAPVLSRVMSAQNPPRHTRMRTAVARRFVEHVRRQQPWVSRLCDHLLE

PVVETLRYGIADMTTLAEAFPRDTIMHVLGLPRGDQEHVCALVTDIFPTSGPLPGQDRDG

AGAMRQLTDYLQGEIARRRRAPGDDLMSELVRAHTACPEALPHDELVSILTGLTVAGYPQ

IAAGIETGIVLMIRHPERTVFLDDPVRARMFVDEVLRYDAPVQFTPAPRIPARTVVLGGV

TIPAGAQVWPVLGAANRDPGVFTRPDEFWPGRGEARHLSFGGGAHYCLGAELVYLEMAML

LRRLRQRLPDLALARPPAQRVGRLRGFTSVAVMRTEADRHDR

>CYP105BT3(2712584359)SMA

VELRTSSYLTGMVMSDSVIQQDVSWPLARTCPMAPPPAYGELRRNPPRKVNLPDGGWAWL

VTKYADVRQALMDPRFSSDDTKPGFRGRIQLPPDRNMNSFWRMDEPEHGKQRHMVMTEFT

ARRIKELRPRIQELVDDLLDRLEQLPRPLDLFTEFCLPLPTLVIARLLGVPEGDYLTFSE

QSRLCLALDAPDKALAAYQDMTAYLHRLAEKKERNPADDLISRLITDHVLTGELAREDLV

PLIRLVLIAGYETTTNQIALSVLSLLTEPGLLAALQEAPERIHPFIEESLRFWSVSHDNI

LRLVDADMDFGGVRMSKGEAVIVAIPSANHDPSVYDEPGRFDMDRDVQKHVAFGHGTHLC

PGAPLARREIEMAITSLFARFPDIRLVGATDDLAFRNESLVYGLNELPVTW

>CYP147A3(2712584482)SMA

MTQTSTWQQILDYSHRADPYPLYAELRKTPVIRLEDGSHVVSTYREIVALLHDPRLSSDM

RNVPELAADAPAQAADEGTPTLPPTFIQTDPPDHDRLRRLLTRNFGPPHRPDRIDSMIPH

MSEIVTTEIDSFAGKDRSGTTRVDIVDDFAYPFPVTVICQLLGVPREDEPRFQAMSDAVV

QAADPNTGEFAERQRRRAKAIADLGQYFAQLLDARHGQRGDDLLSGLLSDDGDEAPMSPE

EILSNAALLLVAGHETTVNLITNGMLTLLRHPEVLDRLRRRDEPDLAARVVEELLRYEPP

VQFLSNNRNTLDDIEIAGTTIPKGSPVTLVLASGSRDPDFIPDPERFDPDREHNEHLGFG

GGIHYCFGAPLARPEAQIALTELAHRLQNPRLVADPPPYRASPGLRGPRHLLVEIDGVKP

APG

>CYP2340B2P(2712584497)SMA

MGSPRYVIYASATTPDLVAAKYAPKVHGFLARLFAESNAGKPLMHEYYENYFDLYWDLHL

GVTGEAIPPEVRTIGAGFTAVLGHWYPTEDIVRENIMRVRELRPRLREWIDRRVQAVIDG

DVQDPEGTFVHYWLKNGQGGEHFRREDIVFECFHNFLAFSQWGNMLYRTMALLDEGSGDP

AVRSWFERAMTNGPDAADGGAFTPLDRYVMELFRTVSPNGGSLSTVSTERGADPRSGSVL

TLHPSVS*DPRHWRNPEEFDPDRYRGVPTSADNAETRSRQAGLARCPFPPAPFTVRDGRR

AEMTNSAFGAVYGVVDGTAYPVCDAAGYAPFGFGYRRCGGEQLTTEFVKEFLRTVWSRGI

EFTTLDLERSEKLPVSPRTVIDDNIGFRQGP*

>CYP1038A12(2712584500)SMA

MDTEAGLGSLPYVPGTGRPVPEAEPGLVERWRSGGGELVELLSQVRVRFGGVAAFRLGSA

PTVLVTDPQAVQHVLARHPEQYVKRSHRARVLIGDGVLAATGAAWKRQRRLLQSQFTGTG

MRRYEQRITEAVRTTAGRWDGYARTGQTLDVGQEMRRFALDAIWRSLTGHPLDDGTEREL

AAVAAVAAALPTLPADVTDAQDAVAADLARIDAVARHAIEAARSGEAGPDGPGLLHVLTD

AATERPEYTDRLIRDELVTLLVAGHETTATTLTWLYLLLDRYPAAREEALAAGGEGSAQR

RQAVQALVHETLRLYPSAWILPRHAAEDDTLAGYAVEAGTDILVCPYLTHRDPQLWPEPE

HFDPRRFNTPDGRPTHPGAYFPFGIGPRACLGLQFALRESTVLLEHLLPVHTPSFHSTPT

KAVYGITVRPDGPTPATLVSPLA

>CYP165B11(2712584526)SMA

MPSSTDTAVPVHTRRDRFDPAGELRLMVGGEPVTRIDGGPQAGDVPVWLVTGYAEVRQVL

GDHRRFSTRRRFGARTAPDGPRPDELVGQLMDYDPPEHTRLRQLLTPEFTLRRMRRLEPG

ITALVTEHLDAMERSGPPADLMAAFASPVPGAALCQFIGVPRDDHADFLRRCHAFLAPGR

GRRRRAAAGELLSRYIAALVARRRKDPDDGFLGMLVRDHGDRITDAELRGVCVLLVLAGL

DNVSGMLGLGTLLLLGHPGQLALVRDDPAAVDGAVDELLRYLTVPHAPTPRTALEDVEVG

GRLIRAGEHVVCSLPMANRDGALLAEPDRFDITREPAAHVAFGHGVHHCLGAAMARMELR

IAFPALLRRFPGLRLAVPESEVPFRVHALAHGVDRLPVTW

>CYP165E3(2712584527)SMA

MARPSDLSMHNRRDRLDPLPELGALSARAPLAEADLTDGPTPSMGWLVTGPEEVRAVLGD

TERFSTAPPADGSRPVQPGNLIQYDPPDHTRLRQLLTPEFTVRRMSRLRPAVDAVVADCL

DALEKAGQSADFMRYVAWPLPGLVMSELFGVPRDDRAELARVLKVSRPAFRGRQVQMTAG

AAYLSYVDQLVTRKRRDPGDDLLGRLVRDHGDDISHEELVGLTAFVIGSGVENMASMLGL

GILALLENPAQLAQLRQCPHLIDQAVEELIRYLSIIPTASPRTARADVPLGGRVIKAGDR

VACSLFAANRVRPPGTPPDRLDITREPAAHVGLGHGIHYCIGASLVRMELTSAYLAVLNR

FPELRCAVPPEEIRFRPQAPYGVETLPVAW

>CYP102D2(2712584720)SMA

MTTQPETDLRPIRSPRGVPLFGHTPQIPSTNPVEYFGKLSKQFPEGLYGMEIAGIEQVFV

WDPDLVAEVCDETRFFKQIDKTPLAHVRDYAGAGLFTAHQHEEEWGMAHRVLLPVFSQRA

MKGYFGQMLEIAQNLVGKWERKEGQPVNITDDYTRLTLDTIALSGFGYRFDSFAKEDLHP

FLNALLQALVESLRRSQELPVMTKMRKADDKKYRENIRLMRDLVENVIKERREGKGTGED

DLLGLMLEATDPETGKGLDDDNVRDQVVTFLIAGHETTSGLLSFATYSLMRNPHILAQAY

AEVDRLLPGDTVPDYDTIMQMDVIPRILEETLRLWAPIPMIGKSPLEDTVIGGCYGLKKG

ARVNILEGPLHTHPKAWERPEEFDINRWLPENRVNHHPHAYKPFGNGVRACIGRQFALTE

ARLALALVLQKFKFADTDDYKMDVKEALTRKPGGFELNVRARQEHERTVFGAADLQTDDT

QAQAAVSGVGVNLTVAYGSSLGSCEDLARTIADRGERSGFGTTLVGLDELGDNLPTEGLL

VVVASSYNGKAPDNAQRFDDLLAAGLPEGSLSNVRFALLGAGNTQWVATYQGFPKRIEAG

LLAAGATRVIERGIADAAGDFDGMATRWMDTLWTTLAEEYAADTSETTGPRFEVQLLTEA

EVRPAIVSEQAYPLTVVANEELVSDATGLWDFSIEPPRPAAKSITIELPDGVTYDTGNHL

AVFAKNEPVLVNRALARLGVDRDQVLRLDQPGGGRTHLPVGTPVTTGLLFTEFVELQDVA

TRSQIQELAEHTQCPWTRPQLQAYTADTAEAEERYQKEILGKRVSVLNLLERFPAVELPL

AVFLEMMGPIRPRFYSISSSPLANPRHVRLTVGLLEGPALSGDGRYRGTCSSYIAGLESG

DVFYGYVRVPSPTFAPPADPATPLLLIGPGTGIAPLRGFLEERAHQHAHGTQVGLSQVFV

GCRHPEHDYFYRQEMQDWEQAGIAQVHTAFSAVTGHPARFVQDAIVGAADTVWQAIQDGA

YVYVCGDGRRMAPAVREALAAIYRKHTGSDDEAAQQWLAQLEADERYQQDVFA

>CYP147B1(2712584731)SMA

MASDTLLARITDYANRPDPYPLYAELREAGPVVRQADGSYLIGTYHEIVALLHDPRMSAD

PRSRTAPAPYEVTRKPSFLRLDDPEHHRLRNSAMRPFGPPHSPGRVDSMRGEIVQLTKEL

AEVFQEGRQIDVVDDFAYPLPVTVICRLLGIPDKDEQLFQDWTDTLVASADIGPEGDTAE

RDQAADQAQQEMGQYLVQLAEQRRGRPTGDMLSDLVNEPDPAARLSEEDLAANTILLFIA

GHETTVNLIANGVLTLLRRPDQLDRLREDPSLLPRAVEELLRYEPPVHMRERVPLVDIDV

AGTTIPGGTSVILALASGSRDPMRFSEPDRFDPTRPDNQHVGFGSGIHLCFGAPLARIEA

EAALGALLPHLGTARLVQDPPPYRQNAMLRGPRHLPIQL

>CYP178A1(2712584996)SMA

MTAMVIDLADLDLFTDGDPHEAWRVLRRDRPVHWNATGGDTASGVGPSGFWALTRYQDVH

DAYVDAGLFSSRWGTVMGGSHRRDADSASGRMLIASDDPQHRLMRQQVHRAFLPALMDRA

RRVVRDYVNAALDRVLKDGGGDFATDVAPELPAGLLAAMFGLERRDALHLLALTRSMIGF

QDPRYHSDSTPPAVLVSSQVEIFDVMMDLMETRRRAPSDDLVSILLGASINGRRFTEDEV

LYNCLNVAVGGNETTPFTASAAVQAFMDFPDQAQRLLDDGGLLPTAVEEIFRWTSTNAYV

QRVATRDVELHGQLIRAGDPVTLWNASANRDEEKFPNPDRFDVRRTPNRHIAFGVGVHRC

IGMGAAQMEIALLLKEIVRRGITFEPAGPPARLRSNFMLGLTCLPVHATVAQDAS

>CYP178A3P(2712585028)SMA

MSATAIDLDDLDLFVSGDPHAAWARLRREAPLHWNAGPDGTGHGALTRYADVETIRAGDT

VTLWNAPANRDPDAFAEPDRLDLGRTPDQHLAFGVAHHRCVGMGAARREIALLTEEIVRR

GLRCEIPGPVRRPRSNFMPGITHLPVTVTDVW

>CYP107X1(2712585345)SMA

MDPAEGLLADPYAVYDRLRDTAPVHRIAGTDGKPAWLVTRYDDVREGLANPLLSLDKKHA

LPGNYRGLALPPALDANLLNMDAPDHTRIRRLVGRAFTLRRVEQLREPVRETAHRLLDAL

GTHGSTDLIASYAAPLPITVICDLLGVPDEHRRDFRAWTDPLVTPDPARPDVARESVVSL

LGFFTGLLADKRKNPADDLLSDLIAVQEEGDRLTEDELMSLAFLILFAGYENTVHLIGNA

VLALLRHPEQLAALREDPARLPDAVGEFARYEGPALLAIRRFPVRDVTIGGVTVPAGETV

LLSLSAANRDPSRFPDPDRLDLGRDAAGHLALGHGVHYCLGAPLARLETEVALAALLERF

PDLALAETEPRRRPSLRARGLLALPVTY

>CYP125A2(2712585756)SMA

MPCPALPDGFDLTDPDLLHQRVPLPEFAELRRTAPVHWVPQQHGLAGFQDDGYWAVTRHA

DVKYVSTHPELFSSYLNTAIIRFHETMQREQIDAQRLFMLNMDPPEHTRVRQIVQRGFTP

RAIRALEDTLRRRARSIVETALADAGPDGSFDFVTQVACELPLQAIAELIGIPQDDRAKI

FDWSNKMIAYDDPEYAITEEVGAESATELIAYAMNMAADRKQCPAKDIVSTLVAAEDVGN

LASDEFGFFVLMLAVAGNETTRNAITHGMHAFLTHPDQWDLYKRERPATAAEEIVRWATP

VVSFQRTATQDTELGGKQIRKGDRVGIFYSSANHDPEVFEHPDVFDITRDPNPHLGFGGG

GPHFCLGKSLAVVEIDLIFGAIADVMPGLRLTGDPRRLRSAWLNGVKGLQVSAG

>CYP154C2(2712585921)SMA

MTTRIALDPFVSDLEAESAALRAAGPLAAVELPGGVPVWAVTHHAEAKKLLTDPRLVKDI

NVWGAWQRGEIAPDWPLIGLANPGRSMLTVDGADHRRMRTLVAQALTPRRVEQMRERITK

LTEELLDRLTGEVVDLKADFAYPLPMYVVADLMGIDEARLPRLGELFEKFFSTQTPPAEV

IATLTELAGIMAETVAAKRAAPGDDLTSALILASEDGDHLTDAEIVSTLQLMVAAGHETT

ISLIVNAVVNLSTHPEQRALVLSGEADWSSVVEETLRYSTPTSHVLIRFATEDVPVGDKV

LPAGDALIVSYGALGRDEAAHGPTAGEFDITRSTENRHISFGHGPHVCPGAALSRLEAGV

ALPALYARFPKLDLAVPAAELRNKPVVTQNDLFELPVRLG

>CYP157A2(2712585922)SMA

VTPAPAPAPVPLSGPRFQTDPAALYREMRREHGSVAPVLLDGDIPAWLVLGYRELHQVTG

DPVLFSRDSELWNQWPNIPADWPLLPMIGHKQPSILYTVGERHRERAAMISDALEAVDPF

ELRSLTEKFADELIDAVCAKGETDLVADYAALLPVRVLALLYGFAEEQGPGLVTALNDMI

DGRDRAIAGQTHLATSMAQLLADRKAEPANDVVSRMLADSGGFTDEEIAQDLMVMMAAGH

QPTADWIGNSLRLMLTDERFAASLFGGRNSVAEAMNEVLWEDTPTQNVAGRWASRDTQLG

GRRVRAGDLLLLGLQGANNDPQVRIDGAALTGGNNAHFSFGHGEHRCPFPAQEIAEVIAR

TGIEVVLDRLPDIDLALPAGSLARRPSPWLRGLTELPVKFTPIPALGGSPA

>CYP154B2(2712586113)SMA

MDMTDMAETAEAPGTAEAPGTAEAPGTAEAPETAEVSGTAEAPRTAETAEAPETNGTNRV

AEGVGAAGVTGMNQCPYALDVTGRDLAGETAQLRTRGPAVEVELPGGVAAWAVVRQKYVK

QLLMDARVSKDARQHWPAFVSGQIDEAWPLYPWVANENMLFAHGDRHARLRRLNAAAFTA

RRTEALRPRVEEITAALLDGLADRPAGEQVDLRAEFAKLLPMRVICELFGIAEANREPLC

TALELVFGTAVPADEMAAAQVKVFGMLAELVAEKRERPGGDLTSALIEARDGDDSRLTEQ

ELLGTLYLMIAAGQETTCTLITNAVAALCAHPDQLSHVREGRADWADVVGETLRTHGPAA

YSPMRFAIDDIELDGVHIKKGDPILVSFAAAAADPEPYGADAAVFDVLRPGRRDDLAFGY

GVHRCLGAPLARLEATTALSALFTRFPDLTSVQPTEELEPVRSFIVNGYGSLPVVLRPH

>CYP107U2(2712586286)SMA

VAGSARRRGRHGGGVGTVAGSAAGTRRLQRERGSRYRHPVNAPHSEPELFTWEFATDPYP

AYAWLREHAPVHRTTLPSGVEAWLVTRYADAKQALADARLSKNPVHHSEDAPGKSKTGIP

GERSANLMTHLLNIDPPDHTRLRRLVSKAFTPRRVAEFAPRVQELTDHLIDQFAQTGSAD

LIHEFAFPLPIYAICDLLGVPREDQDDFRDWAGMMIRHGGGPRGGVARSVKKMRGYLAEL

IHRKREALPADPGPGEDLISGLIRASDHGEHLTENEAAAMCFVLLFAGFETTINLIGNGT

YALLRNPQQRARLQQSIERGEQDLLDTGIEELLRYDGPVELATWRYATEPLDMGGQRIAS

GDPVLVVLAAADRDPARFDEPDTLDLSRSDNQHLGYGHGIHYCIGAPLARLEGRTALATL

LRRLPDLRLAADPADLRWRGGLIMRGLRNLPVEFTAT

>CYP107V1(2712586304)SMA

MSGASARCPFSGAGGGTDVLPLPADPGTAAGAPAPGPPDAPAAPAPRTADRSAPHAADPH

APSTADLPRFPFPGLSGIPLDPVLLDRYRGEPLVPVGLANGREALLVTRYADVRTVLSDD

RFSREAWANGTLFARRSGALALVTSDAPTHTRRRSRVQSRFTHRRAEEDRPRIARIAAEL

LDSLQAADTGRPVDLIAEFTTPFPYRVICEMLGVPVADLDRLLPSVTVMMSAGRFSADEA

ARAHEVMYGYFFGQLAARREAIAAGCPGDDLLTSLLSAPRETRLSDEEIVVFGFGLMMAG

GETTASHLAMCVLQVLGTPGLADRLRRDASAVPAVVEELLRWVWFAGTGGQPHVALEDVE

LAGRVLPAGQVVIPLTDAANRDPGVFPDADEFRPDRAPNPHIGFGHGRHMCLGAAHARVE

LQEGLTAVLERLDHLELAVEPAELRWRDQMFMRGVWELPVRWHTKEDGRC

>CYP170A2(2712586803)SMA

MTLESVKPEAPEAPQLRNPPLAGGGIPGLGHGWKLARDPLGFLAQLRDHGDIVRLKLGPK

TMYAVTTPALTGELALSPDYEIGGPLWESLEGLLGKHGVATANGPTHRRQRRTIQPAFRL

DIIPEYGPIMEEEARAFAARWQPGEIIDCTSESFRVAVRIAARCLLRGHYMDERAERLSI

ALTTVFRGMYRRMVIPAGPLYRLPLPANRKFDRALADLHLLVDEIVAERRASGQKPDDLL

TALLEAKDDNGDPITEQEIHDQVVAILTPGSETIASTIMWLLQVLTEHPEHAAKVCAEVE

SVTGDRTVAFDDVRKLSHTNNVVVEAMRLRPAVWILTRRAVTETELGGYRIPAGADIVYS

PYAIQRDPRSYAEHLEFDPDRWLPERSKDLPKYAMRPFSLGNRKCPSDHFSMAQLTLITA

TVAAKWRFEQVSESSDATRVGITLRPHRLLLKAIPR

>CYP183A1(2712586836)SMA

MSQHTFVAGTAPGAVPVVGHAWQMMRRPLHFMSSLSAHGDLVKIRIGPTSAYVPCHPELL

RQVLTNDRVFDKGGVFYDRARDIAGNGLVTCPYRDHRRQRRLMQSAFQRTQLERYSTAMR

AEIDATAARWHDGTVIDAFPELYGMALRTVARTLYSTPVTEELAQRVEQAFDTVLNGLFR

QMFLPHSLRRLPTPANLRYRNNLRFLHDTVQDLITEYRRDDTQRDDLLSALLASRDEDGG

RLGDTEIHDQVITVMAAGTETVAGTLTWIFHLLSRHPEIEARLYEEIDTVLDGKPPHWDD

LPSLSLTDRIITEALRMYPPAWIFTRLTASDVDLAGVRLPEGTTIVFSPSSVQRHSEAYD

DASRFDPDRWLPDRTSAVARQAFTAFGTGARKCIGDLFARTEATLALATMLSQWRVTVEP

DADVRPVALATVYHPRRLRLRLTARTPGQ

>CYP105B23(2712586946)SMA

VEVGGQTIRAGEGVLCTLSTANRDEGAFTDPDELDLRRDARSHLSFGFGIHQCIGQSLAR

AEPQIALETLLRRLPGLRLAASFEELRFRDGVGFYGVKELPDPCPALPPDRP

>CYP183C1(646969234)SBH

MTDNSRHQPEAKPPVGAKPPVGAQPPGEAQPPAGAKPPAGAKPPVGDFSAASARGAVPLL

GHAAQLLFKPLDFFASLPSQGDLVEIRMGPWPVHVVCHPELVHRLLASDRCFDKGGPLYD

RLRGLIGDGLASCPHAVHRRRRRLVQPAFHHTRLPLYAKVMTQQIDETTRSWADGQILDL

REELTGLATRVLSRTIFTADFSGPAIAALIPYAELITAKLSWRLLWDAALKGVPTPGKRH

FARRTEELRSAVYTMARRYRASDTDHGDLMSMLLSARDDDGSGLTDAEIFAEVVTMVLAG

TGTVPNTLSWAFHTLAQHPDIAKRLHSEVDKVLGGRSATWDDLPALPLTAAIFTEAMRHF

PSDWLVTRVTTEDTELAGRRLAAGSVVAYSGQVIHRRPDLYPQPDLFLPDRWLDTDRKPP

RGAFLSFGGGARKCIGDKFGTIEGVLALSTIAARWRLEPAHPGRARSTSSSRRSTRPPAM

RLVARG

>CYP183D1(646969235)SBH

MTMPNTFTTGLAPGALPLLGHARQLRRQPLPFLESLPGVGDLAEIRLGRERVYVPCHPEL

VRQVLTDDHTFDRGGALQDHFVELFGECVATATYRGHRRQRRLIQPAFCHDRLEGYAPVV

EAEVAALPDSWREGQTFDLFPVLYTLTLRSVIRMLFSDRVDEATMDEVRRLFLAMTGGAA

PRQGVARLIRGTGPTEGRRRRAAHQLRGVVDRIVADYRRAGETGKDMLSALLTAREENGG

RLDDTEVRNQAIIMLAAGSHSPATVLTSVFYLLAEHGGAEHRLHREVDAVLGGRPARWTD

LPDLAFTGRVITETLRLYAPAWTIFRKTTKEVRLAGRDLPAGTTVLVVPLIPHRRGDLFD

CPREFTPDRWRPECDTVTQRGAFKAFGGGARKCVGDVLGMAELKLVVATIASRWRLECLP

DARVRPHAGAVAPPPRYLPVRLVERRP

>CYP1038A1(646969286)SBH

MVDLLAQARELGGVSAVRLGPRPTVLVTDPQAVQHVLALHPDRYVKRSHRARVLVGDGVL

SAVGEPWKRQRRLLQAQFTGAGIRRYEQRIVGAARRAADHWAECARTGTPTDVGDDMRFF

ALDTIWRSLTGHPLDETTNRELAAVEAVGAALPATPSASADVAELRAAVAADLARIDAVA

EHAIAAARRGEAGPEGPGLLRVLVEAGQTHAEYTDQLIRDELVTLLAAGHETTATTLSWL

YLLLDRHPEARRWALNAGPAGSPEREKAFRALASETLRLYPPAWLIPRHATEDDALAGYH

IAASTDIYVCPYLTHRDPALWPEPERFDPERFTTADEGRPSSRHGAYSPFGLGARACLGA

QFAMREMTMLLEHLLPAFTPSFHTTPPSAVFGLTVRPDGPTPATITPTPNGS

>CYP183E1(646969308)SBH

MDSGSAVVQAPGAIPVVGHLTAFLRAPLPFLRGLPAHGPLVTIRLGRQRVIVVCDPELTR

TVLVQDRVFDKGGPLYERLREAGGQGLASCPSGEHRRQRRLIQPAFHHSRFPGYAHVMAE

RMDTLLGGWHDGQTLDLTTETRRIAADVVISAIFGTGLDAAVHTRLATDFHTLLAGFTRH

TLTPKPLRKVPTPGNLRYERATRRVRRTMADLTTTYRAHGDTGETNLLSLLISARDPESD

SPALTDDELVDQAVTMYSAGTETTASAVCWALFLAALHPEVHDRLITEIDTVLDGRTADW

SDLPRLIYTRQVFTEAIRMYPPGWFLTRRTESDTRLGSYDLPKGTTIAYSPYLISHLPGL

YPDPETFDPDRWKPAERGPEVPVTVFGGGARKCIGDQFALVEGVLLLASLVSHWHIDFHP

DSLSLPRLPQLTLNPGKMKATVTARRGGEDRQRHTSQSRVP

>CYP143C1(646969335)SBH

MAERRDDAWRMLMARPEPAPMDNGLAVTSLAAVKAVLKEPNRFSAKKAFDAVETGYPLIP

LAFDPPEQTHYRRILQPFFGPRRIRALEESLRAQAIDLVEAVKARGGCDFVADIAVPFPA

QALLTLLGLPLADRDRFIESKNAALELTADAAGELTLTDEERAARVEQTMALGNYLGELI

QTRRAQPGDDILSEILAIDGDDHLSDTEAMGVCLMLVLAGLETVTDALSLAMERLATHPG

RRRELVEDPSLVPAAVEELLRLDPPAPFLPRVTTEDVEIAGCPVPAGTLVNTHLTTANRD

ETCWPRPHDIDFHRPDNPHTSFGVGVHRCLGTHLARLEMQLLFEEWHRRIPEYAIAEGTS

PRARLVRANIGMESLRLTIPTA

>CYP1031A2(646969336)SBH

MSTPPQSEPVIFNPFTPEFMEDPYPHYAELRRHVPVHEHPGGFWMLSRYEDVDALMRSGL

SVEQRHVAPGPFRDAYTNAGVTDEPRLKGLALLDRDPSDHTRLRKLVSKAFTPRAINAME

PRIRSLVDDALDAIAEAGTVDLVEALAFPLPFTVISQMLGMPPTDNDRLRLLSHTLMRSV

EPTTDIEVMRAVEAADAELFALVGEAVSWKRDHPADDLLTALIAAEDNGDALTHDELVAQ

VAMLYVAGHETTVNLLSGGTLALLRNPDQLKLLRDTPDLIENAVEELLRYDAPVHNSRRV

TLETYHVDGFEIPPGSFILANLAGANRDETFWGPDAEELRLERENARRHLAFGGGIHHCL

GAALARIEGRVAIGELVRRFPALSLDGDVEWNGLLSLRGAARLPVRV

>CYP147F2(646969465)SBH

MTLGSMSARINDYANRADPYPLFAELRKQPVIREDDGTYLVSTYYEVKSLANDPRLSNDT

RNRAPGCARTGGSEENTGLPPSFIFTDPPEHDRLRGTANRPFGPPHSPRFLHDLQADLAK

VVTELLDAFEGKDQVDIVEDFSYPLPVTAICKVLGVPREDEPRFHGWADALAAGVDPHPG

ENGQERADTARQEMGSYLADLIDASRRHPRPGILSALAPHTGPGGRMTPKDLVATAVLLL

VAGHETTVNLITNTTLTLLRHPDVLRRFQHDQDLATPLIEEVLRYEPPVQFVPWTTALAD

IDIADTTIPKGSPVWLMLAAANRDPRRFEDPDRFMPERKDNEHLGFYTGIHYCFGAPLAR

IEAHLALPELFRRVKDFRLLEDPPPYRANAVLRGPRHLPVAIEGVTA

>CYP105B4(646969508)SBH

MTGAAANTTSGTPLDTPEYNIDRSARCPLDPAPAMRARQAEGPLVRVRLWDGTLAWLVTG

WEEHRALMSDQRVSVDPFRPGAPKLSPGEVTAIDVLKKEGKRSTGTSFILMDDPEHARLR

RMVTSAFTIKRVEALRPPTQRITDDLIDTMLAGPKPVDLVEALALPVPSLVISNLLGVPY

DDHEFFQANSRTIINRETTAAERAAARGRLVDYLDGLLGEKLAQPREDLLSGLAERIKTG

ELAREDATEMGVLMLFAGHETTANMITLGTLALLQHPDQLALLRDTEDPKLIASAVDELL

RYLTITHGGQRRVALADIEIAGQVIRAGEGVIPVNEIANRDPSVFPDPDRLDLRRDARRH

VAFGFGIHQCLGQPLARMELQVVYPTLLRRIPTLALAADLAEIPFKHDGFVYGAYRLPVT

W

>CYP107AM2(646969510)SBH

MSQNDPTVRLPLPTDGPLDPPPEWELLRGRCPVATVELPSGDTGTLLTRYDHIKALLSDP

RFSRPAPEDDSARIAPEGAGGVSTNSGMSLSLQSNGEGHQRWRRHVGKYFTARRVTALRP

GMEEMAETLIDAMVQSGSPADLKAALGFPLPVYVICNLLGAPAEDRDRFSYWSDAFLNVS

RYSVAETGAAHEDFVTYMSELIAAKRAEPSEDLISTLIEESRAEGEGLTDLELRDTGMAL

LVAGHETTANMIGKMVAMLLADRTRWDSLLADPSLIRTAVDEVLRSDANLGGYGVRRYLT

EDYEVDGAVLPGGTTVFCGLSAANRDERVFADPDEMDLTRSPNPHLTFGAGAHSCLGQSL

ARTELQVVLEVLLHKLPSLDLAVPVARLRQIEGLTVGGLREVPVRW

>CYP1039A1(646969611)SBH

MMGMGIRGARTAIKLATFRTMMAGAALTGDPAAKLLGRRQPANPFPLYEQIREHGDVYRS

RLGIFCTVSHAQCRAVLKDPRFGMPVPPSPPAWEMYQGDADTLIHPIERSLLAVNPPEHT

RLRRLVAPWFTASALRTRAARVEKIVHSHLDRISDGQDFDLVRDFTAQVPTAVIGDMLGI

EIENYEQFGRWGMALATTIDGVRTMGERRMVRAVLAEMTSFFTQLIEENRRAPRDNAISG

LVGAEVDGRPVTDEELIGLIGLLLAAGLETTVNLISNSVRFLLEHPDQKRLFLDNPDTAP

DVVEEALRYDPPAMFTVRMALEDVELDGRLIPRGGWLVQLLGGANRDPKVFTDPHRFDVT

RGNIREHIAFSAGAHHCVGSGLARLEAEIALRELFARFPDMRIAGDVTMRTARSARGPQS

IPISVAQAVGPDPSRRA

>CYP105H6(646969739)SBH

MATADAIPIEFPRRQPGRLTPPAEYAGLRSQEGLVKSSLPGGSTVWLVTRHEDVRSVLTD

PRISSNPLHEGFPSMGKNGTVPPPDQVPGWFVAYDPPEHGRFRKALIPEFTVRRIKEMQP

AIQRIVDRLIDALLDGGNSADLVAQFALPVPSLVICELLGVPYSDHEYFESRTRVLVTFT

STDEQRESAAKELITYLTKLISIKHKFPGNDLISGLLKNETLTAQEVSGIALLLLIAGHE

TTANNIALGVVMLLLNREWIGDPRAVEEALRYLSVADRVALRVAVEDVEIGGQLIKAGEG

IVPLGAAANHDENAFERADQFDPGRPARHHVAFGYGVHQCLGQHLVRAEMGTAYRTLFER

IPTLRLAVPPEELPFKDEGILFGLHELPVTW

>CYP107BK1(646969792)SBH

MLMLDGPAHRRIRGAISKVFTRASVERLRPRIAAETARLLDAAGEGKTDLIHSLALPLPV

TVTCELLGLPGSDRDQCRRWTEQISRVIDPSITAEDAVEMNAAEVEFREYVADHLKERRS

APRDDILSLLLHAEVDGGQLTEEEIIANVQFLFVAGHETTVNLIGNGLLALLRHPDQLRM

LRDNPEIITDSIDEITRYDPPVQIVSRLLTEDVPLTDVTLPAGAKVMLLFGAAGRDPERY

PDPDRLDLTRTGVKTLAFSGGPHYCIGAALGKLETSMMLTELLRRYSTIELTGDDLVWRP

NVSFRGLQELPLQLVR

>CYP171A2(646969814)SBH

MPAPLQVPDVPGSWPVVGHLPQLARRPLDFLSSLADHGDLVRIRLGRKPVYVATHPDLVR

SLLVTDAHAYTRGAGHAKALAFIGPILVATTGEPHRRQRRMMQPCFHRQRLGSYVSAMCS

AATETADSWSAQDVVDVVPVMTELATAMIAKSLFVSERAAHAEAELRKTGNAILTVARMS

AILPGIYRRLPTPGNRQLPPARTVIEETIAAYRAEGQDHGDMLSTLLRTTDATGTGLTDE

EIRDEVMGLAITGIGGPAAIASWIFYELGQNPDLERRLHEELDTVLDGRPPSSQDLTRLV

FTQRLVKEALRKYPGWVGARRTRESVRLGGHEIPVDAEVMYSAYALQNDPRWYPDPERFD

PDRWDPQQNATRVKKGAWVPFSGGVYKCIGDAFTETETAVAVAVIASRWRLRPADGRSVR

ASHLATHVVPRPLRMVVEPRSRNKDEAERSPHEAPQVSR

>CYP107BM3(646969896)SBH

MTQQPIVLPYGDPAFVADPFPFYRMLCQGQPGEDGPVRRAVIAGGLEVWLVTPFEECLAA

LSDPRLSSDVRLATDPRLMRQLPVAERESVLGNMLRADPPDHTRLRRLVSRAFTARRVAE

LRPHVQEIADRLLDAVVPTGRADLIEDFALPLPVTVISELLGVPTDDRRAFQRWVDHLLP

WGAEPQDPAVVERAWRQMRAYLTGLLATKRVRPGEDLLSALIEARDEQRRLTEDELVSMA

FLLLAAGYITTVNLIGGGIAALLAHPDQLRTLRDDPALLPDAIEELLRYDGPVNPGVARF

ASEDVSIAGVDIPRGATVLVGSALADRDPERFPDPDRLDISRGDSAHLAFGHGIHYCLGA

SLARMEGEVAIGTVLRRLPQLALSVAPGELRWRPTGLRGPERLPVTFTPGASLAAVPS

>CYP157B13(646969912)SBH

MTNVHHPSPVDGPSPLTGCPAHPGAVRMHTEELQADPAELYRRLRRDHGAVAPILLDGDI

PAWLVLGYRELHRVTSDPTLFARDSRRWHAWEYIPADWPLMPFVGYRPTMLFAEGAEHER

RAEAMFDALASVDQFELRAESERVADELIDVFAGSGRAELVADYASQVPIRVVTRLFGLA

PDTAVAIQRDTVEMVTANDGSVAAYQRVHNRMVTLMAEVRRNPGMPGIPAQMAAHRAALT

DEEIVNDLIGTVYASHQSTTDWIGNALGLMLTDERFAVTMAGGRRSIGQALNEVLWENTP

VPAFIGRWAAEDTILGGQRIKKGDCLVLGLAAANADPSVRPDFRADASGNQAHMSFSHGE

HSCPVPARELAEVISMTAIEVLLDRLPDVVLAVSPEELVWRSSIWLRGIAALPVEFTPAL

G

>CYP113G1(646970466)SBH

MSEQAISLTPPSAADGGATLFGWLRRMREESPVVQDNNGSWYVFRHADVQHVLTEYADFS

SDPTPISPKAAEIAKGDLAQIDPPEHHGLRRQISRVFTPRMVAELEPRIEEIGRLLLDEA

GDAGELDLVRQLTHPLPVIVIAEMLGVPAEDRPVFRHWADRIIAAKIAGIDDPETYERLR

SAIEEMDFYLEGQLDERRSRPGDDLLSKMVTAEVDGDPISGAEAVNFARLLLVAGHITTT

MMLSSSILCFDEYPEAAREVREDRSLLPAAIEEVMRYRPPFTITGRYTTRDVELSGVTIP

ERSVVIPWLASANRDERRFMDPDVFDIHRSPNPHVAFGKGIHFCIGAPLARLEGKVALNL

LFDRYSDVRIDRDASLEFYEHNFFGVKSLPVSVKHA

>CYP180A3(646970832)SBH

MVFLPETYAAGVPYALFAEARATRPVCWIEESAVGAWPAGPGFWAVFRHADVKHVLRTPE

VFSSHLGATQIRDPDTPGDLEFVRAMMLNQDPPDHSRTRRVVAAAFTPRAVRELQAVIEA

RARTLVEGVAPRGEADFVELAADLPVWTLAHVMGVPESDRGLLFDWASRVIGYQDAEYAA

SSTADAAGLSPMGRAALAHRPRSLTRPDGRPMNPRSREALADMFAYAHALAERPRPGTVM

AHLREGGLSREEFENMFFLFAVAGNETLRNGIPGGLLTLLDHPESHELLLARPELTDSAV

EEMLRFWPPVIDFRRTATREVELGGQRIRRGDKVVVYHASANRDEAVFNDPDRFDITRSL

GDHVSFGHGPHFCLGAHLARLQMRAMLRGALTRLPGLRRAGEPVRLMSNFQNGLKHLPVS

WETDR

>CYP107X9(646970862)SBH

MQNTANTAVPGPPEPFDTRVLLEDPHTAYAELREAGPVHRIPGPDGRPAWLVTRYDDVRR

ALADPRLSLDKSNALPGNFRGFALPPALDANLLNMDPPDHTRVRRLVTKAFTPGRVERMR

EPVRRAADELLDAVEPAGRADLIAAYAGPLPITVICDLLGVPQRDRRDFRAWSDALIAPD

PERPHVAKEAVGNMLRFYTGLIASKREEPGDDLLSDLIAVRDDAVGGAGEGGDRLTEDEL

TSLAFLLLMAGYENVTHLIGNAVLSLLDHPPLLRELRERLPPAGIAAAVEEFARHDGPAP

LAIRRFPLEDVEIGGVTVPAGETVLLSLASANRDPGHFKEPDRLDPDFGRLGHLAFGHGI

HYCLGAPLARLEAETALAALLSRLPRLRLDISHTELRRRPTIRARGLISLPVAW

>CYP155A4(646970938)SBH

MGYGARKIERAADSDGRSGCPVRRGPDGVWQVCGYAEARAVLRSTDTVQAGLGIETVEKL

PSGLRRPVLYRDGPEHREHRRQTARFFTPRRVDERYREVMVRVAEAQLGKVRAAGRAHLA

DLSFHLAIEVAASVIGLTESRPGIRRRLERFFPEKFGTPGLTSLHGIHWLLRQNVNWLRV

YLADVRPAVRARRGQRRDDLISHLLDEGCSAGEILGECLTFAAAGMVTTREFINVAAWHL

FTDDALLGRYRAADEPGRIAVLHEILRLEPVIGRLRRRTTAALELPGDDGVVTVPTGALV

DVDLESANVDARTVGERPELIRPGRPLAEGAGAVGLAFGAGPHKCPGAHIAILETDIFLS

RLFALPGIRMATPPGVGFKDEIGGYELRGCTVECRA

>CYP154P1(646970967)SBH

MDAISPVHLAPEPTDLHSVEGAALRAAGPVATVALPGGVVVKAVTTREAGAQVLNDPRFS

KSLRHWGAHQRGEIPAEWPLLFILEGEGLLNSDGDLHRRLRQPVQRAFSPRRVRELRPRI

EAVTDRLLAALDDVAPGQETDLQSRFALPLPLDVICYLLGVPEEGGLREELHVLSAAALS

NDAGAEELQRTMAERFPATLMSIIERKRASGDHDDLTMDLVEAMDQGELTVPEVIGNLVV

TVIGGHETTVTLICHAVRGLLTHPETLAAARARQEAGEDPWPDVVEEALRWESPVRALLF

RYATEDVPVVGGGVVREGEAVLLPLATINRCPHAFSEPDRFDPDRPDASRHLSFGYGAHR

CPGASLAKVEAEIALRRLFETFPDIALAQRPAPRAASLGMNSYRELPVVLRPATV

>CYP253B2(646971707)SBH

MTRQAPGPKGEPVLGNARAFQADILQALQRGRREYGDVVRFEGIGPLFPVFFVGHPDGIK

EILQDKHRNFPKTPFVSDRWRALVGDGLICSEGELWKRQRRLCQPAFHRRLITSFATGMT

EVTAELLDRWEAAARAHREVDVTLDMTRLALSVLGGALFGANWRQDSEVMAHAVEVAIGE

AYKKFGKFVSLPEGVPTPANLRFARARKQLDRIIYRVIEDRRADRGPHPDDLLEALMTAT

EDDGSGMTVEQVRNEVMTFMFGGHETVASGLTWALYLLSRHPEVAARLEAEVDEVLGGRL

PGVEDLPRLPYVDRVVRESLRLYPPVSLISRTPLEDDTVQGYDIPKGSMVLLSSFVTHRH

PDFWPNPEGFDPDRWIPLGEQGPHRYAWWPFSGGPRKCIGDVFGLQEMKLVLAMMAQRVR

VRLAPGHPVIPRPGITLGQEHGVIATVALREKAPPAAPRALAGADSEAIAAAAAAAGCPV

HGGGAR

>CYP253A2(646971708)SBH

MTTVFVPPGPRGHPVLGSIREIQRDNVSAFMHAFRHHGDIVNFRGPLRINLLAHPDHVQH

VLRDQHKHYPRPRKVQGCLSTIVGDGLVAAEGGSWLRSRRLTQPAFHRDILRRFGETFTR

TTSGMLDDWERRRGQGRPLDIKSEMMHLSLANLARALFKSDLTDAIARIEPAVQGALSFT

HRRMTSPVDPLRIPSKARGRFRDALGTINSVLYPMIVARRREGGEDDLVSMLIDAKDPGS

GEAFTDEQIRDEVSGFFVAGHETVSTALTWTWYLLSLNPESRRRVQDEVDRTLSGRVPTV

DDLPKLAYTTMVLQEAMRLYPPIFVYMRCAARDDEIGGYRVPAGRWVVVCPYVTHRHPEF

WDNPEGFEPERFTTENSEGRHRMAYLPFGAGPRKCIGDSFAMLQMPLVVAMVAQRFRLDL

VEGQRVFPEPAISLRPRDPMWMWLRPVEEGSR

>CYP157G1(646971841)SBH

MTNPQDPQDLQDPQPPRSAPDTPPPGCPAHAARLYGPDFRREPAETYRRIRREGQVTRVL

LEGDVPVWFVVGYRETRQVLSDVETYGRDPRRWNGWDSVPPDWSLMPWVQYSPMMPFTEG

EEHLQRAAAVSEVLATLDPFVLRGHCERFADQLIDKFAVTGRADLVYDYIYSVPTLVMGE

VFGLAEDEAGLAALAEGLTTSFVSREDAMAGQQRAAAYVAGLVKAKREAPGPDLTSRFIQ

STPNLTEEQHIADVMMLMAAALPLTSYWIGNTLRLLLTDERFATTLTGNRRSIGQAMNEV

LWVDTPLQNLIGRYATRDTVLGGQRIRTGDLVVLGLAAANADPLLWPDGHVGHAGNHSHV

AFSGGEYGCPVGGPETARIIAETTIEVLLDRVPDLALAVAPEELRWMDSVWYRCLESLPV

AFTPTGVMGG

>CYP156C5(646972347)SBH

MTYHPDATAAVGHSPDAEAGVGVAPPPGCPAHAGHAGFGGPARPAAQGLPLYGPEFAADP

HLFYERLRQYGPIAPVELSPGVEAYLITSYSLALEVLRDTERFAKDPRGWRALNEGRIPP

DCPVGPMMMYRPNALFNDGEAHARLRGAITDSLTRLDPYALSEYVERSADTLINAFADRG

EADLLADYAAAIPLLVFNELFGYPAEHGLRLVGTMARLFDSGEDAAEANQELLEYMAGLI

AAKRERPAADITSWMLAHPSRLADDELLQQLMLILAAGTEPQLNLIANALRLLLSDDRFA

GELSGGSLPVQDAIDEVLWTDPPLANFGTRFARHDLDLAGLRIRQGEPILTSYAAANTDP

ELISDGRFGNRAHLAWSIGAHRCPAESPARVIASVAIEKLLDRLPDMELAVPVDQLAWRT

GPFHRALTALPVRFPVTARPTAAAAPAAVSAAVSPPAPAAAPAKPHGSPQTAPDASDTPS

QSPRAAQRRRWGSLAAWWRGQ

>CYP1813A2(646973602)SBH

MTHRTHTTHEFNRFADVRAALADPALVPELPATDGASAGGPAGASVAWLRATVARFSSGE

PHRRRRALVEAELARLEPASLWRAVATGPRGEVRVRVVRALAEALGLPEPGAVAEAVTVV

AGAYFGGTDAAADEAVARLVARLASASADEAALEAVANRIGLLVQACDATAALVESCAGA

ALAEAAGGDVPPARVLREDPPVRTMRRIAARATRVAGREIAEGDVVRLDLATAQRAHPVP

LTFGAPPRVCPGRAHALALADGLLQRPLTAFARLHHQAATPLLLPNAWDYASAAALAARG

FPAVGTTSLGVAAATGLPDGAAATVDATLALARSLGRGSFLFTVDAEGGFSDDVAEVAAL

AHELYDAGAAGINLEDGRADGTLAPVELHAAKIAAVKAAVPALFVNARTDTHWLGRQEDE

TAARLASYEQAGADGVFVPGLSDPDGIASLTAALLVPLNILYTPTGPTLAELAALGVRRV

SLGSLLYRRALAAAVTAATAVRDGRPTDLTAPSYAEVQALAEP

>CYP107U5(646973679)SBH

MTRYADARATLADPRLSKNPIHHSEAAHAKGKVGIPGERSADLMTHLLNIDPPDHTRLRR

LVSKAFTPRRVAAFAPRVQELTDGLIDGFQRRGEADLIHEFAFPLPIYAICDLLGVPRED

QDDFRDWAGMMIRHGGGPRGGVARAVKRIRAYLADLIHRKRESLRENAEGKAGEKADDLI

SGLIRASDHGEQLTENEAAAMCFVLLFAGFETTVNLIGNGTYALLRDPAQCDRLRRAAQE

GDEGLLATGVEELLRYDGPVEISTWRFATEPVVIGGQRIAAGEPVLVVLAAADRDPERFD

SPDVLDLSRADNQHLGYGHGIHYCLGAPLARLEAQTAIGTLLRRLPDLRLAVEPEEIRRR

GGLIMRGLRELPVTWGPII

>CYP156F1(646973684)SBH

MNNQSAFTEPPPGCPAHNSGRRIPMYGPEFAADPQAFYTHMRQYGPTAPVELAPGVDATL

VTDYSAALQLLQNPDTFRKDSRRWRDLNEGKVAPNNPVLPMLAYRPNALFTDGAEHMRLR

QAVTDSLAGVDAHWLSRQVERVSSYLISQFSALGEADLLNDYAKMLPLLVFNELFGCSPS

IGDRLVIGTSGIFDASMDAEKANEELTQALIELIALKRRQPGDDVTSRLMAHPAKLTDEE

MIHQLVMLISAGTEPQRNLIANGLYLLLCDEKHAGGSQAGGLLVEDVIDDVLWNSPPMAN

YAPHYPVCDVELGGNLLRAGDLVLISFAGANTDPALSSSRQILSKRAHLAWSAGPHACPA

KDSGQLIAVGAIENLLNQLPDVELAVPMESLQWRPGPFHRALTSLPARFAPVRAQQTAMM

SPAAQTGPVVTPAAIPQTRPQGKVQKSSAWSSFLAWWKS

>CYP1035A1(646973685)SBH

MSKTLQGLNPPVHTQQRRSLGNVFDRSTLAGLQPFVRQTVEELLDRLSDRLRATGEADFA

TLVGEELPVATISHWLKLPQADHALLRELTHGQAYAQELLPSASQLVQANAAAEGLREYF

TAVVAERRRSLGDDALSRWIRTWDELEPDREVADETLYHLVMFVVIASLETTSTLLSNMV

WLLDQNPRQRSWLLTHPEATPNAVEEVLRYDPPIHLTTRVATEDTELAGTRIERDEVVHV

MIAAANHDPSQFDDPDTFDIGRTAAHLGFGGGIHFCIGAMLARLEATELLQSLNRRFPTL

RVTTPPEWEPRMAFRRLLALHVAEH

>CYP156E1(646973711)SBH

MSQPYESVAQPTAVPPPGCPAHAGGTGTGTGTGAEPLYTPEFFQDPYAVYKRLRETHGPL

APVEIEPGVTAVLCVGYETALEIMRRPETFSKDARNWRAMTEGRVPEATHAAQLIKPRAQ

AGWVDGEAHRRLRSAIDDSMSRIDLGALRGYVERIADSLIDQFAAAGEADLIPEYCQLVS

MLAVGELFGCPKDIADRLFAAMAGLLDGRDPMAAAQEIDATMRALIELKRREPGPDVTSW

LIAHPARLNDEEMVEQLLLMMGMGSEPVPSLISNALRVLLTDERFAGDLAGGGMLVEDAL

DEVLWTDPPLSNLAVHFPLRDVTVSGTRLRAGEPVIISFAGGNQDALDSSQHKQGNRAHL

AFSAGPHTCSGRNQGRLIATAAIEKLLDRIPDIELAVDAEKLEWRPGMYQRGLTALPVRF

APVGELSKSEPAAPAAPAPQVPAAPEVPPAPTGLPAVPDAEGAAGEPGGAPGDADAAKPK

PTGLLGFLARWRRSRK

>CYP147F3(646974330)SBH

MTYGTYLRKITDPANRADPYPLYAELRKTPVLRDEGGPYLVSTYWEIHGLLHDPRLSSDP

RNLDPDAAAALAVTEEADDPTLPPSFLRLDPPEHDRLRRIATYPFGPPHTPRRIHDMRGE

LARIVTDLIDDFEGRDRVDLVDDFAYPFPVTVICRLLGVPRADEPLFHAWADTMLTSLDP

AAAEADVAQRRRAVRQARVELGMYLSELIEDRRRAPAEDMLSSLIHGDGPDGRMSHAELV

STAVLLLIAGHETTVNLIANGMLTLLRHQDVLKRLHDDSRLAVPLVEEVLRYEPPVQLLP

HRTPLTDIDIAGVTIPKGAAIWLVLASGNRDPKRFPDPDRFDPDRKDNQHLGFGSGIHIC

YGAPLARLEGQIALAELARRLENPRLLEDPPPYRHNAVLRGPRHLPVGFDGVRPAREP

>CYP157B5(646974624)SBH

MTTPDPTLPPEDPDTSAAPLPGWPEQPDAVLLHAEDFSRSQEDLYRRLRRDHGPVVPVLL

DGQIPVWMVLGYRELVRVTTDPNVFARDSRRWHSWHLVPPDWELMTFVGYRPTMLFTEGA

EHERRAEAIIDSMEIVDAFELRALCERVADGLIDTFAGRGHADLIADYACQIPLRVLNRM

YGMNEAESAELQQVTVDLVSGKGGPEAHQNMVDRMVALMEHARATPDRKNISTVLVNHPA

GLTDEEIINDLLGNVYAAHQPTTDLIGNALRLMLTDERFAVTMSGGRRSVGQAINEVLWE

ATPVPNWVGRWAAEDTMLAGRHIRKGDCLMLGLAAANADPDVRPDFRSGAAGNQAHMAFS

HGTHGCPPVARELAEAIAMAAIEVLIDRLPDVSLTAAPEDLEWRSKLMMRGVSAIPVQFT

PTSR

>CYP107P4(646974809)SBH

MSSSAAPAPVFDPWQPSFVADPYPAYAALRERGRAHYFEASRQWLIPRHEDVRVLLRDRR

LGRTYLHRFSHEEFGRTAPPPEHEPFHTLNGHGMLDLEPPDHTRLRRLVSKAFTPRTVER

LAPVVERLADELVEAFVAEGGGDLVAAVAEPLPVAVIAEMLGIPPADRAQLRPWSADICG

MFELNPGDEAARRAVRASVEFSEYLRELIAARRRNPGEDLISGLVAAYDEGDRLTEQEMI

STCVLLLNAGHEATVNSTGNGWLALFRHPDQLALLRSDPDALLPTAVDELLRYDTPLQLF

ERWVLDDIEVGGTVIPRGSEVALLFGSANRDPERFPDPDRLDLSRTDNAHFSFGGGIHYC

LGAPLARLELAASFGALLRRAPGLRPAAEPRWNPGFVIRGLQELLVEV

>CYP1037A1(646975198)SBH

MTDITFTPADVVDPELHASGEVHRLWRWMRQHAPVHWHEPGDLPGFWSLTRYDDIRQVYQ

NPAVFSSAQGVLLRPTDLGEDPGSGLTMALTDPPRHRALRGQVADRFSERCARSLAGEMR

AEIRSVVTRAVESGTCDVVHDIGARLSSHNIGRLLGVAPEDRERLLTWTTEAFESGKPLT

SHLELMRYFIDMMYARMEEPADDAMGMFVNNEVQGGLLTETEILLSCENLVGAAENAGLS

MASGILALAAYPQAWQRLVRERDGELVRTAAEEVLRWTSSATHSMRTATADTAVQGRRIA

AGDRVVLWIPSANRDESVFPEPERFDLGRQPNRHLALGTGEHVCIGSTMARHQMRMLLET

LAELVAVIEPAGDVEPLRSIAVNGPAHAPVRLVPR

>CYP107BM5(646975289)SBH

MKAAARKICGMTSPHSTATTTAPVHLTPELVEDPVGAYAEFRAQDQLPQVVLPGLTTPVR

LVTRYADVKAALAEPRLIRDRSKVPGGAGGGDPQAELVEAAFEGFPAEYAKYVAGHLALF

DGEDHARRRAPLTRAFTARRIAALRSFVERTAEDLIRGLTEKGEADLLGEFAYPLTTVVI

CELVGVDEADRGRVCDWIRDFAYGDGSRMADGLVGIVDYVKELIARRRAEPTDDLISALI

EGGGDSKGGDSKGSDGDEAFTEDELVSVVFLLISTGITPPALFLAHALLALFDHPDQLAQ

LRAEPELLGRAVPELLRYVTLVRIGASMYATEDFEFAGTALRKGEPVTVALLAANHDPRE

YGEAPERLDITREFGRGDGHLAFGHGAHYCLGAALGRLVASVVFDQVLIRRPGAALAVRR

DELEFGHWPADGFHLLRLPVRL

>CYP184A2(646976355)SBH

MTEVTEVPYAAGTPLLGSMSDLLGDPLAAYLRARRDHGDVVRFRAGPPGLRREIYGVFSA

EGIQQVLATEAANFRKDNAIYEELRQALGNGLLTSQDEDYRRQRRLIQPLFTRRRVESYA

TAVTSEAAALAARWRETPGGADGALELVEEMRGYALRVVGRVLFGSDVEQTIEVVRDSLP

MLNERARARALSPVKLPRDWPTPANRRAARAQAGLYALCDAIISKRMDGREREEREEREQ

APGPGHAPGPVPEDGAGPEDGAAEDLLTLLIRAHNAEDGSLTRAELREQVLVFFLAGHDT

TATALTFALHLLARHPAEQRRVHEELDRALPDGRTPTAADLEALPRLTMVLKEAMRLFPP

SPAVSRLAVAETVIGGRRIPAGAAVLVSQWVAHRHPAYWEDPERFDPERFTPQAEAGRPR

YAYFPFGGGPRACIGQHFSTLHSVLSLATLLRAYEVEDATDGGDIPLGAGITLLAKGPVR

VRVRSRT

>CYP154D2(646976483)SBH

MTDATDETDATDATDATDGQCPVPHAAAPHAAQDPHRLDPTGAGQHAVNARLRERGAAVP

VLLPGDVTAYAITRHEELKDFTTHPQVAKDACHFAALRDGEIPPGWPLATFATVRGMTTA

DGADHKRLRSLVTRAFTPRRVETLRPAVEELTAALLDRLAEAAAEAPDGVVDLRRHFALP

LPMGVICRLLGVGPEHQDRLHALSNDIVGTRTAPERALAANREMIAILGQVAAARLRSPG

DDLTSALIAAHEEDGDRLSEEELIGTLVLMIIAGHETTLNLITNAVRALCAHRDQLELVR

SGVGEASWGDVVEETLRYDSPVSLFPFRYPTRDLTVGDTVIPQGAPVLVSYTSAGRDPRA

HGADADRFDITRHTRPDAARHLSFGHGAHYCLGAPLARMEATIALDALFTRFPGLDLAVP

DDALVPHPSFVGNSPQELPVRLRPAGPAAHSG

>CYP157F1(646976484)SBH

MTADWADWADGALPAPVPLYGAAPAGDMPALYERMRRDHGPVVPVAIAPGIEAWLVLGHR

ELLHLTRDEQHFSHDPRRWTPLRDGRVPPDSPLIPLVGWRPALLFADGQQHRRMRSAVAE

ALGRLDGHELIRMVRSAAERLIAGFADRRKADLVPQYARLLPLQVVTQILGLDEERGPRL

VEAIAAIVNPTAAATGANRRMGGILLELIEQKKRQPGADLTSWLLEHPVGLSDQEVLHNL

VVIIVAGNQTTVNWIADTVRILLTDPGFRSSLARGHLTVDDALDLVLWRHPPTANFPGRY

ATRDLRFGGQHIRAGDMLILGLAGANADPEVLPEDGRPVIGNRSHLAFGAGPHTCPARDP

ARLITRTAVDTLRHRLPDLELAVPEDQLAWITSPWSKNLAGLPVRFTAPQLAADAADSAD

AAAAGHPADSGDLR

>CYP125A16(646976723)SBH

MSCPALPEGFDFTDPDIYQQRLPFPEFAQLRQTDRVWWIPQPHGVAGFDDDGYWAVTRHA

DVKEVSTKPEIFSSHLNTAIIRFNEHIHRDQIDVQKLIMLNMDPPEHTRVRQIVQRGFTP

RAIRALEDALRERATRIVAGAKEDGSGDFVTDVACELPLQAIAELIGVPQQDRSKIFDWS

NKMVAYDDPELAITEEIGAESAMELISYAMNLAADRKACPAKDIVSQLVAAEDQGNLASD

EFGFFVLLLAVAGNETTRNAISHGMHAFLTHPDQWELFKRERPASAADEIVRWATPVMSF

QRTATQDTELGGQRIAAGQRVGIFYSSANHDPEVFDRPEDFDITRDPNPHLGFGGGGPHF

CLGKSLAELEIRLIFNAIADAMPDVRLAGDPRRLRSPWLNGIKELQVNYG

>CYP154A5(646976751)SBH

MLAWAITDPTLLKQLVTDARVSKDPRQHWPKFINGEIPATWPLIAWVAVENMFTAYGADH

TRLRRLVAKAFTGRRTTALRPRIEHLVSRFLDGLAAGGPDTAVDLKAAYCHPIPMEVICE

LLGVPEGEMRERELEFVSTMFRTNAPPEEAIAAYQGHEALLRELVTLKRHSPGDDITSDL

IAVRDEDGSHLNEQELADTVGVLIAGGFETTVQLLDNAVHAMLTHPDQLALVRSGQASWD

DVIEETLRAQPPVVNVPLRYAVEDIELEGVTIKQGDAIILSLTAVNRHPEVHGEDSDHFD

ITRAVKEHLAFGHGVHYCLGAPLARLEARIALPALFDRFPELQLAVPAQDLLPLPTLISN

GHQTLPVRTGR

>CYP156C3(646976752)SBH

MPTSTPDSGPTPPPGCPAHTGPGQAPGLPESLYGAEFAENPSAVYARLRAYGPVAPVEIS

PGVHASLVTSYSTALEIMRNTETFPRDPRRWEALNNGTVPMDSPVVPMMMYRPNPMYTDG

EEHDRYRGAVSDTLNRLNPHALRTYVEESADLLIDLFGPEGEAELLSEYCARLPLLVFNQ

LFGCPPELSETMVKGMAALLDASEDAVEANAVLIETLLALIALKRLEPGADITSWLLAHP

ARLNDEELVSQLILLLGAGVEPVQNLICNALRLLLSDERFAGDLSGGTMPVDEALDEALW

TDPPIANYATHFPVRDVTIAGHRVGKGTPLLISMGAANNDPQLVSDRRGNRAHLAFGAGP

HACPAKDPARIIASTAVEKLLDRVPDIELAVDPAQLHWRPGPFHRALAALPVHFPPVAAK

YDQAGATATDQTTGGNAWTPSPAPSSSTPKGATSTGREPGSGNGAQRPWWNSLITCWRGR

>CYP156C4(646976754)SBH

MPTSAPDSDPPPPPGCPAHSGQASEPGRPEPLYGPEFAADPAAVYARLRAYGPVAPVELA

PGVHASLATGYATALEILRSPDTFARDPRRWEALADGTVPMDSPIVPLMMYRPTPMYTDG

EDHERYRGAVSDTLNRLNPHPLRTYVEESADLLIDLFGPEGEAELLSEYCARLPLLVFNQ

LFGCPPELSETLVKGMAALFDIDEDAMKADAVLREALSALITTKRAEPAADITSWLIAHP

ARLNDEEMTHQLILLLGAGVEPVKNLIGNALRLLLSDDRFAGDLSGGTMPVDDALDEVLW

TDPPVANFCTHYPVHDVTVAGHRLAKGAPVLISMAAANNDPQLISDRRGNRAHLSFGAGP

HACPAKDPARIITSIAIEKLLDRLPDIELAVHPDQLRWRPGPIHRALAALPVSFPPIAAK

YDAVSAAATDQNPGGNAWNPSPAPSTSTRSPSTPKDATSTERGRDSGNAAQRPWWNSLIT

WWRGR

>CYP268A4(646976874)SBH

MDAPRAVHAGEKRYDSVDLSSLAFWSQTAEERERSFALLRAERPVSWHRPVEGRLLPDPD

DTGFWAVVRHEDIMTVSRRTDLFASSAGVLLENIPEGLLEGSQSLLAMDPPRHTKIRRLV

SGAFTPRHILGMRKRIEAHARRIVGELARRPDGRADFMRDCASLLPMRVISDVMGIPREW

QDTVAATVSQSISGSGTDGEDGEGGERGERGEGGDEQSPLELLIEGNRRLRAMALNLAGL

RRGRPAQDLMTVLVQAEVDGDRLTDDDIADFFLLLCLAGNDSTTQTIGHGLRLLTDLPEQ

RAWLLAGLDGRIGPAVEEILRYATPVLTFRRTAVAPTRLGGRHISAGDKVVMFYASGNRD

AAVFREPGRFDLARDPNPQLSFGGGGAHYCLAAQLARAQLRILFRELLRMLPDIEAGEPE

FVTGTSIHGMTRLPCRFTPHA

>CYP124B2(646977564)SBH

MDPSFWLRPRDERAEVFEKLRALPGPEFVPPRLPWGPLASGYYALSKHADICEVSRRPQD

FSSEGATAILPPEMDEFYGSMINMDNPEHSRLRRIVARSFGRGMVPKFDAMSRRVARRIV

DELIERGPGDFIRPAAEMPIAVLSTMMGISGEDYEFLFERTNTIMGGADPELAADPEKMA

AAVLGALRDLGDYIGRLREDRLARPGPDVITKLVQVQEDGEQLTNQELVSFFILLINAGM

ETTRNVIAQALVLLTEHPDQRQLLLSDFELHARGAVEEILRVGTPINWMRRTATGDCEMN

GHRFRKGDEIFLFYWSANHDEKVFEDAYRFDITRDPNPHLSFGAVGPHFCLGAHLARIEI

IAMLRELLASLPDIRVEGEPVRLASSFIEGFKELSCTF

>CYP157C7(646978053)SBH

MTPPPDHPGTDHLGTAPPPGCPAHAAIRSGTAGDLFGPEHEADPKGFYEWLRAEHGAVAP

VRLAGDLPAWLLLGYRENLEVARTPSRFSRDSRHWGDWREGRVASDSPLLPVVGWQPMCT

FADGAEHERLRAALTESMNRFDRRGMRRQVTRFTHQLVDEFAADGHAELVSGIAEQLPML

VLTHLLGMPDEYGPRLVEATRDLMKGTETALSSNAYVTETLENLVARKRAEPGSDLASWL

LEHPSGLTEEEVVQHLRLVLLTGNETTTNLMANTLRTVLTDPRFRASLAGGHMTLPDAIE

HVLWNEPPLAVIPGRWATGDTELGGRQIRAGDMLLLGLAAGNVDPAVRPDIAAPMYGNRS

HLSFSGGPHECPGQDIGRAIADTGIDTLLLRLPDLRLAVPETELSWTASWISRHLVSLPA

EFTPRGGDSDTLETETGLLAPDFLARSAPQPDPPDQSKALDRPNTPDRPDVRRSSRRRSW

WRSLWRRLRPGPRPPRR

>CYP107E5(646978295)SBH

MTTAETTVQSEAIAYPFNSSEGLGLSQAYQEARNRPGLVRVRMAYGEPAWLVTRYAEARL

VLGDRRFSRAAALHRDEPRQGEGRRNSGILTMDPPEHTRLRTLVAKAFTVHQVEKLRPSV

RQLTHELLDGLEAAGPPADLVDRYALPIPVGVICRLLGVPEEDRPKFRAWSDAALSTSSL

SAEEFDRNRDELRAYMAGLIEIHRAAPQDDLMTSLIEARDAGDRLSELELVDLCVGILVA

GHETTATQIPNFVLTLLDHPGELRRLREEPALLNGAVEELLRFVPLGKGASQPRYATEDI

EVGGQLVRAGEPVLVAVGSANRDALRFDEPGKLNVARPATQHLGFGHGVHHCLGAPLARL

ELQEALGALITRFPGLCLTGDVVWKDQMLVRGPRVMPVGW

>CYP107AM-fragment1(646978401)SBH

MTRAVRMGLPDRMAKEALEVLLRRLPSLEPAVAVEDLRRLEGLAVGGLREVPVRW

>CYP163C1(646978424)SBH

MDTRRALPPQPLDQLDLADPLLHAHHDLGPLWRRLRAEAPVHWQPETARGPGFWAVSGYA

DVVSVLGDSETFTSERGNVLDTLLAGGDSAAGQMLAVTDGRPHQALRSALLKPFSPRSLD

VVVDSVRRGTRALVEEAVERGEVDFAADVAAHIPLAAICDLLGVPAAERQHIIDLTSGAL

SSADGAPTEEATWASRNGLLLYFSELAAERRAKPYDDVVSLLVTKEIDGRPLTHEEIVFN

CYSIIMGGHETTRFAMVGGLRALMEHEDQWQALKSGRVATASAVEEVLRWTTPALHSGRT

ATRDVLLDDRFVEAGDIVVTWLASANRDERVFDRPDEFDLSRTPNKHLSFAHGSHFCLGA

FLARAELTALLESARDLVDKAEPAGPPRYVYSNFLSGMSALPVSLTPSCRVLAGPVAWEG

PTAGLTVGNMTHTPAAVGAEAVSAVVCGMVRLVAPRKLDQVELDHRLVGDLGFHSLVLAE

LGYNLEDLYGLRALTPEETMKLERVKDVIEFVSTEVASGRAQLPDEEELSDLFARYGADA

PTA

>CYP161C1(646978838)SBH

MKELPRLPFDNPAMLGIAPQMRALQQEEPITRVRTAGEDAWLVTRYDEVRALLADRRLGL

SNPNPEQSAKSAARRFMVALMAGDDYDTEATRHAQMRALLVPRFSTRRMRLMKTRIEHHV

DDLLDQLAAGTPPVDLHRALSFPLPTMVICDLLGVPLADRERFGQWARGTFDQSDNQHSA

NTFQQVVDYITELVARKRTEPGDDLLSELIAHKDSALSDADIAHLGNAVLLFGYETTIVR

IDLGTLLLLRNPAQRALLAENPELAPAAVEEILRLGVGGKGSNAIIPRYAHSDITVGETV

IRTGDAVMLAIGAANYDGRAFPDGDLFDLARNKPKSHLAFGHGARHCIGRTLARIELTAV

FERLFRRLPNLRLAVPEESLRWQEHRITGGFDELPVTF

>CYP183A2(646978846)SBH

MTEQTTFSAGAAPGALPVVGHALQIMRHPVNFMASLSAHGDLVEIKIGPTTAYVPTHPEL

LRHVLTNDRIFDKGGIFYDRARDIAGNGLVTCPFADHRRQRRLMQSAFTRGQLKRYAEAM

HAEIEDTASRWQDGMVVDAFQEMYGLALRTVGRTLYSTPVSPELAAKVERSFDVVLNGLF

RQMFLPASIRRLPLPSQRRYKSNLDFLHETTQQLIDDYRSSDTERDDLLAALIASRDDDG

GKLGDKEIHDQVITVMAAGTETVAATLTWVFYLLSQHPEIEAALYDEIDTVLDSRAPQWD

DLPNLSLADRIISETLRLHPPAWLFTRLTASPTELAGRQLPTGSTVVFSPAAVAQYEDAF

DNPKKFDPDRWLPDRIAPASRHAYVPFGTGARKCIGDLYARTEATLGLATILGRWRVTCE

PGMDIRPVPLATVYHPRRLRLRLDARTPRRKTSAVPVPAGGDVT

>CYP1036A1(646978897)SBH

MSQIPASDVDLYTESARTDPYEIYAELRALGPVVHLSRYDLYALPRYDEVRAALMDWQTF

SSARGVFVDPDVNAQLEGITLCSDPPEHTAMRSVLGRPLRPDRMREVTPRIEAEADQVVE

RVVGRGRFEVVTELAEYLPMTVVSDLVGLPDHGREKMLEWAAAIWNTQGPADDRAAAAGP

AVEEFMAFAMNDAVPGKIDPDGWAAQLYEAADRGEIPHDKCPVMMLDYVTPSLDTTILAI

ANAVALFAQNPDQWDLLREDRSLIPHAINESLRLESPVPQFSRVLTEDHEIGGVPLAAGS

RVALLYASANRDERHYPNPTRFDITRRPSDHLAFGRGEHVCVGMHLARLEMSALLERLAD

RVARFEILERRPMINNGLRGLDYLDVAVTPAP

>CYP107AS-fragment1(2511677887)SCT

MTRFPPTPSLFHPPPELNGLQRDRPVTRFRLADGTTGWPATRYEDVCQVPTDPPEHTRPR

RLVAGAFTARRADAMRPRVTDVDADLVENS

>CYP107CR1(2511677904)SCT

MTDSTKPCASGTGTAEADLLSWDFIQDPYLTYHEMRAGTGPRRLVIKTLSTGLRSWLVTE

YSDVRRLLADPRLSKAAGGAAPIIAKHSTEDVSGAAITSESMLFSDPPQHSRLRRMFSRA

FTMRRTLDLRPRVEELTDELLDAIPTGAEIDLVESVAMAIPIAVIGELLGVPRAAHQDLR

RWNRALTSVDSEPSEKYQAYMASLEYFRTLVAQKASSHDGADDLINAMIDPDNEERFEES

ELLSTIFLMMNAGYETTANLISSSVYALLKYPEQLELLRGDATLIPNAVEEFLRYESPLN

LSTLRYTTEPVEVGDTVIPAGEVVFLALSSANRDPNRFAEPDRLDIRRNAATHLAFGHGI

HHCVGAPLARLEGEIVLARLLDRFSHWEAAEPLERLTWRYTLQFRGLERLPVRLHA

>CYP105D12(2511678066)SCT

MTDALTTQQSQALPFPQARTCPYHPPAGYRDVRQTGPVGQVRLYDGRLVWLVTGHAQARA

LLTDPRLSADRQNPRFPVIAPRLAELIRRVRSPLLGVDEPEHGVQRRMLIPSFTVKRTAA

LRPRIQRVVDEALDAMVAQGPPADLVASFALPVPSTVICLLLGVPYADHEFFEGRSRQLL

RGPRAEDVESARQDLNGYLRDLVRARRSRPGDGLLDELVAEQLEPGHLTEDELVSMALLL

LVAGHETTANMISLGTLTLLEHPEQLAAVRTDPGVIPDTVEELLRFLSIADGMARVATAD

IEVAGVTIRAGDGVFLATSEINRDPGAFPEPDALDVRRGARHHVAFGFGVHQCLGQNLAR

AELEIALTTLFTRLPGLRTTVPAEELPVRAGDVIQGLDVLPVTW

>CYP105B23(2511678077)SCT

MTITHPGSTFSVPAAKSGGCPFDPPPAYQRARDEQPVTRVTLWDGSQCWLVTRHQDIREA

LRDRRLSSEADRPGFPFITPNRRALASDRRGNTSFIRMDDPEHARLRKMLTPDFMIKKTE

TLRPRIQEIVDDFLDRMIAKGAPADLVADFALPIPSLVICLLLGVDYADHEFFQERSRIM

LHNNTTPEQVAEARDDLLDYLGDLAAAKRARPDDSIIGKLAARPELSHDEVASMGLLLLI

AGHETTANMTALGTLALLRNPGQLAALRDDPALAPSAVEELLRYLTIVQSGVARVAKDDL

EIGGETVRAGEGVLFMISAANRDPEAFPWGDDLDITQDARRHLAFGFGVHQCLGQPLARV

ELQVALATLVRRLPGLRLAIPFEDVRFRTDMAIYGVHELPVAW

>CYP1062A1(2511678833)SCT

MHDVVNCPSAPGGLPLIGHTLAVLGRPLSFFESTRTDDPLVRVVIGRLQVYLANDPDLVH

RIQVDTDTFERGRFFEVLAGHFDNPPIASNGPAHRGQRRALKPVFNRPSVRQYTDAIVEE

AEALAAGWRPGRLPDVRQQLSDVVACTVLRCLFSTELSTEDVHDIQRTIYQVARRLLPGT

LLPAAVTQVPTPGNRRLAEAMGRFRVLVERLVREREEHGQGHHDALSALLHARHGATGRP

LTPREIRGEFLVLLFAALETTSTTLAWALYEIATHPRVAARLADEVDAVLRDGPATYDGL

QRMPYLRQVLHETLRLHPPTLFTRRTRHAVTLAGVAVPAGAEVGYSPRAMHRHPGLHRDP

ARFDPSRIGSEGALPQGAFFPFGVGAHRCIGEHLAMTTMAAVVAAVVARWTIRLPPRVRV

RETISSMPHPDSLPLQLTTRPSASRRSARYATARYSHPA

>CYP125A24(2511679210)SCT

MSCPAMPDGVPPAGFDFTDPDVYATRVPLPELAALRATAPVWWNAQPHGVAGFGDDGYWV

VTRHADVKEVSTRPEVFSSAANTAIIRFHEAMTREQIDVQRLIMLNMDPPEHTRVRQIVQ

RGFSPRAIRSLNDALRERAARIVADARRTGSGDFVTDIAVELPLQAIAELIGVPQEDRAR

IFDWSNKMVGYDDPELAITEEIGAQSAAELISYAMNLAAARKECPAKDIVSTLVAAADEG

NLGADEFGFFVLLLAVAGNETTRNAITHGMHAFLTHPEQWELFKRTRPATTADEIVRWAT

PVVSFQRTATRDTELGGARIAKGQRVGVFYSSANHDPEVFDRPEVFDITRDPNPHLGFGG

GGPHFCLGASLARLEIDLIFGALADTVPGIRQVGEPRRLRSSWLNGIKEMRVTYE

>CYP107U13(2511679895)SCT

MHDHEPAAPSPSLFSWEFAADPYPSYAWLRENSPVHRTTLPSGVNAWLVTRYADARQALA

DARLSKNPVHHSERAHAKGKVGIPGERSADLMTHLLNIDPPDHTRLRRLVSKAFTPRRVA

EFAPRVQALTDRLIDGFAGRGSADLIHEFAFPLPIYAICDLLGVPAEDQDDFRDWAGTMI

RHGGGPRGGVARAVKRMRAYLLELIHRKRAALGDDLISGLIRASDHGEHLTENEAAAMAF

ILLFAGFETTVNLIGNGTYALLRDPGQRRVLTDALAAGETAVLDTGVEELLRYDGPVELA

TWRFATEPLTIGGRRIGTGEPVLVVLAAADRDPARFDRPDTLDLARRDNPHLGFGHGIHY

CLGAPLARLEGRTALATLLTRLPDLQLAVPEGELRWRGGLIMRGLRELPVTFTPEPDRSA

DTGANKVN

>CYP107AE6(2511680597)SCT

MPMPTVLGVSPTTTSPSRLEPDGMSDPTTDPAFLRDPYPTYAALRSKCPVQQVPSGLGGH

SSYLVTGFEEAREALADPRLSKDTAAFFAGKESRRRLHPAVAHNMLATDPPEHTRLRRLV

TGSFTTRAVEELRPFIARTTDALLDQWPAEGSVDLVAELAVPLPVIVICELLGIPEPDRA

EVRRWSGELFASGAPAVIDAASHSLADYMTGLIANKRSRPGQALLDSLIAARDGDDRLSE

EELVSLGVLLLVAGHETTTNFLGNAVLALLQHPSELQRLRQYPAEITSSLDELLRYDSPI

STATFRFTTEAVTLGGIEIPAGKPVLVAVGAANRDPARWPNPDELDLDRVAAGHLSFGHG

IHRCVGAPLAKAEADIALRKLLTRFPDMQLAVPAEQLTWRRTRLVRGLAALPVLT

>CYP157B11(2511681006)SCT

MTDPTPATGCPAHPDALPLSGPRYQQTPAELYRRMRHEYGPVAPILLDGGVPAWLVLGYR

EVLHVTGNDQLFARDSRRWHAWSRIPPDWPLLPFVGYQPSVLFTEGAEHRRRAGAISEAL

SGVDQFELRAHCERVADELIDAFAGSGEADLMADLAHPLPLLAAIWMVGLPGAATQDLVR

DLTASLDVVEGVDPVAAYQRVQDRIQHLVAQRRSGPTADVTSRMLEHPAGLTDDEVVQDL

ISVIAAAQQPTANWIGNTLRLMLTDDRFAITLSGGRRSVGQALNEVLWEDTPTQNFIGRW

AVRDTQLGGRRIKEGDCLVLGLAAANTDPQVRPDAHAGPGGNHAHMSFSHGEHQCPYPAP

ELAEVIARTAVEVLLDRLPDVVLAVPAERLVWRPSVWMRGLEALPVEFTPSYAVGPAR

>CYP159A9(2511681007)SCT

MADILSPEFAADPYPAYRVMRREAPLIWHEPTRSYILSRYEDVARAFKDPVFTTDNYQWQ

IEPVHGRTILQMSGREHAVRRALVAPAFRGRELQEKFLPVIERNARELIDAFRTAGEADL

VAQFATRFPVNVIADMLGLDKADHDRFHTWYTSVIAFLGNLSGDPEVAAAGARTREEFAA

YLLPVIAERRAHPGDDLLSALCAAEVDGTRMSDEDVKAFCSLLLAAGGETTDKAIASVFA

NLLRHPEQLAAVRADRSLIARAFAETLRYTPPVHMIMRQAAEDVEVSGGVIRAGATVTCL

IGAANRDEERYADPDTFDIFRTDLATDNAFSAAADHLAFALGRHFCVGALLAKAEVETGV

GLLLDAMPDVRLADGHLPVERGVFTRGPESVRVRFTPAAG

>CYP184A4(2511681057)SCT

MSKQAPLVAGHPLLGSLPDLRDDTLGAYLRARRDHGDVVRFEAGPPGLRASLYAVFSADG

VQQILGTEASNFRKENDVYTEIRESIGNGLLTSQDEEYIRQRRMIQPLFTRRRVDAYAGA

VHDEATATAHRWTTASGNVVDVVPEMAEFALRSVTRILFGSDIESAVDVVRRNFPVIGES

VLRRGVAPLRIPRTWPTPANRRTATAQRELYAICDRIIAERTAAGHDDSGDMISLLIGAR

DEDGSALDADSVRDQVLVFLLAGHETTATALAFGLHLLARHPDAQHAARAEVDAVLDGRP

ASAADLERLPYLTRVFKEAMRLYPSVAIMGRRSVADSEVCGFHIPGGSDVYVSPWVTQRH

PDYWENPDAFDPDRFAPDLEAARPRYAWFPFGGGPRSCIGQYFAMLEGIIGLATFLQSYE

FAAVDAEVPLELGMTMRAAGPARVRLTRRPSSGEAAA

>CYP107CS1(2511681384)SCT

MSVTRPSPELSEYDLSDVAFITDPEAGRRWLAGPRPICRGRSFDGSQAWIVTGYEDAKQV

LTDPRFTSRPPGDSHARGLRARGMPEDLVSLFDSMLLSMDRADHDRVRPLVTLAFSARRV

RTMRPFIESLVGELLDAMDPAAENDLVAALADPLPIRVVSELIGVDEVHREQWLRWAQTF

NGPVPPPADQLAPALRGMVEVTRDLIAQRRRRPTDDLISELIRTRDEDGGRLTDDELAAL

AILVIQAGHDSVRQLIALTVLTLLDRPDQLALIRSGRTTWSVALAEVMRHAAPVKHAFRR

FATEPVEVGGVTVAPGEGVLVVLAAANRDPAEFPDPLVLDVTRTPNPHLGFSRGPHFCPG

STLAMTEVEIAMRELFGRFPKLRLAVAGDEVAPRFLLGVQRLPVLLD

>CYP107W2(2511681389)SCT

MEPETTEAPLPFPFAPAPAVCLPSPALAEVRETCPVTRVRLPDGTQAWLVTRHADVRQVL

MDPRFSNRVVAARPEVAESEHGALISQSLIGMDPPEHTRIRRLVTRAFSARRVERLRPRV

AELVDGLIDALEELPRPVDVVRHFAVPLPTTVICEMLGVPEADRAAFQEWSNALVVDWLR

DEGERNAATAALRGYFTELIAAKRAEPGDDLMTELIAAREEGNKLSESELVAQCIGLLSA

GNDTTASLIAMFLMTLLRRPDELARLRAEPAEIPRAVEELLRYVPLAMSGAGGPRLTTEE

VELGGVTIPPGKLVLPAIAAANRDPEVFADPERLDLDRTDNQHLGFGAGIHFCLGAQLAR

VELQEALAGLLRRLPGLRLAVPEEELRMKPASAISGLEALPVEW

>CYP1199A4(2511682187)SCT

MAVSATDRRGRVTVFTPRIDQLLRERRGSDLFRLDPGTIGIAGADLIDTLLASRPANENE

RPTFKPLQGRSISRTEAATVMQAVSHDVKAALKKPAPNNIDLSGEWPHVGHVYLRDMVFG

ADPYRLRVLVDRKLELTTKLTWSVIATGAARPLSPEPSLSRLGGLTTAAGTYNDRRHAMG

LYRRAAAPVCFTVSTLVANALWLGAPFDDDTPNLHILLESMRLLPPSWNILRVASPEFPA

IDARIGATDDILILPLLSHRDPAIWPDPDDFRPDRWADLDGDNQRGYLPFGHANERCWGR

HMVMPLAEHLLDLLRTGGYTVDPAQRSATVPLAGLLGVTGVRVTRT

>CYP107L23(2511682599)SCT

MTTVNDLTTYIDNDADDDILDLRALGPRFRRDPYPVYAGLRARGPVHRVLTTTGTEAWLV

VGYEHGRAALVDPRLSKRWSHASDALRLIAPAPGTHMLNSDPPEHTRLRKLVVKEFTPRR

VESLAPRVQQITDDLLDAMLAAPDGRADLVDAFSFPLPISVICELLGVPFLDRRSFRAWT

DDLLSATDPAQQEATVARVGRYLGELIEEKRRHPGDDLMSALIRTSDEDGDRLSPDELLG

TAWLLLVAGHETTVNLITNAVLALLTHPDQLAALRADPSLVDNAVEETLRWDGPVETPTF

RFTTEPVEIGGTVIPGGGQLVLVALGDADRDPARFPDPERFDITRQPGGHVAFGHGLHYC

LGAPLARLEGRIALRTLLERCPHLALDAHPGELEWRPGLLIRGPYHLPVRFTR

>CYP102G4(2511682645)SCT

MSPTPHSASGTTGAAAATPGAASPAPPVPVADISDTGFGTTPIQQAMALAREHGPVFRRR

FGTFESLLVGSVDAVTELCDDERFVKAVGPVLTNVRQIAGDGLFTAYNDEPNWAKAHDIL

LPAFALSSMHTYHPTMLRVAKRLIAAWDTALADGAPVDVADDMTRMTLDTIGLAGFGYDF

GSFRRGEPHPFVAAMVRGLLHSQALLSRKADDGVDHSAADEAFRADNAYLAQVVDEVIEA

RRASGETGTDDLLGLMLGAPHPSDGTPLDAANIRNQVITFLIAGHETTSGALSFALYYLA

KNPAVLRRAQAEVDALWGDDPDPEPDYTDVGRLTYVRQVLNEALRLWPTAAAFGRQAVTD

TVLDGRVPMRAGDTALVLTPVLHRDPVWGDNVEAFDPERFSPEREAARPVHAFKPFGTGE

RACIGRQFALHEAVMLLGMLIHRYRFLDHADYRLRVRETLTLKPDGFTLKLARRTSADRV

RTVASRAAEGTAGQDAGLPTTARPGTTLTVLHGSNLGACREFAAGLADLGERCGFETTVA

PLDAYRAGDLPRTSPVVVVAASYNGRPTDDAAGFVSWLEQAGPGAADGVRYAVLGVGDRN

WAATYQKVPTLIDERLAECGATRLLERAAADAAGDLAGTVRGFGEALRRALLAEYGDPDS

VGAVAGAEDGYEVTEVTGGPLDALAARHEVVAMTVTETGDLADLTHPLGRSKRFVRLALP

DGATYRTGDHLAVLPANDPALVERAARLLGADPDTVLGVRARRPGRGTLPVDRPVTVREL

LTYHLELSDPATAAQIAVLADRNPCPPEQAELKKLAPGRASVLDLVERYPALTGRLDWPT

VLGTLLPQIRIRHYSVSSSPAVSPGHVDLMVSLLEADGRRGTGSGHLHRVRPGDVVYARV

APCREAFRIAAGDEVPVVMVAAGTGLAPFRGAVADRVALRSAGRELAPALLYFGCDHPEV

DFLHAAELRGAEAAGAVSLRPAFSAAPDGDVRFVQHRIAAEADEVWSLLKGGARVYVCGD

GSRMAPGVREAFTALYASRTGATAEQAAGWLADLVARGRYVEDVYAAG

>CYP158A13(2511682837)SCT

MTTRPHEESRETGRDDAPPVRFWSVDEVEAMDFDPFMEELLQQDSPALVKLPHGTEPAWV

AARYDDVKLVTSDPRFSREALVGRDVTRLAPHFIPLDDAVGFADPPEHTRMRKTVAAMFT

HRRIEKLRPRAEEIAGRLLDTMERAGPPADVMEHLNTPFALGGMSELMGVPEEDWPKMAK

WARLVISAEAGREASEQAKHDIGAYFADLAAQRLAEPRDDVLSHMAAAERDGRLTHQELV

AFAVLMQISGTNSVRFNSSNMVYLLLTHPDHLARLRAERELLPQAVDELLRFVPHRNAVG

MARVAVEDVRLGDVTVRRGDPIYVSYLAANRDPEKFSCPHRMDFDRTFNPHVSFGSGPHY

CVGASLAKMECEVMLGGLLDRFPRLRLAVAPEEIEWRRGELIRGPHALPVTW

>CYP105B24(2511683422)SCT

MTLSHQPLPDLQLPAPRGACPFAPPPAYRQAPIARGTLWDGSTPWIVTRYQDVRAVLADP

RFSSDPHRPGYPFPTPGYRALMRDNPALLGMDSGGYERLQRLVPGYFRGAAMEALRPRVQ

RIADELLEEMAAGGTRADLVTALALPLPSRVICLVLGVPYEDHAFFHRRACLLVDHHGDP

EEIQRAAVELLGYLERLAATLRRAPDGSVVGRLAAQGELSTREIAGLAQLLLLAGYETTT

NMIALSVLALLRNPEQLARLRGRPGLVPGAVDELLRYLTVIQAGVARAATEEVTVAGQLI

RPGEGVLCMVSAANRDAEVFAEPDALEVTRPARRHLAFGYGVRRCLGHRLARIELEVALA

TLWRRLPGLRLAVPFEEIVFRPEVIIYGVAALPVAW

>CYP1038A4(2511683423)SCT

MTAAVPRPPQAPSTPARQAPPGPALIEVLTRAAGHGSPAAFELDGRPMTLVTDPEQVRQV

LACRPEVYVKHSHRARALLGDGLITATGDAWKRQRRLLQARFTVTGVRRYEAGIAAAAER

IARRWSAAAGTGDLVDVGEDMRFFALDTIWRALTGDPLDEAAHRELAAVDTVVAALPTTA

GAPTGDPAEVGAALGRIDATARRVIAAARARRAAHGPSDALLDLLLDASGSDAGDPDRLV

RDELVTLLVAGHETTAQTLAWLFLMLHQNIHVPRTSEPGALVAETLRLYPAVWLVPRCAA

RDDVLGGRRVAAGSGVLVCPYLTHREPAWWPDPERFDPARFLPGGVRPAHPGAYVPFGLG

PRACLGQQFALRETAALLARLLPAFTVELRDPPAAPVFGANLRPGGPLPAVVRRRT

>CYP183M1(2511683426)SCT

MPPNTVPTPVPGGRPLIGHARQLLWRRLPFLESLRDHGDIVVIRLGPWRIHVLNDPALVR

DVLTKRSPDFGLSPQFQVMKRVIGNGLLATDGPFHRRQRKLILPALHHTRIRAYARTMTR

LADARTARWQDGQTLRVDAEFTELATEIVLRCLFSTEIGGADVAAVVAALPDLMSWAGSR

GLDPTGLLGAVPTPLGRRFRRSMAVLDALLARVIGARRADGPATDHPDLLAALLAARDAE

TGEPMSDRQIRDEAMSFLVAGAESVSRTLTWSALLLAGDPEAARRLHQEADRELSGRPAH

FEDLPRLRHTRMVLQEALRLYPPGYLISRAALRDTTLGPYRIPAGATVMFSYYALQRDPR

RFPDPARFDPLRWSPKRGGADREAFTPFGLGPHGCLGESFAWTEMSIVLATLAARWELRS

ASPRPVRPVPTFSLTMAGAPMTVTARPVRTGPVHTLLASRNGG

>CYP107E11(2511683448)SCT

MRLHTAEPAGTADAEPVPYPFNEADGISLADAYEEAREQPGLLRVRMAYGEPAWLATRYA

DARLVLGDRRFSRAEGARHDEPRQSEGRRDSGILSMDPPDHTRLRTLVAKAFTMHQVEKL

RPAVRELADELIDKMVATGAPVDLVEEFALPVPVGVICQLLGVPVEDRPRFRAWSDAALS

TSSLTAEEFDANQEELRAYMRGLIEDHRARPREDLITGLIEARDRDDRLTEQELVDLCVG

ILVAGHETTATQIPNFVVTLLDRPEQWNRLREDPELVPTAVEELMRFVPLGSGASFPRYA

TEDVEVGGTLVRAGEPVLVAVGAANRDPARFDAPQELDLAREGNQHLGFGHGVHHCLGAP

LARLELQEALGALLRRLPGLRIAGDIEWKTQMLVRGPRTLPVGW

>CYP105AA10(2511683531)SCT

MSQALPIPEGLPAERDAGPFDPPRGITRMREARPVMPLIFPDGHEGWLVTGYDAVRQVMA

DTRFSSRLDIGIVHVPYQTPGMPAPTEPSPQIPGMFIAMDPPDHTRLRRKLTGAFTVKRM

KMLEEHIIDITERQLDALARLTPPVDLVKEFALPVPSLVICELLGVPYEDRETFQSNSAQ

FLVKDQTVEEKVGAYNALTTYLAELVTRKRAEPGDDILSDLARHDDLTIEELTGIAFLLL

LAGHETTANMLALGTFALLENPGQLAELRADPGLIPDAVEELMRYLSLADVFYRYATEDI

ELGGETIPKGSTVVVSLLAANRDPHRFDDPDTLDIHRKARGHLSFGHGVHQCLGQQLARI

EMRAGFDGLLRRFPTLHLAIPADQVKLRTDMNIYGVHELPVAWTETPR

>CYP107CT1(2511683575)SCT

MTSANNDASGGCPVGGTHHACPVELDQEFYADPHPTYRAIKAQGNRPTPIVLRTGMAYLP

PGLRGWLVTAYQDVEFVLRDPRFRKSIDEAMPLFAAGTGREGGGDRSSLLYDNMANNDPP

KHTRLRKPLNATFTARAVATKRRDMRRVATETLDALAGRDTFDLVQDFAFPFSIAVICDT

LGVPREDRGTFHSWVQTITGAADHETLRRDTGLMAQYLRDLIGRKRAGTADDVLTQLATS

LAEDEAVAQAYALLAAGYETTANLIVTGFLTLERNPEQKRRLWSDPSLVPGAVEEMLRHQ

SPFNLSLYRYVTEDVEVGGVEIPAGAIVFLSFAAANRDEHRFADPDAFDITTPRREHLAF

GGGIHNCIGKHLARLEAEVAFDALVRRCPGLSVVTPADRFVWKASPTFRGLKNLAVGPGP

RD

>CYP105B25(2511683586)SCT

MTLTSDPAPLPVEPPAGCPFDPPAQFARLRTEEPISRISLADGSWAWLATRYADIRAILG

DPRFSSDTTLPGYPLSGMTGGASTENRGFIRMDPPEHTRLRRMVTREFMVKRVEALRPEV

QRITDELCDEMSRAHAAGEPVDLVEALALPVPSLVISLLLGVPYEDHTIFQRLTGTLLSR

TVSEAERDVARGELRDYLHQLVSAKEAEPGDDILSRLIVEQERPGEITHDDVVAFAALLL

VAGHETTANMIGLSALTLMRDQASADRLRAEPGLIRGAVEELLRYHSIIRNGPRRVATED

VEVGGRLIRAGEGVVAAVPSANRDETVFADPDRLDVCRPNAQHHVAFGYGIHQCLGQALA

RVELQVVIGTLLRRFPGLRPAVPVEEIPFRTDMAIYGCHALPVTW

>CYP183L1(2511683729)SCT

MASRTCATPVVPGAFPMVGHAWPLMRRPLRFAQSLSGHGDLVEVRLGPVRAQVPCDPELL

WRVLTDDRLFDKGGPFFDRVRATIGNGVGACPYGEHRRQRRLIQPSFQPARLKGYAAVME

EEAAALTERWDDGGVIDAYRSLFGAALRSVLRTLFATRAGDAVVDRFRTSVETVLQQLTA

RMFVPAPLLRLPLPVNRRFDRAVADLRRGIDDLVAERRRDRTDRGDLLSALMTAQDDENG

TGCGTGLTDTEIHDQVLTMLTAGSDSVTAAVSWALYLLDRNPEALDRLEREVDAVLAGRA

ARGDDVPALAHTGRVITEALRLYPPGWLFTRVTTAETELGGHRLPPGTTVAFCAPAVHRG

RQLYDDPEEFDPDRWLPERARTLPRGAFTAFGGGARKCAGEAYALTECTLLLATIVSRWR

LRPAPGCDVRPVALSTALRPRRLLMETSARRGLPK

>CYP163D1(2511683814)SCT

MNTVTGVTTFPDLTDPAFWARDDSHAVLRELRRRSPLWRLESEAEGPLWCVLSHALANEV

LGDAARFSSERGSLLGTGRDRAPAGAGKMMALTDPPRHRDLRGLVLPFFSKRKAAELGAR

VADLTRQVVRDALGTARTDFVRDISTTVPLTVMCDLLGVPDEDRDHVVAMCDRAFLGDTP

EERSEAHQQLLPYLFALGLRRRTDPRDDIISQLVTHEVDGRRLPLDEALLNCDNILVGGV

QTVRHTSTMAMLALTRHPHAWQAMRADGYDPETGVEELLRWTSVGLHVLRTARHDTELAG

HHIRAGDRVVVWTPAANRDEAEFHHPDDLLLDRTPNRHLAFGWGPHYCIGAPLARVELAS

LFAALTEAAEHVEVLEPPVPNRSIINFGLDALVVRLHPRGAAG

>CYP156B12(2511684117)SCT

MDSRQGPIPPATPVGCPAHHTTQAARMPLYGEEFAADPGAVYDRLRAYGPAAPVELAPGV

EATLVTEHESALRVLQSPALFARDSRRWKALAEGRVALDSPVLPMMVYRPNCLFTDGTEH

LRLRKAVTDSLGRLNVNRLGRDVERIADYLLDQFSERGMADLLNDYAKLLPLLLFNQLFG

CPAGIGDRLTANMAAIFDGQDVMRANEELTACLMELVALKRRQPGNDITSWLVEHPSGLN

DEELKDQLVMLMGAGVEPERNLIGNALLLLLSADSSTGRSGMRVEDAIDQVLWNNPPIAN

YATHFPVQDVELGGVPLPADTPVVISFAGANTDPALTEAHQVLSKGAHLAWGAGPHACPA

KDPAQLIALTAIERLLNALPDLTLAVPQGSLRWRPGPFHRALVALPVRFSPTPGLRTAAA

RPVTASQPATVPPARTQTASSSGGTAPAKQKGWWSSFLDVFRL

>CYP1035A7(2511684118)SCT

MLGRLRSAQGLADPAPLYDELRSLGDVVPAPWGGYFVTGFQTCSQVLRSRTWLVPDFAWQ

ERQPDPARWQAPATREMTGTLSRLNPPVHTCQRRSLGNLFDRATLTALTPHVERHVAGLL

DGLERRLRADGTADLVTEVSERLPLLTVGGWLGIPVADHRHVLQFTHNQVHAQELLPRKS

ELEQSARATVQLRAYFTDLVRQRRRAPGDDPVSHWIRTWDAMEPDRAAVDEIVYRLTMFI

TIASLETTATLLSSMVWHLLRRPARWDWLRENLQHVADAVEETLRYDPPVRLNSRVAAED

TELAGVPIRKDEMVHVMYGAANHDPRRNPDPHVFDILRRGIHLTFGGGAHYCLGAALGRL

EARTLLTRMLQRFPALRAVAPPEYAPRMVFHRLTSLRVTA

>CYP105BD1(2511684125)SCT

MDPVPPARQAPAPSGTARAAAPPVLPAPGSMPRSCPLGPPDAYRHALETPGFSRVTLQYG

RYGTLLTRYEDVRAALRDERIGSALHQLPPVLDGQSPPGWFFGLDGPEHARYRRVLAKMF

SVTRTRRFEPVIRAITDELLDALEPHATGADRAVDLMAGLAWPLAVRVPCALLGISGDDQ

ATFKRQVDTLIAPGVTREEITEVYRAMWWRMRGLVDAERSGTGGQGLFAELVADQSGPDA

FDDDEIASIGLSLRIAGLDPIAHLLGLGLFALLCHPEQLALVRAAGADAVEELLRFVPFN

NMGAVRVAVQDTVVDGEPVAQGEVVVASLTAANRDATVFADPDTLDVTRHNAAAHLAFGH

GPHRCLGRHLAGQVVTVVLDRLLGRFPGLRLAVAAERVPPFESAGFYGVAELPVHLT

>CYP285A3(2511684259)SCT

MMLPDRPAHVRPEDIGGVDLVDPFLYSDGDPHSVWHAMRRHDPVRWHPVGERLGFWSVTC

YDDGDFVMRDHTCFTSQRGTLLNLLGTDDPAGGRQMAATDPPKHTRMREPLQRSLTNKAV

ERYRERIRHEVRRILAPAVTGEPYDFAGEMTALPMAVTGTIMGLPQRDWAELTRLTLMSI

APDDPEYMTDGGPRATLEAAHRGLFAYFQDVVGERRRDLGDDLLSTLLTMEIDGRPLGAG

EVLSNCYSLLLGANVTTPYVPSAAMERMVADPALAADWLGHPELVQSGVEEALRWSSPAN

HFMRYAVKDVTVHGVEIPAGDAVVVWLGSANRDERAFADPFRFDIRRRPNRHIAFGAGPH

YCVGHTVARVSLRVLFEELIGRFEGFEQAGPAEHLCSNFVAGIKHLPVTARVRAGQERVF

AVAAAS

>CYP1061A1(2511684271)SCT

MDNEIVLTRYADALDAYRRKELGQAQYDEGHVLMAGVLVNLHGTEHRDRRRVENQLFRRE

TFLHYEKEVFPDVTAAVLAPHARRGATELVALGHELMLHLAVLVAGVDRPEGTEAETRRL

AGQLKVFIEALTLAQSTLDKDERRAAIADAFTDWRREFLTPSARRRRALLAGLADGTVPE

RQLPRDVLTLLLRHQDRLGLSDELIARETAFFLLVAAHTSATAFVRAVHHLLEWTARHPE

DARRAADDPRFAQRCVHETIRLNPSSTVGMRRALAPVTLESGITVPEGARVVIDLRTVNR

DPAAYGPDAAEFDPHREIAPGVMPFGLSFAAGMHVCIGQDLAAGVLQSGSGPDASLYGLV

ATAVRELFALGVRRDPAAPPVRDTTTTREYWARYPVLLG

>CYP107AM6(2511684330)SCT

MSEVQDAETLRYPLAGPAALEPPEEWARLWEGCPVARVTLPSGDEATLLTRYADVRLALS

DPRLSREGLQRPDAARVAADDSGGVFTGEMARALNDEGHERWRRMVGKWFTAKRMVALRP

GIEETAERLIDAMVAPGGPADLVAHLAFPLPVLVICDMLGVPDGDRDDFKHWSDAFLNLT

RYTRDELATAQREFTAYMAALLAAKRTRPGDDLLSRLLTSTDADGAPMSERALVATGQAL

LLAGHETTAGFIAKTVAHLLADRRRWERLLADPALVRTAVEEALRFDPNNGFGMLRYVHE

DVEIPSGTLTRGTTVVCSMQAANRDETAFADAGGMDLGRTPNPHLTFGSGAHSCLGQPLA

RTELQAVLHVLLRRLPTLELATDPGALRGVEGLLTTPLRELPVRW

>CYP107P12(2511684341)SCT

MEATAVFDPWSPEFVADPYPGFARLREAGPVHWFEPSRQWLVPRHADVRALLRDRRLGRT

YLHRFSHEEFGRTPPPPEHEPFHVLNGHGLLDLEPPDHTRIRRLVSTAFTPRTVEELAPT

VRRLARERVDALLAEGGGDLMATVAEPLPVAVIGEMLGIPAADRGLLRPWSADICRMYEL

NPDEESARRAVRASVEFSDYLRGLIAHRRTHPGDDLISALIAAHDEGNRLTGQEMVSTCV

LLLNAGHEATVKTVGNGWWALFRDPGQLAALRADHTLLPGAVEELMRYDTPLQLFERWVL

DDIEIGGTRIPRGSEVALLFGSANHDPAAFAAPDRLDLTRADNPHVSLGAGIHYCLGAPL

ARLELAAVFGELLRRAPRMRLLAEPEREGGFVMRGVRGLLVEC

>CYP105AC4(2511684438)SCT

MTSCPVDHAAQSEPVTLPLERPAGCPFDPPAELGRLREQHPLTPMTYPDGHVGWLATGHA

VIRAILADPRFSSRYEILHYPFPGGPTSELPPAPVGDLTGLDAPEHTRYRRLLTGKFTVR

RMRLLTERVEEIVTEHLDAMERSGGPLDLVAAYAQPVPALMICELLGVPYSDRDLFQRHV

ATVNDQNATMADQGAAITAIHEYVHTLVVAKRARPTDDLLGDLTRETELSDGELAGIGTF

LLGAGLDTTANMLSLGAFALLSDPAQLAALRAEPELADPAVEELMRYLSIAHTGARTALE

DVELAGRLIKAGDTVTLSVQAANRDPERFPDPDTLDLRRKATGHLGFGHGIHQCLGQQLA

RVEMRVAIPALFRRFPTLRLAVPAEEVPLRTDSSIYGVHRLPVTWERG

>CYP105BE1(2511684440)SCT

MVDRTDPAAPPGMPRVRRCPYDPAPEYARLREEAPVSRISFPGGGTGWLVTRYQDVRAML

ADPRFSSRQGTTAPQVRPVPAALPEPPPGALLRLDEPEHRTYRRPVMRAFTVKQVTRLRP

RIQRITDDHLDAMERDGGPVDLVAALALPVTSLVICELLGVSYADRGAFQGLAGRLLAVD

ISPEQSAQDRAALAAFMTDLVAAKRRDPGDDLISALIAEADADPDSPLTDHALTILGALL

LIAGHETTANMISLGALTLLDHPDQLAAWRARPELADRAVEELLRYLTIIQFGLARVATE

EVTLGGRTIRAGEMVVAALPSANRDPLMGLADPDRLDITRSPAPHLAFGFGPHQCLGQQL

ARAELQITLGSLFARFPTLRTAVPTAELPFRDDMIIYGLHALPVTW

>CYP105AK5(2511684448)SCT

MANLRKKGFDLSKALPESTLMPLRRDGLDPVPDLGAMRRKAPVTKLKVPLGVNVWLVTGY

EEAKAVLGDAGSFSNDFTNLIGTGVTSGTSPGGLGFADPPDHTRLRRLLTPEFTMRRLSR

LTPRIHTIVEERLAEMAAATGPVDLVEAFAMPIPSLVICELLGVPYEDREEFQRLGTSRF

DVFSGADASFGAISESLSYLRDIVKKERENPGDGLLGMLVREHGDAIDDEELAGLADGVL

TGGLETTASMLALGTLLMLQDRQHFTALLADDAAIDPFVDELLRYLTVVQVAFPRFARRD

LEIGGQAIGSGDIVLCSLSGADRDEHLGPHMEEFDPTRPAAGSHLAFGHGIHRCIGAELA

KMELRAAYPALVRRFPEMTLAVPAEELRFRKYSVVFGMESLPVNLG

>CYP147F12(2511684610)SCT

MPSSSPVQRITDYANRADPYPVYAGLRHTPVVRDEETGIFLVTTYWAVRELLHDPRISSD

ARNLAPGANPLLASDTEESTLPPSFIRLDPPDHDRLRRLAMRPFGPPHSPRRIHDMRGEL

SRIVTGLVDGLAGRGEFDVVDDFAYPFPVTVICRLLGVPKEDEPRFRGLVDAVVAGLSPA

EDAAEQRRSVRRSRLELGQYLAGLIEEHQRHPGDDMLSQLATDEGPEGRLSLVELISTAV

LLLIAGHETTVNLITNGMLTLLRHPEELRRLREDPGTAPGLVEELLRYEPPVHVLPQRTT

LAEIELAGTVIPKGAAVWLLLASANRDAERFADPDRFDPARPDNQHLGFGFGVHACFGAP

LARLEAQTALSELVRRLENPRLAEDPPPYRPSPVLRGPRHLRVAFDGLAG

>CYP1005B3(2511684939)SCT

MEHTSRTGPAGAGCPASGLFTAAFTADPWPVYARLRRTAPVHRDPATGLWLVSRYHDVRR

VLLDPVRFRPDNTLDAVVRLSVPALRELAAAGFDLPPTLANNGSPGHAGLRRLVGRLLSG

GRVAAAVPLVTRLAERHLDRVEAELAAHGRCDLVAALTRDLPFAVMLEVLGLRADVEADD

GVDLATLARWNDASLELFWGFPERSRQPRLARLAAGFHRWLGTLADRAEQARPGLLGLLA

GHRHPDGTPLDRREIVAVCYFMVIAGQATTGQMLATMLLRALHDRVLWPRLGREPGLAAL

WAEEMLRREPPLTTWRRITAGPAEIGGVQLPPGAPLLLLLAATGSDPVVFDAPEELRPGR

PRGREHLSFGIGRHRCPGAALARMEAEVVLRSVSARLPTLRPVRPPGETPLLGLLSFRAP

TEVLVERS

>CYP105AC5(2511684958)SCT

MRTDPPTPPTAVPGPFDPPVTAGRGDPREPVFRTRYPDGTSGWVAVGYPAIRAILADPRF

SARSELRRNPYQDAPAEPARPGMFIAMDPPDHTRYRRLLTGQFTVRRMRRLTDRVERIAE

EHLDRLAAAGPPADLVPAYTAPVPALVICELLGVPAGYRDHFQREVARMSRRDITAPDRR

RAVDDIAAYLRDLVRDKRAAPTDDVLGGLVADAGHLTDEELANLAFLLLGAGFDTTANVL

ALGAFALLTHPGQIPAITDPARIDAAVEELLRYLPVVPGTVRVALEDVEVAGVRIAAGET

VLLSLPGGNRDPGRYADPETLDLGRAAGGHLAFGHGVHQCLGQQLARVELRVAFPALFRR

FPGLRLAVPPEQVPLRSDMVIHGVHALPVTWTGG

>CYP161E1(2511685007)SCT

MERLPFPPADDVLAIAPRYRELQDDEPVAPVHTAVGDPAWLVTRHADVTALFGDERLGRS

HPEPERAARVSASVLLGGPVGDHDTERARHRRMRALLTPAFSARRMRALQPRIAHLVDRL

LDELPAPPADWHDVFSVPLPVMVICELLGVPYADHPRFRRWATELTSLTDPDLAGAARDQ

LVGYVRDLIPDKRRTPGEDVISDLVAAQREAALTDDDIADLSAMLLFAGHETTVTRIDLG

ILLLLDHPEALAALRADPALADGAVEEILRLSALTTTGGLPRYAHDDIAVGGTTLPAGSA

VLLATHAANRDHRVFPDPDAFDITRRPNPHLSFGHGFHYCVGASLARIELREVFSRVPAR

LPGLRLAVPRDAVRLRHDRLTGGLAALPVTW

>CYP105BF1(2511685113)SCT

MISDLAPFPWTRHPLDPPDLYARLREERPVTRVELRGGQPVWLITRYDDVRAILADPRAS

ADLSREGFPRFGFRPPNAHERPFLRMDPPEHTVFRRLLAKCFLTKRMLALRPRIQQLVDE

TVDAMLAAPDHRADLVRDLALPVPSTVLSWILGVPAGDREFFNTETQALLDRENTDNKDA

RERALRAGKALRGYLDGLIADREALTDPGEDILGVLVTAVREGTISRQDAINTAVVLIVA

GHDTTANMAALGTLLLLRHDDQRQLLTEQPELMPQAVEEMLRFLTVVHLVVLRVATEDIE

IGGTVIPAGEGIIPLNFSANRDDAHYPDADRFDVHRKARDHVAFGYGVHQCLGQPLARVE

LEVVFGTLLRRLPGLRLAVPFDDLPFKSHAQINGVAALPVTW

>CYP105BF1(2511975541)SCY

MISDLAPFPWTRHPLDPPDLYARLREERPVTRVELRGGQPVWLITRYDDVRAILADPRAS

ADLSREGFPRFGFRPPNAHERPFLRMDPPEHTVFRRLLAKCFLTKRMLALRPRIQQLVDE

TVDAMLAAPDHRADLVRDLALPVPSTVLSWILGVPAGDREFFNTETQALLDRENTDNKDA

RERALRAGKALRGYLDGLIADREALTDPGEDILGVLVTAVREGTISRQDAINTAVVLIVA

GHDTTANMAALGTLLLLRHDDQRQLLTEQPELMPQAVEEMLRFLTVVHLVVLRVATEDIE

IGGTVIPAGEGIIPLNFSANRDDAHYPDADRFDVHRKARDHVAFGYGVHQCLGQPLARVE

LEVVFGTLLRRLPGLRLAVPFDDLPFKSHAQINGVAALPVTW

>CYP161E1(2511975643)SCY

MERLPFPPADDVLAIAPRYRELQDDEPVAPVHTAVGDPAWLVTRHADVTALFGDERLGRS

HPEPERAARVSASVLLGGPVGDHDTERARHRRMRALLTPAFSARRMRALQPRIAHLVDRL

LDELPAPPADWHDVFSVPLPVMVICELLGVPYADHPRFRRWATELTSLTDPDLAGAARDQ

LVGYVRDLIPDKRRTPGEDVISDLVAAQREAALTDDDIADLSAMLLFAGHETTVTRIDLG

ILLLLDHPEALAALRADPALADGAVEEILRLSALTTTGGLPRYAHDDIAVGGTTLPAGSA

VLLATHAANRDHRVFPDPDAFDITRRPNPHLSFGHGFHYCVGASLARIELREVFSRVPAR

LPGLRLAVPRDAVRLRHDRLTGGLAALPVTW

>CYP105AC5(2511975690)SCY

MRTDPPTPPTAVPGPFDPPVTAGRGDPREPVFRTRYPDGTSGWVAVGYPAIRAILADPRF

SARSELRRNPYQDAPAEPARPGMFIAMDPPDHTRYRRLLTGQFTVRRMRRLTDRVERIAE

EHLDRLAAAGPPADLVPAYTAPVPALVICELLGVPAGYRDHFQREVARMSRRDITAPDRR

RAVDDIAAYLRDLVRDKRAAPTDDVLGGLVADAGHLTDEELANLAFLLLGAGFDTTANVL

ALGAFALLTHPGQIPAITDPARIDAAVEELLRYLPVVPGTVRVALEDVEVAGVRIAAGET

VLLSLPGGNRDPGRYADPETLDLGRAAGGHLAFGHGVHQCLGQQLARVELRVAFPALFRR

FPGLRLAVPPEQVPLRSDMVIHGVHALPVTWTGG

>CYP1005B3(2511975710)SCY

MRGRRIVEHTSRTGPAGAGCPASGLFTAAFTADPWPVYARLRRTAPVHRDPATGLWLVSR

YHDVRRVLLDPVRFRPDNTLDAVVRLSVPALRELAAAGFDLPPTLANNGSPGHAGLRRLV

GRLLSGGRVAAAVPLVTRLAERHLDRVEAELAAHGRCDLVAALTRDLPFAVMLEVLGLRA

DVEADDGVDLATLARWNDASLELFWGFPERSRQPRLARLAAGFHRWLGTLADRAEQARPG

LLGLLAGHRHPDGTPLDRREIVAVCYFMVIAGQATTGQMLATMLLRALHDRVLWPRLGRE

PGLAALWAEEMLRREPPLTTWRRITAGPAEIGGVQLPPGAPLLLLLAATGSDPVVFDAPE

ELRPGRPRGREHLSFGIGRHRCPGAALARMEAEVVLRSVSARLPTLRPVRPPGETPLLGL

LSFRAPTEVLVERS

>CYP147F12(2511976051)SCY

MPSSSPVQRITDYANRADPYPVYAGLRHTPVVRDEETGIFLVTTYWAVRELLHDPRISSD

ARNLAPGANPLLASDTEESTLPPSFIRLDPPDHDRLRRLAMRPFGPPHSPRRIHDMRGEL

SRIVTGLVDGLAGRGEFDVVDDFAYPFPVTVICRLLGVPKEDEPRFRGLVDAVVAGLSPA

EDAAEQRRSVRRSRLELGQYLAGLIEEHQRHPGDDMLSQLATDEGPEGRLSLVELISTAV

LLLIAGHETTVNLITNGMLTLLRHPEELRRLREDPGTAPGLVEELLRYEPPVHVLPQRTT

LAEIELAGTVIPKGAAVWLLLASANRDAERFADPDRFDPARPDNQHLGFGFGVHACFGAP

LARLEAQTALSELVRRLENPRLAEDPPPYRPSPVLRGPRHLRVAFDGLAG

>CYP105AK5(2511976216)SCY

MANLRKKGFDLSKALPESTLMPLRRDGLDPVPDLGAMRRKAPVTKLKVPLGVNVWLVTGY

EEAKAVLGDAGSFSNDFTNLIGTGVTSGTSPGGLGFADPPDHTRLRRLLTPEFTMRRLSR

LTPRIHTIVEERLAEMAAATGPVDLVEAFAMPIPSLVICELLGVPYEDREEFQRLGTSRF

DVFSGADASFGAISESLSYLRDIVKKERENPGDGLLGMLVREHGDAIDDEELAGLADGVL

TGGLETTASMLALGTLLMLQDRQHFTALLADDAAIDPFVDELLRYLTVVQVAFPRFARRD

LEIGGQAIGSGDIVLCSLSGADRDEHLGPHMEEFDPTRPAAGSHLAFGHGIHRCIGAELA

KMELRAAYPALVRRFPEMTLAVPAEELRFRKYSVVFGMESLPVNLG

>CYP105BE1(2511976224)SCY

MRRCPYDPAPEYARLREEAPVSRISFPGGGTGWLVTRYQDVRAMLADPRFSSRQGTTAPQ

VRPVPAALPEPPPGALLRLDEPEHRTYRRPVMRAFTVKQVTRLRPRIQRITDDHLDAMER

DGGPVDLVAALALPVTSLVICELLGVSYADRGAFQGLAGRLLAVDISPEQSAQDRAALAA

FMTDLVAAKRRDPGDDLISALIAEADADPDSPLTDHALTILGALLLIAGHETTANMISLG

ALTLLDHPDQLAAWRARPELADRAVEELLRYLTIIQFGLARVATEEVTLGGRTIRAGEMV

VAALPSANRDPLMGLADPDRLDITRSPAPHLAFGFGPHQCLGQQLARAELQITLGSLFAR

FPTLRTAVPTAELPFRDDMIIYGLHALPVTW

>CYP105AC4(2511976226)SCY

MPEFATPERRATSMTVPPAITRTTGLPPADTPPETAMTSCPVDHAAQSEPVTLPLERPAG

CPFDPPAELGRLREQHPLTPMTYPDGHVGWLATGHAVIRAILADPRFSSRYEILHYPFPG

GPTSELPPAPVGDLTGLDAPEHTRYRRLLTGKFTVRRMRLLTERVEEIVTEHLDAMERSG

GPLDLVAAYAQPVPALMICELLGVPYSDRDLFQRHVATVNDQNATMADQGAAITAIHEYV

HTLVVAKRARPTDDLLGDLTRETELSDGELAGIGTFLLGAGLDTTANMLSLGAFALLSDP

AQLAALRAEPELADPAVEELMRYLSIAHTGARTALEDVELAGRLIKAGDTVTLSVQAANR

DPERFPDPDTLDLRRKATGHLGFGHGIHQCLGQQLARVEMRVAIPALFRRFPTLRLAVPA

EEVPLRTDSSIYGVHRLPVTWERG

>CYP107P12(2511976324)SCY

MEATAVFDPWSPEFVADPYPGFARLREAGPVHWFEPSRQWLVPRHADVRALLRDRRLGRT

YLHRFSHEEFGRTPPPPEHEPFHVLNGHGLLDLEPPDHTRIRRLVSTAFTPRTVEELAPT

VRRLARERVDALLAEGGGDLMATVAEPLPVAVIGEMLGIPAADRGLLRPWSADICRMYEL

NPDEESARRAVRASVEFSDYLRGLIAHRRTHPGDDLISALIAAHDEGNRLTGQEMVSTCV

LLLNAGHEATVKTVGNGWWALFRDPGQLAALRADHTLLPGAVEELMRYDTPLQLFERWVL

DDIEIGGTRIPRGSEVALLFGSANHDPAAFAAPDRLDLTRADNPHVSLGAGIHYCLGAPL

ARLELAAVFGELLRRAPRMRLLAEPEREGGFVMRGVRGLLVEC

>CYP107AM6(2511976334)SCY

MSEVQDAETLRYPLAGPAALEPPEEWARLWEGCPVARVTLPSGDEATLLTRYADVRLALS

DPRLSREGLQRPDAARVAADDSGGVFTGEMARALNDEGHERWRRMVGKWFTAKRMVALRP

GIEETAERLIDAMVAPGGPADLVAHLAFPLPVLVICDMLGVPDGDRDDFKHWSDAFLNLT

RYTRDELATAQREFTAYMAALLAAKRTRPGDDLLSRLLTSTDADGAPMSERALVATGQAL

LLAGHETTAGFIAKTVAHLLADRRRWERLLADPALVRTAVEEALRFDPNNGFGMLRYVHE

DVEIPSGTLTRGTTVVCSMQAANRDETAFADAGGMDLGRTPNPHLTFGSGAHSCLGQPLA

RTELQAVLHVLLRRLPTLELATDPGALRGVEGLLTTPLRELPVRW

>CYP1061A1(2511976397)SCY

MDNEIVLTRYADALDAYRRKELGQAQYDEGHVLMAGVLVNLHGTEHRDRRRVENQLFRRE

TFLHYEKEVFPDVTAAVLAPHARRGATELVALGHELMLHLAVLVAGVDRPEGTEAETRRL

AGQLKVFIEALTLAQSTLDKDERRAAIADAFTDWRREFLTPSARRRRALLAGLADGTVPE

RQLPRDVLTLLLRHQDRLGLSDELIARETAFFLLVAAHTSATAFVRAVHHLLEWTARHPE

DARRAADDPRFAQRCVHETIRLNPSSTVGMRRALAPVTLESGITVPEGARVVIDLRTVNR

DPAAYGPDAAEFDPHREIAPGVMPFGLSFAAGMHVCIGQDLAAGVLQSGSGPDASLYGLV

ATAVRELFALGVRRDPAAPPVRDTTTTREYWARYPVLLG

>CYP285A3(2511976409)SCY

MMLPDRPAHVRPEDIGGVDLVDPFLYSDGDPHSVWHAMRRHDPVRWHPVGERLGFWSVTC

YDDGDFVMRDHTCFTSQRGTLLNLLGTDDPAGGRQMAATDPPKHTRMREPLQRSLTNKAV

ERYRERIRHEVRRILAPAVTGEPYDFAGEMTALPMAVTGTIMGLPQRDWAELTRLTLMSI

APDDPEYMTDGGPRATLEAAHRGLFAYFQDVVGERRRDLGDDLLSTLLTMEIDGRPLGAG

EVLSNCYSLLLGANVTTPYVPSAAMERMVADPALAADWLGHPELVQSGVEEALRWSSPAN

HFMRYAVKDVTVHGVEIPAGDAVVVWLGSANRDERAFADPFRFDIRRRPNRHIAFGAGPH

YCVGHTVARVSLRVLFEELIGRFEGFEQAGPAEHLCSNFVAGIKHLPVTARVRAGQERVF

AVAAAS

>CYP105BD1(2511976540)SCY

MLPAPGSMPRSCPLGPPDAYRHALETPGFSRVTLQYGRYGTLLTRYEDVRAALRDERIGS

ALHQLPPVLDGQSPPGWFFGLDGPEHARYRRVLAKMFSVTRTRRFEPVIRAITDELLDAL

EPHATGADRAVDLMAGLAWPLAVRVPCALLGISGDDQATFKRQVDTLIAPGVTREEITEV

YRAMWWRMRGLVDAERSGTGGQGLFAELVADQSGPDAFDDDEIASIGLSLRIAGLDPIAH

LLGLGLFALLCHPEQLALVRAAGADAVEELLRFVPFNNMGAVRVAVQDTVVDGEPVAQGE

VVVASLTAANRDATVFADPDTLDVTRHNAAAHLAFGHGPHRCLGRHLAGQVVTVVLDRLL

GRFPGLRLAVAAERVPPFESAGFYGVAELPVHLT

>CYP1035A7(2511976547)SCY

MLGRLRSAQGLADPAPLYDELRSLGDVVPAPWGGYFVTGFQTCSQVLRSRTWLVPDFAWQ

ERQPDPARWQAPATREMTGTLSRLNPPVHTCQRRSLGNLFDRATLTALTPHVERHVAGLL

DGLERRLRADGTADLVTEVSERLPLLTVGGWLGIPVADHRHVLQFTHNQVHAQELLPRKS

ELEQSARATVQLRAYFTDLVRQRRRAPGDDPVSHWIRTWDAMEPDRAAVDEIVYRLTMFI

TIASLETTATLLSSMVWHLLRRPARWDWLRENLQHVADAVEETLRYDPPVRLNSRVAAED

TELAGVPIRKDEMVHVMYGAANHDPRRNPDPHVFDILRRGIHLTFGGGAHYCLGAALGRL

EARTLLTRMLQRFPALRAVAPPEYAPRMVFHRLTSLRVTA

>CYP156B12(2511976548)SCY

MGCPAHHTTQAARMPLYGEEFAADPGAVYDRLRAYGPAAPVELAPGVEATLVTEHESALR

VLQSPALFARDSRRWKALAEGRVALDSPVLPMMVYRPNCLFTDGTEHLRLRKAVTDSLGR

LNVNRLGRDVERIADYLLDQFSERGMADLLNDYAKLLPLLLFNQLFGCPAGIGDRLTANM

AAIFDGQDVMRANEELTACLMELVALKRRQPGNDITSWLVEHPSGLNDEELKDQLVMLMG

AGVEPERNLIGNALLLLLSADSSTGRSGMRVEDAIDQVLWNNPPIANYATHFPVQDVELG

GVPLPADTPVVISFAGANTDPALTEAHQVLSKGAHLAWGAGPHACPAKDPAQLIALTAIE

RLLNALPDLTLAVPQGSLRWRPGPFHRALVALPVRFSPTPGLRTAAARPVTASQPATVPP

ARTQTASSSGGTAPAKQKGWWSSFLDVFRL

>CYP163D1(2511976854)SCY

MNTVTGVTTFPDLTDPAFWARDDSHAVLRELRRRSPLWRLESEAEGPLWCVLSHALANEV

LGDAARFSSERGSLLGTGRDRAPAGAGKMMALTDPPRHRDLRGLVLPFFSKRKAAELGAR

VADLTRQVVRDALGTARTDFVRDISTTVPLTVMCDLLGVPDEDRDHVVAMCDRAFLGDTP

EERSEAHQQLLPYLFALGLRRRTDPRDDIISQLVTHEVDGRRLPLDEALLNCDNILVGGV

QTVRHTSTMAMLALTRHPHAWQAMRADGYDPETGVEELLRWTSVGLHVLRTARHDTELAG

HHIRAGDRVVVWTPAANRDEAEFHHPDDLLLDRTPNRHLAFGWGPHYCIGAPLARVELAS

LFAALTEAAEHVEVLEPPVPNRSIINFGLDALVVRLHPRGAAG

>CYP183L1(2511976940)SCY

MASRTCATPVVPGAFPMVGHAWPLMRRPLRFAQSLSGHGDLVEVRLGPVRAQVPCDPELL

WRVLTDDRLFDKGGPFFDRVRATIGNGVGACPYGEHRRQRRLIQPSFQPARLKGYAAVME

EEAAALTERWDDGGVIDAYRSLFGAALRSVLRTLFATRAGDAVVDRFRTSVETVLQQLTA

RMFVPAPLLRLPLPVNRRFDRAVADLRRGIDDLVAERRRDRTDRGDLLSALMTAQDDENG

TGCGTGLTDTEIHDQVLTMLTAGSDSVTAAVSWALYLLDRNPEALDRLEREVDAVLAGRA

ARGDDVPALAHTGRVITEALRLYPPGWLFTRVTTAETELGGHRLPPGTTVAFCAPAVHRG

RQLYDDPEEFDPDRWLPERARTLPRGAFTAFGGGARKCAGEAYALTECTLLLATIVSRWR

LRPAPGCDVRPVALSTALRPRRLLMETSARRGLPK

>CYP107AS(2511977159)SCY

MPTDPPEHTRPRRLVAGAFTARRADAMRPRVTDVDADLVENS

>CYP1274A1(2511977169)SCY

MNSAVTECPTVDLNMLDPDFVQDPYETMAAWRALGPVVYNSHHDQYMIMSHHNCARVLGD

IRHFNSANVLPEMEKAFGGPTFLGTDGARHQRVRGIWEPGFERRAVEAEWAARIQRVVDV

YVDRFVERLRSGESLDALAEMTRIIPTVVTAQLLGVEEDVYDDFIKWSDATAAVLGARLD

PTPRGQEIVAEGAAAAVALNSYIGGLIERHRALGVSDGSLMGQMVFDDFAPSMPDREILA

NGTMLTVAANESTAQVMAMMLYALGRHPDQRRALVADRSLVPAALEEVHRWSTLTQSVLR

FACSDESQVEGFTIPEGSSVVALLGCANRDPARWDNPDSFDIFRLRKAHIGFGFGPHICL

GIHMARMEMHIWLNRLLDELPEYEIAGDIEFGPDFTLRGLRGIPVKAA

>CYP107CR1(2511977176)SCY

MTDSTKPCASGTGTAEADLLSWDFIQDPYLTYHEMRAGTGPRRLVIKTLSTGLRSWLVTE

YSDVRRLLADPRLSKAAGGAAPIIAKHSTEDVSGAAITSESMLFSDPPQHSRLRRMFSRA

FTMRRTLDLRPRVEELTDELLDAIPTGAEIDLVESVAMAIPIAVIGELLGVPRAAHQDLR

RWNRALTSVDSEPSEKYQAYMASLEYFRTLVAQKASSHDGADDLINAMIDPDNEERFEES

ELLSTIFLMMNAGYETTANLISSSVYALLKYPEQLELLRGDATLIPNAVEEFLRYESPLN

LSTLRYTTEPVEVGDTVIPAGEVVFLALSSANRDPNRFAEPDRLDIRRNAATHLAFGHGI

HHCVGAPLARLEGEIVLARLLDRFSHWEAAEPLERLTWRYTLQFRGLERLPVRLHA

>CYP105D12(2511977338)SCY

MTDALTTQQSQALPFPQARTCPYHPPAGYRDVRQTGPVGQVRLYDGRLVWLVTGHAQARA

LLTDPRLSADRQNPRFPVIAPRLAELIRRVRSPLLGVDEPEHGVQRRMLIPSFTVKRTAA

LRPRIQRVVDEALDAMVAQGPPADLVASFALPVPSTVICLLLGVPYADHEFFEGRSRQLL

RGPRAEDVESARQDLNGYLRDLVRARRSRPGDGLLDELVAEQLEPGHLTEDELVSMALLL

LVAGHETTANMISLGTLTLLEHPEQLAAVRTDPGVIPDTVEELLRFLSIADGMARVATAD

IEVAGVTIRAGDGVFLATSEINRDPGAFPEPDALDVRRGARHHVAFGFGVHQCLGQNLAR

AELEIALTTLFTRLPGLRTTVPAEELPVRAGDVIQGLDVLPVTW

>CYP105B23(2511977350)SCY

MTITHPGSTFSVPAAKSGGCPFDPPPAYQRARDEQPVTRVTLWDGSQCWLVTRHQDIREA

LRDRRLSSEADRPGFPFITPNRRALASDRRGNTSFIRMDDPEHARLRKMLTPDFMIKKTE

TLRPRIQEIVDDFLDRMIAKGAPADLVADFALPIPSLVICLLLGVDYADHEFFQERSRIM

LHNNTTPEQVAEARDDLLDYLGDLAAAKRARPDDSIIGKLAARPELSHDEVASMGLLLLI

AGHETTANMTALGTLALLRNPGQLAALRDDPALAPSAVEELLRYLTIVQSGVARVAKDDL

EIGGETVRAGEGVLFMISAANRDPEAFPWGDDLDITQDARRHLAFGFGVHQCLGQPLARV

ELQVALATLVRRLPGLRLAIPFEDVRFRTDMAIYGVHELPVAW

>CYP1062A1(2511978105)SCY

MHDVVNCPSAPGGLPLIGHTLAVLGRPLSFFESTRTDDPLVRVVIGRLQVYLANDPDLVH

RIQVDTDTFERGRFFEVLAGHFDNPPIASNGPAHRGQRRALKPVFNRPSVRQYTDAIVEE

AEALAAGWRPGRLPDVRQQLSDVVACTVLRCLFSTELSTEDVHDIQRTIYQVARRLLPGT

LLPAAVTQVPTPGNRRLAEAMGRFRVLVERLVREREEHGQGHHDALSALLHARHGATGRP

LTPREIRGEFLVLLFAALETTSTTLAWALYEIATHPRVAARLADEVDAVLRDGPATYDGL

QRMPYLRQVLHETLRLHPPTLFTRRTRHAVTLAGVAVPAGAEVGYSPRAMHRHPGLHRDP

ARFDPSRIGSEGALPQGAFFPFGVGAHRCIGEHLAMTTMAAVVAAVVARWTIRLPPRVRV

RETISSMPHPDSLPLQLTTRPSASRRSARYATARYSHPA

>CYP125A24(2511978487)SCY

MSCPAMPDGVPPAGFDFTDPDVYATRVPLPELAALRATAPVWWNAQPHGVAGFGDDGYWV

VTRHADVKEVSTRPEVFSSAANTAIIRFHEAMTREQIDVQRLIMLNMDPPEHTRVRQIVQ

RGFSPRAIRSLNDALRERAARIVADARRTGSGDFVTDIAVELPLQAIAELIGVPQEDRAR

IFDWSNKMVGYDDPELAITEEIGAQSAAELISYAMNLAAARKECPAKDIVSTLVAAADEG

NLGADEFGFFVLLLAVAGNETTRNAITHGMHAFLTHPEQWELFKRTRPATTADEIVRWAT

PVVSFQRTATRDTELGGARIAKGQRVGVFYSSANHDPEVFDRPEVFDITRDPNPHLGFGG

GGPHFCLGASLARLEIDLIFGALADTVPGIRQVGEPRRLRSSWLNGIKEMRVTYE

>CYP107U13(2511979160)SCY

MHDHEPAAPSPSLFSWEFAADPYPSYAWLRENSPVHRTTLPSGVNAWLVTRYADARQALA

DARLSKNPVHHSERAHAKGKVGIPGERSADLMTHLLNIDPPDHTRLRRLVSKAFTPRRVA

EFAPRVQALTDRLIDGFAGRGSADLIHEFAFPLPIYAICDLLGVPAEDQDDFRDWAGTMI

RHGGGPRGGVARAVKRMRAYLLELIHRKRAALGDDLISGLIRASDHGEHLTENEAAAMAF

ILLFAGFETTVNLIGNGTYALLRDPGQRRVLTDALAAGETAVLDTGVEELLRYDGPVELA

TWRFATEPLTIGGRRIGTGEPVLVVLAAADRDPARFDRPDTLDLARRDNPHLGFGHGIHY

CLGAPLARLEGRTALATLLTRLPDLQLAVPEGELRWRGGLIMRGLRELPVTFTPEPDRSA

DTGANKVN

>CYP107AE6(2511979871)SCY

MSDPTTDPAFLRDPYPTYAALRSKCPVQQVPSGLGGHSSYLVTGFEEAREALADPRLSKD

TAAFFAGKESRRRLHPAVAHNMLATDPPEHTRLRRLVTGSFTTRAVEELRPFIARTTDAL

LDQWPAEGSVDLVAELAVPLPVIVICELLGIPEPDRAEVRRWSGELFASGAPAVIDAASH

SLADYMTGLIANKRSRPGQALLDSLIAARDGDDRLSEEELVSLGVLLLVAGHETTTNFLG

NAVLALLQHPSELQRLRQYPAEITSSLDELLRYDSPISTATFRFTTEAVTLGGIEIPAGK

PVLVAVGAANRDPARWPNPDELDLDRVAAGHLSFGHGIHRCVGAPLAKAEADIALRKLLT

RFPDMQLAVPAEQLTWRRTRLVRGLAALPVLT

>CYP157B11(2511980289)SCY

MTDPTPATGCPAHPDALPLSGPRYQQTPAELYRRMRHEYGPVAPILLDGGVPAWLVLGYR

EVLHVTGNDQLFARDSRRWHAWSRIPPDWPLLPFVGYQPSVLFTEGAEHRRRAGAISEAL

SGVDQFELRAHCERVADELIDAFAGSGEADLMADLAHPLPLLAAIWMVGLPGAATQDLVR

DLTASLDVVEGVDPVAAYQRVQDRIQHLVAQRRSGPTADVTSRMLEHPAGLTDDEVVQDL

ISVIAAAQQPTANWIGNTLRLMLTDDRFAITLSGGRRSVGQALNEVLWEDTPTQNFIGRW

AVRDTQLGGRRIKEGDCLVLGLAAANTDPQVRPDAHAGPGGNHAHMSFSHGEHQCPYPAP

ELAEVIARTAVEVLLDRLPDVVLAVPAERLVWRPSVWMRGLEALPVEFTPSYAVGPAR

>CYP159A9(2511980290)SCY

MTTERHVADILSPEFAADPYPAYRVMRREAPLIWHEPTRSYILSRYEDVARAFKDPVFTT

DNYQWQIEPVHGRTILQMSGREHAVRRALVAPAFRGRELQEKFLPVIERNARELIDAFRT

AGEADLVAQFATRFPVNVIADMLGLDKADHDRFHTWYTSVIAFLGNLSGDPEVAAAGART

REEFAAYLLPVIAERRAHPGDDLLSALCAAEVDGTRMSDEDVKAFCSLLLAAGGETTDKA

IASVFANLLRHPEQLAAVRADRSLIARAFAETLRYTPPVHMIMRQAAEDVEVSGGVIRAG

ATVTCLIGAANRDEERYADPDTFDIFRTDLATDNAFSAAADHLAFALGRHFCVGALLAKA

EVETGVGLLLDAMPDVRLADGHLPVERGVFTRGPESVRVRFTPAAG

>CYP184A4(2511980341)SCY

MSKQAPLVAGHPLLGSLPDLRDDTLGAYLRARRDHGDVVRFEAGPPGLRASLYAVFSADG

VQQILGTEASNFRKENDVYTEIRESIGNGLLTSQDEEYIRQRRMIQPLFTRRRVDAYAGA

VHDEATATAHRWTTASGNVVDVVPEMAEFALRSVTRILFGSDIESAVDVVRRNFPVIGES

VLRRGVAPLRIPRTWPTPANRRTATAQRELYAICDRIIAERTAAGHDDSGDMISLLIGAR

DEDGSALDADSVRDQVLVFLLAGHETTATALAFGLHLLARHPDAQHAARAEVDAVLDGRP

ASAADLERLPYLTRVFKEAMRLYPSVAIMGRRSVADSEVCGFHIPGGSDVYVSPWVTQRH

PDYWENPDAFDPDRFAPDLEAARPRYAWFPFGGGPRSCIGQYFAMLEGIIGLATFLQSYE

FAAVDAEVPLELGMTMRAAGPARVRLTRRPSSGEAAA

>CYP107CS1(2511980668)SCY

MSVTRPSPELSEYDLSDVAFITDPEAGRRWLAGPRPICRGRSFDGSQAWIVTGYEDAKQV

LTDPRFTSRPPGDSHARGLRARGMPEDLVSLFDSMLLSMDRADHDRVRPLVTLAFSARRV

RTMRPFIESLVGELLDAMDPAAENDLVAALADPLPIRVVSELIGVDEVHREQWLRWAQTF

NGPVPPPADQLAPALRGMVEVTRDLIAQRRRRPTDDLISELIRTRDEDGGRLTDDELAAL

AILVIQAGHDSVRQLIALTVLTLLDRPDQLALIRSGRTTWSVALAEVMRHAAPVKHAFRR

FATEPVEVGGVTVAPGEGVLVVLAAANRDPAEFPDPLVLDVTRTPNPHLGFSRGPHFCPG

STLAMTEVEIAMRELFGRFPKLRLAVAGDEVAPRFLLGVQRLPVLLD

>CYP107W2(2511980674)SCY

MTRVRLPDGTQAWLVTRHADVRQVLMDPRFSNRVVAARPEVAESEHGALISQSLIGMDPP

EHTRIRRLVTRAFSARRVERLRPRVAELVDGLIDALEELPRPVDVVRHFAVPLPTTVICE

MLGVPEADRAAFQEWSNALVVDWLRDEGERNAATAALRGYFTELIAAKRAEPGDDLMTEL

IAAREEGNKLSESELVAQCIGLLSAGNDTTASLIAMFLMTLLRRPDELARLRAEPAEIPR

AVEELLRYVPLAMSGAGGPRLTTEEVELGGVTIPPGKLVLPAIAAANRDPEVFADPERLD

LDRTDNQHLGFGAGIHFCLGAQLARVELQEALAGLLRRLPGLRLAVPEEELRMKPASAIS

GLEALPVEW

>CYP1199A4(2511981467)SCY

MAVSATDRRGRVTVFTPRIDQLLRERRGSDLFRLDPGTIGIAGADLIDTLLASRPANENE

RPTFKPLQGRSISRTEAATVMQAVSHDVKAALKKPAPNNIDLSGEWPHVGHVYLRDMVFG

ADPYRLRVLVDRKLELTTKLTWSVIATGAARPLSPEPSLSRLGGLTTAAGTYNDRRHAMG

LYRRAAAPVCFTVSTLVANALWLGAPFDDDTPNLHILLESMRLLPPSWNILRVASPEFPA

IDARIGATDDILILPLLSHRDPAIWPDPDDFRPDRWADLDGDNQRGYLPFGHANERCWGR

HMVMPLAEHLLDLLRTGGYTVDPAQRSATVPLAGLLGVTGVRVTRT

>CYP107L23(2511981890)SCY

MTTVNDLTTYIDNDADDDILDLRALGPRFRRDPYPVYAGLRARGPVHRVLTTTGTEAWLV

VGYEHGRAALVDPRLSKRWSHASDALRLIAPAPGTHMLNSDPPEHTRLRKLVVKEFTPRR

VESLAPRVQQITDDLLDAMLAAPDGRADLVDAFSFPLPISVICELLGVPFLDRRSFRAWT

DDLLSATDPAQQEATVARVGRYLGELIEEKRRHPGDDLMSALIRTSDEDGDRLSPDELLG

TAWLLLVAGHETTVNLITNAVLALLTHPDQLAALRADPSLVDNAVEETLRWDGPVETPTF

RFTTEPVEIGGTVIPGGGQLVLVALGDADRDPARFPDPERFDITRQPGGHVAFGHGLHYC

LGAPLARLEGRIALRTLLERCPHLALDAHPGELEWRPGLLIRGPYHLPVRFTR

>CYP102G4(2511981937)SCY

MSPTPHSASGTTGAAAATPGAASPAPPVPVADISDTGFGTTPIQQAMALAREHGPVFRRR

FGTFESLLVGSVDAVTELCDDERFVKAVGPVLTNVRQIAGDGLFTAYNDEPNWAKAHDIL

LPAFALSSMHTYHPTMLRVAKRLIAAWDTALADGAPVDVADDMTRMTLDTIGLAGFGYDF

GSFRRGEPHPFVAAMVRGLLHSQALLSRKADDGVDHSAADEAFRADNAYLAQVVDEVIEA

RRASGETGTDDLLGLMLGAPHPSDGTPLDAANIRNQVITFLIAGHETTSGALSFALYYLA

KNPAVLRRAQAEVDALWGDDPDPEPDYTDVGRLTYVRQVLNEALRLWPTAAAFGRQAVTD

TVLDGRVPMRAGDTALVLTPVLHRDPVWGDNVEAFDPERFSPEREAARPVHAFKPFGTGE

RACIGRQFALHEAVMLLGMLIHRYRFLDHADYRLRVRETLTLKPDGFTLKLARRTSADRV

RTVASRAAEGTAGQDAGLPTTARPGTTLTVLHGSNLGACREFAAGLADLGERCGFETTVA

PLDAYRAGDLPRTSPVVVVAASYNGRPTDDAAGFVSWLEQAGPGAADGVRYAVLGVGDRN

WAATYQKVPTLIDERLAECGATRLLERAAADAAGDLAGTVRGFGEALRRALLAEYGDPDS

VGAVAGAEDGYEVTEVTGGPLDALAARHEVVAMTVTETGDLADLTHPLGRSKRFVRLALP

DGATYRTGDHLAVLPANDPALVERAARLLGADPDTVLGVRARRPGRGTLPVDRPVTVREL

LTYHLELSDPATAAQIAVLADRNPCPPEQAELKKLAPGRASVLDLVERYPALTGRLDWPT

VLGTLLPQIRIRHYSVSSSPAVSPGHVDLMVSLLEADGRRGTGSGHLHRVRPGDVVYARV

APCREAFRIAAGDEVPVVMVAAGTGLAPFRGAVADRVALRSAGRELAPALLYFGCDHPEV

DFLHAAELRGAEAAGAVSLRPAFSAAPDGDVRFVQHRIAAEADEVWSLLKGGARVYVCGD

GSRMAPGVREAFTALYASRTGATAEQAAGWLADLVARGRYVEDVYAAG

>CYP158A13(2511982126)SCY

MTTRPHEESRETGRDDAPPVRFWSVDEVEAMDFDPFMEELLQQDSPALVKLPHGTEPAWV

AARYDDVKLVTSDPRFSREALVGRDVTRLAPHFIPLDDAVGFADPPEHTRMRKTVAAMFT

HRRIEKLRPRAEEIAGRLLDTMERAGPPADVMEHLNTPFALGGMSELMGVPEEDWPKMAK

WARLVISAEAGREASEQAKHDIGAYFADLAAQRLAEPRDDVLSHMAAAERDGRLTHQELV

AFAVLMQISGTNSVRFNSSNMVYLLLTHPDHLARLRAERELLPQAVDELLRFVPHRNAVG

MARVAVEDVRLGDVTVRRGDPIYVSYLAANRDPEKFSCPHRMDFDRTFNPHVSFGSGPHY

CVGASLAKMECEVMLGGLLDRFPRLRLAVAPEEIEWRRGELIRGPHALPVTW

>CYP105B24(2511982717)SCY

MTLSHQPLPDLQLPAPRGACPFAPPPAYRQAPIARGTLWDGSTPWIVTRYQDVRAVLADP

RFSSDPHRPGYPFPTPGYRALMRDNPALLGMDSGGYERLQRLVPGYFRGAAMEALRPRVQ

RIADELLEEMAAGGTRADLVTALALPLPSRVICLVLGVPYEDHAFFHRRACLLVDHHGDP

EEIQRAAVELLGYLERLAATLRRAPDGSVVGRLAAQGELSTREIAGLAQLLLLAGYETTT

NMIALSVLALLRNPEQLARLRGRPGLVPGAVDELLRYLTVIQAGVARAATEEVTVAGQLI

RPGEGVLCMVSAANRDAEVFAEPDALEVTRPARRHLAFGYGVRRCLGHRLARIELEVALA

TLWRRLPGLRLAVPFEEIVFRPEVIIYGVAALPVAW

>CYP1038A4(2511982718)SCY

MTAAVPRPPQAPSTPARQAPPGPALIEVLTRAAGHGSPAAFELDGRPMTLVTDPEQVRQV

LACRPEVYVKHSHRARALLGDGLITATGDAWKRQRRLLQARFTVTGVRRYEAGIAAAAER

IARRWSAAAGTGDLVDVGEDMRFFALDTIWRALTGDPLDEAAHRELAAVDTVVAALPTTA

GAPTGDPAEVGAALGRIDATARRVIAAARARRAAHGPSDALLDLLLDASGSDAGDPDRLV

RDELVTLLVAGHETTAQTLAWLFLMLHQNIHVPRTSEPGALVAETLRLYPAVWLVPRCAA

RDDVLGGRRVAAGSGVLVCPYLTHREPAWWPDPERFDPARFLPGGVRPAHPGAYVPFGLG

PRACLGQQFALRETAALLARLLPAFTVELRDPPAAPVFGANLRPGGPLPAVVRRRT

>CYP183M1(2511982721)SCY

MPPNTVPTPVPGGRPLIGHARQLLWRRLPFLESLRDHGDIVVIRLGPWRIHVLNDPALVR

DVLTKRSPDFGLSPQFQVMKRVIGNGLLATDGPFHRRQRKLILPALHHTRIRAYARTMTR

LADARTARWQDGQTLRVDAEFTELATEIVLRCLFSTEIGGADVAAVVAALPDLMSWAGSR

GLDPTGLLGAVPTPLGRRFRRSMAVLDALLARVIGARRADGPATDHPDLLAALLAARDAE

TGEPMSDRQIRDEAMSFLVAGAESVSRTLTWSALLLAGDPEAARRLHQEADRELSGRPAH

FEDLPRLRHTRMVLQEALRLYPPGYLISRAALRDTTLGPYRIPAGATVMFSYYALQRDPR

RFPDPARFDPLRWSPKRGGADREAFTPFGLGPHGCLGESFAWTEMSIVLATLAARWELRS

ASPRPVRPVPTFSLTMAGAPMTVTARPVRTGPVHTLLASRNGG

>CYP107E11(2511982743)SCY

MRLHTAEPAGTADAEPVPYPFNEADGISLADAYEEAREQPGLLRVRMAYGEPAWLATRYA

DARLVLGDRRFSRAEGARHDEPRQSEGRRDSGILSMDPPDHTRLRTLVAKAFTMHQVEKL

RPAVRELADELIDKMVATGAPVDLVEEFALPVPVGVICQLLGVPVEDRPRFRAWSDAALS

TSSLTAEEFDANQEELRAYMRGLIEDHRARPREDLITGLIEARDRDDRLTEQELVDLCVG

ILVAGHETTATQIPNFVVTLLDRPEQWNRLREDPELVPTAVEELMRFVPLGSGASFPRYA

TEDVEVGGTLVRAGEPVLVAVGAANRDPARFDAPQELDLAREGNQHLGFGHGVHHCLGAP

LARLELQEALGALLRRLPGLRIAGDIEWKTQMLVRGPRTLPVGW

>CYP105AA10(2511982828)SCY

MSQALPIPEGLPAERDAGPFDPPRGITRMREARPVMPLIFPDGHEGWLVTGYDAVRQVMA

DTRFSSRLDIGIVHVPYQTPGMPAPTEPSPQIPGMFIAMDPPDHTRLRRKLTGAFTVKRM

KMLEEHIIDITERQLDALARLTPPVDLVKEFALPVPSLVICELLGVPYEDRETFQSNSAQ

FLVKDQTVEEKVGAYNALTTYLAELVTRKRAEPGDDILSDLARHDDLTIEELTGIAFLLL

LAGHETTANMLALGTFALLENPGQLAELRADPGLIPDAVEELMRYLSLADVFYRYATEDI

ELGGETIPKGSTVVVSLLAANRDPHRFDDPDTLDIHRKARGHLSFGHGVHQCLGQQLARI

EMRAGFDGLLRRFPTLHLAIPADQVKLRTDMNIYGVHELPVAWTETPR

>CYP107CT1(2511982875)SCY

MELDQEFYADPHPTYRAIKAQGNRPTPIVLRTGMAYLPPGLRGWLVTAYQDVEFVLRDPR

FRKSIDEAMPLFAAGTGREGGGDRSSLLYDNMANNDPPKHTRLRKPLNATFTARAVATKR

RDMRRVATETLDALAGRDTFDLVQDFAFPFSIAVICDTLGVPREDRGTFHSWVQTITGAA

DHETLRRDTGLMAQYLRDLIGRKRAGTADDVLTQLATSLAEDEAVAQAYALLAAGYETTA

NLIVTGFLTLERNPEQKRRLWSDPSLVPGAVEEMLRHQSPFNLSLYRYVTEDVEVGGVEI

PAGAIVFLSFAAANRDEHRFADPDAFDITTPRREHLAFGGGIHNCIGKHLARLEAEVAFD

ALVRRCPGLSVVTPADRFVWKASPTFRGLKNLAVGPGPRD

>CYP105B25(2511982886)SCY

MTLTSDPAPLPVEPPAGCPFDPPAQFARLRTEEPISRISLADGSWAWLATRYADIRAILG

DPRFSSDTTLPGYPLSGMTGGASTENRGFIRMDPPEHTRLRRMVTREFMVKRVEALRPEV

QRITDELCDEMSRAHAAGEPVDLVEALALPVPSLVISLLLGVPYEDHTIFQRLTGTLLSR

TVSEAERDVARGELRDYLHQLVSAKEAEPGDDILSRLIVEQERPGEITHDDVVAFAALLL

VAGHETTANMIGLSALTLMRDQASADRLRAEPGLIRGAVEELLRYHSIIRNGPRRVATED

VEVGGRLIRAGEGVVAAVPSANRDETVFADPDRLDVCRPNAQHHVAFGYGIHQCLGQALA

RVELQVVIGTLLRRFPGLRPAVPVEEIPFRTDMAIYGCHALPVTW

>CYP154A14(647542511)SCLF

MTAPAPEPADRHAVQPPHVLDPAARDRAAEDAALRARGSVTRVDVLGEEVWAITDPVLLK

RLLLDGRVSKDSRRHWDRFPEHTTDWPLVLWVAVESMFTAYGPEHRRLRRLIAPVFTART

VNALAPDIERFARELLDDLATTPPGGTADLRERFANPLPLRVIGRLMGLPERMVPDFRRV

VDGVFATAVTAAEAAANTRDLYRTVEELIALKRERPGDDLTSRLIAARDTEGDGQGLSEK

ECGEMLLLIISAGYETTVNLIDQAVVALLTHPGERAAARAGTVSWGDVVEETLRWQAPVP

LLPMRYAIEDIELPGGTVVRRGQAILAAYSAANRHPGLHGPTAGEFDPARADKSHLSFGH

GVHVCLGAPLARLEATIALRLLDERFPRLALAVPPGDLVPLPSFLANGHRSVPVVLEPGA

PA

>CYP154V1(647542584)SCLF

MTKRRPPGPAPPARTRATGSGQGIRPCSGALPRAPAAGRAKIDAVRTLSFPDANTHNRKR

QKRPPRRAESTALPSGRCGTGHITSVNGNSCGGLVMTGTGTRVAPALSSDGGERLYSQLH

ELRSAGPAVRVELPEGVVAWSVTRGDVVRQLATHPHLSRDARGIWPNYRPGAVAWLYTWV

DTRSMATSEGQEHKRLRKMIGPVFGPRRLQNLRPSIEATVDGLLQGLAAEDPREPLDIRA

RFAHEVPTRVMCDFFGVPEDQRPFMLKFLGMALQTGGTPESARRTEQGVKTAMRRLIAAK

RADPGDDMTSLLLTEHEGDQLTEDELISTLNLMMGAGTQTTVALLVHAIQELVTHRDQLA

AALADPSRWDHVVEETLRLHPPVVHLPLRFATADIDLGEGAVIAAGDAVILGFGAEGRDP

AIHDRAEEFDLDRADKTHLAFGHGAHYCLGAPLARLEAAVALPALFTRFPRMALADSEAV

PVPHLSFIANDMGGLNVRLDGGGAAG

>CYP105AC6(647542603)SCLF

MHDRLPTETATARGGNGAAPTAGPVPPPALNQRCPFGPSPGLTALRDEQPLRPMRYPDGH

VGWLATGHPAVRKILADPRFSSRLELMHLPFELDFDGIPSAPAGDLTAMDAPEHTRYRRR

LAGAFTVRRMRLLTERVEEITAEHLDAMERQGTSADLVTAFAQPVPTLVICELLGVPYTD

RSRFQGYTAALSGVDVTLEEQIAAMADLSSYIQELITAKRTAPTDDLLSDLVTDGGFTDA

ELAGIGSFLLAAGLDTTANMIAYGTFALLSHPEQLARLRAEPELAAPAVEELLRYLSIAH

TGIRTALEDVEIDGHTIRAGDTVTLSIEAANRDAERFPDPDTLDLGRRTTGHLAFGHGIH

QCLGQQLARVELTVALPALLARFPGLRLAVPADEVPLREGVNVLGGVHGLPVAW

>CYP136F1(647542851)SCLF

MARRTASSSLAPAPSGSALAPVMGDPGLPYIGYALHTMWNPIRHFRRRYDQYGPVSWGNF

LGRPIVSVQGPEAAQVVLGNREKAFASGPAWDYFIGPFFERGIMLLDSDEHLRHRRIMQQ

AFTRERLAGYLEYIDQSARRGIDAWRPGRRTLLPLFRRLTLDMALDVFLALDLDRAGSHR

VERAFEDAVRAGLALVRFPVPGLRWSRGLKARRYLVDFLTGHIPAKRDRGGDDLFAVLCG

ARSEDGERFSDTDVVNHMIFVLMAAHDTSTIALTTMAYYLARHPEWQERCRDRSLALGDG

PVDSAALDALTELDLVLRESLRLCPPVPLLPRIAVRDTEVLGHHIPAGTFVGVTAFSNHR

LPEHWPDPERFDPERFAVARRTEITHPYSWFPFGGGVHKCIGMHFANLQIKAVMHQMLRT

HAWSVPEGYTWRLDMSTLPVPRDGLPVTLRPSPAAVR

>CYP107AL2(647542979)SCLF

MDTASSETATATDAAAELQYPFPRPSAVQVPPVYDRLRGECPVAKVRLPSGDDGYVVSRY

DDVRTVLADPRFSRAAMLAEGAPRLTAAPPMGGSLFTMDPPEHTRLRRLVSREFTARRVQ

NLRPRIQEMTDELLDGMEKLSPPVDLNPAFAFPLPVMVICELLGVPFEDRDRFRGWSDAF

VSLTSHTPEEVMEQRMSMVQYLGELVQRKRAEPTDDLMGALVQVHDEDGGRLSEIELITM

GITLLVAGHETTVSMIGTCALTLLRHPEHLAALKADPGSIDKVVEELLRINPIGDGGPFR

VTLEDVEVADSVIPQGSGVIAAVCSANQDSARFGADPGVFDPSRPTASAHLAFGHGPHFC

LGAALARAELQIALSSLFRRFPGLALADEVRNLRMTSGMMVHALSRLPVTW

>CYP157B12(647543058)SCLF

MTEADFTASDFSDHPDLSGLSGLSDISAFSGPADPADPTDPARPAGAPPTGCPAHLAHPA

GAIRLSGLEYQQTPAELYRSMRREHGPVAPVLLDGDIPAWLVLGYAEVTHVTAHDELFAR

DSRRWNQWPSIPADWPLLPYVGHQPSVLFTEGAEHQRRAGVITQALEGVDQFELAQWCRE

IAHRLIDVFAGSGRAELMASYAHALPMRAALRMCGMPSGDDTEDLVTDLRISLDAHEGDD

PVAAYVRVGERIQRLVRDKRREPGADVTSRMITHPAGLTDEEIVQDLISVIAAAQQPTAN

WICNALRLMLTDDRFALNVSGGRLSVGQALNEVLWLDTPTQNFIGRWAVRDTRLGGRSIR

AGDCLVLGLAAANTDPQIWPEGHVGAENSAHLSFSNGEHRCPYPAPLLADCVSRTAIETL

LERLPDLVLAVEPQELTWRPSIWMRGLTALPVRFTPAGG

>CYP159A10(647543059)SCLF

MATPHHVPDILSPEFAENPYPVYRAMREHTPLLWHEATRSHVLSRHEDVERAFKDKEGLF

TTDNYGWQIEPVHGRTILQLSGREHAVRRALVAPAFRGDELREKFLPVIERNARELIDAF

RYTGATDLVDSFATRFPVNVIADMLGLDKADHARFHRWYSSVVAYLGNLTGDPEVTAAGE

RTRVEFAEYLLPVIRERRRNPGDDLLSTLCLAEVDGVRMSDEDVKAFCSLLLAAGGETTD

KAIGSVFAHLLAHPEQFAAVREDRSLIGRAFAETLRYTPPVQMIMRQTAAEVTVSGGTIP

AGATVICLIGSANRDAARYRDPDTFDIFRDDLDADTAFSAAAGHLAFALGRHFCVGALLA

RAEVETGIGMLLDAMPGLRLADGFVPAEQGVFTRGHPALPVRFDPVTR

>CYP107X8(647543685)SCLF

MTLQDPTRALLADPYAVYDELRATAPVHRIIGPEGLPVWIVTRYDDVRQGLADPRLSLDK

RNALAGNYRGFRLPPVLDAHLLNMDPPDHTRIRKLVTRAFTTRRIEELRKPVRRIADGLL

DTMAERDRADLVAAYAGPLPVIVISELLGVPEPSRRDFREWTDAVLLPDPDHPERAKEAA

GSLVAFFSGLLAHKRERPGDDLTSALIAVRGEDTARGTGRGADRLSEDELMSLVFLILLA

GYENMVQVIGNAVHALLTHPEWWAALRADPGLLPAAAEEFVRFESPAQLAIRRFPVEDVT

IGGTTVPVGETVMLCLGAANRDPDRFPHPERLDLRRDASGHVALGHGIHYCLGAPLARLE

TEVAIGALLERFPRLALDVPPEELRRRPSTRARGLIALPVRY

>CYP107L25(647543766)SCLF

MVRARRRREHATGGGLRPRGSSEKRRSPAARAGYHVRSTSVLTRENRMSTPPAVDLLQLS

PDFERDPFPVYAALRAQGQVHRVRVPRGMDLYLIVGHEACRAAYTEPRFSRDWVGSGHLS

TISEVDPDQPVLTHMLLTDPPVHTRLRRLVTREFTPRRIEALAPRVQRITDDLLDAMLAD

GKREADLVPSFAFPLPMAVICELLGVPALDRTLFSSWSREIVAPMDPAAEKAAYEQMGVY

LLELIAAKRADPGEDLLSGLIHTVDEGGDRLSPQELVGMCAVLLVAGHETTVNLIGNGVR

ALHAHPEQLAELRADWSLLDGAVEEILRYDGPVENSTIRVVLEDVELSGVTIPKGSAVLI

AQADADRDPERFDAPDTFDIHRDSRGHIAFGHGIHHCLGAPLARLEARIAFRSLLERCPR

LEVIKKDRELPWAEGMLIRGVKELPVRW

>CYP125A25-fragment(647544071)SCLF

FTDPDLLSARVPHPEFALARRTAPVCWVPQRPGTTGFDDGGYWAVTRHADVKYVSTRPDL

FSSYLNTAVIRFHESIQRDQIDVRPHRVYRSSGLRGSGGSAVRRRRPAPGGTGQCRSQAV

STSPGSARRTRRRTGCRSGPSYSPHPT

>CYP125A25(647544073)SCLF

MRCPRLPGLPGIAGIPHGFDFTDPDLLSARVPHPEFALARRTAPVCWVPQRPGTTGFDDG

GYWAVTRHADVKYVSTRPDLFSSYLNTAVIRFHESIQRDQIDVQRLIMLNMDPPEHTRIR

QIVQRGFTPRAVNSLEGVLRERARAIVDAAVARSRTGGSFDFVSTLAVELPLQTIAELIG

IPQRDRARIFHWSNRMASYDDPEFAITEEVGLEATMEFMAYAMNLAAARKECPADDIVSR

LVAAEGSGNLSSDEFGFFVILLAVAGNETTRNALTHGMHAFLTHPEQWELYQRERPATTA

EEIVRWASPIVSFQRTATEDLVLGGQEIARGERVGLFYSSANNDPEVFTDPESFDITRDP

NPHLGFGGGGPHFCLGKSLAVMEIDLMFDALADALPDLRLAGEPRRLRSAWLNGVKELQV

TVDR

>CYP107CU2(647544455)SCLF

MQQITVSDYPSAAFTGDPVPLPEEPVAQVRLPSGDTVWLVSGYDEVRIALAHPLLSRDVG

KHGPRAGTGGAMGTSRWDVRTLQNDGAAHGELRRLAARPFTPRRVEGLRRRVQELTDGCL

DAMEESGPPADLLGALAQPLPIAVIMELFAVPERDREDFVRWSDRIVTLFGITEQEVDDA

HAAMRAYLDGLIAERRARPGDDLLSGWLTAQEGGDRLSDEEVNLLAQSVLIAGFETTVSA

IGAGMWRLFQHPEQLAAVRADRGLLRGTVEEILRHQPMGLFFSMMVARGELELGGVTIRA

GEAVMPLPHAANRDPARFADPHRFDIRRANTGHLGLGHGPHSCLGAALARIELEVAIGTL

NRRFPGLRPVDTDLTALAWRGDRLVAGLAELRVTW

>CYP107U14(647544743)SCLF

MSDCPVSASAPGRGPGQEPGKGPGQGPAPELFSWEFASDPYPAYAWLRENSPVHWTKLPS

GVEAWLVTRYDDARQALADQRLSKNPAHHDEPAHAKGKTGIPGERQAELMTHLLNIDPPD

HTRLRRLVSKAFTPRRVAAFAPRVQELTDRLIDGFAERGEADLIHEFAFPLPIYAICDLL

GVPPEDQDQFRDWAGMMIRHGGGPRGGVARSVKKMRGYLLELIHRKRASLTDEGADDLIS

GLIRASDHGEHLTENEAAAMAFILLFAGFETTVNLIGNGTFALLRHPEQRERLQRSLAAG

ESGLLETGVEELLRHDGPVEMATWRFATEPLVIGGQRIAAGDPVLVVLAAADRDPERFGE

PDVLDLARRDNQHLGYGHGIHYCLGAPLARLEAQTALATLFTRLPELRLSVDPSELRWRG

GLIMRGLRTLPVTFSPAPRHAEIDGVSGS

>CYP161F1P(647544976)SCLF

MISDLAALGELPEEEIVALTAQLIFAGHSTAVRHIVLGVLRLLRHPEQYTALGADPSLVP

GAVEEMLRMSVPSDHGLVRYAHTDLTVDRVTIATSDAVLLFHRVANRDPAAFPDLDRFDT

TRRPDHPSLAFGYATRLCVGAQLARAQLQSILTRLPHRFPALHLAVAPDTLRPTPSRITG

GLEELPVTWRSGPTPQWRGAPLRTL

>CYP1063A1(647545138)SCLF

MSAFPIKSISGVEDSLADFVFDPLDAEFLENPYPVLRALRQDHPAHWHEGMRSWIFSRYD

DCREILHDTERFGADPRAVGQELPPARVSIQTLDGEEHARIQRVVVAALQEADFTLVERR

MARLLRRPPGSGADTVDFVQDIALPVTTRATLSLFGLPADAEERIADSSTVIVRSMMHGL

LTEGETDALAARAAVTDILDTWYGRTGDGLLGAIHRRPEAAALDRTALLNSLRVVLLAGI

NSTQRLLSLAVRTLLARPRGLKEFRAAPSGNRAVHELIRYEGSAQSAARFCRETTTLHGR

RVRRGEQVVALLGSANRDERRFADPDGLDLDRHPNPHLGFGRGTHACLGIPLTLSIARGT

LETLGRDHPGAALAGPVVIEPNPALRGLTSLPVRLR

>CYP107P13(647545169)SCLF

MSALFEPWSPAFVADPYPAYQRLRETGRVHWFEPTRQWLVPHHEDVSALLRDRRLGRTYL

HRFTHEEFGRTAPPAAHEPFHTLNDHGILDLEAPDHTRIRRLVTKAFTPRTVSALEPTVR

RLAAELVDGVKRAGGGDLLADVAEPLPVAVIAEMLGIPESDRALLRPWSADICGMYELNP

SEETARRAVTASLEFSAYLRELIAERRTRPGDDLITALIAAHDEGDRLSEQEMISTCVLL

LNAGHEATVNTTANGWWTLFRHPDQLAALRADHTLLPTAIEELMRYDTPLQMFERWVLDD

IEIDGTVIPRGSELALLFGAANRDPARFPSPDTLDLTRADNPHVTFGAGIHFCLGAPLAR

IELAASFGELLRETPTLRLAAEPQWKPGYVIRGVHALPVEL

>CYP102G10(647545282)SCLF

MNPSTALRRSTRATGRRIGTAPGAGSVPERTRLPLFGHALSVPAGGFTEHTLREARALGP

LFNLRFFDTDCWMVSGPELVAELCDETRFRKSVQPIAAVREFAGDGLFTAFGDEPNWRKA

HNILMPAFSYNALRAYHPTMVTVARRVLDAWDRNAGTPVDVPEDMTRLTLDTIGLCGFGH

DFECFERTTPHPFITAMVSALDHAQRKDTFIPGLDFLRRGAEARQRINIETMNRLADDIV

RRRRAASDGGADSADDLLGLMLHAEDKDTGQPLDDMNIRYQMLTFLIAGHETTSGALSFA

LYYLLKNPSVLAAARAETDALWGTDPDPDPTYEDIGRLHYLRQVLNESLRLWPTAPAFAV

EPLEDTVIGGRYPVHKGQPLTVLTPVLHRDPAWGDNPELFDPARFDPEQADSRPGHLYKP

FGNGERSCIGRQFALHEATLMLGLIVHRYRLIDHADYELKIKETLTIKPDGFTLVPVRRE

ESERRRAATVSRETAERAGAMGPSGTTGGPGATGTAVPAGLRAPGTTLTVLHGSNLGTSR

TLAADLADQGARHGFRTSLAPLDDAVGALAPGQPVLIVASSYNGRPTDDACRFVEWLESG

AAPADVPYAVLGIGDRNWAATYQRVPTLIDEQLTAAGSTPLVERAAVDVSADVATEVERW

SGRVWGALLDRYGVPVDPAEAADAERPAVPTGPRHTVTDTDAAPGLPVPDGLVPLTVVET

GELADLDHPLGRSKRFLRLDLAHGMEYRTADHLLVRPANPAALVDRTAAVLGLDPERTVV

LGAAPGATGRGPSLPTGEPMTVRTLLTHLVELGRPATARQARALAAHAPCPPERATLERH

ADGPTDGPTAATATVTDLLERNASCRPGLADLLDILVPMNLRYYSVSSSPALAPGSADLM

VAAAPVPHRSGEGTFLGTGAAYMADLAPGDTVHGRISPCRDAFRLPDDPSVPAILVSAGT

GLAPFRAEIADRAALPAGTDPAPLVSYFGCDHPDVDYLHRAELEAAEATGAVSLRPAFSR

SPVDGARYVQHRIAADSAELWDLLRSGAHIRVCGDGRRMAPAVRDAFRAIHREHTGGDEE

SADAWLSALMAEDRYVEDVWAG

>CYP156H2(647545939)SCLF

MVPVSAPAGWAPQPTGTEPIRLWEDGFAADPHRYYERLRAQGPIGWAELAPGIPAYIVVD

RRAALDLLHDPETWSHDPRAWEATITEDSPILGMMRWRPNTLFADGDTHVRYRRTLVDAF

SRIEPHDLRERVHRAVDILVSRFGPRGSADLVVEFCRPLMGLIFNNLFGLPDSESDRLTD

SMAQIVEIAESSAQAEAEYGAYVLELIATKMARRGHDLTSWLLDHPLGLTPEEVTWQVFY

TLGAGHEPTANLVSNALSRILGNPAYYTTLTSGSRPVLDAVVEVLRYETPLANYGIHYAR

QPAGLHGVWIQTAVPVVISYGALAYFAEKGSEGAEHPSDASHLSWSAGPHTCPVKQHTLL

IATEAIERLTQWLPDLSPVIPRNRLTWRPGPFHRSLTEFPVRFTPRTPDQSGDRG

>CYP154U2(647545940)SCLF

MSESMSAAPVPPVPPVLPPPPAAPAPPLPPVAPVAIDPFGADIPAEWERLRALGPIVPVE

LPGGIPAWAPTRYRTLRELILDPRVSKDPRKHWARLPEVEHRPEWGWVMSWLGVINVLTA

YGPDHTRLRKLVAPSFTARRTEALRPRVGAITETLLTALGEAAAEPGADGVVDLKDGFTQ

PLPTRVICELFGVPDELKPDLVRLIAAIMNTVDQSAEHAASVQEQFGTVLPALIAHKTEH

PGDDMTTELIRVRDEDGDRLSEEELLFTLLVVIGAGFETTINLIGNTVVTLLDHPEQLAA

VRAGEISWDAVINEVLRVNPPIASLPLRFAVSDIDIEGVTIRAGEAILTTFAAAGLDPER

YGPDAAVFDAARDADDHLAFGVGVHRCIGAPLARLEAGTALPALFERYPDLSLAVDRAEL

RQVGSFIAYGWQTIPLRLS

>CYP124G5(647546455)SCLF

MRRSAPPGSFGSLRRFPPVNAPLSPTGTDLGAGTDLGDPAFWRLPRARRLAAFARLRALD

TPVRFGPEPGFWALVRHADVQEASRDPRHFASAPGVTVPEPAAWAKAVFGDSMVNLDGAE

HTSLRRIVSRAFTPRLLAATGENIRAVADRIVADAVRERPADFVASVASRMPFEVICDLM

GVPERCRAPIAHRIDGASEHVGVARRRLRVPGRGLAALGGLQWLMARLARERRRRPTGDL

ISALVCADIDGQRLSSRQLGAFFSLLLVAGVETTRNALAHGLVLLTDHPGQRALLAEDFD

RYADGAVEEIVRHSTPIIQFRRTVTAERALGGRVFRPGEKVVLLYASANRDERVFDQPDA

FDITRAPNPHLGYGGGGPHHCLGAHLARQEMRALFSALLSRAPRVRATGAPRLVDSSFDN

RVAALPFSLGSGSVPGSG

>CYP107CU1(647546740)SCLF

IGGARGGVRTLQSDGAAHGAVRRLAARPFTPRRVARLRERIQGITDGLLDTMERSGSPAD

LVSSFAYPLPITVICELFAVPEGDRARFGVWSDRIVTLLGISEQEVADARDALQGYLHEL

VTARRAEPGDDVVSGWLTADENGDRLTDDEVVRLSQTVIIGGYETTVNSISAGMWRLFQH

PEQLAAVRADPGLLRGTVEEILRYQPQGLFFLIMVARGDLELGGVTIREGDGVMPLPNAA

NRDAARFADPARFDIHRPPGGHVAFGHGAHACLGSALARIELEVALGTLLGRFPGLRPAV

ADLDELAWRGDRLVCGLRELPVRW

>CYP105M1(647546751)SCLF

MMNEAAPQSDQVAPAYPMHRVCPVDPPPQLAGLRSQKAASRVTLWDGSQVWLVTSHAGAR

AVLGDRRFTAVTSAPGFPMLTRTSQLVRANPESASFIRMDDPQHSRLRSMLTRDFLARRA

EALRPAVRELLDEILGGLVKGERPVDLVAGLTIPVPSRVITLLFGAGDDRREFIEDRSAV

LIDRGYTPEQVAKARDELDGYLRELVEERIENPGTDLISRLVIDQVRPGHLRVEEMVPMC

RLLLVAGHGTTTSQASLSLLSLLTDPELAGRLTEDPALLPKAVEELLRFHSIVQNGLARA

AVEDVQLDDVLIRAGEGVVLSLSAGNRDETVFPDPDRVDVDRDARRHLAFGHGMHQCLGQ

WLARVELEEILAAVLRWMPGARLAVPFEELDFRHEVSSYGLGALPVTW

>CYP136E1(647547024)SCLF

MTAGAHGPLAENTGPVDNTGHSRGSNGAGPDKNPGPAKNPGSAGNPGPAGSPGLAENPGL

VDVDTTHWLDAALQRVPLRTGPLAPPPPGSGLEPVPGDKGLPFLGLGVHTLRYGPAFQLQ

LLRRHGPVSWWQAFGRRIVAVSGPDAVQAVLVNKDKAFATGWPAVIGPWFDGGLLAMDAP

AHLADRRVMQTAYGEEAIAGYVLRMAEDAEAALARWPLGRAFTAVPAIRELSSEVTPRAI

LGVAYEPAGRRIMRAVEECIHAETAAVRLRIPGTSWYRAHRARRLLLAGLTRAVPAARER

AGDDFLSVLSRIGGPDGDRFSTRQLAEHALFTLIASHDTTVVATLASFYFLGRNPEWQAR

ARAQSLARPGGPPTVEALGGLDVLERVVKESMRLVSPSPINMRVAVKDTEVLGHFIPAGQ

LVSVCTGVNQLMPELWHEPQRFDPDRFAPDRNEDRVHRLAWAPFGSGAHKCIGLHVGMLK

VKATLDAMVRRFHWEFPAGYEAAWRFSSLPAPSDGMPVVLTPRTP

>CYP107L24(647547585)SCLF

METIDIAELHGFDANPYAYFARWREQGPVHRLRDPFGTEFWLVLGYEDARAALTDPRLAK

SPEALPDPPYEIHSIGPSLLESDPPDHTRLRRLIAREFTARRVEQLAPRIERITAELLDA

LPPTGRGDLIESLAFPLPITVICELLGVPTKDREVFRSWSSELITPTSEEAGRLALDALT

PYLDTLVEAKRRDPSDDLLSALVATHDEDGDRLSVPELRGLAYLLLVAGHETTVQLIAGA

VRALLVDPVRLTAVRADPSLLDAVIEETLRHEGPILTSAGRFTVAPVEIGGVTVPAGEIV

VVGLGAASRDPARFPDPDTFDPARDTRGHLAFGHGIHFCIGAGLARLEARIALEALLNRL

PALAADPEGGPPEWLPGLLLRGTRRLPVRW

>CYP107BY2(647547905)SCLF

MTADPYPGYAWLREHDPVCPVDDPHAPGRMWLVTRYDDVRACLADRRLGSGAPVNPDPHP

PGLSRLDDPRHAGLRRLVAPAFTPAAVSRLRDRTARTCARAVDSFAGRGRADLVAEYTRE

IPVAVVHDLLGVPEAERAPAADVLDMWYRAKFQQPRDEAKLAEMLGYVRKLVAYKRSHPG

DDLPTRLIESGALTGDELEVMVMTLIGAGHITTIQFLGTTVLRLLDHPVRRAALLGGDID

WSQAINELLRLDPPDHVAEYRYAGEDLTIGDARVAEGDTVLLSLAAANRDPNRFPDPDAL

DLTRDARPHLAFGHGAHTCLGSHLVKLETEIAITTLFGRLPDLTLDIPSSEVDWGYAPTF

RGPRALPVTFTPLSR

>CYP108B13(647548028)SCLF

MDPRIRAAGQALATPSVYADEERLHQALGLLRRQAPVHWVDAPGYRPFWAVTRHADIMEI

GRANRRFRNGPRPVLMPAAMERRAAAAEPGLRTLIHLDDPDHGPLRAVGADWFRTRALGD

EVRGRIADIARRRVDRMAELRGACDIAVDVAHPFSLYSILSLLGLPDSDYRRILELTRRL

RGSDPDAFLAAQAGLFRHFQAVATDRVSRPTEDLASYTAHARIDGERLSSGEAASFLVTI

ATAGHDTVASVVSGGLHALIDHPEQLDRLRDDPSVMPSAVNEMIRWVTPTKSFMRTATED

YDLREVTIRAGDAVLLSYASANRDESVYTDDPFRFDVTRSPNRHLSFGFGPHHCLGAALA

RMEIGAFFAELLPRLRRIELAGPPVLTAGTFVGGLRHLPVTYELNG

>CYP107F7(647548058)SCLF

MRGGHPDPSVCGGGTVRVRLSRPTRPPGAACPGRRTATTAPRPPHREPDPERPVMSTGSS

AHYCPFDYAEALEFDPTLRRFMREEPVARIRLPHGAGEAWLVTGYDDVRTVTTDRRFSRH

AVVGRDFPRMTPEPIVQDEAINVMDPPASSRLRSLVSKGFAPEQIERMRPYIQRAVDDLL

DRMAEDSSADLMRHLAGPLPLITICEVLEIPPADQETLRGHARTMMNISVDNKAAAVRAK

ADLRAYFADLTARRRADPGEDLISVLATARDGDELLDDQELTVMAMVLLITGQDTTTYEL

GNLSYTLLTRPDVRDLLRDRPERLAQTINELLRFIPFRKGVGIPRVATEDVELSGVTIPA

GDIVHVSYLTANRDGRKFDRPDELDFDRTAPSHMTFGWGAHHCLGAPLAQAEMETAFRTL

LERFPGIALAKPAEDVEWNTTSIWRYPLALPVTW

>CYP183Q1(647548116)SCLF

MTKHTARTPAATSAEPIPEAPGAFPLIGHALAMTRDPIRFVTSLPRHGAVVKMRFGPAET

LMICDPELTRRMFLDARTFDKGGYLFDRAREVIGENLITCPHSAHQRARRLTQPAFHPTR

LRGYTSIMSEVIAESAHSWPTDRPFDPVGPLLRLTAQVTARAMFSQGLSGATLDRFVTDI

VTTIDGIYLRSLVPRSLDWLPLPAHVRHWRARPRLDAIYRDIIRTYRAEPGDRGDLLSML

LATQEDREITEGAPPLSDAEIKDQLSGFFAAGTETTASTLAWALHVLAERQDLQERLRTE

VDTVLGGRPAGYDDIERLEQTRRILTEVLRVHSPIFFITRSVMNDTRLGPHLIPAGSIVA

YSPYLVHGDPQLHDRPDVFDPDRWAPGNAPPRHGFIPFASGPRKCIGDVFGVNEATLALT

SFATRWSWTAEPGSRVRPGVGVVVRPQGLRLRLTPRTGRAETGTESAARPAEGTPPGARG

>CYP147B4(647548120)SCLF

MPPETLFRRITDYASRADPYPLYAELREARVARQEDGSYLIGTFADVAALLHDPRISSDL

HNRTDPRAGGLLPPPDDMPPSFIGVDDPEHDRLRRLAMRPFGPPHSPGRIDGLTGEVTRI

ARELAGSLDGRDRIDLVDDFAYPLPVGVICGLLGVPAEDVPQVHAWTESIIAGFDLTPGQ

DEEARTRAAREARRGMAAYLSGLAESRRGHPSGDLLSDLANDHGPDGSLTPGELMVTAVL

LLIAGHETTVNLITNGMLTLLRHPDALALLRADPGLVPGAVEELLRYEPPVQLLPQRTPL

TDVEVAGVTIPRGAPLILVLAAANRDPLRYENADRFDPLRRDIQHFGFGSGVHNCFGAPL

ARLETRVALTELLRALDAPALVEDPPPYRRSPVLRGPRHLLIEQATGGGA

>CYP107AH3(647548131)SCLF

MTSADDPADAAVAAAERCTPEFRRNPYPVYGRLRRTAPVCPLNPPHGITTYLITRYEDAR

AALADPRLSKDMYGALESYRRIFGDSSVALDDHMLNADPPKHTRLRRLVNSGFTPHRVES

LRPRVRQIVTGLLDDCPIGEPVDLLAAFAFPLPIIVICELLGVPPEDRPKVQSWSTTVAQ

TGFSPESKRAQQLAEEALHAYFAELVEQKRRRPSDDLLSVLIGARDENGRLTEHELISSA

FLLMFAGHKTTAYLIGNAVLHLLRHPGQLRAVREDPALIAPAVEEVLRYDGSVETGTFRY

ATEDVGIGGTVIPRGALVQIALSSANRDPAKFDAPDTFDLARPANNQSAHLGFGHGIHYC

LGAPLARLETQLALTGLFDRFPRVALADPAREPEWLVVPFPAFRGLVELPVVLEPPAP

>CYP251E1(647548166)SCLF

MVSRFRVPTAPGGLPFVGHTRRLSHDPLAYLRSLRGAGDLVRLRVGPRCVYLVTCPALTR

RVLVDEARSFDKGAMFDELRLGMGNGLVISGGAFHRRQRRLAQPAFHPARIDGYTRAMTR

RAGERTAAWQSGVTLDLTRELDELTLDILLDTVLGDSAGAVREDVLRWLSAKSGVMRRVL

SPLSAWRARLLRPGPARPLPVTEDTLASLRALLAVEIHRGRLAPVGGGTEDMLSVLIAAR

DPDGVTGMTDAELTDELLTLFLAGTGTVSAALAWAFAEVSRHPAMERALADEALRVLGGP

RPATVADLPELQYTERFLREVLRCHAVWLLMRRTLTAVELGGVTLPPGTDVYLSPDILHH

DPGSFADPDRFDPDRWLAERREPGAQGARGAYLPFGAGNRRCAGDGYAWAEMALVLATTL

ARWRLVPGPGNRPRPRVGTVVRPERLRMIPEPR

>CYP163B7(647548203)SCLF

MNRRKGDPVAPSLDLTDPAAFVRNDPYAYWQEIRVRSPVFWHPPAAGRPGFWVVSRYADV

RACYDDGQRLGSARGTVLDVLLHGDDSAGGKMLAVTDRPRHRALRSLMLRAFSPRVLGEV

VRRVEERTARLVEETVGRGTVDFAADVAAHIPLNTICDLLSIPGRDRAELLAWSKTALAS

ERPDGDRLDALEARNEIVLYLVELALERRARPGDDVISMLASAEADGRPLTPEEIALNCY

SLLLGGDESSRMSAITAVLVLARHPGQWRALREGRIGVDTAVEEILRWATPGMHFARTAR

CDLTLGGQRVRAGDIVTLWNTSANNDETVFDRPRTLDLARRPNPHLALGHGPHFCVGAAL

GRAELRALLTALTAHAGTIEQAGEGRRIYSTFLHGYSSLPVTFRPRRSAPRPDRP

>CYP105BG1(647548208)SCLF

MSTPPAPGVSASTMPFPAPRGGCPFAPPPLYEAAREEEPLTRVTLFDGSEAWLATRHSAV

RLVLSDPRFSADIHTPGFPLTSPRQRFMAQGEPSFVRMDAPEHTRQRRMLTSWFTARRVR

AMRPAVQALVDERIDRIEPRGQADLFSEFALPVPSLVICDILGVPYEDHEFFEGTSRAMA

QWNTTPEESSRCWNALHTYLSELAAAKRECPDERLVSHLAARDDLTLAQVASMCRLLLVA

GHDTTASAIGLSVAALLANPEWAAALRDSPGHSPEAVDELLRLVSPLGHHGIGRAALADV

EVDGRTVRAGEGVLVIPPTANRDGRAFDRVDAFLVAGERDQSTHHLSFGHGPHLCLGAPL

ARLEMEIALETLFRRLPGLRLARPLEELPFRGPGAIHGLDALPVRW

>CYP183P1(647548237)SCLF

MTTQKHDSSTTGNFTTGRAPGALPLIGHAGHLMRRPITFLESLSTYGDLVEIRIGTQRAY

VPCHPELLRQVLTDDRTFDKGGVIIERFRSVLGNGLGTCPHSDHRRQRRLMQPAFSKARL

EEYSAVMRQEITALSGSWQEGQEIDAFAVLYGLGLRVVVRTLLSSDLDDPTVEQVRHSFE

RTLHEFLPRTIIPQQIQQLPLAVNRRYHQELQALDEVVNRIIADCRDAGLERGGLLATLT

ASHAGGGVPMDDSEIHDQVITMLLGGSESTGTTLTWALYLLSQHPEIEMRLHEEVDTVLN

GRPADWNDLPNLSLTHNIITETLRLYPPGWLLSRTTTKEVELAGTRMAPDTTIVFSPHVI

QRQGDIHGCPDVFDPDRWSPDRTADLPRGNFVSFGGGPRKCIGDTFGEVQSTLALSTIAA

RWKMECAPGSDIRPVAFDFAYRPRRLILRPRLR

>CYP157C20(647548280)SCLF

MTTPRPDVPQDLSVPVPPPGCPAHDLGPDGTHRLYGPVAETDPPAMYELLRREYGPVAPV

LLHGDLPAWLVLGHRENLEVMRTPSLFSRDSRRWSMFREHRVPDDSPLRPMIGWQPSCVF

ADGDEHARLRRAVTDGLAQFNRHGIRRHVTRYTHRLAATFAARGEADLIRDYAEQLPMLV

MTRLLGMPEESGPRLVAACLDLMKGTETAVASNDFVADSLRALVRHKREQPGRDFADSLI

THGSGLTEDEIVHHLRLVLIAANETTVNLVANTLRLLLTDRRFRASLSGGQMTLPDAMEQ

VLWDAPPVSVIPGRWATGDTVLGGQHIKAGDMLLLGLAAGNTDPRIRPDRAAPVHGNRSH

LAFSSGPHECPGRDIGRAIAEAGIDTLLTLLPGLELSVPAAGLSTTAAWMTERVTALPVR

FTPRRDLDALPVPSAGGRPRAGGQRPPAPGVAEPATTPTATARRARWTSLFG

>CYP274C1(647548351)SCLF

MDGLAFCLRRDGGRRRVPATEARLPHVPACGAASRKAVVMVNVPEGSARAVGAGRREEAR

GVPDGLPAPPVAPGRLPVLGHLVAMLRDPLGFMASLPAVAEVVTIYVGTRPVHVANSLDL

VQSMLVTEADSFQRGLLFEKAEKVVGPGLTMVEGEAHRRQRKLVQPAFSPRRIEQYTQTM

IEAAEAGVGSWRPGRRMDADRVMYDLGMDVFTRVLFRGALDGASADLVKQATSGIMAGMV

AHSLYPAQWLEKVPIPLNRRFRRGDAQMTEVVDRIIHHYRQGGTADHDGAAILDALMAEN

ERASGRPLDSVELRTEVIHLLVAGAEGPGASLAWLFHELSRHPDVERQLVEELDAVFGDG

PLTVAGLAGLTFTRAVVKESLRVHAPTWLLTRRALKAVALGGHRIPAGGEVAFSLTALHR

TAPQYDDPGRFDPARWLDGRTARLPRSAYMPFGTGRHRCVGDHFTLQVVLVCAVTIARRW

RLVPCPGIRVRERPVALVRPSGLLMDVAAR

>CYP183U1P-N-term(647548353)SCLF

MNPTYTMAQAPGALPVLGHAAAFARRPLDFIRELPDHGDLVRVRLGPLTAHVVCHPDLLH

RVLAEDRVFDKGGPVFETFRKTAGNGLLGCPARDHRRQRRLVQPAFHRSRLPGYSAAMAE

EIAALTEPWRPGQTLDVPALMYRLTTAVTTRCLFASAAQAADLPSLHESVDIVTQGIARR

AMLPVPALHRLPTPANRRYQRTQRYLRHLTDTLIDSYRTQGTDQGDLLSMLLAPQDDGGP

GLTGTEIHDQILTFLLAGIDTTAILLS

>CYP183U1P-C-term(647548354)SCLF

MLDGRPAQHDDLPALELTGRIIQETLRLYPSAWLFTRTVTTDTELGGHPLPARSTIMFSA

YQIHRRADLYPHPDRFDPDRWLNAPKPPPGTHLPFSSGPRKCIAETFALNETTLALATIA

TRWQLDPTPGPPVRPARHITLQPKKLTMRLRERSGSGAGTAP

>CYP1038A5(647548357)SCLF

MDSARSGTIPELAPDAFLRWRESGAETVDLLAQARRLGPVSAFRIGERPVVLATGPQAVQ

EVLARCPERYVKRSHRARVLLGDGLLAAAGEQWRRQRRVLQPHFTGRAVHHYEQLMHDCA

EETARRWAATAARKTPRRLDADLQHYSLDVIWRAATGRPLDDATHRALRVASDVLAALPA

LAPANSPQPDLTAQVDEVDAIVEPLIAAARTDPAPGLLRVLFDSADQQPLYTDRLIRDEL

VTLLTAGYETTATTLGWLFLLLHQHPEQRTWALAAGPAGSPARTEAIRALVSETLRLYPT

AWLLPRYAAEDGTLGGHRVLAGTTILICPYLTHRDPDIWPEPDRFLPQRFLDPHRRTPPG

AYYPFGIGPRACLGAQFAVREITILLERLLPAFTAAFPDIPGQRFGVTIQPDRELTATIT

TTT

>CYP154A15(647548361)SCLF

MSDISSQPYVLDPAARDRAAEDALLRARGSVTRVDVLGEEAWAVTDPALLKQLLLDGRVS

KDARRHWDRFPDHTTGWPLTLWVAVENMFTAYGPEHRRLRRLIAPALTARAIGALEPDIE

RFTRELLDKLAATPPGGTVDLRENFAVPLPIRVIAHLMGLPEHLLPEFRRTVDGVFATDL

TAGEAAAHTQGLYRALDALIALKRAEPGDDLTTRLIAARDTDAGGDGRGLTTQELRDTLL

LMISAGYETTVNLIDQSLTGLLTHPAAHTAARSGTVSWPDVAEETLRWQAPLPFLPMRYA

TTDIALPDGVVIRRGQALLPAYSAANRHPALHGPTAERFDPSRADKTHFSFGHGVHTCLG

APLARLEATIALRMLDARFPALSLAVPAHDLTPLPSFLTNGHRALPVVLRPGTSD

>CYP183N1(647548609)SCLF

MTAPPGHTARPIPLAPGCLPFLGHAVRLIGKPLAFLDEQRGRGDVVEFRIGRQPAYILNH

PDLVQSLLTGSRRRFDRGEIFAIASPLFGNGVAVANGDHHRDRRRAVQPLLSHSRLETYL

ESMAELAAARADGWSDGRRIDLNAEMADVTMNVVAATVFGQSLPAGFGSVIHHQLPLVVA

GLARRAYGPAAVLLDRVPNPERTKYRSALTRIHDVVDTLIAANRDTPTMTALSEGIDERQ

LHDDVTSLLIGGSHTSGAAASWLFILLSRHPEARRRLHQEVDRVLGGRSATPADLPALVH

TRRVVQETLRLYPPVWLFPRRAADDLRLDGYPIMRGTQLFYSPYALHRDPRWYFRPDTFE

PDRWDPDRHEQPPRGAYIPFAAGVHGCPGGDFALAELTLLAATLTAKWNLDLAPGSRPRP

VAAATLGPEPVTMTVSRRSRPADSGDGS

>CYP1065A1(647548623)SCLF

MSPQQTARPRPDSRYGVHMSSLDALDLFDTLARPEQLSDPYPFYDWLRANRPVHLDPDGT

VYLSCYRDSALLKNPDIRDAAEGDGSSHTLNTYNQSLIKTVPPQHTDLRRMGAVAFDPDL

LKRAGAQVRETAGHLADQVAEKPAHGDAADLHSMYSLPFTQRTAAIVFGIPDEDFDLLAA

LPGRMFSALYPKADPATLADADDASRALAAYVEDGLRRRRFTPDSGFDRLARMQDTASMD

DLVRLCWLLWWASYTSSLAAIDLAVLTLLEHPHTIQALRERPDAWVKEALRYRSPHVINS

ANLTTSRPMTIGTVSVPAQTPVRFLISSMNRDPEAFPDADTFDPDRPAMPRHLTFGEGIH

TCIGAKLARMELAIALTTLVDRIPDLALASAPVWRPYTTQRLVSSLLVTSSPTRN

>CYP2189A1(647548725)SCLF

MSFPSSSVDLLDERVLADPCPVHAALRAVGPVVRLARHHAWAITHHQAVEEALTTGILIP

RPADSSTPACPGHHPRPDTAAVRAVQTVLRQAREVLAGHTHAGVTDAAAAARHLTARTVL

ALAGTPRPAPLRLPARKPVTAALAALLHPTPPPTRPKTRPAGPGARPDGAGKGLSPVVRC

ALAHADVTAAGLTETLWQLAHHPDQWTRVRAQPSRLAPAALHEALRLQPPQPHQVFTAAA

TVLLSGVEVAAGDEVWLLHRAAGRDPAQWGPTADSFDIHRPRTNPRLPGSHCPTATALAR

AQTTTLLQALAAHPYLTPAAAPGRTHTTTQHTWLFAPLTTNPTTSHNPRGLPAS

>CYP183E3(647548770)SCLF

MTGGTIARVPGALPLIGHTGAFLKSPLEFLKGLSAHGELVELRFGRKRVLVVCDPDLTQE

MLRRDRVYDKGGPIYDRMREAAGNGLATCPSRDHRRQRRTIQPAFHHSRLPGYARTMTEV

IDEAVRGWDGPEVDLCHETRSIASSVLLTTLFGTAFAPEARDALARDLETILRGGLRRAV

LPEALSALPTPANLRYQRARRRARGMMADLIRAVRAGDGSGDSLFATLVNQHGAGTGDLP

MTDEELVDQAITMYVAGTETTAGVVCWALHLAAQHPQAGRRLADECRSVLGHRAATWEDL

PHLPYTRQTLLEALRLNPPGWLITRLTTTDDARLGDHAVPRGSTIVYSAYLLSRLPARYE

EPEHFAPERWAPGTMGAKVPDGVFGGGARKCIGDEYALIEGVLMLATITARWDVRTAPDA

PKRPHLPALTLNPTRLMATVTPAAAPDGSR

>CYP105BH1(647548838)SCLF

MVDTAPPGPSDAPSYPQPRPCPYQPPPAYERLGAQAPLSRVTLFDGRAVWFVTGYPEARQ

LLADPRISADRERPEFPITAPRLRAEVSRRFILLSMDAPVHGEYRRLLNPDFSRKRIASL

RPVVQSVVDDHLDRMLEQGPPADLLRDFALPVPSRVISELLGLPPEDTELFQRLSGRLLR

AGSADDAQEAARELGDYLGALTARPPEGPGPGLIARLAHDEVATGRLSHADLVRIALVIL

LAGHETTASTITLGTVTLLDHPEQLARMRSDPATVPGAVEEILRCVAVTDLAGVRVATAD

IPVAGQIIRAGEGVLLSSTMANRDRRVHTDPDTFDIDRTGRHHLSFGYGIHQCLGQSLAR

MELEIAFSALFTRIPTLRLAVPVDRLPTRPPSSGAIQGFDELPVTW

>CYP274B1(647548854)SCLF

MTQPLSGRTDGSPPPPERGRRARVPAPGSPVPTAPGRLPGLGHLLPLMRNPVRFLTSLQP

LGGLVRVDIPTPVHVVNSPDLLHRILVTDARSYEKGAAFDKARTILGNGVLFSEQPLHLG

QRRLIQPAFHRSRQPHWRTVAADCAAEHLDRWQDGDTLDVLDEMHRIALEMIIRLFFAAR

PTPRTSERIHQCVNVLLGGIMIRFLSPGTLLERLPTPGNRRFERAIRDLNTIVADLARTA

RTTTANPMGDGDHQDLFTTLCPHAADLDCSLQQSCDEAISVLVAGTETIATTLSWLFHEL

ARHPDHENELRADLAAADGDTTRTGLLDRLLNETLRLHTPNWVLMRRARTEVELDGRTLP

AGTEFLFSLSALHRDPQVFPHPDRFDPDRWLPGNSPVQHRTHFIPFGDGNRKCLGDTFAR

TEMRAVAAAAVTRWTLRHTPGTKVGETRLFATLQPTGLRMTARTAPKPR

>CYP183T1(647548857)SCLF

MTMAESAFTRAAAPGALPVLGHALHVLRDPLTFVSSLPSHGDLVEIRLGPQRMHMVCHPD

LLQQVLTDDRTFDKGGPLFERLRDFMGDGLATCPYAVHRRRRRLAQPAFHHQRLEQYGAV

MTRQLERMLEEWKDGQVLDLFPALSAFTLRTVSRSLFAADLGEDRIDTIQRSFVDVFSGA

LPRMLVPDSLQRLPLPGNRRYREAARTLTDTVNQVIAEYRENGTDQGDLMSMLLAARDED

GSALSDQELRDEVVTLFVAGGETPAAVLSWAAVDLAAHPESLRRLHEETDRVLAGRLPRW

QDLPQLPYTARVIDESMRRYPAGLLLTRTTSRETRLAGLTLPPGSTVAFSPLLLQTRPEY

YHEPERFDPDRWLPDRALDMPRIAFAPFGSGARKCIGDAFAVAEMTLALAGMASRWTWER

TTPTDLRPALSPAAVRPRRVSLRLTARTPAPQAMKPL

>CYP107CW1(647548940)SCLF

METRARSRTRAGEFAPTRQSEHTPAEHNHGRRSPQPRLFCWETPGMGTQDPDRADTDEEP

LDLTDPALVEDPFTAYAHIRARKRMVRGSVPGVDPMWVATRYDDIRAVMSDPRFTIDATG

VPGAPVAHRTEQTWQARGMHRGYEKYLRAGIFDADGADHRRLRGLVGAAFSPRRVTGLRP

RIETVATGLLDRLPDHAEDGVVDLIEHYARPLPITVICELIAVPEADRDRWRARSATLVA

GVCGDELGDALAGMVDDAITLVDHHTAHPGDDLISDLLDPRHRDRLSPEELVALIVNLVV

AGHITTVNLIANGTEALLTHPGQLALLRDDPTLMPHAVDELMRYCGPVVRALPRYALCDT

TLGATPVKAGEAVLPIVSAANRDPAAFTDPDRLDLGRTRRLREHHLGFGHGAHRCLGAHL

AHEEAVIALTTLLDRAPELRLAVDPRRLEHGTNPVNRHLKALPVRW

>CYP1066A1(647548943)SCLF

MASPQTPLRTLHQVPEAPGRLPLVGHVATLARDPFRFLMGLREHGDSVLLHLGRTPVLFV

SDTELVHQALVAQGSVFATGRIFERLGAVFGNGLPVADIPTHRKQRRIMQPAFHRSQMGR

YSTTMTANAEAMVARWSPGRRLVADTELLDLALSNTTEILFSSRLSDADRAEVHRSIPVI

AHDVLVRAVMPAFLDPLPIPLNRRCDTAAARLRVIVEGILDGHYRSPQHHDDLLTVLVQA

RDPETGRPMDGTQLRDELITMLGAGTETTSSIAAWAVHEIARHPVAERRLVAELDAVVGD

RPVTPADIPRLTCAEEILNETVRLHAAPLLTRRTVKPTVLGDIRLPAGTEVAFSPYALHR

DPRHFPHPDAFWPERWQHIGSGRPRSFFLPFGGGAHKCIGDSMAWTQLHITLATIYRRWR

LRPDRSHRVRQLPAAITRPDHLPMIAEPREPAPARAEGARS

>CYP112B1(647548974)SCLF

MLAARDAPPTPVRLPLGIDGWLVTRYDDIRAVLGDPRFRPNGMPPGGYTVNQPYFLSAPG

WLASVEGEEHARLRRLVGPAFSRARIGGLEPVVEQLTHTLLDALEKKGPPADLCAGLTLT

LPSMVLCALLGIPHGDHDRVSAWTGQALIPPALLPPGAAERAHGEFTAYVAEQLARKRRD

PAEDLLSDLAAAGTDGAIAEEEILGLTVGLLVAGLGFTTVQMEYGLCALLRHPDQLALLR

SSPELMDGAVEELLRMFPPGHQLDGTLRYPSEDIEVGGVAIPAESVVLVCAQAAAFDPDR

FDRPQDFDITRTANPHMSFGHGAHYCTGAPLARAELRTVFRALLDRFPRLALAAPLESLQ

VRMTHAGGFQELPITW

>CYP154D11(647549005)SCLF

MLGGPCPRRAPWTYVSRNGAVHGTAPAAGALPSTTSRRDLPYGAFRTESSAREGLSVRAP

RTSAPVRVLPYGASRRGFPYGPPRPAVRAPGRPLDALFTERCRPRTMDPRPAPTRRTPAG

RRAEPTDRRRIGPVGGIRPHLPSGAPMETTDPAESSRTEPLPQLDLSPRRSPHATNARLL

AEGNVADTVLPGGIPGMAVLGHEALKDFLTHPEVTKNPEHFTALQEGRIPENWPMLLFTT

VPGMHTADGAKHRRLRSLISKEFTARRVEELRPRIEGHVSRLLDGLAAAAAAAGDGVVDL

HPHFALPLPLEVICELFGVDEEYRQRMHELTEQTLDVTGDPEPAALAYRELTEVVATVVV

NRTREPGDDLTSALIALREEDGDRLSEDELNATIRTSIIAGHETTMTLISNAVRALCGHR

EQLDTVRSGGASWGDVVEETLRWDAPISYFPFRYPKRDLVVDGTLIPKGTPVLAGYSAAG

RDKAAHGADADRFDVTRAARESAVRHLSLGHGAHFCVGAHLGRTEAEIALRSLFARFPEL

DLAVPEADLLRHPSPLDSATDGLPVRLGTEAG

>CYP183S1(647549011)SCLF

MTSTVAALGHPPPAAPGALPLVGHGLGLYRDPLRLLLRLHRPGTGPGVLRLLLGRRPIVL

VTRRELVHRILVTDPHPYDKGGPFFDFIRYMAGNGLATCNNADHRVQRPLVQPAFHPTAI

SSYGPVVADAGRRVVDDWHDGQTLDVSEEAMKLATLTVLGTLFRDADFAQAVTDSVRTFP

QLEKDGFIRMVLPPSLHRLPLPALRRVDRGRELIRTALAGAIAAHRATGADGNEVLSRLM

AATGPDGTPAFTETDLVDQVFTLIGAGVETSASSVASALHLLATDQDVQHRLRAELADVL

GGRDPVAEDLPRLPLLGRVVTETLRLYPSVWLVSRVTTAPVELGGHRIPAGVDVVFSPYC

LHRDPEVFPRPASFDPDRWLPERAGRAQRQALIPFSTGRRKCVGDTLAMNEIALFLASAL

TRYRFRHAPGSRSRPTALATLSLAGIRLTVHRLTDPDPDPDPGSGPGPDPGADSGFDGHQ

PAEPAR

>CYP107BX6(647549046)SCLF

MTVADRPARRPPLHRTVTGEVGPPRLVDLPDGSPAWLVSRPAEVRQVLSDSRFRRAALWS

GDGPSLSAVPDLVSNPDLMFNQDGEDHLRLRRTLSRAFTPRAVARWEPWIAAMVEQCLDR

LAGCEPPADIVAEYALPLPVMIISRLMGLDPSVRGRLRHWAEHAFSDGSHDGEDVASVMA

EFTAFGADLLARRRRTPGDDLVSSIVLAAETEGGIPEAQLVQLVCGLVVGGHDSTMTMVS

NCVLYLIGERPECWARLGADREAAERLADRMLHLIPLGDDKGSARHAAEDIEVGGVTIPA

GSVVLADCALANRDPETYSARPFDDLFAPLEAPSLSFGAGPHYCLGAWLARLELRLALHR

LAARFPGLRPAEPVDQVEWRLGSTSRGPQRFLVAW

>CYP107CV1(647549072)SCLF

MEHCPYRFPFPRVPATRPPAAYAEFRRDTPVVRVAMPSGDPTYLVTRYEDARMVLTDARF

SRSLELAGLRRGDVGLADHLLLSTLANMDRPEHPRLRRLVAPAFSQETVERLRPRIERIT

DEHLTELIGRRPPVDLTEALCVRLPVAVLLEYLGVPLSGREELLRWTGILTDLSGHTQQE

AEEARELLHHHLREVIAERRRDPGDNVFTDLARRCYEEERITELELEALAMFLLIGGLQT

VVYQLGLIVVALLRSPAHAAELAAAERPEPAIEELLRYTNAVESSLLRITTEDVVVAGTT

IPRGSAVLPAIPAANHDPERFGSPETLDFDRTATPHLTFGHGMHRCLGAPISRLMLNIAV

PALFRRVPELRLAVAEDELSWLPDRLLTGYASIPVTWSPRPVPEGPGPEPVR

>CYP105BG2(647549197)SCLF

MITAVRSGETGVRRTYARTGGDGRRDHRRETVPTAPSPGRAEERPGRRRAACRRSGAPVG

DVSVPGAAGQGRAARLPQASRRRGPSMSSPLGSDVSGSTIPVPAPRGSCPFSPPPVYEAA

REEGPLTRVTLYDGSEAWLATRHSTARLVLSDPRFSADINAPGFPLINPQQRFFAQGEPS

FVRMDAPEHTRQRRMLTSWFTARRVRAMRPAVQALVDERIDRIEPHGQADLYSEFALPVP

SLVICDILGVPYEEHEFFERTTEGLARWNATPEETSRCWADLHAYLSELAAAKREQPDDR

LISHLAARDDLTLAQVASMCRLLLVAGHDTTASAIALSVAVLLEHPERTAALRGGAPERY

ATAADELLRQVSPLGQHGISRAAVADVEVDGCTVRAGEGVLVALSTANRDSRAFDRADEL

VLDGEEGRATGHLSFGHGPHLCLGAPLARLEMEIALETLFRRLPNLRLAQPLEELPFRGA

AAVHGLNGLPARW

>CYP1064A1(647549376)SCLF

MDIPGPEPRADGGVKAVADAGGLHRYQLELHAAYGPVVRFQLPGADTAVSIADPVLLEAT

AHLDERPEGLFSFLDPLCEAGNLQVLPAAEHTPWRRLLLTVLAGRPSHERHLPRFTALTT

ALADRWAEHAEHPGHDRDDGDGGDGGGRPVELQKDLTALSLRMICAYALGSGVTDPEGVV

TAFERVLTAHLGKLYRPPRPGPPDEEAERTERADAALAFLRGAVDEVIAAHRGGRAERSD

LIGALVEAGETPARIRDTVMMTMLAAHHTTGVAVSWALYLLALHPGAAGRATAELDRVLG

ERAAPAYTDLRRLTYLEMVLKEAMRLCPPGPYGARRTDAELVVGGYGIPAGTTVFYPFWA

IHMNPDHWPEPERFRPERFTPRETAKRPRLAHVPFGIGPRSCEGARLAVVEAELILAVLL

KRFRFRLVPGHEVVPVERFVLWAADDIRMTVSPRGRRQDVS

>CYP245A4(647549394)SCLF

MPSPATLPRFDLRGWDSEDIAHPYPVYRRYREAAPVHRGVSGAGEPDTFYVFSYDEVVRV

LSSRSLGRDARRTASAEPEPETETETEPVKVKADAEAEAEVEVGETGRDTGTAAAPVPFP

AGCRALRTLVGNWLVFLDPPRHTELRSLLGTEFSPSVVAALRPRITRIADRLLERFVQDI

GAPGGADLVAGFAAPFPLLVVCELLGLPQEHHRWLRAHALALQEAGTTRSRGRESGWSAR

AEAAAGEFARYFRREIRRRRGETVRRPGTGGPGSGSWAGRRDLLTLLVRAGDRGAPLDTD

AIVGTCVHLLTAGHETTTGLLAKAVLALRRHPDLLDELRAAPALTPGAVEEFLRHDPPVQ

AVTRWAYEDIRLGDHEVPRGSRVVALLGSANRDPARFADPDVLDVRRAPDRQLGFGLGIH

YCLGATLARAEAEIGLRALLDGVPGLGRGGHRVDYADDMVFHGPSRLLLRPSASVPPDSA

A

>CYP244A2(647549398)SCLF

MNVTGRAAAERFTTGPVTTGPAVTEPAVTEPAVAGRAVAGRTRLAQAPATSMPTDPGPFD

CMPELLAAARVAPVVRIPYLDRHAWVVCDPELVRVALTHPALAKDVTLVPAWMRKPGLML

GSQPDPEYARTMIMSDGAHHARIRRLHASVLSPRNTETWGARVGVQVDGFLDELERSRSS

GPGEPGGTGGTSTTGDSGEPGEVNVVTAYTHRVPLAFISEMLGLPPGAREQLRSITDVIL

YSSDYPDRARAVGALYGAVEGWVRDPAPLRDGVITGLLSAATGPDAADGPDAAVTEGEAV

VWTLAMIITGYETTGSLISTALYEALRRPCDERPRTDDEIRAWVEETLRVHPPFPHPTWR

FATEDIDIGGFLIPRGAPVQVNIAAANRRPDEGADSFTPERAGQGHLSFGLGPHYCIGAS

LARLEAQIAVRGFLRRFPRARLSTRTAVAWESEWMIRRMSALPAVLL

>CYP183E2(647549419)SCLF

MQHPRTASVATAPGALPLLGHLPRFVRAPLAFVRTLPAHGELVEIRLGTRRMVVVCDLEL

TRTLLRHDRVFDKGGPLYRRLEEVGGVGLATCPASEHRRQRRLVQPAFHQRCFPGYAQIM

TDRTDAALTCWQEGGTVDLTRETRRITADVLVATVFGADLDPAVHALLAADLHELLAGVF

RRSLLPEPLCRLPTPGNRRHARASARARRLMGELIAGHRAAGAASHRTPDGPADPRSGLL

SLLLAARDPEGGPAFSDEELIDHAVTLYVSGTETTAGAVCWALHFSALHPPVHRRLTEEI

ATVLGGRTATWADLPRLRYTRQTLTEALRLCPPGWLLTRRTEEDTTLGPYALPRGTVLAY

SPYLLHHLPEEHPDPDTFDPNRWQPGPDGSPPPPLFLAFGAGPRRCIGDEFAYLQGTLLL

ASLLSHWDLHPHPSTRPLPRLPQLTLNPGPLPTHLTRPKHHHPSPARPRP

>CYP183R1(647549454)SCLF

MAPGALPLVGHVPALVRDPARWLAACRDTGGIVTVRLGTRRAHLVCSSELVHELLVGQVD

RFDKGGPLFDRVRVVTGDGLITARHRDHRRQRPLMQPAFSPAHVSAYTAAMREECAALTA

RWGPRQRIRALSEMHSLLAGVLVRVLLHADDLPDGAWLAAQVKTLVSGITTRTVLPWAER

LPLPGNRRFQRALREIHGAADRAVDASRSRPEQSRLMAALLAPDLDGDAFTDEDLRGQVV

TLLGAGIETTAATLVWAFLLLARHPEIEARLYEELDTVLGGRLATPDDFRRLPLTRTVVL

ETLRMYPVAWMLTRVTTADVELGGHRFAAGEDFLFSPYQLHHDPAVFPRPGVFDPGRWAA

PPTPAVRQAFIPFGTGRRRCIGDTFALAEVTIALSAIVPRWRLCPAPGGRRAAGRPRFRA

TLTPEDTPMVTLPR

>CYP1199A6(647549502)SCLF

MPTSSPQRLRTTVFSPRLAALMDDHTGQDVFRLEPDTIGVAGPEVMDRVLAARRATETER

PTFKPLLGRSIPRAEASAVTRTVGADVREALRHPLPKDVDLSGPWPWTGHYVLRDLILGR

DPYRLRLLMSRRLELTPVLTWSVVTLGAALPRLPRQGDGRTALADRTAGAMGYQERRYAM

GMYRRSAAPVCFTVSTLVANALWLGFPFADDTPNRNIVHETLRLLPPSWNLLRNASPEYP

AVDGRIGSKDDVLLLPLLSHRDPALWDDPEAFRPDRWDGLDPDRTPGYLPFGHASERCWG

RHMVLPLAELLLTRIRESGLVVDPDQRTAKVPLLGLLGVDEVRLKKPARFR

>CYP285A4(647549591)SCLF

MTPVPAAGQCPVSASPPGALTDPGLFQADGAHEVWRRLRRHTPVSRQEAADGTGYWSVVR

HADACRVLRDHDTFTSERGTLLNLLGKGDPAGGRQMAVTDPPRHGRLRAPLQQALSVKEV

AHDREAVRGAVTRLLDPLADGGVFDFAAAMAALPTAVIGTMMALPEEDWPRLTRLTHACI

AADDPEYQLAAGPQATLVAAHRELFAYFQDTVTQRRRSPGDDLIGVLLAMELDGRPLSPG

EIVSNCYSLLLGATVTTAQPPNAAMAELAGTAALDDWAAHPEALSSGVEEALRWSSPTQH

FMRHTTREVEIGGALIPAGEAVVVWLGSANRDESVFADPDTFDIRRRPNKHIAFGAGAHY

CVGHTVARATLRALFAELLGRFTGFELAGPPRRMRSNIIAGFTRLPLTAKPR

>CYP208A6(647549634)SCLF

MSWAARTPPGPLRRELPRLFRKLVTDRLGLLTEAAALGDAVRVTLGPRTLYVFNHPDYAK

HVLADNSAAYHKGIGLTEARRALGDGLLTSEGELWRTQRRAAQPAFQHQHLAAQADVIVE

ETAKLIARLRAQESGAPVDFTQELTELTLGVLGRTLLHTDLTAYGTVGHAFEAVQDQAMF

EMVTQGMVPLWAPLPQQRRFHQARAELRRVVDQLVAERTDRPAESPADDVLSRLIDSTRR

EPDPEVGRRRLHDDLVTLLLAGHETTASTLGWTFHLLDRHPEAAARVREEARGALGDRAP

VLGDLHALPYTGMVVQEAMRLYPPVWILPRRAQRADDVGGYHVPVGTDVLICPYTLHRHP

EFWAEPERFDPERFDPARPADRPRYAYIPFGGGPRFCIGSNLGMTEAVLVTAMIARELTL

RTVPGREVVPEPMLSLRVRDGLRVEVRAAAQ

>CYP161G1(647549688)SCLF

MALPERLPFERTDPATAAPEFHDLQVSGGIARVRTFAGHPAWLVTRHDDVVRILSDDRFG

RAHPDPPSAPVLSATGQFYGPQGTDHERLETEFRVLRRVTANSLSVRRVRELRPRIEALV

GRRLDRLEAAGAPADLHALLAEPLPVEVVCEVLGVPFADREDFQRWTGDVVYLSEPERVG

EALLRLNDYMRALMERKRAEPADDVLTDLVRARADGHIDEDLAVLWSVALLFAGHVTTVT

RIGTGVRYLIARPDQRRAMLSGPRLLAATVEELLRISQPDLGLVWRWAVVDVDTGSGRIG

AGDLVLFANGPACLDAAVYPEPHRFDIHRDQAPHTAFGAGPHLCVGAALARLELQVVFDL

LFRRFPGLRLAVPEQDLRWRDSQLTGGLLALPVTW

>CYP151A4(647549730)SCLF

MTGPAPTGSVPVFDAADPGFSITSAAVHRARENSWYARTTYGLAVLRHAEVAELLHHPRL

RQGSVSWLARNGVTEGPLADWWASWVLHREGEDHRRLRRLLNPAFSSRSATALLPRFRAL

ATELTDAFADRDRCEFVAEFAEPYAARVIAMLLGIPEQEWPVIARESAVLGLALGVTVRQ

DLDRVERALAALHAYADELIADRRRRERDDFLSRLVHADRDGDRLGDEELRDSLVLLVFG

GFDTTRNQLGLALQTFARHPAQWRFLADRPELGARAAEEVIRVNPTVRWITREALEDFTF

RGLDIPAGTTVQLWTESAGTDPRVHGPYSFDITAEREPHFGFGGGFHHCLGHFVARADIA

EALPVLARRLRDLRIAEDAVWLPDSGNTGPVRLPLLFTPAP

>CYP159A1(637264808)SCO

MSTAQQVPDILSPEFAANPYPAYRTMRDSAPLIRHEATQSWIVSRYEDVERVFKDRAGQF

TTENYDWQIEPVHGRTILQLSGREHAVRRALVAPAFRGADLQERFLPVIERNSRELIDAF

RHTGRADLVADYATRFPVNVIADMLGLDKADHDRFHGWYTSVIAFLGNLSGDQEVAAAGA

RTRTEFAEYMIPVIRERRENPGDDLLSTLCAAEVDGVRMSDEDIKAFCSLLLAAGGETTD

KAIAGIFANLLAHPEQLAAVREDRSLIPRAFAETLRYTPPVHMIMRQTATDVTLSGGTIP

AGATVTCLIGAANRDETRYRDPDRFDIMRDDLTTTTAFSAAADHLAFALGRHFCVGALLA

KAEVEIGVGQLLDALPGLRTEDGFEVVERGVFTRGPQSLPVRFTPAA

>CYP157B1(637264809)SCO

MTDIDPSPHPVAAPGCPVHPDAVPLAGLEYQQTPSELYRGLRAEHGAVAPVLLDGGIPAW

LVLGYPEVSYVTSHDELFARDSRRWNQWGSIPPDWPLLPYVGHQPSVLFTEGEEHRRRAG

VITQALAGIDQFELARDCRHLADRLIAAFAGSGRAELMSGYAHPLPMRAAVRMCGMPHTG

VETRQLVEDLRISLDAAEGDDPVAAYTRVGERIHQLVRHKRERPGPDVTSRMLTHPAGLT

DEEIVQDLISVIAAAQQPTANWIGNTLRLLLTDERFALNVSGGRLSVGEALNEVLWLDTP

TQNFIGRWAVNDTQLGGRHIRAGDCLVLGFAAANTDPQLWPEAHVGAENSAHLSFSNGEH

RCPYPAPLLADVVARTAVETLLERLPDLVLALEPGELTWRPSIWMRGLTALPALFTPVVA

>CYP105D4(637264996)SCO

MTDTDTTTNTDTTTNTHPAAPVAFPQDRTCPYHPPAAYDPLRAARPLARITLFDGRPAWL

VTGHAAARRLLADQRLSTDRTRDGFPATSARLAAVRERRTALLGVDDPEHRAQRRMVLPE

FTLKRAGALRPSIRRIVGERLDAMIAQGPPADLVTAFALPVPSMVICALLGVPYADHEFF

EEQSRRLLRGPLPADTRDARDRLEAYLGELIDRKRRAPGEGLLDDLVRRQASEGATDREQ

LIAFAVILLVAGHETTANMISLGTYTLLTNPGRLAELRADPALLPGAVEELMRVLSIADG

LLRMATEDIDVDGQTIRAGDGVVFSTSVINRDESVYPEPDALDWHRPARHHVAFGFGIHQ

CLGQNLARAELEIALESLFDRLPTLRLAAPADEIPFKPGDTIQGMLELPVAW

>CYP102B1(637265023)SCO

MAQTAREPARDGLPKGFRSAELGWPELHRIPHPPYRLPLLGDVVGASRRTPMQDSLRYAR

RLGPIFRRRAFGKEFVFVWGAALAADLADEARFAKHVGLGVANLRPVAGDGLFTAYNHEP

NWQLAHDVLAPGFSREAMAGYHVMMLDVAARLTGHWDLAEASGRAVDVPGDMTKLTLETI

ARTGFGHDFGSFERSRLHPFVTAMVGTLGYAQRLNTVPAPLAPWLLRDASRRNAADIAHL

NRTVDDLVRERRANGGTGGGTGSGSGSGDLLDRMLETAHPRTGERLSPQNVRRQVITFLV

AGHETTSGALSFALHYLAQHPDVAARARAEVDRVWGDTEAPGYEQVAKLRYVRRVLDESL

RLWPTAPGFAREAREDTVLGGTHPMRRGAWALVLTGMLHRDPEVWGADAERFDPDRFDAK

AVRSRAPHTFKPFGTGARACIGRQFALHEATLVLGLLLRRYELRPEPGYRLRVTERLTLM

PEGLRLHLVRRTAAAPAPGRRTAAPGAADDAGDTVSAPGCPVHRAGD

>CYP158A2(637265428)SCO

MTEETISQAVPPVRDWPAVDLPGSDFDPVLTELMREGPVTRISLPNGEGWAWLVTRHDDV

RLVTNDPRFGREAVMDRQVTRLAPHFIPARGAVGFLDPPDHTRLRRSVAAAFTARGVERV

RERSRGMLDELVDAMLRAGPPADLTEAVLSPFPIAVICELMGVPATDRHSMHTWTQLILS

SSHGAEVSERAKNEMNAYFSDLIGLRSDSAGEDVTSLLGAAVGRDEITLSEAVGLAVLLQ

IGGEAVTNNSGQMFHLLLSRPELAERLRSEPEIRPRAIDELLRWIPHRNAVGLSRIALED

VEIKGVRIRAGDAVYVSYLAANRDPEVFPDPDRIDFERSPNPHVSFGFGPHYCPGGMLAR

LESELLVDAVLDRVPGLKLAVAPEDVPFKKGALIRGPEALPVTW

>CYP157C1(637265857)SCO

MTPESHSPTGTGEPLLEPPPGCPAHGLGPGGLHRLHEAEDLEELYEKLREQHGPVAPALL

HDDVPMWVVLGHAENLHMVSTPSQFCRDSRIWTPLNEGMVKPDHPLMPHIAWQPICSHAE

GDEHKRLRGAVTSAMSDLDYRELRRHIKRYTQRVVNRFCEEGRADLVSQFAEHLPMGVMC

HLLGMPEEYNDRLVEAARDTLKGTETAIASHAYVMEALGRLTATRRADPADDIAGRLVTH

PAGLTDDEVREHLRVVLLAAYEATVNLIGNVMRVVLTDPGFRAQLSGGQMTVPQAVEQSL

WDEPPFSTVFAYFAKQETELGGQRIRAGDGLLLGIAPGNVDPRIRPDLDASMMGNRAHLA

FGGGPHECPGQDIGRAIADAGIDALLMRLPDLQLDCDEDDLRWRSSIASRHLVELPVRFE

PRAQQDIMQQPSHAPTPERHAPWHVGLPKPERRAQPPLPAQPPQPVSVTAAEPQQAPGAG

QPRPRGAWQRFLLWWRGY

>CYP156A1(637267129)SCO

MTLPSTETAPTGEPGRIALYAPEFAADPHAAYRSMRRTHGPLVPVDLAPGVPATLVIGYY

QARRILNDPLRFPADPRAWEKLIPATCPVRPMMEWRPNALRSGGAEHTRYRSANTHAIDQ

VDQHGLRALVEQVASDAIEGFRTAGSADLLTQYSFPIAFRVLSALLGCPDEIGQRIADGM

AKIFDTTNADQGNLILAQAVSDLVTLRRTHPGDDITSRLALHPVRLTDEEMSHQLVTLYG

AGIEPMTNLISNTILKILTDEEFSADLHAGLSTVRDALDAVLYTDPPMANYCISYPPYPV

DVEGVLLPADQPVVISMAAANNDPALTEGVPAGQHGGNRAHLAWSTGPHTCPARSHAYLI

AETAVTHLLDALPETDLARPAAELVWRPGPFHRALESLPVTFPAAQSAAH

>CYP154A1(637267130)SCO

MATQQPALVLDPTGADHHTEHRTLREGGPATWVDVLGVQAWSVSDPVLLKQLLTSSDVSK

DARAHWPAFGEVVGTWPLALWVAVENMFTAYGPNHRKLRRLVAPAFSARRVDAMRPAVEA

MVTGLVDRLAELPAGEPVDLRQELAYPLPIAVIGHLMGVPQDRRDGFRALVDGVFDTTLD

QAEAQANTARLYEVLDQLIAAKRATPGDDMTSLLIAARDDEGDGDRLSPEELRDTLLLMI

SAGYETTVNVIDQAVHTLLTRPDQLALVRKGEVTWADVVEETLRHEPAVKHLPLRYAVTD

IALPDGRTIARGEPILASYAAANRHPDWHEDADTFDATRTVKEHLAFGHGVHFCLGAPLA

RMEVTLALESLFGRFPDLRLADPAEELPPVPSLISNGHQRLPVLLHAG

>CYP107U1(637267347)SCO

MTGSSSAPVPELFSWEFASDPYPAYAWLREHAPVHRTRLPSGVEAWLVTRYADAKQALAD

PRLSKNPAHHDEPAHAKGKTGIPGERKAELMTHLLNIDPPDHTRLRRLVSKAFTPRRVAE

FAPRVQELADGLIDRFADTGSADLIHDFAFPLPIYAICDLLGVPREDQDDFRDWAGMMIR

HQGGPRGGVARSVKKMRGYLADLIHRKRAALPPEPAPGEDLISGLIRASDHGEHLTENEA

AAMAFILLFAGFETTVNLVGNGTYALLTHPEQRERLQTSLAAGERGLLETGVEELLRYDG

PVELATWRFATRPLTIGGQDVAAGDPVLVVLAAADRDPERFTDPDTLDLARRDSQHLGYG

HGIHYCLGAPLARLEGQTALATLLTRLPDLRLAADPAELRWRGGLIMRGLRTLPVSFTPP

ASSAGNGPSPTQK

>CYP107P1(637267888)SCO

MTAATDGPHVSGPAFDPWDPAFVADPYPAFAELRARGRVLYYEPSDQWLVPHHADVSALL

RDRRLGRTYQHRFTHEDFGRTPPPPEQEPFHTLNDHGMLDLEPPDHTRIRRLVSKAFTPR

TVERLKPYVHGLADDLVARLVAAGGGDLLTDVAEPLPVAVIAEMLGIPESDRAPLRPWSA

EICGMYELNPSEETAAKAVRASLDFSDYLRALIAARRKEPGDDLISGLIAAHDEDDRLTE

QEMISTCVLLLNAGHEATVNATTNGWLALFRHPDQLAALRADHSLVPSAVEELMRYDTPL

QLFERWVLDEIEIDGTTLPRGAEVAMLFGSANHDPAVFTDPERLDLTRRDNPHISFSAGI

HYCIGAPLARIELAASMTSLLKRAPGLRLAAEPERRPNFVMRGLTELRVEL

>CYP107T2(637268023)SCO

MGSAPRLIVSLTGGPMTAPTYEELAALRAVGAVHRVFVPGSGESRLVVTRDAARAALTDP

RLRNDIRHSASWDSDGGHAIGHNMLQSDPPQHTRLRRLVAGHFTPGRTAALRPRVERIAH

DLLDALPPAGTADLVARYALPLPVTVICELLGVPESDRGTFHTWSNELVMPTSPEAAGSA

ATALTGYLTELTDAKRRTPDGTLLGDLVAAADSGELTPGELLGMAFLILVAGHETTVNLI

SATVHGLLTHPGQLARLRAEPELTEAAVEESLRYHSPVHASAFRFAAEPLELAGTAIAAG

DPVLVSLAAASRDPAHFPDPDRFDIGRRPRGHLGFGHGPHHCLGAPLARVEAAVAVRLLL

DRHPALALAAGPATLTWRTSTLLRGLVELPVRLG

>CYP170A1(637269500)SCO

MTVESVNPETRAPAAPGAPELREPPVAGGGVPLLGHGWRLARDPLAFMSQLRDHGDVVRI

KLGPKTVYAVTNPELTGALALNPDYHIAGPLWESLEGLLGKEGVATANGPLHRRQRRTIQ

PAFRLDAIPAYGPIMEEEAHALTERWQPGKTVDATSESFRVAVRVAARCLLRGQYMDERA

ERLCVALATVFRGMYRRMVVPLGPLYRLPLPANRRFNDALADLHLLVDEIIAERRASGQK

PDDLLTALLEAKDDNGDPIGEQEIHDQVVAILTPGSETIASTIMWLLQALADHPEHADRI

RDEVEAVTGGRPVAFEDVRKLRHTGNVIVEAMRLRPAVWVLTRRAVAESELGGYRIPAGA

DIIYSPYAIQRDPKSYDDNLEFDPDRWLPERAANVPKYAMKPFSAGKRKCPSDHFSMAQL

TLITAALATKYRFEQVAGSNDAVRVGITLRPHDLLVRPVAR

>CYP156B1(637270588)SCO

MDATTPAVPPPGCPAHADARIPLYGPDFAADPHAYYDYARSYGPSAPVELAPGVDASLVT

DYATALRLLQDNGTFRKDARRWKAFNEGLIPADSPVVPLLAYRPNAMFSDGAEHLRLRQA

ITDAMARIDTARLARSTEQISDYLISQFGSRGSADLMADYAKQLPLFVFNELFGCPADIG

DRILFGISGMFDGVNAERAAEVLFGAVGELVALKRSRPGEDVTSWLMQHETRMTDEEMVY

QLSLILGAGADPLRNLIGNTLHRILIHDEYARQGGLIDEAMEDTLWENPPVPNLAPHYPA

ADVEFAGQKFEAGELIMVSFAAANNSPSLAAARQSGSNRSHLAWSAGPHACPSKDPARQI

TMAAVENLLNRIPDIAAAVPEDSLTWRPGPFTRGLTALPARFTPLPAPDRTPAPAQAPAG

ERAEQSGTARKAAGTGRWSQFLNWLTR

>CYP158A1(637271270)SCO

MTQETTTLTGQSPPPVRDWPALDLDGPEFDPVLAELMREGPLTRVRLPHGEGWAWLATRY

DDVKAITNDPRFGRAEVTQRQITRLAPHFKPRPGSLAFADQPDHNRLRRAVAGAFTVGAT

KRLRPRAQEILDGLVDGILAEGPPADLVERVLEPFPIAVVSEVMGVPAADRERVHSWTRQ

IISTSGGAEAAERAKRGLYGWITETVRARAGSEGGDVYSMLGAAVGRGEVGETEAVGLAG

PLQIGGEAVTHNVGQMLYLLLTRRELMARMRERPGARGTALDELLRWISHRTSVGLARIA

LEDVEVHGTRIAAGEPVYVSYLAANRDPDVFPDPDRIDLDRDPNPHLAYGNGHHFCTGAV

LARMQTELLVDTLLERLPGLRLAVPAEQVAWRRKTMIRGPRTLPCTW

>CYP154C1(637271686)SCO

MTTGTEEARIPLDPFVTDLDGESARLRAAGPLAAVELPGGVPVWAVTHHAEAKALLTDPR

LVKDINVWGAWRRGEIPADWPLIGLANPGRSMLTVDGAEHRRLRTLVAQALTVRRVEHMR

GRITELTDRLLDELPADGGVVDLKAAFAYPLPMYVVADLMGIEEARLPRLKVLFEKFFST

QTPPEEVVATLTELASIMTDTVAAKRAAPGDDLTSALIQASENGDHLTDAEIVSTLQLMV

AAGHETTISLIVNAVVNLSTHPEQRALVLSGEAEWSAVVEETLRFSTPTSHVLIRFAAED

VPVGDRVIPAGDALIVSYGALGRDERAHGPTADRFDLTRTSGNRHISFGHGPHVCPGAAL

SRMEAGVALPALYARFPHLDLAVPAAELRNKPVVTQNDLFELPVRLA

>CYP157A1(637271687)SCO

MSTDAHDVPGAVPLGGPRFQTDPALLYRQMRREHGAVTPVVLDGDVPAWLVLGYRELHQV

TGDPVLFSRDSDLWNQWENIPDDWPLLPMIGRRQPSILYTVGERHRERAAMISDALEAVD

PHLLRGHAERFADELVDRLCAKGEADLVGDYAMLLPVRVLARLYGFPDEQGPALVTALND

MIDGRERALAGQTHLGTSMARLLADRKAAPADDVASRMLADESGFTEEEVAQDLMVMMAA

GHQPTADWIGNSLRLMLTDDRFAASLFGGRNSVAEAMNEVLWEDTPTQNVAGRWAARDTQ

LGGRRIRAGDLVLLGLQGANSDPQVRTDGSALTGGNNAHFSFGHGEHRCPFPAQEVAEVI

ARTGIEVVLDRLPDIDLAVPAGSLTRRPSPWLRGLTELPVRFTPTTALGGTSA

>CYP155A1(637271713)SCO

MADPGDHVVEPGGRRGRTAGVGGGHVVQDGRVSARKADRGRAGPACPVDRAADGTWRVHD

FAVARALLRGPGTVQAGLGIETVEKLPPRVRRPVLYRDGPEHREHRRQTARYFTPRRVDE

HYREPMVRIAEEQLAVLRSAGEAPLSDLAFGLAVGVVSEVVGLRYSRPGIRRRLERFFPE

EFGEPGLTSVRGLYWLVRQNTNWLRIHLADVRPAVRAHRRREHDDLISHLIAEGCSDVEI

LGECLTFAAAGMVTTREFVCLAAWHLFSDAELLGHYRSADETGRLAVLQELLRLEPVIGS

LRRRATGPVELSCRDGPVTVRPGEYVEVHLDDANADPKAVGEEPLLVRPERAGAVGAGLS

FGDGPHRCPGAHIALLETDVFLSRLFALDGVRMSGGPRVAFQEAIDGYEIRDLTVALPRA

GRG

>CYP105N1(637271954)SCO

MTPPESPTASHTPGATPPRDFPIQRGCPFAAPAEYAALRTDDPVARVTLPTRREAWVVTR

YDDVRELLSDPRVSADIRRPGFPALGEGEQEAGARFRPFIRTDAPEHTRYRRMLLPAFTV

RRVRAMRPAVQARVDEILDGMLAAGGPVDLVSAYANAVSTSVICELLGIPRHDLEFFRDV

TRISGSRNSTAEQVSEALGGLFGLLGGLVAERREEPRDDLISKLVTDHLVPGNVTTEQLL

STLGITINAGRETTTSMIALSTLLLLDRPELPAELRKDPDLMPAAVDELLRVLSVADSIP

LRVAAEDIELSGRTVPADDGVIALLAGANHDPEQFDDPERVDFHRTDNHHVAFGYGVHQC

VGQHLARLELEVALETLLRRVPTLRLAGERDQVVVKHDSATFGLEELMVTW

>CYP107F2(2555755236)SCI

MPEDETACPDSGRRPSGCPDRGADRGPDSGADRGQGPERSGTDAIGCPFDFSEDLAFDPA

LADLMEQGPVTRVRLPHGDSEAWLVTSYNGVQQVTTDPRLSRAAIVGRDYPRLTPEPIVS

PESINVIDPPESQRLRRAVTQAFTKPRVRRMRPAIEQVTGALLDEMAAHGPPADLVTHLS

LKLPHHTICELLDVDRADRALLLEYTHRMLTTAPGQKQESADAKRHLRAYFGRLVRQRRD

RPGEDLISTLAAASDEPLSDDELAVLAMTLLLSGNDTATCQISNISYTLLTQPHWWDLLV

AHPERLPEVLDELLRIIPFRKGVGIPRLALADVEIDGTLVRAGDFVHVSYLTANRDPEVF

ARPHAFDPDRPSRPHMTFGWGGHHCVAAPLAMEELQVALGALLTRFPGLRLAVPSSELRW

DTETIRRFPLELPVTW

>CYP107CQ1(2555755438)SCI

MTGTFDWTDVEFDLSDVNFVTDREAGERWTGAPRPVCPGRFSDGADVYVVTRYEAVRSLF

ADRRVSNHPPEGVHLDSMRRRGVPEELLKYFDSTIMTMVPEDHRRVRSLIDRAFSVRRVK

SLRPRIERLADQLLDQMDPEGETDLVAGYAHPISTTVICELLGVDDEYRDQWLKWSEAFT

TFVRPDPEILPPALHGMVDTVIRLIGARRAQPGDDLISDLVQISDETEKLDEVELVALVL

VLVQAGLDTVRHSISLSFFNLLVHPDQLELVKSHPENTVQAVRELMRYSGPIKMALPRFA

AEPIEIDGVTIPKDGQIQLVVGAANNDPERFTDPRVLDVTRADNPQLSFAAGDHFCPGAS

LATAETEIALNKLFARYPDVRLAADPDEVGPRFLKAVTRLPVQLV

>CYP105AJ2(2555755445)SCI

MSETVRPVDGLPMTRGTCPFDPAPELAELREEQPVARMVFPDGHLGWLITGYDEVRRLLA

ARGMSSRGDLLRTPIPLPMAGNRTELAPGMFTAMDPPEHTHYRRRLTAWFSARRTRTMEP

RLTEHVDLYLGRMIEEGGPTDLVAAFAEPVAGLVICELLGVPADRRDVFVKGIKALLTVH

SSAEEAIAGWQNVGGQLMELIRAKREEPTDDLLGTLVSDGAFSDEELATIGSVLLVGGYD

TSKNMIALGTFALLAHPDQYAALAADPGLGAGAVEELLRYVTVMHAGSIRAAGADMDFDG

HHFTEGDAVSLSLAAANRDPSLCEDPDRLDITRPPVAHLSFGYGIHQCVGQQLARLELRI

AFEGLARRLPGLRLAIPEDQVRTNPESIIYGVHELPVTW

>CYP105BB1(2555755509)SCI

MLDWPLARVCPLSPPPALGVVRDGPPTLVRLPAVAGEEQLAWLITRHEDVKAALKDPRLS

ADETRPGFPLRIPVPTDDRPSGFLRMDDPEHGRLRRMVAPEFTARRVRMLRPGLQELTDR

AADALAAGPQPADLVRDFAAHVSALVIARLLGIPDEHTAFFLEQTRILLTDGDPALSLAA

HHRIIAFLDETARAKEKQPGDDLVSRLVTNHVATGELDRADLLGILKLLLIAGHESTATQ

IAFSALSLLTDDGLRAEVLADGGALLPQFVEESMRFWSIIQDNVVRQATEDVRIGETLVG

AGESVMISLLAANHDATVFPRPERLDIHRDASDHLLWGHGAHFCLGASLGRLEVTLALGS

LFTTLPTLRLACEVGQLRVREHPVFHSLTELPVVW

>CYP1038A6(2555755680)SCI

MDTEAGLGSLPQLPGAERPVPVPEAGPEVVERWRARGGELVDLLSEVRARAGGVAAFRLG

PAPTVLVTDPQAVQHVLARRPERYVKRSHRARLLVGDGVLSATGRAWKTQRRLLQAQFTG

TGMRRYEQRIDAAARTTADRWREFARTGRTLDVAREMRRFALDAIWRSLTGHALDDGTER

ELTAVEAVAAALPTLPADASDARDAVAADLARIDAVARHAIERARDGSAGPDGPGLLHVL

TDAAGERPEYTDRLIRDELVTLLVAGHETTATTLTWLHLLLDRHPGARADALAAGSEGSE

RRRQAVQALIHETLRLYPSAWILPRHAAEDDVLEGHTVEAGTDILVCPYLTHRDPELWPD

PEHFAPRRFLAPGGRPTHPGAYFPFGLGPRACLGMQFAVRETTVLLEHLLPYTPAFASLP

TKTDYGITVRPAGPTPATLRPPRT

>CYP158A18(2555755799)SCI

MSLDPASNPPAPAVRPWTVDDLPALEPDPFLDEVLREEPITRIALPHGSGHAWLVTRYED

VRFVTSDPRFSREEVVGRDVTSMAPQAVASQTAGLQYIDPPRHTRLRRVVARAFTARGME

RLRPMAERTAHRMLDAMEKAGPPADLMEHLHTPFPIAVVCFFLGADEDDWHQWAGNSEAL

LSQSTDAERNRAARMATRGRVVDLLRRRRDEPRDDLAGVLAQAAEAGEITDDEAVSLAMA

VYVSGGHAVRNNSGSMMYALLTHPEQLEQLHADPALLPRAVEELFRFVPHRNGVGIPRIA

LEDVELGGHLIRAGEVVYNAYVAANRDPGVFPDPDALDFGREGPGHLAFGHGPHFCLAAL

MARMEAEVMIRAVLDRFPGLRLAVPADEVEFQREGLIRGPRTLPVTWWDDRA

>CYP154B5(2555755806)SCI

MENTACPYALDLTGQDHMAEAAHLRAKGPAVQVELPGGVLAWAVVQQRYVERLLTDPRVS

RSARLHWPPFIEGKITEEWPLYPWVANENMLFAYGEHHTRLRRLIAGAFTARRSEALRPR

VQELSAELLGELAALPPGTPVDLRTAYAEVLPLRVICELFGVPRGAETDALSAALSTVFS

STVSGAEMESARLEAFGRLAALVKAKRERPGDDLTSSLIAARDEGDRLTEDELLGSLFMF

IAAGQDTTATLITNAAGALLTHPEQLAHLRAGRAGWADVVAETMRVHTPGAYAPMRFAVE

DIDLDGVRISKGDCILVNFAAGGHETDRHGSDAHRFDLLRGTDRDILGFGHGPHRCLGAP

LGEIEAASALSRLFDMFPEAQLACAPEELAPLPTFMLNGFRSLPVVLRPAAS

>CYP156B8(2555755826)SCI

MHQRPGGPPPGCPAHGNTQLYGPEFGSDPDARYAYLRGLGPSAPVDIAPGVEVELVTSYD

AALYILQNPASFVRDSRRWRALNEGRVPEDSPALPMMGYRPNALFSDGAAHARLRQAVTD

SLATVNELQLVRQTQQSAEYLISRFSSDRLGQAELMAEYAQPLPLLVFSELFGCPPEIGD

RVIVGISGIFDGTAGADLVLGEALGELIALKRRRPGTDLTTRLMEHPSQLTDEEVLHQLV

TLLSGGTAPLSATIGSSSALILGEEWQTGLPVEDAVTQVLWNYAPIANYAAHYPTHDVEL

GDRVLRANDPVVISFAAANTDPRLAEHREQLSAKAHLAFGAGPHACPAKDPAFVIAVTAV

ETLLNRLPDVEVRVPFKDLNWVPAPWSRSLVALPVRFTPRAVAPSAAQAPVSEPQPAVPS

AYHVAPQASRPAAANASRPKPGLFSRFLAWTRGE

>CYP1035A3(2555755827)SCI

MFSRLRTAKGQANPFPLYAELRSGGGVVAAPWGGHLVTGFDLCDQVLRSREWLEPDTRWR

EEQGDGTRWTAPSSREMSRTLPALNPPDHTWVRRSAGMFDRASLEDLRGTVGRITGRLLD

TLAARLDEGEADFNALVSEELPVATIGHWLGLPTADFARLRDLTHDQVFTQELLPSASQL

AKSDAATAQLRAYFTELVRERRARPGDDPVSRWIAVWDTLESDQDKADEGVYFLALFVLL

AALETTSTLLSTATLLLLEHPRQWDWLTVHPDLVPAAVEESLRYDPPTHVISRVCAQDRL

LGGVEVRKDEMVHLMVAAAHRDPAKHADPELFNLHRKPAHLAFSGGIHYCLGAPLARLEA

QTLLHQLVRRFPRLTLVRRPSWAPRVAFRRLLNLDVALS

>CYP1059A1(2555756066)SCI

MSDGKILFRGTLDERTVSRSSAEPAATVVPMRMGTAGVGLLVNDHGEATRILSDVETYGS

AGELMASVFDEPAESHPDAASEAVFAVNQTDGEEHRRVRRALRRVIDARVRDVSDQLIQD

VQQSIESALALGRLDAVASIGVPVADSVMSRLLGLDANLVSALRNAVYQGYPVRELDATL

VDWVRDRRAHPADDLVSDLMAELPSLDDRQVAANTRLLALSGVEVLAALITNGILCLAQD

PGTQSLVREDETLLPGLVHEVQRYASPIARGIYRMTKRSSVIGKYSVPAGTLLVIGVDVC

NRDTSAFPDAHRFDPTRGRDFEHLTFGRGRHSCLGIPVTRLIAMQALRMFLSGTTRFGLT

VEPSELRFHETMVMNGLVELPIWVECAD

>CYP105B21(2555756070)SCI

MTRHAEQCALLSDRRVSADWMQPGYPFHSQFLRDHHEEGQFLTAMEGPEHLRLRRMIARP

FTPRKVEQLRPTVRQVVDDHIDALLAGPKPADFVADFALPIPSITICHVLGVPYEDHDFF

QHAIRTMTGSDVPDDAVNKSQGELYHYLAKLIGRKLAAPEDDLLSQLATERLATGQLNRH

QLVMLVLFLLISGHETTASMIALGTLALLQHPDQIAVLKDSDGPQVAAAVDELLRYLTTV

QPGRRRVAVEDIEIAGQVIRAGEGLILPEEIGNRDDSVFPDAGKLDLRRDAGQQLAFGFG

VHKCTGQPLARLELQVVFAALFRRIPTLTLAADLADLPFMDDGLGYGVKKMPVSW

>CYP158A2(2555756373)SCI

MSAETLTEPVGEALPPVRHWPAVDLAGTDFDPVLTELMSEGPLTRVQLPNGEGWAWLVTR

YDDVRMVANDPRFSREAVMGRQVTRLAPHFIPDRGAIGFLDPPDHTRLRRAVAAAFTSKG

VERIRGKARVMLDELVDELLQDGPPADLTAAVLSPFPIAVICELMGVPAADRHAMHTWTQ

LILSSSHGKDVSEKAKREMSAYFSDLVGLREGSTGEDVASLLGAAVGRAEITLEEAVGLA

VLLQIGGEAVTNNSGQMFHLLLSRPALVEWLRSDPGIRPRAIDELLRWIPHRNAVGLSRI

ALEDVDVHGVRIRAGDAVYVSYLAANRDPEVFPNPEVIDFSRSPNPHVAFGFGPHYCPGG

MLARLESELLVDALLDRVPGLKLAVPPEQVPFRKGALIRGPEALPVTW

>CYP147F13(2555756448)SCI

MTQAPLFRQITDFANRADPYPLYTELRRTPVLHEEEGGPYVVSSYYDIEALLHDPRVSSD

AANLAASEDDGLGMGDETGGLPPSFLRLDPPEHDRLRRIANSAFGPPHQPRRIENMRGEL

DGIVTGLIDGFGDAREVDLVDQFAYPFPVTVICRLLGVPREDEPRFRSWVDPLVATLDPD

TRRSADPEFVKTAQESRMQLGMYLAGLVEQRTKEPQDDVLSDLANSRGPDGAMTMMEVLS

TAVLLLIAGHETTVNLITNGMLTLLRHPEVLGRLREDPGLSVRIVEELLRYEPPVQIVPQ

RTCITDIELRGTTIPKGSRIWLMIAAGNRDPERFKEPDRFDPDREDIQHLGFGSGIHSCF

GAPLARLETQIALSELARRLENPRLVEDPPPYRPNAVLRGPRHLNVAFDGLR

>CYP157C19(2555756799)SCI

MTPEQQSLTGTDPTSGPPPGCPAHGLGPGGLHRLYGPDAENLDDLYERLREEHGPVAPVL

LHDDVPMWVVLGHAENLHLVRSPSQYCRDSRIWTPLQEGMVKPDHPLMPHIAWQPICSHA

EGDEHQRLRAAVTGAMSTIDHRSMRRHIGRHTQELVNAFCERGRADLVPQFAEHLPMAVM

CEILGMPEEYNDRMVQAARDALKGTETAIESHGYVMDALSRLTTRRRARPDDDFTSHLIT

HPAGLSDDEVREHLRLVLFAAYEATANLLANALRMVLTEPGFRAQLNGGQMTVPEAIEQS

LWDEPPFSTVFGYFAKQDAELGGQRIRKGDGLLFAPAPGNVDPRVRPDLSANMQGNRSHL

AFGGGPHECPGQDIGRAIADVGVDALLTRLSDIRLDCPEEDLRWRSSIASRHLVALPVRF

EPKPQQDVGLPPSASAVPPQRSTWQVGTAHTDPAPAPGPRPVAPPPVPVDPAPAAEPARR

ETLWHRLLRWWRGD

>CYP125A23(2555757571)SCI

MHCPALPDGFDFTDPDLLQSRVPLPEFAELRRAEPVRWIPQSQGLAGFADEGYWAVTRHA

DVKYVSTHPELFSSTLNTAIIRFNEHIERDAIDAQRLILLNMDPPEHTRVRQIVQRGFTP

RSVRALEDRLRARAEGIVTAARARSGPFDFVTEVACELPLQAIAELIGVPQEDRSKIFDW

SNRMIAYDDPEYAITEEVGAQSAAEIIAYAMNMAAERKRCPAHDIVTTLVAAEDEGNLNS

DEFGFFVLMLAVAGNETTRNAITHGMHAFLTHPAQWDLFKRERPATTAEEIVRWATPVNA

FQRTATQDTELGGARIGKGDRVGLFYASANHDPEVFASPDAFDITRDPNPHLGFGGGGPH

FCLGKSLAVLEIDLIFNALADAMPSLRLAGEPRRLRSAWINGVKELRVTTG

>CYP147F14(2555757880)SCI

MTTTETDTLRRILDYSSRADPYPLYAELRETPVARQRDGSYVVSTYREIAGLLHDPHISS

DVRNLARPMPSHAGESTPAFINLDPPEHDRLRRMAMRHFGPPHTPGLVTGMEDDLTAVVT

TLIDGLEDKERIDVVDDFAYPFPVAVICHLLGVPREDEPRFHLWVDALINSIDYNPETDP

KEKLENGLRARKDLREYLGGLLQRRHGKPGEDLLSRMANDDGPDGRMTDEQIVATANLLL

IAGHETTVNLITNGMLTLLRHPDVLRRLREDPDLVVPLVEELLRYEPPVHIIPWRAAYSD

ITVAGTLIPKGAQVMLMLASGSRDPVRFHEPQRFDPDRRDNQHLGFGSGIHLCFGGPLAR

RETQIALTELVRRLDRPQLVTDPPPYRRSPILRGPIHLEVTQGG

>CYP107U1(2555758356)SCI

MTDQPHPPTAPELFTWEFASDPYPAYAWLREHAPVHRTRLPSGVEAWLVTRYADAKQALA

DQRLSKNPAHHDEPAHAKGKTGIPGERKAELMTHLLNIDPPDHTRLRRLVSKAFTPRRVA

EFAPRVQELTDQLIDRFAATGSADLIHEFAFPLPIYAICDLLGVPREDQDDFRDWAGMMI

RHGGGPRGGVARSVKKMRGYLAELIHRKREALPDTPAAGEDLLSGLIRASDHGEHLTENE

AAAMAFILLFAGFETTVNLIGNGTYALLTHPGQRRRLQDSLALGERALLETGVEELLRYD

GPVELATWRFATRPLTVGGQDIAAGDPVLVVLAAADRDPERFADPDVLDLSRRDNQHLGY

GHGIHYCLGAPLARLEGQTALATLLTRLPDLRLAADPAELRWRGGLIMRGLRTLPVEFTP

VP

>CYP107P2(2555759179)SCI

MAAPRDLAFDPWDPAFLADPYPAYAELRSRGRVIRYEPTDQWLVPHHADVSALLRDRRLG

RAYQHRFTHEDFGRTPPAPEHEPFHTLNDHGMLDLEPPDHTRIRRLVSKAFTPRTVQALV

PYVRNLANELVAGLVAAGGGDLLTDVAEPLPVAVIAEMLGIPESDRAPLRPWSADICGMY

ELNPSGDSAERAVRASVEFSDYLRELIAERRENPGDDLISGLIAAHDEGDRLTEQEMIST

AVLLLNAGHEATVNATVNGWWALFRNPDQLAALRADHSLVPAAVEELMRYDTPLQLFERW

VLDDIEIDGTTIPRGAEIAMLFGSANHDPSVFTAPERLDIGRGDNPHISFSAGIHYCIGA

PLARIELAASMTALLERAPTLSLAAEPERKPNFVIRGLEGLDVEVR

>CYP170A10(2555760093)SCI

MTVESVKPVTPRTPELREPPLAGGAVPGLGHGLKLVRDPLAFMSGLREHGDVVRLKLGPK

TVYAVTTPALTGALALSPDYRIDGPLWESLEGLLGEEGVATANGPRHRRQRRTIQPAFRL

DAIPAYGPVMEEEAHGLTERWKPGATVDCTAESFRVAVRIAARCLLRGEYMDERAERLSA

DLATVFRGMYRRMVIPLGPLYRLPFPANREFNRALADLHLLVDEIVAERRASGQKPDDLL

TALLEAKDDNGDPIGEQEIHDQVVAILTPGSETVASTIMWLLQVLAEHPEHAEKVRAEVE

SVTGGRPVGFADVRELRHTNNVVVEAMRLRPAVWILTRRAVTDTELGGYRIPAGADIVYS

PYAVQRDARSYDRHLDFDPDRWLPERAKEVPKYAMSPFSVGNRKCPSDHFSMAQLSLITA

AISAKYRFEQVGGSDDSTRVGITLRPHRLLLRPLPW

>CYP105BC1(2555760778)SCI

MSECEQQLPEWPMPRSCPYAPPDAYEGLRKDPPLKVRIRGGEAWLVTRHADVRQVLNDNR

FSADDQKPGFPIRIQLPPEPGVMSFNRMDGSEHGRLRRMAMTEFTARRTRALRPEVELLV

ERLLDELERGPRPVDLVEHFAVRLPSLVIARMLGVPEEDEATFTEQSRVILSQDATPEET

YGAFVEMTGYLDRLAARRTAEPQDDLISRLVTRYVATGELTHQELVAMARFFLVAGHETS

AHQISLSVLSLLRDPALLAELRADPGLYKPAVEELLRYWSISQDNQVRAAVADVDLGGAR

IRAGEGVIVAIPGANHDESVYPDAGRLDIHRNASGHLAFGFGAHLCPGASLARMELEVCL

SMLFERFPTLRLALPAEDVRFRQNTLVYGLEELPVTW

>CYP105AH2(2555760779)SCI

MSLADHTDILDWPFARTEDGAPPPLLAELRKAPPCVVRIPAGAAESRLAWLVTRYADVRQ

ALADPRLSADETLPGAPVRIQVPPGGNPSSFLRLDDPEHARLRGMIQTEFTARRVKRLRE

PVQRLVDELLDELAAQPQPADLHAVFSRALPTLVIARLLGVPEEDSPFFIEKTRVTISQE

DPAVSQAAFVEMSEYLAKLALRKLADPGDDLISRLAVNHHATGALSLDELVGIARLVLVA

GHETTTNQIALNVLALLRDDDLRDRVAADDGALIPAFIEESMRYWSISQDAMVRLAVEDV

EVGGVTFEKGDAVVISVPAGNHDESVFACPHRIDPDRDTSGHLQWGFGPHYCQGAPLARL

EMELALRSLLRRFPNLRLAADPRTLFRRGTVFHGVTGLPVTW

>CYP180A6(2555760877)SCI

MSLREAPPVPDVFDPRRYAAGVPYADYRLLRDHHPVAWQDEPEVLGWPAGPGFWAVTRHA

DVVRVLKDSATFSSYVGATQIRDPDPDDLPFIRRMMLNQDPPVHGRLRRLVSRAFTPGRV

DRFAAAVRERARGLLARALEEARSGDGTVDVVAAVTDEYALLNLADLLGVPESDRGLLLH

WTQRVIGYQDPGEAGAPVLDGAGRPVDPRSPAMLRDMFAYARQLAVHKRRCPADDILTTL

AHDPELEGAELEMFFFLLTVAGNDTVRSAAPGGLLALAEHPGAYALLRAGKAAPAPALEE

LLRWHPPVLSFRRTAAVDTELAGRAIRAGDKVVVFHASANRDERVFAGPGRLDLARTPNP

HVSFGDGPHVCLGAHFARLQLRLLYEEVLRAVPGLRLAGPAGRLVSNFINGLKSLPLSVV

>CYP183G1(2555760907)SCI

MKTDSDPATRSWRIARAPGGLPLVGHAVQLARRPLEFLGSLPAAGDLVELRLGGRPAYLP

CHPELVQQVLRNARVYDTGGPVKDKARPILGNGLITSDWADHRRQRRMVQPAFHSARIAA

YAVVMREECAAQAETWRAGLPLDVADAMQALTARVTARALFSTEMAPHSVAEIQRSLPVM

VRGAFRRAMDPTGLVARLPLAANREFDAALARLHGLIDGIVGDYRRSGGDRGDLLSALLA

ARDEEDGGAMTDQEVHDQVMTLLLAGVETTASALTWAWHLLAAHPEAEARLHAEVDEVLG

GRAPEYADVPRLVHTQRIFTETLRLYPPAWMFTRMTTEPTELAGHRLPAGTDVLISPYVI

HRIPEFFARPDVFDPDRWLPERVQDVTRGSYLPFGGGSRKCIGDVFGMTEATLALAALAS

RWRLRPVPGTTISPRAEMSLTAGRLPMVPEPR

>CYP183H1(2555760908)SCI

MTDRSWRTGTAPGALPLLGHVPALWRRPLEFLASLPAHGDLVEVRLGPARAYLAAHPELV

RHVLLNPRLFDKGGVFDKARQLLGNSLSVSRGEEHRFQRRLIQPAFHPARIAAYTTAVAA

DTRTVTDAWRDGETRDIGDAMHSLLMRVAARTLFSSGLDEATVEEARQCLRTVSHGIYKR

TVAPMGVMEKLPTPANRAYDHANARLRQIVADMIAARRRSDEDHGDLLSTLLRAEHPETG

QTLDDGQVLDQVVTFLVAGSETTASTLAFVFHLMGRLPEVEKRVHAEVDAALGGRTPVHE

DLPALPYVRNVITETLRLYPPSWMAMRVAAEDVELGGRTVPAGTMILYSAYALHHNPGLF

PDPETFDADRWEGERAAQVPRGALLPFGAGSHKCIGDVLALTETALIVATVAARWRLRPV

PGSALRPEPKATLEPGPLPMVLARR

>CYP107L31(2555761042)SCI

MVDLGEFGDAFRSDPHPVYARLRERGPVHRIRPPGADPDYAAWLVVGYDEARAALADPRL

AKDFAKIDASFLDENLIGKHLLATDPPQHTRLRSLVTRAFTARRVELLRPRVQQITDELL

EEMLPRGRADLIDALAYPLPITVICELLGVPDMDRAEFRKMSTEVVAPSGGDAEYSAMTR

LAEYLTELIEDKRAGGPTGDLLSDLVRTTAEDGDRLSPVELRGMAFLLLIAGHETTVNLI

GNGVHALLTHPGQLAALRADRSLLDGAVEEMLRYEGPVQSATFRYAAESLEIAGTRIERG

EHVMIGLTAAQRDAGRYPDPDRFDIRRDTRGHLAFGHGIHFCLGAPLARLEGRIAIGTLL

ERAPGLALDGPPGEYLPGLLMRGMRTLPVRW

>CYP183A4(2555761117)SCI

MPQQTYVAGTAPGAVPVVGHALQMMRQPVRFMSSLSAHGDLVKIRIGPTEAYVPCHPGLL

RDVLTNDRTYDKGGVFYDRARDIAGNGLVTCPFKDHRRQRRLMQSAFQRSRLQRYATAMH

AEIEATTGRWHDGMVVDAFPELYGMALRTVARTLYSTPVSKELADGVERAFDTVLNGLFR

QMFLPRFLRRLPTPANRRYRRNLDYLHATTQRLIDDYRRDDAEHDDLLSALLASRDEDGG

RLDDTEIHDQVITVMAAGTETVAGTLTWIFHLLSRHPDVEAALYDEIDTVLGGRTPGWDD

LPALSLTDRVISETLRLYPPAWLFTRLTAADTELAGQSLPRGTTIVFSPAAVALNEEAYP

DEQRFDPDRWLPERVTPQARQAFMPFGTGARKCIGDLYARTEAALGLATILGRWRITCEP

DADVRPVPLATVYHPRRLRLRVTARTGRPTAAAPVLPGLGGDAA

>CYP158A6(2555761432)SCI

MTEDTSTLTGQAPSPVRVAGPTEEIVLPGHTPPPVRDWPPPDLDGTEFDPVLAGLMREGP

LTRIRLPFGAGWAWLATRYEDVRLITNDPRFSRTEVTRRQVTRLAPHFAPRPGSLAWADQ

PEHNRLRKPVAGAFTVSAMKRLRPRAQEILDGLVDAVVADGPPADLVERVLEPFPLTVVS

EVMGVPPADRARVHGWTRQIISTSGGAEAAGRAKTGLYDWIAAMVRDRAHSTGEDVYSLL

GAAVTRGEISEEEAVGLAGPLQIGGEAVTHNCGQMLFLLLTHPDLMERMRDRPPERGPVL

DELLRHIPHRSTVGLARIALEDVEVAGHRIAAGEPVYVSYLAANRDPDVFPEPDRIDPDR

AACPHLAFGNGPHYCTGAVLARLLTELLVDTVLDRLPRLRLAIPAEDVRWRHKTMIRGPL

TLPVTW

>CYP102B14(2555761641)SCI

MAETTEAGLPKGFRSAEQGWPALRRIPHPPRRVPLLGDVLGVDRHRPLQDSMRFARELGP

VFRRRVFGNEFVFLWGSRLVADLSDESRFAKHVGLGVANLRPVAGDGLFTAYNHESNWQL

AHDVLAPGFSREAMAGYHGMMLAVAARLTGHWDRELAAGRPVDVPGDMTKLTLETIARTG

FGHDFGSFERDRPHPFVRAMVGTLTYAQRLNSVPVPLAPLLLRGPARRNAADIAHLNRTV

DGLVAARRRSGGGDGDLLDRMLATAHPETGERLSAENVRKQVITFLVAGHETTSGALSFA

LYYLARHPEVAARARAEVDRVWGDTAEPGYDQVAKLRYVRRVLDESLRLWPTAPAYAREA

KQDTVLAGEHPMRRGAWALVLLPMLHRDREVWGEDAERFDPDRFEAGAVRARPPHTFKPF

GTGARACIGRQFALHEATLVLGLLLRRYDLRPEPGYRLRVTERLTVMPEGLRLRLERRPA

PAPAPVTVPDDGPGSAARCPVRGAGD

>CYP105D21(2555761682)SCI

MTELTDITGQATPAEPVAFPQDRTCPYHPPTAYDPLRDGRALARATLFDGRQVWMVTGHA

TARALLADPRLSTDRTRPGFPAPTERFAAVRDRRVALLGLDDPEHRTQRRMMVPSFTLKR

ATELRPAIQRIVDDLLDAMIEQGPPAELVSAFALPVPSTVICDLLGVPYADHEFFEERSR

RLLRGPTADDIQRARAELEGYLGDLIDRKARQREPGDGVLDDLVHQRLRTGELDRTDLVS

LAVILLVAGHETTANMISLGTYTLLRHPDRLAELRADPALLPAAVEELMRMLSIADGLLR

VALEDIEVAGTTIRAGEGVLFSTSVINRDTSQYEDPDTLDFHRSTRHHVAFGFGIHQCLG

QNLARAELEIALGTLLRRLPGLRLAAPADEIPFKPGDTIQGMLELPVTW

>CYP113K3(2555761715)SCI

MTYDEAQGQWRVVDHQGVSAVLADPATYSSDLTPITPTQEDFETFRQGNFVGMDPPEHRK

LRTLVSQAFTPRVVQGLEPRIEDVCARLLDGVADRDRFDLVDTLAYPLPIIVIAELLGIP

ADDHRLFQEWASTLFGGDQLGDSLDMADLERALEAIAPTVREMNGYVLDHIRRRRARPGD

DLTSRLLAAEVDGTRLADEEIVGFVALLLVAGHITTTALLGNAVVTFDGHPGTFPALRED

PGRLPDAVEEVLRWLPPFPELGRRTTRPVVLGGQEIPADTLLMVHLGAANRDPSRFTRPD

VFDVARSPNPHLTFGHGIHFCFGAPLARLEARIALHMLMERFPALTIPSYDDVTYQNPAV

LVGVRHLPVAVTRP

>CYP105BB1(2555761843)SCI

MLDWPLARVCPLSPPPALGVVRDGPPTLVRLPAVAGEEQLAWLITRHEDVKAALKDPRLS

ADETRPGFPLRIPVPTDDRPSGFLRMDDPEHGRLRRMVAPEFTARRVRMLRPGLQELTDR

AADALAAGPQPADLVRDFAAHVSALVIARLLGIPDEHTAFFLEQTRILLTDGDPALSLAA

HHRIIAFLDETARAKEKQPGDDLVSRLVTNHVATGELDRADLLGILKLLLIAGHESTATQ

IAFSALSLLTDDGLRAEVLADGGALLPQFVEESMRFWSIIQDNVVRQATEDVRIGETLVG

AGESVMISLLAANHDATVFPRPERLDIHRDASDHLLWGHGAHFCLGASLGRLEVTLALGS

LFTTLPTLRLACEVGQLRVREHPVFHSLTELPVVW

>CYP105AJ2(2555761907)SCI

MSETVRPVDGLPMTRGTCPFDPAPELAELREEQPVARMVFPDGHLGWLITGYDEVRRLLA

ARGMSSRGDLLRTPIPLPMAGNRTELAPGMFTAMDPPEHTHYRRRLTAWFSARRTRTMEP

RLTEHVDLYLGRMIEEGGPTDLVAAFAEPVAGLVICELLGVPADRRDVFVKGIKALLTVH

SSAEEAIAGWQNVGGQLMELIRAKREEPTDDLLGTLVSDGAFSDEELATIGSVLLVGGYD

TSKNMIALGTFALLAHPDQYAALAADPGLGAGAVEELLRYVTVMHAGSIRAAGADMDFDG

HHFTEGDAVSLSLAAANRDPSLCEDPDRLDITRPPVAHLSFGYGIHQCVGQQLARLELRI

AFEGLARRLPGLRLAIPEDQVRTNPESIIYGVHELPVTW

>CYP107CQ1(2555761914)SCI

MTGTFDWTDVEFDLSDVNFVTDREAGERWTGAPRPVCPGRFSDGADVYVVTRYEAVRSLF

ADRRVSNHPPEGVHLDSMRRRGVPEELLKYFDSTIMTMVPEDHRRVRSLIDRAFSVRRVK

SLRPRIERLADQLLDQMDPEGETDLVAGYAHPISTTVICELLGVDDEYRDQWLKWSEAFT

TFVRPDPEILPPALHGMVDTVIRLIGARRAQPGDDLISDLVQISDETEKLDEVELVALVL

VLVQAGLDTVRHSISLSFFNLLVHPDQLELVKSHPENTVQAVRELMRYSGPIKMALPRFA

AEPIEIDGVTIPKDGQIQLVVGAANNDPERFTDPRVLDVTRADNPQLSFAAGDHFCPGAS

LATAETEIALNKLFARYPDVRLAADPDEVGPRFLKAVTRLPVQLV

>CYP107F2(2555762116)SCI

MPEDETACPDSGRRPSGCPDRGADRGPDSGADRGQGPERSGTDAIGCPFDFSEDLAFDPA

LADLMEQGPVTRVRLPHGDSEAWLVTSYNGVQQVTTDPRLSRAAIVGRDYPRLTPEPIVS

PESINVIDPPESQRLRRAVTQAFTKPRVRRMRPAIEQVTGALLDEMAAHGPPADLVTHLS

LKLPHHTICELLDVDRADRALLLEYTHRMLTTAPGQKQESADAKRHLRAYFGRLVRQRRD

RPGEDLISTLAAASDEPLSDDELAVLAMTLLLSGNDTATCQISNISYTLLTQPHWWDLLV

AHPERLPEVLDELLRIIPFRKGVGIPRLALADVEIDGTLVRAGDFVHVSYLTANRDPEVF

ARPHAFDPDRPSRPHMTFGWGGHHCVAAPLAMEELQVALGALLTRFPGLRLAVPSSELRW

DTETIRRFPLELPVTW

>CYP107P17(2649524637)SCW

MAAFDPWDPAFVADPYPAYAELRARGRVHRYEPTGQWLVPHHADVSALLRDRRLGRTYLH

RFTHEEFGRTPPPPEHEPFHTLNDHGMLDLEPPDHTRIRRLVSKAFTPRTVERLVPYVRE

LAGELVSGLVAAGGGDLLTDVAEPLPVAVIAEMLGIPEADRAALRPWSAEICGMYELNPS

EETARRAVRASVEFSDYLRELIAARREKPGEDLISGLIAAHDEGDRLTEQEMVSTCVLLL

NAGHEATVNATVNGWWALLRHPAQLAALRADHSLVPTAVEELLRYDTPLQLFERWVLEDI

EIDGTVIPRGAELALLFGSANHDPAVFTDPGRLDLTRADNPHISFSAGIHYCIGAPLARI

ELAASMTALLEKAPALALAAEPERKPNFVIRGLTGLPVSL

>CYP107U1(2649525046)SCW

VTDQPHRPTPPPAQAGPAPALFTWEFAADPYPAYAWLREHAPVHRTTLPSGVEAWLVTRY

ADARQALADNRLSKNPAHHAEPAHAKGKTGIPGERKAELMTHLLNIDPPDHTRLRRLVSK

AFTPRRVAEFAPRVQELADRLIDRFAPAGEADLIHEFAFPLPIYAICDMLGVPREDQDDF

RDWAGMMIRHQGGPRGGVARSVKKMRGYLADLIHRKREALPPEPGPGEDLISGLIRASDH

GEHLTENEAAAMAFILLFAGFETTVNLIGNGTYALLTHPGQRARLQRSLAAGDRGLLETG

VEELLRYDGPVELATWRFATEPLTLGGQHIAPGDPVLVVLAAADRDPERFADPDTLDLAR

RDNQHLGYGHGIHYCLGAPLARLEGQTALATLLTRLPDLRLAADPADLRWRGGLIMRGLR

TLPVTFTPAP

>CYP157C23(2649526415)SCW

VTPESHAPTGTGDATLVPPPGCPAHALGPGGLHRLHAAEDLGELYEKLRAEHGPVAPALL

HDDVPVWVVLGHAENLHMVRAPAQFCRDSRIWTPLREGMVRPDHPLMPHIAWQPICSHAE

GDEHKRLRGAVAGAISTIDFRDLRRHVNRHTQRLVNRFCGQGRADLVGQFAEHLPMGVMC

EILGMSGEYNDRLVEAARDALKGTDTAIASHAYVMDALTRLTERRRAEPGEDFASHLITH

PAGLTDDEVREHLRLVLFAAYENTANLISNVLRVVLTDPRFRAQLSGGQMTVPEAVEQSL

WDEPPFSTIFAYFAKQDIELGGQRIRRGDGLLFGIAPGNVDPRVRPDRFADMQGNRSHLA

FGGGPHECPGQDIGRTIADTGVDALLMRLPDVQLDCDEDELTWRSSIASRHLVELPVRFE

PKPQQEVTELPSHRPVPAQRATWHVGMERPAAQTEAVPPPGTTAPSVPPGPPSGARPQPL

PQAQPQPELVPPPAPGDRPQSVWQRVLRWWRRS

>CYP105B39(2649527058)SCW

MPETLTHSSEGQAEWPMPRAATCPFDPPPALKSLQSEAPVSRVRIWDGSTPWLITRYEHT

RALLGDPRISSDATRPGFPRTAGSAQSDTRAPLSFINMDDPEHARLRRMVTAPFAIKRIE

ALRPAVQRIVDDSIDTMLAGPKPVDLVEALALPIPSLVICELLGVPYEDHDLFQHNTKVL

VKLSTAREESAAALTALTDYLERLLTSKLERPGNDLLSDIATRRVATGELTQREAARMGV

LLLIAGHETTANMIALGTCALLQNPDQLALLRDAPDAKTTASAVEELLRYLNITHNGRRR

VALEDIEIGGETIRAGDGLIIAGDIANRDPDVFPDPDRLDITRNARRHVAFGFGVHQCLG

QPLARLELQVVYSTLYRRIPALALAIEPDQVRYKHDGAVYGVYELPVTW

>CYP107AM11(2649527067)SCW

MTDQAERSYAPPSLQLPLSGDTALDPPAEWEELRARCPVAHATLPSGDTAVYLTRYDDVR

ALLSDPRFVRPTERDNAARVAPEGMGGAAVTGSAAVSIPDRGAPHQRWRRRVGRYFTAKR

MTALRPGMTRLAEDLIDAMLADGAPADLRASLGFPFPVYVICDLLGVPAEDRERFSHWSD

SFLSVTRYTADEIRTAQQEFVAYMSGHVAAKRAEPADDLLSTLIAESETEVGEGSEGSDG

GGLSHDELVATGMGLLVAGHETTANMIGKMVSMLLCDRSRWERLLADPSLVRSAVEESLR

FDTNLGFGLRRYIGEDVEIGGHVVPAGSTVVCSMPAANRDERAFDGADTMDLARTPNPHL

TFGVGPHSCLGQALARTELQVVLETLLTRVPTLRLAVPADELRRTEGLLVGGLREVPVRW

>CYP107CH3(2649527097)SCW

MSSREQCPYEDGRVVIDPAFKADAPARYARLRQSGPIHPAEFHLGLKGWVVVGHDLAREA

LTHPALLKDATPAAEALAAAGYVLHRPKVGLGAQMMEADPPEHTRLRRLASAAFTPRRTA

ELAPRIERIAHDLIDALPPSGEADLVEAFNAPLPATVIAELLGIPREHHLDFRRWSGQAL

QVASPEHRPALAGLHGLLGGLIADKRRHPQDDLLSALVAVRDEEDGRLSEEELVGTAMML

VVAGHESTVNLLGNAVLALLLHPEQLRLLRERPELMPGAVEEFLRYDTSVERSTSRYAAE

DLTLGGVPIPRGGMVVVALGSAGHDAPQTAGTDPALLDVARPNPRHLAFGHGIHYCLGAP

LARLETAIALRTLLSRVPELELAAPVDSLDWIGSGIIRGVLSLPVRYRVA

>CYP154A16(2649527111)SCW

VSEQPILVLDPTGSDHHAEHLALRAQGPLARVDILGVQAWAVTDPALLKRLLTSPDVSKD

PRAHWPAFAETVTRWPLALWVAAENMFTAYGGDHRRLRRMIAPAFSARRVAGMRKAIDRM

VATLLDNLAALPAGEPVDLREHLAYPLPIAVIGHLMGVPQEQSDALRGLVDNVFDTTLSP

AEAQANATLLYERLDQLIATKRRVPGDDMTSQLIAARDDETDGSGLTQDELRDTLLLMIS

AGYETTVNVIDRSIHTLLTRPDQLRLVRTGAIGWNNVVEETLRHEPAVKHLPLRYAVNDI

PLPDGRTIARGEAILASYAAANRHPDWHHDPDTFDAARPQQEHLAFGYGVHYCLGAPLAR

LEVATALEHLFDRFPGIRLAVPASELRPVPSLISNGHQTLPVHLHGPLADGPSTA

>CYP1031A3(2649527204)SCW

MTTETPSEPLIFNPFTPDFMSDPYPHYAELRRHVPVHEHPGGFWMLSRYEDVSALMRSGL

SVEQRHVAPGPFRDAYARAGVTDEPRLKGLALLDRDAPDHTRLRKLVTMAFTARAVNAME

GEIRSLVDEALDRIAADGGGDLVEALAFPLPFTVISRMLGMPPTDTVRMRTLTHTLMRSV

EPTTDPEVMRAVEAADAELFDIVGEAVDWKRQNPADDLLTALITAEDHGDVLSRDELIAQ

VTMLYVAGHETTVNLISGGTLALLRNPGQLRLLRDKPELEQNAIEELLRYDAPVHNSRRI

TLEPYEVGGHEIPPGSFILANLAGANRDESYFGPDAEELRLDRENARRHVSFGGGMHLCL

GAALARIEGRVAIGGLVRRFPGLELAGEVEWNGLLSLRGAARLPIRV

>CYP143C2(2649527205)SCW

MTSVDHSTASTVADLPVAERRDDAWRTLSDRGEVVPLADGLAVTSLAVVKAVLREPGRFS

IQKVFEAVETGYPLIPLAFDPPEQTRYRRILQPFFSPRRIRPLEESLRAQAVALVEAVKA

RGACDFVADIAGPFPAQALLTLLGLPLEDRDRFIGWKDAALRLTADAAGEVPLTDEERAA

EVAQTMAMGGYLAELIRTRREEPGDDVLSEILALRGEDRLSDDEALGVCLMLVLAGLETV

TDALGLGMERLATHPERQQELVRDLSLVPAAVEELLRLDPPAPFLPRMTTEDVEIAGCPV

PAGTLINAHLNTANRDEACWPRAHEIDFHRAENPHTSFGLGVHRCLGTHLARLEMRLVFE

EWHRRIPHYAITEGTSHRARLVRANLGLESLHLTF

>CYP171A3(2649527303)SCW

VSSPPSTIPEAPGAWPVLGHLPALLRDPLGFLSAVTERGDLFRIRLGHNTVYLATHPEIV

RTMLVSGAADFTRSKGAAGASRFIGPILVAVSGDSHRRQRRMMQPGFHRGKLDHYVISMS

AAAEETADSWRPGQVVDVPKMASDLSLAMITKALFQSDLGAAAEAELRTTGHDILKVARL

SALAPQLYTSLPTAAKRHMGRTSAAIREAVTAYRADGRDHGDLLSTMLRARDAEGNTMTD

DEVHNEIMGLAVAGIGGPAALTAWIFHELAHDHLIEQRLHAEIDTVLGGRLPTSADLPRL

PYTQRLVKEALRKYPGWVGSRRTVRPVRLGEHELPADVEIMYSSYALQRDPRWYRDPEKL

DPDRWESKETTRDVPKGAWVPFALGTYKCIGDNFALMETAVAVAVIASRWRLRPLKGDRV

RPVAKATHVFPDRLRMIAEPRTPAIPRGHAPADASLEAAARPKELPEP

>CYP155A-fragment2(2649527539)SCW

VSALELPTDDGPLAVRPGEVVELHLDHANTDPRAVGRDPLCVRPGRAMESGAGPAGLSFG

DGPHRCPGAHIALLETDVFLSRLFALDGIRMTAAPRVSFKDVIGGYEIRGLTVGLAPGAA

RSGGGPSPAR

>CYP155A-fragment1(2649527540)SCW

VYDREGTARKVDRGRERPGCPVTRAADGTWRVHDHAVARALLRGPGTVQAGLGVETVEKL

PRRIRRPVLYRDGPEHREHRRQTARFFTPRRVDEHYRDLMVRIAEEQLASLRATGEARLA

DLAFELAVGVVSEVIGLRYGRPGIAHWSLPARRTPRGRSAPASGASPWPPGTCSPTPVCS

TTTDRRTSPGVWPSSRRSCAWNPWSGGCAGAR

>CYP159A1(2649527565)SCW

MSTVQQVPDILSPEFAADPYPAYRVMRDSAPLIWHEATQSYIISRYEDVERVFKDKNGEF

TTDNYDWQIEPVHGKTILQLSGREHAVRRALVAPAFRGSDLQEKFLPVIERNSRELIDAF

RHTGSADLVADYATRFPVNVIADMLGLDKADHARFHRWYTSVIAFLGNLSGDPEVAAAGE

RTRAEFAAYMFPIIRERREKPGDDLLSTLCAAEVDGVRMSDEDIKSFCSLLLAAGGETTD

KAIAGIFANLLRHPDQLAAVREDRSLIARAFAETLRYTPPVHMIMRQSATEVTLSGGTIP

AGATVTCLIGAANRDENRYRDPDRFDIFRDDLTTTTAFSAAADHLAFALGRHFCVGALLA

KAEVEIGVGQLLDAMPDVRLADGFEPAEQGVFTRGPQAVPVRFTPVAP

>CYP157B15(2649527566)SCW

VTDTGSLAAGAAPAGCPAHTGAVRLAGLEYQQTPSQLYRALRREHGAVAPVLLDGDIPAW

LVLGYSELTYVTSHDELFARDSRRWNQWENIPTDWPLLPFVGYQPSVLFTEGEEHQRRAG

VITQALEGIDQFELARDCEQIADRLIASFAGSGEAELMSAYAHALPMRAVVQMCGMPHSG

TDTQQLVDDLRESLDAAEGDDPVAAYTRVGERIHQLVKEKRERPGPDITSRMLLHPAGLS

DDEIVQDLISVIAAAQQPTANWICNTLRLLLTDERFALNVSGGRLSVGEALNEVLWLDTP

TQNFIGRWAVRDTQLGGRLIRAGDCLVLGLAAANTDPQIWPDSHVGAENAAHLSFSNGEH

RCPYPAPLLADVMARTAVETLLERLPDLVLAVEPEQLTWRPSIWMRGLTSLPVRFTPVVQ

>CYP183J5(2649527599)SCW

VTLVAPPPIPRAAGSLPLLGHAVQLMRDNLGFIASLRRDYGPLVEITLQPGTRTVIVQDP

ELIRTMLVDLGPSLDKGRFFEKMGQLLGDSVVTAAGQEHVRKRRQLQPAFARGEIARYVD

IMRDEVSAALDGWRLGQSLDVREAMVKLSLDMLAKTVFAGSLDEATFRRLRRDLSVVMNG

VGARIMLPDWAEKLPLPFNRRFDRARDAVRATIQRAVDDLHASGHDTGDMLSLLLRATDE

ETGRPLTGDQICSEILTLAVAGTETTASVLSWTLYELSRHREVEARVLAELDEVLRGRPV

SFEDVTRLPYLRRVLDEVLRLHHTGWLVTRRTVTDTRLGPWTLPAGTELAYCQHALHRDP

ALFRDPEVFDPDRWLDSEAPPPSGAFLPFGAGKHKCIGDRFALTELITAIATIVRRVRFD

LRGQSVRPVARATVRPQTLLMTVRRREETAGRAGAPGVDSP

>CYP154B4(2649527611)SCW

MAWAVVRPGYVRRLLRDRRVSKDARQHWPAFVEGRITQEWPLFPWVAVENMLFTYGERHA

RLRRLVAGAFTVRRTQALRPGVERNVAGLLGSLAGVPAGQVVDLRAVFCERLPMQVICDL

FGVEEEPGRRLCEAMQTVFSTSVSAQEMTAAQARVFGMLAELVAAKQEVPGDDLTSALIA

VRDRGEGLSGQELLGTLNLMIAAGQETTSTLLTNAVAALLAHPEQLGHVRAGRAGWEDVI

AETMRTRAAAAYSPMRFAVEDIELDGVLIEKGDPILVSFAAAGLDPEQHGEDAAVFDVLR

ADRRDGLGFGHGVHFCLGAPLARMEAGVALAALFERFPGMALARPVEEIDPVPSFIINGY

SSLPVVLQPSAA

>CYP105BT2(2649527654)SCW

MANPAYPMARQCPMAPPPAYTTLRGQGPTKVDLPDGDWAWLLTSYDDVRQAMNDPRFSSD

DTKMSRARTQLPPNENLNSFWRMDEPEHGRLRHMMMTEFTAHRIKEWRPRIQALVDELLD

RLETLPRPVDLYSEFALALPTQVIAQLLGVPQKDYRQFAQQSRTILSLDRPEESWAAYYQ

MNDYLNRLMEEREREPADDLISRLIVDRVKTGELDREELLPMVRFILVSGYETTTSQIAL

SALTLMTNPEVRRQLIEEPERITAFVEESLRFWSVSQDNVLRVVDQDMEFSGATMKVGEL

VVLAVPAANHDERAFPDPERFDLDRGDNRHVAFGFGTHLCAGASLARREVEIAITSLLAR

FPEMRLAVGVDELTFRQKSLVYGLENLPVTW

>CYP107L35(2649527655)SCW

VPATTTVDLLRLSPDFVRDPYPVYAALRAQAPVHRVRTPDGPEIYLVLGHETCRAALTDP

RLSRDWRGSGRLRQIINADEDDPNLAHMSMAEPPDHTRLRRLVTREFTPRRIDALAPRVQ

EITDGLLDAMTADGARRADLVDSFAFPLPMTVICELLGVPELDRHSFRRWSNEMLAPTSP

QAQGAAYADLGAYLPRLIAAKRTEPGDDLLSALIHTVDEEGDRLSPAELVGMCNLLLIAG

HETMTNLVGNGMRALFAHPDQLRLLRGDLGLIDGAVEEMLRYDGPVETSLERLALTDVEL

GGVLIPAGSTVRMVLADADRDTARFARPEAFDIRRDTRGHLAFGHGLHHCLGAALARVEG

RIAIRSLLERCPHITADIGADALPWVPGLLVRGVRKLPVRW

>CYP105AC17(2649527846)SCW

MTSPFRGTTAQDAAGAVRPGRRAPDALTAPAVPAPAPEPVVLSTRRSCPFDPPPELARLR

DEAPVRPLRYPDGHVGWLVTGHEHVRKVLADPRFSARSEFKRVPVPRPGADPFIGRPALP

GWFVDMDRPEHTRFRRLLAGSFSAHRTRLLRPRLERFADDLLEAMAASGRPEADLVGAYA

LPLPSLAICEFLGVPYADRAAFQRDSTLLFSLEATAGEAETAMDALTGLLRALVRHRRRH

PGDDLLSRMVDGGLTDEEAAGAGVLLLTAGHETVAGMLGLGTFVLLCRPGDLAALRADLS

PRAVETAVEELLRYLTIFQFGVPRTPLEDVELAGRLLRAGESVTCCLPAANRDPLRFPDP

DRLDLTRPVGGHVAFGYGIHQCVGQNLARVELAVGYTALFRRFPGLRLAVPPGQVPLGTD

MGFYCVHRLPVTW

>CYP152D-fragment1(2649527873)SCW

VSFAAHALYLWPEYKEGLRSGETGFATAFAHEVGRFYPFAPFLGGQAVTDPTWQGRHVPA

GGLVLLEVYGQNHDERLWKDPYAFRPERFLDPPPGRDELIPGAEGTRAPDTAAPARPWRW

ASWRRSPSAWRPWPTGSHRRT

>CYP157K4(2649528034)SCW

MNDQTTPGAAGGCPVAHGGAPRLYGPEAATDPQGLYSRLRKQYGVVAPVLLEGDIPAWLV

LGYRENRRVLDNPLQFSRDSRIWRDWREGRVDESSPLIPMVGWRPDCVSQDGEPHQRLRA

AVTDNLNAVAGRGIRRHATHYAHKQIDAFAGTGRADLVTDFAEYLPMLVLTRVFGLAEAE

GRRLAESSNLVIKGGADAVAHNERIMGILGELTARKRAEPGSDFTTGLIEHHAGLDEDEI

VNHLRLVLITAHTMTSNLLARALQLVLTDTSWLSGLVSGQLDISTVVEEVMWNRPPLAVL

PGRFATADLELGGRPIKKGDLLVLGLAAGNSDPDIRPDADVSVQGNQSHLAFSAGPHECP

GQNIGQAIIETAVDVLLHRLPGLRLAVPPEELTSTASTWEDRLDSLPVEFTAA

>CYP102G7(2649528053)SCW

MPPTAPHPRPATGTPEVPVVDISATGPGRTPIQQVMGLMREHGPVLVRRLHGRDTLFVAD

LGLVTDLADDARFAKHIGPALENVREFAADGLFTAYNDEPNWAKAHDILMPAFALGSMRT

YHPVMLTVARRLVDHWDRAARAGQPVNVPDDMTRMTLDTIGLAGFGYDFGSFARDEPHPF

VASMVRCLEWSMTRLARTPGQDHTAADAAFRADAAYLARVVDDVIASRTGTDQRAARDLL

GLMLTAEHPADGTTLDAANIRNQVITFLIAGHETTSGAMSFALYYLAKHPAVLQLVQREV

DEVWGDEADPEPTYDEVGRLTYTRQVLNEALRLWPTAAAFSRHALEDTLLGGRVPLRAGQ

GVTVLAPMLHRQPVWGDNPELFDPSRFTAEAEAARPVHAFKPFGTGERACIGRQFALHEA

TMLLAMLVHRYRLHDHAGYRLTVKETLTLKPEGFTLTLTPRTPADRAHPALPGAATAEGA

GTTAGPAAAALPARVRPGTGALFLHGSNYGTCREFAAQLADEAAALGCATEVAPLDAYAQ

GLPADRPVIITAASYNGRPTDDATAFAAWLEGTADLSGVAYAVLGVGDRNWAATYQHVPA

RIDDRLAALGAGRLLDRAAADASGDLTGAVRDFTARLRTALLERYGDPDATAPAAGPAAA

YEVRTLTGGPLDALAERHGLVPMTVTETRDLTAPGHPRRKRFVRVALPDGVTYRTADHLT

VLPANDPALVERAAAAFGVDLGTVLDIRPTRPRRDGLAVDRPLTVRELFTHHVELQERPA

PDRLAALAAANPCPPERAALAALTDDPRTLVELVEDHPALRGALDWPALLELLTPLRPRH

YSISSSPAVSPGHADLMVSVLQAPARSGKGLYRGTGSGHLAAVRPGDTVLARIQPCREAF

RIDAADTRTPVVMVAAGTGLAPFRGVIADRVAALAAGASPAPALLFFGCDAPDADFLHAE

ELRAAEAAGAVGIRPAFSAAPERGISFVQHRIAAEAEEVWALLEAGARVHVCGDGARMAP

GVREAFRTLHRKHTPGADDIAAGEWLDGLVATGRYVEDVYAAG

>CYP102B20(2649528075)SCW

MAGTTKGPAGDGPRRGFRSAELGWPELHRIPRPPHRLPLLGDVLGAHGSTPVQDTLRYAR

QLGPIFRRRAFGKEFVFVWGAGLVAELADEARFAKHVGLGVANLRPVAGDGLFTAYNHEP

NWQLAHDVLAPGFSREAMAGYHRTMLDVAQRLTARWDRAQADGRAVDVPGDMTRLTLETI

ARTGFGHDFGSFERSRPHPFVTAMVGTLSHAQRLNTVPAPLAPWLLRRASRRNAADIACL

NRTVDDLIRARRTAPGQGDLLDRMLETAHPVTGERLSPENVRRQVITFLVAGHETTSGAL

SFALHHLSRQPEVAARARAEVDRVWGDTAEPAYEQVARLRYVRRVLDETLRLWPTAPAFA

REAREDTVLAGAHPMRRGAWALVLTPMLHRDPEVWGPDAERFDPGRFDAAAVRSRPPHTF

KPFGTGARACIGRQFALHEATLVLGLLLRRYELRPDPGYRLRVTERLTLMPEGLRLHLDR

RTAGATPPPLEPAARDGGSAPRCPVRGAGD

>CYP113K3(2649528186)SCW

VTSNQLTERRSPFDALADRWRSLREAGPVRHDERQGVWQVVDHHGVSTVLGDPATYSSDM

SPIAPAQEDFDAFRQGNFVGMDPPHHRKLRTLVSQAFTPRTVQGLAPRIEAVATRLLDGV

ADRDRFDLVDTLAYPLPIIVIAELLGVPAEDHPLFQEWASTLFGGEQLGEAPDMADLERA

LEAIAPTVREMNGYVLDHIRRRRADPGDDLTSRLLGAEVDGVRLTDQEIVGFVALLLVAG

HITTTALLGNAVVAFDREPGTFDALRADPGRIPDALEEVLRWLPPFPELGRRTTRRVTLG

GHEIPADTLVMAHLGAANRDPARFPDPDTFDVTRTPNPHLTFGHGIHFCFGAHLARLEAR

IGTRLLLERFRSLGVPSYDDVTHQNPAVIVGVRHLPVEVTRT

>CYP154Z3(2649528749)SCW

MAVAGPRLTDDTPAPQELALACPVYRLDPLGQDFPAEGRALQAIGPIVPVELPDQVAAWA

VTRRQVADTLLTHPDMRKNPQHWRAYQAGLVPETWPLLQIITTPTMLITDGADHTRLRLP

IQRAFTPRRVEALRPRVEEIVRGLLDTLAAVAPDTSVDLRSTFAFQLPVTVICELYGVDE

PDVRRQLATDTSLLLSSTTPPGERLGAQTSIFSTMAQLIAAKRVRPGDDLTTALIAEFDH

GGMSADELAGTLFLMLIAGHETTQNLLSNAIQRLVENPEQLARILSGHDEEAAWRGVVEE

ALRLDAPAATTMFLYAVRDITIEGVTIRAGEPVLIYTAAVGRDDHVFAKPDAFLPDRANA

HQHRAFGHGPHHCLGAPLARLEAGIALRALFERFSLTAAEPLGAVERITSLSSNAPARVP

VHLTPRRARRTWAQPFGG

>CYP1416A3(2649528750)SCW

MTNSPHVRPSQGAAPLPASGCPVRLEPVRLNSEAVQGSLADLYEPWRKEYGPVVPVELDG

GVPAFLVIGHRTLREVCSQESLYSPDSRNWADWRAGRVPNDWPLLPQVAYQEGSTRFLSG

PEHQRLRGVLASGLAQVEAAPARRYTEWVADRLISRFAPKGQAEVVADYAAPLPLLVMLR

LLGLPHETGEQLLPAIFRLLEGGPGAHRANEEISDIIGRLVVARRAKPDRDLISWLIHGP

VDGGPALSDLEVRNLAWLTVMAGAGGTTGWIGNAMERLVCDETVHTLFLAGKVTIAEIMN

ETHWSNPAVQNVMGRYPLREGQLGNYHVPAGALLVLGLAAANADPEVHVDDRGHTFTNES

HLAFGSGPHECPTPAQRLAKVIGQTAVERFLARCRTPRLRDDGAVQHGGSVIVRQLTKLA

VTFTADTQAALRRTAPGTDVLGESSRSSHYLFPPRMLTQLTSHITD

>CYP183A4(2649528849)SCW

MSEQTTFVAGAAPGAVPVVGHALQMMRHPVNFMTSLSAHGDLVQIKIGPTSAYVPTHPDL

LRYVLTNDRIFDKGGVFYDRARDIAGNGLVTCPFADHRRQRRLMQSAFTRTQLKRYATAM

HAEIEATTARWHDGMVVDAFPELYGMALRTVGRTLYSTPVSPELAAGVERAFDVVLNGLF

RQMFLPRFIRRLPLPANRRYESNLRFLHRTTQELIDDYRSDGAAHDDLLAALLASRDDDG

GRLDDKEIHDQVITVMAAGTETVAGTLTWVFYLLSQHPEIEAALYEEIDTVLGGRAPNWD

DVPNLSLADRIISEALRLHPPAWLFTRLTATETELAGRRLPEGTTIVFSPAAVAQYEDAF

GNPTAFDPDRWLPDRVSPAARQAFMPFGTGARKCIGDLYARTEATLGLATILGRWRVTCE

PDMDVRPVPLATVYHPRRLRLRLSARAPRPAATPVPAPTGGEPT

>CYP161C4(2649528857)SCW

MTELPRLPFDNPDIIGIAPQMLALQKEGPIARVRTAGEDAWLVTRYDEVRTLLADRRLAL

SNPYPERTTKSAARAFMVALMAGDDHHTEAPRHAQMRALLVPRFSTRRMRLMKARIEQHV

DELLDELAASTPPVDLHRALSFPLPTMVVCDLLGVPLADRERFGQWARGTFDQSDNQHSA

NTFQQVVDYMTELVARKRTEPGDDILSELIADKDHTLSDAEIAHLGNAVLLFGYETTIVR

IDLGTLLLLRNPAQRALLAEKPELAPAAVEEILRLGVGGNGSNAIIPRYAHSDITVGDTV

IRAGDAVMLAIGAANYDERAYPGADLFDLTREKPKSHMAFGHGARHCIGRTLARIELTAV

FERLFRRLPDLRLAVPEESLRWQEHRITGGFDEIPVTF

>CYP105AC8(2649528967)SCW

MGEPVHAVPMLPTARRPGCPFDPPEELTDARRHGPISRLTHFGGNPGWLVTGYDLVRSVL

ADPRFSSRRELMNVVDYELPPAPPGEFLLMDEPRHGRYRKPLVGKFTVRRMRLLTERVEQ

ITTACLDAMEKAGPPADLVTSFAKPIPAIVICELLGVPYEDRGSFQEQIDAFVGGGTDED

ELMAAYTATQDYLAGLVAAKRAHPTDDVLSELTDSDLTEEELKGIALVLLVAGLDTTANM

LSLGTFALLENPAQLAALRADPALADRAVEELLRYLSVAKSFMRTALEDVELGGQTIEAG

TTVVLSYHTANRDPERFADPDTLDIRRQHTGHLAFGHGIHQCLGQQLARVELRVALPALI

ARFPALRLAVPAEEVGLRPETADIYGVTSLPVTWDTEAS

>CYP105B36(2649529205)SCW

MPTPAPFPPLPVEPPSGCPFDPPEGLARLRLEEPLSKVALDDGSWAWLATRYADVRAILG

DQRFSSDTSTPGYPVSGMTGGSPRPDAARGFIRMDPPEHTRLRRMVTRDFMVKRVEALRP

TLQRLTDELCDEMERVDRSEHPVDLVQALALPLPSLAISLLLGVPYEDHDTFQRLTGTLL

SRETGEEDRGPARAELLAYIDGLVRAKVAEPGDDIISRLATEQHARGELTHEDLVAFAVL

LLVAGHETTANMIGLSALSLMLDPETAGRLREDPSLVRGAVEELLRFHSIIRNGPRRAAL

EDVEVGGRLIRKGEGVIVAVPSANRDEDVFPDAGRLDITRPNAQHHVAFGYGIHQCLGQA

LARAELQIVITTLLRRFPTMRPAVPVEEIPFRTDMVIYGCHALPVTW

>CYP107W3(2649529855)SCW

MSDATTEPIAFPFPDPPSVCDLPPELAEVRDGRSVAEVRFPDGITGYLVTKHADVRKVLV

DSRFSSKVMASAAAAMSETETGKLMNESLVGMDAPEHTRLRKLVSRGFTARRVESLRPRV

RELVAELLDEMETKPRPVDLVKNFSLPLPVRVVCELLGVPAEDQDTFHAWSNALLGDWQQ

VVEKEAATVALVKYFGELLEAKRKAPADDLMSALIAAREDDDTLTEREIIALCIGILSAG

HETTANQISMFLVQLLRHPEQFAALKADPAALPQAVDELLRYVPLTTTGGIIPRLTTAEV

ELGNGEVLPAGAVVLPAVATANRDPEVFEDGERLDLTRAVNPHLAFGAGIHYCLGAQLAR

IELQEALGALLERMPQVRLAVPESELRLKPASIIRGLESLPITW

>CYP107F10(2649529924)SCW

VEAAEAVWSCPFDFAEALEFDPLLRKLLEEHPVARITMPYGEGEAWLVTRYDDVRTVTTD

RRFSRKAVTGRDFPRMTPEPIVQSEAINLMDPPAVTRLRGLVAKGFTAGQVERMRGRTER

VVDELLTAMAAHDAPDLFAHLASPLPMHTICEVLDIPEPDRKRLRHNALTMMNIGAAGKE

AAVRAKAELRAYFTELTAERRRAPGQDLISSLATARVGDDLLDDRELAVMAMVLLITGQD

TTTYEIANLAYLLLTRDDLLSALRDRPELLPRALDEMLRHIPFRKGVGIPRIATEDVELG

GVTIRAGDVVHVSYLTANRDAEKFPNPHDIDLTRPAVPHMTFGWGSHHCLGAPLAEMEMR

VALQALLTRFPGLRLAVPPEDIRWNTTSIWRHPLALPVTW

>CYP170A17(2649530007)SCW

MTVGSSPEFPAPLGQEAPPPSDTRPQAELLEPPVAGGALPFLGHGWKLARDPLAFLSQLR

DHGDVVRLKLGPKTVYAVTTPALTGAVALSPHYIIAGPLWESLEGLLGKEGVATANGPLH

RRQRRTIQPAFRLDAIPAYGPIMAEEAQALVERWRSGEVLDITAESFRVAVRVSARCLMR

GSYMDDRADRICGALATLFSGMYQRMVLPLGPLYRIPVPANLEFNRALADLHLLVDEIVA

DRRASGQKPDDLLTALLEAKDDNGEPIGEQEIHDQVIAIITPGSETVGSMIMSLLLVLTE

HPELGDKIRDEVKSVVGDRPIAFEDARKLTFTANVIVETMRLYPAVWILTRRAVTDTELG

GYRIPAGADLVYSPYAIQRDARSYERHEEFDPDRWLPERSKDVPKYAMTPFSVGNRKCPS

DHFSMAELTLITAAIAAAFRFEQAPGSDPRPRIGITLRPRRLLVRALPR

>CYP107CH2(2562404297)SDV

MSPHEQCLYEDGRVNLGPAFKADAPGQYARLRDRGPIHPAHFHLGLRGWVVVGYDLAREA

LTHPGLLKDSTPAAEALTAAGYVLNKPSVGLGAQMLEADPPEHGRLRRLASAAFTARRTA

ELAPRVEQIAHDLIDALPAAGETDLVEAFNAPLPTTVIAELFGIPEEHHADFRRWSSYAL

QVASPEHRPALAGLHALLADLIAAKRRDPRDDLLSALVGVRDEQDGRLSEEELVGTAMML

VVAGHESTVNLLGNATLALLRHPDQLRRLRERPELLPGAVEEFLRYDTSVERSTSRYAAY

DLELGGVRIPRGGIVVVALGSAGHDAPQAEDTDPSVLDVSRPGARHLAFGHGIHHCLGAP

LARLETAIGLRTLLSRVPELELAVPVDSLEWIGSGIIRGVRSLPVRYRLA

>CYP105BA1(2562404761)SDV

MTEKTCPITNGLPTLRERPFDPPQVLRAEGPICRMAFPDGHEGWLVTSYQYGREILSDKR

FSSAATHKHLAFPSDRAQDMGEGIPGLFEHMDPPEHTKFRKLLAGQFTLRRMRALTPRIE

EITAQYAEAMLRKGAPADLVADYSVPVSSQVICELLGVPFEDRERFEGNSAKLLRLDLST

EETQAALMDLVGLTGELLMRKQAEPADDVLSVLVNGEDMSLPEAIGATLLLLVAGHETTA

NMLSLGTYALLNNPDQMALLRADESLIEGAVEELLRYLTVVHVGVQRSPTEDVEIGGVTL

HKGDTVLIHLPTANRDPEQFTDPDRLDVTQGTLSHLTFSHGIHQCLGQQLARLELRIGYT

ELLRRFPELRLAATPDEIPMRSNMTVYGVHELPVAW

>CYP156H1(2562404877)SDV

MTTYPTTQQTFSRSAPVRLWEDGFALDPYRYYADLRAQGPVGWAELAPGVPAYVVTDRRA

ALDLLHDTETFSHDPRPWEATVADDSPVLGMMRWRPNTLFADGTAHVRYRTALLDAFDRV

EPHDLRSRVHRAVRLLVGRIGPRGKADLVVDFARPLMALVFNDLFGLPDSASDRLNSALG

KMMEGGAEAAEGEAEFGGYVLELIAAKAERRGEDLPSWLLDHPAGLTPEEVTWQVFLTLG

AGHEPTANLVSNALSRILGNPSYYSTLTSGARPVMDAVVEVLHHETPLANYGIHYARTPV

TFHGAWIRAAVPVVISYGALAHAAEQDHGARRHPGDASHLSWSAGPHACPVKQHTLLIAT

EAIERLTQWLPDLDPVLPRERLTWRPGPFHRSLTGLPVRFSPRSPDLPGGPS

>CYP154U1(2562404878)SDV

MTVTDRTERFALDPFGSDIAAESARLRALGPVVPVDLPGGIPAWAPTGYDTLKQLILDAQ

VSKDPRKHWRLWPEIGEHPSWGWILGWVGVVNMLSTYGPDHTRLRKLVAPSFTQRRTEAM

RSRVETITAELLEKVAGSGDGTVDIKAALAHPLPLRIICELFGVPDELVPDTTRLIAAIM

DTSDPSPEHAASVQQQIGTVLPALIAHRTEHPGDDLTTELIRVRDEDGDRLSDEELLYTL

LLVIGAGFETTVNLIGNAVVALLRDPGQLAAVRSGEIGWDAVVDETLRVHPSIASLPLRF

AVTDIEIAGVTIPAGDAIITTYAAAGLDPEHYGPNADSFDAARGADDHMAFGIGVHRCIG

APLARVEAMTALPALFDRFPGMRLAVDADELRQVPSFIAFGWQEIPVLLGD

>CYP1005B2(2562404935)SDV

MLASPTVTSPALGATTPVSTAVGLFDDAFTRDPYPWLESLRSDAPVHFDADTGLWLVSRH

RDIRHVLLTPGDFLPDNAQNAVTPLPFGALRVLARAGFNLPIALANNGTDSHPGLRRVVT

RFFNAQRVTAAVPVIERIADELLDGVAERLDASGRCDLFTSFAQVLPCRVLMELLGIDGV

PTATLIRWSDASLELFWGRPAPERQLELAVLVAEFHQWLTETVAGGSARPGSFVRALTDH

RLPDGERLDVETAVAACFFVFIAGQSTTGQLIATVLRHALTEPGMWARVASPDDSARAWV

EEVLRREPPVTSWRRVTARPVELGGVELPAGAQLLLLLLGSGSDPEVFDAPDRMCPHRSN

VRQHLAFGVGRHRCPGASLARTEAAVALRTAARRLPSARFVPAADDPMLGLLSFRAPLRV

TVESP

>CYP179B1(2562405101)SDV

MTRSIGESYDPLGTHLQDPYPFFALARETEPVFYSEVLRAWVVTRFDDVRTVLQQHETFS

TANALRSPLPLHPDALAVLERGFPPSSAIINVNGDRHLKVRAPLAKRLGTDAVAAMEPEI

RKRATALVDALAADGSAELMSQYARVLPVDTISDMCGIAAEDRAIIRDGAYACVAVITGG

ASREEEVEAAELFLEFQQVMARYVRECRTRPGPDAFSEIAGVLAPAGPLDDEQLAELVWT

FIGLITAGHSTTTALLGNGLWHLLSRPDQWQLLCRRPELIPGAVEEIARYDTPVHAFFRV

TTREVTLGGRTLEAGADVVALYSAANRDPEEFDQPETFDITRQITRHLTFGHGVHACVGA

RLARLQLAVTLEALTTRLPGLRLVPDSPVRMARQFVDHAPVALHVAW

>CYP157K2(2562405163)SDV

MTDQKPDRPDAPRGCPVAHGSAELARLYGPEAATDPRGIYERLRKEHGSVAPVLLEGDIP

AWLVLGYRDSRRVLDNPRQFARDGRIWRDWREGRVAPNSPLVPMLAWRPDCVSQDGEAHR

RLRGAVSDGLQSAADRGIRRHATHFANKQIDAFADSGRADLVADYADLLPMLVTARILGL

AEAEGRRLVESCAQVMKGGEDAVRHDGVIREIFGELAERRRTEPASDLATGLLEHHAALD

HDEVVAHLRAVLIAAHVTTNLLARVLELVLTDANRLSGLISGQLNISAVVEEAMWNSPPL

AVLPGRFAASDVELGGHRIEEGDLLVLGLAPGNVDPEVRPDPDVSVQGNQSHLAFGAGPH

ECPGQSIGMSIIEVAVDVLLHRLPGLRLAVPPEELNSTASTWMSLLDTLPVEFEV

>CYP102B5(2562405195)SDV

MAETTNGSALSSEPPSALPKGFRSAEQGWPELHRIPHPPRRLPLLGDVLGASRTKPLQDS

VRHARRLGPIFRRKAFNKEFVFVWGGALSADLADETRFAKHVGLGIANLRPVVGDALFTA

YNHEPNWQLAHDVLAPGFSREAMEGYHGMMLDVAGRLTDHWDRAAMAGRTVDVPGDMTKL

TLETIARTGFGHDFGSFERSRPHPFVTAMVGTLAHAQRLNTVPAAFLLRASARRNEADIA

YLNRTVDDLVRERRRGSGGTGDLLDRMLQTAHPETGERLSPENVRRQVITFLVAGHETTS

GALSFALYYLARHPDVAARARAEVDRVWGDAELPAYDQVAKLRYVRRVLDESLRLWPTAP

AFAREAREDTVLAGEHPMRRGAWALVLTPMLHRDPEVWGADAERFDPDRFDPKAVRSRPP

HTFKPFGTGARACIGRQFALHEATLILGLLLRRYALRPDPDYRLRVTERLTLMPDGLRLG

VDRRTEAISDDLRAAAAPS

>CYP154A13(2562405385)SDV

MGHPRPLVIDPTGRDIHGEASRIRERGPVTLVELPDGVQAWAVSSPELLKRLLTDPRVSK

DPRRHWPKWINGEISPEWPLFTWVAVQNMFTAYGGEHKRLRALVSKAFTARRTAALRPRV

EEITAGLLDRIEEAGRHGQVVDLREEFCYPLPIAVISELFGLPEEQGAELRAVVDGVFHT

SATPEEVTDNYARLYAALGELVTLKRKSPGDDLTSALIAARDDGDARLSEQELLDTLVLM

VSAGHETTVNLIDNAIHLLLTHPDQLTHVRSGRVTWEDVVEEALRVEAPVASLPLRYAVE

DLKLSEFGGPDGAVIGKGEAILAAYAAAGRDPGKYGADADRFDVTRVDKEHLAFGYGVHF

CLGAPLGRMEAHIALRALFDRFPGLRLTQSGDELEPVDSFISNGHRTLPVHLS

>CYP156C10(2562405386)SDV

MTSATPAPPPFPPPGCPAHESLYGPEFAADPATVYGRLRDVGPIAPVELAPGVRASLVVG

YEAALHVLRGTETFSKDARRWKDLADGTVPADSPVVPMMMYRPNALWTDGTEHRRLRGAI

TDSLARIDSATLRGYVEASADTLIDRIAPAGQADLLGEYAQVLPLLVFNRMFGCPADYGE

RLVRGMSGIFDGVDAEQANNLLATTLLDLVTLKRRQPGPDVTSWLMAHSAELTDEEMVHT

LVLLMGAGTEPQQNLIANSLRLLLSDDRFAGSLSGGSLPVEEALDEVLWTDPPMANYAVH

YAIHDVVYEGALLRAGQPLVVSLAAANTDPALTVDQRAGNRAHLAWSAGPHNCPAQGPAR

LIAAVAVEKLLDRLPDVELSVPVEDLAWRPGPFHRALAALPVTFPPTTVTPPTPGGDSPL

EPVAPTPRPSAVPPVGRVRRGWGARLLAWWRGE

>CYP183K1(2562405523)SDV

MSFTTGTAPGAIPGLGHIWPLMRSPVDFLTSLPDYGDLVEIRLGVTPGYVPCHPELLRQT

LIDDRTFDKGGKYYDRARAMAGNGVATCAHKDHRRQRRMMQPAFHHQQLERYGPVVEEEI

AALTEHWGDEQVIDAYSVLYGLSLRTVTRTLFAAKVDEEVVEGIRHSFDIAFSGFFRQMF

LPRAVLELPLPANRRHRRALGHLRDTVHRVVADSRATEGDEGNVLAALMASEPDDQEIHD

QVVTVLAAGSETVASTLTWALYLLSEHPEAARALQNEIDTVLAGRPARWADIPQLPGLHR

VVNETVRLYPAGWLFTRVTTRDVELAGTLLKEGSTVVITPVPVHRNTELFEDAAAFAPER

WLPERISGLPRGAFAGFGTGPRKCVGDDYGVGECVLALAAILGGWDVQCEPGADTRPVPL

AAFYRPRKLTMRLIRRVGHHG

>CYP158A6(2562405589)SDV

MNDETTTLSEQAPPAIRDWPATDLAGTEFDPVLADLMREGPLTRIRLPHGEGWAWLATRY

DDVKAITNDPRFGRAEVTRRQVTRMAPHFKPRPGSLAFADQPDHNRLRRPVAGAFTVSAM

KRLRPRAQRILDELMEGVVRDGPPADLIERVLEPFPLTVVSEVMGVPAADRAQVHTWTRT

IISTTGAEDAERAKNGLYGWITETIRARAHSQGDDVYSLLGAAVHREEISETEAVGLAGP

LQIGGEAVTANCGQMLYLLLTRPELMARMRERPEERGAVLDELLRFIPHRSSVGLARIAL

EDVELHGMRIRAGEPVYVSYLAANRDPEVYPDPDRIDPDRSAPPHLAFGNGPHYCTGAVL

ARLQTELLLGTLLERLPGLRLAVAPEDVPWRRRTMIRGPQSLPCAW

>CYP102D2(2562405832)SDV

MATQPVTDLRPIPSPRGIPLLGNTPQIPDTNPVEYFAELSMQFPDGIYGLDIAGIEQIFV

YDPDLVAEVSDETRFFKQIEKTPLQHVRDFAGAGLFTAHQHEEEWGMAHRILLPAFSQRA

MRAYYGQMLEIAQNLVGKWERKAGQPVNITDDYTRLTLDTIALSGFGYRFDSFDKEELHP

FLNALLGALIESLRRSQELPMMTKLRKADAKKYGENVQLMQELVESVIKERREGKGTGEE

DLLGLMLEATDPETGNRLYDDNVRDQVLTFLIAGHETTSGLLAFATYSLMRNPHALAQAY

AEVDRLLPGDTVPDYDTIMQLDVIPRILDETLRLWAPIPSFGKTAREDTVIGGRYELKKG

AKVLILEGPLHTHPKAWDRPDEFDIDRWLPENRVQHHPHGYKPFGNGVRACIGRQFALTE

ARLALALVLQKFKFSDTNDYKMDPREALTRKPGNFELIVRRRQEHERTVFGAADVQTGDT

QAPAAVSGVGVNLTVAYGSSLGSCEDLARTIADRGERSSFGTTLVSLDELGDNLPSEGLL

AVVAASYNGKAPDNAQRFDDLIAAGLPEGSLSNVRFALLGAGNTQWVATYQAFPQRIEAA

LLAAGATPVVERGIADAAGDFDGMASRWMDGLWATLAEEYAADTSDDGGPRYEVQLLTEA

DVRPAIVSEQAYPLTVVANEELVSDATGLWDFSIEPPRPSAKSLTIELPEGVTYDTGNHL

AVFAKNEPALVDRALARLGVDRDQVLRLQQPAGGRTHLPVGTPVTAGLLLTEFLELQDLA

TRSQIQTLAEHTECPWTRPQLEAYTADTEEAGQRYQEEILEKRVSVLTLLERFPAVELPL

AVFLEMMGPIRPRFYSISSSPLASPRQVRLTVGLLEGPALSGDGQYRGTCSSYMARLEPG

DVVYGYVRVPSPTFAPPADPTTPLILIGPGTGIAPLRGFLEERARQYENGTEVGLSQVFV

GCRHPQHDYFYRDEMEMWELSGVARVHTAFSAVTGHPARFVQNAIAGAAGTVWQALQDGA

YVYVCGDGRRMAPAVREALAAVHREHTGGDDEAAQRWLAQLEADERYQQDVFA

>CYP147F7(2562406053)SDV

MTQSLLHRILDHANRADPYPIYEELRRTPVHHEPDGPYVISTYYEIRSLLHDPRISSDAR

NLASHTADPLAEPGSAEGGALPPGFLKLDPPEHDRLRRMTNRPFGPPHSPHRVDGMRPEL

HDIVTGLIDGIVDPGRFDLVEQFSYPFPVTVICRLLGVPREDEPRFHVWADTLAASLDPD

PDADPAERGKGAHDARMELGMYLAGLIEERRKHPGEDMLSQLATAKGGDGAMSTMELLST

AALLLIAGHETTVNLITNGMLTLLRNPDVLRRLREDPRLAVPIVEELLRFEPPVQLVPQR

TTLADIEVRGVTIPKGAPIWLVLASGNRDPRRFEDPDRFDPDRPDIQHLGLGSGIHSCFG

APLARLETQFALAELARRMENPRLVQDPPPYRQNAVLRGPRQLHITCDGIRA

>CYP107L20(2562406134)SDV

MSGVIDLGEFGEEFRSDPHPAYARLRALGPVHRVRPAGWESETWLVVGHEEARAALADPR

LAKDAAKIGLSSLDEELIGKNLLVCDPPQHTRLRGLISRAFTMRRVEELRPRVQRITDDL

LDAMQPYGRAELVESFAHPLPLTVICELLGVPEMDRAEFRKMSTEAVAPTTSGSEYDAFV

GLAAYFTELIEDKRAAGPSGDLLSDLIRTTAEDGDRLSADELRGMAFILLIAGHETTVNL

ITSGVHALLTHPDQLAALRADMTLVDGAVEEMLRYEGPVENATFRFAAEPLEIGGVEVAA

GDPVMVCLTAADRDEARYPDPGRFDIRRDTRGHVAFGHGIHYCLGAPLARLEARTAIRAL

LDRAPDLALDGPPGEWLPGMLMRGMRSLPVRW

>CYP179A3(2562406233)SDV

MDRQPHGRLDELLRDPYPHYERARHTGGLTHVAELDAWMVARDTDVREVLRRPEDFSSAN

ALRPDVLPSPAALAVLGRGFGGRPVVVTADGALHQRLRTPIVRGLSPARVTAVLPYAAER

AAALVDSFAADGSVEFMSAYAHRLPGEVIGRIVGIDPADVPAVVHGGHRAEELLFRPLKE

AEQVAAAEDVVAMQQLLDRYAQRRHAEPREDLCSELVASVLPPGAAELTLEERHELVAHL

QNLLLAGHLTTTALLGTTVLHLLRHPDQWALLCAEPERIPAAVEEAARYDSALQGFRRVT

TRPVTLAGTELPAGAALFVAFGGANRDPERHPRPDAFDITRAPGRHLAFGLGAHGCPGSK

LAREQLRITLEELTRRLPGLRLAEDDPVVMRPTMIHRSPYRLRLTW

>CYP180A6(2562406364)SDV

MTVRPSPPVPDVFDPRQYAAGVPYAAYRVLRDHHPVAWQDEPEVLGWPAGPGFWAVTRHA

DVVRVLKDPLTYSSHLGATQIRDPDPQDLPFIRRMMLNQDPPGHGRLRRLVSRAFTPGRV

DRFTALAEKRAQSLFAGALETAREQDGTVDLVTAVTDDYALLNLADLLGVPERDRGLLLH

WTQRVIGYQDPDESGPPVLDGAGKPVNPRSPAMLRDMFDYAGELAVYKRRYPADDVLTIL

AHDAELTGPELEMFFFLLTVAGNDTVRSAAPGGLLALAEHPESYERLRAGDVELAPAVDE

LLRWHPPVLTFRRTADRDTELAGRRIRAGDKVVVFHASANRDERVFADPDRLDLTRTPNP

HVSFGDGPHVCLGAHFARLQLRVLYREALRTLPVLRSAGPPGRLVSNFINGIKSLPVEVT

>CYP107CN1(2562406433)SDV

MQTQPELEPLLDFSPYRLLTEPDAVHLRMRAEPPVRLVREESGFTYWLISRYAEARQALR

DPGLSNDPRRLGHAVDTANAYSPMANNDPPHHTRLRGLVSHGFTRRRINALAPRAESVTG

ELLDAIAPTGRADIIADLAFPLPVLIICELLGVPTRDRETFRTWAARTLSTAAGPRTREE

RSRRLRAYFTRLVAAKRAAVRPDLAPDEQPDLLSALIVAQERDSSLDDDELIGLAVLLLV

AGHETTTNLIGNGLLTLLLHPDQLVMVREDPTLLASAVEELLRYEGSAGQSSLRVAVDDV

VIGGTVIPRGSVVNIGLSLANRDPEAFRDSDTLDVRRDPNPHLAFGHGIHYCLGAPLARM

LAATALGALLGRCDGLQLAIPPDELRWRQISILCGLTALPVSFDPVRPETSGT

>CYP107CP1(2562406445)SDV

MSPPTEIDTPSFLADQHGYYAGLRETPGPRLVRNPQGLGYWLITRHADARAVLLDPRFSK

DPRLAEQALSAAGYGVFGADSFFLPLVNSDPPDHTRLRRLVSGAFTPRRVEELRPRVERL

THELLDAVPDEDGDEEGVDLMAVLAFPLPVLVISELLGVPHRSRGALLTWATRMLTVSGN

GSGPAERTRRLHRWFASLVAAKRPHVRRDLDQDEQPDLLAALVVAHDQGQRLDDEELVGL

LVLLLVAGHETTTGMIGNAVDALLRHPDQLALLRDRPELLPSAVDELLRYEASLARTTLR

VAREDVKVADTVIPAGSVVSVALSAANRDPEVFPEPDRLDITRARGPHLSFGHGIHFCLG

APLARLQTETVLAVLLHRFPELAAADPEAPQLWRPVGDMRGLLTLPVRLRPAF

>CYP170A9(2562407251)SDV

MTVESVKPETQIYAESELREPPVAGGGVPLLGHGWKLVRDPLSFMAALREHGDVVRLKLG

PKTVYAVTAPDLTGALALSTDFKIDGPLWESLEALLGKEGVATANGPRHRRQRRTIQPAF

RLDAIPAYGPIMEEEAHALTVRWRPGETVDCTSESFRVAVRIAARCLLRGDFMDERAERL

CVALATVFRGMYRRMVIPAGPLYRLPLPPNRKFNRALADLHALVDEIVAERRASGQRPDD

LLTALLEAKDENGEPIGEQEIHDQVVAILTPGSETVASTIMWLLQVLTEHPEHADRVAAE

VESVTGGRPVAFEDVRKLRHTNNVVVEAMRLRPAVWILTRRAVTDTELGGYRIPAGADIV

YSPYAIQRDPKSYADNLRFDPDRWLPERVKEVPKYAMNPFSVGNRKCPSDHFSMAQLTLI

TAALATKYRFEQVSGSNDATRVGITLRPHDLRLRPVSR

>CYP107P2(2562408312)SDV

MGAFDPWDPAFLADPYPAYAELRAKGRVHYFEPTNQWLVPHHADVSALLRDRRLGRTYQH

RFTHEEFGRTAPPAEHEPFHTLNDHGMLDLEPPDHTRIRRLVSKAFTPRTVEQLKPYVAR

LAGELVDRLVDAGGGDLLTDVAEPLPVAVIAEMLGIPEADRAPLRPWSADICGMYELNPP

EETARKAVRASVEFSEYLRELIAERRKEPGEDLISGLIAAHDEGDRLTEQEMISTCVLLL

NAGHEATVNSTVNGWYALFRNPDQLAALRADHTLVPAAIEELMRYDTPLQLFERWVLDEI

EIDGTTIPRGAEIAMLFGSANHDPEVFANPDRLDLTRQENPHISFSAGIHYCIGAPLARI

ELAASMTALLERAPALSLAADPDRKPNFVIRGLEGLTVAV

>CYP107U1(2562409314)SDV

MTDHPTPAPHATAPDLFTWEFAANPYPAYAWLREHAPVHRTTLPSGVEAWLVTRYADAKQ

ALADQRLSKNPEHHAEPAHAKGKTGIPGERKAELMTHLLNIDPPDHTRLRRLVSKAFTPR

RVAEFAPRVQELTDRLIDRFAAEGSADLIHEFAFPLPIYAICDLLGVPREDQDDFRDWAG

MMIRHQGGPRGGVARSVKKMRGYLADLIHRKREALPAEAAPGEDLISGLIRASDHGEHLT

ENEAAAMAFILLFAGFETTVNLIGNGTYALLTHPEQRTRLQDALAAHDTALLETGVEELL

RYDGPVELATWRFATRPLTIGGQDIAPGDPVLVVLAAADRDPERFADPDRLDLARRDNQH

LGYGHGIHYCLGAPLARLEGQTALATLLTRLPDLRLAAEPADLRWRGGLIMRGLRTLPVE

FTPSR

>CYP113J1(2562410014)SDV

MTSATPEAAPFEGPPAVVGDGRELYAWLKRMRREAPVWIDPASGSCHVLRHADALRVLSE

PAVFSSDFSSLAPPPEPGLPNFAEASLSVTDPPRHGQLRKLISQAFTPRTVAGLEPRIQA

VTTELLDSIAERTDFDLVEELSGPLPVVVIAELLGIPSSDREMFRRWAEYLLPPSDEVTA

GEFLEGGYAKTRAAELTEMADYLLSHVQDRRTTPRDDLLSRLAAAEADGERLTDKQMVNF

SAFLLLAGHLTTTLFMSNALLSFAETPGTLTEIRKDPSLIPGALDEVLRYRPPVSFLYRL

TREDTRIGEVDVPAGRIVVTWLLSANRDEVQFPDPDRFDPHRAPTGHLTFSHGIHFCLGA

PLARMESSIVLNALLTRYRTITLGTPTYHQRPDIYGVTSLPVSVEPR

>CYP113J2(2562410015)SDV

MEPTEATATTEIPRPPTGVGDGSQLLDWLRTMRDRHPVWRDDMTGAAHVFRYDDLLKVLS

SPDVFSSDFSAIMPPPDPDAPNFTEGVLTMTDPPRHGKLRRLVSQAFTPRMVAQLEPKVE

VLTRELFDTVAGERDFDLVSAVAYPLPVIVIAEMLGIPPADRELFRGWGEALLSTNYELP

VGAVPDGTMPEQIAAQLKEMHAYLLGHVQDRRTTPRDDLISRLVTAEVDGDALTDGETVS

FLNFLLLAGHLTTSLLMGNTLLSFADEPAQLKAVREDRELVPAALEEVLRHRPPVVFQAR

VTRQETELGGVTLPANVPVMCWQMSGNRDERHFPDPDRFDISRTPNTHLTFGHGIHFCIG

TPLARLESRIVLNELLDRYRQVDVGTPVYYERSPEIFGVKSLPITVETA

>CYP162A5(2562410023)SDV

MSDTGQLVEAPDLADPALYRDGEPEAVWARLRARSPVYRNERVGKESFWAVLGHRAALDV

LRRPEVYASGRGMRLDDSPGATEAASGRMLIVTDPPRHAKLRHVMNAAFTPRTVARLRHA

MRATARGIVEEALERGRCDFVDVAARLPVAVICDLLGVPPADRAFMLDRTMTAFGHAGPG

SADRAAAARAHTDILVYYAELAELRRREPADDVVTALVQGTVDGRPLTDEEIYLNCDGLV

SGGNETTRHATVGGLLALMRDPGQWELASRPDHPMETTAQEILRFTSPAMHVLRTATEDA

ELAGRRVRAGDRVAVWLPAANRDPGVFPDPDRFDALRTPNRHVALAPGEHYCLGSALALA

ELTVFFEELTRRVRLPEPAGEPVRAASNLIRGFESLPVLLRGR

>CYP125A22(2562410179)SDV

MPCPALPEGFDFTDPDLLRHRVPFPEYAELRRAEPVRWIPQPGNIAGFQDEGYWAVTRHA

DVKYVSTHPELFSSWVNTAIIRFNEHIERDAIDAQRLILLNMDPPEHTRVRQIVQRVFTP

RAIRALEQRLRDRAQSIVIEARAHPGPFDFVTQVACELPLQAIAELIGIPQDDRAKIFDW

SNKMISYDDPEYAITEEVGAESATELIAYAMNMAADRKQCPAQDIVTTLVSAEDEGNLGS

DEFGFFVLMLAVAGNETTRNAITHGMHAFLTHPDQWERYKRERCETAAEEIVRWATPVNS

FQRTATQDTELGGKVIKKGDRVGVFYAAANHDPEVFENPDTFDITRDPNPHLGFGGGGPH

YCLGKSLAVLEIDLIFNAIADAMPDLRLTGDPDRLRSAWINGVKHLQVSLD

>CYP163C2(2562410198)SDV

MTLSPLLDAGPVEALDFADPLLHAHHDLGPVWRRLRAEAPVHWQPEADGRPGFWVVSGYA

EVAGVLGDSETYTSERGNVLDTLLAGGDSAAGKMLAVTDGRPHQSLRSALLKPFSPRSLH

VVVDSVRRGTRDLVARAVERGEVDFAADVAAHIPLAAICDLLGVPAEERGHIIDLTSTAL

SSADGAPTEEATWASRNGLLLYFSELAEQRRKKPYDDVVSLLVTREIDGRPLTHEEIVFN

CYSIIMGGHETTRFAMIGGLHALMHHPEQWQALKSGRADVSSAVEEVLRWTTPALHSGRT

ATQDVLLGEQFVEAGDIVTVWPASANRDESVFDRPDDFDLSRSPNKHLSFAYGPHFCLGA

FLARAELSALLESLKDLVAVAEPAGEPRQVYSNFLSGMSALPVTLTGERADAGQPLENG

>CYP107X6(2562410637)SDV

MDLADGLIDQPHTTNRRLRESAPVHRIAGTDGNPAWLVTRYEDVRAALNDPRLSLDKRHA

APGNYRGFALPPALDANLLNMDPPDHTRIRRLVVRAFTPRRVEQLRAPIRQTADRLLDAL

GSEGTTDLVAAYAAPLPITVICDLLGVPDGQRLDFRTWTDTLVAPDPARPGAAKEAVAAM

LAFFTGLLADKRRQPADDLLSDLIAVRDEGDRLTEDELMSLAFLILFAGYENTVQLIGNA

VLGLLRHPEQLAALRADPSRIPAAVEEFTRHEGPALLAIRRFPTEDVTIGGVSVPAGETV

LLSLAAANRDPARFPDPDRLDLDRDASGHLALGHGIHYCLGAPLARAETEIALTALLERF

PELALTEADVRWRPSLRARGLAALDVTYAKNLVTER

>CYP157C17(2562411111)SDV

MTPESHSLTGTDDPRSGPPPGCPAHGLGPGELRRLYGPDAEDLDDLYEELRDEHGPVAPV

LLHDDVPMWVVLGHAENLHMLRTPSTYCRDSRIWSPLLEGMVKPDHPLMPHIAWEPICSY

AEGDEHLRLRGAVTGAMSTIDYRGIRRYINRSTQRLVNQFCEQGRADLVGQFAEHLPMAV

MCEILGMPEEYNDRMVQATRDMLRGTETAIASHAYVMGTLQRLTERRRVEPAADFASQLM

SHPARLTDDEVTKHLWVVLMAAYEATANLIANVLRMVLTDPRFRAQLKGGQMTVPEAVEQ

SLWNEPPFSTVFAYYAKQNTELGGKRIRKGDGLLFGIAPGNVDPRVRPDLAANMQGNRSH

LAFGGGPHECPGQDIARGIADVGVDALLMRLPDVRLDCDEEDLTWRQSISSRHLVDLPVR

FEPKAQQDVMLRPGLNQVPAQRPAWQVSSAPPPQPQPAPQPAPMPAAAPPVPTPQPQRGP

SAWRRFVRWWRGY

>CYP158A7(2562411540)SDV

MTEETLTEILPPIRHWPALDLSGVDFDPVLTELMREGPITRIQLPNGEGWAWLVTRYDDV

RMVTNDPRFSREAVMDRPVTRLAPHFIPARGAVGFLDPPDHNRLRRSVAAAFTAKGVERI

REKSRRMLDELVDELLQDEPPADLSAAVLSPFPIAVICELMGVPAADRHIMHTWTQLILS

SAHGADVSEKAKNEMGAYFAALIGDRENSSAEDVTSLLGAAVGRDEVTTEEAVGLAVLLQ

IGGEAVTNNSGQMFYILLTRPDLAERLRAEPEIRPQAIDELLRYIPHRNAVGLSRIATED

VGIRGVRIRAGDAIYVSYLAANRDPDVFPFPETIDFSRSPNPHVSFGFGPHYCPGGMLAR

MESQLLVDALLDRLPGLRLAVPPEKVPFKKGALIRGPEALPVTW

>CYP1041A2(2562412111)SDV

MALDSPAFRFSRMESLLRGHRLRRLTVDGVDAMFVSDPVLVRRILVSDSKNYGKGELFRK

ARNLSRVGMLSEDEAMHRHYRRLANPYLRSTMVDDYVPTMRDIARAAVTAWRAGDTVDIQ

SEMCRITCAISVGTLISGLPPETTRELGERLSRLAWEMIRKPLYGKSASRAARTSAARRL

SRARTEFRDLLADCIAVLLNSPEARDGYLSSLLADSDAEGNRILTPDQVCEEAVMMMTAA

TVTTASVMSWALYVLSRNPLTEERLLKDLIRRRSGHAAHEHGTSSYTLRFLMEILRLYPP

VWISCRKTLSDVTLDDHALPAGTNVVFSSYLLHRRPDQYPDPNRFDPDRWLTHRIDPAEA

SYIPFGIGSKGCIGEPFAWQELEVVLDVVTQEWKLSADPDRQIRTAPETTLHPRRLLMVP

QPR

>CYP1058A1(2562412170)SDV

MRSGYATESRGLAWLHQCLAEERAESGLAFNPLRDAFQRAPEEVYAELRHRSPVHHSRLL

DCWVVTRYADVARVLRDHTVFRSSPDATTQDLVDPYVTLDPGRPSLFMLDPPDHTRLRGA

VREAFAPEALRRLTPRLEECVRHTVRALGRPGERVDLVPRFATLVPLRVFDLITGLDLHS

EDRVTDWVSDVVRGLEPIATARTAEQALTAYRALGACLDERRAAPAREGTLHFTLARDVE

AGRLSDAEARQLLMFLILAGTKTVSDFLAGAAAELTALPPGAPGRRRVDDALVDDLIVRT

SPVQIVARTAASPAVVGGRSVGVGDRVLLVLASANRDTGREGRDLAFGGGIHRCVGAQLA

RLEGRSALSCLLETYPEVRSAEAVPSRRCVTLRSWDRVIVHL

>CYP105D1(2555835311)SFI

MTEPMTDPARQDLDPIAHATAPTPATPFPQDRGCPYHPPAGYEPLRAARPLSRVTLFDGR

SVWAVTGHALARRLLADPRLSTDRTHPDFPVPAERFANVEQRRVALLGVDDPEHNVQRRM

LIPSFSVKRIGALRPRIQETVDRLLDAMERQGPPAELVSAYALPVPSMVICALLGVPYAD

HEFFEECSQRLLRGPGAADVNRARDELEEYLGALIDRKRTEPGEGLLDELIHRDHPDGPV

DREELISFAVILLIAGHETTANMISLGTFTLLRHPEQLAALRAGETTTAVVVEELLRFLS

IAEGLQRLATEDMEVAGTTIRKGEGVVFSTSLINRDADVFPRAETLDWDRPARHHLAFGF

GVHQCLGQNLARAELDIAMRSLFERLPNLRLAVPAQEIPHKPGDTIQGMLELPVAW

>CYP107F4(2555835587)SFI

MENTSVQNASVHDKETVRSCPFDFAQQLEFDPQLKELLTEEPVSRIRMAYGEGEAWLVTR

YEDVRTVTTDRRFSRSAVLGRDFPRMTPEPIVQAESINLMDPPASSRLRSLVAKSFTPRR

VDQMREGTQRIVDRLLDAMEDEGAPADFVARLSSPLPLITICEALDIPEADRPWLRAHAL

TMMNVGAAGKEDAVRAKAELRGYFTELTADRRRSPGDDLISTLAAARDGAELLDDKELAV

MAMVLLITGQDTTTYQLGNIAYTLLTRPELLKTVQAEPERLPRTIEELLRYIPFRKGVGI

PRIATEDVELGGVAIKAGDVVHVSYLTANRDAGKFDRPDELDPDRPSIPHMTFGWGAHHC

LGAPLATMELEVAFSTLLARFPALRLDAEPEDIRWNTTSIWRYPLALPVTW

>CYP125A19(2555836980)SFI

MRCPHLPDGFDFTDPDLLQARVPHPEFALMRQTAPVWWCTQPTNISGFGDSGYWAVTRHA

DVKYVSTHPELFSSNTNTAVIRFNETISRDQIEVQKLIMLNMDPPEHTRVRQIVQRGFTP

RAVRSLEAALRSRARSIVETALASADADGSFDFVTNIAVELPLQAIAELIGVPQQDRSKI

FDWSNKMAAYDDPEYAITEEVGAEAAMEIVAYSMNLAAARKECPAQDIVSRLVAAEGEGN

LSSDEFGFFVILLAVAGNETTRNAISHGMHAFLTHPEQWELYKRERPKTTAEEIVRWATP

VVSFQRTATQDLELGGQRIRKGERVGLFYSSANNDPEVFDAPEAFDITRDPNPHLGFGGG

GPHFCLGKSLAVMEIDLIFNAIADVLPDLRLLEDPRRLRSAWLNGIKQLQVSASSDS

>CYP107U8(2555837762)SFI

MDAEREAPNGTVVGVGNSVSWGAVHAPVVQVGSVSGGVHTYYAQTPYSSSLPPVSEWPGR

RSRPPRRRPDPPPTPESHRHPARYRQGVTDTPACPHHTSATGEEAPELFTWEFATDPYPA

YAWLREHSPVHRTVLPSGVEAWLVTRYGDAKQALADARLSKNPANHAGSAAAKGKTGIPG

ERKAELMTHLLNIDPPDHTRLRRLVSKAFTPRRVAEFAPRVQELTDRLIDRFIEDGEADL

IHDFAFPLPIYAICDLLGVPREDQDDFRDWAGMMIRHGGGPRGGVARSVKKMRGYLAELI

HRKRENPGDDLISGLIRASDHGEHLTENEAAAMAFILLFAGFETTVNLIGNGTYALLRHP

EQRARLEASLEAGESALLATGIEELLRFDGPVELATWRYATEALTLGGQEIAAGDPVLVV

LAAADRDPDRFADPDALDLSRSDNQHLGYGHGIHYCLGAPLARLEGQAAVATLLRRLPGL

RLAGESADLRWRGGLIMRGLRTLPVEFEPGRRLEESDTLSPL

>CYP107P7(2555838323)SFI

MHVSFDPWSPAFVADPYPAYAALRAAGRAHYFEPTGQWLIPHHSDVSALLRDRRLGRTYL

HRFTHEEFGRTPPPAAHEPFTTLNGQGLLDLEAPDHPRIRRLVSKAFTPRTVENLAPTVR

RLAAELVDAFVAKGGGDLLAEVAEPLPVAVIAEMLGVPEEDRGPLRPWSAAICGMFELNP

SEETAAAAVRASEEFSAYLRGLIAERRKAPGDDLISALIAAHDEGERLTEQEMVSTCVLL

LNAGHEATVNTTVNGWRTLFHHPEQLAALRAAPASLPAAVEELLRYDTPLQMFERWVLDD

IEVDGQVIGRGAEVALLFGSANRDPERFAHPDTLDLTRPDNPHVTFGAGIHYCLGAPLAR

LELTASFGELLRKAPAMRMVAEPEWHPGYVIRGLKELVVEV

>CYP156B5(2555838431)SFI

MDPSPGATPYSAPAGCPMHQKQTSLYGPEFAADPHRVYDAFRAHGPAAPIELAPGVDATL

IVQHEAALRVLQNPALFARDSRRWAALREGAVPMDSPVLPMMAYRPNCLFTDGAEHLRLR

KAVTESLARLNSSRLSRDVERIADYLIDQFIERGTADLLNEYAKLLPLLLFNQLFGCPGD

IGDRLTRSMSAIFDGEDVLRANAELTECLMELVALKRRQPGEDITSWLIQHPAGLRDEEL

KDQLVMLMGAGVEPERNLIANALLLMLAGDQPGAPERRGSGMLVEDALDDVLWNNPPIAN

YATHYPVRDVELDGVTLKAETPVLISFAAANTDPSLTDARQTLSKGAHLAWGAGPHVCPA

KSPATLIALTAIEKILNTVPDLSLAVPASGIGWRPGPFHRAMIALPVRFTPTAARRTTTG

AQPPAQTSAQLPDPYRNAAPQPGAAPRHAAEPAKKPKGWWSSFLDVFRV

>CYP154C4(2555839288)SFI

MTRIALDPFVTDLDGESAALRAAGPLAEVELPGGVHVYAVTRHAEARALLTDSRVVKDIN

VWGAWQRGEIPMDWPLIGLANPGRSMLTVDGADHRRLRTLVAQALTVKRVERLRAGIEAL

TNASLEKLAALPAGQPVDLKAEFAYPLPMNVISELMGVDAADHPRLKELFEKFFSTQTPP

EEVPQMMADLGALFTKIVDAKRANPGDDLTSALIAASEDGDHLTDEEIVNTLQLIIAAGH

ETTISLIVNVVEALQTHPEQRKQVLNGEIGWDGVIEETLRWNTPTSHVLIRFATEDIEVG

DRILPKGEGLIISFGALGRDEEQYGPTAAEFDATRTPNRHIAFGHGPHVCPGAALSRLEA

GIALPALYERFPELELAVPASELRNKPIVTQNDLYELPVELGCPFGHDA

>CYP157A6(2555839289)SFI

MTTTSGCPVTHGSVPLSGPRFQSDPVQLYRDLRRDHGAVAPVVLDGDVPAWLVLGYRELH

QVTGDPVLFSRDSDLWNQWDRIPDDWPLLPMIGRKQPSILYTVGERHSVRAMMISNALEG

VDPFSLKRYAEEFADELIDRFSTKGSVDIIAEYAKLLPALVLARIYGFSDEEAYPLVGAI

NDMIDGRERALAGQQHLATSMFQLLADKHAEPGDDVATRMIADPGGFTDEEVAQDLMVMM

AAGHQPTADWMGNSLRLMLTDDRFAASLSGGRHSVAEAMNEVLWEDTPTQNVAGRWAARD

THLGGRHVRAGDLLLLGIAAANGDPQVRTHASALTGGNNAFLSFGHGEHRCPFPAQETAE

VIARTGIEVLLDRLPDVDLAVPAEQLTRRPSPWLRGLTDLPVLFTPTPALGRPGSFGGPA

>CYP1046A4(2555839315)SFI

MIEDGGGARGCPASREEPRVAHSRTLRFWLDPANIAVRLGQAGPVVRTKAGPAVAFQVND

PSLIRKVGCGEDIFQAWSADPCLRDFTGDGFAGAEGQAHRDRRALMKPALAAPRLTALGP

SVRDSTEGLLAGLPADRPVDVPVEMSRLVAGLVIETVLNSGISPDTLAALARARSVLSAG

VFWKYALSPWPWVPAPRGRAFRRALGTLNEATQEVYERHQPDGDGNDVVSLLKRSSDNRP

DAALHDLRALLFAGIEATASTLAWACYELGRHPDHQAAIRAEADAVLGADAPADAVRPEQ

MPRTAGFIREVTRLHGIPFLVRRPRHATYVGEQPVPAGALVTLPMGALRRDPARYARPEE

FDPLRWSPEARPQLSPAALLPYGLGPRYCPGAAASEILTPVALASLVRSRTLRTTTPDRT

VRVSLELTPMPKGLTMLAAPREPHSPLVPGPTSSEEADPARRP

>CYP107AE4(2555840847)SFI

MTAPRPRQDPLRDPRFFADPYPTYDQLRERCPVQRIPTGSGGHHAYLITGHPEAREAFTD

PRLSKDTARFFAERPSNRDLHPAISRTMLASDPPTHTRHRRVATPLFTTGRVRALRPYIT

RVVDDLTTAWQPGKEVDLVAELALPLPITMICELLGVPEPDRAELATWSHALFDATDTNI

VDAASHRIGDYLTDLVDTARATPGEGPLHSLVRDCEEGDLDRDETVSLAALLIVAGHETT

THFIGNAVLALLRHPEAFDRLRRDPDLIPGALDELLRYDSPVSVATFRHSTENLSVGGTD

IPAGFPVLIAPGAANRDPAQFPHPDHLDLDRDATGHLSFGHGIHRCPGAPLARAEAETAL

RTLVTRFPNTRLAVPAESLTWRRTRLTRGLATLPLILG

>CYP159A7P(2555841100)SFI

MPADRSEALPTARRPHRLAGGQPGLLEPGASTDPYRLRLYRVLRTDFPLGYDPGLGAWLL

SRYADVALALTDPRFTGYPHDGAPRGPVPAPLGLCRGSLVCTPLPDGVTAPGPSADSSPR

ATPASMAATLSAVERTAYVLARRIAGWDRADLVGEFCRWLPAGADAVAAGLSYPDLNAPP

RHAAHQRPGGGTGDCARHTGLRERALASFLANMLDDPDLLAAAAGSGAGAGMLLGRAWTE

TLRRDPPVQIVLRRTRTEVRVSGGTLPGGAPVACLIGAAGRDPARFAAPDRFDPLRADAD

PLLTGPAGCPAALLGRLEAEHGLRALLTAMPRIRWAEGFRPASSGVLTRGPRTLLVRPS

>CYP107L16(2555841259)SFI

MAVLDLRDLPDFTANPYPYYAKLRAEGPVHAVRTEQTERIWLVVGYEEARSVLADQRFGK

DWRTTGRWAAEANPISANMLELDAPHHTRLRRLVVREFTARRIEALRPRVTEITTELLDA

MVPAGAADLVDALAFPLPMTVICELLGVPDIDRDTFRTLSNGIVTPTPAQREADPVGAMS

AYLVQLIEDKRSSPGDDLLSALIRSRDEGGDGLSADELVGMAFVLLVAGHETTVNLISNG

VRALLDHPDQLALLRADPGLLDGAVEEMLRYDGPVETATFRFTREAVPVGSTVIPADEAV

LVALASGGRDPERFEDPDTFDIRRAPQGHLGFGHGAHYCLGAPLARMEARIAIGALLERC

PDLARDPAGGEPEWLPGLLMRGVRRLPVRW

>CYP107L17(2555841260)SFI

MTTEATPGTDALVDLAALGEEFTRDPYPVYAALRAKGPVHRVRIPEGTQAWLVVGYEAGR

RLLADQRLSKQWAAASPALGVTKVSAGTSMLSSDTPDHTRLRKLVAREFTPRRMEQLTPR

VQEMTDELLDRMLAAPDRRADLVEALSYPLPMNVICELLGVPFLDRAAFREWSNQAVSSV

DASKRASSTQAMAAYLTGLLQDKRARPGDDLMSALIHTADEDGDRLSADELMGMAWLLLV

AGHETTVNLITNGVHNLLAHPEQLAALRADFGLIDNAVEEILRFEGPVETPTYRFTTEPV

DIDGTVIPGGGELVLVAMSDANRDPARYSDASRFDITRDARGHIAFGHGIHYCLGAPLAR

IEARVAIRSLLERCPELRLTADPATLAWRTGLLMRGPLSLPVAW

>CYP157A7(2555841465)SFI

MTNPPPATPAPPAGGGGGCPMGSGTGAVPLSGPGFHTEPQALYRSMRRDHGPVVPVELPG

GFPAWLVIGYRELHQVTSDGELFPRDVSLWNQWENIPADWPLLPMVGTPMPSIYFTAGAE

HRRHVEMVVPALEAADPFEIRQHCEQLADRLIDAVCSRGTADLVAEFAVPLPVLVLARLV

GFPDDEGADIAQVLKDLADGGPGAQKAHLRFGEHMSRLVAAKRVHPGDDVTSRMLAHPEP

FTDEEYALDLMAITAAGHLTTADWISNSTRLMLTEDQFADALSGGRHSVAEAMNEVLWED

GPTQILAGRWASRDARLGGQNIARGDMLLLGLGAANADPHIRQQVTDQVVRSGQGGNSAH

MAFSHGEYRCPFPAQEIAEIIARTGIEVLLDRLPDLELAVPPTELVRRPSAFLRGMTALP

VRFTPVRTTGDAL

>CYP154C3(2555841466)SFI

MNCPHTGAAQTDRGAGTITIDPMVQDLDGETTRLRDAGVLARIELLGVPAWTVTRHAEAR

QLLVDPRLVKDIDAWGLWRSGVVTRDWPLIGMIDAGRSMFTVDGAEHRRLRTKTSQALTP

RRLEAIRPAIEKFTDELLDNLDAARGEDGTVDLKAVFAQPLPMKVVGMLMGVDESQHAML

TRQYKAFFSMLTPQDQRLALLAELDVFYTDLVREKTANPTDDLTSALILAEEGGEPLTEE

EVVGNLKAMVAAGHETTIGLILNAVRALLSHPDQLEKVLAGEIGWDAVIEETLRWDTPTT

HLLMRFATEDITVGDTVIEEGEGVVISYRAIGRDIEQHGADADAFDITRATRNRHMTFGH

GPHICPGAALSRVEAAVALPALFARFPGLRLAIPDAEISKLPVMTQNDMAAFPVLLG

>CYP1047A3(2555841473)SFI

MSTQTGPELDTPSRGHALVPGPRGLPLLGNLPQFGKNPLAFFELLRGHGDMVRWRFGRNR

CVFISDPECIGELLTETERTFDQPRLGVAFRAVMGNGIIVARGRDWRRKRSLVQPSVRPK

QVKSYATTMASSAVELADAWSDGERVDVKKEMAALTQKIAVRTIFGVDTPADSEAMGRAM

DVAQLEIGKEFAGIGALLPDWVPTPGRARIRKAAAVIDSEVGRVVARHRDGETERPDLLS

RLLTAVDESGERLSDEEIRDETVTLYIGGHETTSSTLVWAWYLLARNPRVRAALAEELDR

VLGDREPGFEDYAQLTYTQAVVKETLRLYPTIWLITGVAKEGARLGGLPIPEGTRVWSSQ

WSTHRDARWFPEPEEFRPERWDATNGDEIAEYAWFPFGGGPRVCLGTRFAMVESVLILAV

LARRFELDVEPGVVEPVPSLTLQPDRDVLATVRAR

>CYP157C13(2555841492)SFI

MAILIPGATVTTPFHHEPGVSPPPQCPAHNLGTGPGGLRRLYGPEAENDPAGLYDKLRAE

HGTVAPVLLHGDVPAWLVLGHSENLHLTRTPSQFSRDSRRWRALQDGSVAPDHPLAPIFT

WQPVCVFADGAKHERQRGAVTDSMERIDTRGVRRHINRFSNRLVNDFSEKGSADLVSQFA

EHLPMMVMCAIFGMPEEYDERLVQAARDMTRGTETAVASNAHIVSVLTRLVERRRAEPAP

DFASWLVEHPATMSDIEVVEHLRLILIAAYESTANLIANVLRMVLTDPRFRARLSGGHMT

VPEAVEQTLWDEPPFTAVFGRWAVGDTELGGQQIKAGDALLVGIAPANTDPTVRPDLTAN

MEGNRAHLAFSGGPHECPGQDIGRAIADVGVDALLMRLPDLELGTEESELRWVGNIMSRH

LVELPVKFAPSPQQKLDSDPLSVMARAVRPTGDWEISSPARPVPEPAHSIAGMQPAHAPG

AAPQHRPAAAPAETPAPATGEPAPGTAVVPQQRRPAAPARLWQAVSRWWNGY

>CYP107BX3(2555841682)SFI

MTTTDPTGTDPSLIPLHCLRFEEPGPPRLDELPGGAPAWLVSRHADVRQVLSDPRFGRAQ

LYAADAPPLSDVPDLVNNPDLMFNQDGPDHLRLRRTLRRAFTPRAVARWRPWIAAIVEQL

LDRLEGRSGPVDVVEEFTLPLPVAVISRLMGLDDSARDRMRHWSEHAFSDGSHSGEEVES

VLKEFSAFGAELLAERRRDPGDDLISSLVRAADEEGGLPEAQLVSLVCGLVVGGHDSTMT

MLGNALLFLLGDRPESWPRIGADGEAAGRVADRLIHLIPLGDDRGTARHASTDVEVGGVT

IPAGAVVLADCGAANRDPEVFPRHTLDDLFAPLEAPTLSFGAGAHYCLGAWLARTELQIA

LHRLAARFPGLHLSEPPDSVVWRTGTTSRSPRRLHVSW

>CYP124G2(2555841898)SFI

MTVPNQVSGDRAARIDLADPAFWRLPRPERLGAFARLRELEAPVLFTPRPGTARTSGRPF

YALVRHADVLTASRTPKVFASAPGVTTPEPAGWAKALFGNSMVNMDGPEHAALRRMISRR

FTPRLLAATEENIGRLAGRLVDELISERPGDFMPSAASRLPLEVICDLMGIPAAYRPRIA

EQIDHASEQVGVARRGRARLRIPGRGTASLARMQLVMARLARERRRHPQDDLVSALVCAD

IDGEALSSRDLGAFFSLLLVAGVETTRNAIAHGLSLLDRHPEQQELLRSDFDRYIGGAVE

EIVRHSTPIIQFRRTVVSEFPLGGRTFLPGEKVSLLYASANRDESVFTRPDLFDITRSPN

PHLGYGGGGPHHCLGAHLARLEMTALFRELLTRRSVIRRTGDPLLVDSNFDNRVGSLPFT

FGPTVT

>CYP107P16(2630312046)SGU

MAAFDPWDPAFLADPYPAYAELRARGRVHHYEPTDQWLVPHHADVSALLRDRRLGRTYLH

RFRHEDFGRTPPPPEHEPFHTLNDHGMLDLEPPDHTRIRRLVSKAFTPRTVERLAPYVEA

LAGELVGGLVRRGGGDLLTEVAEPLPVAVIAEMLGVPESDRAPLRPWSAQICGMYELNPS

EETAAGAVRASVEFSDYLRDLIAERRKEPGDDLISGLIAAHDEGDRLTEQEMISTCVLLL

NAGHEATVNATVNGWWALFRNPDQLAALRADHSLVPSAVEELMRYDTPLQLFERWVLDDI

EIDGTTIPRGAELALLFGSANHDPAVFTDPGRLDLARAENPHISFSAGIHYCIGAPLARL

ELAASMRALLERAPTLTLAAEPRRKPNFVIRGLEGLTVEIR

>CYP170A22(2630312892)SGU

MTVESVKPGAPRAPQAPALREPPLAAGALPLLGHGAKLARDPLAFMSGLREHGDVVRLRL

GPKTVYAVTTPELTGALALSPDFEIDGPLWESLEGLLGKEGVATANGPRHRRQRRTIQPA

FRLAAMPAYGPVMEEEAHALTTRWRPGETVDCTSESFRVAVRIAARCLLRGDCMDERAER

LCTALAIVFQGMYRRMVVPLGPLYRLPLPANRAFNRALADLHLLVDEIIAERRASGRKPD

DLLTALLAAKDENGEPIGEQEIHDQVVAILTPGSETVASTIMWLLQALAEHPEHADKIRE

EVETVTGGRPVAFADVRALRHTNNVVVEAMRLRPAVWILTRRAVTDTSLGDYRIPAGADI

VYSPYAIQRDPRTYPDHLDFDPDRWLPERAGEVPKYAMSPFSVGNRKCPSDHFSMAQLTL

ITAAVATKYRFEQVPGSDDATRVGITLRPQRLLLRPVPR

>CYP154B5(2630313509)SGU

MNLTSGVHTLDVTGRDLAAEAAMLRARGPAVQVELPGGVRAWAVVRQSQVERLLLDPRVS

KDARRHWPAFVEGRITPDWPLFPWVANENMLFAYGEEHARLRRLVAGAFTARRTAALQPR

IEEITVALLDAMEAASEDGRAELRSAFAESLPLQVVCELFGVPEGEARAELCAALRVVFS

SSVTAETMETARATAFRLLAELVAAKRAAPGDDLTSSLIAARDDGDRLSENELLGTLFLM

IAAGQDTTSTLIVNGVGSLLADPEQLAHVRAGRAGWEDVVDETLRVHSPAAYSPMRFAVE

DIDLDGVLIRRGDPILVNFAAGGRDPERYGDDADRFDLLRTDRDVLGFGHGVHRCLGAPL

AVLEATIAFRALFARFPDLALGCPVEELRPLPTFLLNGFGALPVVLRR

>CYP180A9(2630313618)SGU

MTRPGAPPVPDVFDPRRYATAVPYDAYRVLRDHHPVAWQEEPEVLGWPAGPGFWAVTRHA

DVVRVLKDAAAFSSYLGATQIRDPDPDDLPFIRRMMLNQDPPHHNRLRRLVGRAFTPGRI

ERFTAVAGERARTLLARAVTEARAGDGTVDLVAAVTDDYALLNLADLLGVPESDRHLLLH

WTQRVIGYQDPDEAGPAVRDERGNPVNPRSPAALRDMFDYAQRLAAHKRRHPGDDVLTTL

ARDGELAGAELEMFFFLLTVAGNDTVRAAAPGGLLALAEHPGEYERLRSGSVPLASAVDE

LLRWHPPVLTFRRTAVRDTVLAGRRIRAGDKVVVCHAAANRDERVFADPDRLDLGRAPNP

QVSFGDGPHVCLGAHFARLQLRVLYGEALRALPVLRTAGAPGRLVSNFVNGIKSLPMRIV

P

>CYP107L34(2630313774)SGU

MGDIIDLGDYGEGFRADPHPVYAALRARGPVHRVRLPAPADYESWLVVGYEEARAALADP

RLAKDGSRIGLTPLDEELIGPYLLVTDPPRHTRLRALVTRAFTPRRVELLRPRVQRITDD

LLDAMLPLGRADLVESLAYPLPITVICELLGVPEPDRAEFRRLSTETVAPTSTDSGQDAI

TRLGAYLTGLIEDKRRAGPGDDLLSALIETTTEDGDRLSPKELRGMAYLLLLAGHETTVN

LITNGVHALLTHPAQLAALRADTSLLDGAVEEMLRYEGPVENATFRYAAEPLEIAGTRIE

AGEPVLIGLTSGNRDGTRYPGADRFDIRREPRAHLAFGHGIHYCLGAPLARLEARTAIGT

LLDRAPGLTLDGPPGAWLPGMLIRGMRSLPVRW

>CYP107AH4(2630314172)SGU

MTQAFASEEMSSEEAAAAAASCSREFRANPHPVYARLRETAPVCPMSPPHGVETYLITRH

DDARAALADPRLSKDMYGAIDAYHRIFGDSSIALDDNMLFSDPPKHTRLRRIVGNTFTPK

RVQSLRPRVQQITEDLLDACPASRPVNLLPEFCFPLPLHVICELLGVPQNERKQAQEWSA

TVAQTGFGPEARARLEVAEGNLRDYLVDLIARKRREPDDGLLSALVEAHDQEGALTDHEL

VSTAWVLLFAGHKSTAYQLGNALFHLLTQPEQKRLALRDEQSMAAAVEEIFRFETSVENG

TFRYAKEDVVIRDTLIPKGSLVQVSIAGANRDPEVFEDPDRMDVERPNAQADHLAFGFGP

HYCIGAPLARLEMHLALTTLFGRFPRVTLASAPEDVPWLTVPFPAFRGVAELPVVLDPS

>CYP157K3(2630314387)SGU

MNDQTPSDPAPPRGCPFGHGGGDLTRLYGPEAATDPAGIYERLRTEHGPVAPVLLEGDIP

AWLVLGYRDNRRVLDTPWQFSRDARIWRDWREGRIAETSPLVPMLGWRPDCVSQDGEPHR

RLRGAVTDGLQAAAARGIRRHVTHFANKQIDAFAGTGRADLVADYAEYLPMLVLTRILGL

AEGEGRLLVESCAQVLKGGEEAVAHNERIMEKLGELAERKRSEPGSDFTTALLEHPAALD

AEEVISHLRLVLIAAHTTTSNLLARVLQLVLTDVERLSGLIGGRLNISAVVEEVMWNTPP

LAVMPGRFATADLELGGHRIAEGDLLVLGLAAGNLDPEIRPDAGVSVQGNQSHLAFSSGP

HECPGQNIGQAIIETGVDVLLHRLPGLRLAVPAEELTATASTWESRLDSLPVEFAG

>CYP1005B6(2630314428)SGU

VTSPAPDAAPPAPPLDLLDLFGEGFVADPYPWLDALRAEAPVHHDPRTGLWLVSRYADIR

RVLLDPGAFRPDNAQHAVTPLPVGALRVLARARFGLPPALANNGTPSHPGLRRLVSRFFD

ARRVAAAVPVIERITDELLEAAGAHLDASGGGDLFTSFAQVLPCRVLMELLGVDGVPAAT

LIRWSDASLELFWGRPTPERQLELAPLVAEFHQWLTRTVRAGTAPPGSFVAALARHRLPD

GAPLDAETAVGACFFVFVAGQSTTGQLIATVLRRALAEDGLWPRLADEPGLAEAWVEEVL

RREPPVTSWRRVTARPVILGGVDLPEGAQLLLMLMGSGSDPEVFSAPERMCPHRANIRQH

LAFGVGRHRCPGASLARTEAAVALRRAARRLPRIRPAEDGDPPMLGLLSFRAPLRVAVEQ

R

>CYP113K5(2630314647)SGU

VTSTQLTGAESPLDVLPERWRALRDAGPVTYDEERRQWHVVDHQGVSAVLSDPATYSSDM

TPIAPTQEDFEAFRQGNFVGMDPPEHRKLRTLVSQAFTPRVVHGLAPRIEAVCARLLDGV

ADRDRFDLVDTLAYPLPIIVIAELLGVPAEDHRLFQEWAATLFGGDQLGDSLDMADLERA

LEAIAPTVREMNGYVLDHIRHRRAHPGDDLTSRLLTAEVDGTRLRDEEIVGFVALLLVAG

HITTTALLGNAMVTFDRHPGTLTALREDPGRVPDAVEEVLRWLPPFPELGRRTTRPVVLG

GHDIPADTLLMVHLGAANRDPSRFTSPDVFDAARDPNPHLTFGHGIHFCFGAPLARLEAR

IALQMMMERFPSLAVPSYDDVTYQNPAVIVGVRHLPVAVTRP

>CYP154C1(2630314722)SGU

MTPGTADVRIPLDPFVTDLDGESAKLRAAGPLAAVELPGGVPVWAVTRHAEAKALLTDPR

LVKDINRWGAWQRGEIPADWPLIGLVNPGRSMLTVDGADHRRLRTLVAQALTPRRVEEMR

GRITELTGRLLDALPADGGVVDLKAAFAYPLPMYVVADLMGIDESRLPRLKVLFEKFFST

QTPPDEVVATLTELAGIMAETVAAKRAAPGDDLTSALLSASEDGDRLTDEEVVSTLQLMV

AAGHETTISLIVNAVVNLSTHPEQRALVLSGEVPWPAVIEETLRWSTPTSHVLIRFATED

VPVGDKVLPAGDALIVSYGAIGRDELAHGPTAGDFDITRETRTRHISFGHGPHVCPGAAL

SRLEAGVALPALYARFPDLDLAVPASALRNKPVVTQNDLFELPVRLTP

>CYP157A17(2630314723)SGU

VTPDAVPLSGSRFQTEPARLYREMRRDHGPVAPVLLDGGIPAWLVLGYRELHQVTGDPVL

YSRDSGLWNQWDNIPDDWPLLPMIGRDQPSVLYTVGERHHQRAGMIAKALEGVEPAVLRR

HCERFADELIDAVCTAGTADLVGQYAMLLPVRVLARLYGFSDDESSGLVTALNDMIDGRE

RALDGQRHLGRSMAELVAQRGAAPADDVVSRMLADPNGFTEEEIIQDLMVMTAAGHQPTA

DWIGNSLRLMLTDDRFAASLFGGRHSVAEAMNEVLWEDTPTQNVAGRWAARDTQLGGRHV

LAGDLVLLGLQAANADPQVRTDAAAFTGGNSAHFSFGHGEHRCPFPAQEIAEVIARTAIE

VVLDRLPDIDLAVPADSLTRRPSPWLRGLTELPVRFTPTPALGGNRP

>CYP178B1(2630315081)SGU

MLVVEDSPLYSPEFFADDDPWAYLAGLRADHPVSVHRREDGYEFYALTRYADIYAAYVDH

KRLSSSYGTMVDGSYLPQKDSASGRMLIVTDQPAHTALRKPVKTSGFSREMLGRVGRTVR

RNIRDALGKLSVGDRLDFSTVVAPELPKGVLEVLFGIGPGDAHRLLDATRTMIGYRDEVY

AGTSPLDALVDAQLEVLEFIDDLIGHRLSSGRADDMIGFLAQCVADGTMPRDVAVLNGLN

VAVGGNETTPHTASLTVHTIDGEREQWRRVADGDVGCDVATQEFLRWTSTNSYVQRLAVE

DFPVGEHVIPAGSFVTLWNMSANRDPDVFERPDAFLIDRSDNKQLAFGAGVHRCVGAPAA

TLEIQTFLEELAAWDKAFAVVAPPRRLRSNFMLGLTELQVEVVDAKEFGT

>CYP152D11(2630315171)SGU

MDAPRPLVDRSLPMLAEGYAWLPNRMRESTGQVVRTRVMGRPALAVRGPEAVRFFYDERH

VRRHGAMPGPVLSTLFGHGAVHTYDGDAHRARKDLFLPLLHVDRIAGVVEHVTAAWDDAV

ATWPGRSRVVLLDEAAVVLTRGVCRWAGVPLADEDAEPLARDLTAMVDGFATLGPRHWRA

RSARRRQEARLSRLVEEVRSGAAHAPADSVLDRVCRHRHADGELLEPRTAAVELLNVIRP

TAAVSWFVAFAAHALHRWPANRERLRDGDRAYAAAFTHEIRRFYPFAPFLGGRAVTDLSW

HGEQVPAGGMVLLDVYGQNHDEKLWGDPYAFRPERFLERPVQRDELIPQGGGDPGTGHRC

PGEGMTIGLLEALTTRLARLEYEVPEQDLTISLRRIPARPRSGFVIGDVRPPGV

>CYP107L30(2630315326)SGU

MDVVDLSALGESFTRDPYPVYARLRAQGPVHRIRMPEGGAIAWLVVGYEAGRAALADPRL

SKDWSNASPALPLDAVSSGPHMLRADPPDHTRLRKLVAREFTARRVEGLAPRIQKTTDAL

LDRMLAAPDGRADLVEALSFPLPISVICELLGVPDLDRESFRTWSNDAIGATDPTVRKTA

AASMARYLTTLLEVRRQQPGDDLMSALMHGADDDGDRLSPEELLGMAWLLLVAGHETTVN

LISNGVLALLTHPDQLAALRADPSLIDNAVEEMLRYDGPVETPTYRFTTEPVTIGGTVVP

GGGELVLVALADANRDPARFDSPDRFDITRDARGHVAFGHGIHYCLGAPLARLEARIAIG

SLLDRCTDIALDIHPAAIMWRPGLLIRGPRSLPLRFTR

>CYP183X1(2630315733)SGU

VSASSRKSEQQWTVECAPGAFPLLGHGIALFRRPLAFLNSLPAHGDLVEVRLGPRRAWVV

CHPELAHCMLLDASTFDKGGTLYDRLRSLMGDGVVTCPHERHRRQRRLLQPAFRPSRVAA

HADLMADEAVAVSGRWRAGEEVDVSAAMMTVTARVMSRVLFSDSLDAATHAEVRRCLADV

VRGLFLRTIVPVDAVFRIPTPANLRYRRSVARLHAIVDAAVAERRGGGAHRDDLLGTLLA

AAARDEDGAVSDREVHDQLITLLLTGVETTALCLASAFDLLARHPQAERALHAELDAVLA

DGRRPGAEDLPRLDRTRAVVTETLRLSPPGWLFTRVTTRDAELAGRRLPRGTTVLYSPYL

LHHDPGSFPEPERFLPERWLAGRTAARREHALVPFAAGSRKCLGDGFAMTEATLALAAVA

ARWRLAHRSGPVPRSRPAVTLGPRSLWMTCVPRTQRRTGEAHRAALRAADLRRAGDNGVA

DA

>CYP163B10(2630315836)SGU

MSQPALSVRSLDLTDPATFIENDIHEFWRDVRARQPVYWHEPTDRNPGFWVVSRYADVQS

LYHHGALSSARGNVLDVVLRGDDSAGGSMLAVTDAPRHRSLRNLMFSAFTPRVLGEVVEK

VRKRTDDLVSAVLEKDTFDFAAEVAEQIPMNTICDLLSIPAADRGALLQWNKMALSSGHA

DSSELDALGARNEIVLYFMELAQERRDNPGDDVISMLATSEVDGRLLTVEEVAVNCYSLI

LGGDETSRISAICAVLALIENPDQWQALRTGEVSFESAVEEVIRWATPGIHLARTAVEDF

EIRGAKVRAGDIVTLWNISANNDESVFDQPRRFTLSRSPNRHLSFGHGPHYCLGAYLGRG

ELRALLSALVEHVGGMELRGTPRPIYSNFLTGYESLPVHFEGR

>CYP157C25(2630316243)SGU

VTSDIPTRTGTDDLALAPPPGCPAHGLDGGALTRLHGPRPLDLRSLYEDLREEHGPVAPA

LLHDDVPIWVVLGHAENLRLVGTPALFSRDSRIWTPLVEGRVKPDHPLMPHIAWQPICSH

AEGDEHRRLRGAVTHAMRPINYRILRRAVYRHAQALVNEFCERGEADLVNEYSEYLPMAV

MCDLLGMPTEYDDRIVHAARDMLKGTETAIASNAYITEALARLTARRRAEPGEDIAGLLV

SHEWGLTDDEVREHLRVVLIAAYEATANLLANALRMVLTHPGFRAQLNGGQMTVGQAVEQ

SLWDEPPFSTVFAYFAKQDTELGGRRIRKGDGLLFAPAPGNVDPRVRPDLKAGMRGNRSH

LAFGGGPHECPGQDIGRVIADVGVDALLRRLPDVRLDCEEDELEWRESIASRHLVELPVR

FATRPQQDVEQMPSHTPNPLEGPSWEPPPAEPVPSPSAAPAPRSGPEPEPGPQPQPQSQP

EPEPGPRPAQRSAWQRFLRWWRGY

>CYP107U1(2630317729)SGU

VTDQPHPPTPAPELFTWEFATDPYPAYAWLREHAPVHRTRLPSGVEAWLVTRYADAKQAL

ADQRLSKNPAHHDEPAHAKGKTGIPGERKAELMTHLLNIDPPDHTRLRRLVSKAFTPRRV

AEFAPRVQELTDRLIDGFAAKGSADLIHEFAFPLPIYAICDMLGVPREDQDDFRDWAGMM

IRHGGGPRGGVARSVKKMRGYLADLIHRKREALPAEPAPGEDLISGLIRASDHGEHLTEN

EAAAMAFILLFAGFETTVNLIGNGTYALLTHPGERRRLQRSLAAGERDLLATGVEELLRY

DGPVELATWRFATQPLTIGGQDIAPGDPVLVVLAAADRDPARFTDPDTLDLARRDNQHLG

YGHGIHYCLGAPLARLEGQTALATLLTRLPDLRLAADPAELRWRGGLIMRGLRTLPVEFT

PER

>CYP211A1(2668573307)SGB

MAGLVMSPVEALDALGTVQGRQDPYPFYEAIRAHGQAVPTKPGRFVVVGHDACDRALREP

ALRVQDARSYDVVFPSWRSHSSVRGFTSSMLYSNPPDHGRLRQVVSFAFTPPKVRRMHGV

IEDMTDRLLDRMARLGSGGSPVDLIAEFAARLPVAVISEMIGFPAKDQVWFRDMASRVAV

ATDGFTDPGALTGADAAMDEMSAYFDDLLDRRRRTPADDLVTLLAEAHDGSPGRLDHDEL

MGTMMVLLTAGFETTSFLIGHGAMIALEQRAHAARLRAEPDFADGYVEEILRFEPPVHVT

SRWAAEDLDLLGLSVPAGSKLVLILAAANRDPGRYPEPGRFDPDRYAPRPGGPEATRPLS

FGAGGHFCLGAPLARLEARIALPRLLRRFPDLAVSEPPVYRDRWVVRGLETFPVTLGS

>CYP208A1(2668573323)SGB

MRIDPPGPPLRALPGLLRKLAVDRLGMMRDAAGLGDAVRVSMGPKKLYIFNRPDYAKHVL

ADNSDNYHKGIGLVQSRRVLGDGLLTSDGETWREQRRIVQPAFKPGRINQQAAAVAEEAA

KLVALLRGHEGGGPVDVLQEVTGLTLGVLGRTLLDSNLTAHESLAHSFEEVQDQAMLEMV

SQGTVPAWLPLPPQARFRRARRELYRVADLLVADRRSRMADGGPGDDALSRIIVAADRRR

DDPARARNRLREELVTLLLAGHETTASTLGWTLHLLERHPEVRDRVRAEARAALGDGVPG

PEDLHRLTYTTMVVQEAMRLFPPVWILPRVAQQRDVVGGYTVSAGSDVLVCPYIMHRHPG

LWEDPERFDPERFEPRQTADRPRYAYIPFGAGPRFCVGSNLGMMEAVFVTALVTRDLDLR

TVAGHRAVAEPMLSLRMRGGLPMTVSTAR

>CYP159A5(2668574152)SGB

MTVRAPDILSPEFERDPYAAYRRMRQDAPLLWHEATKSYIVSRYEDVERVFKDKAGEFTT

ENYDWQIEPVHGRTILQLSGREHAVRRALVAPAFRGADLRDKFLPVIERNSRELIDRFRD

SGSVDLVAHYATRFPVNVIADMLGLDKSDYERFHGWYTAVIAFLGNLSGDQDVARAGERT

RVEFAEYMLPIIRERRESPGEDLLSTLCTAEVDGVRMSDEDVKAFCSLLLAAGGETTDKA

IAGIFANLLTHPEQLAAVRADRSLIARAFAETLRYTPPVHMIMRHSATDVELSGGTVPAG

STVTCLIGSANRDEDRYRDPDVFDIFREDLTATNAFSAAADHLAFALGRHFCVGALLARS

EVETGVGQLLDAMPDLRLADGFDPVENGVFTRGPKSLPVLFTPVSG

>CYP157B27(2668574153)SGB

MSTSSPSFDPSFGSPLDPGTSAPSGCPVAPGAVRLSGSSYQQTPTELYRSLRREHGAVAP

VLLDGDVPAWLVLGYAELSYVTTHDELFARDSRRWNQWDSIPPDWPLLPFVGYQPSVLFT

EGDEHRRRAGVITEALEGVDQFELARDCRRIAERLIADFAGSGRTELMSSYVHALPMRVV

VQMCGMPVSGSDTQQLVDDLRISLDAGEGDDPVAAYGRVGDRLRQLVKDKRAVPGPDITS

RMVTHGAGLTDEEIVQDLISVIAAAQQPTSNWICNTLRLLLTDERFAVNVSGGRLSVGEA

LNEVLWLDTPTQNFIGRWAVRDTQLGGRHIRAGDCLVLGIAAANTDPEIWPEAYVGAENS

AHLSFSGGEHRCPYPAPLLADVMARTAVETLLEQLPDLMLAVEPAELTWRPSIWMRGLSA

LPVQFSPMAQ

>CYP107U21(2668574197)SGB

VNDTPACPHSATTPEGAAPAPELFTWEFATDPYPAYAWLREHRPVHRTALPSGVEAWLVT

RYGDARQALADARLSKNPANHAESPHAKGKTGIPGERKAELMTHLLNIDPPDHTRLRRLV

SKAFTPRRVAEFAPRVQELTDQLIDGFIEEGKADLIHDFAFPLPIYAICDLLGVPREDQD

DFRDWAGMMIRHGGGPRGGVARSVKKMRGYLAELIHRKRENPGDDLISGLIRASDHGEHL

TENEAAAMAFILLFAGFETTVNLIGNGTYALLRHPEQRAALQASLHAGESGLLATGIEEL

LRFDGPVEMATWRYATEPLTLGGEEIAAGDPVLVVLAAADRDPERFTDPDVLDLARSDNQ

HLGYGHGIHYCLGAPLARLEGQTALATLLKRLPDLRLAEEATDLRWRGGLIMRGLRTLPV

AFEPGIRSEESDRLSTL

>CYP125A19_ortholog(2668574983)SGB

MRCPHLPDGFDFTDPDLLQDRVPHPEFALMRETAPVWWCTQPANISGFGDEGYWVVTRHA

DVKYVSTHPELFSSNTNTAVIRFNETISRDQIDVQKLIMLNMDPPEHTRVRQIVQRGFTP

RAVRSLEAALRSRARSIVETALASADADGSFDFVTNIAVELPLQAIAELIGVPQDDRSKI

FDWSNKMAAYDDPEYAITEEVGAEAAMEIVAYSMNLAAARKECPAQDIVTQLVAAEGEGN

LSSDEFGFFVILLAVAGNETTRNAISHGMHAFLTHPEQWELYKRERPKTTAEEIVRWATP

VVSFQRTATQDVELGGQRIRKGERVGLFYSSANNDPEVFDAPEAFDITRDPNPHLGFGGG

GPHFCLGKSLAVMEIDLIFNAVADVLPDLRLLEDPRRLRSAWLNGIKQLQVTTAAEA

>CYP107F14(2668576517)SGB

VENTSVHHKESVRTCPFDYAQRLEFDPQLRQLLTEEPVSRIRMAYGEGEAWLVTRYEDVR

TVTTDRRFSRSAVLGRDFPRMTPEPIVQAESINLMDPPASSRLRSLVAKSFTPRRVEQMR

GGTQRVVDRLLDEMTEEGAPADFVARVSSPLPLITICEALDIPEADRPWLRAHAMTMMNV

GAAGKQDAVRAKAELRGYFQELTADRRRSPGEDLISTLATARDGAELLDDDELAVMAMVL

LITGQDTTTYQLGNIAYTLLTRPELLHSLQAEPARLPRTLEELLRHIPFRKGVGIPRIAL

EDVELGGVLIKAGDVVHVSYLTANRDAAKFDRPDELDPDRPSIPHMTFGWGAHHCLGAPL

ATMELEVAFSTLLTRFPALRLDVPPEDIEWNTTSIWRYPLALPVTW

>CYP105D30(2668577006)SGB

MTESTTEPARQDRAFTGTGTATESTPAPPFPQDRECPYHPPTGYEPLRAQGPLSRVTLFD

GRPVWAVTGHALARRLLADPRLSTDRTHPGFPIPAERFTQTRQRRVALLGVDDPEHNAQR

RKLIPSFSVKRIAALRPQIQETVDGLLDAMERQGPPSELVADFALPVPSMVICALLGVPY

ADHAFFEGCSRRLLRGPAASDVDGARIELEDYLGALIDRKRAEPGDGLLDELIHRDHPDG

PVGREDLVSFALILLVAGHETTANMISLGTFTLLRHPGQLAALRSGETTTAAVVEELLRF

LSIAEGLQRLAAEDIEVDGTTIRKGEGVFFSTSLINRDADVFEHPETLDWDRSARHHLAF

GFGVHQCLGQNLARTELDIALRTLFERLPALRLAVPADEIRHKPGDTIQGLLELPVAW

>CYP124G14(2668577225)SGB

MTVPYQPSGDRVLRAADVDLADPAFWRLPRPERLGAFALLRELEAPVLFTPRPGTARTAG

KPFYALVRHADVRTASRTPGVFASAPGVTTPEPAGWAKALFGNSMVNMDGSEHAALRRII

SRRFTPRLLAGVEENVGRLAGRLVDELIAERPRDFVPSAASRLPLEVICDLMGIPKAYRA

RISEQIDQASEHVGVERRGRARIRIPGRGLASLARMQWGMGRLAAERRRRPGDDLVSALV

CADIDGEALSGRQLGAFFSLLLVAGVETTRNAIAHGLFLLDRNPEQRELLRSDFDRYIGG

AVDEIVRHSTPIIQFRRTVTEECALGGRTFLPGEKVALIYASANRDEAVFTHPDRFDITR

SPNPHLGYGGGGPHHCLGAHLARLEMTALFRELIDRRPVMRDLGDPGLVDSNFDNRVGSL

PFTFGPTFT

>CYP107BX10(2668577428)SGB

LNPTDPTEPAPEPMHRLHFDPPGPPCRTELPGGTPAWLVNRYAEVRQVLSDPRFGRAQLY

APDAPALSDVPDLVNDPDLMFNQDGPGHLRLRRTLRRAFTPRAVARWRPWIAAIVEEILD

RLEERPQPADAVAEFALPLPVAVISCLMGLDESAWGRLRHWSEHAFSDGTHESEQVASAL

AEFSAFGADLLAERRRAPGEDLVSGIVAAADEEGGVPEAQLVSLVCGLVVGGHDSTMTML

GNALLYLLGERRETWPRLGADEEAAGRVADRLVHLVPLGDDRGSTRHAATDVEVGGVTIP

AGAIVLADCGRANRDPEVFPHATLHDLFAPLEAPTLSFGAGPHYCLGAWLARTELQLALH

RLAARFPGLRLADPTDAVVWRTGTTSRSPRSLPVRW

>CYP157C34(2668577614)SGB

VTTPFHHEPGAVPPPQCPAHNLDIGPGGLRRLHGPEAENNPAGLYDKLRAEHGTVAPVLL

HGDVPAWLVLGHSENLHLTRTPSQFSRDSRRWRALQDGSVAPDHPLAPIFTWQPVCVFAD

GAKHERQRGAVTDSMERIDTRGVRRHINRFSNRLVNDFCEKGTADLVSQFAEHLPMMVMC

AIFGMPEEYDERLVQAARDMTRGTETAVASNAHIVGVLTRLVERRRAEPAPDFASWLVEH

PATMTDIEVVEHLRLILIAAYESTANLIANVLRMVLIDPRFRARLSGGHMTVPEAVEQTL

WDEPPFTAVFGRWAVGDTELGGQQIKAGDALLVGIAPANTDPTVRPDLTADMGGNRAHLA

FSGGPHECPGQDIGRAIADVGVDALLMRLPDLELGVGESELRWVGNIMSRHLVELPVTFA

PSPQQKLDADPLSVMARTPRPADAWEISSPARTVPEPRHEEAAGAQSAHAPAAVPAAAQP

VPAAPAPGTAPVATIPRQRRPAAPARLWQAVTRWWNGY

>CYP1047A1(2668577632)SGB

MSTQTGPALDTQPRGHAFTPGPKGLPLVGNLPQFGKNPLAFFELLRGHGDMVRWRFGRNR

CVFLSDPDCIGELLTETEHTFDQPKLGIAFRTVLGNGMLVARGRDWRRKRSLVQPSVRPK

QVRSYAATMADSAVELADRWSDGQRIDVKQEMAALTQKIAVRTIFGVDTPADSEAMGRAM

DVAQMEIGKEFAGIGALLPDWVPTPGRVRIRKAAAVIDAEVGRVVARHRDGEEERPDLLS

RLLTAVDESGTHLTDDEIRDETVTLYIGGHETTSTTLVWAWYLLGRNPRVRDALAEELDR

VLGEREPGFDDYARLTYTQAVVKETLRLYPAVWLITGVAKEGAVIGGLPIEEGTRVWASQ

WSAQRDARWFPEPEEFRPERWDAEGGDEIAEYAWFPFGGGPRVCIGTRFAMVEAVLLLAV

LARRFTLDVDQSEVTPVTGLTLQPDRDVTATVRAR

>CYP154C3_ortholog(2668577639)SGB

VNCPHAEAARAGRNAGVMTIDPMVQDLDGETVRLRDAGVLARIDLLGVPAWTVTRHAEAR

QLLVDPRLVKDIDAWGLWQSGVVTRAWPLIGMIDAGRSMFTVDGAEHRRLRTKTSQALTP

RRLEAIRPDIEKFTDELLDALDAARGEDGVVDLKAVFAQPLPMKVVGMLMGVDESQHAML

TRQYKAFFSMLTPQDERLALLAELDVFYTDLVREKTARPTDDLTSALILAEEGGEPLTEE

EVVGNLKAMVAAGHETTIGLVLNAVRALLSHPDQLRKVLDGEIGWDAVVEETLRWDTPTT

HLLMRFATEDIAVGDEVISKGEGVVISYRAIGRDFEQHGPDADAFDITRPTRNRHMTFGH

GPHICPGAALSRVEAGIALPALFARFPGLRLAVPDEEITRLPVMTQNDMAALPVLLG

>CYP157A28(2668577640)SGB

MTNPSSATPASPTGTGGGCPVGAGTGAVALGGPGFDAEPHELYRSMRREHGPVVPVELPG

GFPAWLVIGYRQLHQVTSDGELYPRDVSLWNQWGNIPADWPLLPMVGTPMPSIYFTAGAE

HRRHVDMVVPALEGADPFEIRQHCEQLADRLIDAVCSRGTADLVAEFAEPLPVLVLARLV

GFPDDEGADIARVLKDLADGGPDAQKAHLRFGDHMQRLVADKRARPGDDVTSRMLAHPGP

FTDEEYALDLMAITAAGHLTTADWISNSTRLMLTEDQFADALSGGRHSVAEAMNEVLWED

GPTQILAGRWAARDTRLGGRSIARGDMLLLGLGAANADPHIRQQVTASAVRSGQGGNSAH

LAFSHGEYRCPFPAQEIAEIIARTGIEVLLDRLPDLQLAVPATDLVRRPSAFLRGTTALP

VRFAPVRTTGDAL

>CYP1373A2(2668577685)SGB

MTQSAASPTSRVPRLPGRALIGNTWEFKRDRLALFSRTFAECGDLGVYTIAGQDLYLANS

VELVHEILIRNGGLFEKTDRFRSFARPLLGDGLLTATNEQHRPNRRMIQPRFHSAAVKTS

ADIAARVTRTVADRWSDGAVVDVRREMVAVTLGIVGQNLFSRDVLGEADELGNALTDAIH

GFDSQASALIPLTIEWPTPANLRYRRAIERLERTFYDLLAERRAAVRRPDDWLNLLMEST

YEDGTPIGDRQIRDEALNMFMPGHETTATALSWSFHLLAEHPEVYDRLLAEVDEVLGGRR

PTLEDLPRLPYALQVFKETMRLYPPVYMFTRQATVDVEVLGHVLPAGAAVVFSPYALHRR

ADYFHDPERFDPERFSPEREAGLPRHAYLPFGAGHRVCIGNHHALLNGHIALVTLAQQVR

LSSVPGASPVMEPMVTLRPRGSLPMRVERRSRPAAHDGPPTTWERPAHGEAPAACPYADN

S

>CYP107L48(2668577909)SGB

VLTTEPLVDLAALGEQFTRDPYPAYAALRAQGPVHRVRIPEGADAWLVVGYEAGRALLAD

QRLSKHWSRASPTLGVSKVSAGSSMLGSDAPDHTRLRKLVAREFTPRRMEELAPRIQEMT

DDLLDAMLAAPDRSADLVEALSFPLPMSVICELLGVPFLDREDFRTWSGQAVSSIDPSVR

ASSTQAMTAYIAGLLADKREKPGEDLLSALIHTADEGGDRLSGEELIGMAWLLLVAGHET

TVNLITNGVHNLLAHPGQLAALRADFSLIDNAVEEILRFEGPVETPTYRFTTEPIEVGGT

VIPGGGELVLVAMSDANRDPDRYPGGDRFDITRDARGHIAFGHGIHYCLGAPLARIEARI

AIRSLLERCPELRLTADPATLTWRAGMLMRGPLSLPVAW

>CYP107L49(2668577910)SGB

MAELDLRDLPDFTADPYPYYAELRAEGPVHTVRTEHLERLWLIVGYEEGRAALADQRLGK

DWRPGGGWKASEAQLNANMLELDAPHHTRLRRLVVREFTARRIEALRPRVTEVTDRLLDA

MVPQGSADLVDALAFPLPMTVICELLGVPDIDRDAFRVLSNGIVTPTPEQRDADPVGAMS

AYLVDLIADKRRSPGEDLLSALIRATDEDGDSLSSAELVGMAFLLLVAGHETTVNLIANG

VRALLDHPEQLALLRTDPGLVDGAVEEMLRYDGPVENATFRFAREPVTVGSTVIPRDAVV

LVALASADRDPDRFPAPDTFDIRREPQGHLAFGHGAHYCLGAPLARMEARIAIGALLERC

PGLARDESGGELDWLPGLLMRGTRGLPVRW

>CYP159A7P(2668578054)SGB

MPVDRPDALPAARRPHRLSGGLPGLLAPGATTDPYRLRLYRLLRTDYPLGYDPALGAWLL

SRYGDVALALTDPRFTGYPHDGAPRGRAPVPLGLCRGSLVCGPLTVPWSTAAPAVERTAY

VLARRIAGRDRADLVADFCRWLPAGAAAAAAGLAHPDLSALHRGARHRRALTGAGDCTGS

TALREHALASFLANMLDDPDLLAAATAGAARGDGSGSLIGRAWAETLRRDPPVQIVLRRT

RTEVAVSGGTLPADAPVACLIGAAGRDPARFAAPDRFDPLRADGDPLLIGPAGCPAALLG

RLEAEHGLRALLTAMPGIRWADGFRPVAGGLLTRGPRALLVRPS

>CYP157A29(2668579666)SGB

VTTVSGCPVTHTSVPLSGPRFQSDPVQLYRDLRRDHGAIAPVVLDGDVPAWLVLGYRELH

QVTGDPVLFSRDSELWNQWDRIPDDWPLLPMIGRRQPSILYTVGERHSVRAMMISNALEG

VDPFSLKRYAEEFADELIDRFCTKGSVDIIAEYAKLLPALVLARIYGFSDEEAHPLVGAI

NDMIDGRERALAGQQHLGASMFQLLADKHAEPGDDVATRMLADPGGFTDEEVAQDLMVMM

AAGHQPTADWMGNSLRLMLTDDRFAASLSGGRHSVAEAMNEVLWEDTPTQNVAGRWAARD

THIGGRHIRAGDLLLLGLAAANGDPQVRTDGSALTGGNNAFLSFGHGEHRCPFPAQETAE

VIARTGIEVLLDRLPDVDLAVPAEQLTRRPSPWLRGLTDLPVLFTPTPALGRPGSHGGPA

>CYP154C4_ortholog(2668579667)SGB

MTRIALDPFVRDLDGESAALRAAGPLAEVELPGGVHVYAVTRHAEARALLTDSRVVKDIN

VWGAWQRGEIPMDWPLIGLANPGRSMLTVDGAEHRRLRTLVAQALTVKRVERLRAGIEAL

TNASLEKLAALPAGEPVDLKAEFAYPLPMNVISELMGVDAADHPRLKELFEKFFSTQTPP

EEVPQMMADLGTLFTKIVDGKRANPGDDLTSALIAASENGDHLTDEEIVNTLQLIIAAGH

ETTISLIVNVVEALQTHPEQRKKVLNGEIGWDGVIEETLRWNTPTSHVLIRFATEDIEVG

DKVLPKGEGLIISFGALGRDEESYGPTAGEFDATRTPNRHIAFGHGPHVCPGAALSRLEA

GIALPALYERFPELDLAVPASDLRNKPIVTQNDLHELPVELGCPFGGDA

>CYP107P7_ortholog(2668579980)SGB

MHVSFDPWSPAFVADPYPAYTALRAAGRAHWFEPTGQWLIPHHSDVSALLRDRRLGRTYL

HRFSHEEFGRTPPPPEHEPFTTLNGQGILDLEAPDHPRIRRLISKAFTPRTVENLAPTVR

RLAAELVDAFVAKGGGDLLAEVAEPLPVAVIAEMLGVPEADRGLLRPWSAAICGMFELNP

SAETAAAAVAASVDFSTYLRGLITERRADPGDDLISALIAAHDEGERLTEQEMISTCVLL

LNAGHEATVNTTVNGWRTLFHHPEQLAALRADPALLPTAIEELLRYDTPLQMFERWVLDD

IEIDGQVIGRGAEVALLFGSANRDPERFARPDVLDLSRTDNPHITFGAGIHFCLGAPLAR

LELAASFGELLRKAPALRMTTEPEWQPGYVIRGLKELRAEV

>CYP156B5_ortholog(2668580071)SGB

MDPQPGATPYSAPAGCPMHQQQTSLYGPEFAADPHRFYDAARTHGPAAPIELAPGVDATL

IVQHEAALRVLQNPALFARDSRRWAALREGAVPMDSPVLPMMIYRPNCLFTDGAEHLRLR

KAVTESLARLNSSRLSRDVERIADYLIDQFIERGTADLLNEYAKLLPLLLFNQLFGCPGD

IGDRLTRSMSAIFDGEDVLRANAELTECLMELVSIKRRQPGDDITSWLIQHPAGLRDEEL

KDQLVMLMGAGVEPERNLIGNALLLMLAGEQPGAPERRGSGMLVEDALDDVLWNNPPIAN

YATHYPVRDIELDGVVLKAETPVLISFAAANSDPGLTDARQTLSKGAHLAWGAGPHVCPA

KSPATLIALTAIEKILNTVPDLALAVPASGVAWRPGPFHRALVALPVRFTPTAARRTAAG

VQPPAQTSAQLSDPYRSATSAAPVTPRHAAGPAKKQKGWWSSFLDVFRV

>CYP1035A13(2668580072)SGB

VSITGGTGRNPDGRRAVIGLLRRLNSPEGQAEPYGILAELRTMGDVVRAPWNGYFVTGFD

TCSQVLRGRDWLAPDLAWQERQDDSKQWDAIATREMTTTLARLNAPEHTCQRRSLGNLFD

RSTIERLAPDVERDADRLLDELAEKLRWGEADFVSTVSEQLPISTVGSWLGIPPEDHPHI

LEITHNQVFAQELLPTKSQLAVSAEATLALRAYFTELVARRRAEPRHDVLTGWIHTWDAM

EPDREKADEILYRLTMFVTIASLETTATLLTSMTHLLTEPTRWAWLRQYPEHIDAAVDEV

LRYDPPIHINTRIAAEDTVLAGVPVKKDSMIHVLYGAANHDPRRNPDPDGFDILRGGSHL

TFGGGVHYCLGAALAKLEARTLLARMLDRFPTLRTAGPPEYATRMVFRRITSLGVAL

>CYP107X15(2685032828)SGS

MDLADGLLDHPYDVYRRLRDTAPVHRITGPDGTPAWLVTRYEDVKAALADPRLSLDKRHA

TPGTYKGFSLPPALDVNLLNMDPPDHTRIRRLVGRAFTPRRVQQLRAPVRRTADQLLDAL

GPHGTTDLVASYAAPLPITVICDLLGIPEGDRLDFRQWTDGLTAPDLARPQAAKEAMAAM

LGFLTRLLETKRQAPADDLLSDLIAVRDEGDRLGEDELMSLAFLILFAGYENTVQLIGNA

VLALLQHPDQLAALREDPDRLPGAVEEFARYEGPMLLAMRRFPVEDVTIAGVTVPAGETV

WVSLSAADRDPARFPDPDRLDLSRDASGHLALGHGIHYCLGAPLARMETEIALAALLERF

PELALAEDEVNWRPSLRARGLRKLPVRYRSAQDGDRLRNHDAHDD

>CYP125A57(2685033236)SGS

MHCPALPDGFDFTDPDLLHHRVPLPEFAELRRAEPVRWIAQPHGLAGFADDGYWALTRHA

DVKYASTHPELFSSYLNTAIIRFNEHVQRDAIDAQRFILLNMDPPEHTRVRQIVQRGFTP

RSIRALEERLRARAEAIVTAARARSGPFDFVTEVACELPLQAIAELIGVPQEDRSKIFDW

SNRMIAYDDPEYAITEEVGAQSAAEIIAYAMNMAAERKRCPARDIVTTLVAAEDEGNLNS

DEFGFFVLMLAVAGNETTRNAITHGMHAFLTHPEQWELFKRERPQTTAEEIVRWATPVNA

FQRTATQDTELGGARIRKGDRVGLFYASANHDPEVFASPDAFDITRDPNPHLGFGGGGPH

YCLGKSLAILEIDLIFNAIADAMPSLRLVGDPRRLRSAWINGVKELQVTTA

>CYP147F22(2685033563)SGS

MTPDTISARITDYANRADPYPLYAELRKTPVLREDDGTYLVSTYYEVRNLANDPRLSNDT

RNRPSGYSRTADAEDTGLPPSFIFTDPPVHDRLRETINRPFGPPHSPRFLDGLRGELAKV

VTELLDAFEGKDQVDIVEDFAYPLPVTAICKVLGVPREDEPRFHGWADALASSLDPQAGE

EGQEKAQRARQELGAYLSDLIETKRRRPGPGILSTLAPDMTPADLEATAVLLLVAGHETT

VNAITNTTLTLLRHPDVLDRFQKDPDLAVPLIEEVLRYEPPVQFVPWTTALADIDVAGTT

IPKGSPVWLMLAAANRDPKRFMDPDRFDPHRKDNEHLGFYTGIHYCFGAPLARIELHLAV

PELFRRVTFSRLLEDPPPYRANAVLRGPRHLPVAIEGLTA

>CYP147F23(2685033580)SGS

MTSADDWQAFKRDGRIVVVGASLAGLRAAEALRDEGFTGSLTMIGDELGEPYDRPPLSKQ

VLTEWVPAGGTALPRRRGIDAEWLLGVPATGLDLATNHVRLADGREVPFDRVLISTGVRA

RPWFVESEAALDGVFVVRTREHAESLQRALAAGPSRVLVIGAGFTGSEIASICRERDIPV

TVAELAPAPLVGALGAVIGEVAADMQRAHGVDLRCGVNVTRLEGDAQGRFRRAHFDDGST

IDADVAVVALGSIRNTEWLRNSGLAAGVWGITCDTGCRALDLNGRVTDDVFAAGDVARCP

NPIYEYRLISLEHWANAVEQAEVAAHNMVSAQADHWHHLSIPLFWSIQFGVNIKSVGVPT

FADEVAVTQGSLDDRRFVTAYGYRGRVTAAVSFNNAKWLYHYRRLIETAAPFPPPCPTPD

QPADMKPVPVDLPGPTLLAQGATVVVTGHDPGERRVTAVHQHRQEEGRVTTTETPGTLQR

IFDYSARADPYPLYAELRETPVARQEDGSYVISTYHEITDILNDPHLSSDVRNLSRPMPS

VEGGATSSFIHMDPPEHDRLRRMATRHFGPPHTPGLVTGMEDDLTATVGSLIDGFAGKEQ

IDVVDDFAFPLPVTVICHLLGVPREDEPRFHLWVNDIMNSIDYNPKTDPKEKLDKGVQAR

KDLRQCLGRLVEQRHGRPGDGLLGRLANDDGPDGRMADDDIVATAKLLLIAGHETIVNLV

TNGMLTLLRHPQVLQRLRDEPDLVVPLVEELLRYEPPVQIIPWRAAYSDITVGDTVIPKG

SQIMLMLASGSRDPKRFHDPDRFDPDRRDNQHLGFGSGIHLCFGGPLARLETQIALTELV

RRLDRPRLVADPPPYRPSPVLRGPIHLDIEQGDG

>CYP107U22(2685034157)SGS

VTEQLHPPTAPDLFTWEFATDPYPAYAWLREYAPVHRTRLPSGVEAWLVTRYADAKQALA

DQRLSKNPAHHDEPAHAKGKTGIPGERKAELMTHLLNIDPPDHTRLRRLVSKAFTPRRVA

EFAPRVQELTDRLIDGFAKTGSADLIHEFAFPLPIYAICDLLGVPREDQDDFRDWAGMMI

RHGGGPRGGVARSVKKMRGYLAELIHRKREALPAEPTPGEDLISGLIRASDHGEHLTENE

AAAMAFILLFAGFETTVNLIGNGTYALLTHPEQRHRLQTSLAAGEKDLLETGVEELLRYD

GPVELATWRFATRPLTIGGQDIAAGDPVLVVLAAADRDPERFADPDVLDLSRRDNQHLGY

GHGIHYCLGAPLARLEGQAALATLLTRLPDLRLAADPAELRWRGGLIMRGLRTLPVEFTP

AP

>CYP113V2(2685035241)SGS

MAGFPALTELDGAGWSGLLRWLADARRDAPVLELAGMHHVFRAEDVRTVLSDSELFSSDR

TRMMPPTAQLGRGNLTMMDPPDHTRMRRIVNQAFTPASVGGLAPAIDEIAGELVSGLTPE

RFDLVRDLAYPLPIRVISRFLGLPEEEHGRFRTWSVGFSRGDAAQMDAMHRYLVEVAAAK

RARPVRDLMSRLATAEVDGAPATVDEIASLSGLILLAGHVTTTSLIAAAVRELCVRPELA

AEVRDHDRIEDLVLEALRTRPAFAQVTRIAAGDATLSGVTVPAGALVSAWILSANHDPLL

NPDPERFLLDRPARRHLSFGHGVHYCLGGPLAQLEAVAAVRAVVRRFARLTVLAPVEFHP

LPTLSIRRLVLAGQAYGA

>CYP2027A2(2685035264)SGS

MTTAPVRLPEHLDHPFSPAGRINPHPAYRWLRTNDPVHHDAASGLWLVTGHADCQTVLRD

PRFSAAKGQRERARDDALPPSMLTTDPPDHHRLRAPGALLLGPSAVRSVAGDLTRDVDEL

LDRLGRRPAAERPDAVADIGRPLATAVFGRLFGLAAAEYARFETLARAASINLDPMVPPA

RAALGRTAAAALTRYLDAHCATLARGGRAAPLAAFAADDRLTRSEMLGVLNLAVVGGWQP

LAELVGNALCSLLPDRAAVRRLAAGDDTSGEGGDAGGEAARRAVDELLRLEAPIPFTARV

TTDTVELAGGTLPAGARVLAVISAANRDPAVFADPDELRVDRHPNPHLAFGGGPHFCLAA

QLVRQAGGLLLPRLLRRFPGMRGPHGVPDWDRCLVPRRLRVFPVDLGVPGADDKDPGTAE

EDHG

>CYP1385A2(2685035268)SGS

MAVMRESPLAFITELARTYGGITRHRADGETVYLLNTPEYARHVLKDNGANYTKEGTPDD

AMLRPLLGNGLLTSNGDDWARQRQLTAPAFRPSSVRTFDGIVTDATSGMLERWRPAIETG

TPLAVDDHLTALTLGILTRAILGADLDGIGEGFGRAVDAVNRCIGHYVPDPDPDPADTAR

RFAGFSRARAFLDMVTRTLIASRRAAGPSAPGSGPGHAGNLLDTMMSAGHLVSDEDLRDQ

VLTVVMAGHETTAKSLTWTLHLLDRHPHEAAKVREEVDRVLGGRPARAEDLADLPVCQRA

VKEAMRLYPPVWLISRRAVGADVIGGYHVEPGTLVCVSQWVLHRHPEYWDAPDAYRPDRF

DGASLPSHLYLPFGGGDRICVGQHFAMLEAVLVLATLTQSVRLETVEGFEVEPEALVTLR

PKHGMTMIARPR

>CYP107P2_ortholog(2685035364)SGS

MAGPTDLAFDPWDPAFLADPYPAYAELRARGRVIWYEPTRQWLVPHHADVSALLRDRRLG

RTYQHRFSHEEFGRSAPPPEHEPFHTLNDHGMLDLEPPDHTRIRRLVSKAFTPRTVERLG

PYVADLAGRLVDRLVAAGGGDLLTDVAEPLPVAVIAEMLGIPEPDRAPLRPWSAEICGMY

ELNPSQETARRAVRASVEFSDYLRELIAERRKEPGDDLISGLIAAHDEGDRLTEQEMIST

AVLLLNAGHEATVNATVNGWYALFRNPGQLAALRGDHSLIPSAVEELMRYDTPLQLFERW

VLDDIEIDGTVIPRGAEIAMLFGSANHDPEVFHHPGELDLAREENPHISFSAGIHYCIGA

PLARIELAASMTALLERAPTLRPAEEPERKPNFVIRGLEGLRVEVR

>CYP170A10(2685036377)SGS

MTVESVKPVTAETAEPREAPLAGGAVPVLGHGLQLVRDPLAFMSGLRRHGDVVRLKLGPK

TVYAVTAPALTGALALSPDYKIDGPLWESLEGLLGKEGVATANGPRHRRQRRTIQPAFRL

DAIPGYGPVMEDEAHALTERWQPGRTIDCTSESFRIAVRIAARCLLRGDFMDERAERLSI

DLATVFRGMYRRMVVPLGPLYRLPLPGNREFNRALADLHLLVDEIIAERRASGQKPDDLL

TALLEAKDDNGDPIGEQEIHDQVVAIVTPGSETVASTIMWLLQVLAEHPEHAEKVRAEVE

SVTGGRPVGFADVRALRHTNNVVVEAMRLRPAVWILTRRAVTDTTLGGYRIPAGADIVYS

PYAIQRDGRSYDRHLDFDPDRWLPERAKDVPKYAMSPFSVGNRKCPSDHFSMAQLSLITA

AVSARYRFEQVSGSNDTTRVGITLRPHRLLLRPVRW

>CYP102D2(2685037184)SGS

MTTHSANGLRPIRSPRGIPLLGYTPQIPDTNPVEYFNELSKQFPEGIYGMDIAGIEQVFV

YDPDLVAEVCDETRFFKQIEKTPLSHVRDYTGAGLFTAHQHEEEWAMAHRILLPAFSQRA

MKTYYGQMLEIAQNLVGKWESREGQPVAITDDYTRLTLDTIALSGFGYRFQSFDKEELHP

FLNSLLEALIESMRRSQELPMMTRLRKADDRKYRENIQLMRDLVESVIKERREGKGTGEE

DLLGLMLEATDPETGELLADENVRDQVVTFLIAGHETTSGLLSFATYSLMRNPHVLAQAY

AEVDRLLPGDTVPDYDTIMQLDVIPRILEETLRLWAPIPLIAKAPIDDTVIGDRYELKKG

TRANILMGALHSHPKAWDRPEEFDIDRWLPENRAEQHPHAYKPFGNGVRACIGRQFALTE

ARLALALVLQKFRFSDTTDYKMDVREALTRKPGDFQLVVRRRQEHQRTVFGAADLRTGDT

QSQAAVSGVGVNLTVAYGSSLGSCEDLARTIADRGERSGFGTTLMSLDELGDNLPTEGLL

VVVAASYNGKAPDNAQRFDDLLATGLPQGSLANVRFALLGAGNTQWVATYQAFPKRIEEG

LLAAGATPVVERGIADAAGDFDGMATRWMDILWATLAEEYAADTSQASGPRYQVQLLTES

DVRPAIVSEQAYPLTVVANEELVADATGLWDFAIEPPRPSAKSITVELPENVTYDTGNHL

AVFAKNEAALVERALRRLGVDYGQVLRLDQPGGGRTHLPVGTPVTAGILLTEFLELQDVA

TRSQIQTLAEYTQCPWTRPQLQAYTADTREAEEHYQKEILGKRISVLGLLERFEAIELPL

AVFLEMMGPIRPRFYSISSAPSANPRHVRLTVGLLEGPAMSGDGQYRGTCSSYIAGLEPG

DVVYGYVRVPSPTFAPPADPATPLILIGPGTGIAPLRGFLEERATQHANGTQVGLSQVFV

GCRHPEHDYFYKQEMQDWEQTGIAQVHTAYSAVTGHPARFVQNAIANAADTVWQAIEDGA

YIYVCGDGRRMAPAVREALAAIHCQRTGSDDETAQQWLAQLEADERYQQDVFA

>CYP147C2(2685037194)SGS

VVEETPWQQALRYANRANPYPFYEELRRTPVARQPDGTYVVSTYQEIVALLHDPRVSSDV

RKLPVPVAAPAEGSAEAEPITEAVISLEPNIITQDPPEHDRDRRMMTPRFVGPPHSPHLI

SDLEPEIRRIVDGLLDNMQGKTRFDAVDEFAYPLPVTVICKVLGVPLEDEPRFHSWIETA

LDALDFGPEAASEEMQSRLAGGRRAVQEFGQFAADLLDRYARQPGPGMLSAMVNEDGPEE

RMSQGVLASNALLLIFAGHETTVNLIAHSVLTLLRHPDALEKLRRRPELIVPGVEELLRF

ESSVQFWHTRSAVEDIDIAGTTIPKGAPIFLAYGSANRDPQRFTDPDELDLERCDNQHLG

FSQGIHFCFGAPLARLEVQVAVGEFVRRVQNPRLVEDPPPYRHNQIFRGPRHVLVDIDGI

HD

>CYP247A3_ortholog(2685037248)SGS

VRLTPGPARDIDLDRVDLFDLDLYTSGDPHPIWDAMRAKAPLHHQVLPDGREFWSVTRYD

DVCRVLGDHREFTSERGTVPTHLGTDDVAAGVLLTSTDPPRHTEVRRPLGAKLTARAVKS

WEDSIRRSIVRFLEPALDGGTFDLAEKALLLPAMVTGPLLGIPEKDWEELVQLTAMVTAP

SDQHFQQGSEAATLAIAHHELVSYVKEWVKTRRAAGGEDDSLLHHLMSVRPGGAPLTDEE

IALDGYSILLGANVTTPHTVSGTVQALIERPEQFEKAQADPSLIPNLVEEGLRWTSAACN

FMRYAVDDVQIGAGTIPARGAVVAWIGSANRDESQFPDPHVFDITRDGAKRQVAFGFGSH

FCIGAPLARLTLRVFFEELLQRFGSVELDGEPQHLRSYFIAGMTHLPIVAQKRKTP

>CYP105BC2(2685037348)SGS

MADSCEPLPAWPMPRSCPYAPPDAYEGLRKDPPLKVRIRGGEAWLVTRHADVRQVLNDNR

FSSDDQQPGFPIRIQLPPEPGVMSFSRMDDPEHGRLRRMAMTEFTARRTRALRPEVELLV

EQLLDELAAGPNPVDLVKSFALRLPSLVIARMLGVPEDDEADFAEQSQVILSQDASPEET

YGAFVEMSQFLDRLAAQRTADPQDDLISRLATRYVAAGELTHQELVAMARFFLVAGHETT

AHQISLSVLSLLRDPDQLSELRADPALFKPAVEELLRYWSISQDNQVRAAAADVELGGAR

IRKGDGVIVAIPGANHDESVYPDPDRLDIRRNASGHLAFGFGAHLCPGASLARMELEVCL

SGLFARFPTLRLAVPSEDVRFRQNTIVYGLEDLPVTW

>CYP180A26(2685037461)SGS

VSVREAPPVPDVFDPRRYAAAVPYADYRVLRDHHPVAWQEEPEVLGWPAGPGFWAVTRHA

DVVRVLKESATYSSYVGATQIRDPDPEDLPFIRRMMLNQDPPHHGRLRRLVSRAFTPGRV

DRFAAVARERARTLLGLALDEARSGDGTADLVAAVTDEYALLNLADLLGVPESDRRLLLH

WTQRVIGYQDPDEAAPPVLDAAGKPVNPRSPAMLRDMFAYARQLAAHKRRCPADDILTTL

AHDAELADAELEMFFFLLTVAGNDTVRGAAPGGLLALAEHPEAYGRLRAGKAGVRPAVDE

LLRWHPPVLTFRRTAARDTELAGRRIRAGDKVVVFHASANRDERVFAEPDRLDLARTPNP

HVSFGDGPHVCLGAHFARLQLRLLYEEVLRALPGPRPAGPPGRLVSNFINGFKSLPLSIV

>CYP107F15(2685038606)SGS

MTSHDTTARSCPFDFADGLEFDPSLAALAQRGPVSRIRLPYGETEAWLVTSFAGVRQVTC

DPRFSRAAIVGRDYPRMTPEPIVSPESINVADPPHATRLRHVAAQAFTRERVAGMRPAVD

RVVAGLLTAMAEAGPPADLVTHLSVPLPHLTICELLAVPESDRDHLRTCTMRLLATSPDT

KQDAADAKAELRKYFADLIPDRRRAPGEDLLSAMAAAPVPEGEEPLSDDELAVLAVTLIL

SGNDTATCQISNIAYLLLTRPEERSALEREPARFPDALEELLRFIPFRKGVGIPRIALED

AEVEGTAIRAGDYVHVSYLAANRDPVIFPDPHTLDLERPAHPHMTFGWGGHHCLAAPLAR

AELDSAVTGLLTRFPHLRLDVDRDEIQWDTGTIRRFPIRLPVTW

>CYP107CJ12(2685038797)SGS

MTTLEPLPDPVPLTGCPYKADPYPLYERMREAGPVHRVLFPSGVQAWLVTGYDAAHAALN

DDRLGKNHDRGNDRWRARASIMPEPQHSQLQAHLLHQDPPVHTRMRRFVTDAFTPRRVER

LRPRFQELADAVVDALPESGPADLVAGFAARFPFLVLAEVIGLPQELAARFDRDWGKVVQ

PVGPTDPGRPLYEARLHGLQSYIADVVAHKRDHGDDDLLGRLVVARDRGELSQEELDSMI

FQLLVAGQEPVTNQITTALIALFRNPEQLARLREEPGLLPRAVEELFRYDSAFELTTWRF

FDKDSDLHGTGIPAGDSVIVSLCAANRDPERFPDPDTLDLDRTPNPHLAFGHGIHFCPGA

ALARAELQIALGTLLTRLPGLHLAVADEDIAWIPAVLGRGTNQLPAGYDRRL

>CYP154B7(2685038809)SGS

MENTACPYALDLMGQDHMGEAATLRAQGPAVPVELPGGVLAWAVVQQKYVERLLTDPRVS

RSARLHWPAFIEGKITEEWPLYPWVANENMLFAYGEHHTRLRRLVAGAFTARRSEALRPR

VEELSAELLGRFAALAPGTQVDLRTAYAEVLPLRVICELFGVPHGAETDALSAALSTVFS

STVPAQEMEAARLEAFGRLATLVKAKRERPGDDLTSSLIAARDHGDRLSEDELLGSLFMF

IAAGQDTTATLITNAAGALLTHPEQLAHVREGRAGWPDVIAETMRMHTPGAYAPMRFAVE

DIDLDGVRIRKGDCILVNFAAGGHETDRHGPDADRFDLLRTADRDVLGFGHGPHRCLGAP

LGEIEAASALSRLFELFPDARLACAPEELAPLPTFMLNGYRSLPVVLRPSAS

>CYP156B19(2685038831)SGS

MQQRSGIQGPPPGCPAHGNLPLYGPQFGSDPDGHYEHLRGLGPSAPVDIAPGVEVELVTN

YDAALHILQNPASFVRDSRRWKALNEGRIPEDSPALPMMGYRPNALFSDGAAHARLRQAV

TDSLATVNELQLVRQTQQSADYLISRFSSDRLGQAELMAEYAQPLPLLVFSDLFGCPPEI

GDRVIVGISGIFDGTAGADLVLAEALAELIALKRRRPGSDLTTRLMEHSAGLTDEETLHQ

LVTLLSGGTAPLAATIGTGSALILGDDWQAGLPVEDAVTQVLWNYAPIANYAAHYPTHDV

ELGGRILRANEPVVISFAGANTDPRLAEHREQLSAKAHLAFGAGPHACPAKDPAFIIAVT

AVETLLNRLPDIEVRVPFKDLSWVPAPWSRSLVALPVRFTSRTVTSTPAPAPQPQAAAAA

PHRVPSPAPQPAATASSKPKPGLFSRFLAWTRGE

>CYP1035A14(2685038832)SGS

VEIAITPPSDRRVTVSLFSRLRTAKGQANPFPIYAELRSRGGVSAAPWGGHLVTSFELCD

RVLRGRDWLEPDARWREQQVSGSRWTAPSSREMSRTLPALNAPEHTQVRRSAGTFDRGSL

EGMRGTVGRITGRLLDSLAERLHEGEADFNALVGEELPVAVIGHWLGLPTADFARLRELT

HDQVFTQELLPSASQLARSDTATAELRTYFMDLVRERRARPGEDPVSRWIAAWDGMESDR

DRADEAVYFLVLFVLLAALETTATLLSTMTLLLLEHPRQWDWLTVHPDLVPAAVEETLRY

DPPTHVISRVSAQDCVLGGVGIAKDEMVHLLVGAAHRDPARHKDPELFNLHRKPAHLAFS

GGVHYCLGAPLARMEAQTLLYQLVRRFPRLTLVRRPSWAPRVAFRRLLNLDVALS

>CYP121A4(2685038910)SGS

MTTAPDLRTYPALLDFPFSWDGTRPPAEVEDLRAATPVRRVRTIAGDEAWLVSSYELCRQ

VLKDDRFSLKDTSAPGVPRQYALTIPPEVVNNMGNITGAGLRRAVLKALNPKAPGLTEWL

RAEAHRLVDLMEADGPTTDLRAAFCEPYSAGMHCRILGIPQTEAPAFLRSLDIAFMNSPC

PITGARINWDRDIARMSGLLDDPATHGLMGELAALRDDPDYAHLTDEMLATVGVTMFGAG

VISTSGFLAMALVYLLTHPIAWDLLRDRPDLIGPGVEELLRVNLSIGDALPRLALEDVRL

GDVEVRAGELVLVLVEGANLDPEKFPDPHRFDVHRDNTADHLSFGGGAHYCPATALGRAH

ARIALEVLLDRLPELALAVPSGQLVWRTGFMKRIPERLPVTW

>CYP154U6(2685038926)SGS

VSDIARIPLDPFGTDIAAESARLRALGPVVAVELPGGAPAWAPTGYDVLRELILDPLVSK

DARRHWRLWPRLADHPTWSWIATWVGVANMLSAYGPDHTRLRRLVAPSFTHRRTEALRPR

VAAVTAELLDALEAEGADGRPVDLRAGLAHPLPIRVICELLGAPEELRPDAGRLIADIMD

TSDQSPERAASVQERIGTVLGALIAHRREDPGDDLTTELIRVRDEDGDRLCDEELLHTLL

LVVGAGFETTVNLIGNAVVALLTHPGQLAAVRSGQVGWEGVVEETLRVHPSIAALPLRFA

VADLKVGEVTIPAGDAIITTYACAGLDPARYGPDAAGFDAARGAGDHLAFGVGVHRCVGA

PLARMEALTALPALFARFPGLRLAVPADELRHVPSFIAHGWQEVPVLLGA

>CYP183X2(2685039142)SGS

MSHVVPEQERAWKVGTAPGIFPLVGHGIALYRRPLAFLNSLPAYGDLVEIRLGPQRAWMV

CHPELVHRMLKDTRTFDKGGPLFDRLRTLMGNGVVTCPHQDHRRQRRLLQPAFRPSRLAE

RADLLGEEAEAVCREWEAGRQVDISAAMMALTTRVISRVLFSDSLDTGTAAEVRQCLAAI

VHGLFVRTVVPIDALFRVPTPANRRYRRAVERTHAIIDAAIAERRRGDPRDDLLGTLLQA

THGDGAEPAVTAQEVHDQLVSLLLAGTESTALCLASAFSLLAQNPEAERRLHAEVDAVLP

GGRRPGPDDLPRLVHTRAVVTETLRHSPPGWLFTRVTTRETEFAGCRLPRGATVLYSPYL

LHHDPAWFPDPGRFLPDRWLPGPGAAGAHGALIPFATGSRTCIGEAFAMAEATVALATVA

GRWRLRHVPGPVEPPRPGATLGPRSLVMICESRARVPVGTALHTGSRAPGATQASRVQAS

RMSGDSGVDDA

>CYP154D19(2685039193)SGS

VEPTPATAPHRMDPSGGCPHAANARLLARGAVAPVLLPGGIEGMAVLGHDALRDFLQHPD

VAKDARHFRALREGRIGAGWPLLTFATVQGMTTADGDDHRRLRSLVSRAFTPRRVEELRP

RIEELTASLLDGLERAAEAGGGVADLREHFALPLPMSVISELLGVDAEFRDRLHHLSNQV

VATDIEPDEAVAANRELVAVLAAIAAAKAEGPGDDLTSALIAARDEDGDRLGGQELIGTL

VLMIIAGHETTLNLITNAVRALCGHRGQLELVRAGKASWADVVEETLRWDAPVSYFPFRY

PVRDLTVDGTLIPAGTPVLAGYSAAGRDPAAHGPDADRFDVTRPARADGVRHLSLGHGAH

YCLGAPLARLEATVALERLFARFPRLDLAVAEDELTRHSSFVGNSVRTLPVRLGDRAGRH

DAESAIPKP

>CYP1038A6(2685039265)SGS

MDTAAGPGSLHQVPGTGRPLPEAGLGLVERWRATGGELVELLSQVRERFGGIAAFRLGPA

PTVLVTDPQAVQHVLARQPELYVKRSHRARLLIGDGVLAATGTAWKRQRRLLQSQFTGTG

MRRYEQRITEAARTTAGRWARYARTGQTLDVGREMRRFALDAIWRSLTGHPLDDGTEREL

AAVAAVTAALPTLPADATEAQGAVATDLARIDAAARRAIDAARSGAAGPHGPGLLHILTE

AAAERPEYTDRLIRDELVTLLVAGHETTATTLTWLYLLLDRHPAARDEALAAGAEGSAQR

RQAIQALVHETLRLYPSAWILPRHATEDDTLAGYAIEAGTDVLVCPYLTHRDPELWPDPE

HFAPRRFITPDGRPTHPGAYFPFGIGPRACLGLQFALRESTVLLEHLLPVHTLAFSSTPT

KTAYGLTVRPDGPTTATLLAAGPDSRRGGVG

>CYP107BU4(2685039710)SGS

MVTARRFPLGSATTLTELEDDPHPRLALLRAHEPVSWVPVLGGWLVTRRDLALRVMRDAA

TFTVDDPRFSTARVVGPSMLSLEGAEHKRHRAPFTDPFRTQDVHDRFTGFVEQEAARLVE

GIRPRGSAELRREVAGPLAVAVVAESLGLVDADAEAVLSWYDAIVSAVSDITAGLSANPA

GAVAFDRLRASVEATVAAGSDGSLLVAASESMRLTLPEVVANAAVLMFGGIETTEGMIAN

AVLHLLAHPGQLALVHADPDLVDAAIEESLRLEPAAAVVDRYATADVTIGGADVRRGDLV

VVSLAGANRDPEVFQSPDSFDLRRGNSRLQLAFAHGPHFCPGAHLARLETRACLLALLDR

LPGLRLDPASSSAPRGLVFRKPPALRVRWDVPARR

>CYP158A21(2685039890)SGS

MSEETLTVSGQPHAETLSETLPPVRHWPALDLAGADFDPVLAELMREGPVTRIQLANGEG

WAWLVTRYDDVRMVANDPRFSREAVMGRQVTRLAPHFIPDRDAVGFLDPPDHTRLRRSVA

AAFTAKGVERVRHKARGMLDELVDELLQDGPPADLTATVLSPFPIAVICELMGVPAADRH

GVHTWTQLILSSSYGKEISERAKREMSAYFSDLIGLREGSTAEDVTSLLGAAVGRGEVTL

EEAVGLAVLLQIGGEAVTNNSGQMFYLLLTRPDLVERLRRAPGIRPRAIDELLRHIPHRN

AVGLSRIALEDVEIRGVRIRAGDPVYVSYLAANRDPDVFPDPETIDFSRSPNPHVAFGAG

PHYCPGGMLARLESELLVDALLDRVPGLRLAVPPNQVPFRKGALIRGPEALPVTW

>CYP147F24(2685039987)SGS

MTHASLLRQITDYANRANPYPLYEELRKTPVLHEEEDGPYVISSYWDIESLLHDPRISSD

QSNVTAAGEDELSGPEATGLPPSFIRLDPPEHDRLRRMANSSFGPPHRPRRIDSMRGELA

EIVTGLIDDFADARQIDVVDRFAYPFPVTVICRLLGVPREDEPRFRALVDPIVAGLDPGS

RTSAESMKTAQEARLQLGMYLNGLVEQRAKDPREDMLSDLVNSNGPDGAMTTMEVLSTAV

LLLIAGHETTVNLITNGMLTLLRHPRYLQRLRDDPGLSVKIVEELLRYEPPVQLLPQRTC

ITDIEVRGVTIPKGSRIWLVLAAGNRDPERFHDPDRFDPDREDIQHLGFGSGIHSCFGAP

LARLEAQLALAELVRRLDNPRLVEDPPPYRPNAVLRGPRHLNITFDGLR

>CYP156A28(2685040067)SGS

MYAPEFAADPYAAYAQMRERYGSLAPVYLDPDVPATLVIGYHTAVRILHDPERFPADPRI

WQQNVPTHCPVLPMLEWRPNALRNAGPAHERYRAANTAALGEARQHSLHSTVQQIAVPLI

NDICEVGHADLLAQYAHPLTFRALNAVLGCPPEVGQRVATGMSAVFEGVNAEAGNAMLAE

ALAELVDLKRSHPGMDITTGLLMHPARLNEFEMMHQLVTLYGAGIEPQQNLIANTLRLIL

SDDRYSGDVMRGSLSVRDALDELLFTDPPLANYCVSYPPSPVEIDNVVLPAHQPVVISMA

ACNNDPAAVSQQRAGNRSHLTWSAGPHACPAQPLAYLIAQVGIEQILDALPEMELAVPAD

QLTYRPGPFHRALTGLPVRFPPLPHLTLP

>CYP154A42(2685040068)SGS

MDRVPLLILDPTGRARPAEEAALHAAGPAILVDILGVTAWSVSDPAILKSLLNDPRVSKD

ASKHWPAFISGEITEKWPLALWVGVDNMFTAYGDSHRRLRRLVSQAFTARRTAEMVPRIE

QITGRLIDQMASKPGEVVELREALAFPLPIQVIGELMGLPEGVWPRFRTAVDGVFDTTLN

SDEARANTMSLYGILTELVAAKRNAPGEDITSALINARDYEGDGSVLTEQELLDTLLLVI

SAGYETTVNLLDQAVTALLTHPDQLAHVRAGRASWDAVIDETLRYAAPISHLPLRYAVED

IPLPEGMVIRQGEAILASYGAAGRHPNLHGTTAGTFDVTREDKQHLSFGYGSHFCLGALL

ARAEARAALPALFDRFPDIALAVLAEELEPLASLLVNGHRTLPVRL

>CYP1060A4(2685040231)

MGRMNEPPRLPGGGFAAWSRDRLGVARRGADECGDVWQLESGVYVAATAEPCEAVLRRAQ

DFPKAPSPLFPPLKRSSGAPTPEERAHAHAARMRGLRAQAVAARIGEIAPRTARCADQWP

TGQDVEILPLVRHALAEIGVHYLFSEDAPALLPFASQLFLAREVLVRPSRWAWPRWIPTP

ARRFRTRQQAAFTDALRPVVRRRRSSGRLGDDVLGQMLRPSSHYGPLPEEAVLDTLPGIT

VATFETPSRAAGWILLALARYPQAADRVAAEAALLPADPAATTSAHLDSLQYTQALVREV

LRLHPPSWLLTRRVARRTQLAGYTVDAGSTVLVCPYTAHRDAREHSDPDQFRPERWLGDA

DSPAKPGVFLSFGTGPHGCEGAALAMAMLTLLTAQTARRYHLSEPPGPEPSYQVATFEGL

ATVGLRLRATLRSREQSRFQ

>CYP157C35(2685040547)

VTPEPQSLTGTDPVPGPPPGCPAHGLGAGGPHRLYGPDAADLDDLYERLRDEHGPVAPVL

LHDDVPMWVVLGHTENLHLVRSPAQYTRDSRIWTPLQEGMVKPDHPLMPHIAWQPICSHA

EGDEHQRLRAAVTGAMSTIDHRNMRRHIGRYTQELVNDFCERGRADLVSQFAEHLPMAVM

CELFGMPEEYNDRMVQAARDALKGTETAIQSHTYVMDALSRLTTRRRARPDDDFTSHLIT

HPAGLTDDEVREHLRLVLFAAYEATANLLANALRMVLTEPGFRAQLNGGQMTVPEAIEQS

LWDEPPFSTVLGYFAKQDTELGGQIIRKGDGLLFAPAPGNVDPRVRPDLSASMQGNRSHL

AFGGGPHECPGQDIGRAIADVGVDALLTRLSDIQLDCPEEDLRWRSSIASRHLVALPVRF

EPKPQQDVQRPPSPAPIPSQRATWQTGTPRTEPAPAAEPRPMVPPPVPEPAVAPRQARRA

GLWRRLLRWWRGE

>CYP107KX1(2685041040)SGS

MTFASLPATLFGDDLVADPHSIYAQLRDADPVHRTVTPDGAPVWVVTRYEDVRAVLADPR

LSLNKSNAQTPDGYQSSMPPELDAHLLNMDPPDHTRLRRLVAKAFTPRRVEALRDRVQTM

TGGLLATMAGPRVDLMQALAVPLPMGVICELLGVPEEDRRDFRSWTDTLLSAAATTTDSR

AAMRQMHKYLTDIIQDKRSRPADDLLSALIHARDDRDSLTEPELLSLAFLLLFAGYNNAT

YLIGNTALGLMLDPKLLKAVQDDTVPIRAVIEESLRWNSPSPLGVRRFALEDVAIGGTVI

PAGSRVWVSIASANRDETKFPSPETFDPHRTTAHLAFGHGIHYCLGAPLARMEAEIAIPA

LVRRFPTLRLDIPENGPDWLHSFRKRGLKTLPVTC

>CYP1047B3P(2685041405)SGS

MNRLIGDRLAGGVDSGQGDDLLARLLAARDEGERALSPKEVRDEAVTLWAAGHATTSTAL

TWAWYLLARSRRARARLTEELDRVIGGRPPAFDDYEQLTELGHTEAEMADLAAAGTIRLP

DTANR

>CYP107KY1(2685041423)SGS

VQLSEPSDEIGNETETEMVGDSFARHPHDVYRRWREEGGVRKVRFAGAAPLAGWVVTGHA

ACRAALADPRLSKDGATEAYARHEGLPVGGPGGGLTSHMLNSDPPRHTRLRRLVQQAFTQ

RRVADLRPRIEAHVTTLLDALDGTDEGDEVDLIARFALPLPLAVIFDLLGADPAAHGILQ

VRGHTVSGDGGDGEVSVPTAEAMLERLRALIAEKRARPGDDLLSALLTAGDGGDRLTGQE

VTSMAFLLVIAGHQTTVNLVANGLLALLAHPAQLAAVRADPSLIPSLTDEVLRHESPFAL

ASLRYTTEPVTIDGTTIPAGEFVQIALAAANRDPEVFADPDRFDVTRDASQHLAFGHGVH

HCLGAPLARLQAEIAFAHVLRRFPGLRLAHPERTAEWQDNPRHRGLLTLPVRLR

>CYP1199A7(2685041424)SGS

MRRTLFHPRLAALFRDHLGQDVFRLEPDTVGVAGHEAADRILAARRATETERPTFKPLHG

RSISRGEASSVMRTIGGDVREALKKPRSRPEDVDLSGVWPLRGHLFLRDLILGQDPYRLR

ILMSRNLELTPKLTWAVIAAGAALPGRVGRRGAPLTAIAGLTAEAAGYQERRYAMGMYRR

AAAPVCFTVSTLVANALWLGAPFDPGTPNRHILYEAMRLLPPSWNILRNACPEYAALDER

IGTGDDVLVLPLLSHRDPKLWEDPHVFRPERWETLDPDTAPGYLPFGHSSERCWGRHMVM

PLAELLLDLVRGAGLAVSPEQTSAKVPLVGLLGVEDVQVTRVRRAAV

>CYP1419A6(2685041620)SGS

MTANASTTSLSVPGPTAAAVRAARRRDRAVYTRSHPLLFTLLAVTRRRSVTRIGDAVLVH

GTDPYRTALTRIPLDRTATGTTGGAARELSTGGTLFDQEGAGHRDARRALAEDVGAGGVE

RLRPVWRAVLARRLAPLGMGRTVDLVPLARELAGTTVCALLDAPGEPARVAEAAARAAAA

AVRDHLPGPRLPGTARAAAAATARLETLLATDTPGANGETALRAMLAVAAVNTTVAALPR

AVAWCSDARLWDEAADDRLRPALVGELLRVIAPSPLLPRVAAADANLDGCPVRAGDRLIL

VARHAAQAHNQPPDARHPTPPAVAQLVFGAGTHACPGARLARAQLDDTLAALASYRPTVV

RARVDRQAALPGWRSLTIRAVGDRESVEER

>CYP107BK3(2685041779)SGS

MTVVFDPRDPSVRTDPYPIYRRLREADPIHRSNFGYWVLSRYADVDAVLRAPEASSEFYR

NTTWANRRGGPESPLVQSVQNWMLMLDGPAHRRIRSVISKVFTRASVERLRPRIAAETER

LLDAVGEGDTDLIQSLALPLPVTVTCELLGLPTRDRDQCRRWTEHISRVIDPSITDEDAA

DMNAAEVEFREYVSGHIKERRATPREDILSLLVHADVDGERLTDAEIIANIQFLFVAGHE

TTVNLIGNGLLALLRHPEQLRILRENPELIANSVDEISRYDTPVQIVSRILGGSVELGDV

TLPEGAKVMLLFGAAGRDPERYPDPDRLDVTRTGTKTLAFSGGPHYCLGAGLGKMETAIV

LTELLRRYSKIELTGEDLVWRPNVSFRGLRELPLRLIR

>CYP2027A2(2685041839)SGS

VTTASSPVARGARRIPGPPRISTLPVLARMVRSRLDVMVWAAAEYGDAVRLPLGPKTLYF

FNHPDHAKHVLADNAENYTKGLGMIHARRALGDGLLTSEGELWRRQRRTIQPVFQAKRIT

RQLGAIGDEAERLAARLRSRIGEGPVDMRAEMTAFTLGVLGRTLIDADLGVFESLGGSFE

AVQNQAMFDAITLGKVPLWLPLPLQTRFRRARRDLQRIVDRLAADRAAGPAGDDVVSRLI

ESVRRQPDQRVGRIRMRDELVTLLLAGHETTASTLSWAFHLLDEHPDVWERLHDEAVAVF

GHGPLTIESLHELTYTTQVLDEVIRLCPPVWLLPRIARTDDEIGGFPVGAGADVLLCPYL

LHRHPAFWASPTRFDPERFTSAASAGRNRYSYIPFGAGPRFCVGNSLGMMEATVVLATVA

RDLRLTKVPGYEVAGEAMLTLRIRGGLPMTVRPVT

>CYP147F25(2685041873)SGS

MTTTETPDMLRRILDYSSRADPYPLYAELRRTPVARQEDGSYVVSTYRELTDLLHDPHLS

SDVRNLSRPMPQGQGDATPAFINMDPPEHDRLRRMAMRHFGPPHTPGLVTGVEPALTSAV

GSLIDDFAGKEQIDIVDDFAYPFPVSVICHLLGVPREDEPRFRVWVNDLINSIDYDPKTD

PKEKLEKGVQARKDLRQYLGGLLEQRHDRPGDDLLSRMANDDGPDGRMADDDIVSTANLL

LIAGHETTVNLITNGMLTLLRHPDVLQRLRDEPGLVVPLVEELLRYEPPVHIIPWRAAYS

DITVADTVIPKGSQIMLMLASGSRDQNRFHDPDRFDPDRRDNQHLGFGSGIHLCFGGPLA

RREAQIALGELVRRLDRPRLVADPPPYRRSPVLRGPIHLHVEQAAG

>CYP134A7(2685041957)SGS

MPVVNPRFSVLSEDFAASPYRSFAWLREQAPVHYEPAIDSYFLSHYRDVKRVLTDHETFT

TETLQVRAEPVMRGPVLAQMTGAEHTAKRKIVVRGFTGQALQDQIRAIRANTAELMDPFL

PRGRMDLVNDFGKPLAVHVTLDVLGLDRKDWQQVAAWHSGVAEFITSITLTPERRRHCMD

CAEQLEAYLVPVIEERRRRPGEDLISKLCTAEFDGIAMSDRDVTALIINVLAAATEPADK

TLALLFKHLIDHPEQLAQVRQDPNLLPAAIAETLRYTPPVQLIPRQAEENAVFAGTTVPA

GATVFCMIGAANRDPEAFAAPDTFDIHRPDLGTARSFTAAAQHLAFGTGLHQCVGAAFAR

AEIETVAAMLLPLLDQVRYSPGFRYRETGLYTRGPVSLSLDFTPVHESGRG

>CYP105D31(2685042198)SGS

MTELTDITGPATPAEPVAFPQDRTCPYHPPTAYDPLRDGRALARATLFDGREVWMVTGHS

TARALLADPRLSSDRTRPGFPVPIARFAAVRDRRLALLGVDDPLHHTQRRMMVPSFTLKR

ATELRPTIRRIVDGLLDAMIEKGPPAELVSAFALPVPSMVICGLLGVPYADHEFFEAQSR

RLLRGPSAADTQDARERLEAYLGDLVDLKARQAEPGDGVLDDLVHHPYREGALDRDELVS

LAVILLVAGHETTANMISLGTYTLLQHPERLAELRADPALLPAAVEELMRMLSIADGLLR

MALEDIEIGGTTIRAGDGVLFATSVINRDTARYDDPDSLDFHRSARHHVAFGFGIHQCLG

QNLARAELEIALGALIARLPGLRLAAPAEEIPFKPGDTIQGMLELPVTW

>CYP102G39b(2685042223)SGS

VHAFKPFGTGERACIGRQFALHEATMLLAMLVHRYRLHDHADYRLTVKETLTLKPEGFTL

TLTPRTDTDRVHAPLPGTATAAGAEGPAPDTLPVGVRPGTRALFLHGSNYGTCRAFAAQL

ADEAAAVGCVTEVAALDDYADGLPTDRPVMITAASYNGRPTDDATAFTAWLDGTPDLTGV

TYAVLGVGDRNWAATYQQVPTRIDARLAELGATRLTDRAAADASGDLTGTVRDFTTRLRT

ALLQEYGDPDATAAEEPATGYEVRTLTGGPLDALAERHGLVPMTVTEAYDLTAPGYPRTK

RFLRIALPDGVTYRTADHLTVLPANAPDLVDRAAAALGVDLGTVLDIRAGRPRRDGLATD

RPVTVRHLLTHQVELQERPTARQLALLAEANPCPPERAALAALTGDDPRTLVEIVEDHPS

LRGALDWPHLLDLLTPIRPRHYSLSSSPAADPRHADLMVSVLDAPARSGKGRYRGTGSGH

LASLLPGDTVYARVQPCREVFRIDGTAPVVMVAAGTGLAPFRGAVADRAAARAAGAELPP

ALLYFGCDAPDADFLHAGELRAAESAGTVGLRPAFSAAPENGALFEQHRIAAEADEVWDL

LTAGARVYVCGDGSRMAPGVREAFRALYRERTPGADDTAAVRWLDSLVADGRYVEDVYAA

G

>CYP102G39a(2685042224)SGS

MPPTALHPEPAGTLPGVPVVDITATGPGHTPIQQVMELMRAHGPVLVRRLHGRDVLMVGD

ADLVADLADEERFAKHIGPALENVRAFTADGLFTAYNDEPNWAKAHDILMPAFALGSMKT

YHPVMLKVARRLIGSWDRAARAGQPVNVPDDMTRMTLDTIGLAGFDYDFGSFERAEPHPF

VESMVRCLEWAMTHLARTPGKDYTEADAAFRADADYLAGVVDDVIAARTGTDQSGAEDLL

GLMLTARHPADGTTLDTANIRNQVITFLIAGHETTSGAMSFALYYLAKHPSALRLVQREA

DALWGDTPDPEPTYEDIGRLTYTRQVLNEALRLWPTAAAFSRHAREDTLLGGRIPLRAGQ

GRHRARPDAAPAARVGRQPGAVRPGALHARGGGRPPGARLQALRYR

>CYP102B14(2685042235)SGS

MAETAQRGLPKGFRGAEQGWPELHRIPHPPRRLPLLGDVLGVDRRRPLQDSLRFARQLGP

IFRRRAFGNEFVFLWGARLAADIADESRFAKHVGLGVANLRPVAGDGLFTAYNHEPNWQL

AHDVLAPGFSREAMEGYHRMMLAVADRLTGRWDREQAAGRAVDVPGDMTKLTLETIARTG

FGHDFGSFERERPHPFVTAMVGTLTYAQRLNTVPVPLAPLLLRGAARRNAADIAHLDGTV

DDLVAARRRSGGGDGDLLDRMLETAHPETGERLSAENVRKQVITFLVAGHETTSGALSFA

LYYLARHPEVAARARAEVDRVWGDTAEPGYDQVARLRYVRRVLDESLRLWPTAPAFAREA

RQDTVLAGEHPMRRGAWALVLTPMLHRDPEVWGEDAERFDPDRFDAKAVRARPAHTFKPF

GTGARACVGRQFALHEATLVLGLLLRRYELRPDPDYRLRITERLTVMPEGLRLRLERRTA

PSCAAARSVTVPDDGSGSGARCPVHGAGD

>CYP158A22(2685042553)SGS

MTGITEEPGPLTGQAPPPPVRDWPALDLDGTEFDPVLAGLMSEGPLTRIRLPFGEGWAWL

ATRYEDVKLVTNDPRFSRAEVTRRQVTRLAPNFAPRPGSLAWADQPAHNRLRRAVAGAFT

VSAMKQLRPRAQEILDELVDGVVRDGPPVDLVERVLEPFPLTVVSEVMGVPPADRDRVHA

WTRQIISTSGGTEAADRAKKGLYGWIGETVRARAVSPAEDVYTLLGAAVARGEITAEEAV

GLAGPLQIGGEAVTHHSGQTLFLILTRPELTERMRARPEARGPVLDELLRYIPHRSTVGL

ARIALEDVELAGRRIRAGEPVYVSYLAANRDPDAFPDPDTIHPDRDPNPHLAFGNGPHHC

TGAVLARLQTELLVGTLLDRLPGLRLAVPPEQVRWRHKTMIRGPRTLPVTW

>CYP105D1(641694565)SGR

MTESTTEPARQDPAPTAPPTQPTSTTPFPQNRDCPYHPPTGYQPLRADRPLSRVTLFDGR

PVWAVTGHALARRLLADPRLSTDRTHPDFPVPAERFANVERRRVALLGVDDPEHNAQRRM

LIPSFSVKRIAALRPRIQETVDGLLDAMERQGPPSELVADFALPVPSMVICALLGVPYAD

HEFFEGCSRRLLQGPGAADVNEARIELEGYLGALIDRKRVEPGEGLLDELIHRDHPGGPV

DREDLVSFAVILLVAGHETTANMISLGTFTLLNHPEQLEALRSGRTTTAAVVEELLRFLS

IAEGLQRLATEDIEVAGTTIREGEGVFFSTSLINRDTEVYENPETLDWDRPSRHHLAFGF

GVHQCLGQNLARTELDIALRTLFERLPGLRLAVPAHEIRHKPGDTIQGLLHLPVAW

>CYP1046A3(641694712)SGR

MSVDRTACPGQPSVPPGEGMLTHSRALRFWLDPANLAARREQAGPVVPTRTGPATAFQVN

DPALLRKIGTDEDTFRFWGPDPSLRDFTEDGVVGLEGAAHRKRRAVMRPAFSASRLTTLG

PAAQARTRRLLAGLPADRPLDMRMEMSRLACGLLVSCVLNSELAPDTLSRIASARSTLSS

GMFWRYALAPWPWVPVPRRRACRRALAELDEAVRQLLARHQPHPDGRDLVSVLEAATPEN

PRVVQRDVRALLIAGMETSASTLAWACYELGRHPHYQQALRDEADAAPDPSRLHADQLPL

ATAFVQEVTRLHGIPFLVRRTRHQTVQGGVRIPAGAVVTLPLGALRRDRDRYRDPDAFDP

KRWLPHAEPPPAPTALLAYGLGPRYCPGAAAADAMLPVALATLAGSRTLRPARPNRKIGM

SLELTPTPKGLTMYATPR

>CYP107AE5(641694722)SGR

MNDASEDSRSAPDLSSGCTAPRPRCPVRAVTSGSDGRESYLVTGYAEARDALSDVRLSKD

TAAFFAGKGSRRRLHAAVAHTMLASDPPRHTRLRKLVTGAFTSGSIAALRPSIARLTDEL

LDRWPAGGAVDVVAALAVPLPVMVICELLGVPETDRPRVQRWSADLFAAGDPGRTDAASH

AVAAYMTGLIVSKRLHPGDSLLDRLIAARERGDRLGEEELVSLAVLLLVAGHETTTNFLG

NAVLALLRHPAELNRLRGDPGTIPRALDELLRFDPPVSTSTFRFATEAMSLGGTEIPAGV

PVLVDINAANRDPERFTAPDRLDLDRDATGHLGFGHGIHRCLGAPLAKAEAHIALRAILT

RFPDIRLAVAADRLDRRRTRLISGLDSLPVLL

>CYP124G2(641694831)SGR

MLLAAGGRTMAWRTVEVMDMTVPYQLSEGRVLRAADVDLADPAFWRLPRPVRLRAFALLR

ELEEPVLFTPRAGTARTAGKPFRALVRHADVRTASRTPQVFASAPGVTTPEPAGWAKALF

GNSMVNMDGPEHAALRRIISRRFTPRLLAEAEENVGRLAGRLVDELIAERPRDFMPSAAS

RLPLEVICDLMGVPAAYRARIAEQIDHASEHVGVERRGRARLRIPGRGLASLARMQFVMG

RLARERRQRPEDDLVSALVNADIDGEALSGRQLGAFFSLLLVAGVETTRNAIAHGLFLLD

RHPEQRELLRSDFDRYIDGAVDEIVRHSTPIIQFRRTVAEECALGGRTFLPGEKVALIYA

SANRDETVFTHPDRFDITRSPNPHLGYGGGGPHHCLGAHLARLEMTALFRELIARRPVMR

DLGDPDLVDSNFDNRVGSLPFTFGPTFT

>CYP162C1(641694884)SGR

MSGEAGGCPAGTARGPDLTDPATYRDADYFAQWQRARRDHPVVRLESPRFGAFWSVTAHA

AARQVLERPESFTSTRGMRLGGEPAAVSAASGRMLVVSDGPAHTRLRSAHAPWFAGQAVS

RLKDALRSRLDALLADLADGSPVDVPARLARPLPTWLVCGMLGVPEEDWEELALLAAAAF

DETETSTASARRAASAGVFAYFAELLEKRRADPGDDLVSALVHQPGGDRLTDEEILLTCD

GLVNGGLGTTRHAVSGAVLAFAAHPRQWERLRADRGLVPTAVEEILRWVSPPLHIMRTAT

EDVLLGGARIRAGERVVLWIPSCNRDESVFAEPDAFRVDRRPNPHLGLGGGPHYCIGASL

ARLELRTLLRALLDHVARFESDPVLTRTPSTFLHGLDRLEVTLIPAADAAACSPAESEDP

>CYP208A1(641694913)SGR

MRTDPPGPPVSALPGLLRKLAVDRLEMMKDAAALGDAVRVSMGPKKLYIFNRPDYAKHVL

ADNSDNYHKGIGLVQSRRVLGDGLLTSDGEVWRAQRQTVQPAFKPGRINRQANAVAEEGA

KLVALLRAHEGGGPVDVLHEVTGLTLGVLGRTLLDSDLSSQDTLAPSFEEVQDQAMLEMV

SQGMVPGWLPLPPQARFRRARRELYRVADLLVADRSARMADGEPGDDALARIIEAAGRGN

GPPRRVRGKLREELVTLLLAGHETTASTLGWTLHLLERHPEVRAAVREEARSVLGERLPD

LDDLHRLTWTTKVVQEAMRLYPPVWVLPRVAQREDEVGGYTVSARADVLICPYIMHRNPR

LWEDPERFDPERFDPQAVASRPRYAYIPFGAGPRFCVGSNLGMMEAVFVTALITRDLDLR

TVPGHRAVAEPMLSLRMRGGLPMTVSVAG

>CYP154M2(641694924)SGR

MVVESRCPVVIDRTGQNIHAEADRLREQGPVARVELPGGVRAWSVTGYDVALSVLGDQRF

SKDPRKHWTAYANGEIGDDFPLIGWVLMDNLTTAHGSDHSRLRRLTAKAFTPRRVSAMRS

AIEQACTELLDELAESGPGDKVDLKARFAHPLPARVICDLVGVAPQDRAAMLRGGEVNVD

TTTSPEDAAANVERWHQEMHEFVESKRRTPGDDLTSDLIAAQEEDGSRLSPSELVGTLHL

MLGTGTEPVMNLIANAVHLLLTHPEQRAELRAGRISWDDVIEETLRAEAPVAHLPFRFPT

EDVEIGGVTIPRGDPVLIAFAAAGRDPAVHGPSAGRFDPSRADKAHLAFGHGIYRCIGMP

LAREEARIVLPALFTRFPDLDLAVAPEDIEPQGSFIMNGLRTLPVRLRSEGAGR

>CYP107BX5(641695112)SGR

MHRLFFEEPGPPRPAELPGGDPAWLVSRYADVRQVLSDPRFGRARLYAPEAPALSGVPDL

VNNPDLMFNQDGSDHLRLRRTLRRAFTPRAVARWRPWIAATVEGILDRLESRPQPADVVA

EFALPLPVAVISRLMGLDESVWDRMRYWSEHAFSDGTHEREQVAAALKEFSAFGAHLLAE

RRSTPGEDLVSGLVTAADEEGGVPEAQLVSLVCGLVVGGHDSTMTMLGNALLYLLGERRE

TWPRLGADEEAAGLLVERLVHLVPLGDDRGSTRHAAEDVEVSGVRIPAGAIVIADCGMAN

RDPEVFPPATLYDLFAPLEAPTLSFGAGPHYCLGAWLARTELQLALHRLAARFPELRLAD

PVDAVVWRTGTTSRSPRRLGVRW

>CYP107BY1(641695198)SGR

MTADPYPGYAWLREHDPVCPVGGPHVPGRMWLVTRYDDVRACLADRRLGSRAPVDPDPHP

PGLSHLDGPGHARLRRLVAAAFTPAAVARLRDRTARTCAHAVESFAGRGHADLVAEYTRE

IPVAVMHDLLGVPETERAPAADVLDMWYRAKFRQPRDEASLAELLDYVGELVAYKRTHPG

DDLTTRLIDSDALTGEELEVMVMTLIGAGHITTIQFLGTTVLHLLDHPGHRAALLRGDLD

WPRAVNELLRLDSPSHVAEYRYAGEDMTLADARVGEGDVVLLSLAAANRDPDRFPDPGTL

DLTRDARPHLAFGHGAHTCLGSHLVRLETEIAVTTLFGRLPDLALDIPGGEVAWGYAPTF

RGPLALPVTFTPSTSR

>CYP157C13(641695363)SGR

MTTPFHHEPGTVPPPQCPAHNLDIGPGGLRRLHGPEAENNPAGLYDKLRAEHGTVAPILL

HGDVPAWLVLGHSENLHVTRTPSQFSRDSRRWRALQDGSVAPDHPLAPIFTWQPICVFAD

GAKHERQRGAVTDSMERIDTRGVRRHINRFSNRLVNDFCEKGTADLVGQFAEHLPMMVVC

AIFGMPEEYDERLVQAARDMTRGTETAVASNAHIVSVLTRLVERRRAEPSPDLASWLVEH

PATMTDTEVIEHLRLIMIAAYESTANLIANVLRMVLIDPRFRARLSGGHMTVPEAVEQTL

WDEPPFTAVFGRWAVGDTELGGQQIKAGDALLVGIAPANTDPTVRPDLGADMGGNRAHLA

FSGGPHECPGQDIGRAIADVGVDALLMRLPDLELGVGESELHWVGNIMSRHLVELPVKFA

PGPQQKLDADPLTVMARAPRPADAWEISSPARQVPEPRHEAVVAQPAHAPGAAPTAEPDP

APAAPPAPEPAAAPEPAPVATIPQQRRPAAPARFWQAVTRWWSGY

>CYP1047A1(641695383)SGR

MSTQTGPALGTPPRGHAFVPGPRGLPLVGNLPQFGKNPLAFFELLRGHGDMVRWRFGRKR

CVFLADPDLVGELLTETERTFDQPRLGIAFRTVLGNGMLVARGRDWRRKRSLVQPSVRPK

QVTSYATTMAGCAVELADRLADGQRIDVKREMSALTQKIAVRTIFGVDTPADSEAMGRAM

DVAQMEIGKEFAGLGALLPDWVPTPGRTRIRKAAGVIDAEVRRVVARHRDGDEERPDLLS

RLLTAVDESGTRLSDEEIRDEAVTLYIGGHETTSTTLVWAWYLLARNPRVREALAEELDR

VLGDRDPGFGDYAQLTYAQAVVKETLRLFPAVWLITGIAKEGATIGGLPVAEGTRVWSSQ

WATHRDARWFPEPEEFRPERWDAESGDAIPEYAWFPFGGGPRVCIGTRFAMVESVLLLAV

LARRFTLDVDPGEITPLTGLTLQPDRDVLATVRAR

>CYP154C3(641695389)SGR

MNCPHTAAAQTDPGAGTVVIDPMVQDLDGETARLRDAGVLARIDLLGVPAWTVTRHAEAR

QLLLDQRLVKDIDAWGLWQSGVVTRAWPLIGMIDAGRSMFTVDGAEHRRLRTKTSQALTP

RRLEAIRPEIEKFTDELLDALDAARGEDGVVDLKSVFAQPLPMKVVGMLMGVDESQHAML

TRQYKAFFSMLTPQEERLALLAELDVFYTDLVREKTARPTDDLTSALILAEEGGEPLTEE

EVVGNLKAMVAAGHETTIGLVLNAVRALLSHPDQLRMVLAGEAGWDAVIEETLRWDTPTT

HLLMRFATEDITVGDDVIRKGEGVVVSYRAIGRDVGHHGPDADAFDITRPTRNRHMTFGH

GPHICPGAALSRVEAGIALPALFTRFPGLRLAVPDEEITKLPVMTQNDMTAFPVLLG

>CYP157A7(641695390)SGR

MTNPSSATPASTAGTGGGCPVGAGTGAVPLGGPGFLAEPREFYRSLRRDHGPVVPVELPG

GLPAWLVIGYRELHQVTSDGEMFPRDVSLWNQWENVPADWPLLPMVGTPMPSIYFTAGAE

HRRHVDMVVPALEEADPFEIRRHCEQLADRLIDAVCTRGTADLVAEYAEPLPVLVLARLV

GFPDDEGADIARVLKDLADGGPGAQKAHLSFGEHMQRLVAAKRARPGDDVTSRMLAHPEP

FTDQEYALDLMAITAAGHLTTADWISNSTRLMLTEDQFADALSGGRHSVAEAMNEVLWED

GPTQILAGRWAARDARLGGRDIARGDMLLLGLGAANADPHIRQQVTASAVRSGQGGNSAH

LAFSHGEYRCPFPAQEIAEIIARTGIEVLLDRLPDLELAVPATELVRRPSAFLRGTTALP

VRFTPVRTTGDAL

>CYP107L17(641695583)SGR

MTTEPLVDLAALGEQFTRDPYPAYAALRAKGPVHRVRIPEGAEAWLVVGYEQGRALLADQ

RLSKHWSRASPSLGVSKVSAGSSMLGSDAPDHTRMRKLVAREFTPRRMEQLAPRVQEMTD

GLLDAMLAAPDRTADLVEALSFPLPMAVICELLGVPSLDREAFRTWSGQAVSSVDPSLRA

SSTQAMTAYIAGLLADKRERPGEDLLSALIHTSDEDGDRLSGDELIGMAWLLLVAGHETT

VNLITNGVHNLLAHPDQLAALRADFTLIDNAVEEILRFEGPVETPTYRFTTDPIEVGGTV

IPGGGELVLVAMSDANRDPARYPDGSRFDITRDARGHIAFGHGIHYCLGAPLARIEARIA

IRSLLERCPELRSAADPATLPWRTGILMRGPLSLPVGW

>CYP107L16(641695584)SGR

MAVLDLRDLPDFTTNPYPYYAKLRAEGPVHAVRTEEMEQRVWLVVGHAEARAALADQRLG

KDWRHTGLWTESEAALSANMLELDAPHHTRLRRLVSREFTARRIEALRPRVTEITGELLD

AMAPRGSADLVDALAFPLPMTVICELLGVPDIDRDAFRALSNGIVTPTPEQRGADPAGAM

GAYLDGLIENKRRSPGDDLLSGLIRTGEADGEGLSSAELVGMAFLLLVAGHETTVNLIAN

GVRALLDHPDQLALLRADPGLLDNAVEEMLRYDGPVETATFRFARETLTIGDTEIHVDEP

VLVALASADRDPLRFRDPDTFDIRREPQGHLGFGHGVHYCLGAPLARMEARIAIGALLER

FPGLARDPSGGELDWLPGLLMRGARGLPVRW

>CYP159A7P(641695731)SGR

MRADSRRTDSRGHRMPADRPDALPAARRRHRLAGGQPGLLAPGATTDPYRLRLYRLLRTH

YPLGYDPGLGAWLLSRYTDVALALTDPRFTGYPHDGAPRGRAPVPLGLCRGSLVCVPPAV

PYRTAEPAVERTAYVLARRIARRDRADLVADFCRWLPAGAAAVAAGRGLSTLPRGGAPGR

RAGAVPDDCAGPTALREHALASFLANMLDDPDLLAAATAGDGAATLLGRAWAETLRRDPP

VQIVLRRTRTEVAVSGGTLPADAPVACLIGAAGRDPARFGAPDRFDPLRSDADPLLIGPA

GCPAALLGGLEAEHGLRALLAAMPGIRWADGFRPAAGGLLTRGPRTLLVRPS

>CYP157A6(641697429)SGR

MTTVSGCPVTHTSVPLSGPRFQSDPVQLYREMRRDHGAIAPVVLDGDVPAWLVLGYRELH

QVTGDPVLFSRDSDLWNQWDRIPDDWPLLPMIGRKQNSILYTVGERHSVRAMMISNALEG

VDPFSLKRYAEEFADELIDRFCTKGSVDIIAEYAKLLPALVLARIYGFSDEEAHPLVGAI

NDMIDGRERALAGQQHLATSMFRLLADKHAEPGDDVASRMIADTGGFTDEEVAQDLMVMM

AAGHQPTADWMGNSLRLMLTDDRFAASLSGGRHSVAEAMNEVLWEDTPTQNVAGRWAARD

THLGGRHIRAGDLLLLGLAAANGDPQVRTDGSALTGGNNAFLSFGHGEHRCPFPAQETAE

VIARTGIEVLLDRLPDVDLAVAADQLTRRPSPWLRGLTDLPVLFTPTPAIGRPGSFGGPA

>CYP154C4(641697430)SGR

MTRIALDPFVRDLDGESAALRAAGPLAEVELPGGVHVYAVTRHAEARALLTDSRVVKDIN

VWNAWQRGEIPMDWPLIGLANPGRSMLTVDGADHRRLRTLVAQALTVKRVERLRAGIEAL

TNASLDRLAAHPAGAPVDLKAEFAYPLPMNVISELMGVDAADHPRLKELFEKFFSTQTPP

EEVPQMMADLGTLFTKIVDSKRANPGDDLTSALIAASEDGDHLTDEEIVNTLQLIIAAGH

ETTISLIVNVVEALATHPEQREKVLNGEIGWDGVIEETLRWNTPTSHVLIRFATEDIKVG

DRVLPKGEGLIVSFGALGRDEEQYGPTAGDFDAGRTPNRHIAFGHGPHVCPGAALSRLEA

GIALPALYERFPELDLAVPAAELRNKPIVTQNDLHELPVKLGCPFGHDA

>CYP163B5(641697589)SGR

MTTATSTLHGVDLTDPQTFLDRKDDLVGLWQEFRSHSPVHWHPVEGRQVPGFWVLSRYRD

VMEVYRDNKRFTSERGNVLATLLEGGDSAAGQMLAVTDGRRHRELRNLLLKAFSPRLLAS

VVEGVRRRADRLVREAVGRGSCDFAQDVAEHIPMATIADLLGAPAADRDYLLSLTKQALS

AEEAGQSAEEAVVARNELLYYFSELAEIRREDPRDDVVSVLATATVDGKPLTEQEIVFNC

YSVIIGGDETSRLSMICAVHELMEHPDQWRRLVSGEVSVDSAVEEVLRWVTPAMHFGRRA

LTDVEIGGRTIRAGDVVTLWNSSANYDETVFDRPEEFDLARTPNKHVSFGYGPHFCLGAY

LGRGEIHALLTALRTHVAAMEPTAPARPIHSNFLHGYSSLPVSLRPVTGDRT

>CYP107BZ1(641697596)SGR

MTGTQTLSKYWMLTNEFTQNPYPVLDHVRREGPVRELSFPDGGRAWVVTRYEEAKAALAD

PRLSRDIHVHYRLMSRRTGRALTPPPEEANHLANLEPPRHTPLRRAISFAFTPRRAEALR

PKVERIADDLLDRLGEAPEAELIAGYADPLPVITIAELMGVPADAWPDFLRWSTALRTHS

PTDGSGVLDRNVQELSAYMADLIARKEREPGEDLLSALIHAAPENRLTPTEILSTGFALM

TGGNDTTASLVGGVIAALLTHPRERARLLADTGRWGKSMDELIRYVSPISNALQRVTTEP

VDVGGVTIPAGEVVVVCVMSTNRDTGQFPGHPDRLDLDRVKPAHLSFGFGIHYCSGAHLA

KVITEVSARRLFERFPAARLAVDPSRLRYQQNVVVRPLEALPVLLRP

>CYP107P7(641697732)SGR

MHVSFDPWSPAFVADPYPAYTALRAAGRAHWFEPTGQWLIPHHSDVSALLRDRRLGRTYL

HRFSHEEFGRTPPPAAHEPFTTLNGQGILDLEAPDHPRIRRLISRAFTPRTVENLAPTVR

RLAAELVDAFVAKGGGDLLAEVAEPLPVAVIAEMLGVPEADRGLLRPWSAAICGMFELNP

SEETAEAAVRASVDFSAYLRGLIAERRADPGEDLVSALIAAHDEGERLTEQEMISTCVLL

LNAGHEATVNTTVNGWRTLFHHPEQLAALRADPALLPSAVEELLRYDTPLQMFERWVLDD

IELDGQVIERGAEVALLFGSANRDPERFARPDTLDLSRQDNPHLTFGAGIHFCLGAPLAR

LELAASFGELLRKAPALRMTAEPEWHPGYVIRGLKELRAEV

>CYP156B5(641697819)SGR

MDPQPGATPYTAPAGCPMHQQRTALYGPEFAADPHRFYDAARTHGPAAPIELSPGVEATL

IVQHEAALRVLQNPALFARDSRRWAALREGAVPMDSPVLPMMVYRPNCLFTDGAEHLRLR

KAVTESLSRLNSSRLSRDVERIADYLIDQFIERGTADLLNEYAKLLPLLLFNQLFGCPGD

IGDRLTRSMSAIFDGEDVLRANAELTECLMELVALKRRQPGEDITSWLIQHPAGLRDEEL

KDQLVVLMGAGVEPERNLIANALLLMLAGEAPGAPERRGSGMLVEDALDDVLWNNPPIAN

YATHFPVQDIELDGVVLKAETPVLISFAAANSDPGLTDARQTLSKGAHLAWGAGPHVCPA

KSPATLIALTAIEKILNTVPDLALAVPASGVAWRPGPFHRALVALPVRFTPTAARRAATG

AQPAAPVSAQLPDPFRNTPSAPSAAPRHAQEPAKKQKGWWSSFLDVFRV

>CYP159A1(641698743)SGR

MFMSVPAPDILSPEFERDPYRAYRLMRQDTPLMWHEATGSYIVSRYEDVERVFKDKEGEF

TTENYDWQIEPVHGRTILQLSGREHAVRRALVAPAFRGSDLREKFLPVIERNSRELIDGF

RDAGSADLVADYATRFPVNVIADMLGLDKSDYERFHGWYTAVIAFLGNLSGDAEVARAGE

RTRVEFAEYMLPIIRKRREAPGDDLLSTLCTAEVDGVRMGDEDIKAFCSLLLAAGGETTD

KAIAGIFTNLLRHPEQLEAVRADRGLIPRAFAETLRYTPPVHMIMRKSATEVELSGGTVP

AGVTVTCLIGAANRDEDRYRNPDSFDIFREDLTATNAFSAAADHLAFALGRHFCVGALLA

KAEVEIGVGQLLDAMPDLRLADGFDPVERGVFTRGPQSLPVRFTPVSG

>CYP157B14(641698744)SGR

MSTSSPSFGPQAPASCPVGAGAGAVRLSGASYQQTPTQLYRSLRRDHGAVAPVLLDGDVP

AWLVLGYAELSYVLTHDELFARDSRRWNQWETIPPDWPLMPFVGYQPSVLFTEGDEHRRR

AGVITEALEGIDQFELARDCRRIADRLIADFAGSGRTELMSSYVHALPMRAVVEMCGMPV

SGSDTQQLVDDLRISLDAGEGDDPVAAYGRVGDRLRQLVEDKRAAPGADITSRMVTHGAG

LTDEEIVQDLISVIAAAQQPTANWICNTLRLLLTDERFALNVSGGRLSVGEALNEVLWLD

TPTQNFIGRWAVRDTQLGGRHIRAGDCLVLGLAAANTDPEIWPESYVGAENSAHLSFSGG

EHRCPYPAPLLADVMARTAVETLLEQLPDLMLAVDPTELSWRPSIWMRGLSTLPVQFSPM

AQ

>CYP107U8(641698788)SGR

MNDSPAPRPSEPSACPHSPAGPHGAPELFTWEFATDPYPAYAWLREHRPVHRTALPSGVE

AWLVTRYGDAREALADARLSKNPANHAESPHAKGKTGIPGERKAELMTHLLNIDPPDHTR

LRRLVSKAFTPRRVAEFAPRVQELTDRLIDDFVEKGSADLIHDFAFPLPIYAICDLLGVP

EEDQDDFRDWAGMMIRHGGGPRGGVARSVKKMRGYLAELIHRKRENPGDDLISGLIRASD

HGEHLTENEAAAMAFILLFAGFETTVNLIGNGTYALLRHPGQRARLEASLAAGESALLAT

GLEELLRFDGPVEMATWRYATEALTLGGEEIAAGDPVLVVLAAADRDPARFTDPDTLDLA

RRDNQHLGYGHGIHYCLGAPLARLEGQTALTTLLRRLPDLRLAGEPGDLRWRGGLIMRGL

RTLPVAFEPGSRTRKSDTASTL

>CYP125A19(641699541)SGR

MRCPHLPDGFDFTDPDLLQSRVPHPEFALMRETAPVWWCTQPRNISGFGDEGYWAVTRHA

DVKYVSTHPELFSSNTNTAVIRFNETISRDQIDVQKLIMLNMDPPEHTRVRQIVQRGFTP

RAVRSLEAALRSRARSIVGTAQASADAHGSFDFVTDIAVELPLQAIAELIGVPQEDRSKI

FDWSNKMAAYDDPEYAITEEVGAEAAMEIVAYSMNLAAARKECPAQDIVSQLVAAEGEGN

LSSDEFGFFVILLAVAGNETTRNAISHGMHAFLTHPEQWELYKRERPKTTAEEIVRWATP

VVSFQRTATQDVELGGQRIRKGERVGLFYSSANNDPEVFDAPEAFDITRDPNPHLGFGGG

GPHFCLGKSLAVMEIDLIFNAIADVLPDLRLLEDPRRLRSAWLNGIKELRVTTAAA

>CYP107F4(641701006)SGR

MENTSVQNKETVRNCPFDYAHELEFDPQLRQLLTEEPVSRIRMAYGEGEAWLVTRYEDVR

TVTTDRRFSRSAVLGRDFPRMTPEPIVQAESINLMDPPASSRLRGLVAKSFTPRRVEQMR

GGTQRVVDRLLDEMEEEGSPADFVARVSAPLPLITICEALDIPEADRPWLRAHAMTMMNV

GAAGKQDAVRAKAELRGYFQELTADRRRSPGEDLISTLATARDGDELLDDDELAVMAMVL

LITGQDTTTYQLGNIAYTLLTRPDLLRSLRAEPQRLPRTLEELLRHIPFRKGVGIPRIAL

EDVELSGVLIKAGDVVHVSYLTANRDSAKFDRPDELDPDRPTIPHMTFGWGAHHCLGAPL

ATMELEVAFSTLLTRFPALRLDVPPEDVSWNTTSIWRYPLALPVTW

>CYP107CA2(641701176)SGR

MTATGHEIRDYPFGPVDRLDLDPALVEICGEHPVLRVRLPFGGDGWLVTRYADVRAVLSD

PRFSRSAAAGDHVPRTVAVAPPPTSIMGMDPPDHTRLRRRVMRAFTVRSIDALRPRIAEI

VNDLVDTMTEGDGPADLAAVLTWPLPITVICEMLGVPRADQDRFTEWVDGLLILDDPERS

ADARRQLGDYLAVLIAQRRAEPTDDLLGELAADSGKDPLSEEELVGLGVSLLSAGQEATA

NQIGNFVYTLLTRPALWRELVADPSIVPRAVEELSRFIPISATAGFTRVATEDLELGGQL

IRAGDAVVAELGMANRDSAVFDRPEEIDFHREQIPHVTFGYGIHHCLGAQLARVELRVVL

ETLVTRLPGLRLAVPADQLAWRTERLIRGVAALPVRW

>CYP102B7(SHJG_8045)shy

MAATTETMETGLPKGFRSAEQGWPELRRIPRPPHRVPLLGDVLGVDRHRPLQDSMRLARE

LGPIFRRRAFGKEFVFVWGARLVADLADESRFAKHVGLGVANLRPVAGDGLFTAYNHEPN

WQLAHDVLAPGFSREAMEGYHGMMLSVADRLTDHWDRHLAAGRTVDVPGDMTRLTLETIA

RTGFGHDFGSFERDRPHPFVTAMVGTLAYAQRLNSVPGPLAPLLLRTAARRNAADIAHLN

RTVDGLVAARRRSGGGEGDLLDRMLATAHPRTGEKLSPENVRKQVITFLVAGHETTSGAL

SFALHHLARHPEIAARARAEVAQVWGDTPRPGYDQVARLRYVRRVLDESLRLWPTAPAFA

REARRDTVLAGDHPMRRGAWTLVLTPMLHREPEVWGEDAERFDPDRFTPAAVRARPPHTF

KPFGTGARACIGRQFALHEATLVLGLLLRRYDLHADPGYRLSVAERLTLMPEGLRLRLER

RPALTKADEGRPAGARGSVRGAADAAWARPARDAAADDRRHDPAGPAQPPSASRCPVHGA

AD

>CYP102G6(SHJG_8055)shy

MPPTALRTEPAGTLTGVPVVDITASGPGRTPIQQTMELMREHGPVLVRRLHGRDVMFVAD

ANLVADLADEERFAKHVGPALRNVREFAADGLFTAYNDEPNWAKAHDILMPAFALGSMRT

YHPVMLKVARRLIDAWDRAARAGRPVDVPDDMTRMTLDTIGLAGFDYDFGSFERAEPHPF

VESMVRCLEWSMTRLARTPGTDHSAADAAFRKDADHLARVVDDVIAARTGTDQSGAEDLL

GLMLSAPHPADGTTLDAANIRNQVITFLIAGHETTSGAMSFALYYLAKHPAVLRLVQREA

DALWGDTADPEPSYEDIGRLTHTRQVLNEALRLWPTAAAFSRHAREDTLLGGRIPLRAGQ

AVTVLAPMLHRQPVWGDNPELFDPERFTPEAEAARPVHAFKPFGTGERACIGRQFALHEA

TMLLALLVHRYRLHDHAGYRLTVKETLTLK

>CYP105D11(SHJG_8085)shy

MTELTDISAPAAPPGPVTFPQNRTCPYHPPTGYGPLRDGRPLSRVTLYDGREVWLVTGYS

AARALLADPRLSTERRRPGFPMPTPRFAAGRDRRVALLGVDDPEHHRQRRMLIPSFTVKR

AAALRPWIQRIVDELLDAMIAQGPPAELVSAFALPVPSMVICGLLGVPYADHEFFEEQSR

RLLRGPTAADTVQARGRLEDYLGGLIDAKAAEAEPGDGILDELVHDRLRTGELDRDDAVS

LAIILLVAGHETTANMISLGTYTLLRHPDRLAELRADPALLPAAVEELMRMLSIAEGLQR

VALEDIEVDGTTIRAGDGVLFGTSVINRDTSVYDDPDALDFHRPDRHHVAFGFGIHQCLG

QNLARAELEIALGSLFTRLPELRLAVPAEEIPFKPGDTVQGMLELPVTW

>CYP105AZ1(SHJG_1918)shy

MTQSADAAPETGSPLPRFPMRRTCPFSEPREYAGMRANAPVSRAALKVNGKPTWLVTRHE

DVRQVLGDSRVSSNLKLPGYPHQFHIPEEMLAQVRLMMLNMDPPEHSAQRRMLIPEFTAR

RVREMRPRIQQIVDEHVDAMLAQGGPVDLVTALALPVPSLVICELLGVPYEDHAQFEEWS

AAMMNHDLSPAEYGAAVQALDTYLDKLVTLKEDEPGDDLISRFLEKNRTEKVADHVDVVT

MARLMLVGGHETTANMIALGVLALLRHPEQMAALRDDPALLPNAIEELLRVFSISDSGTA

RVAVADIEVGGVTIRAGEGILALNNAANHDESVFPDPGTLDIRREEARSHLAFGYGIHQC

IGANLARVELETVYGTLLRRVPGLRLAAEPEELRFKDDAMVYGVYELPVTW

>CYP105AZ2(SHJG_1917)shy

MMTAPAEPADEAALPEFPMRRACPFSPPAAYAELRETEPVSRARLKVNGKPAWLVTRHDL

YKKLLGDSRVSANLKLPGYPLQVPVPQETLQSVPLTFLSMDPPDHTVQRRMLAPEFSVRR

MRELRGRVQQIVDEQIDQMLAKGADGPVDLVTALALPVPSLVICELLGVPYEDHGRFEEW

AWAIMNHDISDEDRGRAHYELDRYVDGLVTAKESEPGDDMISRLIEFNRRTPAVEHSDIV

SMSKLMLVTGHETTANMIALGVLALLEHPDQLAALRAEPELMPKAVEELLRLFSISDAGT

ARVALEDIELGGVTIRAGEGILPLNNAANHDERVFPDPDRLDVRREARSHLAFGYGVHQC

IGQNLARMELDVVYSTLLRRVPTLRLAAPVEELRFKDDAIVYGLYELPVTW

>CYP107F6(SHJG_2265)shy

MSSHDPAVLDCPFDFADALEYDPALDALARRGPVSRIRLPYGEAEAWLVTSFAGVRQVTC

DPRFSRAAIVGRDYPRMTPEPIVSPESINVTDPPHATRLRHVAAQAFTRERVAAMRPAVD

RVVAGLLTAMDEAGPPADLVTHLSVPLPHLTICELLAVPEADRDELRAHTMRLLATSPDA

RQDAADAKACLRTYFAERIPARRRSPGEDLLSTLATAPVPEGEEPLSDDELAVLAVTLIL

SGNDTATCQISDIAYLLLTRPEEMAALARDPGRFPGALEELLRFIPFRKGVGIPRIALED

AEIEGVPIRAGDYVHVSYLAANRDPDVFPDPHRLDLERPVRPHMTFGWGGHHCLAAPLAR

AELDSAVSGLLARFPKLRLDVPAEDIEWDNGTIRRFPLSLPVTW

>CYP107L22(SHJG_7304)shy

MIDLTEYGDALRRDPHPVYARLRERGPVHRVRLATPGGAWETWLVVGYEEARAALADQRL

AKDIARTGFVPLDEQLIGKYLLVADPPQHTRLRGLVARAFTMRRVERLRPRIRQITDELL

DDMLPRGHADLVDSLAYPLPITVICELLGVPEMDRAEFRKTSTEVVAPTGTDSERAATVR

LAEYLTELIEDKRCAGPTGDLLSDLIRTTAEDGDRLSMDELRGMAYLLLIAGHETTVNLI

GNAVLALLTHPDQLAALRADPSLLDGAVEETLRWEGPVETATYRFAAEPLEIAGTRIGRG

DDVLVGLTAAQRDGARFTDPDRFDIRRDTRGHLAFGHGIHYCLGAPLARLEAGIALGALL

DRAPGLALDGEPGEWLPGMLMRGVRSLPVRW

>CYP107P2(SHJG_5396)shy

MAGLDDLAFDPWDPAFVADPYPAYAELRARGRVIRYEPTDQWLVPHHADVSALLRDRRLG

RTYQHRFGHEEFGRTPPPPEHEPFHTLNDHGMLDLEPPDHTRIRRLVSKAFTPRTVERLR

PYVEGLANDLVAALVGNGGGDLLKDVAEPLPVAVIAEMLGIPEADRGQLRPWSADICGMY

ELNPSEETAARAVRASVEFSDCLRELIAARREDPGDDLISGLIAAHDEGDRLTEQEMIST

AVLLLNAGHEATVNATVNGWWALFRNPDQLAALRADHSLIPSAIEELMRYDTPLQLFERW

VLDDIEIDGTTVPRGSEIAMLFGSANHDPAVFTDPARLDLTRKDNPHISFSAGIHYCIGA

PLARLELTASMTALLRQAPTLTPTEEPTRKPNFVIRGLEGLGVELG

>CYP107U1(SHJG_4569)shy

MTDQPHPAQPSSHRDQPAPALFTWEFATDPYPAYAWLREHAPVHRTRLPSGVEAWLVTRY

ADARQALADQRLSKNPAHHDEPAHAKGKTGIPGERKAELMTHLLNIDPPDHTRLRRLVSK

AFTPRRVAEFAPRVRELTDHLIDQFARRGSADLIHEFAFPLPIYAICDLLGVPREDQDDF

RDWAGMMIRHGGGPRGGVARSVKKMRGYLAELIHKKREALPAEPAPGEDLISGLIRASDH

GEHLTENEAAAMAFILLFAGFETTVNLIGNGTYALLTHPDQRRRLEESLARRETELLATG

VEELLRFDGPVELATWRFATEPLTIGGQRIAPGDPVLVVLAAADRDPERFADPDVLDLAR

RDNQHLGYGHGIHYCLGAPLARLEGQTALSTLLTRLPDLRLAADPAELRWRGGLIMRGLR

TLPVEFTPSA

>CYP107X7(SHJG_0818)shy

MSNPDPTSPSEPAVDSAELFQDPYAVYGRLREEGPVHRITGTDGLPAWLVTRYDDVRRAL

ADPRLSLDKRNATPGGYRGMALPPALDANLLNMDPPDHTRIRRMVTKVFTPRHVEGLRVP

IRRAADRLLDSLAGQDEADLIPSYAAPLPITVICDLLGVPQAQRPDFRAWTDALVAPDPT

RPERAREAVGRLLSFFTRLIADKRARPADDLLSALIAVRDEEDRLSEDELMSLAFLILVA

GYENTVHLIGNAVAALLAHPDQLAALRADVGLLDRAVEELARYDGPVPLAIRRFPTEDIV

IGGATVPAGETVLLSLAAAHRDPRRFTDPDRLDLGRDATGHLALGHGIHYCLGAPLARME

TGIALTALFDRFPGLSLAVPPQDLRRRPSMRSRGLLALPVRTADRPGRTGRGASVG

>CYP107AH2(SHJG_2350)shy

MTTTHEAVAAAERCTPEFRRDPHAVYAHLRDSAPVCPMRPPHGNETYLVTRYDDARAALS

DPRLSKDMYGAMDAYRRIFGDSSVALDDNMLNSDAPKHTRLRRLVNSAFTPRRVEALRPR

IEEIVRDLLDECPARERFDLLPAFAFPLPIIVICDLLGVPPEDRTRMQHLSTTVAQTGFG

EEAKRAQQQAEEGLHAYFTDLLTAKRERPGDDLLSALIAARDNDGGLTESELVSTAFLLM

FAGHKTTAYLIGNAVHHLLSHPAQLRAVREDPELIRAAVEELVRYDGSVESATFRYATED

VEYGGTLIPKGALVQIAISSANRDPRKFDAPDELDVRRPGNAQDAHLGFGHGSHYCLGAP

LARLETQLALTRLFERFPRMALADPAGAPRWLEVPFPAFRGLAELPVVLDPAG

>CYP107BM5(SHJG_8434)shy

MSQQPVLLPYADPAFVADPFPLYRRLREEGPVRRAIIAGGLDAWLVTRYEDGLAALSDPR

LSSDVRDASDPRLIEQLPEFERESMLSTMLRSDPPDHTRLRRLVSKAFTARRVAGLRPRI

QEITDRLLDAVVPAGRAELVADFALPLPVTVISELLGVPVDDRYDFQRWTDAMLVRGEEM

PDPVVVDEAWHRMRAYLAKHLEAKRARPGDDLLSALINAHDHEQRLSHDELIAMTFLLLV

AGYITTVNLIAGGIVALLTHPGQLALLRERPELLPDAIEEFLRYDGPVSPGIARFAREDV

EIAGVTVPRGATVLIASAIADRDPARFTEPERLDITRRDNGHLAFGHGIHYCLGAPLARL

EGQVAVGTVLRRLPDLALAVPPAQLAWRPGGLRGPARLPVTFTPGGLG

>CYP107CH3(SHJG_0351)shy

MSAHEHCPYQDGRVVIDHAFKADSPARYARLRRLGPIHPAEFHLGLKGWVIVGHDLAREA

LTHPALLKDATPAAEALAAAGYVLHQPSVGLGAQMMEADPPEHTRLRRLASAAFTPRRTA

ELAPRIERIAHDLIDAMPPSGELDLVEAFNAPLPATVIAELLGIPPRYHLDFRRWSGQAL

QVASPEHRPALAALHGLLAGLVADKRRRPQDDLLSALVAVRDEEDGRLSEEELVGTAMML

VVAGHESTVHLLGNAVLALLRHPGQLRLLRERPELVPGAVEEFLRYDTSVERSTSRYASE

DLELGGVPIPRGSMVVVALGSAGHDAPQPDGDDPAVLDVTRSNARHLAFGHGIHHCLGAP

LARLETAIALRTLLSRVPELELAVPPDSLDWIGSGIIRGVLSLPVRYRVG

>CYP107CH4(SHJG_0497)shy

MDTHQQARPVGEPQERVIMSPEFKADAHRQYARLRAKGPVHPAQFFPGITGWVVVDYDLA

REALTHPALLKDPEPAAEKLEAAGFLGHKRGTGFGGQMLEADPPEHTRLRRLVSGAFSPK

RTAGMEPRITEIADRLVDAMPPSGELDLVEAFTAPLPVAVIAELLGIPEADRQDFRRWTT

LAFQVGHPEYASAVASLHGFLRGLADDKRRAPGDDLMSALVAARDEDDGRLSQDELAGTA

ALLVVAGHETTVNLLGNAVLALLQHPGQLRLLREDLGLLPDAIEEFLRYDTSVERTTNRY

AAEDLELGGVRIPRGGVVAVALASASRAAPLPGGGDPDVLDITRPAARHLSFGHGIHHCL

GAPLARLEARVALRTLLARVPHLELAVPADSLDWFPAGMVRGVLSLPVRYRRT

>CYP113K2(SHJG_8125)shy

MISKQPTGERLLLEELPDRWRGLREAGPVRYDETQGVWQVLDHETVAAVLADPATYSSDM

SALAPTQSDFETFRQGNFVGMDPPEHRKLRTLVSQAFTPRVVQGLGPRIEAVCARLLDAV

ADRDRFDLVDALAYPLPIIVIAELLGIPAEEHRLFQEWASVLFGGDQLGEAPDMADLERA

LEAIAPTVREMNGYMLDHIRARRADPGDDLTSRLIAAEVDGVRLADQEMVGFVALLLVAG

HITTTALLGNAVVTFDRHPGTNAALRAEPARIPAAVEEVLRWLPPFPELGRRVTRPVVLG

GHEIAPDTLLMAHLGAANRDPARFAAPDVFDVTRHPNPHLTFGHGIHFCFGAPLARLEAR

IALRMLHERFRMLAIPSYEDIAYQNPAVIIGVRHLPVEVRRP

>CYP147B3(SHJG_1823)shy

MASETLLARITDYASRPDPYPLYAELRAAGPVVQQADGSYLIGTYHEVAALLHDPRMSVD

PRTRGGEAQQLPFLRLDDPEHHRLRTLAMRPFGPPHSPRRVDGMRGEIDRITQELLASFR

AGEQIDLVDDFAYPLPVTVICRLLGVPREDEPLFRAWSDALVAAADVRPGADTTETDKAG

EQARMEMGGYLVNLAEQRRGKPSDDMLSAFVNEPDPALRLTREELAETAVLLLIAGHETT

VNLITNGVLTLLRRPDQLDLLRREPDLLPRAVEELLRFEPPVHMRERIPRADVDVAGTTL

PEGASVILVLASASRDPKRFDEPDRFDPTRPDNQHFGFGSGIHLCYGGPLARIEAYSALG

ALLPHLGTARLVQDPPPYRQNAMLRGPRHLPLQL

>CYP147F10(SHJG_2714)shy

MPHASIARRITDYANRANPYPVYEELRRTPVLHEEEGGPYLISSYYDIKALLHDPRISSD

AANVAAAGDDELDQTEETGGLPPSFLRLDPPEHDRLRRIANSAFGPPHRPRRIENMRGEM

REIVTGLIDAFGDARQVDLVDQFAYPFPVTVICRLLGVPREDEPRFRTWVDPLVASLDPA

AGPDGDSDARKRAREARMQLGMYLAGLVEERTKEPRDDLLSDLAAGRGPHGSLSMMEVLS

TSVLLLIAGHETTVNLITNGMLTLLRHPEILQRLRADPGLSVNIVEELLRYEPPVQLVPQ

RTCITDIEVRGVTIPKGSRIWLVLAAGNRDPERFTDPQRFDPDRGDIQHLGFGSGIHSCF

GAPLARLEAQIALSELARRLEGPRLVEDPPPYRRNAVLRGPRHLDVAFDGLR

>CYP154C1(SHJG_8229)shy

MTTGTEAPRIALDPFVTDLDAESAALRAAGPLAAVELPGGVPVWAVTHHAEAKALLTDPR

LVKDINVWGAWRRGEIPADWPLIGLANPGRSMLTVDGADHRRLRTLVAQALTPRRVERMR

ERIEKLTQDLLDALPADGGTVDLKAAFAYPLPMYVIADLMGIDEGLLPRLKVLFEKFFST

QTAPEEVVATLTELAQIMARTVSAKRAAPGDDLTSALILASEDGDRLTDEEIVSTLQLMV

AAGHETTISLIVNAVVNLSTHPEQRALVLSGRADWSAVVEETLRYSTPTSHVLIRFATED

VPVGDKVIPAGDALIVSYGAIGRDEGAHGPTAGEFDITRDTRNRHISFGHGPHVCPGAAL

SRLEAGVALPALYARFPGLDLAVPASELRNKPVVTQNDLFALPVRLNP

>CYP154D10(SHJG_2299)shy

MDTSAARTPHRLDPAGGCPHAVNARLLARGAVAPVELPGGIEGMAVLGHEALKEFLQHPH

VAKNARHFTALREGRVGEGWPLLTFATVPGMTTADGDDHRRLRSLAARAFTPRRVAELRP

RVEELTESLLDGLARAAAAGDGVADLRRHFALPLPMGVICELLGVDVEFRDRLHRLGSLV

VATDTAPAEAVAANRDIVAVLGEIAAAKAARPGDDLTSALLAARDEGGDRLGEQELIGTL

LLMIIAGHETTLNLITNAVRALCAHPEQLGLVRAGRASWADVVEETLRWDAPVSYFPFRY

PVRDLTVAGTLVPAGTPVLAGYSAAGRDPAAHGPDADRFDLTRSARPGAVRHLSLGHGAH

YCLGAPLARLEATVALERLFTRFPSLRLAVPEAGLVPHASFVGNSVRALPVRLAAG

>CYP154D9(SHJG_0095)shy

MLGHAELKAFLADPDVAKDPHHFAALQRGEIPEGWPLTLFCSVEGMANRDGSDHRRLRRL

VGEAFTPGRVAALRPRITKLTEKLLDQVGAAASTSGEGIVDVRQQLSVPLPLEVIGELMG

IPQEHRDPLHDLSVRIVATQAEPAETLTANLEMATLMATVVAAKAQQPGDDLISALLAAR

DGDDRLTQEELIGTLIVLIVAGHDTTTHLVTNAIRALCTHREQLALVRSGQASWEDVVEE

TLRWDAPVSYFPFRYPVKDITVGGTELPAGTPVLAAYSAAGRDRRAFGPDADRFDVTRSR

RPGAARHISFGHGPHFCLGAPLARLEASVALERLFARWPDLDIVASNDELGRYPTFVGNS

VLHLPVRLTP

>CYP156B11(SHJG_2491)shy

MHDAAFAADPHQVYDRLRAHGPAGPVELAPGVDATLVVGHEMALRVLQNSTLFARDARRW

KALNEGAIGLDSPVLPMMAYRPNCLFTDGAVHLRLRKAVTDSLARLNVTRIRRDVEPIAD

YLIDQFSERGRADLLNDYAKLLPLLLFNKLFGCPADIGDTLTSAMSAIFDGKDALRANEE

LTACLMELIALKRRRPGDDVTSWLIQHPAGLTDEELKDQLVMLMGAGVEPERNLIGNALL

LLLSPDTSGRDSGLLVEEAIDDVLWNETPIANYATHFPVQDVDLGGVVAEANTPVVISFA

AANSDPALAEARRKHSKGAHLAWGAGPHACPAKDPAQVIAVTAIEKILNALPDLTLAVPE

KELAWRPGPFHRALVALPVVFSTTPATRMVSALQNRTGSAAAEQPLPSVPASAASAGRQE

SARKKGFWSSFLDIFRV

>CYP157A13(SHJG_8230)shy

MTTPDAVPLSGPRFQTEPARLYREMRRDHGAVVPVLLDGGIPAWLVLGYRELHQVTGDPV

LFSRDSDLWNQWENIPADWPLLPMIGHKQPSILYTVGERHRQRAAMISNALEEVDPFELR

AHAERFADELIDALCSAGEADLVAQYAMLLPVRVLARLYGFPDEDGPGLVTALNDMIDGR

ERALAGQAHLAASMAQLLADRRKEPAADVVSRMLADDSGFSDEEIAQDLMVMMAAGHQPT

ADWIGNSLRLMLTDDRFAASLFGGRNSVAEAMNEVLWEDTPTQNVAGRWAARDTHLGGRR

VKAGDLLLLGLQGANSDPQVRTDASALTGGNNAHFSFGHGEHRCPFPAQEIAEVIARTGI

EVVLDRLPDIDLAVPAESLTRRPSPWLRGLSGLPVRFSPVPAR

>CYP157B2(SHJG_1327)shy

MSDTTGFPSTDAPPPGCPAHGSAVPLAGLEYQQTPSQLYRTLRREHGAVAPVLLDGGIPA

WLVLGYPEVCYVTAHDELFARDSRRWNQWEHIPPDWPLLPYVGYQPSVLFTEGAEHQRRA

GVITQALEGVDQFELARECQLIAARLISSFSGSGRAELMSMYAHALPARGVLWMCGMPAE

DTDTERLVDDLRISLDAGEGDDPVAAYTRVGERIMRLVKEKRERPGPDVTSRMILHPAGL

GDEEIVQDLISVIAAAQQPTANWICNTLRLLLTDERFAVNVAGGRVSVGEALNEVLWLDT

PTQNFIGRWAVRDTQLGGRHIREGDCLVLGLAAANTDPQIWPEPHAGSGNSAHLSFSNGE

HRCPYPAPLLADVMARTAVETLLEHLPDLVLAVEPEELTWRPSIWMRGLTSLPVEFTPAM

N

>CYP157C19(SHJG_3061)shy

MTPEFPSPTGTPDTLSGPPPGCPAHGLGPGSPRRLYGPDAEDLGDLYERLREEHGPVAPV

LLHDDVPMWVVLGHAENLQLVRNPSQFTRDSRIWTPLREGMVKPDHPLTPHIAWQPVCSH

AEGEEHKRLRGAVTAAMETIDHRGVRRHIGRYTQILVNSFCERGRAELVSQFAEHLPMAV

MCEILGMPEEYNDRMVQAARDALKGTETANQSHAYVMDALSRLTTRRRVRPEDDFTSHLL

THPAGLGDDEVREHLRVVLFAAYEATANLLANALRMVLTEPGFRAQLNGGQMTVPEAIEQ

SLWDEPPFSTVFGYYAKQDTELGGRQIRKGDGLLFAPAPGNVDPRVRPDLSASMQGNRAH

LAFGGGPHECPGQDIGRAIADVGVDALLTRLSDIQLDCAEEDLRWRSSIASRHLVALPVR

FEPKPQQDVDMPPRAMPLPPRRNDWQVGTLSGDPAPAAEPAPAPARASFVPAPTPPTPEP

PRPRGLWGRLLRWWRAE

>CYP158A11(SHJG_2646)shy

MIEEATRTGVEAMPPIRHWPALDLTGTDFDPVLAELMDEGPVTRVQLPNGEGWAWLVTRY

DDVRMVANDPRFSREAVVDRPVTRLAPHFIPDRGAVGFLDPPDHTRLRRSVAAAFTAKGV

ERVRDRARGMLDEMVDDLLAAGPPADLTAAVLGPFPIAVICELMGVPAGDRHGMHTWTQL

ILSAAHGKEVSERAKREMSSYFADLIGLRENSAGEDVTSLLGTAVGRREITLEQAVGLAV

LLQIGGEAVTANSGQMFHLLLTRPGLTARLRADPAIRPRAIDELLRYLPHRNAVGLSRIA

LEDVEIAGVRIRAGDPVYVSYLAANRDPDVFPAPDTVDFSRRPNPHVAFGFGPHYCPGGM

LARLEEELLMNALLDRVPGLRLAVPPDQVPFRKGALIRGPECLPVTW

>CYP158A12(SHJG_6732)shy

MEDLPALVPDPFLDVLAREEPVARIRLPYGEGCAWLVTRYEDVRFVTSDPRFSREQVVGR

AVTTMRPVPVASQTAGLQYIDPPRHTRLRQVVARAFTGRSMRRLRPLAERRAAGLLDAME

RAGAPADLMEHLHGPFPIAVLGDFLGVEEEDWRRWAATGEALLSAGAESGERAREAARAT

RARITELLRRRRADHRDDLAGVLARAAEAGEITDDEAVSLAIAVQVSGGHAVRSNSGSMM

YALLTHPGHLHRLRREPELLPRAVEELFRYVPHRNGVGIPRVATADVEVGGRLIRAGDVV

YNAYLAANRDPEVFPDPDALDFDRGNLAHVAFGHGPHHCLAAVMARMEAEVMIGAVLTRF

PGIRLAVPPEEVEFQRRGLIRGPRTLPVTW

>CYP159A2(SHJG_1326)shy

MSAAHHLPDILSPEFAANPYPAYAVMREKEPLIWHEATQSYIISRYEDVERVFKDKKAEF

TTDNYNWQLEPVHGKTILQLSGREHAVRRALVAPAFRGSDLEQKFLPVIERNSRELIDAF

RHTGSADIVNDYATRFPVNVIADMLGLDKADHARFHGWYTAVIAFLGNLSGDPEVAAAGE

RTRVEFAEYMLPVIRERRANPGDDLLSALCAAEVDGVRMSDEDIKAFCSLLLAAGGETTD

KAIAGILANLLSHPDQLAAVRADRSLIPAAFAETLRYTPPVQMIMRQSATDVEVTGGTIP

AGATVTCLIGAANRDERRYRDPDRFDIFRDDLATTSAFSAAAGHLAFALGRHFCVGALLA

KAEVEVGLNQLLDAMPDLRLADGHDLVEQGVFTRGPKTLPVRFTPVTA

>CYP163B6(SHJG_1395)shy

MTPPTTPVRTPLPPSALPGLDLSDPATHARHDLSEIWRTLRAEDPVHLHRPAGGGPAFWV

VTRFADATEVYRAPDRFTSARGNVLASLLQGGDSASGVMAAVTDGPRHRELRRVLLKAFS

PRALTGVVEKVEKAGHDLVAAVVGRPECDFAEDVAGHLPLKAICDLLDVPEQDRAFLLRL

TRTALSSDGPDQSPFDVWQARNDILAYFGELATSRRERPGTDAVSVLATAETEGRPLTMD

EVVANCYSLILGGDETSRLSMIGAVLAFIEHPAQWRAFKNGEVSVDAAVDEVLRWTTPAL

HFGRTATADTLLNGVSIASGDIVTVWNASANRDEAAFTDPDRFDLGRMPNKHLSFGHGPH

YCVGAYLGRAEIAGLLKALREQVDRMELTGRPEPIYSSMLSGFSSLPVRLVA

>CYP170A10(SHJG_6336)shy

MTVESVQPANPGTPGTPENPETPDLCEPPLAGGAVPVLGHGLKLVRDPLAFMSGLRDHGD

VVRLRLGPKTVYAVTAPALTGALALNPDFKIDGPLWESLEGLLGKEGVATANGPRHRRQR

RTIQPAFRLDAIPGYGPVMEEEAHALTDRWKPGGTIDCTSESFRVAVRIAARCLLRGDYM

DERAERLSLDLATVFRGMYRRMVVPLGPLYRLPFPANRAFNRALADLHLLVDEIVAERRA

SGQKPDDLLTALLEAKDDNGDPIGEQEIHDQVVAILTPGSETVASTIMWLLQVLAAHPEH

AEKVRTEVESVTGGRPVGFEHVRSLTHTNNVVVEAMRLRPAVWILTRRAVTDTALGGYRI

PAGADIVYSPYAIQRDARSYARHLDFDPDRWLPERAKEVPKYAMSPFSVGNRKCPSDHFS

MTQLSLITAAISAKYRFEQVSGSDDTTRVGITLRPQNLLLRAMPW

>CYP180A8(SHJG_7146)shy

MREAPPVPDVFDPRRYAAGVPHDDYRVLRDHHPVAWQEEPEVLGWPAGPGFWAVTRHADV

VRVLKDAETYSSYAGATQIRDPDPEDLPFIRRMMLNQDPPGHGRLRRLVSRAFTPGRVDR

FAAIARERARTLLAGALEAARAGDGTVDLVSAVTDEYALLNLADLLGVPESDRRLLLRWT

QQVIGYQDPDEAGPPVLDGAGRPVNPRSPAMLADMFAYAGQLAAYKRRYPADDIMTTLAH

DAELAEAELEMFFFLLTVAGNDTVRAAAPGGLLALAEHPEAYEPLRAGKAAVPSAVDELL

RWHPPVLTFRRTAVRDTELAGRPIRAGDKVVVFHASANRDERVFAAPDRLDLTRAPNPHV

SFGDGPHVCLGAHFARLQLRLLHEEVLRVLPGPPRLAGPPGRLVSNFINGIKSLPLHVT

>CYP180B1(SHJG_1790)shy

MTTTAQSVTDAVREPLPLADVDLANLDHFTDGVTPWRMFHTLRHEAPVHWQPEEAPNSGF

WSLTRHADIARVDRDAETFTSTRFVNLEEVDDDQIKKRASILELDGVRHRALRSLLQRQF

GASVINSYADFLRGLTATTLDAALAKGTFDFVKEVSADFPINVLARLLDVPPEDNQQLID

WGNRIIGNTDPDYADVLLHSEESEKYRDLPFRSPASLEVFAYGRELARQRRGGTGTDLIS

KLVNETPRDGVPLSPQDFDNYFLLLVVAGNETTRHTITHSMLALIQHPEQLARLQEDPSL

IPTAVEEFLRWASPVYHFRRTATRDVELGGKHIKEGDKVVMWFASGNRDEEVFGNPYDFD

VTRRNNDHITFGKGSPHLCLGNLLARTEIRIMFEELIPRLADIKLAGDVPRVRSNFVNGI

KKLPVEVTPA

>CYP285B1(SHJG_1853)shy

MRISTPHPGGPGTGIDLDTVDLFDPELYAVGDPHAIWTVMRERAPVHRQTLPDGRSFWSV

TKYHDVNDVLRDHTRFTSNRGTLLSILGGTDPAGGKMMAASDPPVHTAMREPLNKVLSHR

ALKSRQPQIRRVVHRLLAPLLDGGTWDVAAAGAGFPMAFTGTLMGLPEADWPRLTRLTTM

AVAPEDHDFRESAGDSTLTAAHHELFAYFSGQVKRRVRHGFAEDDLIGFLTELEAGGRRL

RHDEIVYNCYSLLLGANVTTPHAIAATVLALMEHPQEYRRLLADPSLTTGAVEEGLRWAS

PANHFMRYVTQDITLRGQELKAGDAVVAWLGSANRDEEVFPDPFRFDVTRSPNRHVAFGF

GPHYCIGAPLARIALRLLFEEVVALVEAFAPAGPVEHLTSNFVAGIKRMPLTATLREGAA

RTLAEAVAADGPVPA

>CYP1035A6(SHJG_2490)shy

MADPLPVWEELRALGDVVPAPWGGYFVTGFEACSQVLRGRNWLVPDFDWQERRPDPSRWR

EPATREMTRTLSRLNPPVHTFQRRALGNLFDRGTLEAMRPRIAGHVTRLLDRLAAQLRTH

GEADFVDTVGDRLPIHTVGQWLAIPAEDYPRILDFTHRQVHAQELLPTKTELAVSAQATL

EMRDFFTRLIAHRRRHLGNDVLSGWIRYWDAQYPEDRAAADQTLYDLTMFITIASLETTA

TLLTNAVWFLTRDPARADWLRRHPEHIDDAIDEVLRYDPPIHLNTRYAADDTVLAGVPVT

KDTAVHVLYGAANHDPRRNENPHVFDIRRKGAHLTFGGGAHYCLGMALARLEARVLLGEL

LERFPTLRPTADPAYASRMVFRRVTSLTVTA

>CYP1043A2(SHJG_8442)shy

MVSRRTAAALAAGGALLLSSPAWLPGKVVALRTKVFARVNGDEGMLLPDATFGPDRFQEV

YGHPAAGGRSKGAALSDLFWYWLAPGADVHQEHLEAGPRYDEVARRTLAILSGPSAELYD

AAARHTRRALDAAATGRVRTVRLRDLMMPVWAEFFYGLVFGEPCPAEARRLIVAHAEDVV

NALKCTRPRRMRRRNRLTRYLLRRIAAGDVPHTLPAGLTRRQQAYYLQGTFFNTAVVQMS

EAMAHLLLVLAQHPDVQERVAATPDDDRYLTHVMNETFRLYPLFGVAHRITTADIALDGL

PTIPAGSVLTFSYPDYHATGYADPERFDPGRWETLSAKNAHHIPFGVAANRPCPAWRLAP

VVMRAATREVLARYVLDSPVSHTRSIPHRAPCLLLPRDRPVPDRPRRVLRTALAVRDRAE

DVGRSLLQLLFGTWMVLDARRTRPAATYFARYDTEGRPLDGDHGSPGTAAGPGQAPATCP

YTGRSA

>CYP1060A1(SHJG_2545)shy

MLRRAEDFPKAPSPLFPPLKGSNRAPTAEERAHAHAARMRGLRAQAVAARVGEIAPGAAR

FAEQWPTGRDVEVLPAARHALAEIGVGYLFSEDAPALLPFAARLFLAREVLARPSRWEWP

RWVPTPARRFRTRQQAAFTEALRPIVRRRRASGRLGDDVLGQMLQPSARYGPLPEGAVLD

TLPGITVATFEAPTRAAGWILLHLARHPWAADRVAAEAASLPADPAATTSAHLDDLRYTQ

ALVREVLRLHPPSWLLTRQTARQTELADYTINAGSTVLVCPYTAHRDAREHAEPDEFRPE

RWLDDSGSPADPGAFLSFGTGPHGCEGAALAMAMLTLLTAHTARCHHLSEPPGPPGFQVT

TFEGLATAGLRLRATSRAPERSGLR

>CYP105B22(SHJG_0294)shy

MLADPRFSADPSRPGFPAPTEGFAAQGRQEMQALSMQDDPEHARQRRMLIGRFTIKQIAA

MTPRLEQIIDDLLDRMQAAGPPTDLVEAFALSMPSQVISELLGVPRRDHALFQRVAGTII

SRESTVREFTAARDELAGFLGDLIRRKDADPGDDLLSSLVVTRLRTGELTPALLVETAIT

LLVAGHETTTNQLALGTLVLLRNPDHLAVVRDSDDPARVASAVEELLRYLSITQNGLSRV

ATEDVEIAGRLICAGEGVIVPNASGNRDAAVFRDPDRFDIDRPDVRGHLAFGYGVHQCLG

QNLARKELQLAYPALWRRFPGLRTTLPDEDIRFKHDMIAYGVHELPVTW

>CYP125A32(SHJG_3828)shy

MTRHADVRHVSTHPELFSSSLNTAIIRFDEHIRSEATDAQRLILLNMDPPEHTRVRQIVQ

RGFTPRSIRALEDRLRARAEAIAAARARSGPFDFVTEVACELPLQAIAELIGVPQDDRSR

IFDRSNKP

>CYP147F11(SHJG_8814)shy

MESDLTATVNSLIDDFAGKERIDVVDDFACRFPVTVTCHLLGVPREEEPRFHVWVNDIMN

SVDYDPETDPREKLDKGVQARKDLRRSLGGLVEQRHGRSGDGLLARLANDDGPDGRMTDE

EIVATAKLLLIAGHETVVNLIANGMLTLLRHPQVLERLRHEPDLVAPLVEELLRYEPSVH

IIPWRAAYSDITVADTVIPKGSQIMLMLASGSRDPKRFHGPDRFDPDRRDNQHLGFGSGI

HSCFGAALARRETQIALTELVRRLHRPRLVADPPPYRPSPILRGPIHLDVEQGGD

>CYP105B22(2562371964)SHO

MLADPRFSADPSRPGFPAPTEGFAAQGRQEMQALSMQDDPEHARQRRMLIGRFTIKQIAA

MTPRLEQIIDDLLDRMQAAGPPTDLVEAFALSMPSQVISELLGVPRRDHALFQRVAGTII

SRESTVREFTAARDELAGFLGDLIRRKDADPGDDLLSSLVVTRLRTGELTPALLVETAIT

LLVAGHETTTNQLALGTLVLLRNPDHLAVVRDSDDPARVASAVEELLRYLSITQNGLSRV

ATEDVEIAGRLICAGEGVIVPNASGNRDAAVFRDPDRFDIDRPDVRGHLAFGYGVHQCLG

QNLARKELQLAYPALWRRFPGLRTTLPDEDIRFKHDMIAYGVHELPVTW

>CYP125A32(SHJGH_3593)sho

MTRHADVRHVSTHPELFSSSLNTAIIRFDEHIRSEATDAQRLILLNMDPPEHTRVRQIVQ

RGFTPRSIRALEDRLRARAEAIAAARARSGPFDFVTEVACELPLQAIAELIGVPQDDRSR

IFDRSNKP

>CYP107CH3(2562372021)SHO

MSAHEHCPYQDGRVVIDHAFKADSPARYARLRRLGPIHPAEFHLGLKGWVIVGHDLAREA

LTHPALLKDATPAAEALAAAGYVLHQPSVGLGAQMMEADPPEHTRLRRLASAAFTPRRTA

ELAPRIERIAHDLIDAMPPSGELDLVEAFNAPLPATVIAELLGIPPRYHLDFRRWSGQAL

QVASPEHRPALAALHGLLAGLVADKRRRPQDDLLSALVAVRDEEDGRLSEEELVGTAMML

VVAGHESTVHLLGNAVLALLRHPGQLRLLRERPELVPGAVEEFLRYDTSVERSTSRYASE

DLELGGVPIPRGSMVVVALGSAGHDAPQPDGDDPAVLDVTRSNARHLAFGHGIHHCLGAP

LARLETAIALRTLLSRVPELELAVPPDSLDWIGSGIIRGVLSLPVRYRVG

>CYP107CH3(2562372166)SHO

MDTHQQARPVGEPQERVIMSPEFKADAHRQYARLRAKGPVHPAQFFPGITGWVVVDYDLA

REALTHPALLKDPEPAAEKLEAAGFLGHKRGTGFGGQMLEADPPEHTRLRRLVSGAFSPK

RTAGMEPRITEIADRLVDAMPPSGELDLVEAFTAPLPVAVIAELLGIPEADRQDFRRWTT

LAFQVGHPEYASAVASLHGFLRGLADDKRRAPGDDLMSALVAARDEDDGRLSQDELAGTA

ALLVVAGHETTVNLLGNAVLALLQHPGQLRLLREDLGLLPDAIEEFLRYDTSVERTTNRY

AAEDLELGGVRIPRGGVVAVALASASRAAPLPGGGDPDVLDITRPAARHLSFGHGIHHCL

GAPLARLEARVALRTLLARVPHLELAVPADSLDWFPAGMVRGVLSLPVRYRRT

>CYP107X1(2562372487)SHO

MSNPDPTSPSEPAVDSAELFQDPYAVYGRLREEGPVHRITGTDGLPAWLVTRYDDVRRAL

ADPRLSLDKRNATPGGYRGMALPPALDANLLNMDPPDHTRIRRMVTKVFTPRHVEGLRVP

IRRAADRLLDSLAGQDEADLIPSYAAPLPITVICDLLGVPQAQRPDFRAWTDALVAPDPT

RPERAREAVGRLLSFFTRLIADKRARPADDLLSALIAVRDEEDRLSEDELMSLAFLILVA

GYENTVHLIGNAVAALLAHPDQLAALRADVGLLDRAVEELARYDGPVPLAIRRFPTEDIV

IGGATVPAGETVLLSLAAAHRDPRRFTDPDRLDLGRDATGHLALGHGIHYCLGAPLARME

TGIALTALFDRFPGLSLAVPPQDLRRRPSMRSRGLLALPVRTADRPGRTGRGASVG

>CYP159A1(2562372996)SHO

MSAAHHLPDILSPEFAANPYPAYAVMREKEPLIWHEATQSYIISRYEDVERVFKDKKAEF

TTDNYNWQLEPVHGKTILQLSGREHAVRRALVAPAFRGSDLEQKFLPVIERNSRELIDAF

RHTGSADIVNDYATRFPVNVIADMLGLDKADHARFHGWYTAVIAFLGNLSGDPEVAAAGE

RTRVEFAEYMLPVIRERRANPGDDLLSALCAAEVDGVRMSDEDIKAFCSLLLAAGGETTD

KAIAGILANLLSHPDQLAAVRADRSLIPAAFAETLRYTPPVQMIMRQSATDVEVTGGTIP

AGATVTCLIGAANRDERRYRDPDRFDIFRDDLATTSAFSAAAGHLAFALGRHFCVGALLA

KAEVEVGLNQLLDAMPDLRLADGHDLVEQGVFTRGPKTLPVRFTPVTA

>CYP157B15(2562372997)SHO

MSDTTGFPSTDAPPPGCPAHGSAVPLAGLEYQQTPSQLYRTLRREHGAVAPVLLDGGIPA

WLVLGYPEVCYVTAHDELFARDSRRWNQWEHIPPDWPLLPYVGYQPSVLFTEGAEHQRRA

GVITQALEGVDQFELARECQLIAARLISSFSGSGRAELMSMYAHALPARGVLWMCGMPAE

DTDTERLVDDLRISLDAGEGDDPVAAYTRVGERIMRLVKEKRERPGPDVTSRMILHPAGL

GDEEIVQDLISVIAAAQQPTANWICNTLRLLLTDERFAVNVAGGRVSVGEALNEVLWLDT

PTQNFIGRWAVRDTQLGGRHIREGDCLVLGLAAANTDPQIWPEPHAGSGNSAHLSFSNGE

HRCPYPAPLLADVMARTAVETLLEHLPDLVLAVEPEELTWRPSIWMRGLTSLPVEFTPAM

N

>CYP163B6(2562373065)SHO

MTPPTTPVRTPLPPSALPGLDLSDPATHARHDLSEIWRTLRAEDPVHLHRPAGGGPAFWV

VTRFADATEVYRAPDRFTSARGNVLASLLQGGDSASGVMAAVTDGPRHRELRRVLLKAFS

PRALTGVVEKVEKAGHDLVAAVVGRPECDFAEDVAGHLPLKAICDLLDVPEQDRAFLLRL

TRTALSSDGPDQSPFDVWQARNDILAYFGELATSRRERPGTDAVSVLATAETEGRPLTMD

EVVANCYSLILGGDETSRLSMIGAVLAFIEHPAQWRAFKNGEVSVDAAVDEVLRWTTPAL

HFGRTATADTLLNGVSIASGDIVTVWNASANRDEAAFTDPDRFDLGRMPNKHLSFGHGPH

YCVGAYLGRAEIAGLLKALREQVDRMELTGRPEPIYSSMLSGFSSLPVRLVA

>CYP180B1(2562373391)SHO

MTTTAQSVTDAVREPLPLADVDLANLDHFTDGVTPWRMFHTLRHEAPVHWQPEEAPNSGF

WSLTRHADIARVDRDAETFTSTRFVNLEEVDDDQIKKRASILELDGVRHRALRSLLQRQF

GASVINSYADFLRGLTATTLDAALAKGTFDFVKEVSADFPINVLARLLDVPPEDNQQLID

WGNRIIGNTDPDYADVLLHSEESEKYRDLPFRSPASLEVFAYGRELARQRRGGTGTDLIS

KLVNETPRDGVPLSPQDFDNYFLLLVVAGNETTRHTITHSMLALIQHPEQLARLQEDPSL

IPTAVEEFLRWASPVYHFRRTATRDVELGGKHIKEGDKVVMWFASGNRDEEVFGNPYDFD

VTRRNNDHITFGKGSPHLCLGNLLARTEIRIMFEELIPRLADIKLAGDVPRVRSNFVNGI

KKLPVEVTPA

>CYP147B5(2562373424)SHO

MASETLLARITDYASRPDPYPLYAELRAAGPVVQQADGSYLIGTYHEVAALLHDPRMSVD

PRTRGGEAQQLPFLRLDDPEHHRLRTLAMRPFGPPHSPRRVDGMRGEIDRITQELLASFR

AGEQIDLVDDFAYPLPVTVICRLLGVPREDEPLFRAWSDALVAAADVRPGADTTETDKAG

EQARMEMGGYLVNLAEQRRGKPSDDMLSAFVNEPDPALRLTREELAETAVLLLIAGHETT

VNLITNGVLTLLRRPDQLDLLRREPDLLPRAVEELLRFEPPVHMRERIPRADVDVAGTTL

PEGASVILVLASASRDPKRFDEPDRFDPTRPDNQHFGFGSGIHLCYGGPLARIEAYSALG

ALLPHLGTARLVQDPPPYRQNAMLRGPRHLPLQL

>CYP285B1(2562373454)SHO

MRISTPHPGGPGTGIDLDTVDLFDPELYAVGDPHAIWTVMRERAPVHRQTLPDGRSFWSV

TKYHDVNDVLRDHTRFTSNRGTLLSILGGTDPAGGKMMAASDPPVHTAMREPLNKVLSHR

ALKSRQPQIRRVVHRLLAPLLDGGTWDVAAAGAGFPMAFTGTLMGLPEADWPRLTRLTTM

AVAPEDHDFRESAGDSTLTAAHHELFAYFSGQVKRRVRHGFAEDDLIGFLTELEAGGRRL

RHDEIVYNCYSLLLGANVTTPHAIAATVLALMEHPQEYRRLLADPSLTTGAVEEGLRWAS

PANHFMRYVTQDITLRGQELKAGDAVVAWLGSANRDEEVFPDPFRFDVTRSPNRHVAFGF

GPHYCIGAPLARIALRLLFEEVVALVEAFAPAGPVEHLTSNFVAGIKRMPLTATLREGAA

RTLAEAVAADGPVPA

>CYP105AZ2(2562373518)SHO

MMTAPAEPADEAALPEFPMRRACPFSPPAAYAELRETEPVSRARLKVNGKPAWLVTRHDL

YKKLLGDSRVSANLKLPGYPLQVPVPQETLQSVPLTFLSMDPPDHTVQRRMLAPEFSVRR

MRELRGRVQQIVDEQIDQMLAKGADGPVDLVTALALPVPSLVICELLGVPYEDHGRFEEW

AWAIMNHDISDEDRGRAHYELDRYVDGLVTAKESEPGDDMISRLIEFNRRTPAVEHSDIV

SMSKLMLVTGHETTANMIALGVLALLEHPDQLAALRAEPELMPKAVEELLRLFSISDAGT

ARVALEDIELGGVTIRAGEGILPLNNAANHDERVFPDPDRLDVRREARSHLAFGYGVHQC

IGQNLARMELDVVYSTLLRRVPTLRLAAPVEELRFKDDAIVYGLYELPVTW

>CYP105AZ1(2562373519)SHO

MTQSADAAPETGSPLPRFPMRRTCPFSEPREYAGMRANAPVSRAALKVNGKPTWLVTRHE

DVRQVLGDSRVSSNLKLPGYPHQFHIPEEMLAQVRLMMLNMDPPEHSAQRRMLIPEFTAR

RVREMRPRIQQIVDEHVDAMLAQGGPVDLVTALALPVPSLVICELLGVPYEDHAQFEEWS

AAMMNHDLSPAEYGAAVQALDTYLDKLVTLKEDEPGDDLISRFLEKNRTEKVADHVDVVT

MARLMLVGGHETTANMIALGVLALLRHPEQMAALRDDPALLPNAIEELLRVFSISDSGTA

RVAVADIEVGGVTIRAGEGILALNNAANHDESVFPDPGTLDIRREEARSHLAFGYGIHQC

IGANLARVELETVYGTLLRRVPGLRLAAEPEELRFKDDAMVYGVYELPVTW

>CYP107F6(2562373867)SHO

MSSHDPAVLDCPFDFADALEYDPALDALARRGPVSRIRLPYGEAEAWLVTSFAGVRQVTC

DPRFSRAAIVGRDYPRMTPEPIVSPESINVTDPPHATRLRHVAAQAFTRERVAAMRPAVD

RVVAGLLTAMDEAGPPADLVTHLSVPLPHLTICELLAVPEADRDELRAHTMRLLATSPDA

RQDAADAKACLRTYFAERIPARRRSPGEDLLSTLATAPVPEGEEPLSDDELAVLAVTLIL

SGNDTATCQISDIAYLLLTRPEEMAALARDPGRFPGALEELLRFIPFRKGVGIPRIALED

AEIEGVPIRAGDYVHVSYLAANRDPDVFPDPHRLDLERPVRPHMTFGWGGHHCLAAPLAR

AELDSAVSGLLARFPKLRLDVPAEDIEWDNGTIRRFPLSLPVTW

>CYP154D1(2562373901)SHO

MDTSAARTPHRLDPAGGCPHAVNARLLARGAVAPVELPGGIEGMAVLGHEALKEFLQHPH

VAKNARHFTALREGRVGEGWPLLTFATVPGMTTADGDDHRRLRSLAARAFTPRRVAELRP

RVEELTESLLDGLARAAAAGDGVADLRRHFALPLPMGVICELLGVDVEFRDRLHRLGSLV

VATDTAPAEAVAANRDIVAVLGEIAAAKAARPGDDLTSALLAARDEGGDRLGEQELIGTL

LLMIIAGHETTLNLITNAVRALCAHPEQLGLVRAGRASWADVVEETLRWDAPVSYFPFRY

PVRDLTVAGTLVPAGTPVLAGYSAAGRDPAAHGPDADRFDLTRSARPGAVRHLSLGHGAH

YCLGAPLARLEATVALERLFTRFPSLRLAVPEAGLVPHASFVGNSVRALPVRLAAG

>CYP107AH2(2562373952)SHO

MTTTHEAVAAAERCTPEFRRDPHAVYAHLRDSAPVCPMRPPHGNETYLVTRYDDARAALS

DPRLSKDMYGAMDAYRRIFGDSSVALDDNMLNSDAPKHTRLRRLVNSAFTPRRVEALRPR

IEEIVRDLLDECPARERFDLLPAFAFPLPIIVICDLLGVPPEDRTRMQHLSTTVAQTGFG

EEAKRAQQQAEEGLHAYFTDLLTAKRERPGDDLLSALIAARDNDGGLTESELVSTAFLLM

FAGHKTTAYLIGNAVHHLLSHPAQLRAVREDPELIRAAVEELVRYDGSVESATFRYATED

VEYGGTLIPKGALVQIAISSANRDPRKFDAPDELDVRRPGNAQDAHLGFGHGSHYCLGAP

LARLETQLALTRLFERFPRMALADPAGAPRWLEVPFPAFRGLAELPVVLDPAG

>CYP1035A8(2562374092)SHO

MADPLPVWEELRALGDVVPAPWGGYFVTGFEACSQVLRGRNWLVPDFDWQERRPDPSRWR

EPATREMTRTLSRLNPPVHTFQRRALGNLFDRGTLEAMRPRIAGHVTRLLDRLAAQLRTH

GEADFVDTVGDRLPIHTVGQWLAIPAEDYPRILDFTHRQVHAQELLPTKTELAVSAQATL

EMRDFFTRLIAHRRRHLGNDVLSGWIRYWDAQYPEDRAAADQTLYDLTMFITIASLETTA

TLLTNAVWFLTRDPARADWLRRHPEHIDDAIDEVLRYDPPIHLNTRYAADDTVLAGVPVT

KDTAVHVLYGAANHDPRRNENPHVFDIRRKGAHLTFGGGAHYCLGMALARLEARVLLGEL

LERFPTLRPTADPAYASRMVFRRVTSLTVTA

>CYP156B11(2562374093)SHO

MHDAAFAADPHQVYDRLRAHGPAGPVELAPGVDATLVVGHEMALRVLQNSTLFARDARRW

KALNEGAIGLDSPVLPMMAYRPNCLFTDGAVHLRLRKAVTDSLARLNVTRIRRDVEPIAD

YLIDQFSERGRADLLNDYAKLLPLLLFNKLFGCPADIGDTLTSAMSAIFDGKDALRANEE

LTACLMELIALKRRRPGDDVTSWLIQHPAGLTDEELKDQLVMLMGAGVEPERNLIGNALL

LLLSPDTSGRDSGLLVEEAIDDVLWNETPIANYATHFPVQDVDLGGVVAEANTPVVISFA

AANSDPALAEARRKHSKGAHLAWGAGPHACPAKDPAQVIAVTAIEKILNALPDLTLAVPE

KELAWRPGPFHRALVALPVVFSTTPATRMVSALQNRTGSAAAEQPLPSVPASAASAGRQE

SARKKGFWSSFLDIFRV

>CYP1060A2(2562374146)SHO

MLRRAEDFPKAPSPLFPPLKGSNRAPTAEERAHAHAARMRGLRAQAVAARVGEIAPGAAR

FAEQWPTGRDVEVLPAARHALAEIGVGYLFSEDAPALLPFAARLFLAREVLARPSRWEWP

RWVPTPARRFRTRQQAAFTEALRPIVRRRRASGRLGDDVLGQMLQPSARYGPLPEGAVLD

TLPGITVATFEAPTRAAGWILLHLARHPWAADRVAAEAASLPADPAATTSAHLDDLRYTQ

ALVREVLRLHPPSWLLTRQTARQTELADYTINAGSTVLVCPYTAHRDAREHAEPDEFRPE

RWLDDSGSPADPGAFLSFGTGPHGCEGAALAMAMLTLLTAHTARCHHLSEPPGPPGFQVT

TFEGLATAGLRLRATSRAPERSGLR

>CYP158A14(2562374247)SHO

MIEEATRTGVEAMPPIRHWPALDLTGTDFDPVLAELMDEGPVTRVQLPNGEGWAWLVTRY

DDVRMVANDPRFSREAVVDRPVTRLAPHFIPDRGAVGFLDPPDHTRLRRSVAAAFTAKGV

ERVRDRARGMLDEMVDDLLAAGPPADLTAAVLGPFPIAVICELMGVPAGDRHGMHTWTQL

ILSAAHGKEVSERAKREMSSYFADLIGLRENSAGEDVTSLLGTAVGRREITLEQAVGLAV

LLQIGGEAVTANSGQMFHLLLTRPGLTARLRADPAIRPRAIDELLRYLPHRNAVGLSRIA

LEDVEIAGVRIRAGDPVYVSYLAANRDPDVFPAPDTVDFSRRPNPHVAFGFGPHYCPGGM

LARLEEELLMNALLDRVPGLRLAVPPDQVPFRKGALIRGPECLPVTW

>CYP147F13(2562374315)SHO

MPHASIARRITDYANRANPYPVYEELRRTPVLHEEEGGPYLISSYYDIKALLHDPRISSD

AANVAAAGDDELDQTEETGGLPPSFLRLDPPEHDRLRRIANSAFGPPHRPRRIENMRGEM

REIVTGLIDAFGDARQVDLVDQFAYPFPVTVICRLLGVPREDEPRFRTWVDPLVASLDPA

AGPDGDSDARKRAREARMQLGMYLAGLVEERTKEPRDDLLSDLAAGRGPHGSLSMMEVLS

TSVLLLIAGHETTVNLITNGMLTLLRHPEILQRLRADPGLSVNIVEELLRYEPPVQLVPQ

RTCITDIEVRGVTIPKGSRIWLVLAAGNRDPERFTDPQRFDPDRGDIQHLGFGSGIHSCF

GAPLARLEAQIALSELARRLEGPRLVEDPPPYRRNAVLRG

>CYP157C19(2562374673)SHO

MTPEFPSPTGTPDTLSGPPPGCPAHGLGPGSPRRLYGPDAEDLGDLYERLREEHGPVAPV

LLHDDVPMWVVLGHAENLQLVRNPSQFTRDSRIWTPLREGMVKPDHPLTPHIAWQPVCSH

AEGEEHKRLRGAVTAAMETIDHRGVRRHIGRYTQILVNSFCERGRAELVSQFAEHLPMAV

MCEILGMPEEYNDRMVQAARDALKGTETANQSHAYVMDALSRLTTRRRVRPEDDFTSHLL

THPAGLGDDEVREHLRVVLFAAYEATANLLANALRMVLTEPGFRAQLNGGQMTVPEAIEQ

SLWDEPPFSTVFGYYAKQDTELGGRQIRKGDGLLFAPAPGNVDPRVRPDLSASMQGNRAH

LAFGGGPHECPGQDIGRAIADVGVDALLTRLSDIQLDCAEEDLRWRSSIASRHLVALPVR

FEPKPQQDVDMPPRAMPLPPRRNDWQVGTLSGDPAPAAEPAPAPARASFVPAPTPPTPEP

PRPRGLWGRLLRWWRAE

>CYP125A31(2562375444)SHO

MTRHADVRHVSTHPELFSSSLNTAIIRFDEHIRSEATDAQRLILLNMDPPEHTRVRQIVQ

RGFTPRSIRALEDRLRARAEAIAAARARSGPFDFVTEVACELPLQAIAELIGVPQDDRSR

IFDRSNKP

>CYP107U1(2562376201)SHO

MTDQPHPAQPSSHRDQPAPALFTWEFATDPYPAYAWLREHAPVHRTRLPSGVEAWLVTRY

ADARQALADQRLSKNPAHHDEPAHAKGKTGIPGERKAELMTHLLNIDPPDHTRLRRLVSK

AFTPRRVAEFAPRVRELTDHLIDQFARRGSADLIHEFAFPLPIYAICDLLGVPREDQDDF

RDWAGMMIRHGGGPRGGVARSVKKMRGYLAELIHKKREALPAEPAPGEDLISGLIRASDH

GEHLTENEAAAMAFILLFAGFETTVNLIGNGTYALLTHPDQRRRLEESLARRETELLATG

VEELLRFDGPVELATWRFATEPLTIGGQRIAPGDPVLVVLAAADRDPERFADPDVLDLAR

RDNQHLGYGHGIHYCLGAPLARLEGQTALSTLLTRLPDLRLAADPAELRWRGGLIMRGLR

TLPVEFTPSA

>CYP107P2(2562377051)SHO

MAGLDDLAFDPWDPAFVADPYPAYAELRARGRVIRYEPTDQWLVPHHADVSALLRDRRLG

RTYQHRFGHEEFGRTPPPPEHEPFHTLNDHGMLDLEPPDHTRIRRLVSKAFTPRTVERLR

PYVEGLANDLVAALVGNGGGDLLKDVAEPLPVAVIAEMLGIPEADRGQLRPWSADICGMY

ELNPSEETAARAVRASVEFSDCLRELIAARREDPGDDLISGLIAAHDEGDRLTEQEMIST

AVLLLNAGHEATVNATVNGWWALFRNPDQLAALRADHSLIPSAIEELMRYDTPLQLFERW

VLDDIEIDGTTVPRGSEIAMLFGSANHDPAVFTDPARLDLTRKDNPHISFSAGIHYCIGA

PLARLELTASMTALLRQAPTLTPTEEPTRKPNFVIRGLEGLGVELG

>CYP170A10(2562378000)SHO

MTVESVQPANPGTPGTPENPETPDLCEPPLAGGAVPVLGHGLKLVRDPLAFMSGLRDHGD

VVRLRLGPKTVYAVTAPALTGALALNPDFKIDGPLWESLEGLLGKEGVATANGPRHRRQR

RTIQPAFRLDAIPGYGPVMEEEAHALTDRWKPGGTIDCTSESFRVAVRIAARCLLRGDYM

DERAERLSLDLATVFRGMYRRMVVPLGPLYRLPFPANRAFNRALADLHLLVDEIVAERRA

SGQKPDDLLTALLEAKDDNGDPIGEQEIHDQVVAILTPGSETVASTIMWLLQVLAAHPEH

AEKVRTEVESVTGGRPVGFEHVRSLTHTNNVVVEAMRLRPAVWILTRRAVTDTALGGYRI

PAGADIVYSPYAIQRDARSYARHLDFDPDRWLPERAKEVPKYAMSPFSVGNRKCPSDHFS

MTQLSLITAAISAKYRFEQVSGSDDTTRVGITLRPQNLLLRAMPW

>CYP158A18(2562378401)SHO

MPLDPTPAPSDPALAPADRGPGHGPTPPPPPIRFWPVEDLPALVPDPFLDVLAREEPVAR

IRLPYGEGCAWLVTRYEDVRFVTSDPRFSREQVVGRAVTTMRPVPVASQTAGLQYIDPPR

HTRLRQVVARAFTGRSMRRLRPLAERRAAGLLDAMERAGAPADLMEHLHGPFPIAVLGDF

LGVEEEDWRRWAATGEALLSAGAESGERAREAARATRARITELLRRRRADHRDDLAGVLA

RAAEAGEITDDEAVSLAIAVQVSGGHAVRSNSGSMMYALLTHPGHLHRLRREPELLPRAV

EELFRYVPHRNGVGIPRVATADVEVGGRLIRAGDVVYNAYLAANRDPEVFPDPDALDFDR

GNLAHVAFGHGPHHCLAAVMARMEAEVMIGAVLTRFPGIRLAVPPEEVEFQRRGLIRGPR

TLPVTW

>CYP180A6(2562378818)SHO

MSVREAPPVPDVFDPRRYAAGVPHDDYRVLRDHHPVAWQEEPEVLGWPAGPGFWAVTRHA

DVVRVLKDAETYSSYAGATQIRDPDPEDLPFIRRMMLNQDPPGHGRLRRLVSRAFTPGRV

DRFAAIARERARTLLAGALEAARAGDGTVDLVSAVTDEYALLNLADLLGVPESDRRLLLR

WTQQVIGYQDPDEAGPPVLDGAGRPVNPRSPAMLADMFAYAGQLAAYKRRYPADDIMTTL

AHDAELAEAELEMFFFLLTVAGNDTVRAAAPGGLLALAEHPEAYEPLRAGKAAVPSAVDE

LLRWHPPVLTFRRTAVRDTELAGRPIRAGDKVVVFHASANRDERVFAAPDRLDLTRAPNP

HVSFGDGPHVCLGAHFARLQLRLLHEEVLRVLPGPPRLAGPPGRLVSNFINGIKSLPLHV

T

>CYP107L31(2562378976)SHO

MIDLTEYGDALRRDPHPVYARLRERGPVHRVRLATPGGAWETWLVVGYEEARAALADQRL

AKDIARTGFVPLDEQLIGKYLLVADPPQHTRLRGLVARAFTMRRVERLRPRIRQITDELL

DDMLPRGHADLVDSLAYPLPITVICELLGVPEMDRAEFRKTSTEVVAPTGTDSERAATVR

LAEYLTELIEDKRCAGPTGDLLSDLIRTTAEDGDRLSMDELRGMAYLLLIAGHETTVNLI

GNAVLALLTHPDQLAALRADPSLLDGAVEETLRWEGPVETATYRFAAEPLEIAGTRIGRG

DDVLVGLTAAQRDGARFTDPDRFDIRRDTRGHLAFGHGIHYCLGAPLARLEAGIALGALL

DRAPGLALDGEPGEWLPGMLMRGVRSLPVRW

>CYP102B19(2562379724)SHO

MAATTETMETGLPKGFRSAEQGWPELRRIPRPPHRVPLLGDVLGVDRHRPLQDSMRLARE

LGPIFRRRAFGKEFVFVWGARLVADLADESRFAKHVGLGVANLRPVAGDGLFTAYNHEPN

WQLAHDVLAPGFSREAMEGYHGMMLSVADRLTDHWDRHLAAGRTVDVPGDMTRLTLETIA

RTGFGHDFGSFERDRPHPFVTAMVGTLAYAQRLNSVPGPLAPLLLRTAARRNAADIAHLN

RTVDGLVAARRRSGGGEGDLLDRMLATAHPRTGEKLSPENVRKQVITFLVAGHETTSGAL

SFALHHLARHPEIAARARAEVAQVWGDTPRPGYDQVARLRYVRRVLDESLRLWPTAPAFA

REARRDTVLAGDHPMRRGAWTLVLTPMLHREPEVWGEDAERFDPDRFTPAAVRARPPHTF

KPFGTGARACIGRQFALHEATLVLGLLLRRYDLHADPGYRLSVAERLTLMPEGLRLRLER

RPALTKADEGRPAGARGSVRGAADAAWARPARDAAADDRRHDPAGPAQPPSASRCPVHGA

AD

>CYP102G6(2562379734)SHO

MPPTALRTEPAGTLTGVPVVDITASGPGRTPIQQTMELMREHGPVLVRRLHGRDVMFVAD

ANLVADLADEERFAKHVGPALRNVREFAADGLFTAYNDEPNWAKAHDILMPAFALGSMRT

YHPVMLKVARRLIDAWDRAARAGRPVDVPDDMTRMTLDTIGLAGFDYDFGSFERAEPHPF

VESMVRCLEWSMTRLARTPGTDHSAADAAFRKDADHLARVVDDVIAARTGTDQSGAEDLL

GLMLSAPHPADGTTLDAANIRNQVITFLIAGHETTSGAMSFALYYLAKHPAVLRLVQREA

DALWGDTADPEPSYEDIGRLTHTRQVLNEALRLWPTAAAFSRHAREDTLLGGRIPLRAGQ

AVTVLAPMLHRQPVWGDNPELFDPERFTPEAEAARPVHAFKPFGTGERACIGRQFALHEA

TMLLALLVHRYRLHDHAGYRLTVKETLTLKPEGFTLTLTPRTPADRVHAPLPGAAAAPAA

GTPAPDALPARVRPGTRALFLHGSNYGTCRDFAARLADEAAAVGCATEVAALDAYKDGLP

TDRPVVIAAASYNGRPTDDAVAFTAWLDGTPDLTGVTYAVLGVGDRNWAATYQQVPTRID

ARLAELGATRLTERAAADASGDLTGAVREFTARVRSALLSAYGDPDAGHDTTDEPAHAYA

VRTLSGGPLDALAERHGLVPMTVTEARDLTAPGHPRTKRFLRVALPEGVTYRTADHLTVL

PANAPELVDRAVAAFGLDADAVLDIRPTRSRPWGSARTGEAERGGALAVDRPVTVRQLLT

HHVELQERPTSRQAALLAEANPCPPERAALAALPGDDPRTLVELAEDHPALRGALDWPLL

LDLLTPLRPRHYSISSAPAADPRHADLMVSVLDAPARSGKGRHRGTGSGHLASLRPGDTV

YARVQPCREAFRIDHTAPVVMVAAGTGLAPFRGAVADRLAARRTGARLPAGLLYFGCDAP

DVDYLHAEELRAAERAGAVSLRPAFSAAPVNGAAFVQHRIAAEAGEVWELLGAGARVYVC

GDGARMAPGVRAAFRTLYRERTPGADEAAADRWLDTLVADGRYVEDVYAAG

>CYP105D21(2562379764)SHO

MTELTDISAPAAPPGPVTFPQNRTCPYHPPTGYGPLRDGRPLSRVTLYDGREVWLVTGYS

AARALLADPRLSTERRRPGFPMPTPRFAAGRDRRVALLGVDDPEHHRQRRMLIPSFTVKR

AAALRPWIQRIVDELLDAMIAQGPPAELVSAFALPVPSMVICGLLGVPYADHEFFEEQSR

RLLRGPTAADTVQARGRLEDYLGGLIDAKAAEAEPGDGILDELVHDRLRTGELDRDDAVS

LAIILLVAGHETTANMISLGTYTLLRHPDRLAELRADPALLPAAVEELMRMLSIAEGLQR

VALEDIEVDGTTIRAGDGVLFGTSVINRDTSVYDDPDALDFHRPDRHHVAFGFGIHQCLG

QNLARAELEIALGSLFTRLPELRLAVPAEEIPFKPGDTVQGMLELPVTW

>CYP113K3(2562379804)SHO

MISKQPTGERLLLEELPDRWRGLREAGPVRYDETQGVWQVLDHETVAAVLADPATYSSDM

SALAPTQSDFETFRQGNFVGMDPPEHRKLRTLVSQAFTPRVVQGLGPRIEAVCARLLDAV

ADRDRFDLVDALAYPLPIIVIAELLGIPAEEHRLFQEWASVLFGGDQLGEAPDMADLERA

LEAIAPTVREMNGYMLDHIRARRADPGDDLTSRLIAAEVDGVRLADQEMVGFVALLLVAG

HITTTALLGNAVVTFDRHPGTNAALRAEPARIPAAVEEVLRWLPPFPELGRRVTRPVVLG

GHEIAPDTLLMAHLGAANRDPARFAAPDVFDVTRHPNPHLTFGHGIHFCFGAPLARLEAR

IALRMLHERFRMLAIPSYEDIAYQNPAVIIGVRHLPVEVRRP

>CYP154C1(2562379907)SHO

MTTGTEAPRIALDPFVTDLDAESAALRAAGPLAAVELPGGVPVWAVTHHAEAKALLTDPR

LVKDINVWGAWRRGEIPADWPLIGLANPGRSMLTVDGADHRRLRTLVAQALTPRRVERMR

ERIEKLTQDLLDALPADGGTVDLKAAFAYPLPMYVIADLMGIDEGLLPRLKVLFEKFFST

QTAPEEVVATLTELAQIMARTVSAKRAAPGDDLTSALILASEDGDRLTDEEIVSTLQLMV

AAGHETTISLIVNAVVNLSTHPEQRALVLSGRADWSAVVEETLRYSTPTSHVLIRFATED

VPVGDKVIPAGDALIVSYGAIGRDEGAHGPTAGEFDITRDTRNRHISFGHGPHVCPGAAL

SRLEAGVALPALYARFPGLDLAVPASELRNKPVVTQNDLFALPVRLNP

>CYP157A13(2562379908)SHO

MTTPDAVPLSGPRFQTEPARLYREMRRDHGAVVPVLLDGGIPAWLVLGYRELHQVTGDPV

LFSRDSDLWNQWENIPADWPLLPMIGHKQPSILYTVGERHRQRAAMISNALEEVDPFELR

AHAERFADELIDALCSAGEADLVAQYAMLLPVRVLARLYGFPDEDGPGLVTALNDMIDGR

ERALAGQAHLAASMAQLLADRRKEPAADVVSRMLADDSGFSDEEIAQDLMVMMAAGHQPT

ADWIGNSLRLMLTDDRFAASLFGGRNSVAEAMNEVLWEDTPTQNVAGRWAARDTHLGGRR

VKAGDLLLLGLQGANSDPQVRTDASALTGGNNAHFSFGHGEHRCPFPAQEIAEVIARTGI

EVVLDRLPDIDLAVPAESLTRRPSPWLRGLSGLPVRFSPVPAR

>CYP107BM6(2562380112)SHO

MSQQPVLLPYADPAFVADPFPLYRRLREEGPVRRAIIAGGLDAWLVTRYEDGLAALSDPR

LSSDVRDASDPRLIEQLPEFERESMLSTMLRSDPPDHTRLRRLVSKAFTARRVAGLRPRI

QEITDRLLDAVVPAGRAELVADFALPLPVTVISELLGVPVDDRYDFQRWTDAMLVRGEEM

PDPVVVDEAWHRMRAYLAKHLEAKRARPGDDLLSALINAHDHEQRLSHDELIAMTFLLLV

AGYITTVNLIAGGIVALLTHPGQLALLRERPELLPDAIEEFLRYDGPVSPGIARFAREDV

EIAGVTVPRGATVLIASAIADRDPARFTEPERLDITRRDNGHLAFGHGIHYCLGAPLARL

EGQVAVGTVLRRLPDLALAVPPAQLAWRPGGLRGPARLPVTFTPGGLG

>CYP1043A2(2562380120)SHO

MVSRRTAAALAAGGALLLSSPAWLPGKVVALRTKVFARVNGDEGMLLPDATFGPDRFQEV

YGHPAAGGRSKGAALSDLFWYWLAPGADVHQEHLEAGPRYDEVARRTLAILSGPSAELYD

AAARHTRRALDAAATGRVRTVRLRDLMMPVWAEFFYGLVFGEPCPAEARRLIVAHAEDVV

NALKCTRPRRMRRRNRLTRYLLRRIAAGDVPHTLPAGLTRRQQAYYLQGTFFNTAVVQMS

EAMAHLLLVLAQHPDVQERVAATPDDDRYLTHVMNETFRLYPLFGVAHRITTADIALDGL

PTIPAGSVLTFSYPDYHATGYADPERFDPGRWETLSAKNAHHIPFGVAANRPCPAWRLAP

VVMRAATREVLARYVLDSPVSHTRSIPHRAPCLLLPRDRPVPDRPRRVLRTALAVRDRAE

DVGRSLLQLLFGTWMVLDARRTRPAATYFARYDTEGRPLDGDHGSPGTAAGPGQAPATCP

YTGRSA

>CYP105AC8(2584328925)SLE

MGEPVHAVTMLPTARRPGCPFDPPEELTDARRHGPISRLTHFGGDPGWLITGHDLVRSVL

ADPRFSSRRELMNVVDYELPPAPPGEFLLMDEPQHGRYRKPLVGKFTVRRMRQLTERVEQ

ITAACLDAMEEAGPPADLVTSFAKPIPAIVICELLGVPYEDRGSFQEQIDAFVGGGTDED

ELMAAYTATQDYLAGLVAAKRAHPTDDVLSELTDSDLTGEELQGIALVLLVAGLDTTANM

LSLGTFALLENPAQLAALRADPALADRAVEELLRYLSVAKSFMRTALEDVELGGQTIEAG

TTVVLSYHTANRDPERFADPDTLDIRRQSTGHLAFGHGIHQCLGQQLARVELRVALPALI

GRFPTLRLAVPAEEVDLRPETADIYGVRSLPVTWDAEAS

>CYP102B20(2584329004)SLE

MAGTTKGPAGDGPPRGFRSAELGWPELHRIPRPPHRLPLLGDVLGAHGSTPVQDTLRYAR

QLGPIFRRRAFGKEFVFVWGAGLVADLADEARFAKHVGLGVANLRPVAGDGLFTAYNHEP

NWQLAHDVLAPGFSREAMAGYHRMMLDVAQRLTARWDRAQAAGRAVDVPGDMTRLTLETI

ARTGFGHDFGSFEHSRPHPFVTAMVGTLSYAQRLNTVPAPLAPWLLRRASRRNAADIACL

NRTVDDLIRARRTAPGQGDLLDRMLETAHPVTGERLSPENVRRQVITFLVAGHETTSGAL

SFALHHLSRQPEVAARARAEVDRVWGDTAEPAYEQVARLRYVRRVLDETLRLWPTAPAFA

REAREDTVLAGAHPMRRGAWALVLTPMLHRDPRVWGPDAERFDPGRFDAAAVRSRPPHTF

KPFGTGARACIGRQFALHEATLVLGLLLRRYELRPDPGCRLRVTERLTLMPEGLRLHLAR

RTAGVTPPPLEPAARDGGSAPRCPVRGAGD

>CYP107F10(2584329470)SLE

VEAAEAVWSCPFDFAEALEFDPLLRKLLEEHPVARITMPYGEGEAWLVTRYDDVRTVTTD

RRFSRKAVTGRDFPRMTPEPIVQSEAINLMDPPAVTRLRGLVAKGFTAGQVERMRGRTER

VVDDLLTAMAAHDAPDLFAHLASPLPMHTICEVLDIPEPDRKRLRHNALTMMNIGAAGKE

AAVRAKAELRAYFTELTAERRRAPGQDLISSLATARVGDDLLDDRELAVMAMVLLITGQD

TTTYEIANLGYLLLTRDDLLSTLRDRPELLPRALDEMLRHIPFRKGVGIPRVATEDVELG

GVTIRAGDVVHVSYLTANRDAEKFPNPHDIDLTRPAVPHMTFGWGSHHCLGAPLAEMEMR

VALQALLTRFPGLRLAVPPEDIRWNTTSIWRHPLALPVTW

>CYP199A13(2584329695)SLE

MPGAPAAVTPPTCDADPFAAEHLEHPEPLHRSLREAGPVVHLSRYDVHALARHREVHAAL

VDWQTFRSGAGVGLADFRREKPWRPPSLLLEADPPHHDAPRRVLREILSPPALRRLRAVW

QRAAEDLVDTLLATHGTEFDAVGALAEAFPLRVFPDAVGLGPDGRENLLPYGTMAFNAFG

PRNGLVAADAHRAAELSAWVNAQCVREALDEDGFGARIWAAADRGEITHEQAPLVVRSLL

TAGVDTTVHGLAATLYAFAAHPGQWRRLRERPELARVAFDEAVRWQSPVQTFFRTATTDV

TIAGTVVPEGSKILMFLGAANRDPARWPDPDRFDLGRDPSGHVGFGMGIHQCVGQHVARL

EAEALLTALARRVDHIEPADEPRRRLNNTLRSWASLPVRVRPAA

>CYP105B39(2584329708)SLE

MPETLTHSSEGQAEWPMPRAATCPFDPPPALKSLQSEAPISRVRIWDGSTPWLITRYEHT

RALLGDPRISSDPTRPGFPRTVGSAQSDTRAPLSFINMDDPEHARLRRMVTAPFAIKRIE

ALRPAVQRIVDDSIDTMLAGPKPVDLVEAVALPIPSLVICELLGVPYEDHDLFQHNTKVL

VKLSTAREESAAALAALTDYLERLLTAKLERPGNDLLSDIATRRVATGELTQREAARMGV

LLLIAGHETTANMIALGTCALLQNPDQLALLRDAPDAKTTASAVEELLRYLNITHNGRRR

VALEDIEIGGETIRAGDGLIIAGDIANRDPDVFPDPDRLDITRDARRHVAFGFGVHQCLG

QPLARLELQVVYSTLYRRIPTLALAIEPDQVRYKHDGAVYGVYELPVTW

>CYP105BR1(2584329859)SLE

LEANLPTSYPVPRTCPLDPAPVFGSLRENRRLHRVRLDFDGSEVWLVTRHSDARAVLSDE

RFSSDFSREGFPARMTVQPPGPGTFIRMDPPEHSRLRRAVVGEFKRKRVEALRPAVQTIV

DDLIDSMLKSGSPADLVQCVALPLPTLVICELLGVPYADRHFFQECTGVIGDHSATPARR

QVVRDELRAYIDRLVGRKTEHPEDDLLSRVAAERDRDHLTHDEIVGIATLLMIAGFETIA

NQIGVGTLTLLRHPEQIRALLADPSLVPGVVEETLRHQTVIDYGLRRVAVQDVEIAGQTI

RAGEGVVVVLASANRDEDAFPDPDRLDPRREANEHLAFGHGLHQCVGQLLARLQLSVLWG

TLFTRVPTLRTAEPLEEIPFRTDMFVHGVHALPVTW

>CYP107EG1(2584329860)SLE

VAQLDEEAPRPADAGGTPPSFPFPITSALDPSPEYARLRAEKPVSRVTLPSGDTAWIVTR

YEDVRFVFNDARFSRQAATRPEAPKLMPGVEGDPDSIVSKDAPDHTRLRRLVAPAFTVRR

IEGMREGIQSTVDGLLDAMEEAGKPGDVVSSLAGPLPIITICDLLGVPPADRDRFREWSD

TMFRTSPDELETAVAARNALIGYLAAMVQQRRAKPADDLLGVLIAARDNDDRLSERELIS

FAGGLIVAGYETTANRLANAVLVLLRNPDQLELLRADPELIADAVEELLRFIPGGAAGGL

MRVAVEDVEVGGELIRAGDGVIAITNSANRDESVWDSPDRFDITRKPGSHTSFGHGIHHC

VGAQLARLELQIGIGTLLRRFPGLRLAVPEAELPWKKNVVIHALEALPVTW

>CYP107U1(2584331141)SLE

SPAQAGPAPALFTWEFAADPYPAYAWLREHAPVHRTTLPSGVEAWLVTRYADARQALADN

RLSKNPAHHAEPAHAKGKTGIPGERKAELMTHLLNIDPPDHTRLRRLVSKAFTPRRVAEF

APRVQELADRLIDRFAPGGEADLIHEFAFPLPIYAICDMLGVPREDQDDFRDWAGMMIRH

QGGPRGGVARSVKKMRGYLADLIHRKREALPPEPGPGEDLISGLIRASDHGEHLTENEAA

AMAFILLFAGFETTVNLIGNGTYALLTHPGQRARLQQSLAAGDRGLLETGVEELLRYDGP

VELATWRFATEPLTIGGQHIAPGDPVLVVLAAADRDPERFADPDTLDLARRDNQHLGYGH

GIHYCLGAPLARLEGQTALATLLTRLPDLRLAADPADLRWRGGLIMRGLRTLPVTFTPAP

>CYP170A17(2584331404)SLE

MTVGSSPESPAPSGQEAPPSSDTRPQAELREPPVAGGALPFLGHGWKLARDPLAFLSQLR

DHGDVVRLELGPKTVYAVTTPALTGAVALSPDYIIAGPLWESLESLLGKEGVATANGPLH

RRQRRTIQPAFRLDAIPAYGPIMAEEAQALVERWRSGEVLDITAESFRVAVRVSARCLMR

GSYMDDRADRICSALATLFSGMYQRMVLPLGPLYRIPVPANLEFNRALADLHLLVDEIVA

DRRASGQKPDDLLTALLEAKDDNGEPIGEQEIHDQVIAILTPGSETVGSMIMSLLLVLTE

HPELGDKIRDEVKSVVGDRPVAFEDARKLTFTANVIVETMRLYPAVWILTRRAVTDTELG

GYRIPAGADLVYSPYAIQRDARSYERHEEFDPDRWLPERSKDVPKYAMSPFSVGNRKCPS

DHFSMAELTLITAAIAAAFRFEQAPGSDPRPRIGITLRPRRLLVRALPR

>CYP1418A1(2584331691)SLE

VNLSSTGTVTYPFDDGKEKALAPYDPELRASSGLAEVRMSFGDPAWLVTRNVEVRTALMD

QRLRSREPSMDPGAPRMHPDTPSIPVNIAALSGPEHVCGRKVMAKSFTARRVPEFQPRTT

ESSRGLVDRLVGNGPVADFVDVFALSLPLHVICDMLGVPAGDVGQIMEWSDAFLEMKFTE

PAPQQVGEYVTAFTEYLGGIIAARLVTLGEGVMGEMAEAVAAGAATLPEAAMLALGLMVT

GLENSSIALANFVYVLDRTDQRRALCDNQHLVPTTVEELLRFLPLSAGAVLAGQATEDVA

VDDHLVRAGETVILAYGAANRDEAVFHQPAEAELDREANTLTELTTRMPELRVHAADGEL

AWLITRQSRGFAELVPRQATFARSRRPARPLATPAGSPSTSSTRTSGRHTESTHRTPLLD

GQTLPAAALAVSW

>CYP107L33(2584331924)SLE

MPEVIDLGGYGDAFRTDPHPVYAELRARGPVHRVRLPAPDDAHPTWLVVGYEEARAALAD

PRLAKDPGGIGATFLDEELIGKHLLLADPPRHTRLRGLVSRAFTARRVRELAPRVRHITD

DLLDTMLPHGRADLVESFAYPLPISVICELLGVPETDRAAFRKMSTEVVAPTSPDSERDA

VERFAAYLTGLIEDKRCAGPGGDLLSDLIRTTADDGDRLSPEELRGMAYLLLIAGHETTV

NLITNAVLTLLRHPGQLAAVRADPGLADAVVEETLRYEGPVENATFRYAAEPLEIGGVPV

ATGETVMIGLTAADRDPGRYPGPDRFDIHRDTRGHLAFGHGVHYCLGAPLARLEARTAIR

SLLERAPGLSLDGPPGDWLPGTLIRGVRSLPVRW

>CYP1031A3(2584331962)SLE

MTTETPSEPLIFNPFTPDFMNDPNPHYAELRRHVPVHEHPGGFWMLSRYEDASALMRSGL

SVEQRHVAPGPFRDAYAKAGVTDQPRPKGLALLDRDAPDHTRLRKLVTMAFTARAVNAME

GEIRSLVDEALDRIAADGGGDLVEALAFPLPFTVISRMLGMPPTDTVRMRTLTHTLMRSV

EPTTDPEVMRAVEAADAELFDIVGEAVDWKRQNPADDLLTALITAEDHGDVLSRDELIAQ

VTMLYVAGHETTVNLISGGTLALLRNPGQLRLLRDKPELEQNAIEELLRYDAPVHNSRRI

TAEPYEVGGHEIPPGSFILANLAGANRDESYFGPDAEELRLDRENARRHVSFGGGMHLCL

GAALARIEGRVAIGGLVRRFPGLELAGEVEWNGLLSLRGAARLPVRV

>CYP107P17(2584332128)SLE

MAAFDPWDPAFVADPYPAYAELRARGRVHHYEPTGQWLVPHHADVSALLRDRRLGRTYLH

RFTHEEFGRTPPPPEHEPFHTLNDHGMLDLEPPDHTRIRRLVSKAFTPRTVERLVPYVRG

LADELVSGLVAAGGGDLLTDVAEPLPVAVIAEMLGIPEADRAALRPWSADICGMYELNPS

EETARRAVRASVEFSEYLRELIAARREKPGEDLISGLIAAHDEGDRLTEQEMISTCVLLL

NAGHEATVNATVNGWWALLRHPAQLAALRADHSLVPTAVEELLRYDTPLQLFERWVLEDI

EIDGTVIPRGAELALLFGSANHDPAVFTDPGRLDLTRADNPHISFSAGIHYCIGAPLARI

ELAASMTALLEKAPALALAAEPERKPNFVIRGPAGLPVSL

>CYP105B36(2584332408)SLE

MPTPAPFPPLPVEPPSGCPFDPPEGLARLRLEEPLSKVALDDGSWAWLATRYADVRAILG

DQRFSSDTSTPGYPVSGMTGGSPRPDAARGFIRMDPPEHTRLRRMVTRDFMVKRVEALRP

TLQRLTDELCDEMERVDRSEHPVDLVRALALPLPSLAISLLLGVPYEDHDTFQRLTGTLL

SRETGEEDRGPARAELLAYIDGLVRAKVAEPGDDIISRLATEQHARGELTHEDLIAFAVL

LLVAGHETTANMIGLSALSLMLDPETAGRLREDPSLVRGAVEELLRFHSIIRNGPRRAAL

EDVEVGGRLIRKGEGVIVAVPSANRDEDVFPDAGRLDITRPNAQHHVAFGYGIHQCLGQA

LARAELQIVITTLLRRFPTMRPAVPVEEIPFRTDMVIYGCHALPVTW

>CYP157K4(2584332933)SLE

MNDQTTPGAAGGCPVAHGGAPRLYGPEAATDPQGLYSRLRKQYGVVAPVLLEGDVPAWLV

LGYRENRRVLDNPLQFSRDSRIWRDWREGRVDESSPLIPMVGWRPDCVSQDGEPHQRLRA

AVTDNLNAVAGRGIRRHATHYAHKQIDAFAGAGRADLVSDFAEYLPMLVLTRVFGLAEAE

GRRLAESSNLVIKGGADALAHNERIMGILGELTARKRAEPGSDFTTGLIEHHAGLDEEEI

VNHLRLVLITAHTMTSNLLARALQLVLTDTSWLSGLVSGQLDISTVVEEVMWNRPPLAVL

PGRFATADLELGGCPIKKGDLLVLGLAAGNSDPDIRPDADVSVQGNQSHLAFSAGPHECP

GQNIGQAIIETAVDVLLHRLPGLRLAVPPEELTSTASTWEDRLDSLPVEFTAA

>CYP159A1(2584333094)SLE

MSTVQQVPDILSPEFAADPYPAYRVMRDSAPLIWHEATQSYIISRYEDVERVFKDKNGEF

TTDNYDWQIEPVHGKTILQLSGREHAVRRALVAPAFRGSDLQEKFLPVIERNSRELIDAF

RHTGSADLVADYATRFPVNVIADMLGLDKADHGRFHRWYTSVIAFLGNLSGDPEVAAAGE

RTRVEFAEYMFPIIRERREKPGDDLLSTLCAAEVDGVRMSDEDIKSFCSLLLAAGGETTD

KAIAGIFANLLRHPDQLAAVREDRGLIARAFAETLRYTPPVHMIMRQSATEVTLSGGTIP

AGATVTCLIGAANRDENRYRDPDRFDIFRDDLTTTTAFSAAADHLAFALGRHFCVGALLA

KAEVEIGVGQLLDAMPDVRLADGFQPAEQGVFTRGPQAVPVRFTPVAP

>CYP157B15(2584333095)SLE

VTDTGSLAAGAAPAGCPAHAGAVRLAGLEYQQTPSQLYRALRREHGAVAPVLLDGDIPAW

LVLGYSELTYVTSHDELFARDSRRWNQWENIPADWPLLPFVGYQPSVLFTEGEEHRRRAG

VITQALEGVDQFELARDCEQIADRLIASFAGSGEAELMSAYAHALPMRAVVQMCGMPHSG

TDTQQLVDDLRISLDAAEGDDPVAAYTRVAERIHQLVKEKRERPGPDITSRMLQHPAGLA

DEEIVQDLISVIAAAQQPTANWICNTLRLLLTDERFALNVSGGRLSVGEALNEVLWLDTP

TQNFIGRWAVRDTQLGGRLIRAGDCLVLGLAAANTDPQIWPESHVGAENAAHLSFSNGEH

RCPYPAPLLADVMARTAVETLLERLPDLVLAVEPEQLTWRPSIWMRGLTSLPVRFTPVVQ

>CYP183J2(2584333126)SLE

MIESKGAFVTLVAPPPIPRAAGSLPLLGHAVQLMRDNLGFIASLRRDYGPLVEITLQPGT

RTVIVQDPELIRAMLVDLGPSLDKGRFFEKMGQLLGDSVVTAAGQEHVRKRRQLQPAFAR

GEIARYVDIMRDEVSTALDGWRLGQSLDVREAMVKLSLDMLAKTVFAGSLDEATFRRLRR

DLSVVMNGVGARIMLPDWAEKLPLPFNRRFDQARDAVRATIQRAVDDLQASGHDTGDMLS

MLLRATDEETGRPLTGDQICSEILTLAVAGTETTASVLSWTLYELSRHREVEARVLAELD

EVLQGRPVSFEDVTRLPYLRRVLDEVLRLHHTGWLVTRRTVTDTRLGPWTLPAGTELAYC

QHALHRDPALFRDPEVFDPDRWLDSEEPPPSGAFLPFGAGKHKCIGDRFALTELITAIAT

IVRRVRFDLRGQSVRPVARATVRPQALLMTVRRREETAGQAGTPGANSL

>CYP113K3_ortholog(2584333406)SLE

MATGGALPRAHGIRVRPVRDGRARAPPKGEKVTSNQLTEPRLPFDALADRWRSVCEAGPV

RYDERQGVWQVVDHHGVSTVLGDPATYSSDMSPIAPAQEDFDAFRQGNFVGMDPPQHRKL

RTLVSQAFTPRTVQGLAPRIEAVATRLLDGVADRDRFDLVDTLAYPLPIIVIAELLGVPV

EDHPLIQEWASTLFSGEQLGETPDMADLERALEAIAPTVREMNGYVLDHIRRRRADPGDD

LTSRLLGAEVDGVRLTDQEIVGFVALLLVAGHITTTALLGNAVVAFDRQPGTFDALRADP

GRIPDALEEVLRWLPPFPELGRRTTRRVTLGGHEIPADTLVMAHLGAANRDPVRFPDPDT

FDVTRTPNPHLTFGHGIHFCFGAHLARLEARIGTRLLLERFRSLGIPSYDDVAYQNPAVI

VGVRHLPVEVART

>CYP161C4(2584333611)SLE

MTELPRLPFDNPDIIGIAPQMFALQKEGPIARVRTAGEDAWLVTRYDEVRTLLADRRLAL

SNPYPERTTKSAARAFMVALMAGDDYHTEAPRHAQMRALLVPRFSTRRMRLMKSRIEQHV

DELLDELAASTPPVDLHRALSFPLPTMVVCDLLGVPLADRERFGQWARGTFDQSDNQHSA

NTFQQVVDYMTELVARKRIEPGDDILSELIADKDHALSDAEIAHLGNAVLLFGYETTIVR

IDLGTLLLLRNPAQRALLVEKPELAPAAVEEILRLGVGGNGSNAIIPRYAHSDITVGDTV

IRAGDAVMLAIGAANYDERAYPGADLFDLTREKPKSHMAFGHGARHCIGRTLARIELTAV

FERLFRRLPDLRLAVPEETLRWQEHRITGGFDEIPVTF

>CYP183A4(2584333619)SLE

MSEQTTFVAGAAPGAVPVVGHALQMMRHPVNFMTSLSAHGDLVQIKIGPTSAYVPTHPDL

LRYVLTNDRIFDKGGVFYDRARDIAGNGLVTCPFADHRRQRRLMQSAFTRTQLKRYATAM

HAEIEATTARWHDGMVVDAFPELYGMALRTVGRTLYSTPVSPELAAGVERAFDVVLNGLF

RQMFLPRFIRRLPLPANRRYESNLRFLHRTTQELIDDYRSDGAAHDDLLAALLASRDDDG

GRLDDKEIHDQVITVMAAGTETVAGTLTWVFYLLSQHPEIEAALYEEIDTVLGGRAPDWD

DLPNLSLADRIISEALRLHPPAWLFTRLTATETELAGRRLPEGTTIVFSPAAVAQYEDAF

GNPTAFDPDRWLPDRVSPAARQAFMPFGTGARKCIGDLYARTEATLGLATILGRWRVTCE

PDMDVRPVPLATVYHPRRLRLRLSARTPRPAATPVPAPAGGDPA

>CYP105BT1(2584333841)SLE

MANPAYPMARQCPMAPPPAYTTLRGQGPTKVDLPDGGWAWLITSYDDVRQAMNDPRLSSD

DTKMARARTQLPPNENLNSFWRTDEPEHGRLRHMMMTEFTAHRIKEWRPRIQALVDELLD

HLEELPRPVDLYSEFALALPTQVIAQLLGVPQKDYRQFAQQSRTILSLDRPEESWAAYYE

MNDYLNRLIEEREREPADDLISRLIVDRVKTGELDRDELLPMVRFILVSGFETTTSQIAL

SALTLMTNPEVRRQLIEEPERITAFVEESLRFWSVSQDNILRVVDQDMEFAGTAMSVGEL

VVLAIPSANHDERAFPDPERFDLDRGDNRHVAFGFGTHLCAGASLARREVDIAITSLLAR

FPDMRLAVDVGDLTFRQKSLVYGLENLPVTW

>CYP107L35(2584333842)SLE

VPATTTVDLLRLSPDFVRDPYPVYAALRAQAPVHRVRTPDGPEIYLVLGHETCRAALTDP

RLSRDWRGSGRLRQIINADEDDPNLAHMSMAEPPDHTRLRRLVTREFTPRRIDTLAPRMQ

QITDRLLDAMTADGARRADLMDSFAFPLPMTVICELLGVPELDRQSFRRWSNEMLAPTSP

QAQGAAYADLGAYLPRLIGVKRTEPGEDLLSALIHTVDEEGDRLSPAELVGMCNLLLIAG

HETVVNLIGNGMRALFAHPDQLRLLRGNPGLIDGAVEEMLRYDGPVETSLERLALTDVEL

GGVLIPAGSTVRMVLADADRDTARFAQPDRFDIRRDTRGHLAFGHGLHHCLGAALARVEG

RIAIRSLLERCPHITADTGADTLAWVPGLLVRGVRKLPVRW

>CYP102G7(2584333916)SLE

MPPTAPHPRPATGTPEVPVVDISATGPGSTPIQQVMGLMREHGPVLVRRLHGRDTLFVAD

LGLVTDLADDARFAKHIGPALENVREFAADGLFTAYNDEPNWAKAHDILMPAFALGSMRT

YHPVMLTVARRLVDHWDRAARAGQPVNVPDDMTRMTLDTIGLAGFGYDFGSFARDEPHPF

VASMVRCLEWSMTRLARTPGQDHTAADAAFRADAAHLARVVDDVIASRTGTDQRAARDLL

GLMLTAEHPADGTTLDAANIRNQVITFLIAGHETTSGAMSFALYYLAKHPAVLQLVQREV

DELWGDQADPEPTYDEVGRLTYTRQVLNEALRLWPTAAAFSRHALEDTLLGGRVPLRAGQ

GVTVLAPMLHRQPVWGDNPELFDPSRFTAEAEAARPVHAFKPFGTGERACIGRQFALHEA

TMLLAMLVHRYRLHDHAGYRLTVKETLTLKPEGFTLTLTPRTPADRAHPALPGAAAAEGA

>CYP154A16(2584334045)SLE

VSEQPILVLDPTGSDHHAEHLALRAHGSLARVDILGVQAWAVTDPALLKRLLTSPDVSKD

PRAHWPAFAETVTRWPLALWVAAENMFTAYGSDHRRLRRMIAPAFSARRVAAMKEAIDRM

VATLLDILAALPAGEPVDLREHLAYPLPIAVIGHLMGVPEEQRDALRGLVDNVFDTTLSP

AEAQANATLLYERLDQLIATKRRFPGDDMTSQLIAARDDETDGSGLTQDELRDTLLLMIS

AGYETTVNVIDQSIHTLLTRPDQLRLVRTGAIGWNDVVEETLRHEPAVKHLPLRYAVNDI

PLPDGRTIARGEAILASYAAANRHPDWHHDPDTFDAARAHQEHLAFGHGVHYCLGAPLAR

LEVATALEHLFDRFPGIRLAVPASELRPVPSLISNGHQTLPVHLHGPLADGPSTA

>CYP2266A2(2584334378)SLE

VFLDRKSLWPHLLRWEKLAADADGPFRLRFGTLFIADANAARQVLVESADSYVEQSGFFR

IGPTPLPREPRIQASRELIKVLAHHGPNSSFDIDRAVHELRDGRGRLRHQRWGAELIRRY

FAPVIAHERHAEINSLVDVYVTSSIVGDDIVGHAIRGPHRSVPRIRAGFAEQLKRLPAGG

TGANDLVDVVLSLRGELSPADRAQLLQRLVLSAVGFTGVALEWVVLLGIQHGYNSPAVRP

EHIRSLVRETLRLYPTAWRLVRVAAADHDMDGIWVRKGEHVLIGTHAIHRTGAVWDDPLH

FLPSRWEKLTDDQRKSYLPFGKGGAMCPANGFALKALEHLGYLILHDFEGDVRLRRRKPH

VRTLLAPPAGWTRLTRKRTTSGSNGR

>CYP1416A1(2584334380)SLE

MTSSPHVRPSQGAAPLPASGCPVHSEPVRLNSEAVQGSLADLYEPWRSQYGPVVPVELDG

GVPAFLVIGHRTLREVCSQESLYSPDSRNWADWRAGRVPNDWPLLPQVAYQEGSTRFLSG

PEHRRLRGVLASGLAQVEAASARRYTEWVADRLISRFAPKGRTEVVADYAAPLPLLVMLR

LLGLPHEAGEQLLPAIFRLLEGGPGAHRANEEISDIIGRLVVARRAKPDRDLISWLIHGP

VDGGPALSDLEVRNLAWLTVMAGAGGTTGWIGNAMERLVCDEKVHTLFLAGKVTIAEIMN

ETHWSNPAVQNVMGRYPLHEVRLGNYHVPAGALLVLGLAAANADPEVHVDDSGHTFTNES

HLAFGSGPHECPTAAQRLAKIIGQTAVERFLARCRTPRLRDDGAVQHGGSVIVRQLTKLA

VIFTADTRTAVRHTASGTDALGESSRSSHYLFPPRMLTQLTSHITA

>CYP154Z1(2584334381)SLE

MAVTGRRLTDDTPAPHELALACPVYRLDPLGQDFPAEGRALQAIGPIVPVELPDQVAAWA

VTRRQVADALLTHPDMRKNPRHWRAYQAGLVPETWPLLQIITTPTMLIMDGADHTRLRLP

MQRAFTPRRVEALRPRVEEIVRGLLDTLAAVAPGTSVDLRSTFAFQLPVTVICELYGVDE

PEVRRRLATDTSALLSSTTPPGERLGAQTSVFGTMAQLIAAKRARPGDDLTTALIAEFDN

GGMSAEELAGTLFLMLIAGHETTQNLLSNAIQRLVENPEQLARVLSGHDDEDAWRGVVEE

ALRLDAPAATTMFLYAVRDITIEGVTIGAGEPVLIYTSAVGRDGHVFADPDAFLPDRANA

HQHRAFGHGPHHCLGAPLARLESRIALRGLFERFSLTAAEPLDAIERITSLSSNAPARVP

VYLTPR

>CYP155A8(2584334599)SLE

VYDRGSTARKVDRGRERPGCPVTRAEDGTWRVHDHAVARALLRGPGTVQAGLGVETVEKL

PRRIRRPVLYRDGPEHREHRRQTARFFTPRRVDEHYRDLMVRIAEEQLASLRAAGEARLA

DLAFELAIGVVSEVIGLRYGRPGIRRRLERFFPEEFGEPGLTSVRGLYWLVRQNANWLRI

HLADVRPAIRAHRRREHDDLISHLIGEGCSDAEILGECLTFAAAGMVTTREFVSLAAWHL

FTDAGLLDHYRSADEPGRLAVLQEILRLEPVVGRLRRRAVSALELPTDDGPLAVRPGEVV

ELRLDHTNTDPRAVGRDPLCVRPGRAMEFGAGPAGLSFGDGPHRCPGAHIALLETDVFLS

RLFALDGIRMTAPPRASFKDVIGGYEIRGLTVGLPPGADRSGPGPGSAR

>CYP157C23(2584334765)SLE

VTPESHAPTGTGDATLAPPPGCPAHALGPGGLHRLHAAEDLGELYEKLRAEHGPVAPALL

HDDVPVWVVLGHAENLHMVRAPAQFCRDSRIWTPLREGMVRPDHPLMPHIAWQPICSHAE

GDEHKRLRGAVAGAISTIDFRDLRRHVNRHAQRLVNRFCGQGRADLVGQFAEHLPMGVMC

EILGMSGEYNDRLVEAARDALKGTDTAIASHAYVMDALARLTARRRAEPGEDLASHLITH

PAGLTDDEVREHLRLVLFAAYENTANLISNVLRVVLTDPRFRAQLSGGQMTVPEAVEQSL

WDEPPFSTIFAYFAKQDIELGGQRIRRGDGLLFGIAPGNVDPRVRPDRFADMQGNRSHLA

FGGGPHECPGQDIGRTIADTGVDALLMRLPDVQLDCDEDELTWRSSIASRHLVELPVRFE

PKPQQEVTEPPSHRPVPAQRAVWHVGMERSAVRTAAVRPPGTTAPSVPPGPPSGPQPEQL

PQAQPQPEIVPPPAPAAASEQPAPAPGDRPQSVWQRVLRWWRSS

>CYP154B8(2584334808)SLE

MEAVACPFALDVLGRDLAGEAAVLRERGPAVRVELPGGVMAWAVVRPGYVRRLLRDRRVS

KDARQHWPAFVEGRITQDWPLFPWVAVENMLFTYGERHARLRRLVAGAFTVRRTQALRPG

VERNVARLLGSLAGVPAGQVVDLRAVFCELLPMRVICDLFGVAEEPGRELCEAMQTVFST

SVSAQEMTAAQARVFGMLAELVAAKQEAPGDDLTSALIAVQDRGEGLSGQELLGTLNLMI

AAGQETTSTLLTNAIAALLAHPEQLEHVRAGRAGWEDVIAETMRTRAAAAYSPMRFAVED

IELDGVLIEKGDPILVSFAAAGLDPEQHGEDAAVFDVLRADRRDGLGFGHGAHFCLGAPL

ARMEAGVALAALFERFPGMTLARPVEEIGPVPSFIINGYSSLPVVLRPSATCAT

>CYP107AM25(2584335259)SLE

MTDQAERPYAPPSLHLPLSGDTPLDPPAEWEELRARCPVAHATLPSGDTAVYLTRYDDVR

ALLSDPRFVRPTDRDNAARLAPEGMGGAAVTGSTAVAIPDRGAPHQRWRRRVGRYFTAKR

MTALRPGMTRLAEDLVDAMLADGAPADLRASLGFPFPVYVICDLLGVPAEDRERFSHWSD

SFLSVTRYTADKIRTAQQEFVAYMSGHVAAKRAEPADDLLSTLIAESETEGSEGSDDGGG

LSHDELVATGMGLLVAGHETTANMIGKMVSMLLCDRSRWEQLLADPSLVRSAVEESLRFD

TNLGFGLRRYIGEDVEIGGHVVPAGSTVVCSMPAANRDERAFDDADTMDLARTPNPHLTF

GAGPHSCLGQALARTELQVVLEALLARVPTLRLAVPVDELRRTEGLLVGGLREVPVRW

>CYP107CH3(2584335418)SLE

MSSPEQCPYEDGRIVIDPAFKADAPARYARLRQSGPIHPAEFHLGLKGWVVVGHDLAREA

LTHPALLKDATPATEALAAAGYVLHRPEVGLGAQMMEADPPEHTRLRRLASTAFTPRRTA

ELAPRIERIAHDLIDALPPSGEVDLVEAFNAPLPATVIAELLGIPREHHLDFRRWSGQAL

QVALPEHRPALAGLHGLLGGLIADKRRRPETDLLSALVAVRDEEDGRLSEEELVGTAMML

VVAGHESTVNLLGNAVLALLLHPEQLRLLRGRPELMPGAVEEFLRYDTSVERSTSRYAAE

DLTLGGVLIPRGGMVVVALGSAGHDAPQTVGTDPALLDVTRPNPRHLAFGHGIHYCLGAP

LARLETAIALRTLLSRLPELELAAPVDALDWIGSGIIRGVLSLPVRYRVA

>CYP166A2(2584335907)SLE

MTDAISFEVPWARTDKFDPPEVFRTLREERPLARMVYPDGHVGWIVSSYELVREVLSDPR

FSHSSEIGHFPVTHHGQVVPNHPKIPGMFIHMDPPDHTRYRRMLTGEFTVRRANRLAPRA

EALAAEQAEVLRRQGAPADLLAHYARPFVLRMLSEVVGLPYDERDRYAHAPTLLHDPDAA

MEDAAAAYAEAGAFFDEVIERRRKEPEDDLISRLVAEGQLTTEELRNIVTLLLFAGYETT

ESALAVGVFALLHHTDQLAEVRAAPQHLDAAVEELLRYLTVNQYDTYRTALEDIELHGEV

IKKGDSVTVSLPAANRDPAKFACPAKLDLGRDTSGHVAFGFGIHQCLGQNLARVELRAGL

GALLRAFPDLRLAVPADEVPLRLQGSVFAVKKLPVAW

>CYP107Q3(2584335918)SLE

MTITPEDAVEPIDLFSPEVVADPFGWYARLREAPTLLKGTMMGGPPMWLATRYDDVYQVL

TDRRFLTNPPAEAAGDPDIREGVFKRLDFPPDLIEYMANILNASDGVDHARLRRLVSSSL

TARRVSNLRPRVEEITESLLDQLAEAGRDGTPVDLVEAFCYPLPVTVICELVGIDEPERP

HWRAWGDAMATMDAEHLPDTLRTCIRLAQEVLDARRAEPRDDLVTALVQAQEEQGNRVSD

REMIGLLFSLVTAGHQTTTYLIGNSVLTLLENPDQLARLKADPSLWPQAVRELQRLGPIQ

FGQPRFPSEDVELRGVTIRAGEPVAPLILAANTDPRKFPDPDRLDIERMAGGSESHLGFG

KGIHRCLGQHLAYQEAEVALHGLFTRFPDLSLAVPREELPWILRPGFTRTATLPLRLA

>CYP105D28(2584335919)SLE

MTEAVAFPQDRSCPYHPPTGYRPLAARDPLAKVTLFDGREVWAVTGHAEARRLLIDPRMS

SDRTNPAFPRTNPAVRQMRTPITAALAGVDDPVHKVQRRMLIPSFTLNRINGLRPRIQET

VDQLLDAMVANGSPTELVGAFAQPLPSMTMCHLLGVPYEDHDFFEEQTIRLTNGPRPLEA

KAALMGYLDALIDKKRQEPGEGLFDDLVHQQFLPGNLEREVLLELVWVLLVAGHDTSANM

ISLGTFTLLQHPERLAELRADLSLVPAAVEELLRYLTIADGLPRVATADIEVGGTTIRKD

DGVVFLASLINRDGDLHERPDELDWHRSHRDHFSFGFGIHQCLGQNLARALLEIAFRSLL

ERLPGLRLAVPAQEVPFKLGQVFQGMVELPVAW

>CYP105B41(SLINC_0400)SLS

MADTLAGPVPESPDSLPEFPMPRQARCPFDPPPALKDLQEKAPLTKVRLWDGSEPWLVTK

YADQRALLGDPRVSADTDSPGYPTKASPEGGEGKLSFIMMDDPEHARLRRMVTAPFAIKR

VEALRPAVQKIVDDLIDGMLGRPGPVDLVEEFALPIPSLVICELLGVPYDDHAFFQDNTK

TMVHRDATPEQRGQASREVAGYLATLIGKRLAEPRDDLLSGIAGRVTAGEIDHRQATEMA

LLLLIAGHETTANMIALGTLALLQNPDQLALLRESDDPKFVAGAVEELLRYLHITHLGRR

RAVTEDIEIGGRLVRAGEGVIMANEIANRDPEVFPDPDRLDLTRDARRHVAFGFGVHQCL

GQPLARMELQVVYGTLYKRIPTLKLACALEDVRFKNDAFIYGVHELPVSW

>CYP163A5(SLINC_0740)SLS

MEENLMTTRRTIAPDELDTLNLADPRLHAESDLSAVWRHLREHEPVHWNPATDSAPGFWA

VTRHADVTAVYRDSTRFTSEGGNVLETLLAGGDSAAGRMLAITDGPRHAALRRILLSAFS

PRALEPIVASVRRTVERLLRDAIDKGSCDFAADVAAGIPLGAICDLLGVPDADRAHVLSL

TSSALGSHDADSTAADAWIAKSEILLYFAGLARDRREGGHADVIALLAGSEVEGLPLDDD

EIMLNCYSLILGGDETARLSMVGAALALLEHPDQWQALRNGDAGIDTAVEEILRWTTPAL

HSGRTATTDTEVGGRAIRTGDIVTVWNASANQDERIFEAPERLLLNRTPNKHVTFAYGPH

FCLGAYLARAEIGAVLAGLRDQVAAMEQTGPAGPVYSNFLSGLGSLPLMLKGA

>CYP1424A1(SLINC_1335)SLS

MTSAVASFNPLDLTHTDDPYPRLAALRRDRPVSSPMPGWHVVVRDEDVRAVLADPEVWSS

RRNNTSVQGDERELALSQVDPPAHTRLRRLLSPAFARTVLAELEPVVRERAETLADRVGN

GSDLVPAFTAPLPRAVLAAFCQVADADAPDYDRWAAAFTDLLGHRAHSDWPAFERWALRA

ADGPALGGVLARLDRTEAVTFLHFLIVAGVPNLRHALGNLVLRLVAEDHWRSDGLPEAIE

ESLRLDPPALWQMRTATRDTAVAGTAVRAGQRVIAVVASANRDPERWGEDADTFRPDRPG

LRAHLAFGKGPHACLGASFTRLQLRVAAEVLIERHPRLRLAPGFRFRRAGDFMSRGPAAL

PVLCGVSSP

>CYP158A15(SLINC_13670)SLS

MTEETLAETLPPVTDWPALDLNGVDFDPVLAELMRQGPVTRIKLPNGEGWAWLVTRHDDV

RMVTNDPRFGREAVMDRQVTRLAPHFIPARGAVGFLDPPDHTRLRRSVAAAFTARGVERV

REKSRRLLDELVGGMLRDGPPADLVASVLTPFPIAVICELMGVPAADRHTMHTWTQLILS

SAHGAQVSEKAKNEMGACFADLIGLREGSTAEDVTSLLGAAVGREEITTEEAVGLAVLLQ

IGGEAVTNNSGQMFYLLLTRPDLAERLRAEPEIRPKAIDELLRHIPHRNAVGLSRIALED

VEIRGHRIRAGDAVYVSYLAANRDPEVFPDPDTIDLTRSPNPHVSFGFGPHYCPGGMLAR

LESGLLVDTLLDRVPDLRLAVPPEQVPFKKGALIRGPEALPVIW

>CYP183Y1(SLINC_1829)SLS

MTGQIDVLTTGWSDGQTVEVGAQMYGLASDTIARTLFTAEAAAPAVDAVTASLPDIFRGI

YQQAVLPPLLRRLPLPPNVRYERARNRAWAAVSKTISSYRQDGGPHGDVLSMLLKTQDDA

GEPLGDAEIHAQIMTLLVAGIDTTAVALTWAVHLLCQYPDVQERLRIETTEVLGTRTATW

EDLPRLDLARRVITETLRLYPPGWIFTRTTSAEARLADAVIPSETTLVYSPYLIHRDPRF

NARPDFFDPDRWLPEREHTRGTLIPFGGGARKCLGDNYAMTLATLALSTIVAQWRLSHGG

LPTPAVEPRMTLAPHKVQVRVESLESEPLADSIRQ

>CYP157C22(SLINC_1832)SLS

MTPESFSPADTYDPTAAPPPGCPAHGIGPGGLRRLYGPDAADLGAVYEELRAEHGPVAPA

LLHDDVPIWVVLGHGENMRMVSTPTHFCRDPRQWDALQNGTIKPDHPLMPHFAWQPICCH

AEGDEHRRLRGAVTGAMSTIDHRGIRRYANRATQLLVNKFCEKGSADLVSQFAEHLPMAV

MCEILGMPEEYNERIVEAARDMLKGTETAIASNAYIMDALTRLTVRRRAEPEEDFTSHLI

NHPARLSDMEISQHLRVVLIAAYETTANLLANVLRVVLTDPRFRAQLNGGQMTVPEAVEQ

SLWDEPPFSTILPYVAKQDTELGGQRVRRGDALLFGIAPGNVDPRVRPEPNANMQGNRSH

LAFGGGPHECPGQDIGRAIADVGVDALLTRIPDVQLACEEHELNWRSFILNRHLVELPVR

FEPKVQQDVSRRPALNPESPQRADDWQVGTVQTRSAAPQPPAAEPVAAETPATAAASTAR

PNVFRRLLSWWRGE

>CYP125A31(SLINC_2675)SLS

MPCPALPEGFDFTDPDLLQHHVPLAELAEVRRAEPVCWIPQPSNLAGFEDEGYWAVTRHA

DVKYVSTHPELFSSYLNTAIIRFNEHIERDAIDAQRLILLNMDPPEHTRVRQIVQRVFTP

RAIRALEDNLRSRALAIAANARARTGSFDFVAEVACELPLQAIAELIGIPQDDRAKIFDW

SNKMISYDDPEYAITEEVGAESATELIAYAMNMAADRKECPAKDIVTTLVSAEDEGNLAS

DEFGFFVLMLAVAGNETTRNAITHGMHAFLTEPGQWELYKRERPVTAAEEIVRWATPVNS

FQRTATQDTELGGKQIKKGDRLGLFYAAANHDPEVFENPDTFDITRDPNPHLGFGGGGPH

YCLGKSLAVLEIDLIFNAVADAMPDLKAVADPRRLRSAWINGVKELRVRAG

>CYP107U1(SLINC_3520)SLS

MTDQPIPLSPAPELFTWEFAGDPYPAYAWLREHAPVQRTRLPSGVEAWLVTRYGDAKQAL

ADPRLSKNPAHHDEPAHAKGKTGIPGERKAELMTHLLNIDPPDHTRLRRLVSKAFTPRRV

AEFAPRVQELTDQLIDGFADKGSADLIHEFAFPLPIYAICDLLGVPREDQDDFRDWAGMM

IRHQGGPRGGVARSVKKMRGYLADLIHRKREALPDEPTPGEDLISGLIRASDHGEHLTEN

EAAAMAFILLFAGFETTVNLIGNGTYALLTHPEQRARLQKSLAGGDRGLLETGVEELLRY

DGPVEMATWRFATRPLSIGGQDIAAGDPVLVVLAAADRDPERFADPDVLDLGRRDNQHLG

YGHGIHYCLGAPLARLEGQTALATLLTRLPDLQLAADPTDLRWRGGLIMRGLRTLPVQFA

PR

>CYP156B13(SLINC_3558)SLS

MDTHHSPAAAGARCPMHDGTFTADPQQIYDFVRAHGPAGPVELAPGVDATLVVEHEMALR

VLQNPSLFARDSRRWKGLNEGRVPLDSPVLAMMAYRPNCLYTDGAVHLRLRQAVTASLDR

LNTRRLRRDVEPIADFLIDQFSERGRADLINDYAKMLPALLFNKLFGCPADIGDTLTGAL

GAFFDGVDVQRANEELTAGLMELVSIKRRRPADDVTSWLIQHPAGLTDEELKDQLIILMG

AGFEPLRNLIGHALHLLLSEEGAGGSGMLVEEAIDHILWNNPPIANYATHFPVQDVDLGG

LIVEANTPVVISFGGANSDPALKEARESRNKGAHLAWGAGPHACPAKDPALVIAVTAIER

LLNALPDLTLGASDDELQWRPGPFQHALVGLPVRFTPTPATRMVSALQTAAPQPAAEQPG

VQTGAEPARAGQPAPKRGFWSTLVDLFRV

>CYP107P15(SLINC_4609)SLS

MAAAPDLAFDPWDPAFVADPYPAYAELRARGRVHYYAPTNQWLVPHHADVSALLRDRRLG

RTYQHRFTHEDFGRTAPPPEHEPFHVLNDHGMLDLEPPDHTRIRRLVSKAFTPRTVERLG

PYVRGLADELVAALVARGGGDLLTDVAEPLPVAVIAEMLGIPEADRAPLRPWSADICGMY

ELNPPEEVAAKAVRASVEFSDYLRELIAERREKPGDDLISGLIAAHDEGDRLTEQEMIST

CVLLLNAGHEATVNATVNGWWALFRTPDQLAALRADHTLVPSAVEELMRYDTPLQLFERW

VLDDIEVAGTTIPRGAEVALLFGSANHDPEVFRDPGRLDVTRRDNPHISFSAGIHYCIGA

PLARLELAASMTALLEKAPTLGLAAEPVRKPNFVIRGLEGLPVTV

>CYP170A20(SLINC_5615)SLS

MTVESVKPEAFPASEQREAPLAGGGVPGLGHGWKLVRDPLGFLARLRDDGDLVRLRLGPK

TVYAVTAPHLVGDMLTSPDYEIGGPLWDTLDVLLGKGVATSNGPRHRRQRQTIQPSFRKE

VIHEYERVMVEESVAFAARWRPGDTVDVTSEAFRVAVRMTARCFLQIERIDDLAERLSTA

LATVFGGMYRRMILSFGPFYRLPLPSHREFDRGLAELHRLADEVIAERRAATEKPDDLLT

ALLEAKDEKGEPVNYQEVHDQVIAILTPGSETVGSQLMWILQLLAEHPEQADRVSEEVKS

VVGDGPVTFGDLRKLTHTNNVITEALRIRPAVWILTRRAMAETELGGYRIPAGADIVYSP

LALQRDPRSYEQHLDFDPDRWLPGHSKQVPKYAMGPFSAGNRKCPADHFSMAQLGVMLAT

VIPRWRFERLPEADESPRVGITLRPKHLLLKAVPR

>CYP182A1(SLINC_5858)SLS

MEAQPRPTAPADSPALGSLPVEPLLTSEFDVDPGAVYERLRATYGPVAPVGLLGVPVWLV

LGYPEVTEVLRNESQWRRDIRYWRARAEGRLPPDWPLAAGYEVRQMMFMDDEEHLAARRT

HHAALRPFSGSPEGWELRAAVERYADELIALLASESGTTGFADLGAQYTRPLLLMVTTRL

FGCPVELGDELVMDLWRMLDGGPGAGPATGRALASFTRLAAHRRARPGDDLTSYMLLADP

DLTDEQLGRELFMNAVYLNDITGNMVLNTLLEVLRGNATVRRSLSAGQLGETVDRAALAN

PPVANMCFRFAARDVRLGGFWIRAGDAVSPSAASAHRDLLAIGSSHLVGSAVSTHAHLGW

GAGPHQCPSAARELGGQIVSTAVGRVFDHFVRAELTLPPDQLPWRSGPVVRGLRLLPVRY

ELSPGHAKTPRHAAPVPTSPTHTPDGQAKRLLSALRRLMFGGRD

>CYP107BM8(SLINC_6504)SLS

MAAVHLTPELVEHPVGAYAELRSRPGLGHVVLPGLATPVRLVTRHDDVRAALGEPRLVRD

RSAIADCELPDPQAELLAQGIEGLPPEYATYLSGHLALFDGDEHARRRGPLTRAFTARRV

AALRPFVERTAQQLIEPLAERGRADLLGEFAYPLSTAVICALVGVDAADRDRVCGWIRDF

AYGDGGRALEGLGGIVEYTKDLVARRRVEPTDDLVSALLADGGLTDDEIVGVFFLLIDTG

ITPPALFLAHAVLALLDHPDQLERLRADPELLSRAVPELLRYVTLVRVGATLYATEDFVF

AGTRLRRGEAVTVALFAADHDPAAYDVPERLDVTREFGRGDGHLAFGHGPHYCIGAALGR

LVTGVVLEELVVRRSAAPELAVDRADVPFGHWPGDGFHLLRLPVRL

>CYP107L32(SLINC_6791)SLS

MDDVIDLGEYGDAFRTDPHPVYAGLRERGPVHRIRPPGSTPDYWSWLVVGHEEVRAALAD

PRLSKDGRRIGMVFNDEQLIGRHLLGTDPPEHTRLRGLVSRAFTMRRVERLRPRVQEITD

GLLDAMLPLGRADLVESLAYPLPITVICELLGVPEMDRAEFRKQSTEVVAPTSPENSYDA

VLRLGEYLTELIEDKRRSGPGDDLLGDLIRTTAEDGDRLSPAELRGMAFLLLIAGHETTV

NLITNAVHALLTHPDQLAALRADMSLVDSAVEETLRHEGPVANATFRYAAEPLEIAGTPI

AQGDPVMIGLTAADRDEARYPDPHRFDIHRDTRGHVAFGHGIHFCVGAPLARLEARTALR

SLLERAPALTLDGPPGEWLPGMLIRGLRSLPVRW

>CYP182B1(SLINC_7237)SLS

MEFHLVDEGESAPGDDAPLEALAPEPLLTRDYETRPALVYERLRQRHGPVAPVDLLGVPA

WLVLGYKESLQVLQEDAAWPKGLENWRARTEGRVPADWPLGPSLEVNHVLIQGGPGYPAL

RSAWDTALRPFQDPRHPQAKRLKAAVTVYADDLITLLAQGGRTGMADLSAQFSRPLPLMA

ASHLLGFPGSQGDDALMDMWRVLDAGPDAEPALERLLETLMRLAAAKMERPGDDFPSHLL

AAHPDLSVDALARELFMLLGMTSDHVGILISNTVVEVISGESEGGVRAALSAGMVRETMN

RVVMRKPPLVNFVPRFAARDTRLGNYTIRAGDPVWVSSAAAHADPLFAGQMAPGSTLSSR

AHLSWGAGPRQCPARELASVTIAAAGVGRLFERFGHLELALPVDQLPWRSSPFMRGLRSL

PVRYELASATVPPLPVPEEAVAAATGVPDQAEKRRSSLWRYLTGLIRSGG

>CYP158A1(SLINC_7316)SLS

MTEETTTLTPQAPPPVRHWPALDLKGTEFDPLLAEFMREGPLTRIQLPHGEGWAWLATRY

DDVKTITNDPRFSRAEVTGRQVTRLAPHFKPRPGSLAFADQPDHNRLRRAVAGAFTVGAA

KRLRPRAQEMLDGLVDGMVRAGPPADLVERVLEPFPVALVSEVMGVPAADRERVHAWTRT

IISTSGGAEAAERAKCGLYGWITDTVRARAHSTGDDVYSLLGAAVARGDIGADEAVGLAG

PLQIGGEAVTHNCGQLLYLVLTRPELMARMREEPGARAAAVDELLRHIPHRTSVGLARIA

LEDVELHGHRIAAGDAVYVSYLAANRDPAVFPDPERIDVDRDPNPHLAFGNGPHHCTGAV

LARMQTELLVGTLLDRLPGLRLAVPADQVLWRRKTMIRGPQTLPVTW

>CYP102B22(SLINC_7657)SLS

MAERTPGTGRPRGFRSAELGWPELERLPHPPRRVPLLGDVLGVDRHSPLQDTVRHARRLG

PIFRRKAFGREFVFTWGADLVADLADEARFAKHVGLGVANLRPVAGDGLFTAYNHEPNWQ

LAHDVLAPGFNREAMQGYHAMMLAVAGRLTDHWDRAQAAGLEVDVPGDMTKLTLETIART

GFGHDFGSFERARPHPFVAAMVGTLTYAQRLNSVPFPQLLRAAARRNEADIAHLNRTVDE

LVRDRRTSGADGGGDLLDRMLETSHPQTGERLSAENVRRQVITFLVAGHETTSGALSFAL

HYLARHPEIAARARAEVDQVWGDTAEPGYDQVAKLRHVRRVLDETLRLWPTAPAFAREAR

EDTVLAGVHPMRRGAWTLILLPMVHRDPAVWGADAERFDPDRFEAKAVRSRPAHTYKPFG

TGARACIGRQFALHEATLVLGLLLRRYELRPDPAYRLRVTERLTLMPERLRLRLERRSAP

VTVPSPAAAEAPASEPRCPVHGAGD

>CYP102G9(SLINC_7669)SLS

MPPTAQTTPDLPDVPVVDISDTGPGCTPLQQVMGLMREHGPLLVRRLHGRDVTFVADLDL

VTELADETRFAKAVGPALENVREFAADGLFTAYSEEPNWARAHDILMPAFALGSMRTYHP

VMLEVARRLIGSWDRAAFAGRPVNVPDDMTRMTLDTIGLAGFGYDFGSFDRDEPHPFVES

MVRCLEWAMTRLARTPGSDHTAADEAFRTDAAHLAQVVDDVINARLASGESGGDDLLGLM

LTATHPADGTTLDTTNIRNQVITFLIAGHETTSGAMSFAMYYLAKHPAVLQLVRREVDEL

WGDTADPEPTFDEVGRLTYTRQVLNEALRLWPTAAAFSRQAREDTLLGGRIPLRAGQALT

VVAPMLHRQPVWGDNPELFDPSRFTPEAEAARSPHAFKPFGTGERACIGRQFALHEATML

LALLVHRYRLHDHADYRLTVKEALTLKPDGFTLTLTPRTGADRAHPPLPGAAPLTEAAAV

SDAALPARVRRGTRALFLHGSNYGTCRDFAARLADEAAAVGCETEVAPLDAYAGGLPTDR

TVVITAASYNGQPTDDARQFATWLDEATDAGGVHYAVLGVGDRNWAATYQHVPTRIDERL

AELGATRLLDRAAGDASGDLTGTVRAFTADLRTALLTAHGDPDAVAPAPEPETAYEVRTL

TSGPLDALAERHDLVPMTVTEAYGLTAPGHPRLKRFVRLALPAGTTYRTADHVTVLPVND

KALVERAAAALGVDLDSVLDIRATRPRSDGLAVDRPLTVRQLLAHHVELLRRPSAGQRSA

LATANPCPPERAQLAALGDDDPRTLVELFEAYPALRGALDWPALLDLLTPLRPRHYSVSS

SPATDPGHVDLMVSLLEAPARSGNGTYRGVGSGHLAGVQPGDTVLARVQPCRDAFRVDHS

RPVVMIAAGTGLAPFRGAVADRVAALARGEQLPPALCYFGCDAPEADFLHAEELRAAQTA

GAVRLRPAFSAVPEGEVRFVQHRVAAEADEVAELLDAGARVYVCGDGSRMAPGVREAFRT

LYRERTPGADEAAAERWLDGLVRDGRYVEDVYAAG

>CYP107BX8(SLINC_7684)SLS

MSTDSTSRPLCPMHHIAFPESGPPRPGTLATGTPVWLVTRYAEVRQVLMDPRFDRRSLKA

EDAPPLLVVPNLLDTPDGLLNQDGPAHQRLRGTVQRAFTPRAIARWRPWVASVVESLLDD

FARRPRPADIIEGFTRPLPVAVISRLMGLDHADWDRIRDWADHALSGGAHTADEVGTAML

EFGVFCADLVAERRKDPGDDLVSGLVAAGDGLGIDERQLVILVLGLVVAGHETTMTALGN

IVVHLLTDGREAWPRLAEYEDNAATAVENLLRTVPLSEGRVLPGLIRRAVEDVELGGVTI

PAGAVVAVQINSANRDPEVFPPGPPDLFTPLSSPTVVFGAGPHHCLGAWLARLELGLALH

RLAARFPGLKAEFTPETIEWREGQMTRSPRRLPVSW

>CYP105D21(SLINC_7705)SLS

MTETQPVAFPQDRTCPYHPPTGYDSLRAERPLSRITLYDGTQAWMVSGHGAARALLADPR

LSTDRTHPRFPAPTARFAAVRNKRAALLGVDDPEHRTQRRMMIPSFTLKRATELRPRIQQ

VVDERLDAMIAQGPPAELVSAFALPVPSTVICALLGVPYADHDFFEGQSRRLLRGPAAED

VLDAREQLASYFDELIDRKEKQRDPGDGVLDELVHRRLREGEMDREELIALALILLVAGH

ETTANMISLGTYTLLQHPDRLAELRGDPALLPIAVEELMRMLSIADGLLRMATEDIEVAG

TTIRAGEGVVFATSVINRDEDVYADPDTLDWHRPARHHVAFGFGIHQCLGQNLARAELEI

ALRSLFERLPTLRLAAPAEEIPFKPGDTVQGMLELPVTW

>CYP108B16(SLINC_7942)SLS

MPMSVAEAGVTLADPTAYADEDRLHRALDLLRREAPVHWVMPPGYHPFWAVTRHADVREV

ERRSDVFLSGPRPMLAPARTVASGTGGGRKGLLRPLAHLDGAEHHVMRAVTASRFTPQAV

AALRPQIRDLARGAVDRMAGRQGEVCEFVGEVADVYALGVLLLLLGLDDSEAATVGRFTP

AARRSMAPEQRTATMREFYTYFLALTADRRARPRDDLASVIANARVDGQLLDDHEVLSQF

VILLMAGHDTASVTVAGGLLALTEHPGQLSALRDGAARLPDAVEEMIRWVTPVKAIMRTA

RVDHALHGVRIRAGEAVLLSYPSANRDESVFPDPHRFDVHRTSNRQLAFGYGVHHCLGAS

LARLEIEEFFAALLPRLVSATVAGPPVQLTTTFSGGLKLFPVRCVLKN

>CYP180B1(SLINC_8202)SLS

MTSPTARAVPTTRAELRAALDEPMPLDQVDLADLDNFADGVTPWRMFHTLRHQDPIHWQP

EEAPDSGFWAVTRHADIARVDRDAETFTSTKFVNLEEVDEDQIKKRASILELDGVRHRAL

RSVIQRQFGAGVINSYTDFLRGLTATTLDAALAKGTFDFVADVSADFPINVLARLLDVPP

KDNQRLIDWGNRIIGNTDPDYADVLLNSAESEQYRDLPFRSPASLEVFEYGRELARQRRG

GDGTDLVSKLVNTTPRDGVPLSAQDFDNYFLLLVVAGNETTRHTISHSMLALLQHPEQLA

RLKDDPSLIPTAVEEFLRWASPVYHFRRTATRDVELGGKQVKEGDKVVMWFASGNRDEDV

FDNPYDLDVTRTDNDHVTFGKGSPHLCLGNLLARTEIRIMFEELIPRIADIKLAGDVPRV

RSNFVNGIKKLPVEVTLT

>CYP1215A8(SLINC_8266)SLS

MSMRTPSSLLRAFRPQVQGRLYDFYDELRSTDDLFWDHRLDAWVATGHAVVSSAAGDPRL

SSVRYPDIAAVSEELRPLARVLSRQMLYSDAPDHPRLRALISKAFAPRAVATLRARIVEA

VDRIITHAAPTGRMDIVADLARPLPLTIICDLLDVPHRDRPALAAWSEPVAEAIGNSRLD

ADGNRAASQSMADLLTYLRGLLTRQDTPPAPHTLRALIDAQAQDTGQDLDELLANCALLL

IAGHETTTHFIGNATLALLRHPQAADQLRRRPDLMPAAVEELLRYDAPVQLMLRRARQDM

DLAGRAVGEGQTVLLVCGAANRDPAVFPDPHVLDFGRAGGRHMAFGHGPHFCLGAALARM

EGAIALEALLTRLPDLRLDGTEPQWQRSLNFRGLTRLDVAFAPVPDAQDTGVQEPVAGLS

REAGGLQV

>CYP105BQ1(SLINC_8569)SLS

MESTSPPGVMPTARQNPFDPPEELRRRQRLGPIHRMTYADGDQGWLVTGFAAARATLTDP

RFSVRPDRMRSPTAQPATAPAPPGFFLRTDPPEHDHYRRLLTGHFTVRRMKLLEPEIEAI

VNDHLNAMELAGGPADLLKTFALPFPSQVIGELLGVPYSDRQGFQRNAATLLNADLTPEP

RQAAVRELMAYLRDLLRHKRSRPEEDVLSGLAAHEGLAADEKAGLALLLLIAGHETTANL

LALGTFALLANPAQLAEVRDQEEGAPAVVEELLRYLTIIPHVVRVAREDTELHGCPIKAG

ESVTVALSAANRDADHFTDPDALDLTRSTAGHLAFGHGLHRCLGQRLARAEIRIALPALL

RRFPALRLTVAPEEVPLRSAMTVYGVRELPVTW

>CYP107T1(2598191635)SLV

MTAPTYEELAALRAVGAVHRVFVPGSGESRLVVTRDAARAALTDPRLRNDIRHSASWDSD

GGHAIGHNMLQSDPPQHTRLRRLVAGHFTPGRTAALRPRVERIAHGLLDALPPAGTADLV

ARYALPLPVTVICELLGVPESDRGTFHTWSNELVMPTSPEAAGSAATALTGYLTELTDAK

RRTPDGTLLGDLVAAADSGELTPGELLGMAFLILVAGHETTVNLISATVHGLLTHPGQLA

RLRAEPELTEAAVEESLRYHSPVHASAFRFAAEPLELAGTAIAAGDPVLVSLAAASRDPA

HFPDPDRFDIGRRPRGHLGFGHGPHHCLGAPLARVEAAVAVRLLLDRHPALALAADPATL

TWRTSTLLRGLVELPVRLG

>CYP107P1(2598191763)SLV

MTAATDGPHVSGPAFDPWDPAFVADPYPAFAELRARGRVLYYEPSDQWLVPHHADVSALL

RDRRLGRTYQHRFTHEDFGRTPPPPEQEPFHTLNDHGMLDLEPPDHTRIRRLVSKAFTPR

TVERLKPYVHGLADDLVARLVAAGGGDLLTDVAEPLPVAVIAEMLGIPESDRAPLRPWSA

EICGMYELNPSEETAAKAVRASLDFSDYLRALIAARRKEPGDDLISGLIAAHDEDDRLTE

QEMISTCVLLLNAGHEATVNATTNGWLALFRHPDQLAALRADHSLVPSAVEELMRYDTPL

QLFERWVLDEIEIDGTTLPRGAEVAMLFGSANHDPAVFTDPERLDLTRRDNPHISFSAGI

HYCIGAPLARIELAASMTSLLKRAPGLRLAAEPERRPNFVMRGLTELRVEL

>CYP107U1(2598192190)SLV

MTGSSSAPVPELFSWEFASDPYPAYAWLREHAPVHRTRLPSGVEAWLVTRYADAKQALAD

PRLSKNPAHHDEPAHAKGKTGIPGERKAELMTHLLNIDPPDHTRLRRLVSKAFTPRRVAE

FAPRVQELADGLIDRFADTGSADLIHDFAFPLPIYAICDLLGVPREDQDDFRDWAGMMIR

HQGGPRGGVARSVKKMRGYLADLIHRKRAALPPEPAPGEDLISGLIRASDHGEHLTENEA

AAMAFILLFAGFETTVNLVGNGTYALLTHPEQRERLQTSLAAGERGLLETGVEELLRYDG

PVELATWRFATRPLTIGGQDVAAGDPVLVVLAAADRDPERFTDPDTLDLARRDSQHLGYG

HGIHYCLGAPLARLEGQTALATLLTRLPDLRLAADPAELRWRGGLIMRGLRTLPVSFTPP

ASSAGNGPSPTQK

>CYP154A1(2598192412)SLV

MATQQPALVLDPTGADHHTEHRTLREGGPATWVDVLGVQAWSVSDPVLLKQLLTSSDVSK

DARAHWPAFGEVVGTWPLALWVAVENMFTAYGPNHRKLRRLVAPAFSARRVDAMRPAVEA

MVTGLVDRLAELPAGEPVDLRQELAYPLPIAVIGHLMGVPQDRRDGFRALVDGVFDTTLD

QAEAQANTARLYEVLDQLIAAKRATPGDDMTSLLIAARDDEGDGDRLSPEELRDTLLLMI

SAGYETTVNVIDQAVHTLLTRPDQLALVRKGEVTWADVVEETLRHEPAVKHLPLRYAVTD

IALPDGRTIARGEPILASYAAANRHPDWHEDADTFDATRTVKEHLAFGHGVHFCLGAPLA

RMEVTLALESLFGRFPDLRLADPAEELPPVPSLISNGHQRLPVLLHAG

>CYP156A1(2598192413)SLV

MTLPSTETAPTGEPGRIALYAPEFAADPHAAYRSMRRTHGPLVPVDLAPGVPATLVIGYY

QARRILNDPLRFPADPRAWEKLIPATCPVRPMMEWRPNALRSGGAEHTRYRSANTHAIDQ

VDQHGLRALVEQVASDAIEGFRTAGSADLLTQYSFPIAFRVLSALLGCPDEIGQRIADGM

AKIFDTTNADQGNLILAQAVSDLVTLRRTHPGDDITSRLALHPVRLTDEEMSHQLVTLYG

AGIEPMTNLISNTILKILTDEEFSADLHAGLSTVRDALDAVLYTDPPMANYCISYPPYPV

DVEGVLLPADQPVVISMAAANNDPALTEGVPAGQHGGNRAHLAWSTGPHTCPARSHAYLI

AETAVTHLLDALPETDLARPAAELVWRPGPFHRALESLPVTFPAAQSAAH

>CYP157C1(2598193685)SLV

VTPESHSPTGTGEPLLEPPPGCPAHGLGPGGLHRLHEAEDLEELYEKLREQHGPVAPALL

HDDVPMWVVLGHAENLHMVSTPSQFCRDSRIWTPLNEGMVKPDHPLMPHIAWQPICSHAE

GDEHKRLRGAVTSAMSDLDYRELRRHIKRYTQRVVNRFCEEGRADLVSQFAEHLPMGVMC

HLLGMPEEYNDRLVEAARDTLKGTETAIASHAYVMEALGRLTATRRADPADDIAGRLVTH
[truncated: 371,562 more chars]
