# Supplementary material for: Similarities, variations, and evolution of cytochrome P450s in Streptomyces versus Mycobacterium
Source: Sci Rep. 2019 Mar 8;9:3962. doi: 10.1038/s41598-019-40646-y (PMC6408508; doi:10.1038/s41598-019-40646-y)
Supplement: Supplementary file 2 — Dataset 1 [file 41598_2019_40646_MOESM2_ESM.docx]

**Similarities, variations, and evolution of cytochrome P450s in *Streptomyces* versus *Mycobacterium***

Louisa Moshoeshoe Senate^1@^, Martin Phalane Tjatji^1@^, Kayla Pillay^2@^, Wanping Chen^3@^, Ntokozo Minenhle Zondo^2^, Puleng Rosinah Syed^4^, Fanele Cabangile Mnguni^2^, Zinhle Edith Chiliza^2^, Hans Denis Bamal^1^, Rajshekhar Karpoormath^4^, Thandeka Khoza^5^, Samson Sitheni Mashele^1^, Jonathan Michael Blackburn^6^, Jae-Hyuk Yu^7,8^, David R Nelson^9*^, Khajamohiddin Syed^2*^

^1^ Unit for Drug Discovery Research, Department of Health Sciences, Faculty of Health and Environmental Sciences, Central University of Technology, Bloemfontein 9300, Free State, South Africa

^2^Department of Biochemistry and Microbiology, Faculty of Science and Agriculture, University of Zululand, KwaDlangezwa 3886, KwaZulu-Natal, South Africa

^3^ College of Food Science and Technology, Huazhong Agricultural University, Wuhan, Hubei Province, China

^4^ Department of Pharmaceutical Chemistry, College of Health Sciences, University of KwaZulu-Natal, Durban 4000, KwaZulu-Natal, South Africa

^5^Department of Biochemistry, School of Life Sciences, University of KwaZulu-Natal (Pietermaritzburg campus), Scottsville, 3209, KwaZulu-Natal, South Africa

^6^Institute of Infectious Disease & Molecular Medicine; Department of Integrative Biomedical Sciences, Faculty of Health Sciences, University of Cape Town, Cape Town 7925, South Africa

^7^Department of Bacteriology, University of Wisconsin-Madison, 3155 MSB, 1550 Linden Drive, Madison WI 53706, USA

^8^ Department of Systems Biotechnology, Konkuk University, Seoul, Republic or Korea

^9^ Department of Microbiology, Immunology and Biochemistry, University of Tennessee Health Science Center, Memphis, TN 38163, USA

 @ Authors contributed equally to the work

* Corresponding authors’ email:

[drnelson1@gmail.com](mailto:drnelson1@gmail.com) & [khajamohiddinsyed@gmail.com](mailto:khajamohiddinsyed@gmail.com)

**Supplemenatary Dataset 4.** Secondary metabolite biosynthetic gene clusters and P450 analysis in 48 *Streptomyces* species and 60 mycobacterial species. The number of P450s that formed part of each cluster and their corresponding P450 names (in parenthesis) were also presented in the table. Standard gene cluster abbreviation terminology available on the anti-SMASH database^33^ was used in the table.

***Streptomyces* species**

| ***Streptomyces coelicolor*** | | | | | | | |
| --- | --- | --- | --- | --- | --- | --- | --- |
| **Cluster** | **Type** | **Frrom** | **To** | **Most similar known cluster** | **MIBiG BGC-ID** | **P450s** | **Name of the P450** |
| Cluster 1 | T1pks-Otherks | 86637 | 139654 | Leinamycin_biosynthetic_gene_cluster (2% of genes show similarity) | BGC0001101_c1 |  |  |
| Cluster 2 | Terpene | 166501 | 192038 | Isorenieratene_biosynthetic_gene_cluster (100% of genes show similarity) | BGC0000664_c1 |  |  |
| Cluster 3 | Lantipeptide | 235986 | 271084 | Sanglifehrin_A_biosynthetic_gene_cluster (4% of genes show similarity) | BGC0001042_c1 |  |  |
| Cluster 4 | Nrps | 493989 | 544920 | Coelichelin_biosynthetic_gene_cluster (100% of genes show similarity) | BGC0000325_c1 |  |  |
| Cluster 5 | Bacteriocin | 791584 | 801799 | Informatipeptin_biosynthetic_gene_cluster (42% of genes show similarity) | BGC0000518_c1 |  |  |
| Cluster 6 | T3pks | 1257625 | 1298749 | Herboxidiene_biosynthetic_gene_cluster (8% of genes show similarity) | BGC0001065_c1 | 1 | CYP158A2 |
| Cluster 7 | Ectoine | 1995500 | 2005898 | Ectoine_biosynthetic_gene_cluster (100% of genes show similarity) | BGC0000853_c1 |  |  |
| Cluster 8 | Melanin | 2939306 | 2949875 | Lactonamycin_biosynthetic_gene_cluster (3% of genes show similarity) | BGC0000238_c1 |  |  |
| Cluster 9 | Siderophore | 3033895 | 3045682 | Desferrioxamine_B_biosynthetic_gene_cluster (100% of genes show similarity) | BGC0000940_c1 |  |  |
| Cluster 10 | Nrps | 3523335 | 3603988 | Calcium-dependent_antibiotic_biosynthetic_gene_cluster (90% of genes show similarity) | BGC0000315_c1 |  |  |
| Cluster 11 | T2pks | 5509801 | 5552424 | Actinorhodin_biosynthetic_gene_cluster (100% of genes show similarity) | BGC0000194_c1 |  |  |
| Cluster 12 | Terpene | 5671016 | 5692101 | Albaflavenone_biosynthetic_gene_cluster (100% of genes show similarity) | BGC0000660_c1 | 1 | CYP170A1 |
| Cluster 13 | T2pks | 5766945 | 5809487 | Spore_pigment_biosynthetic_gene_cluster (66% of genes show similarity) | BGC0000271_c1 |  |  |
| Cluster 14 | Siderophore | 6335587 | 6347533 | - | - |  |  |
| Cluster 15 | T1pks | 6429549 | 6476442 | Undecylprodigiosin_biosynthetic_gene_cluster (100% of genes show similarity) | BGC0001063_c1 |  |  |
| Cluster 16 | Bacteriocin | 6632343 | 6643659 | - | - |  |  |
| Cluster 17 | Terpene | 6656219 | 6678399 | - | - |  |  |
| Cluster 18 | Siderophore | 6842315 | 6855522 | Enduracidin_biosynthetic_gene_cluster (8% of genes show similarity) | BGC0000341_c1 |  |  |
| Cluster 19 | T1pks-Butyrolactone | 6871293 | 6951959 | Coelimycin_biosynthetic_gene_cluster (100% of genes show similarity) | BGC0000038_c1 |  |  |
| Cluster 20 | Nrps | 7088264 | 7136089 | Nogalamycin_biosynthetic_gene_cluster (40% of genes show similarity) | BGC0000249_c1 |  |  |
| Cluster 21 | Lantipeptide | 7409664 | 7432456 | SAL-2242_biosynthetic_gene_cluster (100% of genes show similarity) | BGC0000546_c1 |  |  |
| Cluster 22 | Terpene | 7506017 | 7532758 | Hopene_biosynthetic_gene_cluster (100% of genes show similarity) | BGC0000663_c1 |  |  |
| Cluster 23 | T1pks-Otherks | 7570412 | 7618555 | Arsenopolyketides_biosynthetic_gene_cluster (100% of genes show similarity) | BGC0001283_c1 |  |  |
| Cluster 24 | Lantipeptide | 7685016 | 7709795 | - | - |  |  |
| Cluster 25 | Other | 7973470 | 8014357 | - | - |  |  |
| Cluster 26 | Indole | 8269637 | 8290764 | Ravidomycin_biosynthetic_gene_cluster (5% of genes show similarity) | BGC0000263_c1 |  |  |
| Cluster 27 | T3pks-Terpene-Nrps | 8475102 | 8558352 | Coelibactin_biosynthetic_gene_cluster (100% of genes show similarity) | BGC0000324_c1 | 1 | CYP105N1 |
| ***Streptomyces avermitilis* MA-4680** | | | | | | | |
| Cluster 1 | Terpene | 76073 | 97080 | - | - |  |  |
| Cluster 2 | Lassopeptide | 290466 | 313052 | SSV-2083_biosynthetic_gene_cluster (25% of genes show similarity) | BGC0000579_c1 |  |  |
| Cluster 3 | T1pks | 478846 | 587017 | Filipin_biosynthetic_gene_cluster (100% of genes show similarity) | BGC0000059_c1 | 2 | CYP105D6; CYP105P1 |
| Cluster 4 | Nrps | 734376 | 780552 | Herbimycin_biosynthetic_gene_cluster (13% of genes show similarity) | BGC0000074_c1 | 1 | CYP147B1 |
| Cluster 5 | Nrps-T1pks-Otherks | 958292 | 1062269 | Landepoxcin_biosynthetic_gene_cluster (11% of genes show similarity) | BGC0001202_c1 | 2 | CYP178A1; CYP178A3P |
| Cluster 6 | T1pks | 1117817 | 1222573 | Avermectin_biosynthetic_gene_cluster (100% of genes show similarity) | BGC0000025_c1 | 1 | CYP171A1 |
| Cluster 7 | Terpene | 1277518 | 1302828 | Carotenoid_biosynthetic_gene_cluster (100% of genes show similarity) | BGC0000633_c1 |  |  |
| Cluster 8 | Melanin | 1419869 | 1430225 | Melanin_biosynthetic_gene_cluster (100% of genes show similarity) | BGC0000908_c1 |  |  |
| Cluster 9 | T1pks-Nrps | 1875828 | 1932282 | Daptomycin_biosynthetic_gene_cluster (7% of genes show similarity) | BGC0000336_c1 |  |  |
| Cluster 10 | Terpene | 2010191 | 2036846 | Hopene_biosynthetic_gene_cluster (92% of genes show similarity) | BGC0000663_c1 |  |  |
| Cluster 11 | Siderophore | 2436883 | 2449994 | Grincamycin_biosynthetic_gene_cluster (8% of genes show similarity) | BGC0000229_c1 |  |  |
| Cluster 12 | Terpene | 2625583 | 2647760 | - | - | 1 | CYP180A1 |
| Cluster 13 | Lassopeptide | 2660814 | 2683259 | - | - |  |  |
| Cluster 14 | Bacteriocin | 2692334 | 2703797 | - | - |  |  |
| Cluster 15 | T1pks-Butyrolactone-Otherks | 2746968 | 2811733 | Chlorizidine_A_biosynthetic_gene_cluster (11% of genes show similarity) | BGC0001172_c1 |  |  |
| Cluster 16 | T2pks-T1pks-Otherks | 2859868 | 2921483 | Mannopeptimycin_biosynthetic_gene_cluster (14% of genes show similarity) | BGC0000388_c1 | 2 | CYP107Y1; CYP181A1 |
| Cluster 17 | Siderophore | 3004049 | 3016109 | - | - |  |  |
| Cluster 18 | T2pks | 3464232 | 3506747 | Spore_pigment_biosynthetic_gene_cluster (100% of genes show similarity) | BGC0000271_c1 |  |  |
| Cluster 19 | T1pks | 3516766 | 3646347 | Oligomycin_biosynthetic_gene_cluster (100% of genes show similarity) | BGC0000117_c1 | 2 | CYP107W1; CYP105B23 |
| Cluster 20 | Terpene | 3744725 | 3765735 | Pentalenolactone_biosynthetic_gene_cluster (100% of genes show similarity) | BGC0000678_c1 | 1 | CYP183A1 |
| Cluster 21 | Terpene | 3780128 | 3801219 | Albaflavenone_biosynthetic_gene_cluster (100% of genes show similarity) | BGC0000660_c1 | 1 | CYP170A2 |
| Cluster 22 | Nrps | 3910306 | 3957489 | Meilingmycin_biosynthetic_gene_cluster (2% of genes show similarity) | BGC0000093_c1 |  |  |
| Cluster 23 | Nrps | 3960213 | 4012310 | Naphthomycin_biosynthetic_gene_cluster (9% of genes show similarity) | BGC0000106_c1 |  |  |
| Cluster 24 | Butyrolactone-Otherks | 4335459 | 4381172 | Herboxidiene_biosynthetic_gene_cluster (4% of genes show similarity) | BGC0001065_c1 | 2 | CYP107V1; CYP107U2 |
| Cluster 25 | Ladderane-Arylpolyene-Nrps | 4480084 | 4560161 | WS9326_biosynthetic_gene_cluster (22% of genes show similarity) | BGC0001297_c1 |  |  |
| Cluster 26 | Terpene | 4831782 | 4852768 | - | - |  |  |
| Cluster 27 | Siderophore | 6378181 | 6389953 | Desferrioxamine_B_biosynthetic_gene_cluster (100% of genes show similarity) | BGC0000940_c1 |  |  |
| Cluster 28 | Melanin | 6495109 | 6505495 | Melanin_biosynthetic_gene_cluster (100% of genes show similarity) | BGC0000909_c1 |  |  |
| Cluster 29 | Lassopeptide | 6869386 | 6891873 | Pimaricin_biosynthetic_gene_cluster (11% of genes show similarity) | BGC0000125_c1 |  |  |
| Cluster 30 | Nrps | 6903259 | 6953329 | SCO-2138_biosynthetic_gene_cluster (50% of genes show similarity) | BGC0000595_c1 |  |  |
| Cluster 31 | Ectoine | 7666129 | 7676533 | Ectoine_biosynthetic_gene_cluster (100% of genes show similarity) | BGC0000853_c1 |  |  |
| Cluster 32 | Other | 7913326 | 7957201 | Pristinamycin_biosynthetic_gene_cluster (2% of genes show similarity) | BGC0000952_c3 |  |  |
| Cluster 33 | T3pks | 8471426 | 8512484 | Herboxidiene_biosynthetic_gene_cluster (8% of genes show similarity) | BGC0001065_c1 | 1 | CYP158A3 |
| Cluster 34 | T1pks | 8533602 | 8578155 | Kirromycin_biosynthetic_gene_cluster (5% of genes show similarity) | BGC0001070_c1 | 1 | CYP105R1 |
| Cluster 35 | Siderophore | 8727144 | 8740816 | - | - |  |  |
| Cluster 36 | T1pks-Otherks | 8758095 | 8809766 | Herboxidiene_biosynthetic_gene_cluster (12% of genes show similarity) | BGC0001065_c1 |  |  |
| Cluster 37 | Bacteriocin-Lantipeptide | 8921460 | 8953908 | Informatipeptin_biosynthetic_gene_cluster (100% of genes show similarity) | BGC0000518_c1 |  |  |
| ***Streptomyces griseus*_NBRC_13350** | | | | | | | |
| Cluster 1 | Terpene | 47111 | 72706 | Isorenieratene_biosynthetic_gene_cluster (100% of genes show similarity) | BGC0000664_c1 |  |  |
| Cluster 2 | Lantipeptide | 149896 | 172562 | - | - |  |  |
| Cluster 3 | T1pks-Nrps | 274490 | 327047 | Daptomycin_biosynthetic_gene_cluster (7% of genes show similarity) | BGC0000336_c1 | 1 | CYP105D1 |
| Cluster 4 | T3pks-Nrps | 463570 | 571330 | Tetronasin_biosynthetic_gene_cluster (11% of genes show similarity) | BGC0000163_c1 |  |  |
| Cluster 5 | Melanin | 603389 | 613793 | Melanin_biosynthetic_gene_cluster (100% of genes show similarity) | BGC0000911_c1 | 1 | CYP124G2 |
| Cluster 6 | T1pks-Nrps | 640959 | 793028 | Sporolide_biosynthetic_gene_cluster (53% of genes show similarity) | BGC0000150_c1 | 3 | CYP162C1; CYP208A1; CYP154M2 |
| Cluster 7 | Bacteriocin | 899922 | 910719 | Tetronasin_biosynthetic_gene_cluster (3% of genes show similarity) | BGC0000163_c1 |  |  |
| Cluster 8 | T1pks-Nrps | 936042 | 985512 | SGR_PTMs_biosynthetic_gene_cluster (100% of genes show similarity) | BGC0001043_c1 | 1 | CYP107BX5 |
| Cluster 9 | Nrps | 1040073 | 1091469 | Nucleocidin_biosynthetic_gene_cluster (47% of genes show similarity) | BGC0001387_c1 | 1 | CYP107BY1 |
| Cluster 10 | Terpene | 1127168 | 1153741 | Hopene_biosynthetic_gene_cluster (69% of genes show similarity) | BGC0000663_c1 |  |  |
| Cluster 11 | Terpene | 1481583 | 1502896 | 2-methylisoborneol_biosynthetic_gene_cluster (100% of genes show similarity) | BGC0000658_c1 |  |  |
| Cluster 12 | Bacteriocin | 1704704 | 1716146 | - | - |  |  |
| Cluster 13 | Linaridin | 1766706 | 1787314 | Legonaridin_biosynthetic_gene_cluster (22% of genes show similarity) | BGC0001188_c1 |  |  |
| Cluster 14 | Siderophore | 2025368 | 2040162 | - | - |  |  |
| Cluster 15 | Terpene | 2450742 | 2471749 | - | - |  |  |
| Cluster 16 | Lantipeptide | 2816830 | 2839457 | AmfS_biosynthetic_gene_cluster (100% of genes show similarity) | BGC0000496_c1 |  |  |
| Cluster 17 | Melanin | 2879832 | 2890254 | Melanin_biosynthetic_gene_cluster (100% of genes show similarity) | BGC0000912_c1 |  |  |
| Cluster 18 | T1pks-Nrps | 2907245 | 2961895 | Enduracidin_biosynthetic_gene_cluster (10% of genes show similarity) | BGC0000341_c1 |  |  |
| Cluster 19 | Nrps | 3029350 | 3094603 | Arixanthomycin_biosynthetic_gene_cluster (5% of genes show similarity) | BGC0000200_c1 |  |  |
| Cluster 20 | Ladderane-Arylpolyene-Nrps | 3747228 | 3873950 | Skyllamycin_biosynthetic_gene_cluster (46% of genes show similarity) | BGC0000429_c1 | 2 | CYP163B5; CYP107BZ1 |
| Cluster 21 | Lantipeptide | 4496390 | 4520962 | - | - |  |  |
| Cluster 22 | Melanin | 4960832 | 4971221 | Grixazone_biosynthetic_gene_cluster (76% of genes show similarity) | BGC0000662_c1 |  |  |
| Cluster 23 | Thiopeptide | 5142038 | 5174689 | - | - |  |  |
| Cluster 24 | Siderophore | 5573822 | 5585600 | Desferrioxamine_B_biosynthetic_gene_cluster (100% of genes show similarity) | BGC0000941_c1 |  |  |
| Cluster 25 | Lantipeptide | 5645167 | 5668604 | - | - |  |  |
| Cluster 26 | Otherks | 6216239 | 6257198 | Salinilactam_biosynthetic_gene_cluster (8% of genes show similarity) | BGC0000142_c1 |  |  |
| Cluster 27 | Ectoine | 6603626 | 6614024 | Ectoine_biosynthetic_gene_cluster (100% of genes show similarity) | BGC0000853_c1 |  |  |
| Cluster 28 | Amglyccycl | 6935101 | 6958692 | Streptomycin_biosynthetic_gene_cluster (26% of genes show similarity) | BGC0000717_c1 |  |  |
| Cluster 29 | T1pks-Terpene | 7069072 | 7191428 | BE-7585A_biosynthetic_gene_cluster (23% of genes show similarity) | BGC0000203_c1 |  |  |
| Cluster 30 | T1pks | 7270248 | 7372684 | Tautomycin_biosynthetic_gene_cluster (17% of genes show similarity) | BGC0000159_c1 |  |  |
| Cluster 31 | T1pks-Linaridin | 7552138 | 7639551 | Grisemycin_biosynthetic_gene_cluster (100% of genes show similarity) | BGC0000583_c1 |  |  |
| Cluster 32 | T3pks | 7888518 | 7929636 | Herboxidiene_biosynthetic_gene_cluster (6% of genes show similarity) | BGC0001065_c1 | 1 | CYP107F4 |
| Cluster 33 | Transatpks-T1pks-Otherks-Nrps | 7995928 | 8182236 | Griseobactin_biosynthetic_gene_cluster (94% of genes show similarity) | BGC0000368_c1 | 1 | CYP107CA2 |
| Cluster 34 | Terpene | 8196573 | 8236655 | Carotenoid_biosynthetic_gene_cluster (100% of genes show similarity) | BGC0000649_c1 |  |  |
| Cluster 35 | Butyrolactone | 8268444 | 8279388 | Coelimycin_biosynthetic_gene_cluster (8% of genes show similarity) | BGC0000038_c1 |  |  |
| Cluster 36 | Terpene | 8473224 | 8498819 | Isorenieratene_biosynthetic_gene_cluster (100% of genes show similarity) | BGC0000664_c1 |  |  |
| ***Streptomyces globisporus* C-1027** | | | | | | | |
| Cluster 1 | Terpene | 104229 | 125263 | A54145_biosynthetic_gene_cluster (3% of genes show similarity) | BGC0000291_c1 |  |  |
| Cluster 2 | Butyrolactone | 177217 | 188161 | Coelimycin_biosynthetic_gene_cluster (16% of genes show similarity) | BGC0000038_c1 |  |  |
| Cluster 3 | Terpene | 222188 | 244401 | - | - |  |  |
| Cluster 4 | Terpene-Nrps | 284764 | 451409 | Griseobactin_biosynthetic_gene_cluster (100% of genes show similarity) | BGC0000368_c1 |  |  |
| Cluster 5 | T3pks | 472394 | 513512 | Lasalocid_biosynthetic_gene_cluster (9% of genes show similarity) | BGC0000087_c1 | 1 | CYP107F4 |
| Cluster 6 | Terpene-Nrps | 844594 | 917380 | Isorenieratene_biosynthetic_gene_cluster (85% of genes show similarity) | BGC0000664_c1 |  |  |
| Cluster 7 | Terpene | 1181612 | 1202688 | Steffimycin_biosynthetic_gene_cluster (19% of genes show similarity) | BGC0000273_c1 |  |  |
| Cluster 8 | Ectoine | 1631082 | 1641480 | Ectoine_biosynthetic_gene_cluster (100% of genes show similarity) | BGC0000853_c1 |  |  |
| Cluster 9 | Lantipeptide | 2652852 | 2675896 | - | - |  |  |
| Cluster 10 | Siderophore | 2742719 | 2754497 | Desferrioxamine_B_biosynthetic_gene_cluster (100% of genes show similarity) | BGC0000941_c1 |  |  |
| Cluster 11 | Thiopeptide | 3122859 | 3151721 | - | - |  |  |
| Cluster 12 | Nrps | 3335176 | 3400695 | Oxazolomycin_biosynthetic_gene_cluster (6% of genes show similarity) | BGC0001106_c1 |  |  |
| Cluster 13 | Lassopeptide | 4463970 | 4486667 | SRO15-2005_biosynthetic_gene_cluster (100% of genes show similarity) | BGC0000578_c1 |  |  |
| Cluster 14 | Lantipeptide | 4552589 | 4594559 | Labyrinthopeptin_A1,A3_/_labyrinthopeptin_A2_biosynthetic_gene (40% of genes show similarity) | BGC0000519_c1 |  |  |
| Cluster 15 | T1pks-Nrps | 5167813 | 5222510 | Leinamycin_biosynthetic_gene_cluster (4% of genes show similarity) | BGC0001101_c1 |  |  |
| Cluster 16 | Lantipeptide | 5299671 | 5322418 | AmfS_biosynthetic_gene_cluster (100% of genes show similarity) | BGC0000496_c1 |  |  |
| Cluster 17 | Ectoine | 5532667 | 5543041 | Pristinamycin_biosynthetic_gene_cluster (23% of genes show similarity) | BGC0000952_c2 |  |  |
| Cluster 18 | Terpene | 5664830 | 5685807 | - | - |  |  |
| Cluster 19 | Siderophore | 6082612 | 6097344 | - | - |  |  |
| Cluster 20 | Bacteriocin | 6331423 | 6342799 | - | - |  |  |
| Cluster 21 | Ectoine | 6564225 | 6574599 | Kosinostatin_biosynthetic_gene_cluster (11% of genes show similarity) | BGC0001073_c1 |  |  |
| Cluster 22 | Arylpolyene-Ladderane | 6765985 | 6819639 | Skyllamycin_biosynthetic_gene_cluster (24% of genes show similarity) | BGC0000429_c1 | 1 | CYP1373A2 |
| Cluster 23 | Terpene | 6959815 | 6986388 | Hopene_biosynthetic_gene_cluster (69% of genes show similarity) | BGC0000663_c1 |  |  |
| Cluster 24 | Bacteriocin-T1pks-Nrps | 7040610 | 7115186 | SGR_PTMs_biosynthetic_gene_cluster (100% of genes show similarity) | BGC0001043_c1 | 1 | CYP107BX10 |
| Cluster 25 | Melanin | 7287746 | 7298213 | Istamycin_biosynthetic_gene_cluster (4% of genes show similarity) | BGC0000700_c1 | 1 | CYP124G14 |
| Cluster 26 | T3pks | 7337452 | 7378504 | Tetronasin_biosynthetic_gene_cluster (11% of genes show similarity) | BGC0000163_c1 |  |  |
| Cluster 27 | T1pks-Nrps | 7483759 | 7536082 | Daptomycin_biosynthetic_gene_cluster (7% of genes show similarity) | BGC0000336_c1 | 1 | CYP105D30 |
| ***Streptomyces scabiei* 87.22** | | | | | | | |
| Cluster 1 | Nrps | 121440 | 196006 | Coelibactin_biosynthetic_gene_cluster (54% of genes show similarity) | BGC0000324_c1 |  |  |
| Cluster 2 | Nrps | 331163 | 401309 | Enduracidin_biosynthetic_gene_cluster (12% of genes show similarity) | BGC0000341_c1 |  |  |
| Cluster 3 | Lantipeptide | 401538 | 424192 | Labyrinthopeptin_A1,A3_/_labyrinthopeptin_A2_biosynthetic_gene (60% of genes show similarity) | BGC0000519_c1 |  |  |
| Cluster 4 | Terpene | 560917 | 582239 | 2-methylisoborneol_biosynthetic_gene_cluster (100% of genes show similarity) | BGC0000658_c1 |  |  |
| Cluster 5 | Terpene | 601915 | 628277 | Carotenoid_biosynthetic_gene_cluster (63% of genes show similarity) | BGC0000633_c1 |  |  |
| Cluster 6 | Bacteriocin-Lantipeptide | 958478 | 990742 | Informatipeptin_biosynthetic_gene_cluster (85% of genes show similarity) | BGC0000518_c1 |  |  |
| Cluster 7 | Butyrolactone | 1354264 | 1365265 | Calcimycin_biosynthetic_gene_cluster (10% of genes show similarity) | BGC0000032_c1 |  |  |
| Cluster 8 | Terpene | 1443175 | 1469862 | Hopene_biosynthetic_gene_cluster (92% of genes show similarity) | BGC0000663_c1 |  |  |
| Cluster 9 | Siderophore | 2066419 | 2079787 | Grincamycin_biosynthetic_gene_cluster (8% of genes show similarity) | BGC0000229_c1 |  |  |
| Cluster 10 | Other | 2213586 | 2256048 | Medermycin_biosynthetic_gene_cluster (13% of genes show similarity) | BGC0000245_c1 |  |  |
| Cluster 11 | Terpene | 2272242 | 2294449 | Meridamycin_biosynthetic_gene_cluster (40% of genes show similarity) | BGC0001012_c1 | 1 | CYP154A4 |
| Cluster 12 | Bacteriocin | 2343619 | 2354977 | - | - |  |  |
| Cluster 13 | Terpene | 2615765 | 2637636 | FD-594_biosynthetic_gene_cluster (6% of genes show similarity) | BGC0000222_c1 |  |  |
| Cluster 14 | Siderophore | 2788855 | 2800942 | - | - |  |  |
| Cluster 15 | Lantipeptide-Nrps | 3580343 | 3663950 | Thaxtomin_biosynthetic_gene_cluster (54% of genes show similarity) | BGC0000444_c1 | 4 | CYP246A1; CYP1048A1; CYP156D1; CYP154L1 |
| Cluster 16 | T2pks | 4846105 | 4888608 | Spore_pigment_biosynthetic_gene_cluster (83% of genes show similarity) | BGC0000271_c1 |  |  |
| Cluster 17 | T1pks-Nrps | 4907380 | 4959979 | Herboxidiene_biosynthetic_gene_cluster (2% of genes show similarity) | BGC0001065_c1 | 1 | CYP107AM1 |
| Cluster 18 | Lantipeptide | 5327449 | 5350019 | - | - |  |  |
| Cluster 19 | Bacteriocin-Bottromycin | 6296673 | 6328951 | Bottromycin_A2_biosynthetic_gene_cluster (75% of genes show similarity) | BGC0000469_c1 | 1 | CYP283A1 |
| Cluster 20 | Siderophore | 6440872 | 6452644 | Desferrioxamine_B_biosynthetic_gene_cluster (83% of genes show similarity) | BGC0000940_c1 |  |  |
| Cluster 21 | Melanin | 6583132 | 6593572 | Melanin_biosynthetic_gene_cluster (100% of genes show similarity) | BGC0000909_c1 |  |  |
| Cluster 22 | Butyrolactone-T1pks-Otherks | 6948618 | 7031953 | RK-682_biosynthetic_gene_cluster (45% of genes show similarity) | BGC0000140_c1 | 1 | CYP107AL1 |
| Cluster 23 | Ectoine | 7828037 | 7838435 | Ectoine_biosynthetic_gene_cluster (100% of genes show similarity) | BGC0000853_c1 |  |  |
| Cluster 24 | Other | 8049774 | 8093640 | Pristinamycin_biosynthetic_gene_cluster (2% of genes show similarity) | BGC0000952_c3 |  |  |
| Cluster 25 | Terpene | 8145730 | 8166746 | - | - | 1 | CYP157C5 |
| Cluster 26 | Indole-T1pks | 8700511 | 8773617 | Fortimicin_biosynthetic_gene_cluster (6% of genes show similarity) | BGC0000695_c1 | 1 | CYP156B2 |
| Cluster 27 | T1pks | 8793133 | 8848674 | Lasalocid_biosynthetic_gene_cluster (11% of genes show similarity) | BGC0000087_c1 | 1 | CYP107AK1 |
| Cluster 28 | Terpene | 9109494 | 9130543 | Oxazolomycin_biosynthetic_gene_cluster (6% of genes show similarity) | BGC0001106_c1 |  |  |
| Cluster 29 | T1pks-Linaridin | 9288856 | 9423896 | Concanamycin_A_biosynthetic_gene_cluster (92% of genes show similarity) | BGC0000040_c1 |  |  |
| Cluster 30 | Siderophore | 9430375 | 9443981 | - | - |  |  |
| Cluster 31 | Melanin-Nrps | 9526250 | 9606840 | Scabichelin_biosynthetic_gene_cluster (100% of genes show similarity) | BGC0000423_c1 |  |  |
| Cluster 32 | Terpene | 9618596 | 9639636 | - | - |  |  |
| ***Streptomyces sp.* SirexAA-E** | | | | | | | |
| Cluster 1 | Nrps | 183422 | 233810 | Thiocoraline_biosynthetic_gene_cluster (10% of genes show similarity) | BGC0000445_c1 |  |  |
| Cluster 2 | T2pks-Terpene | 248129 | 297390 | Spore_pigment_biosynthetic_gene_cluster (83% of genes show similarity) | BGC0000271_c1 |  |  |
| Cluster 3 | Melanin | 333103 | 343633 | Melanin_biosynthetic_gene_cluster (100% of genes show similarity) | BGC0000911_c1 | 1 | CYP124G3 |
| Cluster 4 | Nrps | 689835 | 740721 | Coelichelin_biosynthetic_gene_cluster (100% of genes show similarity) | BGC0000325_c1 |  |  |
| Cluster 5 | Nrps | 809951 | 864046 | Coelibactin_biosynthetic_gene_cluster (100% of genes show similarity) | BGC0000324_c1 | 1 | CYP105N1 |
| Cluster 6 | Terpene | 989127 | 1010161 | Steffimycin_biosynthetic_gene_cluster (19% of genes show similarity) | BGC0000273_c1 |  |  |
| Cluster 7 | Ectoine | 1441158 | 1451556 | Ectoine_biosynthetic_gene_cluster (100% of genes show similarity) | BGC0000853_c1 |  |  |
| Cluster 8 | Otherks | 1930630 | 1983417 | Nataxazole_biosynthetic_gene_cluster (25% of genes show similarity) | BGC0001213_c1 |  |  |
| Cluster 9 | Otherks | 2409002 | 2449961 | Meilingmycin_biosynthetic_gene_cluster (2% of genes show similarity) | BGC0000093_c1 |  |  |
| Cluster 10 | Lantipeptide | 2462308 | 2485445 | - | - |  |  |
| Cluster 11 | Butyrolactone | 3534233 | 3545165 | Lactonamycin_biosynthetic_gene_cluster (3% of genes show similarity) | BGC0000238_c1 |  |  |
| Cluster 12 | Terpene | 5143473 | 5164480 | Carbapenem_MM_4550_biosynthetic_gene_cluster (10% of genes show similarity) | BGC0000842_c1 |  |  |
| Cluster 13 | Siderophore | 5511501 | 5526094 | - | - |  |  |
| Cluster 14 | Nrps | 5617605 | 5679551 | WAP-8294A2_(lotilibcin)_biosynthetic_gene_cluster (30% of genes show similarity) | BGC0000461_c1 |  |  |
| Cluster 15 | Terpene | 5745560 | 5771085 | Isorenieratene_biosynthetic_gene_cluster (100% of genes show similarity) | BGC0000664_c1 |  |  |
| Cluster 16 | T2pks-T1pks-Otherks | 5841512 | 5903128 | Hedamycin_biosynthetic_gene_cluster (15% of genes show similarity) | BGC0000233_c1 | 2 | CYP181A1; CYP107Y1 |
| Cluster 17 | Bacteriocin | 5953056 | 5964366 | - | - |  |  |
| Cluster 18 | T1pks | 6260096 | 6354741 | Vicenistatin_biosynthetic_gene_cluster (60% of genes show similarity) | BGC0000167_c1 | 2 | CYP105AZ1; CYP105AZ2 |
| Cluster 19 | Terpene | 6577011 | 6599080 | Leinamycin_biosynthetic_gene_cluster (2% of genes show similarity) | BGC0001101_c1 |  |  |
| Cluster 20 | Terpene | 6730096 | 6756683 | Hopene_biosynthetic_gene_cluster (69% of genes show similarity) | BGC0000663_c1 |  |  |
| Cluster 21 | T1pks-Nrps | 6922336 | 6971791 | SGR_PTMs_biosynthetic_gene_cluster (100% of genes show similarity) | BGC0001043_c1 | 1 | CYP107BX4 |
| Cluster 22 | T2pks-Butyrolactone-Nrps | 7145734 | 7230746 | Auricin_biosynthetic_gene_cluster (41% of genes show similarity) | BGC0000201_c1 | 1 | CYP105A4 |
| ***Streptomyces violaceusniger* Tu 4113** | | | | | | | |
| Cluster 1 | Siderophore | 886115 | 897905 | Desferrioxamine_B_biosynthetic_gene_cluster (100% of genes show similarity) | BGC0000940_c1 |  |  |
| Cluster 2 | Lantipeptide | 1145837 | 1170458 | - | - |  |  |
| Cluster 3 | Other | 1450857 | 1493703 | Echosides_biosynthetic_gene_cluster (100% of genes show similarity) | BGC0000340_c1 |  |  |
| Cluster 4 | Siderophore | 2078339 | 2090252 | - | - |  |  |
| Cluster 5 | Bacteriocin | 2301044 | 2312369 | - | - |  |  |
| Cluster 6 | T2pks | 2543707 | 2586222 | Spore_pigment_biosynthetic_gene_cluster (75% of genes show similarity) | BGC0000271_c1 |  |  |
| Cluster 7 | Terpene | 2936974 | 2963422 | Hopene_biosynthetic_gene_cluster (76% of genes show similarity) | BGC0000663_c1 | 2 | CYP107BW1; CYP1013A2 |
| Cluster 8 | Lantipeptide | 3160291 | 3199125 | BE-7585A_biosynthetic_gene_cluster (23% of genes show similarity) | BGC0000203_c1 |  |  |
| Cluster 9 | Nrps | 3219941 | 3299320 | Lipopeptide_8D1-1_/_lipopeptide_8D1-2_biosynthetic_gene_clus... (6% of genes show similarity) | BGC0001370_c1 | 2 | CYP162A3; CYP107CK1 |
| Cluster 10 | Lassopeptide | 3364827 | 3387368 | SSV-2083_biosynthetic_gene_cluster (50% of genes show similarity) | BGC0000579_c1 |  |  |
| Cluster 11 | T1pks | 3419279 | 3467405 | - | - |  |  |
| Cluster 12 | Butyrolactone | 3770063 | 3780995 | Meilingmycin_biosynthetic_gene_cluster (2% of genes show similarity) | BGC0000093_c1 |  |  |
| Cluster 13 | Hserlactone | 3791483 | 3812241 | Daptomycin_biosynthetic_gene_cluster (3% of genes show similarity) | BGC0000336_c1 |  |  |
| Cluster 14 | T1pks | 3977601 | 4057790 | Elaiophylin_biosynthetic_gene_cluster (87% of genes show similarity) | BGC0000053_c1 |  |  |
| Cluster 15 | T1pks | 4154756 | 4201139 | Salinomycin_biosynthetic_gene_cluster (8% of genes show similarity) | BGC0000144_c1 |  |  |
| Cluster 16 | T1pks | 4202037 | 4333901 | Nigericin_biosynthetic_gene_cluster (100% of genes show similarity) | BGC0000114_c1 | 1 | CYP124B3 |
| Cluster 17 | T1pks | 4514441 | 4670396 | ECO-02301_biosynthetic_gene_cluster (39% of genes show similarity) | BGC0000052_c1 | 2 | CYP107BW1; CYP105AX1 |
| Cluster 18 | T1pks-Nrps | 5128359 | 5213730 | Actinomycin_biosynthetic_gene_cluster (10% of genes show similarity) | BGC0000296_c1 |  |  |
| Cluster 19 | T1pks-Nrps | 5224048 | 5312355 | Naphthomycin_biosynthetic_gene_cluster (15% of genes show similarity) | BGC0000106_c1 |  |  |
| Cluster 20 | Other | 5434110 | 5476848 | Echosides_biosynthetic_gene_cluster (11% of genes show similarity) | BGC0000340_c1 |  |  |
| Cluster 21 | Otherks | 5520939 | 5561958 | Galbonolides_biosynthetic_gene_cluster (20% of genes show similarity) | BGC0000065_c1 | 2 | CYP183F2; CYP105AV1 |
| Cluster 22 | Terpene | 5684250 | 5706013 | Brasilicardin_A_biosynthetic_gene_cluster (36% of genes show similarity) | BGC0000632_c1 |  |  |
| Cluster 23 | Bacteriocin-Lantipeptide-T1pks-Otherks-Nrps | 5741925 | 5876020 | Leinamycin_biosynthetic_gene_cluster (5% of genes show similarity) | BGC0001101_c1 | 4 | CYP105AQ2; CYP155A5; CYP107E9; CYP105AN3 |
| Cluster 24 | Other | 5927077 | 5968123 | Herboxidiene_biosynthetic_gene_cluster (3% of genes show similarity) | BGC0001065_c1 |  |  |
| Cluster 25 | T1pks | 6014424 | 6098236 | Hygrocin_biosynthetic_gene_cluster (83% of genes show similarity) | BGC0000075_c1 | 1 | CYP107AD1 |
| Cluster 26 | Lantipeptide-T1pks-Nrps | 6091811 | 6215900 | Meridamycin_biosynthetic_gene_cluster (52% of genes show similarity) | BGC0001011_c1 | 1 | CYP105AY1 |
| Cluster 27 | Terpene | 6421015 | 6442313 | 2-methylisoborneol_biosynthetic_gene_cluster (100% of genes show similarity) | BGC0000658_c1 |  |  |
| Cluster 28 | Terpene | 6555062 | 6576216 | - | - |  |  |
| Cluster 29 | T1pks | 6675986 | 6758493 | Herbimycin_biosynthetic_gene_cluster (66% of genes show similarity) | BGC0000074_c1 | 1 | CYP105U1 |
| Cluster 30 | T1pks-Otherks | 7181560 | 7243764 | Meilingmycin_biosynthetic_gene_cluster (2% of genes show similarity) | BGC0000093_c1 |  |  |
| Cluster 31 | T1pks | 7437179 | 7552962 | Reveromycin_biosynthetic_gene_cluster (36% of genes show similarity) | BGC0000135_c1 |  |  |
| Cluster 32 | T1pks-Siderophore | 7736179 | 7814751 | Apoptolidin_biosynthetic_gene_cluster (23% of genes show similarity) | BGC0000021_c1 |  |  |
| Cluster 33 | Ectoine | 7966340 | 7976744 | Ectoine_biosynthetic_gene_cluster (100% of genes show similarity) | BGC0000853_c1 |  |  |
| Cluster 34 | Terpene | 8459184 | 8480494 | - | - | 2 | CYP156C9; CYP125A20 |
| Cluster 35 | Lantipeptide | 8814684 | 8837881 | Actagardine_biosynthetic_gene_cluster (9% of genes show similarity) | BGC0000495_c1 |  |  |
| Cluster 36 | T1pks | 9065094 | 9247443 | ECO-02301_biosynthetic_gene_cluster (57% of genes show similarity) | BGC0000052_c1 |  |  |
| Cluster 37 | Ladderane | 9434566 | 9475807 | WS9326_biosynthetic_gene_cluster (25% of genes show similarity) | BGC0001297_c1 |  |  |
| Cluster 38 | Nrps | 9526467 | 9570633 | Ochronotic_pigment_biosynthetic_gene_cluster (75% of genes show similarity) | BGC0000918_c1 |  |  |
| Cluster 39 | Indole | 9805824 | 9826966 | 7-prenylisatin_biosynthetic_gene_cluster (40% of genes show similarity) | BGC0001294_c1 | 2 | CYP107U9; CYP156B6 |
| Cluster 40 | Ladderane-Arylpolyene-Nrps | 10111293 | 10214141 | Skyllamycin_biosynthetic_gene_cluster (46% of genes show similarity) | BGC0000429_c1 | 3 | CYP163B4; CYP107CF1; CYP107CE1 |
| Cluster 41 | Terpene | 10465383 | 10487659 | Desotamide_biosynthetic_gene_cluster (9% of genes show similarity) | BGC0001196_c1 | 1 | CYP147F5 |
| ***Streptomyces cattleya* NRRL 8057** | | | | | | | |
| Cluster 1 | Butyrolactone | 1 | 6680 | - | - |  |  |
| Cluster 2 | T1pks-Lassopeptide | 32796 | 99728 | Mannopeptimycin_biosynthetic_gene_cluster (11% of genes show similarity) | BGC0000388_c1 |  |  |
| Cluster 3 | T1pks | 129575 | 276643 | Akaeolide_biosynthetic_gene_cluster (28% of genes show similarity) | BGC0001199_c1 | 2 | CYP107AS; CYP107CR1 |
| Cluster 4 | Terpene | 570378 | 591112 | - | - |  |  |
| Cluster 5 | Lantipeptide | 626485 | 651045 | - | - |  |  |
| Cluster 6 | Other | 968655 | 1009491 | Pyrrolomycin_biosynthetic_gene_cluster (5% of genes show similarity) | BGC0000130_c1 |  |  |
| Cluster 7 | Ectoine | 1153014 | 1163418 | Ectoine_biosynthetic_gene_cluster (100% of genes show similarity) | BGC0000853_c1 |  |  |
| Cluster 8 | Lantipeptide | 1527378 | 1550026 | AmfS_biosynthetic_gene_cluster (80% of genes show similarity) | BGC0000496_c1 |  |  |
| Cluster 9 | Butyrolactone | 2315622 | 2326407 | Rabelomycin_biosynthetic_gene_cluster (6% of genes show similarity) | BGC0000262_c1 |  |  |
| Cluster 10 | T3pks | 2334065 | 2375096 | - | - |  |  |
| Cluster 11 | Nrps | 2784016 | 2856738 | Salinosporamide_biosynthetic_gene_cluster (19% of genes show similarity) | BGC0001041_c1 |  |  |
| Cluster 12 | Lantipeptide | 2936415 | 2965706 | Kanamycin_biosynthetic_gene_cluster (1% of genes show similarity) | BGC0000703_c1 | 1 | CYP107AE6 |
| Cluster 13 | Terpene | 3092166 | 3114391 | - | - |  |  |
| Cluster 14 | T1pks-Nrps | 3416953 | 3486060 | Akaeolide_biosynthetic_gene_cluster (20% of genes show similarity) | BGC0001199_c1 | 1 | CYP184A4 |
| Cluster 15 | Transatpks-T1pks-Nrps | 3784857 | 3878760 | Kirromycin_biosynthetic_gene_cluster (18% of genes show similarity) | BGC0001070_c1 | 2 | CYP107CS1; CYP107W2 |
| Cluster 16 | Other | 4554858 | 4595580 | Oviedomycin_biosynthetic_gene_cluster (12% of genes show similarity) | BGC0000253_c1 |  |  |
| Cluster 17 | Siderophore | 4913457 | 4928849 | - | - |  |  |
| Cluster 18 | Bacteriocin | 5057506 | 5068354 | - | - |  |  |
| Cluster 19 | Terpene | 5321497 | 5348209 | Hopene_biosynthetic_gene_cluster (76% of genes show similarity) | BGC0000663_c1 |  |  |
| Cluster 20 | T3pks-Terpene | 5403543 | 5473532 | R1128_biosynthetic_gene_cluster (21% of genes show similarity) | BGC0000261_c1 | 1 | CYP158A13 |
| Cluster 21 | Other | 5972017 | 6015850 | Paromomycin_biosynthetic_gene_cluster (10% of genes show similarity) | BGC0000712_c1 |  |  |
| Cluster 22 | Nrps-Otherks | 6074191 | 6141816 | Cephamycin_C_biosynthetic_gene_cluster (73% of genes show similarity) | BGC0000319_c1 |  |  |
| Cluster 23 | Lantipeptide | 6142053 | 6171709 | - | - | 1 | CYP105AA10 |
| Cluster 24 | T1pks-Butyrolactone-Nrps | 6163651 | 6250787 | U-68204_biosynthetic_gene_cluster (64% of genes show similarity) | BGC0001355_c1 | 2 | CYP107CT1; CYP105B25 |
| ***Streptomyces cattleya* NRRL 8058 = DSM 46488** | | | | | | | |
| Cluster 1 | Butyrolactone | 1 | 6681 | Chrysomycin_biosynthetic_gene_cluster (5% of genes show similarity) | BGC0000211_c1 |  |  |
| Cluster 2 | T1pks-Lassopeptide | 32797 | 99729 | Mannopeptimycin_biosynthetic_gene_cluster (11% of genes show similarity) | BGC0000388_c1 |  |  |
| Cluster 3 | T1pks | 129576 | 276592 | Hygrocin_biosynthetic_gene_cluster (22% of genes show similarity) | BGC0000075_c1 | 3 | CYP107AS; CYP1274A1; CYP107CR1 |
| Cluster 4 | Terpene | 570471 | 591205 | - | - |  |  |
| Cluster 5 | Lantipeptide | 626578 | 651138 | - | - |  |  |
| Cluster 6 | Other | 968747 | 1009583 | Pyrrolomycin_biosynthetic_gene_cluster (5% of genes show similarity) | BGC0000130_c1 |  |  |
| Cluster 7 | Ectoine | 1152703 | 1163107 | Ectoine_biosynthetic_gene_cluster (100% of genes show similarity) | BGC0000853_c1 |  |  |
| Cluster 8 | Lantipeptide | 1526812 | 1549460 | AmfS_biosynthetic_gene_cluster (80% of genes show similarity) | BGC0000496_c1 |  |  |
| Cluster 9 | Butyrolactone | 2315078 | 2326091 | Rabelomycin_biosynthetic_gene_cluster (6% of genes show similarity) | BGC0000262_c1 |  |  |
| Cluster 10 | T3pks | 2333868 | 2374899 | - | - |  |  |
| Cluster 11 | Nrps | 2783826 | 2856549 | Stenothricin_biosynthetic_gene_cluster (31% of genes show similarity) | BGC0000431_c1 |  |  |
| Cluster 12 | Lantipeptide | 2936367 | 2965589 | Kanamycin_biosynthetic_gene_cluster (1% of genes show similarity) | BGC0000703_c1 | 1 | CYP107AE6 |
| Cluster 13 | Terpene | 3092046 | 3114271 | - | - |  |  |
| Cluster 14 | T1pks-Nrps | 3416761 | 3485868 | Akaeolide_biosynthetic_gene_cluster (20% of genes show similarity) | BGC0001199_c1 | 1 | CYP184A4 |
| Cluster 15 | Transatpks-T1pks-Nrps | 3784664 | 3878568 | Kirromycin_biosynthetic_gene_cluster (20% of genes show similarity) | BGC0001070_c1 | 2 | CYP107CS1; CYP107W2 |
| Cluster 16 | Bacteriocin | 4006188 | 4016460 | - | - |  |  |
| Cluster 17 | Other | 4554666 | 4595388 | Oviedomycin_biosynthetic_gene_cluster (12% of genes show similarity) | BGC0000253_c1 |  |  |
| Cluster 18 | Siderophore | 4913334 | 4928699 | - | - |  |  |
| Cluster 19 | Bacteriocin | 5057400 | 5068248 | - | - |  |  |
| Cluster 20 | Terpene | 5321391 | 5348103 | Hopene_biosynthetic_gene_cluster (76% of genes show similarity) | BGC0000663_c1 |  |  |
| Cluster 21 | T3pks-Terpene | 5403437 | 5473426 | R1128_biosynthetic_gene_cluster (21% of genes show similarity) | BGC0000261_c1 | 1 | CYP158A13 |
| Cluster 22 | Other | 5972016 | 6015924 | Paromomycin_biosynthetic_gene_cluster (7% of genes show similarity) | BGC0000712_c1 |  |  |
| Cluster 23 | Nrps-Otherks | 6074210 | 6141835 | Cephamycin_C_biosynthetic_gene_cluster (73% of genes show similarity) | BGC0000319_c1 |  |  |
| Cluster 24 | Lantipeptide | 6142071 | 6171727 | - | - | 1 | CYP105AA10 |
| Cluster 25 | T1pks-Butyrolactone-Nrps | 6163675 | 6251650 | U-68204_biosynthetic_gene_cluster (64% of genes show similarity) | BGC0001355_c1 | 2 | CYP107CT1; CYP105B25 |
| ***Streptomyces pratensis/flavogriseus* IAF 45** | | | | | | | |
| Cluster 1 | Blactam-T1pks-Nrps | 70873 | 221343 | Carbapenem_MM_4550_biosynthetic_gene_cluster (65% of genes show similarity) | BGC0000842_c1 | 2 | CYP247A3; CYP107BX5 |
| Cluster 2 | Nrps | 327693 | 378596 | Coelichelin_biosynthetic_gene_cluster (90% of genes show similarity) | BGC0000325_c1 |  |  |
| Cluster 3 | Terpene | 393138 | 418630 | Isorenieratene_biosynthetic_gene_cluster (100% of genes show similarity) | BGC0000664_c1 |  |  |
| Cluster 4 | Blactam | 634715 | 658209 | Clavulanic_acid_biosynthetic_gene_cluster (20% of genes show similarity) | BGC0000845_c1 |  |  |
| Cluster 5 | Terpene | 738599 | 765184 | Hopene_biosynthetic_gene_cluster (69% of genes show similarity) | BGC0000663_c1 |  |  |
| Cluster 6 | T1pks | 1104244 | 1198984 | Vicenistatin_biosynthetic_gene_cluster (60% of genes show similarity) | BGC0000167_c1 | 2 | CYP105AZ2; CYP105AZ1 |
| Cluster 7 | Bacteriocin | 1409256 | 1420548 | - | - |  |  |
| Cluster 8 | Nrps | 1560425 | 1622443 | Arylomycin_biosynthetic_gene_cluster (22% of genes show similarity) | BGC0000306_c1 |  |  |
| Cluster 9 | Siderophore | 1894420 | 1909004 | - | - |  |  |
| Cluster 10 | Terpene | 1973589 | 1994659 | - | - |  |  |
| Cluster 11 | Bacteriocin | 2151745 | 2161966 | - | - |  |  |
| Cluster 12 | Butyrolactone | 3819031 | 3829957 | Lactonamycin_biosynthetic_gene_cluster (3% of genes show similarity) | BGC0000238_c1 |  |  |
| Cluster 13 | T1pks-Nrps | 4023172 | 4080102 | Istamycin_biosynthetic_gene_cluster (11% of genes show similarity) | BGC0000700_c1 | 3 | CYP1029A2; CYP1423A2; CYP285A2 |
| Cluster 14 | Siderophore | 4727646 | 4739427 | Desferrioxamine_B_biosynthetic_gene_cluster (83% of genes show similarity) | BGC0000940_c1 |  |  |
| Cluster 15 | Lantipeptide | 4792335 | 4815403 | - | - |  |  |
| Cluster 16 | Terpene | 5226078 | 5247109 | - | - | 1 | CYP157K1 |
| Cluster 17 | Ectoine | 5705549 | 5715947 | Ectoine_biosynthetic_gene_cluster (100% of genes show similarity) | BGC0000853_c1 |  |  |
| Cluster 18 | T2pks-Otherks | 6079056 | 6136319 | Cinerubin_B_biosynthetic_gene_cluster (28% of genes show similarity) | BGC0000212_c1 |  |  |
| Cluster 19 | Terpene | 6181067 | 6202113 | Steffimycin_biosynthetic_gene_cluster (19% of genes show similarity) | BGC0000273_c1 |  |  |
| Cluster 20 | Ectoine-Terpene | 6482662 | 6509101 | Ectoine_biosynthetic_gene_cluster (100% of genes show similarity) | BGC0000853_c1 |  |  |
| Cluster 21 | Bacteriocin | 6532212 | 6542439 | - | - |  |  |
| Cluster 22 | T3pks | 6695745 | 6736803 | Tetronasin_biosynthetic_gene_cluster (11% of genes show similarity) | BGC0000163_c1 |  |  |
| Cluster 23 | Melanin | 6830784 | 6841248 | Melanin_biosynthetic_gene_cluster (100% of genes show similarity) | BGC0000911_c1 | 1 | CYP124G4 |
| Cluster 24 | T2pks-Terpene | 6882032 | 6931236 | Spore_pigment_biosynthetic_gene_cluster (83% of genes show similarity) | BGC0000271_c1 |  |  |
| Cluster 25 | Nrps | 7082613 | 7132319 | Zorbamycin_biosynthetic_gene_cluster (6% of genes show similarity) | BGC0001058_c1 | 2 | CYP1035A4; CYP156B9 |
| Cluster 26 | Butyrolactone | 7211688 | 7222623 | - | - |  |  |
| ***Streptomyces bingchenggensis*** | | | | | | | |
| Cluster 1 | Bacteriocin-T1pks-Nrps | 115407 | 214376 | Leinamycin_biosynthetic_gene_cluster (4% of genes show similarity) | BGC0001101_c1 | 2 | CYP183C1; CYP183D1 |
| Cluster 2 | Terpene | 229745 | 251688 | Actinomycin_biosynthetic_gene_cluster (25% of genes show similarity) | BGC0000296_c1 | 1 | CYP183E1 |
| Cluster 3 | Transatpks-Otherks-Nrps | 330056 | 401421 | Heterocyst_glycolipids_biosynthetic_gene_cluster (28% of genes show similarity) | BGC0000869_c1 |  |  |
| Cluster 4 | Bacteriocin-Lantipeptide-T1pks | 583067 | 635091 | Tetronasin_biosynthetic_gene_cluster (5% of genes show similarity) | BGC0000163_c1 | 1 | CYP1039A1 |
| Cluster 5 | Transatpks-T1pks-Nrps | 766606 | 1101234 | Meilingmycin_biosynthetic_gene_cluster (54% of genes show similarity) | BGC0000093_c1 | 3 | CYP105H6; CYP107BK1; CYP171A2 |
| Cluster 6 | T1pks | 1126684 | 1179715 | Meilingmycin_biosynthetic_gene_cluster (20% of genes show similarity) | BGC0000093_c1 |  |  |
| Cluster 7 | Nrps | 1181504 | 1228730 | Chloroeremomycin_biosynthetic_gene_cluster (6% of genes show similarity) | BGC0000322_c2 | 2 | CYP107BM3; CYP157B13 |
| Cluster 8 | Nrps-Furan-T1pks-Otherks | 1336592 | 1514066 | Lipopeptide_8D1-1_/_lipopeptide_8D1-2_biosynthetic_gene_clus... (11% of genes show similarity) | BGC0001370_c1 |  |  |
| Cluster 9 | Terpene | 1545363 | 1566433 | - | - |  |  |
| Cluster 10 | Other | 1572192 | 1632869 | Stenothricin_biosynthetic_gene_cluster (22% of genes show similarity) | BGC0000431_c1 |  |  |
| Cluster 11 | T1pks-Nrps | 1820646 | 1956768 | Lasalocid_biosynthetic_gene_cluster (24% of genes show similarity) | BGC0000087_c1 | 1 | CYP113G1 |
| Cluster 12 | T1pks-Otherks | 2057881 | 2122132 | Cremimycin_biosynthetic_gene_cluster (20% of genes show similarity) | BGC0000042_c1 |  |  |
| Cluster 13 | Terpene | 2182414 | 2203337 | Kinamycin_biosynthetic_gene_cluster (5% of genes show similarity) | BGC0000236_c1 |  |  |
| Cluster 14 | Other | 2477371 | 2520403 | Piericidin_A1_biosynthetic_gene_cluster (33% of genes show similarity) | BGC0001169_c1 | 1 | CYP154P1 |
| Cluster 15 | T1pks | 2625076 | 2746943 | Amphotericin_biosynthetic_gene_cluster (35% of genes show similarity) | BGC0000015_c1 |  |  |
| Cluster 16 | Terpene | 2813692 | 2835947 | Geosmin_biosynthetic_gene_cluster (100% of genes show similarity) | BGC0000661_c1 |  |  |
| Cluster 17 | T1pks-Nrps | 2969559 | 3026953 | Clarexpoxcin_biosynthetic_gene_cluster (26% of genes show similarity) | BGC0001203_c1 |  |  |
| Cluster 18 | Thiopeptide | 3030559 | 3059514 | - | - |  |  |
| Cluster 19 | Terpene | 3417796 | 3444497 | Hopene_biosynthetic_gene_cluster (76% of genes show similarity) | BGC0000663_c1 |  |  |
| Cluster 20 | Lassopeptide | 3518110 | 3545015 | Chaxapeptin_biosynthetic_gene_cluster (42% of genes show similarity) | BGC0001307_c1 |  |  |
| Cluster 21 | T2pks | 3622902 | 3665426 | Spore_pigment_biosynthetic_gene_cluster (83% of genes show similarity) | BGC0000271_c1 |  |  |
| Cluster 22 | Nrps | 3880299 | 3933590 | Calcium-dependent_antibiotic_biosynthetic_gene_cluster (17% of genes show similarity) | BGC0000315_c1 |  |  |
| Cluster 23 | Bacteriocin | 4012751 | 4024121 | - | - |  |  |
| Cluster 24 | Nrps | 4052289 | 4100532 | Enduracidin_biosynthetic_gene_cluster (8% of genes show similarity) | BGC0000341_c1 |  |  |
| Cluster 25 | T1pks | 4124810 | 4172660 | Borrelidin_biosynthetic_gene_cluster (9% of genes show similarity) | BGC0000031_c1 |  |  |
| Cluster 26 | Siderophore | 4305798 | 4317810 | - | - |  |  |
| Cluster 27 | Other | 4995984 | 5038752 | Echosides_biosynthetic_gene_cluster (88% of genes show similarity) | BGC0000340_c1 |  |  |
| Cluster 28 | T1pks-Nrps | 7330156 | 7378757 | Phosphonoglycans_biosynthetic_gene_cluster (3% of genes show similarity) | BGC0000806_c1 | 1 | CYP1037A1 |
| Cluster 29 | Siderophore-Nrps | 7742586 | 7808875 | Desferrioxamine_B_biosynthetic_gene_cluster (100% of genes show similarity) | BGC0000940_c1 |  |  |
| Cluster 30 | Arylpolyene-Ladderane | 8061655 | 8104092 | Skyllamycin_biosynthetic_gene_cluster (16% of genes show similarity) | BGC0000429_c1 |  |  |
| Cluster 31 | Nrps | 8115580 | 8170587 | Skyllamycin_biosynthetic_gene_cluster (16% of genes show similarity) | BGC0000429_c1 |  |  |
| Cluster 32 | T2pks-Oligosaccharide-Otherks | 8166510 | 8248974 | Saquayamycin_Z_/_galtamycin_B_biosynthetic_gene_cluster (47% of genes show similarity) | BGC0000267_c1 |  |  |
| Cluster 33 | Lantipeptide | 8306880 | 8331657 | - | - |  |  |
| Cluster 34 | Lantipeptide | 8346685 | 8375315 | SBI_06990_alpha_/_SBI_06989_beta_biosynthetic_gene_cluster (100% of genes show similarity) | BGC0001229_c1 |  |  |
| Cluster 35 | Otherks | 9124966 | 9166030 | Chlortetracycline_biosynthetic_gene_cluster (14% of genes show similarity) | BGC0000209_c1 | 1 | CYP268A4 |
| Cluster 36 | Ectoine | 9613076 | 9623480 | Ectoine_biosynthetic_gene_cluster (100% of genes show similarity) | BGC0000853_c1 |  |  |
| Cluster 37 | Siderophore | 9790988 | 9804831 | - | - |  |  |
| Cluster 38 | T1pks | 9898279 | 10029213 | Nanchangmycin_biosynthetic_gene_cluster (100% of genes show similarity) | BGC0000105_c1 | 1 | CYP124B2 |
| Cluster 39 | Butyrolactone | 10562794 | 10573729 | - | - |  |  |
| Cluster 40 | Terpene | 10597183 | 10618109 | Oxazolomycin_biosynthetic_gene_cluster (9% of genes show similarity) | BGC0001106_c1 |  |  |
| Cluster 41 | Other | 10715172 | 10757925 | Echosides_biosynthetic_gene_cluster (11% of genes show similarity) | BGC0000340_c1 |  |  |
| Cluster 42 | Lantipeptide | 10862642 | 10886007 | - | - |  |  |
| Cluster 43 | T1pks-Nrps | 10895566 | 10951243 | Herboxidiene_biosynthetic_gene_cluster (4% of genes show similarity) | BGC0001065_c1 |  |  |
| Cluster 44 | Otherks-Nrps | 10975893 | 11048987 | Himastatin_biosynthetic_gene_cluster (8% of genes show similarity) | BGC0001117_c1 | 1 | CYP163C1 |
| Cluster 45 | Other | 11070003 | 11112945 | - | - |  |  |
| Cluster 46 | Transatpks-Terpene-Nrps | 11442027 | 11575518 | Oxazolomycin_biosynthetic_gene_cluster (45% of genes show similarity) | BGC0001106_c1 | 2 | CYP161C1; CYP183A2 |
| Cluster 47 | T1pks | 11900331 | 11936683 | A54145_biosynthetic_gene_cluster (3% of genes show similarity) | BGC0000291_c1 |  |  |
| ***Streptomyces hygroscopicus* subsp. *jinggangensis* 5008** | | | | | | | |
| Cluster 1 | Amglyccycl | 358721 | 379965 | Validamycin_biosynthetic_gene_cluster (59% of genes show similarity) | BGC0000722_c1 |  |  |
| Cluster 2 | T1pks | 392106 | 450957 | Validamycin_biosynthetic_gene_cluster (18% of genes show similarity) | BGC0000722_c1 | 1 | CYP105B22 |
| Cluster 3 | Bacteriocin | 702612 | 713424 | - | - |  |  |
| Cluster 4 | Nrps | 725003 | 785737 | Laspartomycin_biosynthetic_gene_cluster (6% of genes show similarity) | BGC0000379_c1 |  |  |
| Cluster 5 | T3pks | 869601 | 910647 | Calcium-dependent_antibiotic_biosynthetic_gene_cluster (17% of genes show similarity) | BGC0000315_c1 |  |  |
| Cluster 6 | Terpene | 996365 | 1017102 | Kedarcidin_biosynthetic_gene_cluster (1% of genes show similarity) | BGC0000081_c1 | 1 | CYP107X1 |
| Cluster 7 | Terpene | 1059813 | 1080706 | - | - |  |  |
| Cluster 8 | Siderophore | 1105331 | 1118637 | - | - |  |  |
| Cluster 9 | Phenazine-Nrps | 1334791 | 1396137 | Azinomycin_B_biosynthetic_gene_cluster (6% of genes show similarity) | BGC0000960_c1 |  |  |
| Cluster 10 | T2pks | 1404229 | 1446738 | Dactylocycline_biosynthetic_gene_cluster (28% of genes show similarity) | BGC0000216_c1 |  |  |
| Cluster 11 | T1pks-Nrps | 1489978 | 1547401 | Antimycin_biosynthetic_gene_cluster (93% of genes show similarity) | BGC0000958_c1 |  |  |
| Cluster 12 | Nrps | 1622283 | 1695049 | Laspartomycin_biosynthetic_gene_cluster (18% of genes show similarity) | BGC0000379_c1 | 1 | CYP163B6 |
| Cluster 13 | T1pks | 1811332 | 1857499 | Ansatrienin_(mycotrienin)_biosynthetic_gene_cluster (7% of genes show similarity) | BGC0000957_c1 |  |  |
| Cluster 14 | Lantipeptide-Terpene-Nrps | 1971071 | 2064317 | Carotenoid_biosynthetic_gene_cluster (54% of genes show similarity) | BGC0000633_c1 |  |  |
| Cluster 15 | Nrps | 2205659 | 2262627 | Zorbamycin_biosynthetic_gene_cluster (8% of genes show similarity) | BGC0001058_c1 | 1 | CYP285B1 |
| Cluster 16 | T1pks | 2293648 | 2404726 | Vicenistatin_biosynthetic_gene_cluster (60% of genes show similarity) | BGC0000167_c1 | 2 | CYP105AZ2; CYP105AZ1 |
| Cluster 17 | T3pks | 3138718 | 3179740 | Herboxidiene_biosynthetic_gene_cluster (7% of genes show similarity) | BGC0001065_c1 | 1 | CYP158A14 |
| Cluster 18 | Ectoine | 3881676 | 3892080 | Ectoine_biosynthetic_gene_cluster (100% of genes show similarity) | BGC0000853_c1 |  |  |
| Cluster 19 | T2pks | 4251729 | 4294228 | Spore_pigment_biosynthetic_gene_cluster (83% of genes show similarity) | BGC0000271_c1 |  |  |
| Cluster 20 | Butyrolactone | 4609696 | 4620709 | Methylenomycin_biosynthetic_gene_cluster (14% of genes show similarity) | BGC0000914_c1 |  |  |
| Cluster 21 | Melanin | 4830523 | 4841017 | Istamycin_biosynthetic_gene_cluster (4% of genes show similarity) | BGC0000700_c1 |  |  |
| Cluster 22 | Siderophore | 4931971 | 4943740 | Desferrioxamine_B_biosynthetic_gene_cluster (100% of genes show similarity) | BGC0000940_c1 |  |  |
| Cluster 23 | Nrps | 6457264 | 6510229 | Arginomycin_biosynthetic_gene_cluster (10% of genes show similarity) | BGC0000883_c1 |  |  |
| Cluster 24 | T2pks-Ladderane-Nrps | 6727130 | 6796406 | Skyllamycin_biosynthetic_gene_cluster (20% of genes show similarity) | BGC0000429_c1 |  |  |
| Cluster 25 | Terpene | 7197859 | 7218872 | Albaflavenone_biosynthetic_gene_cluster (100% of genes show similarity) | BGC0000660_c1 | 1 | CYP170A10 |
| Cluster 26 | Siderophore | 7848002 | 7860050 | - | - |  |  |
| Cluster 27 | Bacteriocin-Nrps | 7996885 | 8075186 | Meilingmycin_biosynthetic_gene_cluster (6% of genes show similarity) | BGC0000093_c1 |  |  |
| Cluster 28 | Bacteriocin | 8112492 | 8123820 | - | - |  |  |
| Cluster 29 | Terpene | 8147354 | 8169507 | - | - | 1 | CYP180A6 |
| Cluster 30 | Siderophore | 8310258 | 8323412 | Grincamycin_biosynthetic_gene_cluster (8% of genes show similarity) | BGC0000229_c1 |  |  |
| Cluster 31 | Butyrolactone | 8344332 | 8355243 | Methylenomycin_biosynthetic_gene_cluster (14% of genes show similarity) | BGC0000914_c1 |  |  |
| Cluster 32 | Terpene | 8696575 | 8723398 | Hopene_biosynthetic_gene_cluster (92% of genes show similarity) | BGC0000663_c1 |  |  |
| Cluster 33 | Lantipeptide | 9016982 | 9041437 | - | - |  |  |
| Cluster 34 | Bacteriocin-Nrps | 9252598 | 9340733 | Informatipeptin_biosynthetic_gene_cluster (57% of genes show similarity) | BGC0000518_c1 | 1 | CYP113K3 |
| Cluster 35 | T3pks | 9689376 | 9730512 | Yatakemycin_biosynthetic_gene_cluster (48% of genes show similarity) | BGC0000466_c1 |  |  |
| Cluster 36 | Lassopeptide | 9750195 | 9772223 | - | - |  |  |
| Cluster 37 | Butyrolactone | 9868256 | 9936950 | A-500359s_biosynthetic_gene_cluster (5% of genes show similarity) | BGC0000949_c1 |  |  |
| Cluster 38 | Thiopeptide | 10075365 | 10100043 | Cyclothiazomycin_biosynthetic_gene_cluster (57% of genes show similarity) | BGC0000603_c1 |  |  |
| ***Streptomyces hygroscopicus* subsp. *jinggangensis* TL01** | | | | | | | |
| Cluster 1 | Amglyccycl | 132410 | 153654 | Validamycin_biosynthetic_gene_cluster (55% of genes show similarity) | BGC0000722_c1 |  |  |
| Cluster 2 | T1pks | 165795 | 224646 | Validamycin_biosynthetic_gene_cluster (18% of genes show similarity) | BGC0000722_c1 | 1 | CYP105B22 |
| Cluster 3 | Bacteriocin | 476341 | 487153 | - | - |  |  |
| Cluster 4 | Nrps | 498732 | 559466 | Laspartomycin_biosynthetic_gene_cluster (6% of genes show similarity) | BGC0000379_c1 |  |  |
| Cluster 5 | T3pks | 643330 | 684376 | Calcium-dependent_antibiotic_biosynthetic_gene_cluster (17% of genes show similarity) | BGC0000315_c1 |  |  |
| Cluster 6 | Terpene | 770094 | 790831 | Kedarcidin_biosynthetic_gene_cluster (1% of genes show similarity) | BGC0000081_c1 | 1 | CYP107X1 |
| Cluster 7 | Terpene | 833542 | 854435 | - | - |  |  |
| Cluster 8 | Siderophore | 879060 | 892366 | - | - |  |  |
| Cluster 9 | Phenazine-Nrps | 1108524 | 1169870 | Azinomycin_B_biosynthetic_gene_cluster (6% of genes show similarity) | BGC0000960_c1 |  |  |
| Cluster 10 | T2pks | 1177962 | 1220471 | Dactylocycline_biosynthetic_gene_cluster (28% of genes show similarity) | BGC0000216_c1 |  |  |
| Cluster 11 | T1pks-Nrps | 1263711 | 1321134 | Antimycin_biosynthetic_gene_cluster (93% of genes show similarity) | BGC0000958_c1 |  |  |
| Cluster 12 | Nrps | 1396016 | 1468782 | Laspartomycin_biosynthetic_gene_cluster (18% of genes show similarity) | BGC0000379_c1 | 1 | CYP163B6 |
| Cluster 13 | T1pks | 1585065 | 1631232 | Ansatrienin_(mycotrienin)_biosynthetic_gene_cluster (7% of genes show similarity) | BGC0000957_c1 |  |  |
| Cluster 14 | Lantipeptide-Terpene-Nrps | 1664585 | 1757831 | Carotenoid_biosynthetic_gene_cluster (54% of genes show similarity) | BGC0000633_c1 |  |  |
| Cluster 15 | Nrps | 1899173 | 1956141 | Zorbamycin_biosynthetic_gene_cluster (8% of genes show similarity) | BGC0001058_c1 | 1 | CYP285B1 |
| Cluster 16 | T1pks | 1987162 | 2098240 | Vicenistatin_biosynthetic_gene_cluster (60% of genes show similarity) | BGC0000167_c1 | 2 | CYP105AZ2; CYP105AZ1 |
| Cluster 17 | T3pks | 2832233 | 2873255 | Herboxidiene_biosynthetic_gene_cluster (7% of genes show similarity) | BGC0001065_c1 | 1 | CYP158A14 |
| Cluster 18 | Ectoine | 3575399 | 3585803 | Ectoine_biosynthetic_gene_cluster (100% of genes show similarity) | BGC0000853_c1 |  |  |
| Cluster 19 | T2pks | 3945338 | 3987837 | Spore_pigment_biosynthetic_gene_cluster (83% of genes show similarity) | BGC0000271_c1 |  |  |
| Cluster 20 | Butyrolactone | 4303305 | 4314318 | Methylenomycin_biosynthetic_gene_cluster (14% of genes show similarity) | BGC0000914_c1 |  |  |
| Cluster 21 | Melanin | 4524132 | 4534626 | Istamycin_biosynthetic_gene_cluster (4% of genes show similarity) | BGC0000700_c1 |  |  |
| Cluster 22 | Siderophore | 4625580 | 4637349 | Desferrioxamine_B_biosynthetic_gene_cluster (100% of genes show similarity) | BGC0000940_c1 |  |  |
| Cluster 23 | Nrps | 6150956 | 6203921 | Arginomycin_biosynthetic_gene_cluster (10% of genes show similarity) | BGC0000883_c1 |  |  |
| Cluster 24 | T2pks-Ladderane-Nrps | 6420822 | 6490098 | Skyllamycin_biosynthetic_gene_cluster (20% of genes show similarity) | BGC0000429_c1 |  |  |
| Cluster 25 | Terpene | 6891676 | 6912689 | Albaflavenone_biosynthetic_gene_cluster (100% of genes show similarity) | BGC0000660_c1 | 1 | CYP170A10 |
| Cluster 26 | Siderophore | 7541947 | 7553995 | - | - |  |  |
| Cluster 27 | Bacteriocin-Nrps | 7690830 | 7769131 | Meilingmycin_biosynthetic_gene_cluster (6% of genes show similarity) | BGC0000093_c1 |  |  |
| Cluster 28 | Bacteriocin | 7806437 | 7817765 | - | - |  |  |
| Cluster 29 | Terpene | 7841299 | 7863452 | - | - | 1 | CYP180A6 |
| Cluster 30 | Siderophore | 8004203 | 8017357 | Grincamycin_biosynthetic_gene_cluster (8% of genes show similarity) | BGC0000229_c1 |  |  |
| Cluster 31 | Butyrolactone | 8038418 | 8049329 | Methylenomycin_biosynthetic_gene_cluster (14% of genes show similarity) | BGC0000914_c1 |  |  |
| Cluster 32 | Terpene | 8390775 | 8417598 | Hopene_biosynthetic_gene_cluster (92% of genes show similarity) | BGC0000663_c1 |  |  |
| Cluster 33 | Lantipeptide | 8711185 | 8735640 | - | - |  |  |
| Cluster 34 | Bacteriocin-Nrps | 8946801 | 9034936 | Informatipeptin_biosynthetic_gene_cluster (57% of genes show similarity) | BGC0000518_c1 | 1 | CYP113K3 |
| Cluster 35 | T3pks | 9383644 | 9424780 | Yatakemycin_biosynthetic_gene_cluster (48% of genes show similarity) | BGC0000466_c1 |  |  |
| Cluster 36 | Lassopeptide | 9444463 | 9466491 | - | - |  |  |
| Cluster 37 | Butyrolactone | 9562524 | 9631218 | A-500359s_biosynthetic_gene_cluster (5% of genes show similarity) | BGC0000949_c1 |  |  |
| Cluster 38 | Thiopeptide | 9769634 | 9794312 | Cyclothiazomycin_biosynthetic_gene_cluster (57% of genes show similarity) | BGC0000603_c1 |  |  |
| ***Streptomyces venezuelae*** | | | | | | | |
| Cluster 1 | Ectoine | 237842 | 248258 | Ectoine_biosynthetic_gene_cluster (100% of genes show similarity) | BGC0000853_c1 |  |  |
| Cluster 2 | Terpene | 274553 | 296739 | - | - |  |  |
| Cluster 3 | T3pks-T1pks-Nrps | 504136 | 604067 | Thiotetronate_Tu_3010_biosynthetic_gene_cluster (20% of genes show similarity) | BGC0001352_c1 |  |  |
| Cluster 4 | Lantipeptide-Terpene | 614220 | 645285 | Chrysomycin_biosynthetic_gene_cluster (5% of genes show similarity) | BGC0000211_c1 | 1 | CYP157C14 |
| Cluster 5 | Lantipeptide | 707463 | 730315 | Venezuelin_biosynthetic_gene_cluster (100% of genes show similarity) | BGC0000563_c1 |  |  |
| Cluster 6 | Indole | 867489 | 890695 | Rebeccamycin_biosynthetic_gene_cluster (36% of genes show similarity) | BGC0000823_c1 | 1 | CYP245A3 |
| Cluster 7 | Other | 1031023 | 1073914 | Chloramphenicol_biosynthetic_gene_cluster (100% of genes show similarity) | BGC0000893_c1 |  |  |
| Cluster 8 | Other | 2055965 | 2096690 | - | - | 1 | CYP121A2 |
| Cluster 9 | Siderophore | 2794973 | 2806751 | Desferrioxamine_B_biosynthetic_gene_cluster (100% of genes show similarity) | BGC0000941_c1 |  |  |
| Cluster 10 | Lassopeptide | 3408328 | 3430687 | Phosphonoglycans_biosynthetic_gene_cluster (3% of genes show similarity) | BGC0000806_c1 |  |  |
| Cluster 11 | Other | 4408196 | 4451900 | Lactonamycin_biosynthetic_gene_cluster (10% of genes show similarity) | BGC0000238_c1 |  |  |
| Cluster 12 | Butyrolactone | 4522134 | 4533171 | - | - |  |  |
| Cluster 13 | Melanin | 5003818 | 5014228 | Istamycin_biosynthetic_gene_cluster (8% of genes show similarity) | BGC0000700_c1 |  |  |
| Cluster 14 | Butyrolactone | 5462370 | 5517716 | A-503083_biosynthetic_gene_cluster (3% of genes show similarity) | BGC0000288_c1 |  |  |
| Cluster 15 | Thiopeptide | 5531076 | 5557501 | BD-12_biosynthetic_gene_cluster (14% of genes show similarity) | BGC0001379_c1 |  |  |
| Cluster 16 | T3pks | 5785193 | 5826323 | K-252a_biosynthetic_gene_cluster (11% of genes show similarity) | BGC0000814_c1 | 1 | CYP158A5 |
| Cluster 17 | Siderophore | 5869901 | 5883169 | - | - |  |  |
| Cluster 18 | Siderophore | 5935668 | 5950407 | - | - |  |  |
| Cluster 19 | Bacteriocin | 6350466 | 6361866 | - | - |  |  |
| Cluster 20 | T2pks-Butyrolactone | 6479470 | 6531416 | Rabelomycin_biosynthetic_gene_cluster (33% of genes show similarity) | BGC0000262_c1 |  |  |
| Cluster 21 | Other | 6672467 | 6716369 | Herbimycin_biosynthetic_gene_cluster (6% of genes show similarity) | BGC0000074_c1 | 2 | CYP105AC2; CYP180A5 |
| Cluster 22 | Ladderane-Nrps | 6720590 | 6855167 | Skyllamycin_biosynthetic_gene_cluster (24% of genes show similarity) | BGC0000429_c1 | 4 | CYP1056A1; CYP107CL1; CYP162A4; CYP163B5 |
| Cluster 23 | Terpene | 7021575 | 7048100 | Hopene_biosynthetic_gene_cluster (69% of genes show similarity) | BGC0000663_c1 |  |  |
| Cluster 24 | Bacteriocin | 7128838 | 7139692 | - | - |  |  |
| Cluster 25 | T2pks | 7421589 | 7464101 | Spore_pigment_biosynthetic_gene_cluster (83% of genes show similarity) | BGC0000271_c1 |  |  |
| Cluster 26 | Melanin | 7484949 | 7495338 | Melanin_biosynthetic_gene_cluster (28% of genes show similarity) | BGC0000908_c1 |  |  |
| Cluster 27 | Nrps | 7706602 | 7760938 | Albachelin_biosynthetic_gene_cluster (80% of genes show similarity) | BGC0001211_c1 |  |  |
| Cluster 28 | Terpene | 7788497 | 7809951 | Kanamycin_biosynthetic_gene_cluster (1% of genes show similarity) | BGC0000703_c1 |  |  |
| Cluster 29 | T3pks | 7946080 | 7987237 | Alkylresorcinol_biosynthetic_gene_cluster (100% of genes show similarity) | BGC0000282_c1 |  |  |
| Cluster 30 | Terpene-Nrps | 8179935 | 8226158 | A54145_biosynthetic_gene_cluster (3% of genes show similarity) | BGC0000291_c1 |  |  |
| ***Streptomyces davawensis*** | | | | | | | |
| Cluster 1 | Lassopeptide | 298906 | 321414 | - | - |  |  |
| Cluster 2 | T1pks-Nrps | 699548 | 770346 | Lasalocid_biosynthetic_gene_cluster (9% of genes show similarity) | BGC0000087_c1 | 1 | CYP105BA1 |
| Cluster 3 | Other | 886131 | 928683 | Meridamycin_biosynthetic_gene_cluster (5% of genes show similarity) | BGC0001011_c1 | 1 | CYP1005B2 |
| Cluster 4 | Bacteriocin-Lantipeptide | 1091732 | 1132429 | Informatipeptin_biosynthetic_gene_cluster (100% of genes show similarity) | BGC0000518_c1 | 1 | CYP179B1 |
| Cluster 5 | Lassopeptide | 1361367 | 1383943 | - | - |  |  |
| Cluster 6 | T1pks | 1456468 | 1499518 | RK-682_biosynthetic_gene_cluster (100% of genes show similarity) | BGC0000140_c1 |  |  |
| Cluster 7 | Terpene | 1551356 | 1572273 | - | - | 1 | CYP183K1 |
| Cluster 8 | Terpene | 1719195 | 1745819 | Hopene_biosynthetic_gene_cluster (100% of genes show similarity) | BGC0000663_c1 |  |  |
| Cluster 9 | T3pks | 2207909 | 2248970 | - | - |  |  |
| Cluster 10 | Siderophore | 2297883 | 2311023 | Grincamycin_biosynthetic_gene_cluster (8% of genes show similarity) | BGC0000229_c1 |  |  |
| Cluster 11 | Lantipeptide-T1pks-Nrps | 2350589 | 2428901 | Jerangolid_biosynthetic_gene_cluster (9% of genes show similarity) | BGC0000080_c1 | 1 | CYP179A3 |
| Cluster 12 | Butyrolactone-Terpene | 2547059 | 2574343 | Gamma-butyrolactone_biosynthetic_gene_cluster (100% of genes show similarity) | BGC0000850_c1 | 1 | CYP180A6 |
| Cluster 13 | Bacteriocin-Oligosaccharide | 2613657 | 2675880 | Meilingmycin_biosynthetic_gene_cluster (5% of genes show similarity) | BGC0000093_c1 | 2 | CYP107CN1; CYP107CP1 |
| Cluster 14 | T2pks-T1pks | 2749113 | 2852588 | Pyrrolomycin_biosynthetic_gene_cluster (45% of genes show similarity) | BGC0000130_c1 |  |  |
| Cluster 15 | Siderophore | 2995354 | 3007165 | - | - |  |  |
| Cluster 16 | Terpene-Nrps | 3556587 | 3614866 | Albaflavenone_biosynthetic_gene_cluster (100% of genes show similarity) | BGC0000660_c1 | 1 | CYP170A9 |
| Cluster 17 | Phosphonate | 4565919 | 4606854 | Rhizocticin_biosynthetic_gene_cluster (19% of genes show similarity) | BGC0000926_c1 |  |  |
| Cluster 18 | Butyrolactone | 4950501 | 4961502 | - | - |  |  |
| Cluster 19 | Siderophore | 6101969 | 6113741 | Desferrioxamine_B_biosynthetic_gene_cluster (100% of genes show similarity) | BGC0000940_c1 |  |  |
| Cluster 20 | Melanin-Nrps | 6185869 | 6254487 | Melanin_biosynthetic_gene_cluster (100% of genes show similarity) | BGC0000909_c1 |  |  |
| Cluster 21 | T1pks-Nrps | 6539485 | 6608235 | SCO-2138_biosynthetic_gene_cluster (50% of genes show similarity) | BGC0000595_c1 | 3 | CYP113J1; CYP113J2; CYP162A5 |
| Cluster 22 | T3pks | 6619324 | 6660409 | SWA-2138_biosynthetic_gene_cluster (42% of genes show similarity) | BGC0000597_c1 |  |  |
| Cluster 23 | Otherks-Nrps | 6741355 | 6816556 | Taromycin_biosynthetic_gene_cluster (10% of genes show similarity) | BGC0000439_c1 | 2 | CYP125A22; CYP163C2 |
| Cluster 24 | Ectoine | 7452784 | 7463182 | Ectoine_biosynthetic_gene_cluster (100% of genes show similarity) | BGC0000853_c1 |  |  |
| Cluster 25 | Other | 7566704 | 7610435 | Gobichelin_biosynthetic_gene_cluster (11% of genes show similarity) | BGC0000366_c1 |  |  |
| Cluster 26 | Other | 7715214 | 7759068 | - | - |  |  |
| Cluster 27 | T3pks | 8237578 | 8278642 | Herboxidiene_biosynthetic_gene_cluster (8% of genes show similarity) | BGC0001065_c1 | 1 | CYP158A7 |
| Cluster 28 | Nrps | 8403464 | 8462129 | Scabichelin_biosynthetic_gene_cluster (100% of genes show similarity) | BGC0000423_c1 |  |  |
| Cluster 29 | Lantipeptide | 8666789 | 8691350 | - | - |  |  |
| Cluster 30 | Terpene-T3pks-Cyanobactin-Nrps | 8866036 | 8983549 | Furaquinocin_A_biosynthetic_gene_cluster (100% of genes show similarity) | BGC0001078_c1 | 2 | CYP1041A2; CYP1058A1 |
| Cluster 31 | Melanin-Terpene | 9217881 | 9244553 | Melanin_biosynthetic_gene_cluster (57% of genes show similarity) | BGC0000908_c1 |  |  |
| ***Streptomyces albus* J1074** | | | | | | | |
| Cluster 1 | T1pks-Nrps | 3011 | 61711 | - | - |  |  |
| Cluster 2 | T1pks-Nrps | 224752 | 274162 | SGR_PTMs_biosynthetic_gene_cluster (100% of genes show similarity) | BGC0001043_c1 | 1 | CYP107BX2 |
| Cluster 3 | Terpene | 308626 | 335190 | Hopene_biosynthetic_gene_cluster (76% of genes show similarity) | BGC0000663_c1 |  |  |
| Cluster 4 | Bacteriocin | 415688 | 425903 | - | - |  |  |
| Cluster 5 | Otherks | 669953 | 711002 | Avermectin_biosynthetic_gene_cluster (66% of genes show similarity) | BGC0000025_c1 | 1 | CYP1420A1 |
| Cluster 6 | Bacteriocin | 879961 | 891289 | - | - |  |  |
| Cluster 7 | Nrps | 1136316 | 1199422 | Tetronasin_biosynthetic_gene_cluster (9% of genes show similarity) | BGC0000163_c1 | 1 | CYP146A3 |
| Cluster 8 | Siderophore | 1268164 | 1283196 | - | - |  |  |
| Cluster 9 | Terpene | 1531774 | 1554059 | Kanamycin_biosynthetic_gene_cluster (1% of genes show similarity) | BGC0000703_c1 |  |  |
| Cluster 10 | Terpene | 1865241 | 1886293 | Albaflavenone_biosynthetic_gene_cluster (100% of genes show similarity) | BGC0000660_c1 | 1 | CYP170B5 |
| Cluster 11 | Thiopeptide | 2376688 | 2409159 | - | - |  |  |
| Cluster 12 | Bacteriocin | 2560714 | 2571226 | Goadsporin_biosynthetic_gene_cluster (12% of genes show similarity) | BGC0000565_c1 |  |  |
| Cluster 13 | Lantipeptide | 2694944 | 2735867 | SAL-2242_biosynthetic_gene_cluster (100% of genes show similarity) | BGC0000546_c1 | 1 | CYP154A1 |
| Cluster 14 | Nrps | 3553726 | 3604015 | Scabichelin_biosynthetic_gene_cluster (40% of genes show similarity) | BGC0000423_c1 |  |  |
| Cluster 15 | Nrps | 3877105 | 3982798 | Herboxidiene_biosynthetic_gene_cluster (4% of genes show similarity) | BGC0001065_c1 |  |  |
| Cluster 16 | Nrps | 4469477 | 4513826 | - | - |  |  |
| Cluster 17 | Siderophore | 4740450 | 4752270 | Desferrioxamine_B_biosynthetic_gene_cluster (100% of genes show similarity) | BGC0000941_c1 |  |  |
| Cluster 18 | Ectoine | 5635346 | 5645744 | Ectoine_biosynthetic_gene_cluster (100% of genes show similarity) | BGC0000853_c1 |  |  |
| Cluster 19 | Other | 6337383 | 6381213 | Auricin_deoxysugar_moieties_biosynthetic_gene_cluster (13% of genes show similarity) | BGC0000727_c1 |  |  |
| Cluster 20 | Bacteriocin-Terpene | 6396161 | 6430092 | Carotenoid_biosynthetic_gene_cluster (54% of genes show similarity) | BGC0000633_c1 |  |  |
| Cluster 21 | T3pks | 6520374 | 6561471 | Herboxidiene_biosynthetic_gene_cluster (12% of genes show similarity) | BGC0001065_c1 | 1 | CYP107F4 |
| Cluster 22 | Lantipeptide-T1pks-Nrps | 6566423 | 6838639 | Candicidin_biosynthetic_gene_cluster (100% of genes show similarity) | BGC0000034_c1 | 1 | CYP105H3 |
| ***Streptomyces albus* DSM 41398** | | | | | | | |
| Cluster 1 | Nrps | 117650 | 166226 | Incednine_biosynthetic_gene_cluster (9% of genes show similarity) | BGC0000078_c1 | 1 | CYP105BK3 |
| Cluster 2 | Nrps | 183666 | 273577 | Cahuitamycins_biosynthetic_gene_cluster (25% of genes show similarity) | BGC0001351_c1 |  |  |
| Cluster 3 | T1pks-Butyrolactone-Nrps | 284628 | 428617 | Salinomycin_biosynthetic_gene_cluster (100% of genes show similarity) | BGC0000144_c1 | 1 | CYP107EJ1 |
| Cluster 4 | Amglyccycl | 746865 | 768097 | Pyralomicin_biosynthetic_gene_cluster (22% of genes show similarity) | BGC0001038_c1 |  |  |
| Cluster 5 | T1pks-Otherks | 843887 | 1009285 | PM100117_/_PM100118_biosynthetic_gene_cluster (60% of genes show similarity) | BGC0001359_c2 | 2 | CYP113Y1; CYP105DB1 |
| Cluster 6 | Bacteriocin-Terpene | 1051608 | 1089082 | - | - |  |  |
| Cluster 7 | T1pks | 1174261 | 1276844 | Oligomycin_biosynthetic_gene_cluster (55% of genes show similarity) | BGC0000117_c1 | 1 | CYP107DU1 |
| Cluster 8 | Thiopeptide | 1281647 | 1316387 | - | - |  |  |
| Cluster 9 | Terpene-T1pks | 1416484 | 1519438 | Lasalocid_biosynthetic_gene_cluster (20% of genes show similarity) | BGC0000087_c1 |  |  |
| Cluster 10 | Lantipeptide-Terpene-Nrps | 1512526 | 1617897 | Isorenieratene_biosynthetic_gene_cluster (85% of genes show similarity) | BGC0000664_c1 |  |  |
| Cluster 11 | Lantipeptide | 1622376 | 1645752 | - | - |  |  |
| Cluster 12 | Siderophore | 1912797 | 1924731 | - | - |  |  |
| Cluster 13 | Other | 2456612 | 2498069 | Arginomycin_biosynthetic_gene_cluster (20% of genes show similarity) | BGC0000883_c1 |  |  |
| Cluster 14 | Terpene | 2586173 | 2607264 | Albaflavenone_biosynthetic_gene_cluster (100% of genes show similarity) | BGC0000660_c1 | 1 | CYP170B5 |
| Cluster 15 | T1pks | 2726842 | 2791263 | Streptazone_E_biosynthetic_gene_cluster (58% of genes show similarity) | BGC0001296_c1 |  |  |
| Cluster 16 | Lassopeptide | 2823976 | 2846376 | - | - |  |  |
| Cluster 17 | Butyrolactone | 3380027 | 3391028 | - | - |  |  |
| Cluster 18 | Melanin | 3764392 | 3775000 | Istamycin_biosynthetic_gene_cluster (4% of genes show similarity) | BGC0000700_c1 |  |  |
| Cluster 19 | T1pks-Otherks | 3889534 | 3937675 | Arsenopolyketides_biosynthetic_gene_cluster (83% of genes show similarity) | BGC0001283_c1 |  |  |
| Cluster 20 | Arylpolyene-Nrps | 4012866 | 4110187 | Albachelin_biosynthetic_gene_cluster (70% of genes show similarity) | BGC0001211_c1 | 1 | CYP107T3 |
| Cluster 21 | Other | 4614979 | 4658800 | Herboxidiene_biosynthetic_gene_cluster (2% of genes show similarity) | BGC0001065_c1 |  |  |
| Cluster 22 | Other | 5088621 | 5131383 | Stenothricin_biosynthetic_gene_cluster (13% of genes show similarity) | BGC0000431_c1 |  |  |
| Cluster 23 | Butyrolactone-Otherks | 5451890 | 5493951 | Macrotetrolide_biosynthetic_gene_cluster (25% of genes show similarity) | BGC0000243_c1 |  |  |
| Cluster 24 | Lantipeptide | 5665895 | 5688501 | SAL-2242_biosynthetic_gene_cluster (100% of genes show similarity) | BGC0000546_c1 |  |  |
| Cluster 25 | Arylpolyene | 5725897 | 5794495 | Svaricin_biosynthetic_gene_cluster (9% of genes show similarity) | BGC0001382_c1 | 1 | CYP107KW1 |
| Cluster 26 | T2pks-Otherks | 5913570 | 6003941 | Kinamycin_biosynthetic_gene_cluster (48% of genes show similarity) | BGC0000236_c1 | 1 | CYP1193A1 |
| Cluster 27 | T1pks | 6434045 | 6478607 | - | - |  |  |
| Cluster 28 | Bacteriocin | 6561595 | 6572500 | - | - |  |  |
| Cluster 29 | Melanin-Arylpolyene-Ladderane | 6572744 | 6632562 | Salinilactam_biosynthetic_gene_cluster (8% of genes show similarity) | BGC0000142_c1 |  |  |
| Cluster 30 | Ectoine | 6947249 | 6957647 | Ectoine_biosynthetic_gene_cluster (100% of genes show similarity) | BGC0000853_c1 |  |  |
| Cluster 31 | Terpene | 7787708 | 7814387 | Hopene_biosynthetic_gene_cluster (61% of genes show similarity) | BGC0000663_c1 |  |  |
| Cluster 32 | Nucleoside | 7841287 | 7861661 | - | - |  |  |
| Cluster 33 | T1pks-Nrps | 7925460 | 8051064 | Undecylprodigiosin_biosynthetic_gene_cluster (13% of genes show similarity) | BGC0001063_c1 | 1 | CYP1194A1 |
| Cluster 34 | Siderophore | 8262822 | 8276665 | - | - |  |  |
| Cluster 35 | T1pks-Phenazine | 8302435 | 8384669 | Elaiophylin_biosynthetic_gene_cluster (79% of genes show similarity) | BGC0000053_c1 |  |  |
| ***Streptomyces* sp. PAMC 26508** | | | | | | | |
| Cluster 1 | Butyrolactone | 207520 | 218455 | - | - |  |  |
| Cluster 2 | Nrps | 289719 | 343175 | Zorbamycin_biosynthetic_gene_cluster (6% of genes show similarity) | BGC0001058_c1 | 2 | CYP156B9; CYP1035A4 |
| Cluster 3 | T2pks-Terpene | 491367 | 540556 | Spore_pigment_biosynthetic_gene_cluster (83% of genes show similarity) | BGC0000271_c1 |  |  |
| Cluster 4 | Melanin | 581362 | 591826 | Melanin_biosynthetic_gene_cluster (100% of genes show similarity) | BGC0000911_c1 | 1 | CYP124G4 |
| Cluster 5 | T3pks | 688185 | 729243 | Tetronasin_biosynthetic_gene_cluster (11% of genes show similarity) | BGC0000163_c1 |  |  |
| Cluster 6 | Bacteriocin | 882331 | 892558 | - | - |  |  |
| Cluster 7 | Terpene-Ectoine | 915537 | 941976 | Ectoine_biosynthetic_gene_cluster (100% of genes show similarity) | BGC0000853_c1 |  |  |
| Cluster 8 | Terpene | 1222262 | 1243308 | Steffimycin_biosynthetic_gene_cluster (19% of genes show similarity) | BGC0000273_c1 |  |  |
| Cluster 9 | T2pks-Otherks | 1288058 | 1345321 | Cinerubin_B_biosynthetic_gene_cluster (28% of genes show similarity) | BGC0000212_c1 |  |  |
| Cluster 10 | Ectoine | 1758238 | 1768636 | Ectoine_biosynthetic_gene_cluster (100% of genes show similarity) | BGC0000853_c1 |  |  |
| Cluster 11 | Terpene | 2220628 | 2241659 | - | - | 1 | CYP157K1 |
| Cluster 12 | Lantipeptide | 2650699 | 2673779 | - | - |  |  |
| Cluster 13 | Siderophore | 2726161 | 2737942 | Desferrioxamine_B_biosynthetic_gene_cluster (83% of genes show similarity) | BGC0000940_c1 |  |  |
| Cluster 14 | T1pks-Nrps | 3360869 | 3417772 | Istamycin_biosynthetic_gene_cluster (11% of genes show similarity) | BGC0000700_c1 | 3 | CYP285A2, CYP1423A2, CYP1029A2 |
| Cluster 15 | Butyrolactone | 3618213 | 3629124 | Lactonamycin_biosynthetic_gene_cluster (3% of genes show similarity) | BGC0000238_c1 |  |  |
| Cluster 16 | Bacteriocin | 5270198 | 5280419 | - | - |  |  |
| Cluster 17 | Terpene | 5440267 | 5461337 | - | - |  |  |
| Cluster 18 | Siderophore | 5525938 | 5540522 | - | - |  |  |
| Cluster 19 | Nrps | 5813270 | 5875303 | Arylomycin_biosynthetic_gene_cluster (22% of genes show similarity) | BGC0000306_c1 |  |  |
| Cluster 20 | Bacteriocin | 5954424 | 5965716 | - | - |  |  |
| Cluster 21 | T1pks | 6177678 | 6272426 | Vicenistatin_biosynthetic_gene_cluster (60% of genes show similarity) | BGC0000167_c1 | 2 | CYP105AZ1; CYP105AZ2 |
| Cluster 22 | Terpene | 6622210 | 6648795 | Hopene_biosynthetic_gene_cluster (69% of genes show similarity) | BGC0000663_c1 |  |  |
| Cluster 23 | Blactam | 6729751 | 6753170 | Clavulanic_acid_biosynthetic_gene_cluster (20% of genes show similarity) | BGC0000845_c1 |  |  |
| Cluster 24 | Nrps | 6809052 | 6859494 | BE-14106_biosynthetic_gene_cluster (10% of genes show similarity) | BGC0000029_c1 |  |  |
| Cluster 25 | Bacteriocin-Otherks | 6902116 | 6963314 | A54145_biosynthetic_gene_cluster (5% of genes show similarity) | BGC0000291_c1 | 1 | CYP1057A1 |
| Cluster 26 | Terpene | 7090034 | 7115526 | Isorenieratene_biosynthetic_gene_cluster (100% of genes show similarity) | BGC0000664_c1 |  |  |
| Cluster 27 | Nrps | 7129534 | 7180438 | Coelichelin_biosynthetic_gene_cluster (90% of genes show similarity) | BGC0000325_c1 |  |  |
| Cluster 28 | Blactam-T1pks-Nrps | 7287163 | 7437851 | Carbapenem_MM_4550_biosynthetic_gene_cluster (65% of genes show similarity) | BGC0000842_c1 | 2 | CYP107BX5; CYP247A3 |
| ***Streptomyces fulvissimus*** | | | | | | | |
| Cluster 1 | Terpene | 24365 | 49959 | Isorenieratene_biosynthetic_gene_cluster (100% of genes show similarity) | BGC0000664_c1 |  |  |
| Cluster 2 | Nrps | 117602 | 164831 | A-500359s_biosynthetic_gene_cluster (5% of genes show similarity) | BGC0000949_c1 |  |  |
| Cluster 3 | Ectoine | 192433 | 202834 | - | - |  |  |
| Cluster 4 | Butyrolactone | 290337 | 301287 | Coelimycin_biosynthetic_gene_cluster (16% of genes show similarity) | BGC0000038_c1 |  |  |
| Cluster 5 | Terpene | 330914 | 353127 | - | - |  |  |
| Cluster 6 | Nrps-Transatpks-T1pks-Otherks | 362798 | 478874 | Griseobactin_biosynthetic_gene_cluster (94% of genes show similarity) | BGC0000368_c1 |  |  |
| Cluster 7 | Nrps | 483517 | 534421 | Coelichelin_biosynthetic_gene_cluster (81% of genes show similarity) | BGC0000325_c1 |  |  |
| Cluster 8 | T1pks-Nrps | 540366 | 590843 | Arsenopolyketides_biosynthetic_gene_cluster (45% of genes show similarity) | BGC0001283_c1 |  |  |
| Cluster 9 | T3pks | 601562 | 642680 | Herboxidiene_biosynthetic_gene_cluster (6% of genes show similarity) | BGC0001065_c1 | 1 | CYP107F4 |
| Cluster 10 | Terpene | 1114958 | 1136052 | Steffimycin_biosynthetic_gene_cluster (19% of genes show similarity) | BGC0000273_c1 |  |  |
| Cluster 11 | Ectoine | 1612719 | 1623117 | Ectoine_biosynthetic_gene_cluster (100% of genes show similarity) | BGC0000853_c1 |  |  |
| Cluster 12 | Lantipeptide | 2590811 | 2614077 | - | - |  |  |
| Cluster 13 | Siderophore | 2685518 | 2697296 | Desferrioxamine_B_biosynthetic_gene_cluster (100% of genes show similarity) | BGC0000941_c1 |  |  |
| Cluster 14 | Other | 2783366 | 2827199 | Bottromycin_A2_biosynthetic_gene_cluster (39% of genes show similarity) | BGC0000469_c1 |  |  |
| Cluster 15 | Thiopeptide | 3079000 | 3111360 | - | - |  |  |
| Cluster 16 | Nrps | 3311019 | 3375870 | Phosphonoglycans_biosynthetic_gene_cluster (3% of genes show similarity) | BGC0000806_c1 |  |  |
| Cluster 17 | T2pks | 4454092 | 4496646 | Auricin_biosynthetic_gene_cluster (44% of genes show similarity) | BGC0000201_c1 |  |  |
| Cluster 18 | Lassopeptide | 4558373 | 4580998 | SRO15-2005_biosynthetic_gene_cluster (100% of genes show similarity) | BGC0000578_c1 |  |  |
| Cluster 19 | T1pks | 5106090 | 5171059 | Streptazone_E_biosynthetic_gene_cluster (83% of genes show similarity) | BGC0001296_c1 |  |  |
| Cluster 20 | Lantipeptide | 5444394 | 5467015 | AmfS_biosynthetic_gene_cluster (100% of genes show similarity) | BGC0000496_c1 |  |  |
| Cluster 21 | Terpene | 5812728 | 5842233 | Calcimycin_biosynthetic_gene_cluster (17% of genes show similarity) | BGC0000032_c1 |  |  |
| Cluster 22 | Siderophore | 6318303 | 6333008 | - | - |  |  |
| Cluster 23 | Lantipeptide | 6520512 | 6545133 | Nataxazole_biosynthetic_gene_cluster (7% of genes show similarity) | BGC0001213_c1 |  |  |
| Cluster 24 | Other | 6603133 | 6647134 | Lomofungin_biosynthetic_gene_cluster (13% of genes show similarity) | BGC0001302_c1 |  |  |
| Cluster 25 | Bacteriocin | 6707089 | 6718420 | - | - |  |  |
| Cluster 26 | Nrps | 6852653 | 6913175 | Salinomycin_biosynthetic_gene_cluster (14% of genes show similarity) | BGC0000144_c1 |  |  |
| Cluster 27 | Arylpolyene | 6967295 | 7010476 | Hexaricin_biosynthetic_gene_cluster (6% of genes show similarity) | BGC0001376_c1 |  |  |
| Cluster 28 | T2pks | 7029570 | 7070850 | Nonactin_biosynthetic_gene_cluster (92% of genes show similarity) | BGC0000252_c1 |  |  |
| Cluster 29 | Terpene | 7318585 | 7345177 | Hopene_biosynthetic_gene_cluster (69% of genes show similarity) | BGC0000663_c1 |  |  |
| Cluster 30 | T1pks-Nrps | 7412328 | 7461708 | SGR_PTMs_biosynthetic_gene_cluster (100% of genes show similarity) | BGC0001043_c1 | 1 | CYP107BX3 |
| Cluster 31 | Bacteriocin | 7500970 | 7511770 | Tetronasin_biosynthetic_gene_cluster (3% of genes show similarity) | BGC0000163_c1 |  |  |
| Cluster 32 | Nrps | 7648530 | 7706884 | Valinomycin_biosynthetic_gene_cluster (22% of genes show similarity) | BGC0000453_c1 |  |  |
| Cluster 33 | Melanin | 7712879 | 7723352 | Melanin_biosynthetic_gene_cluster (100% of genes show similarity) | BGC0000911_c1 | 1 | CYP124G2 |
| Cluster 34 | T3pks | 7755934 | 7796986 | Alkylresorcinol_biosynthetic_gene_cluster (100% of genes show similarity) | BGC0000282_c1 |  |  |
| ***Streptomyces collinus*** | | | | | | | |
| Cluster 1 | Lantipeptide | 175267 | 198335 | Streptocollin_biosynthetic_gene_cluster (100% of genes show similarity) | BGC0001226_c1 |  |  |
| Cluster 2 | Lassopeptide-Nrps | 195828 | 265307 | Azicemicin_biosynthetic_gene_cluster (11% of genes show similarity) | BGC0000202_c1 |  |  |
| Cluster 3 | Transatpks-T1pks-Nrps | 341018 | 503094 | Kirromycin_biosynthetic_gene_cluster (81% of genes show similarity) | BGC0001070_c1 | 2 | CYP107CQ1; CYP105AJ2 |
| Cluster 4 | Terpene | 688085 | 713347 | Isorenieratene_biosynthetic_gene_cluster (100% of genes show similarity) | BGC0001227_c1 |  |  |
| Cluster 5 | Other | 749234 | 793100 | Lasalocid_biosynthetic_gene_cluster (11% of genes show similarity) | BGC0000087_c1 |  |  |
| Cluster 6 | Melanin-Terpene | 1068125 | 1093783 | Melanin_biosynthetic_gene_cluster (71% of genes show similarity) | BGC0000908_c1 |  |  |
| Cluster 7 | T1pks-Nrps | 1113779 | 1169492 | Piericidin_A1_biosynthetic_gene_cluster (16% of genes show similarity) | BGC0000124_c1 |  |  |
| Cluster 8 | Bacteriocin | 1176893 | 1188815 | - | - | 2 | CYP1059A1; CYP105B21 |
| Cluster 9 | Other | 1456613 | 1499876 | Stenothricin_biosynthetic_gene_cluster (13% of genes show similarity) | BGC0000431_c1 |  |  |
| Cluster 10 | T3pks | 1500891 | 1541931 | Herboxidiene_biosynthetic_gene_cluster (6% of genes show similarity) | BGC0001065_c1 | 1 | CYP158A2 |
| Cluster 11 | T1pks-Otherks | 1600538 | 1653105 | Cinnamycin_biosynthetic_gene_cluster (14% of genes show similarity) | BGC0000503_c1 |  |  |
| Cluster 12 | Ectoine | 2273646 | 2284050 | Ectoine_biosynthetic_gene_cluster (100% of genes show similarity) | BGC0000853_c1 |  |  |
| Cluster 13 | Thiopeptide-T2pks-Otherks-Nrps | 3030814 | 3123812 | Sch47554_/_Sch47555_biosynthetic_gene_cluster (25% of genes show similarity) | BGC0000268_c1 |  |  |
| Cluster 14 | Melanin | 3299612 | 3310022 | Istamycin_biosynthetic_gene_cluster (5% of genes show similarity) | BGC0000700_c1 |  |  |
| Cluster 15 | Siderophore | 3392488 | 3404257 | Desferrioxamine_B_biosynthetic_gene_cluster (100% of genes show similarity) | BGC0000940_c1 |  |  |
| Cluster 16 | T2pks | 4049256 | 4091753 | Spore_pigment_biosynthetic_gene_cluster (83% of genes show similarity) | BGC0000271_c1 |  |  |
| Cluster 17 | T1pks-Nrps | 4541482 | 4597772 | Chalcomycin_biosynthetic_gene_cluster (7% of genes show similarity) | BGC0000035_c1 |  |  |
| Cluster 18 | Nrps | 4902691 | 4957721 | PM100117_/_PM100118_biosynthetic_gene_cluster (8% of genes show similarity) | BGC0001359_c2 |  |  |
| Cluster 19 | Nrps-Ladderane | 5425623 | 5484284 | Calcium-dependent_antibiotic_biosynthetic_gene_cluster (27% of genes show similarity) | BGC0000315_c1 |  |  |
| Cluster 20 | Terpene | 5642053 | 5663066 | Albaflavenone_biosynthetic_gene_cluster (100% of genes show similarity) | BGC0000660_c1 | 1 | CYP170A10 |
| Cluster 21 | Siderophore | 6264134 | 6276302 | - | - |  |  |
| Cluster 22 | Bacteriocin | 6405006 | 6417150 | Lipopeptide_8D1-1_/_lipopeptide_8D1-2_biosynthetic_gene_clus... (4% of genes show similarity) | BGC0001370_c1 |  |  |
| Cluster 23 | T1pks | 6430389 | 6483375 | Tetronasin_biosynthetic_gene_cluster (3% of genes show similarity) | BGC0000163_c1 | 2 | CYP105BC1; CYP105AH2 |
| Cluster 24 | Bacteriocin | 6548725 | 6560143 | - | - |  |  |
| Cluster 25 | Terpene | 6578041 | 6600203 | - | - | 1 | CYP180A6 |
| Cluster 26 | Siderophore | 6758341 | 6771498 | - | - |  |  |
| Cluster 27 | Terpene | 6862292 | 6883302 | Pentalenolactone_biosynthetic_gene_cluster (58% of genes show similarity) | BGC0000653_c1 | 1 | CYP183A4 |
| Cluster 28 | Terpene | 7118021 | 7144778 | Hopene_biosynthetic_gene_cluster (92% of genes show similarity) | BGC0000663_c1 |  |  |
| Cluster 29 | Other | 7416924 | 7458297 | A-503083_biosynthetic_gene_cluster (7% of genes show similarity) | BGC0000288_c1 |  |  |
| Cluster 30 | Bacteriocin-Nrps | 7527490 | 7616173 | Informatipeptin_biosynthetic_gene_cluster (57% of genes show similarity) | BGC0000518_c1 | 1 | CYP113K3 |
| Cluster 31 | Transatpks-T1pks-Nrps | 7769831 | 7931907 | Kirromycin_biosynthetic_gene_cluster (81% of genes show similarity) | BGC0001070_c1 | 2 | CYP105AJ2; CYP107CQ1 |
| Cluster 32 | Lantipeptide-Lassopeptide-Nrps | 8007618 | 8107658 | Streptocollin_biosynthetic_gene_cluster (100% of genes show similarity) | BGC0001226_c1 |  |  |
| ***Streptomyces rapamycinicus*** | | | | | | | |
| Cluster 1 | T3pks-T1pks-Nrps | 38640 | 150103 | Lobophorin_biosynthetic_gene_cluster (36% of genes show similarity) | BGC0001183_c1 |  |  |
| Cluster 2 | Nrps | 532970 | 583958 | Coelichelin_biosynthetic_gene_cluster (90% of genes show similarity) | BGC0000325_c1 |  |  |
| Cluster 3 | Butyrolactone | 900466 | 911455 | - | - |  |  |
| Cluster 4 | T1pks | 1067818 | 1206134 | ECO-02301_biosynthetic_gene_cluster (39% of genes show similarity) | BGC0000052_c1 | 1 | CYP105AX1 |
| Cluster 5 | T1pks | 1392961 | 1533488 | Nigericin_biosynthetic_gene_cluster (100% of genes show similarity) | BGC0000114_c1 | 1 | CYP124B3 |
| Cluster 6 | T1pks | 1536114 | 1579959 | Salinomycin_biosynthetic_gene_cluster (8% of genes show similarity) | BGC0000144_c1 |  |  |
| Cluster 7 | T1pks | 1680321 | 1757212 | Elaiophylin_biosynthetic_gene_cluster (87% of genes show similarity) | BGC0000053_c1 |  |  |
| Cluster 8 | Hserlactone | 1933809 | 1954564 | Daptomycin_biosynthetic_gene_cluster (4% of genes show similarity) | BGC0000336_c1 |  |  |
| Cluster 9 | Butyrolactone | 1965192 | 1976124 | Meilingmycin_biosynthetic_gene_cluster (2% of genes show similarity) | BGC0000093_c1 |  |  |
| Cluster 10 | T1pks-Nrps | 2182193 | 2235170 | Meilingmycin_biosynthetic_gene_cluster (4% of genes show similarity) | BGC0000093_c1 |  |  |
| Cluster 11 | T3pks-Nrps | 2292584 | 2394338 | Feglymycin_biosynthetic_gene_cluster (78% of genes show similarity) | BGC0001233_c1 |  |  |
| Cluster 12 | T1pks-Nrps | 2500404 | 2707075 | Neocarzilin_biosynthetic_gene_cluster (57% of genes show similarity) | BGC0000111_c1 | 3 | CYP161D1; CYP105AT1; CYP107L12 |
| Cluster 13 | Lantipeptide | 2709760 | 2747820 | BE-7585A_biosynthetic_gene_cluster (23% of genes show similarity) | BGC0000203_c1 |  |  |
| Cluster 14 | Lassopeptide | 2873655 | 2896097 | Streptomycin_biosynthetic_gene_cluster (4% of genes show similarity) | BGC0000717_c1 |  |  |
| Cluster 15 | Terpene | 2934213 | 2960877 | Hopene_biosynthetic_gene_cluster (76% of genes show similarity) | BGC0000663_c1 | 1 | CYP1013A2 |
| Cluster 16 | T2pks | 3350102 | 3392617 | Spore_pigment_biosynthetic_gene_cluster (75% of genes show similarity) | BGC0000271_c1 |  |  |
| Cluster 17 | Bacteriocin | 3679519 | 3690859 | - | - |  |  |
| Cluster 18 | Siderophore | 3859558 | 3871444 | - | - |  |  |
| Cluster 19 | Other | 4448217 | 4490991 | Echosides_biosynthetic_gene_cluster (100% of genes show similarity) | BGC0000340_c1 |  |  |
| Cluster 20 | Siderophore | 5052198 | 5063985 | Desferrioxamine_B_biosynthetic_gene_cluster (100% of genes show similarity) | BGC0000940_c1 |  |  |
| Cluster 21 | T1pks-Nrps | 5362278 | 5478116 | TP-1161_biosynthetic_gene_cluster (41% of genes show similarity) | BGC0000615_c1 | 1 | CYP105AU1 |
| Cluster 22 | Terpene | 6406844 | 6429246 | - | - | 1 | CYP147F5 |
| Cluster 23 | Nrps-Arylpolyene-Ladderane | 6723014 | 6828195 | Skyllamycin_biosynthetic_gene_cluster (44% of genes show similarity) | BGC0000429_c1 | 3 | CYP107CE1; CYP107CF1; CYP163B4 |
| Cluster 24 | Indole | 7126202 | 7147344 | 7-prenylisatin_biosynthetic_gene_cluster (40% of genes show similarity) | BGC0001294_c1 | 2 | CYP156B6; CYP107U9 |
| Cluster 25 | Nrps | 7363926 | 7407936 | Ochronotic_pigment_biosynthetic_gene_cluster (75% of genes show similarity) | BGC0000918_c1 |  |  |
| Cluster 26 | Arylpolyene-Ladderane | 7459089 | 7501424 | WS9326_biosynthetic_gene_cluster (25% of genes show similarity) | BGC0001297_c1 |  |  |
| Cluster 27 | T1pks | 7670799 | 7852915 | ECO-02301_biosynthetic_gene_cluster (57% of genes show similarity) | BGC0000052_c1 |  |  |
| Cluster 28 | Terpene | 8356513 | 8377778 | - | - | 2 | CYP125A20; CYP156C8 |
| Cluster 29 | Ectoine | 9039123 | 9049527 | Ectoine_biosynthetic_gene_cluster (100% of genes show similarity) | BGC0000853_c1 |  |  |
| Cluster 30 | T1pks-Siderophore | 9207893 | 9280685 | Apoptolidin_biosynthetic_gene_cluster (23% of genes show similarity) | BGC0000021_c1 |  |  |
| Cluster 31 | Terpene | 9291107 | 9312180 | Xiamycin_biosynthetic_gene_cluster (26% of genes show similarity) | BGC0000665_c1 |  |  |
| Cluster 32 | T1pks | 9618433 | 9752874 | Oligomycin_biosynthetic_gene_cluster (50% of genes show similarity) | BGC0000117_c1 | 3 | CYP194B3; CYP194B4; CYP107BS2 |
| Cluster 33 | Other | 9825530 | 9867362 | BD-12_biosynthetic_gene_cluster (75% of genes show similarity) | BGC0001379_c1 |  |  |
| Cluster 34 | T1pks-Nrps | 9905472 | 10151658 | Rapamycin_biosynthetic_gene_cluster (96% of genes show similarity) | BGC0001040_c1 | 3 | CYP105AW1; CYP122A4; CYP107G2 |
| Cluster 35 | Otherks | 10231970 | 10272998 | Galbonolides_biosynthetic_gene_cluster (20% of genes show similarity) | BGC0000065_c1 | 1 | CYP183F1 |
| Cluster 36 | Terpene | 10275433 | 10296563 | 2-methylisoborneol_biosynthetic_gene_cluster (100% of genes show similarity) | BGC0000658_c1 |  |  |
| Cluster 37 | Terpene | 10714282 | 10735382 | - | - |  |  |
| Cluster 38 | T1pks-Arylpolyene-Ladderane | 10828170 | 10879625 | Skyllamycin_biosynthetic_gene_cluster (18% of genes show similarity) | BGC0000429_c1 | 1 | CYP107B3 |
| Cluster 39 | T1pks-Nrps | 11126450 | 11244528 | Meridamycin_biosynthetic_gene_cluster (73% of genes show similarity) | BGC0001011_c1 | 1 | CYP105AY1 |
| Cluster 40 | T1pks | 11250323 | 11334466 | Hygrocin_biosynthetic_gene_cluster (90% of genes show similarity) | BGC0000075_c1 | 1 | CYP107AD1 |
| Cluster 41 | Bacteriocin-Nrps-Lantipeptide-T1pks-Otherks | 11489476 | 11707824 | Bafilomycin_biosynthetic_gene_cluster (50% of genes show similarity) | BGC0000028_c1 | 3 | CYP105AN3; CYP107E9; CYP155A5 |
| Cluster 42 | Terpene | 11918860 | 11939768 | - | - | 1 | CYP105AV1 |
| Cluster 43 | Other | 11989963 | 12032665 | Herboxidiene_biosynthetic_gene_cluster (4% of genes show similarity) | BGC0001065_c1 |  |  |
| Cluster 44 | T1pks | 12189709 | 12284210 | Concanamycin_A_biosynthetic_gene_cluster (21% of genes show similarity) | BGC0000040_c1 | 1 | CYP107CD1 |
| Cluster 45 | Nrps | 12397424 | 12450754 | - | - |  |  |
| Cluster 46 | T1pks | 12467081 | 12520518 | Geldanamycin_biosynthetic_gene_cluster (21% of genes show similarity) | BGC0000066_c1 |  |  |
| Cluster 47 | Terpene | 12663104 | 12688864 | Carotenoid_biosynthetic_gene_cluster (63% of genes show similarity) | BGC0000633_c1 |  |  |
| ***Streptomyces albulus* NK660** | | | | | | | |
| Cluster 1 | Butyrolactone | 191856 | 202725 | Chalcomycin_biosynthetic_gene_cluster (7% of genes show similarity) | BGC0000035_c1 | 1 | CYP107L43 |
| Cluster 2 | T1pks | 223312 | 268246 | Ansamitocin_biosynthetic_gene_cluster (12% of genes show similarity) | BGC0000020_c1 |  |  |
| Cluster 3 | Other | 293048 | 333884 | Guadinomine_biosynthetic_gene_cluster (7% of genes show similarity) | BGC0000998_c1 | 1 | CYP1190A1 |
| Cluster 4 | T1pks-Nrps | 384583 | 440308 | Sporolide_biosynthetic_gene_cluster (8% of genes show similarity) | BGC0000150_c1 |  |  |
| Cluster 5 | Other | 455350 | 498610 | Stenothricin_biosynthetic_gene_cluster (13% of genes show similarity) | BGC0000431_c1 |  |  |
| Cluster 6 | Other | 729299 | 772400 | - | - | 1 | CYP1192A1 |
| Cluster 7 | Lantipeptide | 802532 | 826984 | Himastatin_biosynthetic_gene_cluster (8% of genes show similarity) | BGC0001117_c1 |  |  |
| Cluster 8 | T1pks-Nrps | 956162 | 1047337 | Siomycin_biosynthetic_gene_cluster (7% of genes show similarity) | BGC0000611_c1 | 1 | CYP1191A1 |
| Cluster 9 | Nrps | 1251461 | 1315374 | Laspartomycin_biosynthetic_gene_cluster (11% of genes show similarity) | BGC0000379_c1 | 1 | CYP107EB1 |
| Cluster 10 | Transatpks-T1pks-Nrps | 1323278 | 1373000 | Cinnabaramide_biosynthetic_gene_cluster (18% of genes show similarity) | BGC0000971_c1 | 2 | CYP163B9; CYP105AA13 |
| Cluster 11 | T1pks | 1518010 | 1626108 | Nystatin_biosynthetic_gene_cluster (81% of genes show similarity) | BGC0000115_c1 | 2 | CYP105H9; CYP161A7 |
| Cluster 12 | Bacteriocin | 1681644 | 1691859 | - | - |  |  |
| Cluster 13 | T2pks | 1739194 | 1781709 | Spore_pigment_biosynthetic_gene_cluster (83% of genes show similarity) | BGC0000271_c1 |  |  |
| Cluster 14 | Lantipeptide-Lassopeptide | 2314663 | 2344101 | Streptomycin_biosynthetic_gene_cluster (8% of genes show similarity) | BGC0000717_c1 |  |  |
| Cluster 15 | Siderophore | 2603121 | 2614923 | Desferrioxamine_B_biosynthetic_gene_cluster (80% of genes show similarity) | BGC0000941_c1 |  |  |
| Cluster 16 | Ectoine | 2689910 | 2700320 | Ectoine_biosynthetic_gene_cluster (100% of genes show similarity) | BGC0000853_c1 |  |  |
| Cluster 17 | Terpene | 5036219 | 5058516 | Salinomycin_biosynthetic_gene_cluster (6% of genes show similarity) | BGC0000144_c1 |  |  |
| Cluster 18 | Transatpks-Nrps | 6189940 | 6292793 | Oxazolomycin_biosynthetic_gene_cluster (24% of genes show similarity) | BGC0001106_c1 | 3 | CYP107B6; CYP1189A1; CYP1189A2 |
| Cluster 19 | Siderophore | 6726080 | 6740335 | - | - |  |  |
| Cluster 20 | T3pks-Otherks-Butyrolactone-Nrps | 6807354 | 6922217 | Svaricin_biosynthetic_gene_cluster (15% of genes show similarity) | BGC0001382_c1 | 2 | CYP107F9; CYP163C3 |
| Cluster 21 | Lantipeptide | 7171814 | 7196375 | - | - |  |  |
| Cluster 22 | T2pks-Oligosaccharide-Nucleoside-Nrps | 7502025 | 7608293 | Rabelomycin_biosynthetic_gene_cluster (39% of genes show similarity) | BGC0000262_c1 | 2 | CYP113D6; CYP157C28 |
| Cluster 23 | Terpene | 7715089 | 7741766 | Hopene_biosynthetic_gene_cluster (61% of genes show similarity) | BGC0000663_c1 |  |  |
| Cluster 24 | Butyrolactone | 7992340 | 8048938 | Oxazolomycin_biosynthetic_gene_cluster (9% of genes show similarity) | BGC0001106_c1 |  |  |
| Cluster 25 | Butyrolactone | 8290273 | 8301271 | Coelimycin_biosynthetic_gene_cluster (8% of genes show similarity) | BGC0000038_c1 | 1 | CYP107AE9 |
| Cluster 26 | Nrps | 8435386 | 8490322 | Azicemicin_biosynthetic_gene_cluster (11% of genes show similarity) | BGC0000202_c1 |  |  |
| Cluster 27 | Lantipeptide | 8499891 | 8561229 | SAL-2242_biosynthetic_gene_cluster (100% of genes show similarity) | BGC0000546_c1 | 1 | CYP251G1 |
| Cluster 28 | Butyrolactone | 8599016 | 8609951 | Lactonamycin_biosynthetic_gene_cluster (7% of genes show similarity) | BGC0000238_c1 |  |  |
| Cluster 29 | Other | 8842091 | 8884604 | Stenothricin_biosynthetic_gene_cluster (13% of genes show similarity) | BGC0000431_c1 |  |  |
| Cluster 30 | Terpene-T1pks | 8946818 | 9103383 | Nystatin_biosynthetic_gene_cluster (81% of genes show similarity) | BGC0000115_c1 | 2 | CYP161A6; CYP105H1 |
| Cluster 31 | T1pks | 9157043 | 9240750 | Kedarcidin_biosynthetic_gene_cluster (4% of genes show similarity) | BGC0000081_c1 |  |  |
| Cluster 32 | Lantipeptide | 9260281 | 9288895 | 9-methylstreptimidone_biosynthetic_gene_cluster (25% of genes show similarity) | BGC0000171_c1 |  |  |
| Cluster 33 | T1pks | 9330879 | 9360281 | - | - |  |  |
| ***Streptomyces albus* ZPM** | | | | | | | |
| Cluster 1 | Nrps | 17026 | 109753 | Himastatin_biosynthetic_gene_cluster (24% of genes show similarity) | BGC0001117_c1 | 3 | CYP107EL1; CYP163B8; CYP107EA2 |
| Cluster 2 | Butyrolactone | 330907 | 341776 | Chalcomycin_biosynthetic_gene_cluster (7% of genes show similarity) | BGC0000035_c1 | 1 | CYP107L43 |
| Cluster 3 | Other | 433815 | 474639 | Guadinomine_biosynthetic_gene_cluster (7% of genes show similarity) | BGC0000998_c1 | 1 | CYP1190A1 |
| Cluster 4 | T1pks-Nrps | 526610 | 582304 | Sporolide_biosynthetic_gene_cluster (8% of genes show similarity) | BGC0000150_c1 |  |  |
| Cluster 5 | Other | 596797 | 640057 | Stenothricin_biosynthetic_gene_cluster (13% of genes show similarity) | BGC0000431_c1 |  |  |
| Cluster 6 | Other | 883066 | 926167 | Tetarimycin_biosynthetic_gene_cluster (5% of genes show similarity) | BGC0000274_c1 | 3 | CYP147F21; CYP1060A2; CYP1192A1 |
| Cluster 7 | Lantipeptide | 954336 | 978788 | Himastatin_biosynthetic_gene_cluster (8% of genes show similarity) | BGC0001117_c1 |  |  |
| Cluster 8 | T1pks | 1103165 | 1150568 | Hygrocin_biosynthetic_gene_cluster (6% of genes show similarity) | BGC0000075_c1 | 1 | CYP1191A1 |
| Cluster 9 | Nrps | 1413155 | 1468194 | Carbapenem_MM_4550_biosynthetic_gene_cluster (6% of genes show similarity) | BGC0000842_c1 |  |  |
| Cluster 10 | Transatpks-T1pks-Nrps | 1476172 | 1525894 | Cinnabaramide_biosynthetic_gene_cluster (18% of genes show similarity) | BGC0000971_c1 | 2 | CYP163B9; CYP105AA13 |
| Cluster 11 | T1pks | 1670952 | 1716162 | Rimocidin_biosynthetic_gene_cluster (63% of genes show similarity) | BGC0000138_c1 | 1 | CYP105H9 |
| Cluster 12 | Bacteriocin | 1838897 | 1849112 | - | - |  |  |
| Cluster 13 | T2pks | 1896764 | 1939279 | Spore_pigment_biosynthetic_gene_cluster (83% of genes show similarity) | BGC0000271_c1 |  |  |
| Cluster 14 | Siderophore | 2770965 | 2782767 | Desferrioxamine_B_biosynthetic_gene_cluster (80% of genes show similarity) | BGC0000941_c1 |  |  |
| Cluster 15 | Siderophore | 2865668 | 2880351 | - | - |  |  |
| Cluster 16 | Ectoine | 3021273 | 3031683 | Ectoine_biosynthetic_gene_cluster (100% of genes show similarity) | BGC0000853_c1 |  |  |
| Cluster 17 | Terpene | 5358147 | 5380444 | Salinomycin_biosynthetic_gene_cluster (6% of genes show similarity) | BGC0000144_c1 |  |  |
| Cluster 18 | Other | 6045012 | 6089226 | Meridamycin_biosynthetic_gene_cluster (5% of genes show similarity) | BGC0001011_c1 |  |  |
| Cluster 19 | Transatpks-Nrps | 6554322 | 6638419 | Oxazolomycin_biosynthetic_gene_cluster (24% of genes show similarity) | BGC0001106_c1 | 2 | CYP1189A1; CYP1189A2 |
| Cluster 20 | T3pks | 7015396 | 7056448 | FD-594_biosynthetic_gene_cluster (8% of genes show similarity) | BGC0000222_c1 | 1 | CYP107F9 |
| Cluster 21 | Otherks-Butyrolactone-Nrps | 7041728 | 7131800 | FD-594_biosynthetic_gene_cluster (8% of genes show similarity) | BGC0000222_c1 | 1 | CYP163C3 |
| Cluster 22 | Lantipeptide | 7528854 | 7553415 | - | - |  |  |
| Cluster 23 | T2pks-Oligosaccharide-Nucleoside-Nrps | 7788124 | 7894680 | Rabelomycin_biosynthetic_gene_cluster (39% of genes show similarity) | BGC0000262_c1 | 2 | CYP113D6; CYP157C28 |
| Cluster 24 | Terpene | 8001771 | 8028454 | Hopene_biosynthetic_gene_cluster (61% of genes show similarity) | BGC0000663_c1 |  |  |
| Cluster 25 | Butyrolactone | 8283727 | 8340249 | Oxazolomycin_biosynthetic_gene_cluster (9% of genes show similarity) | BGC0001106_c1 |  |  |
| Cluster 26 | Butyrolactone | 8585464 | 8596462 | Coelimycin_biosynthetic_gene_cluster (8% of genes show similarity) | BGC0000038_c1 | 1 | CYP107AE9 |
| Cluster 27 | Nrps | 8706076 | 8762919 | Azicemicin_biosynthetic_gene_cluster (13% of genes show similarity) | BGC0000202_c1 |  |  |
| Cluster 28 | Lantipeptide | 8771860 | 8833243 | SapB_biosynthetic_gene_cluster (75% of genes show similarity) | BGC0000551_c1 | 1 | CYP251G1 |
| Cluster 29 | Butyrolactone | 8870590 | 8881525 | Lactonamycin_biosynthetic_gene_cluster (7% of genes show similarity) | BGC0000238_c1 |  |  |
| Cluster 30 | Other | 9116811 | 9160601 | Stenothricin_biosynthetic_gene_cluster (13% of genes show similarity) | BGC0000431_c1 |  |  |
| Cluster 31 | T1pks | 9240892 | 9287062 | Nystatin_biosynthetic_gene_cluster (50% of genes show similarity) | BGC0000115_c1 | 2 | CYP161A6; CYP105H1 |
| Cluster 32 | Terpene | 9358138 | 9379196 | Marinacarboline_biosynthetic_gene_cluster (23% of genes show similarity) | BGC0001137_c1 |  |  |
| Cluster 33 | Other | 9444178 | 9526669 | Toxoflavin_biosynthetic_gene_cluster (50% of genes show similarity) | BGC0000929_c1 |  |  |
| Cluster 34 | Lantipeptide | 9545426 | 9574041 | 9-methylstreptimidone_biosynthetic_gene_cluster (25% of genes show similarity) | BGC0000171_c1 |  |  |
| Cluster 35 | Nrps | 9674825 | 9767552 | Himastatin_biosynthetic_gene_cluster (24% of genes show similarity) | BGC0001117_c1 | 3 | CYP107EA2; CYP163B8; CYP107EL1 |
| ***Streptomyces lividans*** | | | | | | | |
| Cluster 1 | Terpene | 16436 | 37485 | A54145_biosynthetic_gene_cluster (8% of genes show similarity) | BGC0000291_c1 |  |  |
| Cluster 2 | T3pks-Terpene-Nrps | 135815 | 218933 | Coelibactin_biosynthetic_gene_cluster (100% of genes show similarity) | BGC0000324_c1 | 1 | CYP105N1 |
| Cluster 3 | Indole | 408329 | 429456 | Ravidomycin_biosynthetic_gene_cluster (5% of genes show similarity) | BGC0000263_c1 |  |  |
| Cluster 4 | Other | 683230 | 724117 | - | - |  |  |
| Cluster 5 | Terpene | 1013200 | 1039941 | Hopene_biosynthetic_gene_cluster (92% of genes show similarity) | BGC0000663_c1 |  |  |
| Cluster 6 | Lantipeptide | 1112999 | 1135713 | SapB_biosynthetic_gene_cluster (100% of genes show similarity) | BGC0000551_c1 |  |  |
| Cluster 7 | Nrps | 1379776 | 1427601 | Nogalamycin_biosynthetic_gene_cluster (40% of genes show similarity) | BGC0000249_c1 |  |  |
| Cluster 8 | Butyrolactone-T1pks | 1504046 | 1584720 | Coelimycin_biosynthetic_gene_cluster (100% of genes show similarity) | BGC0000038_c1 |  |  |
| Cluster 9 | Siderophore | 1600479 | 1613686 | Enduracidin_biosynthetic_gene_cluster (8% of genes show similarity) | BGC0000341_c1 |  |  |
| Cluster 10 | Terpene | 1777516 | 1799696 | Herboxidiene_biosynthetic_gene_cluster (2% of genes show similarity) | BGC0001065_c1 |  |  |
| Cluster 11 | Bacteriocin | 1811789 | 1823105 | - | - |  |  |
| Cluster 12 | T1pks | 2069522 | 2116415 | Undecylprodigiosin_biosynthetic_gene_cluster (100% of genes show similarity) | BGC0001063_c1 |  |  |
| Cluster 13 | Siderophore | 2198321 | 2210267 | - | - |  |  |
| Cluster 14 | T2pks | 2687566 | 2730108 | Spore_pigment_biosynthetic_gene_cluster (66% of genes show similarity) | BGC0000271_c1 |  |  |
| Cluster 15 | Terpene | 2804079 | 2825092 | Albaflavenone_biosynthetic_gene_cluster (100% of genes show similarity) | BGC0000660_c1 | 1 | CYP170A1 |
| Cluster 16 | T2pks | 2943724 | 2986218 | Actinorhodin_biosynthetic_gene_cluster (100% of genes show similarity) | BGC0000194_c1 |  |  |
| Cluster 17 | Nrps | 4737916 | 4818557 | Calcium-dependent_antibiotic_biosynthetic_gene_cluster (90% of genes show similarity) | BGC0000315_c1 |  |  |
| Cluster 18 | Siderophore | 5290107 | 5301891 | Desferrioxamine_B_biosynthetic_gene_cluster (100% of genes show similarity) | BGC0000940_c1 |  |  |
| Cluster 19 | Melanin | 5384385 | 5394954 | Lactonamycin_biosynthetic_gene_cluster (3% of genes show similarity) | BGC0000238_c1 |  |  |
| Cluster 20 | Ectoine | 6336408 | 6346806 | Ectoine_biosynthetic_gene_cluster (100% of genes show similarity) | BGC0000853_c1 |  |  |
| Cluster 21 | T3pks | 7041742 | 7082866 | Herboxidiene_biosynthetic_gene_cluster (8% of genes show similarity) | BGC0001065_c1 | 1 | CYP158A2 |
| Cluster 22 | Bacteriocin | 7570510 | 7580725 | Informatipeptin_biosynthetic_gene_cluster (42% of genes show similarity) | BGC0000518_c1 |  |  |
| Cluster 23 | Nrps | 7826445 | 7877382 | Coelichelin_biosynthetic_gene_cluster (100% of genes show similarity) | BGC0000325_c1 |  |  |
| Cluster 24 | Lantipeptide | 8100785 | 8135884 | Sanglifehrin_A_biosynthetic_gene_cluster (4% of genes show similarity) | BGC0001042_c1 |  |  |
| Cluster 25 | Terpene | 8180092 | 8205599 | Isorenieratene_biosynthetic_gene_cluster (100% of genes show similarity) | BGC0000664_c1 |  |  |
| Cluster 26 | T1pks-Otherks | 8241896 | 8294872 | Leinamycin_biosynthetic_gene_cluster (2% of genes show similarity) | BGC0001101_c1 |  |  |
| Cluster 27 | Terpene | 8307799 | 8328848 | Glycopeptidolipid_biosynthetic_gene_cluster (10% of genes show similarity) | BGC0000362_c1 |  |  |
| ***Streptomyces glaucescens*** | | | | | | | |
| Cluster 1 | Thiopeptide | 154073 | 181725 | Lactazole_biosynthetic_gene_cluster (55% of genes show similarity) | BGC0000606_c1 |  |  |
| Cluster 2 | Other | 193911 | 235617 | Acarviostatin_biosynthetic_gene_cluster (37% of genes show similarity) | BGC0000804_c1 | 1 | CYP178B1 |
| Cluster 3 | Amglyccycl | 273980 | 300700 | 5'-hydroxystreptomycin_biosynthetic_gene_cluster (95% of genes show similarity) | BGC0000690_c1 |  |  |
| Cluster 4 | Terpene | 365767 | 391614 | Carotenoid_biosynthetic_gene_cluster (63% of genes show similarity) | BGC0000633_c1 |  |  |
| Cluster 5 | Melanin | 622415 | 632819 | Melanin_biosynthetic_gene_cluster (57% of genes show similarity) | BGC0000908_c1 |  |  |
| Cluster 6 | Nrps | 994531 | 1107137 | SW-163_biosynthetic_gene_cluster (32% of genes show similarity) | BGC0000434_c1 | 1 | CYP163B10 |
| Cluster 7 | Other | 1120487 | 1161569 | BE-14106_biosynthetic_gene_cluster (17% of genes show similarity) | BGC0000029_c1 |  |  |
| Cluster 8 | T1pks-Otherks | 1613939 | 1668625 | Xantholipin_biosynthetic_gene_cluster (6% of genes show similarity) | BGC0000279_c1 |  |  |
| Cluster 9 | Ectoine | 1897008 | 1907412 | Ectoine_biosynthetic_gene_cluster (100% of genes show similarity) | BGC0000853_c1 |  |  |
| Cluster 10 | Melanin | 2820776 | 2831324 | Melanin_biosynthetic_gene_cluster (80% of genes show similarity) | BGC0000909_c1 |  |  |
| Cluster 11 | Siderophore | 2914060 | 2925829 | Desferrioxamine_B_biosynthetic_gene_cluster (100% of genes show similarity) | BGC0000940_c1 |  |  |
| Cluster 12 | Terpene | 5085549 | 5106472 | Albaflavenone_biosynthetic_gene_cluster (100% of genes show similarity) | BGC0000660_c1 | 1 | CYP170A22 |
| Cluster 13 | Siderophore | 5682914 | 5694878 | - | - |  |  |
| Cluster 14 | Lassopeptide | 5766933 | 5789727 | - | - |  |  |
| Cluster 15 | Butyrolactone | 5802942 | 5813874 | Coelimycin_biosynthetic_gene_cluster (8% of genes show similarity) | BGC0000038_c1 |  |  |
| Cluster 16 | Bacteriocin | 5903335 | 5914678 | - | - |  |  |
| Cluster 17 | Terpene | 5942344 | 5964521 | - | - | 1 | CYP180A9 |
| Cluster 18 | T2pks | 5992056 | 6034562 | Tetracenomycin_biosynthetic_gene_cluster (100% of genes show similarity) | BGC0000275_c1 |  |  |
| Cluster 19 | Siderophore | 6106223 | 6119335 | Grincamycin_biosynthetic_gene_cluster (5% of genes show similarity) | BGC0000229_c1 |  |  |
| Cluster 20 | Terpene | 6451173 | 6478211 | Hopene_biosynthetic_gene_cluster (69% of genes show similarity) | BGC0000663_c1 |  |  |
| Cluster 21 | T2pks | 6563775 | 6640031 | Spore_pigment_biosynthetic_gene_cluster (66% of genes show similarity) | BGC0000271_c1 | 1 | CYP107AH4 |
| Cluster 22 | Terpene | 6811706 | 6832737 | - | - | 1 | CYP157K3 |
| Cluster 23 | Bacteriocin | 6851421 | 6861636 | Informatipeptin_biosynthetic_gene_cluster (42% of genes show similarity) | BGC0000518_c1 |  |  |
| Cluster 24 | Lassopeptide | 7061912 | 7084480 | - | - |  |  |
| Cluster 25 | Nrps | 7094980 | 7173584 | Zorbamycin_biosynthetic_gene_cluster (8% of genes show similarity) | BGC0001058_c1 | 1 | CYP113K5 |
| ***Streptomyces vietnamensis*** | | | | | | | |
| Cluster 1 | Lantipeptide | 346045 | 373904 | Venezuelin_biosynthetic_gene_cluster (75% of genes show similarity) | BGC0000563_c1 |  |  |
| Cluster 2 | Nrps | 399968 | 455320 | Streptolydigin_biosynthetic_gene_cluster (10% of genes show similarity) | BGC0001046_c1 |  |  |
| Cluster 3 | Ectoine | 566113 | 576529 | Ectoine_biosynthetic_gene_cluster (100% of genes show similarity) | BGC0000853_c1 |  |  |
| Cluster 4 | Terpene | 641540 | 663738 | - | - |  |  |
| Cluster 5 | Butyrolactone | 690180 | 701109 | Coelimycin_biosynthetic_gene_cluster (8% of genes show similarity) | BGC0000038_c1 |  |  |
| Cluster 6 | Butyrolactone-T1pks-Nrps | 742368 | 867912 | Lankamycin_biosynthetic_gene_cluster (26% of genes show similarity) | BGC0000085_c1 |  |  |
| Cluster 7 | Other | 2414787 | 2455359 | Lomofungin_biosynthetic_gene_cluster (13% of genes show similarity) | BGC0001302_c1 | 1 | CYP121A2 |
| Cluster 8 | Siderophore | 3014427 | 3025998 | - | - |  |  |
| Cluster 9 | Siderophore | 3104104 | 3115873 | Desferrioxamine_B_biosynthetic_gene_cluster (100% of genes show similarity) | BGC0000941_c1 |  |  |
| Cluster 10 | T2pks | 4190266 | 4232787 | Granaticin_biosynthetic_gene_cluster (64% of genes show similarity) | BGC0000227_c1 |  |  |
| Cluster 11 | Other | 4692700 | 4736419 | Saframycin_A_biosynthetic_gene_cluster (12% of genes show similarity) | BGC0000422_c1 |  |  |
| Cluster 12 | Melanin | 5341238 | 5351639 | Istamycin_biosynthetic_gene_cluster (8% of genes show similarity) | BGC0000700_c1 |  |  |
| Cluster 13 | Other | 5791545 | 5832648 | Stambomycin_biosynthetic_gene_cluster (16% of genes show similarity) | BGC0000151_c1 |  |  |
| Cluster 14 | Thiopeptide | 5844801 | 5871088 | BD-12_biosynthetic_gene_cluster (14% of genes show similarity) | BGC0001379_c1 |  |  |
| Cluster 15 | Siderophore | 6229349 | 6241184 | - | - |  |  |
| Cluster 16 | T2pks-Nrps | 6341578 | 6422345 | Kinamycin_biosynthetic_gene_cluster (51% of genes show similarity) | BGC0000236_c1 | 3 | CYP1029A3; CYP1423A1; CYP285A5 |
| Cluster 17 | Bacteriocin | 6509824 | 6521212 | - | - |  |  |
| Cluster 18 | Butyrolactone | 6646523 | 6657473 | Griseoviridin_/_viridogrisein_biosynthetic_gene_cluster (11% of genes show similarity) | BGC0000459_c1 |  |  |
| Cluster 19 | Terpene | 7009307 | 7036249 | Hopene_biosynthetic_gene_cluster (69% of genes show similarity) | BGC0000663_c1 |  |  |
| Cluster 20 | Bacteriocin | 7087107 | 7097922 | - | - |  |  |
| Cluster 21 | Other | 7175741 | 7217552 | Enduracidin_biosynthetic_gene_cluster (14% of genes show similarity) | BGC0000341_c1 |  |  |
| Cluster 22 | Terpene | 7297687 | 7319081 | Griseobactin_biosynthetic_gene_cluster (11% of genes show similarity) | BGC0000368_c1 |  |  |
| Cluster 23 | Lantipeptide | 7778604 | 7802417 | - | - | 1 | CYP107LD1 |
| Cluster 24 | T2pks-Lantipeptide-Terpene | 7912992 | 7991337 | Spore_pigment_biosynthetic_gene_cluster (83% of genes show similarity) | BGC0000271_c1 | 2 | CYP154C11; CYP157A19 |
| Cluster 25 | Other | 8287563 | 8331471 | - | - |  |  |
| Cluster 26 | T1pks-Nrps | 8367712 | 8417038 | Heat-stable_antifungal_factor_biosynthetic_gene_cluster (75% of genes show similarity) | BGC0000999_c1 |  |  |
| Cluster 27 | Lantipeptide | 8457714 | 8484769 | A54145_biosynthetic_gene_cluster (5% of genes show similarity) | BGC0000291_c1 |  |  |
| ***Streptomyces* sp. 769** | | | | | | | |
| Cluster 1 | Butyrolactone-T1pks-Nrps | 37787 | 174548 | Coelimycin_biosynthetic_gene_cluster (29% of genes show similarity) | BGC0000038_c1 | 1 | CYP105BA3 |
| Cluster 2 | T1pks-Arylpolyene | 205753 | 287809 | Ansamitocin_biosynthetic_gene_cluster (14% of genes show similarity) | BGC0000020_c1 | 3 | CYP107EM1; CYP1198A1; CYP105BV1 |
| Cluster 3 | Terpene | 390343 | 411401 | 2-methylisoborneol_biosynthetic_gene_cluster (100% of genes show similarity) | BGC0000658_c1 |  |  |
| Cluster 4 | Lantipeptide-Nrps | 462632 | 544432 | A-503083_biosynthetic_gene_cluster (7% of genes show similarity) | BGC0000288_c1 |  |  |
| Cluster 5 | Terpene | 605351 | 626247 | - | - |  |  |
| Cluster 6 | Terpene | 705884 | 726852 | - | - | 1 | CYP157C31 |
| Cluster 7 | T1pks | 796740 | 940715 | Nystatin_biosynthetic_gene_cluster (100% of genes show similarity) | BGC0000115_c1 | 2 | CYP105H1; CYP161A6 |
| Cluster 8 | Butyrolactone | 1390494 | 1401339 | Coelimycin_biosynthetic_gene_cluster (8% of genes show similarity) | BGC0000038_c1 | 1 | CYP107AE10 |
| Cluster 9 | Bacteriocin-Butyrolactone | 1650849 | 1758890 | Sch47554_/_Sch47555_biosynthetic_gene_cluster (10% of genes show similarity) | BGC0000268_c1 |  |  |
| Cluster 10 | Terpene | 2074720 | 2101403 | Hopene_biosynthetic_gene_cluster (61% of genes show similarity) | BGC0000663_c1 |  |  |
| Cluster 11 | Lassopeptide | 2283178 | 2305712 | - | - |  |  |
| Cluster 12 | T3pks | 2967642 | 3008706 | FD-594_biosynthetic_gene_cluster (8% of genes show similarity) | BGC0000222_c1 | 1 | CYP107F9 |
| Cluster 13 | Nrps | 3068017 | 3130113 | Desotamide_biosynthetic_gene_cluster (63% of genes show similarity) | BGC0001196_c1 |  |  |
| Cluster 14 | Siderophore | 3136814 | 3151493 | - | - |  |  |
| Cluster 15 | Other | 3470479 | 3511162 | Albonoursin_biosynthetic_gene_cluster (83% of genes show similarity) | BGC0000851_c1 |  |  |
| Cluster 16 | T1pks | 3951761 | 4057662 | Aldgamycin_biosynthetic_gene_cluster (47% of genes show similarity) | BGC0001396_c1 | 3 | CYP105DE1; CYP105BS1; CYP1197A1 |
| Cluster 17 | Other | 4149655 | 4193803 | - | - |  |  |
| Cluster 18 | Linaridin | 4625017 | 4645924 | Legonaridin_biosynthetic_gene_cluster (55% of genes show similarity) | BGC0001188_c1 |  |  |
| Cluster 19 | Terpene | 4789780 | 4812017 | Salinomycin_biosynthetic_gene_cluster (6% of genes show similarity) | BGC0000144_c1 |  |  |
| Cluster 20 | Lantipeptide | 6168869 | 6193334 | Melanin_biosynthetic_gene_cluster (40% of genes show similarity) | BGC0000909_c1 |  |  |
| Cluster 21 | Ectoine | 7150807 | 7161211 | Ectoine_biosynthetic_gene_cluster (100% of genes show similarity) | BGC0000853_c1 |  |  |
| Cluster 22 | Siderophore | 7239980 | 7251785 | Desferrioxamine_B_biosynthetic_gene_cluster (80% of genes show similarity) | BGC0000941_c1 |  |  |
| Cluster 23 | Thiopeptide-T1pks | 7382358 | 7445646 | Virginiamycin_biosynthetic_gene_cluster (33% of genes show similarity) | BGC0001116_c1 | 1 | CYP184A8 |
| Cluster 24 | Lantipeptide | 7631682 | 7669877 | Streptomycin_biosynthetic_gene_cluster (16% of genes show similarity) | BGC0000717_c1 |  |  |
| Cluster 25 | Nrps | 7834376 | 7918670 | Streptolydigin_biosynthetic_gene_cluster (15% of genes show similarity) | BGC0001046_c1 | 1 | CYP1196A1 |
| Cluster 26 | T2pks | 8232006 | 8274521 | Spore_pigment_biosynthetic_gene_cluster (83% of genes show similarity) | BGC0000271_c1 |  |  |
| Cluster 27 | Bacteriocin | 8327176 | 8337427 | - | - |  |  |
| Cluster 28 | T1pks | 8699010 | 8746527 | Zorbamycin_biosynthetic_gene_cluster (6% of genes show similarity) | BGC0001058_c1 |  |  |
| Cluster 29 | Transatpks-Nrps | 8764353 | 8873800 | Cycloheximide_/_actiphenol_biosynthetic_gene_cluster (50% of genes show similarity) | BGC0000175_c1 | 2 | CYP107CA2; CYP154D14 |
| Cluster 30 | Nrps | 8897613 | 8945199 | A54145_biosynthetic_gene_cluster (6% of genes show similarity) | BGC0000291_c1 |  |  |
| Cluster 31 | Lantipeptide | 8994980 | 9034826 | Hygrocin_biosynthetic_gene_cluster (6% of genes show similarity) | BGC0000075_c1 | 1 | CYP107L42 |
| Cluster 32 | Terpene-T3pks-T1pks-Nrps | 9424609 | 9517486 | Friulimicin_biosynthetic_gene_cluster (9% of genes show similarity) | BGC0000354_c1 |  |  |
| Cluster 33 | T1pks-Otherks | 9531023 | 9582274 | - | - |  |  |
| Cluster 34 | Lantipeptide-Nrps | 9598330 | 9713563 | Zorbamycin_biosynthetic_gene_cluster (8% of genes show similarity) | BGC0001058_c1 | 2 | CYP163B; CYP1278B1 |
| Cluster 35 | Terpene-T1pks | 9773372 | 9896021 | Natamycin_biosynthetic_gene_cluster (68% of genes show similarity) | BGC0000108_c1 | 1 | CYP105H8 |
| Cluster 36 | T1pks-Butyrolactone-Nrps | 9926227 | 10062988 | Coelimycin_biosynthetic_gene_cluster (29% of genes show similarity) | BGC0000038_c1 | 1 | CYP105BA3 |
| ***Streptomyces cyaneogriseus*** | | | | | | | |
| Cluster 1 | T1pks | 181794 | 235227 | Pristinamycin_biosynthetic_gene_cluster (7% of genes show similarity) | BGC0000952_c3 | 2 | CYP107L35; CYP105BT1 |
| Cluster 2 | Terpene | 343990 | 368554 | Carotenoid_biosynthetic_gene_cluster (45% of genes show similarity) | BGC0000633_c1 |  |  |
| Cluster 3 | T2pks | 511967 | 554485 | Spore_pigment_biosynthetic_gene_cluster (83% of genes show similarity) | BGC0000271_c1 |  |  |
| Cluster 4 | Transatpks-T1pks-Nrps | 751250 | 905378 | Tautomycin_biosynthetic_gene_cluster (27% of genes show similarity) | BGC0000159_c1 |  |  |
| Cluster 5 | Nrps | 1136002 | 1186077 | Azicemicin_biosynthetic_gene_cluster (8% of genes show similarity) | BGC0000202_c1 |  |  |
| Cluster 6 | T3pks | 1300604 | 1341668 | BE-14106_biosynthetic_gene_cluster (17% of genes show similarity) | BGC0000029_c1 |  |  |
| Cluster 7 | Other | 1422519 | 1464243 | Kirromycin_biosynthetic_gene_cluster (3% of genes show similarity) | BGC0001070_c1 |  |  |
| Cluster 8 | Ectoine | 1984280 | 1994678 | Ectoine_biosynthetic_gene_cluster (100% of genes show similarity) | BGC0000853_c1 |  |  |
| Cluster 9 | Butyrolactone | 2792194 | 2803150 | Lactonamycin_biosynthetic_gene_cluster (3% of genes show similarity) | BGC0000238_c1 |  |  |
| Cluster 10 | Melanin | 2865688 | 2876242 | Melanin_biosynthetic_gene_cluster (60% of genes show similarity) | BGC0000909_c1 |  |  |
| Cluster 11 | Siderophore | 2968691 | 2980460 | Desferrioxamine_B_biosynthetic_gene_cluster (100% of genes show similarity) | BGC0000940_c1 |  |  |
| Cluster 12 | Lassopeptide | 4593899 | 4616468 | TP-1161_biosynthetic_gene_cluster (20% of genes show similarity) | BGC0000615_c1 |  |  |
| Cluster 13 | Terpene | 5096111 | 5117199 | Albaflavenone_biosynthetic_gene_cluster (100% of genes show similarity) | BGC0000660_c1 | 1 | CYP170A17 |
| Cluster 14 | T1pks | 5255104 | 5335576 | Oligomycin_biosynthetic_gene_cluster (44% of genes show similarity) | BGC0000117_c1 | 1 | CYP107W3 |
| Cluster 15 | Other | 5447763 | 5489730 | BD-12_biosynthetic_gene_cluster (17% of genes show similarity) | BGC0001379_c1 |  |  |
| Cluster 16 | Siderophore | 5842192 | 5854036 | - | - |  |  |
| Cluster 17 | Bacteriocin | 6155544 | 6166959 | - | - |  |  |
| Cluster 18 | Terpene | 6194937 | 6217144 | Herboxidiene_biosynthetic_gene_cluster (2% of genes show similarity) | BGC0001065_c1 |  |  |
| Cluster 19 | Siderophore | 6382826 | 6396032 | - | - | 1 | CYP105AC8 |
| Cluster 20 | Terpene | 6517415 | 6538428 | Pentalenolactone_biosynthetic_gene_cluster (58% of genes show similarity) | BGC0000653_c1 | 2 | CYP161C4; CYP183A4 |
| Cluster 21 | T1pks | 6613280 | 6660692 | - | - |  |  |
| Cluster 22 | Terpene | 6884308 | 6910993 | Hopene_biosynthetic_gene_cluster (84% of genes show similarity) | BGC0000663_c1 |  |  |
| Cluster 23 | Terpene | 6974573 | 6995385 | Isorenieratene_biosynthetic_gene_cluster (18% of genes show similarity) | BGC0001227_c1 |  |  |
| Cluster 24 | Transatpks-Nrps | 7022565 | 7087583 | Reveromycin_biosynthetic_gene_cluster (9% of genes show similarity) | BGC0000135_c1 |  |  |
| Cluster 25 | Otherks-Nrps | 7273843 | 7389100 | Taromycin_biosynthetic_gene_cluster (26% of genes show similarity) | BGC0000439_c1 |  |  |
| Cluster 26 | Terpene | 7480737 | 7501765 | - | - | 1 | CYP157K4 |
| Cluster 27 | Bacteriocin | 7518472 | 7528687 | Informatipeptin_biosynthetic_gene_cluster (42% of genes show similarity) | BGC0000518_c1 |  |  |
| Cluster 28 | T2pks | 7575898 | 7618389 | Enterocin_biosynthetic_gene_cluster (55% of genes show similarity) | BGC0000220_c1 |  |  |
| Cluster 29 | T3pks-Fused-Nrps | 7679429 | 7762396 | Pheganomycin_biosynthetic_gene_cluster (23% of genes show similarity) | BGC0001148_c1 | 1 | CYP105AC17 |
| ***Streptomyces lydicus* A02** | | | | | | | |
| Cluster 1 | T2pks | 1 | 36695 | Xantholipin_biosynthetic_gene_cluster (46% of genes show similarity) | BGC0000279_c1 | 1 | CYP105BW1 |
| Cluster 2 | Melanin | 105776 | 116342 | - | - |  |  |
| Cluster 3 | Thiopeptide | 155035 | 187474 | A54145_biosynthetic_gene_cluster (3% of genes show similarity) | BGC0000291_c1 |  |  |
| Cluster 4 | Transatpks | 215263 | 294994 | Dorrigocin_/_migrastatin_biosynthetic_gene_cluster (72% of genes show similarity) | BGC0000177_c1 | 2 | CYP147K2; CYP1038A7 |
| Cluster 5 | Terpene | 366916 | 388229 | 2-methylisoborneol_biosynthetic_gene_cluster (75% of genes show similarity) | BGC0000657_c1 | 1 | CYP147F28P |
| Cluster 6 | Butyrolactone | 514149 | 525132 | Meilingmycin_biosynthetic_gene_cluster (3% of genes show similarity) | BGC0000093_c1 | 1 | CYP107AE8 |
| Cluster 7 | Ladderane | 697287 | 738447 | Chloramphenicol_biosynthetic_gene_cluster (11% of genes show similarity) | BGC0000893_c1 |  |  |
| Cluster 8 | Other | 942629 | 983345 | Streptomycin_biosynthetic_gene_cluster (4% of genes show similarity) | BGC0000717_c1 | 1 | CYP1469A2 |
| Cluster 9 | Terpene | 1000786 | 1022123 | Frankiamicin_biosynthetic_gene_cluster (21% of genes show similarity) | BGC0001197_c1 |  |  |
| Cluster 10 | T2pks-Terpene | 1213663 | 1277337 | Spore_pigment_biosynthetic_gene_cluster (83% of genes show similarity) | BGC0000271_c1 |  |  |
| Cluster 11 | Lantipeptide | 1349036 | 1373093 | - | - |  |  |
| Cluster 12 | Terpene | 1435606 | 1495524 | Siomycin_biosynthetic_gene_cluster (11% of genes show similarity) | BGC0000655_c1 | 1 | CYP157C29 |
| Cluster 13 | Cyanobactin-T1pks-Nrps | 1582697 | 1669681 | BE-14106_biosynthetic_gene_cluster (10% of genes show similarity) | BGC0000029_c1 |  |  |
| Cluster 14 | T3pks-Nrps | 1677418 | 1775435 | Kanamycin_biosynthetic_gene_cluster (7% of genes show similarity) | BGC0000703_c1 | 2 | CYP107F8; CYP105D25 |
| Cluster 15 | Lantipeptide | 1792777 | 1817670 | Planosporicin_biosynthetic_gene_cluster (100% of genes show similarity) | BGC0000544_c1 |  |  |
| Cluster 16 | Bacteriocin-Nrps | 2117695 | 2188684 | Enduracidin_biosynthetic_gene_cluster (16% of genes show similarity) | BGC0000341_c1 |  |  |
| Cluster 17 | Butyrolactone | 2305530 | 2316477 | Neocarzinostatin_biosynthetic_gene_cluster (4% of genes show similarity) | BGC0000112_c1 |  |  |
| Cluster 18 | Siderophore | 2460921 | 2475144 | - | - |  |  |
| Cluster 19 | Terpene | 4148488 | 4170725 | Salinomycin_biosynthetic_gene_cluster (6% of genes show similarity) | BGC0000144_c1 |  |  |
| Cluster 20 | Terpene | 5784113 | 5805222 | - | - | 1 | CYP157C42 |
| Cluster 21 | Lassopeptide | 6126895 | 6149273 | Chaxapeptin_biosynthetic_gene_cluster (42% of genes show similarity) | BGC0001307_c1 |  |  |
| Cluster 22 | Lassopeptide | 6229829 | 6252047 | - | - |  |  |
| Cluster 23 | Ectoine | 6717405 | 6727821 | Ectoine_biosynthetic_gene_cluster (100% of genes show similarity) | BGC0000853_c1 |  |  |
| Cluster 24 | Siderophore | 6806328 | 6818127 | Desferrioxamine_B_biosynthetic_gene_cluster (80% of genes show similarity) | BGC0000941_c1 |  |  |
| Cluster 25 | T1pks | 6896429 | 6976696 | ECO-02301_biosynthetic_gene_cluster (46% of genes show similarity) | BGC0000052_c1 |  |  |
| Cluster 26 | T2pks-Oligosaccharide | 7320728 | 7395690 | Grincamycin_biosynthetic_gene_cluster (58% of genes show similarity) | BGC0000229_c1 | 1 | CYP113D5 |
| Cluster 27 | Siderophore | 7657288 | 7672394 | Scabichelin_biosynthetic_gene_cluster (20% of genes show similarity) | BGC0000423_c1 |  |  |
| Cluster 28 | Bacteriocin-Lantipeptide | 7921105 | 7949889 | Herbimycin_biosynthetic_gene_cluster (10% of genes show similarity) | BGC0000074_c1 |  |  |
| Cluster 29 | Lassopeptide-Nrps | 8201080 | 8256818 | Streptomycin_biosynthetic_gene_cluster (2% of genes show similarity) | BGC0000717_c1 | 2 | CYP1278B2; CYP163B11 |
| Cluster 30 | Nrps | 8258692 | 8317893 | Friulimicin_biosynthetic_gene_cluster (12% of genes show similarity) | BGC0000354_c1 |  |  |
| Cluster 31 | Other | 8473783 | 8514496 | A54145_biosynthetic_gene_cluster (3% of genes show similarity) | BGC0000291_c1 | 1 | CYP121A3 |
| Cluster 32 | T1pks | 8525251 | 8643939 | Natamycin_biosynthetic_gene_cluster (100% of genes show similarity) | BGC0000108_c1 | 2 | CYP161A5; CYP105H3 |
| Cluster 33 | T1pks | 8655758 | 8700065 | - | - | 1 | CYP186D1 |
| Cluster 34 | Lantipeptide | 8785297 | 8808044 | SAL-2242_biosynthetic_gene_cluster (60% of genes show similarity) | BGC0000546_c1 |  |  |
| Cluster 35 | Lantipeptide-Lassopeptide | 9136656 | 9205488 | A-503083_biosynthetic_gene_cluster (7% of genes show similarity) | BGC0000288_c1 |  |  |
| ***Streptomyces xiamenensis* 318** | | | | | | | |
| Cluster 1 | Lantipeptide-T1pks | 186123 | 258027 | Xiamenmycin_biosynthetic_gene_cluster (100% of genes show similarity) | BGC0000933_c1 |  |  |
| Cluster 2 | Siderophore | 856962 | 868779 | Desferrioxamine_B_biosynthetic_gene_cluster (60% of genes show similarity) | BGC0000941_c1 |  |  |
| Cluster 3 | Ectoine | 910771 | 921175 | Ectoine_biosynthetic_gene_cluster (100% of genes show similarity) | BGC0000853_c1 |  |  |
| Cluster 4 | Terpene | 1252926 | 1273975 | Chlortetracycline_biosynthetic_gene_cluster (5% of genes show similarity) | BGC0000209_c1 | 1 | CYP157C32 |
| Cluster 5 | Butyrolactone | 1925564 | 1936604 | - | - |  |  |
| Cluster 6 | T1pks-Nrps | 2569761 | 2618819 | Thioviridamide_biosynthetic_gene_cluster (10% of genes show similarity) | BGC0000625_c1 | 2 | CYP1029A4; CYP285A6 |
| Cluster 7 | Other | 2649340 | 2690476 | Simocyclinone_biosynthetic_gene_cluster (13% of genes show similarity) | BGC0000270_c1 |  |  |
| Cluster 8 | Butyrolactone | 3050670 | 3061389 | Methylenomycin_biosynthetic_gene_cluster (9% of genes show similarity) | BGC0000914_c1 |  |  |
| Cluster 9 | T2pks | 3777683 | 3820216 | Lomaiviticin_biosynthetic_gene_cluster (38% of genes show similarity) | BGC0000240_c1 |  |  |
| Cluster 10 | Lantipeptide-Linaridin | 3913176 | 3941259 | - | - | 1 | CYP1223B1 |
| Cluster 11 | Linaridin-T1pks-Lassopeptide-Nrps | 4332894 | 4397384 | Heat-stable_antifungal_factor_biosynthetic_gene_cluster (37% of genes show similarity) | BGC0000999_c1 | 1 | CYP107LF1 |
| Cluster 12 | Terpene | 4494948 | 4515865 | - | - | 1 | CYP183W1 |
| Cluster 13 | Thiopeptide | 4549081 | 4583346 | - | - |  |  |
| Cluster 14 | Siderophore | 4879386 | 4893738 | - | - |  |  |
| Cluster 15 | Nrps | 5106208 | 5164444 | Valinomycin_biosynthetic_gene_cluster (16% of genes show similarity) | BGC0000453_c1 |  |  |
| Cluster 16 | T1pks-Nrps | 5264533 | 5315551 | Carotenoid_biosynthetic_gene_cluster (18% of genes show similarity) | BGC0000633_c1 |  |  |
| Cluster 17 | Terpene | 5429897 | 5451770 | Hopene_biosynthetic_gene_cluster (30% of genes show similarity) | BGC0000663_c1 |  |  |
| Cluster 18 | T3pks-Terpene-Nrps | 5469251 | 5606895 | Herboxidiene_biosynthetic_gene_cluster (7% of genes show similarity) | BGC0001065_c1 | 1 | CYP107F11 |
| Cluster 19 | T1pks-Nrps | 5621426 | 5672095 | Akaeolide_biosynthetic_gene_cluster (12% of genes show similarity) | BGC0001199_c1 |  |  |
| Cluster 20 | Nrps | 5686309 | 5752451 | WS9326_biosynthetic_gene_cluster (15% of genes show similarity) | BGC0001297_c1 |  |  |
| ***Streptomyces* sp. Mg1** | | | | | | | |
| Cluster 1 | Terpene | 127319 | 152797 | Isorenieratene_biosynthetic_gene_cluster (85% of genes show similarity) | BGC0000664_c1 |  |  |
| Cluster 2 | T1pks | 501941 | 550367 | Maklamicin_biosynthetic_gene_cluster (6% of genes show similarity) | BGC0001288_c1 |  |  |
| Cluster 3 | Terpene-Nrps | 554730 | 625429 | Xantholipin_biosynthetic_gene_cluster (4% of genes show similarity) | BGC0000279_c1 | 1 | CYP154Q2 |
| Cluster 4 | Lantipeptide-Terpene | 745744 | 780037 | - | - | 2 | CYP157C26; CYP134A3 |
| Cluster 5 | Other | 949375 | 993295 | Divergolide_biosynthetic_gene_cluster (13% of genes show similarity) | BGC0001119_c1 | 1 | CYP105DF1 |
| Cluster 6 | Terpene | 1027112 | 1048392 | 2-methylisoborneol_biosynthetic_gene_cluster (100% of genes show similarity) | BGC0000658_c1 |  |  |
| Cluster 7 | T1pks | 1057302 | 1248586 | ECO-02301_biosynthetic_gene_cluster (75% of genes show similarity) | BGC0000052_c1 |  |  |
| Cluster 8 | Thiopeptide | 1270469 | 1302825 | Thaxtomin_biosynthetic_gene_cluster (18% of genes show similarity) | BGC0000444_c1 | 2 | CYP2238A1; CYP1048A3 |
| Cluster 9 | Terpene-Otherks | 1637598 | 1706261 | A201A_biosynthetic_gene_cluster (5% of genes show similarity) | BGC0001138_c1 | 2 | CYP154D15; CYP157C27 |
| Cluster 10 | Siderophore | 3145817 | 3157598 | Desferrioxamine_B_biosynthetic_gene_cluster (100% of genes show similarity) | BGC0000940_c1 |  |  |
| Cluster 11 | Lantipeptide | 4292765 | 4317277 | - | - |  |  |
| Cluster 12 | Lantipeptide | 5106228 | 5130687 | Meilingmycin_biosynthetic_gene_cluster (2% of genes show similarity) | BGC0000093_c1 |  |  |
| Cluster 13 | Siderophore | 5851645 | 5863480 | - | - |  |  |
| Cluster 14 | Bacteriocin | 6103526 | 6114881 | - | - |  |  |
| Cluster 15 | Terpene | 6180567 | 6202783 | - | - |  |  |
| Cluster 16 | Terpene | 6479912 | 6506775 | Hopene_biosynthetic_gene_cluster (61% of genes show similarity) | BGC0000663_c1 |  |  |
| Cluster 17 | Lantipeptide | 6634722 | 6657028 | - | - |  |  |
| Cluster 18 | T1pks | 6892260 | 6958618 | Aldgamycin_biosynthetic_gene_cluster (44% of genes show similarity) | BGC0001396_c1 | 2 | CYP105L3; CYP1995C1 |
| Cluster 19 | Lantipeptide | 6975153 | 7013297 | SapB_biosynthetic_gene_cluster (75% of genes show similarity) | BGC0000551_c1 |  |  |
| Cluster 20 | Melanin | 7093620 | 7121271 | Herboxidiene_biosynthetic_gene_cluster (3% of genes show similarity) | BGC0001065_c1 |  |  |
| Cluster 21 | Siderophore | 7189328 | 7202508 | - | - |  |  |
| Cluster 22 | T2pks-T3pks | 7208586 | 7277065 | Spore_pigment_biosynthetic_gene_cluster (66% of genes show similarity) | BGC0000271_c1 |  |  |
| Cluster 23 | Terpene | 7293753 | 7314790 | - | - | 1 | CYP251F1 |
| ***Streptomyces* sp. CNQ-509** | | | | | | | |
| Cluster 1 | T1pks-Transatpks-Terpene | 114915 | 263143 | Tetronomycin_biosynthetic_gene_cluster (90% of genes show similarity) | BGC0000164_c1 | 1 | CYP105AC15 |
| Cluster 2 | Terpene | 506604 | 528829 | Laspartomycin_biosynthetic_gene_cluster (6% of genes show similarity) | BGC0000379_c1 |  |  |
| Cluster 3 | Nrps | 1031663 | 1082217 | Taromycin_biosynthetic_gene_cluster (6% of genes show similarity) | BGC0000439_c1 | 2 | CYP107LC1; CYP105BK2 |
| Cluster 4 | Ectoine | 1297153 | 1307551 | Ectoine_biosynthetic_gene_cluster (100% of genes show similarity) | BGC0000853_c1 |  |  |
| Cluster 5 | Terpene | 1840979 | 1861968 | - | - |  |  |
| Cluster 6 | Bacteriocin | 2037173 | 2047970 | Arginomycin_biosynthetic_gene_cluster (6% of genes show similarity) | BGC0000883_c1 |  |  |
| Cluster 7 | Siderophore | 2496813 | 2509146 | Desferrioxamine_B_biosynthetic_gene_cluster (80% of genes show similarity) | BGC0000941_c1 |  |  |
| Cluster 8 | Nrps | 2913716 | 2970785 | - | - |  |  |
| Cluster 9 | Other | 3012179 | 3056803 | Sporolide_biosynthetic_gene_cluster (6% of genes show similarity) | BGC0000150_c1 |  |  |
| Cluster 10 | Terpene | 3438020 | 3459228 | Fosfazinomycin_biosynthetic_gene_cluster (21% of genes show similarity) | BGC0000937_c1 |  |  |
| Cluster 11 | T2pks | 3511946 | 3554521 | Tetracenomycin_biosynthetic_gene_cluster (41% of genes show similarity) | BGC0000275_c1 | 1 | CYP1064A5 |
| Cluster 12 | Lantipeptide | 3683952 | 3708270 | - | - |  |  |
| Cluster 13 | Terpene | 4340279 | 4383600 | Pactamycin_biosynthetic_gene_cluster (3% of genes show similarity) | BGC0000119_c1 |  |  |
| Cluster 14 | Fused | 4512434 | 4536010 | - | - | 1 | CYP1341E2 |
| Cluster 15 | Thiopeptide | 4563155 | 4597839 | - | - |  |  |
| Cluster 16 | T3pks | 4639896 | 4680939 | Pactamycin_biosynthetic_gene_cluster (11% of genes show similarity) | BGC0000119_c1 | 2 | CYP157A20; CYP154C13 |
| Cluster 17 | T1pks | 4837274 | 4883564 | Laspartomycin_biosynthetic_gene_cluster (9% of genes show similarity) | BGC0000379_c1 |  |  |
| Cluster 18 | T3pks-Terpene-Otherks | 5581524 | 5655064 | Merochlorin_biosynthetic_gene_cluster (26% of genes show similarity) | BGC0001083_c1 | 1 | CYP123D1 |
| Cluster 19 | Terpene | 5674860 | 5699525 | Isorenieratene_biosynthetic_gene_cluster (85% of genes show similarity) | BGC0000664_c1 |  |  |
| Cluster 20 | Siderophore | 6179666 | 6194253 | - | - |  |  |
| Cluster 21 | T1pks-Nrps | 6797099 | 6869660 | Arsenopolyketides_biosynthetic_gene_cluster (37% of genes show similarity) | BGC0001283_c1 | 1 | CYP105BA2 |
| Cluster 22 | T2pks | 7072594 | 7145146 | Fluostatin_biosynthetic_gene_cluster (35% of genes show similarity) | BGC0000223_c1 |  |  |
| Cluster 23 | Phenazine | 7178028 | 7223783 | Marinophenazines_biosynthetic_gene_cluster (88% of genes show similarity) | BGC0001221_c1 |  |  |
| Cluster 24 | T3pks-Nrps | 7442803 | 7539290 | A47934_biosynthetic_gene_cluster (50% of genes show similarity) | BGC0000290_c1 | 2 | CYP165E2; CYP165B8 |
| Cluster 25 | Terpene | 7568186 | 7609590 | Hopene_biosynthetic_gene_cluster (61% of genes show similarity) | BGC0000663_c1 | 1 | CYP157C32 |
| Cluster 26 | T3pks | 7613012 | 7654100 | Alkylresorcinol_biosynthetic_gene_cluster (100% of genes show similarity) | BGC0000282_c1 |  |  |
| Cluster 27 | Nrps | 7676692 | 7724338 | Oxazolomycin_biosynthetic_gene_cluster (12% of genes show similarity) | BGC0001106_c1 |  |  |
| Cluster 28 | Siderophore | 7759755 | 7773368 | - | - |  |  |
| ***Streptomyces ambofaciens* ATCC 23877** | | | | | | | |
| Cluster 1 | T2pks-Butyrolactone | 142506 | 222698 | Fluostatin_biosynthetic_gene_cluster (29% of genes show similarity) | BGC0000223_c1 | 1 | CYP154K2 |
| Cluster 2 | T1pks-Terpene-Nrps | 340601 | 411878 | Antimycin_biosynthetic_gene_cluster (100% of genes show similarity) | BGC0000958_c1 |  |  |
| Cluster 3 | Indole | 706084 | 727211 | - | - | 1 | CYP156B15 |
| Cluster 4 | Terpene | 783854 | 815417 | Carotenoid_biosynthetic_gene_cluster (27% of genes show similarity) | BGC0000633_c1 |  |  |
| Cluster 5 | Ectoine | 2000972 | 2011370 | Ectoine_biosynthetic_gene_cluster (100% of genes show similarity) | BGC0000853_c1 |  |  |
| Cluster 6 | Melanin | 2873096 | 2883623 | Melanin_biosynthetic_gene_cluster (60% of genes show similarity) | BGC0000909_c1 |  |  |
| Cluster 7 | Siderophore | 2980232 | 2992004 | Desferrioxamine_B_biosynthetic_gene_cluster (100% of genes show similarity) | BGC0000940_c1 |  |  |
| Cluster 8 | Butyrolactone | 3468493 | 3478867 | - | - |  |  |
| Cluster 9 | Nrps | 4538916 | 4580316 | Zorbamycin_biosynthetic_gene_cluster (6% of genes show similarity) | BGC0001058_c1 |  |  |
| Cluster 10 | Terpene | 5302337 | 5323356 | Albaflavenone_biosynthetic_gene_cluster (100% of genes show similarity) | BGC0000660_c1 | 1 | CYP170A1 |
| Cluster 11 | T2pks | 5372707 | 5415255 | Spore_pigment_biosynthetic_gene_cluster (66% of genes show similarity) | BGC0000271_c1 |  |  |
| Cluster 12 | Siderophore | 5867408 | 5879165 | - | - |  |  |
| Cluster 13 | Oligosaccharide-T1pks-Nrps | 5942161 | 6143480 | Midecamycin_biosynthetic_gene_cluster (41% of genes show similarity) | BGC0000096_c1 | 2 | CYP107EP1; CYP113B4 |
| Cluster 14 | Bacteriocin | 6235154 | 6246572 | - | - |  |  |
| Cluster 15 | Terpene | 6259614 | 6281794 | Herboxidiene_biosynthetic_gene_cluster (2% of genes show similarity) | BGC0001065_c1 |  |  |
| Cluster 16 | Siderophore | 6452952 | 6466082 | Grincamycin_biosynthetic_gene_cluster (8% of genes show similarity) | BGC0000229_c1 |  |  |
| Cluster 17 | Terpene | 6901163 | 6927934 | Hopene_biosynthetic_gene_cluster (92% of genes show similarity) | BGC0000663_c1 |  |  |
| Cluster 18 | Nrps | 7171867 | 7240586 | Congocidine_biosynthetic_gene_cluster (100% of genes show similarity) | BGC0000327_c1 |  |  |
| Cluster 19 | Terpene | 7277128 | 7298159 | - | - | 1 | CYP157K5 |
| Cluster 20 | Bacteriocin | 7314194 | 7324409 | Informatipeptin_biosynthetic_gene_cluster (42% of genes show similarity) | BGC0000518_c1 |  |  |
| Cluster 21 | Nrps | 7565299 | 7616302 | Coelichelin_biosynthetic_gene_cluster (100% of genes show similarity) | BGC0000325_c1 |  |  |
| Cluster 22 | Terpene | 7629726 | 7650940 | Isorenieratene_biosynthetic_gene_cluster (57% of genes show similarity) | BGC0000664_c1 |  |  |
| Cluster 23 | T1pks | 7665382 | 7835031 | Stambomycin_biosynthetic_gene_cluster (100% of genes show similarity) | BGC0000151_c1 | 2 | CYP107EF1, CYP107EP1 |
| Cluster 24 | Lantipeptide-Terpene | 7921717 | 7968985 | Tetronasin_biosynthetic_gene_cluster (5% of genes show similarity) | BGC0000163_c1 |  |  |
| Cluster 25 | T1pks | 8007437 | 8054819 | Clavams_biosynthetic_gene_cluster (18% of genes show similarity) | BGC0000843_c1 |  |  |
| Cluster 26 | T2pks-Butyrolactone | 8081268 | 8161435 | Fluostatin_biosynthetic_gene_cluster (29% of genes show similarity) | BGC0000223_c1 | 1 | CYP154K2 |
| ***Streptomyces pristinaespiralis* HCCB 10218** | | | | | | | |
| Cluster 1 | Oligosaccharide-Ectoine-T2pks-Nrps-T1pks-Otherks | 136600 | 410171 | Pristinamycin_biosynthetic_gene_cluster (93% of genes show similarity) | BGC0000952_c3 | 3 | CYP154A22; CYP107EH1; CYP113C2 |
| Cluster 2 | T3pks | 668742 | 709860 | Lasalocid_biosynthetic_gene_cluster (11% of genes show similarity) | BGC0000087_c1 |  |  |
| Cluster 3 | Thiopeptide-Nrps | 1072428 | 1148005 | Albachelin_biosynthetic_gene_cluster (80% of genes show similarity) | BGC0001211_c1 |  |  |
| Cluster 4 | Terpene | 1167008 | 1193675 | Hopene_biosynthetic_gene_cluster (69% of genes show similarity) | BGC0000663_c1 |  |  |
| Cluster 5 | Lassopeptide | 1363490 | 1386215 | Kanamycin_biosynthetic_gene_cluster (1% of genes show similarity) | BGC0000703_c1 |  |  |
| Cluster 6 | Bacteriocin | 2055619 | 2066929 | - | - |  |  |
| Cluster 7 | Thiopeptide-T2pks | 2205468 | 2274572 | Spore_pigment_biosynthetic_gene_cluster (83% of genes show similarity) | BGC0000271_c1 |  |  |
| Cluster 8 | Terpene | 2311778 | 2337445 | Isorenieratene_biosynthetic_gene_cluster (85% of genes show similarity) | BGC0000664_c1 |  |  |
| Cluster 9 | Siderophore | 2467192 | 2482043 | - | - |  |  |
| Cluster 10 | Lantipeptide | 2837152 | 2859773 | SapB_biosynthetic_gene_cluster (100% of genes show similarity) | BGC0000551_c1 |  |  |
| Cluster 11 | Melanin | 3390526 | 3401059 | Melanin_biosynthetic_gene_cluster (100% of genes show similarity) | BGC0000911_c1 | 1 | CYP124G6 |
| Cluster 12 | T1pks-Otherks | 4034744 | 4089572 | R1128_biosynthetic_gene_cluster (21% of genes show similarity) | BGC0000261_c1 |  |  |
| Cluster 13 | Siderophore | 5528888 | 5540660 | Desferrioxamine_B_biosynthetic_gene_cluster (100% of genes show similarity) | BGC0000940_c1 |  |  |
| Cluster 14 | Terpene | 5662970 | 5684067 | - | - |  |  |
| Cluster 15 | Ectoine | 6550882 | 6606667 | Ectoine_biosynthetic_gene_cluster (100% of genes show similarity) | BGC0000853_c1 |  |  |
| Cluster 16 | Terpene | 7528164 | 7549282 | - | - |  |  |
| Cluster 17 | Bacteriocin | 7675340 | 7685558 | Sch47554_/_Sch47555_biosynthetic_gene_cluster (3% of genes show similarity) | BGC0000268_c1 |  |  |
| Cluster 18 | Terpene | 8034955 | 8057159 | - | - |  |  |
| Cluster 19 | T2pks-Oligosaccharide-Nrps-Otherks | 8110148 | 8245788 | Pristinamycin_biosynthetic_gene_cluster (97% of genes show similarity) | BGC0000952_c3 | 3 | CYP154B6; CYP113C2, CYP107EH1 |
| Cluster 20 | Ectoine-Nrps | 8233370 | 8340124 | Pristinamycin_biosynthetic_gene_cluster (100% of genes show similarity) | BGC0000952_c1 |  |  |
| Cluster 21 | T1pks-Ectoine-Otherks | 8335201 | 8395993 | Kosinostatin_biosynthetic_gene_cluster (9% of genes show similarity) | BGC0001073_c1 | 1 | CYP154A22 |
| ***Streptomyces* sp. CFMR 7** | | | | | | | |
| Cluster 1 | Thiopeptide-T1pks-Nrps | 58429 | 118641 | Daptomycin_biosynthetic_gene_cluster (7% of genes show similarity) | BGC0000336_c1 |  |  |
| Cluster 2 | Terpene | 123726 | 149324 | Isorenieratene_biosynthetic_gene_cluster (100% of genes show similarity) | BGC0000664_c1 |  |  |
| Cluster 3 | T3pks-Nrps | 258620 | 375779 | Herboxidiene_biosynthetic_gene_cluster (12% of genes show similarity) | BGC0001065_c1 |  |  |
| Cluster 4 | Melanin | 410995 | 421465 | Melanin_biosynthetic_gene_cluster (100% of genes show similarity) | BGC0000911_c1 | 1 | CYP124G2 |
| Cluster 5 | Nrps | 428038 | 486263 | Valinomycin_biosynthetic_gene_cluster (22% of genes show similarity) | BGC0000453_c1 |  |  |
| Cluster 6 | Nrps | 557485 | 624704 | Tallysomycin_biosynthetic_gene_cluster (7% of genes show similarity) | BGC0001048_c1 |  |  |
| Cluster 7 | Bacteriocin-T1pks-Nrps | 651520 | 718561 | SGR_PTMs_biosynthetic_gene_cluster (100% of genes show similarity) | BGC0001043_c1 | 1 | CYP107BX7 |
| Cluster 8 | T1pks | 755925 | 855340 | Bafilomycin_biosynthetic_gene_cluster (100% of genes show similarity) | BGC0000028_c1 |  |  |
| Cluster 9 | Nrps | 877606 | 932660 | A201A_biosynthetic_gene_cluster (8% of genes show similarity) | BGC0001138_c1 | 1 | CYP154A18 |
| Cluster 10 | Terpene | 955342 | 981915 | Hopene_biosynthetic_gene_cluster (69% of genes show similarity) | BGC0000663_c1 |  |  |
| Cluster 11 | Nrps | 1423338 | 1482428 | Asukamycin_biosynthetic_gene_cluster (11% of genes show similarity) | BGC0000187_c1 |  |  |
| Cluster 12 | Bacteriocin | 1611798 | 1623204 | - | - |  |  |
| Cluster 13 | Nrps | 1723980 | 1767063 | FD-594_biosynthetic_gene_cluster (6% of genes show similarity) | BGC0000222_c1 |  |  |
| Cluster 14 | Nrps | 1868317 | 1911696 | Marformycins_biosynthetic_gene_cluster (12% of genes show similarity) | BGC0001214_c1 |  |  |
| Cluster 15 | Siderophore | 2160199 | 2174961 | - | - |  |  |
| Cluster 16 | Amglyccycl | 2552447 | 2573640 | Neocarzilin_biosynthetic_gene_cluster (21% of genes show similarity) | BGC0000111_c1 |  |  |
| Cluster 17 | Terpene | 2584713 | 2605690 | - | - |  |  |
| Cluster 18 | Lantipeptide | 2947152 | 2969935 | AmfS_biosynthetic_gene_cluster (100% of genes show similarity) | BGC0000496_c1 |  |  |
| Cluster 19 | Lantipeptide | 3663589 | 3683780 | - | - |  |  |
| Cluster 20 | Lassopeptide | 3813538 | 3836280 | SRO15-2005_biosynthetic_gene_cluster (100% of genes show similarity) | BGC0000578_c1 |  |  |
| Cluster 21 | T2pks | 3890875 | 3933366 | Rabelomycin_biosynthetic_gene_cluster (25% of genes show similarity) | BGC0000262_c1 |  |  |
| Cluster 22 | Ectoine-Butyrolactone | 4682870 | 4698288 | Skyllamycin_biosynthetic_gene_cluster (8% of genes show similarity) | BGC0000429_c1 |  |  |
| Cluster 23 | Other | 4842524 | 4883618 | - | - |  |  |
| Cluster 24 | Nrps | 5023924 | 5090780 | Phosphonoglycans_biosynthetic_gene_cluster (3% of genes show similarity) | BGC0000806_c1 |  |  |
| Cluster 25 | Otherks | 5155943 | 5196989 | - | - |  |  |
| Cluster 26 | Thiopeptide | 5320375 | 5352933 | - | - |  |  |
| Cluster 27 | Other | 5591139 | 5635029 | Bottromycin_A2_biosynthetic_gene_cluster (36% of genes show similarity) | BGC0000469_c1 |  |  |
| Cluster 28 | Siderophore | 5714230 | 5726008 | Desferrioxamine_B_biosynthetic_gene_cluster (100% of genes show similarity) | BGC0000941_c1 |  |  |
| Cluster 29 | Lantipeptide | 5832437 | 5866769 | - | - |  |  |
| Cluster 30 | Ectoine | 6815095 | 6825493 | Ectoine_biosynthetic_gene_cluster (100% of genes show similarity) | BGC0000853_c1 |  |  |
| Cluster 31 | Terpene | 7283944 | 7305038 | Steffimycin_biosynthetic_gene_cluster (19% of genes show similarity) | BGC0000273_c1 |  |  |
| Cluster 32 | T3pks | 7787378 | 7828496 | Herboxidiene_biosynthetic_gene_cluster (6% of genes show similarity) | BGC0001065_c1 | 1 | CYP107F4 |
| Cluster 33 | Terpene | 7878228 | 7899235 | - | - |  |  |
| Cluster 34 | Melanin-Nrps | 7892226 | 7956790 | Coelichelin_biosynthetic_gene_cluster (81% of genes show similarity) | BGC0000325_c1 |  |  |
| Cluster 35 | Transatpks-T1pks-Otherks-Nrps | 7970068 | 8086862 | Griseobactin_biosynthetic_gene_cluster (94% of genes show similarity) | BGC0000368_c1 |  |  |
| Cluster 36 | Terpene | 8091617 | 8113830 | - | - |  |  |
| Cluster 37 | Butyrolactone | 8140824 | 8151774 | Coelimycin_biosynthetic_gene_cluster (16% of genes show similarity) | BGC0000038_c1 |  |  |
| Cluster 38 | Arylpolyene | 8160105 | 8203268 | A-500359s_biosynthetic_gene_cluster (5% of genes show similarity) | BGC0000949_c1 | 1 | CYP105D20 |
| ***Streptomyces* sp. CdTB01** | | | | | | | |
| Cluster 1 | T2pks-Nrps | 203006 | 302123 | Friulimicin_biosynthetic_gene_cluster (21% of genes show similarity) | BGC0000354_c1 |  |  |
| Cluster 2 | Other | 316900 | 357316 | Lomaiviticin_biosynthetic_gene_cluster (35% of genes show similarity) | BGC0000240_c1 |  |  |
| Cluster 3 | T3pks | 448018 | 489094 | Herboxidiene_biosynthetic_gene_cluster (3% of genes show similarity) | BGC0001065_c1 |  |  |
| Cluster 4 | Nrps | 600355 | 704555 | Azicemicin_biosynthetic_gene_cluster (11% of genes show similarity) | BGC0000202_c1 |  |  |
| Cluster 5 | Indole | 962291 | 983421 | - | - | 1 | CYP145C3 |
| Cluster 6 | Lassopeptide | 1077842 | 1100421 | Carbapenem_MM_4550_biosynthetic_gene_cluster (6% of genes show similarity) | BGC0000842_c1 |  |  |
| Cluster 7 | Bacteriocin-Lantipeptide | 1408106 | 1438334 | Informatipeptin_biosynthetic_gene_cluster (57% of genes show similarity) | BGC0000518_c1 |  |  |
| Cluster 8 | Terpene | 2019582 | 2046239 | Hopene_biosynthetic_gene_cluster (92% of genes show similarity) | BGC0000663_c1 |  |  |
| Cluster 9 | Siderophore | 2557703 | 2570808 | Grincamycin_biosynthetic_gene_cluster (8% of genes show similarity) | BGC0000229_c1 |  |  |
| Cluster 10 | Terpene | 2703518 | 2725674 | - | - | 1 | CYP180A29 |
| Cluster 11 | Bacteriocin | 2777155 | 2788468 | - | - |  |  |
| Cluster 12 | Bacteriocin | 2856792 | 2868627 | Lipopeptide_8D1-1_/_lipopeptide_8D1-2_biosynthetic_gene_clus... (4% of genes show similarity) | BGC0001370_c1 |  |  |
| Cluster 13 | Siderophore | 2978195 | 2990246 | - | - |  |  |
| Cluster 14 | Other | 3311789 | 3352478 | - | - |  |  |
| Cluster 15 | Other | 3415999 | 3456520 | - | - |  |  |
| Cluster 16 | Other | 3840003 | 3880713 | - | - |  |  |
| Cluster 17 | Terpene | 3987144 | 4008067 | Albaflavenone_biosynthetic_gene_cluster (100% of genes show similarity) | BGC0000660_c1 | 1 | CYP170A19 |
| Cluster 18 | T2pks | 4156435 | 4198941 | Spore_pigment_biosynthetic_gene_cluster (83% of genes show similarity) | BGC0000271_c1 |  |  |
| Cluster 19 | Butyrolactone | 4903660 | 4914622 | Gilvocarcin_biosynthetic_gene_cluster (7% of genes show similarity) | BGC0000226_c1 |  |  |
| Cluster 20 | Butyrolactone | 5451362 | 5462285 | Pactamycin_biosynthetic_gene_cluster (5% of genes show similarity) | BGC0000119_c1 |  |  |
| Cluster 21 | Bacteriocin-Lassopeptide-Nrps | 5563100 | 5662606 | Chaxapeptin_biosynthetic_gene_cluster (42% of genes show similarity) | BGC0001307_c1 |  |  |
| Cluster 22 | Siderophore | 6377594 | 6389363 | Desferrioxamine_B_biosynthetic_gene_cluster (100% of genes show similarity) | BGC0000940_c1 |  |  |
| Cluster 23 | Melanin | 6480146 | 6490643 | Melanin_biosynthetic_gene_cluster (100% of genes show similarity) | BGC0000909_c1 |  |  |
| Cluster 24 | Ectoine | 7604852 | 7615256 | Ectoine_biosynthetic_gene_cluster (100% of genes show similarity) | BGC0000853_c1 |  |  |
| Cluster 25 | Other | 7842280 | 7886146 | - | - |  |  |
| Cluster 26 | Terpene | 7938794 | 7959870 | Pradimicin_biosynthetic_gene_cluster (7% of genes show similarity) | BGC0000256_c1 | 2 | CYP157C39; CYP183B5 |
| Cluster 27 | T1pks | 8789168 | 8833130 | Maduropeptin_biosynthetic_gene_cluster (3% of genes show similarity) | BGC0001008_c1 |  |  |
| Cluster 28 | Melanin | 9121961 | 9132335 | Melanin_biosynthetic_gene_cluster (57% of genes show similarity) | BGC0000908_c1 |  |  |
| Cluster 29 | Terpene | 9309416 | 9335435 | Carotenoid_biosynthetic_gene_cluster (63% of genes show similarity) | BGC0000633_c1 |  |  |
| Cluster 30 | T3pks-T1pks-Nrps | 9576854 | 9677125 | Antimycin_biosynthetic_gene_cluster (100% of genes show similarity) | BGC0000958_c1 |  |  |
| Cluster 31 | Thiopeptide | 9678619 | 9723583 | ECO-02301_biosynthetic_gene_cluster (7% of genes show similarity) | BGC0000052_c1 |  |  |
| Cluster 32 | Butyrolactone | 9877671 | 9888660 | Merochlorin_biosynthetic_gene_cluster (4% of genes show similarity) | BGC0001083_c1 |  |  |
| ***Streptomyces reticuli*** | | | | | | | |
| Cluster 1 | T1pks | 58809 | 169393 | Naphthomycin_biosynthetic_gene_cluster (71% of genes show similarity) | BGC0000106_c1 | 1 | CYP166A3 |
| Cluster 2 | Bacteriocin-Nrps | 436777 | 524492 | Informatipeptin_biosynthetic_gene_cluster (57% of genes show similarity) | BGC0000518_c1 | 1 | CYP113K6 |
| Cluster 3 | Other | 782580 | 824268 | Tambromycin_biosynthetic_gene_cluster (14% of genes show similarity) | BGC0001368_c1 | 1 | CYP162B2 |
| Cluster 4 | Terpene | 956043 | 980632 | Carotenoid_biosynthetic_gene_cluster (54% of genes show similarity) | BGC0000633_c1 |  |  |
| Cluster 5 | Other | 996598 | 1037308 | Kanamycin_biosynthetic_gene_cluster (1% of genes show similarity) | BGC0000703_c1 | 2 | CYP121A5; CYP154U7 |
| Cluster 6 | Melanin | 1135647 | 1146024 | Melanin_biosynthetic_gene_cluster (57% of genes show similarity) | BGC0000908_c1 |  |  |
| Cluster 7 | Nrps-T1pks-Otherks | 1309947 | 1435677 | Sanglifehrin_A_biosynthetic_gene_cluster (43% of genes show similarity) | BGC0001042_c1 |  |  |
| Cluster 8 | T2pks-T1pks-Otherks | 1583764 | 1641835 | Hedamycin_biosynthetic_gene_cluster (31% of genes show similarity) | BGC0000233_c1 |  |  |
| Cluster 9 | T2pks-T3pks-Otherks | 1910507 | 1979532 | Herboxidiene_biosynthetic_gene_cluster (8% of genes show similarity) | BGC0001065_c1 | 1 | CYP158A14 |
| Cluster 10 | Ectoine-T1pks-Otherks | 2033920 | 2093478 | Ectoine_biosynthetic_gene_cluster (75% of genes show similarity) | BGC0000853_c1 |  |  |
| Cluster 11 | Lassopeptide | 2310021 | 2332519 | Chaxapeptin_biosynthetic_gene_cluster (42% of genes show similarity) | BGC0001307_c1 |  |  |
| Cluster 12 | Ectoine | 2761145 | 2771549 | Ectoine_biosynthetic_gene_cluster (100% of genes show similarity) | BGC0000853_c1 |  |  |
| Cluster 13 | T2pks | 3172003 | 3214516 | Spore_pigment_biosynthetic_gene_cluster (83% of genes show similarity) | BGC0000271_c1 |  |  |
| Cluster 14 | Nrps | 3313387 | 3364223 | Laspartomycin_biosynthetic_gene_cluster (6% of genes show similarity) | BGC0000379_c1 | 2 | CYP107LB1; CYP105BK2 |
| Cluster 15 | Melanin | 3838744 | 3849271 | Istamycin_biosynthetic_gene_cluster (5% of genes show similarity) | BGC0000700_c1 |  |  |
| Cluster 16 | Siderophore | 3940015 | 3951784 | Desferrioxamine_B_biosynthetic_gene_cluster (100% of genes show similarity) | BGC0000940_c1 |  |  |
| Cluster 17 | Butyrolactone-Amglyccycl-T1pks-Nrps | 5652600 | 5761564 | Pyralomicin_biosynthetic_gene_cluster (14% of genes show similarity) | BGC0001038_c1 | 1 | CYP1037A2 |
| Cluster 18 | Terpene | 6427916 | 6448839 | Albaflavenone_biosynthetic_gene_cluster (100% of genes show similarity) | BGC0000660_c1 | 1 | CYP170A23 |
| Cluster 19 | Siderophore | 7100383 | 7112428 | - | - |  |  |
| Cluster 20 | Nrps | 7197457 | 7312041 | Friulimicin_biosynthetic_gene_cluster (30% of genes show similarity) | BGC0000354_c1 | 1 | CYP2045A1 |
| Cluster 21 | Bacteriocin-Nrps | 7368052 | 7446646 | Meilingmycin_biosynthetic_gene_cluster (6% of genes show similarity) | BGC0000093_c1 |  |  |
| Cluster 22 | Bacteriocin | 7495248 | 7506561 | - | - |  |  |
| Cluster 23 | Terpene | 7535955 | 7558117 | - | - | 1 | CYP180A28 |
| Cluster 24 | Siderophore | 7728182 | 7741322 | - | - |  |  |
| Cluster 25 | Terpene | 8260062 | 8286833 | Hopene_biosynthetic_gene_cluster (92% of genes show similarity) | BGC0000663_c1 |  |  |
| Cluster 26 | Lassopeptide | 8302751 | 8325328 | Rifamycin_biosynthetic_gene_cluster (3% of genes show similarity) | BGC0000136_c1 |  |  |
| ***Streptomyces* sp. 4F** | | | | | | | |
| Cluster 1 | Lantipeptide | 141260 | 165398 | - | - |  |  |
| Cluster 2 | Nrps-T2pks-Otherks-T1pks-Phenazine | 266514 | 406568 | Antimycin_biosynthetic_gene_cluster (100% of genes show similarity) | BGC0000958_c1 | 1 | CYP105DD1 |
| Cluster 3 | Lantipeptide | 577739 | 609900 | - | - |  |  |
| Cluster 4 | T3pks | 656707 | 697780 | Alkylresorcinol_biosynthetic_gene_cluster (100% of genes show similarity) | BGC0000282_c1 |  |  |
| Cluster 5 | Terpene | 916010 | 940106 | Carotenoid_biosynthetic_gene_cluster (54% of genes show similarity) | BGC0000633_c1 |  |  |
| Cluster 6 | T2pks | 1143767 | 1186276 | Spore_pigment_biosynthetic_gene_cluster (83% of genes show similarity) | BGC0000271_c1 |  |  |
| Cluster 7 | Ectoine | 2042040 | 2052438 | Ectoine_biosynthetic_gene_cluster (100% of genes show similarity) | BGC0000853_c1 |  |  |
| Cluster 8 | Nrps | 2648339 | 2697721 | SCO-2138_biosynthetic_gene_cluster (64% of genes show similarity) | BGC0000595_c1 |  |  |
| Cluster 9 | Siderophore | 3056286 | 3068058 | Desferrioxamine_B_biosynthetic_gene_cluster (100% of genes show similarity) | BGC0000940_c1 |  |  |
| Cluster 10 | Terpene | 5286843 | 5307928 | Albaflavenone_biosynthetic_gene_cluster (100% of genes show similarity) | BGC0000660_c1 | 1 | CYP170A16 |
| Cluster 11 | Nrps | 5920868 | 6005627 | Friulimicin_biosynthetic_gene_cluster (18% of genes show similarity) | BGC0000354_c1 |  |  |
| Cluster 12 | Bacteriocin | 6205199 | 6216509 | - | - |  |  |
| Cluster 13 | Terpene | 6254217 | 6276373 | Herboxidiene_biosynthetic_gene_cluster (3% of genes show similarity) | BGC0001065_c1 |  |  |
| Cluster 14 | T1pks-Nrps | 6330111 | 6379887 | Althiomycin_biosynthetic_gene_cluster (66% of genes show similarity) | BGC0000955_c1 |  |  |
| Cluster 15 | Siderophore | 6456557 | 6469796 | Grincamycin_biosynthetic_gene_cluster (11% of genes show similarity) | BGC0000229_c1 |  |  |
| Cluster 16 | Terpene | 6897309 | 6924002 | Hopene_biosynthetic_gene_cluster (92% of genes show similarity) | BGC0000663_c1 |  |  |
| Cluster 17 | Bacteriocin | 7347393 | 7357608 | Informatipeptin_biosynthetic_gene_cluster (57% of genes show similarity) | BGC0000518_c1 |  |  |
| Cluster 18 | T2pks-Otherks | 7710378 | 7781259 | Hedamycin_biosynthetic_gene_cluster (34% of genes show similarity) | BGC0000233_c1 | 1 | CYP105DD1 |
| Cluster 19 | Lantipeptide | 7882374 | 7906512 | - | - |  |  |
| ***Streptomyces leeuwenhoekii* C34(2013)** | | | | | | | |
| Cluster 1 | T1pks | 99264 | 143430 | A54145_biosynthetic_gene_cluster (3% of genes show similarity) | BGC0000291_c1 |  |  |
| Cluster 2 | T1pks | 191701 | 240196 | Cremimycin_biosynthetic_gene_cluster (15% of genes show similarity) | BGC0000042_c1 |  |  |
| Cluster 3 | T3pks-T1pks-Nrps | 324784 | 458084 | Pristinamycin_biosynthetic_gene_cluster (12% of genes show similarity) | BGC0000952_c3 | 3 | CYP107L35; CYP105BT1; CYP154B4 |
| Cluster 4 | Bacteriocin | 572464 | 582679 | Informatipeptin_biosynthetic_gene_cluster (42% of genes show similarity) | BGC0000518_c1 |  |  |
| Cluster 5 | Terpene | 598795 | 619823 | - | - | 1 | CYP157K4 |
| Cluster 6 | Lassopeptide | 642180 | 664830 | Labyrinthopeptin_A1,A3_/_labyrinthopeptin_A2_biosynthetic_gene (40% of genes show similarity) | BGC0000519_c1 | 1 | CYP102B20 |
| Cluster 7 | Nrps | 714060 | 794426 | Rishirilide_B_biosynthetic_gene_cluster (7% of genes show similarity) | BGC0001179_c1 | 1 | CYP113K3 |
| Cluster 8 | Nrps-Transatpks-Terpene-Otherks | 1046004 | 1155931 | Leinamycin_biosynthetic_gene_cluster (18% of genes show similarity) | BGC0001101_c1 | 2 | CYP107EG1; CYP105BR1 |
| Cluster 9 | Terpene-T1pks | 1211564 | 1331006 | Chaxamycin_A_/_chaxamycin_B_/_chaxamycin_C_/_chaxamycin_D (96% of genes show similarity) | BGC0001287_c1 | 3 | CYP107Q3; CYP105D28; CYP166A2 |
| Cluster 10 | T1pks | 1497127 | 1544539 | - | - | 3 | CYP154Z1; CYP1416A1; CYP2266A2 |
| Cluster 11 | Terpene | 1624097 | 1645110 | Pentalenolactone_biosynthetic_gene_cluster (58% of genes show similarity) | BGC0000653_c1 | 2 | CYP183A4; CYP161C4 |
| Cluster 12 | T1pks-Siderophore | 1776281 | 1833813 | Grincamycin_biosynthetic_gene_cluster (8% of genes show similarity) | BGC0000229_c1 | 2 | CYP107L33; CYP105AC8 |
| Cluster 13 | Terpene | 1972277 | 1994487 | Herboxidiene_biosynthetic_gene_cluster (2% of genes show similarity) | BGC0001065_c1 |  |  |
| Cluster 14 | Bacteriocin | 2013690 | 2025087 | - | - |  |  |
| Cluster 15 | Siderophore | 2293580 | 2305424 | - | - |  |  |
| Cluster 16 | T1pks-Nrps | 2668194 | 2719415 | BD-12_biosynthetic_gene_cluster (17% of genes show similarity) | BGC0001379_c1 |  |  |
| Cluster 17 | T3pks | 2937137 | 2978264 | Herboxidiene_biosynthetic_gene_cluster (3% of genes show similarity) | BGC0001065_c1 | 1 | CYP107F10 |
| Cluster 18 | Terpene | 3047731 | 3068819 | Albaflavenone_biosynthetic_gene_cluster (100% of genes show similarity) | BGC0000660_c1 | 1 | CYP170A17 |
| Cluster 19 | Lassopeptide | 3552136 | 3574705 | TP-1161_biosynthetic_gene_cluster (20% of genes show similarity) | BGC0000615_c1 |  |  |
| Cluster 20 | Bacteriocin | 3683199 | 3693399 | Arginomycin_biosynthetic_gene_cluster (10% of genes show similarity) | BGC0000883_c1 |  |  |
| Cluster 21 | Siderophore | 5232176 | 5243945 | Desferrioxamine_B_biosynthetic_gene_cluster (100% of genes show similarity) | BGC0000940_c1 |  |  |
| Cluster 22 | Melanin | 5330379 | 5340933 | Melanin_biosynthetic_gene_cluster (100% of genes show similarity) | BGC0000910_c1 |  |  |
| Cluster 23 | Butyrolactone-Amglyccycl | 5385171 | 5417416 | Neocarzilin_biosynthetic_gene_cluster (21% of genes show similarity) | BGC0000111_c1 |  |  |
| Cluster 24 | Ectoine | 6176293 | 6186691 | Ectoine_biosynthetic_gene_cluster (100% of genes show similarity) | BGC0000853_c1 |  |  |
| Cluster 25 | Other | 6710095 | 6751819 | Kirromycin_biosynthetic_gene_cluster (3% of genes show similarity) | BGC0001070_c1 |  |  |
| Cluster 26 | T3pks | 6822979 | 6864043 | BE-14106_biosynthetic_gene_cluster (17% of genes show similarity) | BGC0000029_c1 |  |  |
| Cluster 27 | T1pks | 7141058 | 7240871 | Brasilinolide_biosynthetic_gene_cluster (4% of genes show similarity) | BGC0001381_c1 | 1 | CYP1418A1 |
| Cluster 28 | T1pks | 7355977 | 7439461 | JBIR-100_biosynthetic_gene_cluster (66% of genes show similarity) | BGC0001348_c1 | 1 | CYP1031A3 |
| Cluster 29 | Other | 7486047 | 7529121 | Herbimycin_biosynthetic_gene_cluster (10% of genes show similarity) | BGC0000074_c1 | 1 | CYP107AM11 |
| Cluster 30 | T2pks-Terpene | 7530162 | 7588405 | Spore_pigment_biosynthetic_gene_cluster (83% of genes show similarity) | BGC0000271_c1 |  |  |
| Cluster 31 | Terpene | 7744176 | 7768730 | Carotenoid_biosynthetic_gene_cluster (54% of genes show similarity) | BGC0000633_c1 |  |  |
| ***Streptomyces rubrolavendulae*** | | | | | | | |
| Cluster 1 | Terpene | 137572 | 159833 | - | - |  |  |
| Cluster 2 | Terpene | 301875 | 322972 | - | - | 3 | CYP251A3; CYP157C21; CYP158A20 |
| Cluster 3 | Other | 488022 | 530907 | Desotamide_biosynthetic_gene_cluster (9% of genes show similarity) | BGC0001196_c1 |  |  |
| Cluster 4 | Lantipeptide | 608457 | 633367 | Akaeolide_biosynthetic_gene_cluster (12% of genes show similarity) | BGC0001199_c1 |  |  |
| Cluster 5 | Indole | 654870 | 678301 | Staurosporine_biosynthetic_gene_cluster (60% of genes show similarity) | BGC0000825_c1 | 2 | CYP245A6; CYP244A3 |
| Cluster 6 | Lassopeptide | 1093159 | 1115588 | - | - |  |  |
| Cluster 7 | Ectoine | 1271932 | 1282330 | Ectoine_biosynthetic_gene_cluster (100% of genes show similarity) | BGC0000853_c1 |  |  |
| Cluster 8 | Siderophore | 2154239 | 2166161 | Desferrioxamine_B_biosynthetic_gene_cluster (100% of genes show similarity) | BGC0000940_c1 |  |  |
| Cluster 9 | Melanin | 3552050 | 3562661 | Istamycin_biosynthetic_gene_cluster (11% of genes show similarity) | BGC0000700_c1 |  |  |
| Cluster 10 | Bacteriocin | 3689684 | 3700145 | - | - |  |  |
| Cluster 11 | Nrps | 5063839 | 5170579 | Viomycin_biosynthetic_gene_cluster (95% of genes show similarity) | BGC0000458_c1 | 2 | CYP159A23; CYP157B32 |
| Cluster 12 | T1pks | 5199891 | 5265457 | Bleomycin_biosynthetic_gene_cluster (9% of genes show similarity) | BGC0000963_c1 |  |  |
| Cluster 13 | Thiopeptide | 5298877 | 5374556 | Cyclooctatin_biosynthetic_gene_cluster (100% of genes show similarity) | BGC0000677_c1 | 2 | CYP183H3; CYP183G3 |
| Cluster 14 | Bacteriocin | 5488275 | 5499714 | - | - |  |  |
| Cluster 15 | Other | 5563360 | 5607193 | Auricin_deoxysugar_moieties_biosynthetic_gene_cluster (24% of genes show similarity) | BGC0000727_c1 |  |  |
| Cluster 16 | Siderophore | 5623819 | 5639364 | Macrotetrolide_biosynthetic_gene_cluster (33% of genes show similarity) | BGC0000244_c1 |  |  |
| Cluster 17 | Nrps | 5676095 | 5774369 | Kanamycin_biosynthetic_gene_cluster (2% of genes show similarity) | BGC0000703_c1 |  |  |
| Cluster 18 | Terpene | 5934523 | 5961241 | Hopene_biosynthetic_gene_cluster (69% of genes show similarity) | BGC0000663_c1 |  |  |
| Cluster 19 | Terpene | 5978656 | 6004547 | Isorenieratene_biosynthetic_gene_cluster (100% of genes show similarity) | BGC0000664_c1 |  |  |
| Cluster 20 | Amglyccycl | 6026409 | 6060840 | Neomycin_biosynthetic_gene_cluster (63% of genes show similarity) | BGC0000709_c1 |  |  |
| Cluster 21 | Terpene | 6213351 | 6234451 | Herbimycin_biosynthetic_gene_cluster (6% of genes show similarity) | BGC0000074_c1 | 1 | CYP251F2 |
| Cluster 22 | Lantipeptide-Nrps | 6258519 | 6316171 | - | - | 1 | CYP1207A10 |
| Cluster 23 | T3pks-Butyrolactone | 6387947 | 6471130 | Lomofungin_biosynthetic_gene_cluster (26% of genes show similarity) | BGC0001302_c1 | 1 | CYP158A19 |
| Cluster 24 | Nrps | 6479692 | 6532273 | Daptomycin_biosynthetic_gene_cluster (7% of genes show similarity) | BGC0000336_c1 | 2 | CYP107E12; CYP285D1 |
| ***Streptomyces parvulus*** | | | | | | | |
| Cluster 1 | Terpene | 90078 | 111136 | 2-methylisoborneol_biosynthetic_gene_cluster (100% of genes show similarity) | BGC0000658_c1 |  |  |
| Cluster 2 | Terpene | 132842 | 201970 | Isorenieratene_biosynthetic_gene_cluster (100% of genes show similarity) | BGC0000664_c1 |  |  |
| Cluster 3 | Indole | 313179 | 334306 | Ravidomycin_biosynthetic_gene_cluster (5% of genes show similarity) | BGC0000263_c1 | 2 | CYP156B15; CYP183J3 |
| Cluster 4 | Nrps | 556539 | 624553 | Actinomycin_biosynthetic_gene_cluster (82% of genes show similarity) | BGC0000296_c1 |  |  |
| Cluster 5 | Ectoine | 1603590 | 1613988 | Ectoine_biosynthetic_gene_cluster (100% of genes show similarity) | BGC0000853_c1 |  |  |
| Cluster 6 | Melanin | 2492824 | 2503330 | Melanin_biosynthetic_gene_cluster (60% of genes show similarity) | BGC0000909_c1 |  |  |
| Cluster 7 | Siderophore | 2584513 | 2596282 | Desferrioxamine_B_biosynthetic_gene_cluster (100% of genes show similarity) | BGC0000940_c1 |  |  |
| Cluster 8 | Nrps | 4119934 | 4178843 | Borrelidin_biosynthetic_gene_cluster (4% of genes show similarity) | BGC0000031_c1 |  |  |
| Cluster 9 | Terpene | 4829070 | 4850155 | Albaflavenone_biosynthetic_gene_cluster (100% of genes show similarity) | BGC0000660_c1 | 1 | CYP170A1 |
| Cluster 10 | T2pks | 4896168 | 4938716 | Spore_pigment_biosynthetic_gene_cluster (66% of genes show similarity) | BGC0000271_c1 |  |  |
| Cluster 11 | Other | 5248686 | 5289795 | Calcium-dependent_antibiotic_biosynthetic_gene_cluster (10% of genes show similarity) | BGC0000315_c1 |  |  |
| Cluster 12 | Siderophore | 5414789 | 5426603 | - | - |  |  |
| Cluster 13 | Bacteriocin | 5626803 | 5638137 | - | - |  |  |
| Cluster 14 | Terpene | 5655570 | 5677768 | Herboxidiene_biosynthetic_gene_cluster (2% of genes show similarity) | BGC0001065_c1 |  |  |
| Cluster 15 | Siderophore | 5827742 | 5840921 | Grincamycin_biosynthetic_gene_cluster (8% of genes show similarity) | BGC0000229_c1 |  |  |
| Cluster 16 | Lantipeptide | 6160164 | 6182773 | SAL-2242_biosynthetic_gene_cluster (100% of genes show similarity) | BGC0000546_c1 |  |  |
| Cluster 17 | Terpene | 6248979 | 6275723 | Hopene_biosynthetic_gene_cluster (92% of genes show similarity) | BGC0000663_c1 |  |  |
| Cluster 18 | Terpene | 6617669 | 6638700 | Versipelostatin_biosynthetic_gene_cluster (5% of genes show similarity) | BGC0001204_c1 | 2 | CYP157K6; CYP105D33 |
| Cluster 19 | Bacteriocin | 6649380 | 6659595 | Informatipeptin_biosynthetic_gene_cluster (42% of genes show similarity) | BGC0000518_c1 |  |  |
| Cluster 20 | Nrps | 6874609 | 6925528 | Coelichelin_biosynthetic_gene_cluster (90% of genes show similarity) | BGC0000325_c1 |  |  |
| Cluster 21 | T3pks | 6973951 | 7015135 | Kanamycin_biosynthetic_gene_cluster (1% of genes show similarity) | BGC0000703_c1 |  |  |
| ***Streptomyces lydicus* 103** | | | | | | | |
| Cluster 1 | Lantipeptide | 140498 | 176168 | Svaricin_biosynthetic_gene_cluster (12% of genes show similarity) | BGC0001382_c1 |  |  |
| Cluster 2 | T1pks | 632618 | 678779 | Laspartomycin_biosynthetic_gene_cluster (4% of genes show similarity) | BGC0000379_c1 | 1 | CYP147F26 |
| Cluster 3 | T1pks-Nrps | 679298 | 735222 | Antalid_biosynthetic_gene_cluster (18% of genes show similarity) | BGC0001235_c1 |  |  |
| Cluster 4 | Lassopeptide | 1043432 | 1066034 | - | - |  |  |
| Cluster 5 | Ectoine | 2288692 | 2299108 | Ectoine_biosynthetic_gene_cluster (100% of genes show similarity) | BGC0000853_c1 |  |  |
| Cluster 6 | Siderophore | 2381075 | 2392883 | Desferrioxamine_B_biosynthetic_gene_cluster (80% of genes show similarity) | BGC0000941_c1 |  |  |
| Cluster 7 | Terpene-T1pks-Nrps | 2855513 | 2966648 | Streptolydigin_biosynthetic_gene_cluster (97% of genes show similarity) | BGC0001046_c1 | 2 | CYP107FH2; CYP107B26 |
| Cluster 8 | T2pks | 3269571 | 3312086 | Spore_pigment_biosynthetic_gene_cluster (83% of genes show similarity) | BGC0000271_c1 |  |  |
| Cluster 9 | Bacteriocin | 3367107 | 3377340 | - | - |  |  |
| Cluster 10 | Lantipeptide | 3656937 | 3679549 | SapB_biosynthetic_gene_cluster (100% of genes show similarity) | BGC0000551_c1 |  |  |
| Cluster 11 | T1pks-Nrps | 3761430 | 3831019 | SW-163_biosynthetic_gene_cluster (10% of genes show similarity) | BGC0000434_c1 | 1 | CYP163G1 |
| Cluster 12 | Thiopeptide | 3985879 | 4047327 | Cyclothiazomycin_biosynthetic_gene_cluster (38% of genes show similarity) | BGC0000603_c1 | 1 | CYP147F27 |
| Cluster 13 | Terpene-Nrps | 4476045 | 4560849 | Isorenieratene_biosynthetic_gene_cluster (100% of genes show similarity) | BGC0000664_c1 |  |  |
| Cluster 14 | Bacteriocin | 4574348 | 4586282 | - | - |  |  |
| Cluster 15 | Butyrolactone | 4723978 | 4734838 | Hygrocin_biosynthetic_gene_cluster (6% of genes show similarity) | BGC0000075_c1 | 1 | CYP107AE12 |
| Cluster 16 | Melanin-Nrps | 4933655 | 4994238 | WS9326_biosynthetic_gene_cluster (10% of genes show similarity) | BGC0001297_c1 | 1 | CYP107FV4 |
| Cluster 17 | Other | 5056238 | 5097611 | A-503083_biosynthetic_gene_cluster (7% of genes show similarity) | BGC0000288_c1 | 1 | CYP1005B7 |
| Cluster 18 | Nucleoside-Lassopeptide-Nrps | 5141027 | 5208382 | Rapamycin_biosynthetic_gene_cluster (14% of genes show similarity) | BGC0001040_c1 | 1 | CYP157C36 |
| Cluster 19 | Terpene | 5409855 | 5436452 | Hopene_biosynthetic_gene_cluster (69% of genes show similarity) | BGC0000663_c1 |  |  |
| Cluster 20 | Terpene | 5579689 | 5600747 | Kanamycin_biosynthetic_gene_cluster (53% of genes show similarity) | BGC0000706_c1 |  |  |
| Cluster 21 | Bacteriocin | 5993231 | 6004689 | - | - |  |  |
| Cluster 22 | Butyrolactone | 6060071 | 6071060 | Griseoviridin_/_viridogrisein_biosynthetic_gene_cluster (5% of genes show similarity) | BGC0000459_c1 |  |  |
| Cluster 23 | T3pks-Nrps | 6090851 | 6154344 | Feglymycin_biosynthetic_gene_cluster (36% of genes show similarity) | BGC0001233_c1 | 1 | CYP107F15 |
| Cluster 24 | Siderophore | 6256819 | 6271591 | - | - |  |  |
| Cluster 25 | Nrps | 7791714 | 7850912 | Mannopeptimycin_biosynthetic_gene_cluster (81% of genes show similarity) | BGC0000388_c1 |  |  |
| Cluster 26 | Terpene | 7923764 | 7946001 | Salinomycin_biosynthetic_gene_cluster (6% of genes show similarity) | BGC0000144_c1 |  |  |
| ***Streptomyces* sp. SAT1** | | | | | | | |
| Cluster 1 | Lantipeptide | 1 | 24200 | Herboxidiene_biosynthetic_gene_cluster (7% of genes show similarity) | BGC0001065_c1 |  |  |
| Cluster 2 | Phosphonate-Nrps | 74302 | 148960 | Scabichelin_biosynthetic_gene_cluster (30% of genes show similarity) | BGC0000423_c1 | 1 | CYP1618A1 |
| Cluster 3 | Thiopeptide-Terpene | 215878 | 265579 | Isorenieratene_biosynthetic_gene_cluster (85% of genes show similarity) | BGC0000664_c1 | 1 | CYP180B6 |
| Cluster 4 | Butyrolactone | 285579 | 296460 | U-68204_biosynthetic_gene_cluster (14% of genes show similarity) | BGC0001354_c1 | 1 | CYP105B73 |
| Cluster 5 | T1pks | 354232 | 403174 | Herboxidiene_biosynthetic_gene_cluster (2% of genes show similarity) | BGC0001065_c1 |  |  |
| Cluster 6 | Other | 708904 | 752167 | Stenothricin_biosynthetic_gene_cluster (13% of genes show similarity) | BGC0000431_c1 |  |  |
| Cluster 7 | Terpene | 868664 | 889695 | Oxazolomycin_biosynthetic_gene_cluster (9% of genes show similarity) | BGC0001106_c1 | 1 | CYP183X4 |
| Cluster 8 | T3pks | 922781 | 963839 | Herboxidiene_biosynthetic_gene_cluster (7% of genes show similarity) | BGC0001065_c1 | 1 | CYP158A25 |
| Cluster 9 | Ectoine | 1573240 | 1583644 | Ectoine_biosynthetic_gene_cluster (100% of genes show similarity) | BGC0000853_c1 |  |  |
| Cluster 10 | Melanin | 2467228 | 2477665 | Melanin_biosynthetic_gene_cluster (60% of genes show similarity) | BGC0000909_c1 |  |  |
| Cluster 11 | T2pks | 3223020 | 3265538 | Spore_pigment_biosynthetic_gene_cluster (83% of genes show similarity) | BGC0000271_c1 |  |  |
| Cluster 12 | Other | 3883484 | 3925397 | Salinosporamide_biosynthetic_gene_cluster (16% of genes show similarity) | BGC0001041_c1 | 1 | CYP107P31 |
| Cluster 13 | Terpene | 4852785 | 4873798 | Albaflavenone_biosynthetic_gene_cluster (100% of genes show similarity) | BGC0000660_c1 | 1 | CYP170A18 |
| Cluster 14 | Siderophore | 5500156 | 5512363 | - | - |  |  |
| Cluster 15 | Nrps-T1pks-Otherks | 5556590 | 5645890 | Nataxazole_biosynthetic_gene_cluster (77% of genes show similarity) | BGC0001213_c1 |  |  |
| Cluster 16 | Bacteriocin | 5864472 | 5875941 | - | - |  |  |
| Cluster 17 | Terpene | 5883559 | 5905742 | - | - |  |  |
| Cluster 18 | Siderophore | 6064359 | 6077546 | Grincamycin_biosynthetic_gene_cluster (5% of genes show similarity) | BGC0000229_c1 |  |  |
| Cluster 19 | Terpene | 6367454 | 6394225 | Hopene_biosynthetic_gene_cluster (92% of genes show similarity) | BGC0000663_c1 |  |  |
| Cluster 20 | Bacteriocin-T1pks | 6551532 | 6664649 | Bafilomycin_biosynthetic_gene_cluster (94% of genes show similarity) | BGC0000028_c1 | 1 | CYP158A24 |
| Cluster 21 | Nrps | 6783735 | 6830197 | Griseobactin_biosynthetic_gene_cluster (47% of genes show similarity) | BGC0000368_c1 |  |  |
| Cluster 22 | Lantipeptide | 6903694 | 6926321 | Informatipeptin_biosynthetic_gene_cluster (57% of genes show similarity) | BGC0000518_c1 |  |  |
| Cluster 23 | Phosphoglycolipid | 7099434 | 7124720 | Teichomycin_biosynthetic_gene_cluster (66% of genes show similarity) | BGC0001395_c1 | 1 | CYP107E32 |
| Cluster 24 | Lantipeptide | 7146616 | 7187284 | - | - |  |  |
| Cluster 25 | Other | 7191528 | 7243622 | - | - |  |  |
| Cluster 26 | Bacteriocin-Terpene-Nrps | 7252610 | 7427729 | Coelichelin_biosynthetic_gene_cluster (100% of genes show similarity) | BGC0000325_c1 | 2 | CYP1722A3; CYP1618A2 |
| Cluster 27 | Terpene | 7437496 | 7458770 | 2-methylisoborneol_biosynthetic_gene_cluster (100% of genes show similarity) | BGC0000658_c1 |  |  |
| ***Streptomyces clavuligerus*** | | | | | | | |
| Cluster 1 | Siderophore | 10562 | 24130 | - | - |  |  |
| Cluster 2 | Terpene-T1pks-Nrps | 76329 | 184101 | Herboxidiene_biosynthetic_gene_cluster (8% of genes show similarity) | BGC0001065_c1 | 3 | CYP105BG1; CYP163B7; CYP251E1 |
| Cluster 3 | T3pks | 276819 | 317874 | Naringenin_biosynthetic_gene_cluster (100% of genes show similarity) | BGC0001310_c1 | 1 | CYP107F7 |
| Cluster 4 | Indole-Terpene-Nrps | 531915 | 645805 | Nucleocidin_biosynthetic_gene_cluster (47% of genes show similarity) | BGC0001387_c1 | 2 | CYP107NSF1; CYP107BY2 |
| Cluster 5 | Nrps | 657794 | 707790 | Holomycin_biosynthetic_gene_cluster (100% of genes show similarity) | BGC0000373_c1 |  |  |
| Cluster 6 | Terpene | 715154 | 741936 | Hopene_biosynthetic_gene_cluster (69% of genes show similarity) | BGC0000663_c1 |  |  |
| Cluster 7 | Nrps | 821138 | 866647 | Calcium-dependent_antibiotic_biosynthetic_gene_cluster (5% of genes show similarity) | BGC0000315_c1 |  |  |
| Cluster 8 | Lantipeptide | 1047480 | 1085758 | AmfS_biosynthetic_gene_cluster (80% of genes show similarity) | BGC0000496_c1 |  |  |
| Cluster 9 | T2pks | 1098934 | 1141500 | Spore_pigment_biosynthetic_gene_cluster (75% of genes show similarity) | BGC0000271_c1 |  |  |
| Cluster 10 | Bacteriocin | 1178193 | 1189566 | - | - |  |  |
| Cluster 11 | Nrps | 1300723 | 1379478 | A201A_biosynthetic_gene_cluster (15% of genes show similarity) | BGC0000873_c1 |  |  |
| Cluster 12 | Siderophore | 1428060 | 1440111 | - | - |  |  |
| Cluster 13 | T1pks-Nrps | 1680579 | 1735743 | Kanamycin_biosynthetic_gene_cluster (1% of genes show similarity) | BGC0000703_c1 | 1 | CYP136E1 |
| Cluster 14 | Lantipeptide | 1791838 | 1816396 | - | - |  |  |
| Cluster 15 | Nucleoside | 1918425 | 1939396 | Tunicamycin_biosynthetic_gene_cluster (92% of genes show similarity) | BGC0000880_c1 |  |  |
| Cluster 16 | Blactam-Nrps | 1997030 | 2057025 | Clavulanic_acid_biosynthetic_gene_cluster (75% of genes show similarity) | BGC0000845_c1 | 1 | CYP105M1 |
| Cluster 17 | Melanin | 2367325 | 2377759 | Melanin_biosynthetic_gene_cluster (100% of genes show similarity) | BGC0000911_c1 | 1 | CYP124G5 |
| Cluster 18 | Blactam | 3437138 | 3458112 | Clavams_biosynthetic_gene_cluster (81% of genes show similarity) | BGC0000843_c1 |  |  |
| Cluster 19 | Lantipeptide | 3971117 | 3995628 | - | - |  |  |
| Cluster 20 | Butyrolactone | 4147482 | 4158450 | Lactonamycin_biosynthetic_gene_cluster (3% of genes show similarity) | BGC0000238_c1 |  |  |
| Cluster 21 | Nrps | 4164716 | 4212044 | - | - |  |  |
| Cluster 22 | Siderophore | 4567319 | 4579139 | Desferrioxamine_B_biosynthetic_gene_cluster (100% of genes show similarity) | BGC0000941_c1 |  |  |
| Cluster 23 | Ectoine | 5558823 | 5569248 | Ectoine_biosynthetic_gene_cluster (100% of genes show similarity) | BGC0000853_c1 |  |  |
| Cluster 24 | T1pks-Butyrolactone-Otherks | 6201299 | 6288439 | Daptomycin_biosynthetic_gene_cluster (12% of genes show similarity) | BGC0000336_c1 | 1 | CYP107AL2 |
| Cluster 25 | Terpene | 6639864 | 6662038 | - | - |  |  |
| Cluster 26 | T1pks | 6788964 | 6883702 | Bafilomycin_biosynthetic_gene_cluster (66% of genes show similarity) | BGC0000028_c1 | 1 | CYP154A14 |
| ***Streptomyces griseochromogenes*** | | | | | | | |
| Cluster 1 | T2pks-Butyrolactone | 122962 | 169387 | Skyllamycin_biosynthetic_gene_cluster (6% of genes show similarity) | BGC0000429_c1 |  |  |
| Cluster 2 | Thiopeptide-Bacteriocin | 257601 | 305335 | GE37468_biosynthetic_gene_cluster (100% of genes show similarity) | BGC0000605_c1 | 1 | CYP113V2 |
| Cluster 3 | Phosphonate-Bacteriocin | 525563 | 578114 | Thioviridamide_biosynthetic_gene_cluster (21% of genes show similarity) | BGC0000625_c1 |  |  |
| Cluster 4 | Terpene | 1454562 | 1475575 | Albaflavenone_biosynthetic_gene_cluster (100% of genes show similarity) | BGC0000660_c1 | 1 | CYP170A10 |
| Cluster 5 | T1pks | 1534283 | 1580330 | Tiancimycin_biosynthetic_gene_cluster (16% of genes show similarity) | BGC0001378_c1 |  |  |
| Cluster 6 | Nrps-T1pks-Otherks | 1759065 | 1815292 | Caerulomycin_A_biosynthetic_gene_cluster (20% of genes show similarity) | BGC0000966_c1 |  |  |
| Cluster 7 | Siderophore-T1pks-Nrps | 2196713 | 2380559 | Apoptolidin_biosynthetic_gene_cluster (30% of genes show similarity) | BGC0000021_c1 |  |  |
| Cluster 8 | T1pks-Nrps | 2485653 | 2611697 | Polyoxypeptin_biosynthetic_gene_cluster (51% of genes show similarity) | BGC0001036_c1 | 1 | CYP247A3_ortholog |
| Cluster 9 | Bacteriocin | 2620971 | 2632827 | Lipopeptide_8D1-1_/_lipopeptide_8D1-2_biosynthetic_gene_clus... (4% of genes show similarity) | BGC0001370_c1 |  |  |
| Cluster 10 | T1pks | 2638274 | 2691239 | Meilingmycin_biosynthetic_gene_cluster (3% of genes show similarity) | BGC0000093_c1 | 1 | CYP105BC2 |
| Cluster 11 | Lassopeptide | 2696145 | 2718685 | Nanchangmycin_biosynthetic_gene_cluster (9% of genes show similarity) | BGC0000105_c1 |  |  |
| Cluster 12 | Bacteriocin | 2761295 | 2772596 | - | - |  |  |
| Cluster 13 | Terpene | 2797534 | 2819723 | - | - | 1 | CYP180A26 |
| Cluster 14 | Phosphonate-Terpene | 2914963 | 2978479 | FR900098_biosynthetic_gene_cluster (27% of genes show similarity) | BGC0000904_c1 |  |  |
| Cluster 15 | Siderophore | 3069319 | 3082416 | Marineosin_biosynthetic_gene_cluster (9% of genes show similarity) | BGC0000091_c1 |  |  |
| Cluster 16 | Terpene | 3525277 | 3551973 | Hopene_biosynthetic_gene_cluster (84% of genes show similarity) | BGC0000663_c1 |  |  |
| Cluster 17 | T1pks-Nrps | 3577665 | 3670783 | Filipin_biosynthetic_gene_cluster (46% of genes show similarity) | BGC0000059_c1 |  |  |
| Cluster 18 | Nrps | 3857540 | 3912587 | Avilamycin_A_biosynthetic_gene_cluster (5% of genes show similarity) | BGC0000026_c1 |  |  |
| Cluster 19 | Other | 3937513 | 3978886 | A-503083_biosynthetic_gene_cluster (7% of genes show similarity) | BGC0000288_c1 |  |  |
| Cluster 20 | Bacteriocin-Lantipeptide | 4145274 | 4177698 | Informatipeptin_biosynthetic_gene_cluster (100% of genes show similarity) | BGC0000518_c1 |  |  |
| Cluster 21 | Lantipeptide-T1pks-Nrps | 4509770 | 4638651 | Meilingmycin_biosynthetic_gene_cluster (16% of genes show similarity) | BGC0000093_c1 | 2 | CYP208A9; CYP107BK3 |
| Cluster 22 | Bacteriocin-T1pks | 4660365 | 4727894 | Blasticidin_biosynthetic_gene_cluster (17% of genes show similarity) | BGC0000874_c1 |  |  |
| Cluster 23 | Lantipeptide | 4734648 | 4757377 | Blasticidin_biosynthetic_gene_cluster (60% of genes show similarity) | BGC0000874_c1 |  |  |
| Cluster 24 | Thiopeptide | 4867845 | 4896118 | Lactazole_biosynthetic_gene_cluster (66% of genes show similarity) | BGC0000606_c1 |  |  |
| Cluster 25 | Lantipeptide-T1pks-Otherks | 5181716 | 5260298 | A54145_biosynthetic_gene_cluster (3% of genes show similarity) | BGC0000291_c1 |  |  |
| Cluster 26 | Nrps | 5367740 | 5446027 | Napsamycin_biosynthetic_gene_cluster (82% of genes show similarity) | BGC0000950_c1 | 1 | CYP107KX1 |
| Cluster 27 | Ladderane | 5670509 | 5712983 | Metatricycloene_biosynthetic_gene_cluster (32% of genes show similarity) | BGC0001369_c1 |  |  |
| Cluster 28 | Bacteriocin | 5821912 | 5832472 | Streptothricin_biosynthetic_gene_cluster (8% of genes show similarity) | BGC0000432_c1 |  |  |
| Cluster 29 | Lantipeptide | 5857594 | 5887006 | - | - |  |  |
| Cluster 30 | Other | 6211240 | 6251734 | Kanamycin_biosynthetic_gene_cluster (1% of genes show similarity) | BGC0000703_c1 | 2 | CYP121A4; CYP154U6 |
| Cluster 31 | Lassopeptide | 6260073 | 6282641 | - | - |  |  |
| Cluster 32 | Melanin-Terpene | 6357273 | 6382845 | Melanin_biosynthetic_gene_cluster (57% of genes show similarity) | BGC0000908_c1 |  |  |
| Cluster 33 | T1pks-Nrps | 6415636 | 6471337 | - | - |  |  |
| Cluster 34 | Terpene | 6484659 | 6505666 | - | - | 1 | CYP183X2 |
| Cluster 35 | Lantipeptide | 6664388 | 6702945 | - | - |  |  |
| Cluster 36 | Nucleoside | 6780943 | 6801308 | Toyocamycin_biosynthetic_gene_cluster (30% of genes show similarity) | BGC0000881_c1 |  |  |
| Cluster 37 | Terpene | 6813225 | 6834391 | 2-methylisoborneol_biosynthetic_gene_cluster (75% of genes show similarity) | BGC0000657_c1 |  |  |
| Cluster 38 | Siderophore | 6843831 | 6857240 | - | - |  |  |
| Cluster 39 | Siderophore | 7263646 | 7276995 | - | - |  |  |
| Cluster 40 | Phosphonate-T3pks-Nrps-Ladderane | 7289629 | 7395040 | Herboxidiene_biosynthetic_gene_cluster (8% of genes show similarity) | BGC0001065_c1 | 1 | CYP158A21 |
| Cluster 41 | T1pks-Otherks | 7616533 | 7668101 | Cinnamycin_biosynthetic_gene_cluster (14% of genes show similarity) | BGC0000503_c1 |  |  |
| Cluster 42 | Other | 8072352 | 8116209 | - | - |  |  |
| Cluster 43 | Ectoine | 8338442 | 8348846 | Ectoine_biosynthetic_gene_cluster (100% of genes show similarity) | BGC0000853_c1 |  |  |
| Cluster 44 | Lassopeptide | 8845999 | 8868499 | Chaxapeptin_biosynthetic_gene_cluster (28% of genes show similarity) | BGC0001307_c1 |  |  |
| Cluster 45 | Terpene | 8994774 | 9016135 | Kanamycin_biosynthetic_gene_cluster (2% of genes show similarity) | BGC0000703_c1 |  |  |
| Cluster 46 | Melanin-Nrps | 9444124 | 9491130 | Melanin_biosynthetic_gene_cluster (80% of genes show similarity) | BGC0000909_c1 |  |  |
| Cluster 47 | Siderophore | 9562300 | 9574069 | Desferrioxamine_B_biosynthetic_gene_cluster (100% of genes show similarity) | BGC0000940_c1 |  |  |
| Cluster 48 | T2pks-T1pks | 10278621 | 10365301 | Spore_pigment_biosynthetic_gene_cluster (83% of genes show similarity) | BGC0000271_c1 |  |  |
| Cluster 49 | T2pks-Oligosaccharide-Nrps-Otherks | 10485649 | 10561348 | Saquayamycin_Z_/_galtamycin_B_biosynthetic_gene_cluster (41% of genes show similarity) | BGC0000267_c1 |  |  |
| ***Streptomyces* sp. S10(2016)** | | | | | | | |
| Cluster 1 | Other | 224145 | 266760 | A-500359s_biosynthetic_gene_cluster (5% of genes show similarity) | BGC0000949_c1 |  |  |
| Cluster 2 | Other | 343674 | 387549 | - | - |  |  |
| Cluster 3 | Melanin | 544336 | 554704 | Melanin_biosynthetic_gene_cluster (57% of genes show similarity) | BGC0000908_c1 |  |  |
| Cluster 4 | T3pks-Nrps | 924883 | 1081396 | Feglymycin_biosynthetic_gene_cluster (57% of genes show similarity) | BGC0001233_c1 |  |  |
| Cluster 5 | Ectoine | 2149543 | 2159941 | Ectoine_biosynthetic_gene_cluster (100% of genes show similarity) | BGC0000853_c1 |  |  |
| Cluster 6 | Terpene-Nrps | 2878958 | 2945938 | SCO-2138_biosynthetic_gene_cluster (71% of genes show similarity) | BGC0000595_c1 |  |  |
| Cluster 7 | Melanin | 3247945 | 3258424 | Istamycin_biosynthetic_gene_cluster (5% of genes show similarity) | BGC0000700_c1 |  |  |
| Cluster 8 | Siderophore | 3354794 | 3366563 | Desferrioxamine_B_biosynthetic_gene_cluster (100% of genes show similarity) | BGC0000940_c1 |  |  |
| Cluster 9 | Thiopeptide | 3912000 | 3938554 | Platencin_biosynthetic_gene_cluster (9% of genes show similarity) | BGC0001156_c1 |  |  |
| Cluster 10 | Butyrolactone | 4637521 | 4648519 | Pristinamycin_biosynthetic_gene_cluster (3% of genes show similarity) | BGC0000952_c3 | 1 | CYP154K3 |
| Cluster 11 | Butyrolactone | 4778184 | 4789218 | Methylenomycin_biosynthetic_gene_cluster (23% of genes show similarity) | BGC0000914_c1 |  |  |
| Cluster 12 | Nrps | 4941976 | 4998990 | A54145_biosynthetic_gene_cluster (3% of genes show similarity) | BGC0000291_c1 |  |  |
| Cluster 13 | Terpene | 5837066 | 5862615 | Isorenieratene_biosynthetic_gene_cluster (100% of genes show similarity) | BGC0000664_c1 |  |  |
| Cluster 14 | Terpene | 6000626 | 6021549 | Albaflavenone_biosynthetic_gene_cluster (100% of genes show similarity) | BGC0000660_c1 | 1 | CYP170A15 |
| Cluster 15 | T2pks | 6221507 | 6264022 | Spore_pigment_biosynthetic_gene_cluster (83% of genes show similarity) | BGC0000271_c1 |  |  |
| Cluster 16 | Siderophore | 6648574 | 6660568 | - | - |  |  |
| Cluster 17 | T1pks | 6803083 | 6866736 | Streptazone_E_biosynthetic_gene_cluster (75% of genes show similarity) | BGC0001296_c1 |  |  |
| Cluster 18 | Bacteriocin | 6993517 | 7005028 | - | - |  |  |
| Cluster 19 | Terpene | 7037519 | 7059687 | - | - |  |  |
| Cluster 20 | Siderophore | 7289433 | 7302563 | Lividomycin_biosynthetic_gene_cluster (6% of genes show similarity) | BGC0000708_c1 |  |  |
| Cluster 21 | Nrps | 7374569 | 7511121 | Telomycin_biosynthetic_gene_cluster (61% of genes show similarity) | BGC0001406_c1 | 2 | CYP163B15; CYP113Z1 |
| Cluster 22 | Terpene | 7918247 | 7944944 | Hopene_biosynthetic_gene_cluster (92% of genes show similarity) | BGC0000663_c1 |  |  |
| Cluster 23 | Nrps | 8045066 | 8095973 | Coelichelin_biosynthetic_gene_cluster (100% of genes show similarity) | BGC0000325_c1 |  |  |
| Cluster 24 | Bacteriocin-Lantipeptide | 8623448 | 8655847 | Informatipeptin_biosynthetic_gene_cluster (85% of genes show similarity) | BGC0000518_c1 |  |  |
| Cluster 25 | T1pks | 8723410 | 8767378 | Oxazolomycin_biosynthetic_gene_cluster (9% of genes show similarity) | BGC0001106_c1 |  |  |
| Cluster 26 | Indole-T3pks | 8857041 | 8900715 | 7-prenylisatin_biosynthetic_gene_cluster (60% of genes show similarity) | BGC0001294_c1 |  |  |
| Cluster 27 | Terpene | 8976981 | 8998324 | 2-methylisoborneol_biosynthetic_gene_cluster (100% of genes show similarity) | BGC0000658_c1 |  |  |
| ***Streptomyces lincolnensis*** | | | | | | | |
| Cluster 1 | Other | 249090 | 291954 | Herbimycin_biosynthetic_gene_cluster (10% of genes show similarity) | BGC0000074_c1 |  |  |
| Cluster 2 | Other | 299197 | 339826 | Lincomycin_biosynthetic_gene_cluster (75% of genes show similarity) | BGC0000907_c1 |  |  |
| Cluster 3 | Ladderane | 451428 | 492678 | Skyllamycin_biosynthetic_gene_cluster (12% of genes show similarity) | BGC0000429_c1 | 1 | CYP105B41 |
| Cluster 4 | Melanin-Terpene | 576129 | 602496 | Melanin_biosynthetic_gene_cluster (57% of genes show similarity) | BGC0000908_c1 |  |  |
| Cluster 5 | Nrps | 863441 | 922947 | Pristinamycin_biosynthetic_gene_cluster (12% of genes show similarity) | BGC0000952_c1 | 1 | CYP163A5 |
| Cluster 6 | Nrps | 1585396 | 1641767 | Coelibactin_biosynthetic_gene_cluster (63% of genes show similarity) | BGC0000324_c1 | 1 | CYP1424A1 |
| Cluster 7 | T3pks | 1646746 | 1687819 | Herboxidiene_biosynthetic_gene_cluster (8% of genes show similarity) | BGC0001065_c1 | 1 | CYP158A15 |
| Cluster 8 | Terpene | 2183010 | 2204059 | - | - | 2 | CYP183Y1; CYP157C22 |
| Cluster 9 | Other | 2262790 | 2306653 | Pristinamycin_biosynthetic_gene_cluster (2% of genes show similarity) | BGC0000952_c3 |  |  |
| Cluster 10 | Ectoine | 2542017 | 2552421 | Ectoine_biosynthetic_gene_cluster (100% of genes show similarity) | BGC0000853_c1 |  |  |
| Cluster 11 | Butyrolactone | 3105357 | 3115644 | - | - |  |  |
| Cluster 12 | Melanin | 3706522 | 3716998 | Istamycin_biosynthetic_gene_cluster (5% of genes show similarity) | BGC0000700_c1 |  |  |
| Cluster 13 | Siderophore | 3814323 | 3826095 | Desferrioxamine_B_biosynthetic_gene_cluster (100% of genes show similarity) | BGC0000940_c1 |  |  |
| Cluster 14 | Ladderane | 5253351 | 5294520 | Colabomycin_biosynthetic_gene_cluster (13% of genes show similarity) | BGC0000213_c1 |  |  |
| Cluster 15 | Amglyccycl-Butyrolactone | 6047626 | 6079735 | Pyralomicin_biosynthetic_gene_cluster (18% of genes show similarity) | BGC0001038_c1 |  |  |
| Cluster 16 | Terpene | 6671108 | 6692193 | Albaflavenone_biosynthetic_gene_cluster (100% of genes show similarity) | BGC0000660_c1 | 1 | CYP170A20 |
| Cluster 17 | T1pks-Nrps | 6978487 | 7040117 | BD-12_biosynthetic_gene_cluster (17% of genes show similarity) | BGC0001379_c1 |  |  |
| Cluster 18 | Siderophore | 7415509 | 7427461 | - | - |  |  |
| Cluster 19 | Phosphoglycolipid | 7574317 | 7603039 | Teichomycin_biosynthetic_gene_cluster (88% of genes show similarity) | BGC0001395_c1 |  |  |
| Cluster 20 | Bacteriocin-T2pks | 7704225 | 7765020 | Pamamycin_biosynthetic_gene_cluster (95% of genes show similarity) | BGC0001150_c1 |  |  |
| Cluster 21 | Terpene-Butyrolactone | 7842166 | 7872226 | Gamma-butyrolactone_biosynthetic_gene_cluster (100% of genes show similarity) | BGC0000850_c1 |  |  |
| Cluster 22 | Other | 7902702 | 7944810 | Medermycin_biosynthetic_gene_cluster (13% of genes show similarity) | BGC0000245_c1 |  |  |
| Cluster 23 | Nrps-Siderophore | 8091854 | 8156922 | Grincamycin_biosynthetic_gene_cluster (8% of genes show similarity) | BGC0000229_c1 | 1 | CYP107L32 |
| Cluster 24 | T2pks | 8254465 | 8296926 | Kanamycin_biosynthetic_gene_cluster (4% of genes show similarity) | BGC0000703_c1 | 1 | CYP105B41 |
| Cluster 25 | Nrps | 8404510 | 8455426 | Coelichelin_biosynthetic_gene_cluster (100% of genes show similarity) | BGC0000325_c1 |  |  |
| Cluster 26 | Terpene | 8628322 | 8654989 | Hopene_biosynthetic_gene_cluster (92% of genes show similarity) | BGC0000663_c1 |  |  |
| Cluster 27 | T1pks | 8690932 | 8739022 | Meilingmycin_biosynthetic_gene_cluster (5% of genes show similarity) | BGC0000093_c1 |  |  |
| Cluster 28 | Terpene | 9074027 | 9095814 | Ansatrienin_(mycotrienin)_biosynthetic_gene_cluster (11% of genes show similarity) | BGC0000957_c1 |  |  |
| Cluster 29 | Bacteriocin-Lantipeptide | 9254500 | 9284511 | Informatipeptin_biosynthetic_gene_cluster (71% of genes show similarity) | BGC0000518_c1 |  |  |
| Cluster 30 | Siderophore | 9308354 | 9323052 | Scabichelin_biosynthetic_gene_cluster (20% of genes show similarity) | BGC0000423_c1 |  |  |
| Cluster 31 | T1pks | 9567580 | 9611593 | Daptomycin_biosynthetic_gene_cluster (4% of genes show similarity) | BGC0000336_c1 |  |  |
| Cluster 32 | Nrps | 9921582 | 9972122 | Incednine_biosynthetic_gene_cluster (5% of genes show similarity) | BGC0000078_c1 |  |  |
| Cluster 33 | T1pks-Nrps | 10025162 | 10106144 | Antimycin_biosynthetic_gene_cluster (93% of genes show similarity) | BGC0000958_c1 |  |  |
| Cluster 34 | T1pks | 10167404 | 10211375 | Lomofungin_biosynthetic_gene_cluster (8% of genes show similarity) | BGC0001302_c1 |  |  |
| ***Streptomyces noursei*** | | | | | | | |
| Cluster 1 | Lassopeptide | 116803 | 139284 | A54145_biosynthetic_gene_cluster (5% of genes show similarity) | BGC0000291_c1 |  |  |
| Cluster 2 | Terpene | 222311 | 243384 | 2-methylisoborneol_biosynthetic_gene_cluster (75% of genes show similarity) | BGC0000657_c1 |  |  |
| Cluster 3 | T1pks | 349984 | 410183 | Ebelactone_biosynthetic_gene_cluster (60% of genes show similarity) | BGC0000051_c1 | 5 | CYP105BV1; CYP1198A1; CYP107EM1; CYP1060A2; CYP147F29 |
| Cluster 4 | Terpene | 584083 | 604979 | - | - |  |  |
| Cluster 5 | Terpene | 688002 | 708970 | - | - | 1 | CYP157C31 |
| Cluster 6 | T1pks | 768290 | 912056 | Nystatin_biosynthetic_gene_cluster (100% of genes show similarity) | BGC0000115_c1 | 2 | CYP105H1; CYP161A1 |
| Cluster 7 | Terpene | 1064783 | 1085721 | - | - |  |  |
| Cluster 8 | Butyrolactone | 1243689 | 1254624 | Merochlorin_biosynthetic_gene_cluster (4% of genes show similarity) | BGC0001083_c1 | 1 | CYP107AE10 |
| Cluster 9 | Butyrolactone | 1473958 | 1530565 | Sch47554_/_Sch47555_biosynthetic_gene_cluster (10% of genes show similarity) | BGC0000268_c1 |  |  |
| Cluster 10 | Other | 1573019 | 1616555 | 4-hydroxy-3-nitrosobenzamide_biosynthetic_gene_cluster (21% of genes show similarity) | BGC0000885_c1 | 1 | CYP1248A3 |
| Cluster 11 | T1pks | 1644455 | 1691954 | Actinomycin_biosynthetic_gene_cluster (10% of genes show similarity) | BGC0000296_c1 | 1 | CYP107EQ1 |
| Cluster 12 | Terpene | 1957660 | 1984391 | Hopene_biosynthetic_gene_cluster (61% of genes show similarity) | BGC0000663_c1 |  |  |
| Cluster 13 | Lassopeptide | 2156897 | 2179430 | Echosides_biosynthetic_gene_cluster (11% of genes show similarity) | BGC0000340_c1 |  |  |
| Cluster 14 | T1pks | 2213917 | 2292303 | Erythromycin_biosynthetic_gene_cluster (60% of genes show similarity) | BGC0000055_c1 | 3 | CYP107KZ1; CYP1420A1; CYP107A3 |
| Cluster 15 | Bacteriocin | 2670339 | 2681181 | - | - |  |  |
| Cluster 16 | T3pks | 2741982 | 2783037 | FD-594_biosynthetic_gene_cluster (8% of genes show similarity) | BGC0000222_c1 | 1 | CYP107F9 |
| Cluster 17 | Nrps | 2853066 | 2915225 | Desotamide_biosynthetic_gene_cluster (63% of genes show similarity) | BGC0001196_c1 |  |  |
| Cluster 18 | Siderophore | 2921611 | 2936270 | - | - |  |  |
| Cluster 19 | Other | 3248238 | 3288957 | Albonoursin_biosynthetic_gene_cluster (83% of genes show similarity) | BGC0000851_c1 |  |  |
| Cluster 20 | Other | 3884567 | 3928700 | Meridamycin_biosynthetic_gene_cluster (5% of genes show similarity) | BGC0001011_c1 |  |  |
| Cluster 21 | Linaridin | 4361283 | 4382173 | Legonaridin_biosynthetic_gene_cluster (55% of genes show similarity) | BGC0001188_c1 |  |  |
| Cluster 22 | Thiopeptide-Bacteriocin | 4401731 | 4437294 | TP-1161_biosynthetic_gene_cluster (37% of genes show similarity) | BGC0000615_c1 | 1 | CYP113D4 |
| Cluster 23 | Terpene | 4523671 | 4545989 | Salinomycin_biosynthetic_gene_cluster (6% of genes show similarity) | BGC0000144_c1 |  |  |
| Cluster 24 | Lantipeptide | 6032833 | 6057286 | - | - |  |  |
| Cluster 25 | T1pks | 6511142 | 6554210 | Collismycin_A_biosynthetic_gene_cluster (18% of genes show similarity) | BGC0000973_c1 | 1 | CYP105AB15 |
| Cluster 26 | Ectoine | 6984258 | 6994662 | Ectoine_biosynthetic_gene_cluster (100% of genes show similarity) | BGC0000853_c1 |  |  |
| Cluster 27 | Siderophore | 7075833 | 7087638 | Desferrioxamine_B_biosynthetic_gene_cluster (80% of genes show similarity) | BGC0000941_c1 |  |  |
| Cluster 28 | Lantipeptide | 7331458 | 7356001 | Streptomycin_biosynthetic_gene_cluster (12% of genes show similarity) | BGC0000717_c1 |  |  |
| Cluster 29 | T2pks | 7864563 | 7907078 | Spore_pigment_biosynthetic_gene_cluster (83% of genes show similarity) | BGC0000271_c1 |  |  |
| Cluster 30 | Bacteriocin | 7950154 | 7960369 | - | - |  |  |
| Cluster 31 | Transatpks-Nrps | 8270365 | 8375658 | Cycloheximide_/_actiphenol_biosynthetic_gene_cluster (50% of genes show similarity) | BGC0000175_c1 | 3 | CYP107CA2; CYP154D14; CYP105AC16 |
| Cluster 32 | Nrps | 8386157 | 8448082 | Friulimicin_biosynthetic_gene_cluster (12% of genes show similarity) | BGC0000354_c1 |  |  |
| Cluster 33 | Terpene | 8910362 | 8931330 | Filipin_biosynthetic_gene_cluster (15% of genes show similarity) | BGC0000059_c1 | 1 | CYP105A7 |
| Cluster 34 | Terpene-T3pks-T1pks-Nrps | 9016679 | 9111228 | Leinamycin_biosynthetic_gene_cluster (2% of genes show similarity) | BGC0001101_c1 |  |  |
| Cluster 35 | Lantipeptide-Nrps | 9139090 | 9243844 | Zorbamycin_biosynthetic_gene_cluster (6% of genes show similarity) | BGC0001058_c1 | 2 | CYP163B; CYP1278B1 |
| Cluster 36 | Terpene-T1pks | 9247044 | 9370657 | Nystatin_biosynthetic_gene_cluster (72% of genes show similarity) | BGC0000115_c1 | 1 | CYP105H8 |
| Cluster 37 | Lassopeptide | 9676601 | 9699082 | A54145_biosynthetic_gene_cluster (5% of genes show similarity) | BGC0000291_c1 |  |  |

**Mycobacterial species**

| ***Mycobacterium africanum*GM041182** | | | | | | | |
| --- | --- | --- | --- | --- | --- | --- | --- |
| Cluster | Type | From | To | Most similar known cluster | MIBiG BGC-ID | P450s | Name of the P450 |
|  |  |  |  |  |  |  |  |
| Cluster 1 | Nrps | 88242 | 137625 | - | - |  |  |
| Cluster 2 | T1pks | 462468 | 506676 | - | - |  |  |
| Cluster 3 | Bacteriocin | 882527 | 893324 | - | - |  |  |
| Cluster 4 | Nrps | 1298767 | 1355947 | Glycopeptidolipid_biosynthetic_gene_cluster (13% of genes show similarity) | BGC0000365_c1 |  |  |
| Cluster 5 | T3pks | 1528107 | 1569288 | BE-7585A_biosynthetic_gene_cluster (14% of genes show similarity) | BGC0000203_c1 |  |  |
| Cluster 6 | T1pks | 1705714 | 1752040 | Glycopeptidolipid_biosynthetic_gene_cluster (20% of genes show similarity) | BGC0000365_c1 |  |  |
| Cluster 7 | T3pks-T1pks | 1850834 | 1904620 | Streptomycin_biosynthetic_gene_cluster (12% of genes show similarity) | BGC0000717_c1 | 1 | CYP139A1 |
| Cluster 8 | Nrps | 1950426 | 1992024 | - | - |  |  |
| Cluster 9 | T1pks | 2267339 | 2319794 | Reveromycin_biosynthetic_gene_cluster (15% of genes show similarity) | BGC0000135_c1 |  |  |
| Cluster 10 | Other | 2514759 | 2555628 | - | - | 3 | CYP124A1; CYP128A1; CYP121A1 |
| Cluster 11 | Nrps | 2625730 | 2685663 | Mycobactin_biosynthetic_gene_cluster (100% of genes show similarity) | BGC0001021_c1 |  |  |
| Cluster 12 | Other | 2885311 | 2928817 | - | - |  |  |
| Cluster 13 | T1pks-Nrps | 3203861 | 3298599 | Glycopeptidolipid_biosynthetic_gene_cluster (6% of genes show similarity) | BGC0000365_c1 |  |  |
| Cluster 14 | Terpene | 3787393 | 3808301 | Hopene_biosynthetic_gene_cluster (15% of genes show similarity) | BGC0000663_c1 |  |  |
| Cluster 15 | T1pks-Nrps | 4213486 | 4299107 | Glycopeptidolipid_biosynthetic_gene_cluster (10% of genes show similarity) | BGC0000365_c |  |  |
|  |  |  |  | ***Mycobacterium tuberculosis*C** |  |  |  |
| Cluster 1 | Nrps | 88185 | 137568 | - | - |  |  |
| Cluster 2 | T1pks | 465451 | 509659 | - | - |  |  |
| Cluster 3 | Bacteriocin | 885597 | 896394 | - | - |  |  |
| Cluster 4 | T1pks-Nrps | 1294041 | 1347876 | Glycopeptidolipid_biosynthetic_gene_cluster (10% of genes show similarity) | BGC0000365_c1 |  |  |
| Cluster 5 | T3pks | 1523766 | 1564947 | BE-7585A_biosynthetic_gene_cluster (14% of genes show similarity) | BGC0000203_c1 |  |  |
| Cluster 6 | T1pks | 1702580 | 1748906 | Glycopeptidolipid_biosynthetic_gene_cluster (20% of genes show similarity) | BGC0000365_c1 |  |  |
| Cluster 7 | T3pks-T1pks | 1845411 | 1904899 | Streptomycin_biosynthetic_gene_cluster (12% of genes show similarity) | BGC0000717_c1 | 1 | CYP139A1 |
| Cluster 8 | Nrps | 1950635 | 2006069 | Glycopeptidolipid_biosynthetic_gene_cluster (20% of genes show similarity) | BGC0000362_c1 | 1 | CYP144A1 |
| Cluster 9 | T1pks | 2274453 | 2326911 | Reveromycin_biosynthetic_gene_cluster (15% of genes show similarity) | BGC0000135_c1 |  |  |
| Cluster 10 | Other | 2523905 | 2564774 | Glycopeptidolipid_biosynthetic_gene_cluster (20% of genes show similarity) | BGC0000362_c1 | 3 | CYP124A1; CYP128A1; CYP121A1 |
| Cluster 11 | Nrps | 2633986 | 2693919 | Mycobactin_biosynthetic_gene_cluster (100% of genes show similarity) | BGC0001021_c1 |  |  |
| Cluster 12 | Other | 2892773 | 2936279 | - | - |  |  |
| Cluster 13 | T1pks-Nrps | 3221603 | 3316055 | Glycopeptidolipid_biosynthetic_gene_cluster (10% of genes show similarity) | BGC0000365_c1 |  |  |
| Cluster 14 | Terpene | 3801146 | 3822054 | Hopene_biosynthetic_gene_cluster (15% of genes show similarity) | BGC0000663_c1 |  |  |
| Cluster 15 | T1pks-Nrps | 4222934 | 4308555 | Glycopeptidolipid_biosynthetic_gene_cluster (10% of genes show similarity) | BGC0000365_c1 |  |  |
|  |  |  |  | ***Mycobacterium tuberculosis* F11** |  |  |  |
| Cluster 1 | Nrps | 88290 | 137673 | - | - |  |  |
| Cluster 2 | T1pks | 468901 | 513109 | - | - |  |  |
| Cluster 3 | Bacteriocin | 888983 | 899780 | - | - |  |  |
| Cluster 4 | T1pks-Nrps | 1297442 | 1360980 | Glycopeptidolipid_biosynthetic_gene_cluster (10% of genes show similarity) | BGC0000365_c1 |  |  |
| Cluster 5 | T3pks | 1529166 | 1570347 | BE-7585A_biosynthetic_gene_cluster (14% of genes show similarity) | BGC0000203_c1 |  |  |
| Cluster 6 | T1pks | 1706720 | 1753046 | Glycopeptidolipid_biosynthetic_gene_cluster (20% of genes show similarity) | BGC0000365_c1 |  |  |
| Cluster 7 | T3pks-T1pks | 1849550 | 1907677 | Streptomycin_biosynthetic_gene_cluster (12% of genes show similarity) | BGC0000717_c1 | 1 | CYP139A1 |
| Cluster 8 | T1pks | 2289143 | 2341598 | Reveromycin_biosynthetic_gene_cluster (15% of genes show similarity) | BGC0000135_c1 |  |  |
| Cluster 9 | Other | 2539029 | 2579898 | - | - | 3 | CYP124A1; CYP128A1; CYP121A1 |
| Cluster 10 | Nrps | 2650995 | 2710928 | Mycobactin_biosynthetic_gene_cluster (100% of genes show similarity) | BGC0001021_c1 |  |  |
| Cluster 11 | Other | 2911188 | 2954694 | - | - |  |  |
| Cluster 12 | T1pks-Nrps | 3236918 | 3331427 | Glycopeptidolipid_biosynthetic_gene_cluster (10% of genes show similarity) | BGC0000365_c1 |  |  |
| Cluster 13 | Terpene | 3815207 | 3836115 | Hopene_biosynthetic_gene_cluster (15% of genes show similarity) | BGC0000663_c1 |  |  |
| Cluster 14 | T1pks-Nrps | 4248946 | 4334567 | Glycopeptidolipid_biosynthetic_gene_cluster (10% of genes show similarity) | BGC0000365_c1 |  |  |
|  |  |  |  | ***Mycobacterium tuberculosis* H37Ra** |  |  |  |
| Cluster 1 | Nrps | 89514 | 138897 | - | - |  |  |
| Cluster 2 | T1pks | 467041 | 511249 | - | - |  |  |
| Cluster 3 | Bacteriocin | 887778 | 898575 | Glycopeptidolipid_biosynthetic_gene_cluster (20% of genes show similarity) | BGC0000362_c1 |  |  |
| Cluster 4 | T1pks-Nrps | 1295036 | 1358526 | Glycopeptidolipid_biosynthetic_gene_cluster (10% of genes show similarity) | BGC0000365_c1 |  |  |
| Cluster 5 | T3pks | 1526135 | 1567316 | BE-7585A_biosynthetic_gene_cluster (14% of genes show similarity) | BGC0000203_c1 |  |  |
| Cluster 6 | T1pks | 1703600 | 1749926 | Glycopeptidolipid_biosynthetic_gene_cluster (20% of genes show similarity) | BGC0000365_c1 |  |  |
| Cluster 7 | T3pks-T1pks | 1855678 | 1913805 | Streptomycin_biosynthetic_gene_cluster (12% of genes show similarity) | BGC0000717_c1 | 1 | CYP139A1 |
| Cluster 8 | T1pks | 2284431 | 2336886 | Reveromycin_biosynthetic_gene_cluster (15% of genes show similarity) | BGC0000135_c1 | 1 | CYP139A1 |
| Cluster 9 | Other | 2536787 | 2577656 | Glycopeptidolipid_biosynthetic_gene_cluster (20% of genes show similarity) | BGC0000362_c1 | 2 | CYP124A1; CYP128A1 |
| Cluster 10 | Nrps | 2649668 | 2709601 | Mycobactin_biosynthetic_gene_cluster (100% of genes show similarity) | BGC0001021_c1 |  |  |
| Cluster 11 | Other | 2909839 | 2953345 | - | - |  |  |
| Cluster 12 | T1pks-Nrps | 3237437 | 3331946 | Glycopeptidolipid_biosynthetic_gene_cluster (10% of genes show similarity) | BGC0000365_c1 |  |  |
| Cluster 13 | Terpene | 3812768 | 3833676 | Hopene_biosynthetic_gene_cluster (15% of genes show similarity) | BGC0000663_c1 |  |  |
| Cluster 14 | T1pks-Nrps | 4244391 | 4330012 | Glycopeptidolipid_biosynthetic_gene_cluster (10% of genes show similarity) | BGC0000365_c1 |  |  |
|  |  |  |  | ***Mycobacterium tuberculosis* H37Rv** |  |  |  |
| Cluster 1 | Nrps | 88156 | 137539 | - | - |  |  |
| Cluster 2 | T1pks | 465731 | 509939 | - | - |  |  |
| Cluster 3 | Bacteriocin | 886472 | 897269 | - | - |  |  |
| Cluster 4 | Nrps | 1300035 | 1357215 | Glycopeptidolipid_biosynthetic_gene_cluster (13% of genes show similarity) | BGC0000365_c1 |  |  |
| Cluster 5 | T3pks | 1524825 | 1566006 | BE-7585A_biosynthetic_gene_cluster (14% of genes show similarity) | BGC0000203_c1 |  |  |
| Cluster 6 | T1pks | 1702083 | 1748409 | Glycopeptidolipid_biosynthetic_gene_cluster (20% of genes show similarity) | BGC0000365_c1 |  |  |
| Cluster 7 | T3pks-T1pks | 1854160 | 1912287 | Streptomycin_biosynthetic_gene_cluster (12% of genes show similarity) | BGC0000717_c1 | 1 | CYP139A1 |
| Cluster 8 | Nrps | 1957969 | 2014661 | - | - | 1 | CYP144A1 |
| Cluster 9 | T1pks | 2274531 | 2326986 | Reveromycin_biosynthetic_gene_cluster (15% of genes show similarity) | BGC0000135_c1 |  |  |
| Cluster 10 | Other | 2526883 | 2567752 | - | - | 2 | CYP124A1; CYP128A1 |
| Cluster 11 | Nrps | 2637700 | 2697633 | Mycobactin_biosynthetic_gene_cluster (100% of genes show similarity) | BGC0001021_c1 |  |  |
| Cluster 12 | Other | 2897871 | 2941377 | - | - |  |  |
| Cluster 13 | T1pks-Nrps | 3225445 | 3319954 | Glycopeptidolipid_biosynthetic_gene_cluster (10% of genes show similarity) | BGC0000365_c1 |  |  |
| Cluster 14 | Terpene | 3804090 | 3824998 | Hopene_biosynthetic_gene_cluster (15% of genes show similarity) | BGC0000663_c1 |  |  |
| Cluster 15 | T1pks-Nrps | 4235945 | 4321566 | Glycopeptidolipid_biosynthetic_gene_cluster (10% of genes show similarity) | BGC0000365_c1 |  |  |
|  |  |  |  | ***Mycobacterium tuberculosis*Haarlem** |  |  |  |
| Cluster 1 | Nrps | 88292 | 137675 | - | - |  |  |
| Cluster 2 | T1pks | 470237 | 514445 | Glycopeptidolipid_biosynthetic_gene_cluster (25% of genes show similarity) | BGC0000362_c1 |  |  |
| Cluster 3 | Bacteriocin | 890036 | 900833 | - | - |  |  |
| Cluster 4 | T1pks-Nrps | 1298849 | 1362387 | Glycopeptidolipid_biosynthetic_gene_cluster (10% of genes show similarity) | BGC0000365_c1 |  |  |
| Cluster 5 | T3pks | 1525449 | 1566630 | BE-7585A_biosynthetic_gene_cluster (11% of genes show similarity) | BGC0000203_c1 |  |  |
| Cluster 6 | T1pks | 1704363 | 1750689 | Glycopeptidolipid_biosynthetic_gene_cluster (38% of genes show similarity) | BGC0000362_c1 |  |  |
| Cluster 7 | T3pks-T1pks | 1847252 | 1905379 | Streptomycin_biosynthetic_gene_cluster (12% of genes show similarity) | BGC0000717_c1 | 1 | CYP139A1 |
| Cluster 8 | T1pks | 2279163 | 2331618 | Reveromycin_biosynthetic_gene_cluster (15% of genes show similarity) | BGC0000135_c1 |  |  |
| Cluster 9 | Nrps | 2633645 | 2693578 | Mycobactin_biosynthetic_gene_cluster (100% of genes show similarity) | BGC0001021_c1 |  |  |
| Cluster 10 | Other | 2893131 | 2936637 | - | - |  |  |
| Cluster 11 | T1pks-Nrps | 3221367 | 3315876 | Glycopeptidolipid_biosynthetic_gene_cluster (10% of genes show similarity) | BGC0000365_c1 |  |  |
| Cluster 12 | Terpene | 3797784 | 3818692 | Hopene_biosynthetic_gene_cluster (15% of genes show similarity) | BGC0000663_c1 |  |  |
| Cluster 13 | T1pks-Nrps | 4232784 | 4318405 | Glycopeptidolipid_biosynthetic_gene_cluster (10% of genes show similarity) | BGC0000365_c1 |  |  |
|  |  |  |  | ***Mycobacterium tuberculosis* KZN 1435** |  |  |  |
| Cluster 1 | Nrps | 88287 | 137670 | - | - |  |  |
| Cluster 2 | T1pks | 465990 | 510198 | - | - |  |  |
| Cluster 3 | Bacteriocin | 886314 | 897111 | - | - |  |  |
| Cluster 4 | T1pks-Nrps | 1093650 | 1188160 | Glycopeptidolipid_biosynthetic_gene_cluster (10% of genes show similarity) | BGC0000365_c1 |  |  |
| Cluster 5 | Other | 1472438 | 1515944 | - | - |  |  |
| Cluster 6 | Nrps | 1716070 | 1776003 | Mycobactin_biosynthetic_gene_cluster (100% of genes show similarity) | BGC0001021_c1 |  |  |
| Cluster 7 | Other | 1848840 | 1889373 | Glycopeptidolipid_biosynthetic_gene_cluster (20% of genes show similarity) | BGC0000362_c1 | 3 | CYP121A1; CYP128A1; CYP124A1 |
| Cluster 8 | T1pks | 2089725 | 2142183 | Reveromycin_biosynthetic_gene_cluster (15% of genes show similarity) | BGC0000135_c1 |  |  |
| Cluster 9 | Nrps | 2409168 | 2464677 | Glycopeptidolipid_biosynthetic_gene_cluster (20% of genes show similarity) | BGC0000362_c1 | 1 | CYP144A1 |
| Cluster 10 | T3pks-T1pks | 2510425 | 2568552 | Streptomycin_biosynthetic_gene_cluster (12% of genes show similarity) | BGC0000717_c1 | 1 | CYP139A1 |
| Cluster 11 | T1pks | 2665057 | 2711383 | Glycopeptidolipid_biosynthetic_gene_cluster (17% of genes show similarity) | BGC0000362_c1 |  |  |
| Cluster 12 | T3pks | 2847698 | 2888879 | BE-7585A_biosynthetic_gene_cluster (14% of genes show similarity) | BGC0000203_c1 |  |  |
| Cluster 13 | T1pks-Nrps | 3055078 | 3118618 | Glycopeptidolipid_biosynthetic_gene_cluster (10% of genes show similarity) | BGC0000365_c1 |  |  |
| Cluster 14 | Terpene | 3798602 | 3819510 | Hopene_biosynthetic_gene_cluster (15% of genes show similarity) | BGC0000663_c1 |  |  |
| Cluster 15 | T1pks-Nrps | 4222891 | 4308512 | Glycopeptidolipid_biosynthetic_gene_cluster (10% of genes show similarity) | BGC0000365_c1 |  |  |
|  |  |  |  | ***Mycobacterium tuberculosis* KZN 605** |  |  |  |
| Cluster 1 | Nrps | 88285 | 137668 | - | - |  |  |
| Cluster 2 | T1pks | 465991 | 510199 | - | - |  |  |
| Cluster 3 | Bacteriocin | 886319 | 897116 | - | - |  |  |
| Cluster 4 | T1pks-Nrps | 1093652 | 1188160 | Glycopeptidolipid_biosynthetic_gene_cluster (10% of genes show similarity) | BGC0000365_c1 |  |  |
| Cluster 5 | Other | 1472550 | 1516056 | - | - |  |  |
| Cluster 6 | Nrps | 1716185 | 1776118 | Mycobactin_biosynthetic_gene_cluster (100% of genes show similarity) | BGC0001021_c1 |  |  |
| Cluster 7 | Other | 1848956 | 1889489 | Glycopeptidolipid_biosynthetic_gene_cluster (20% of genes show similarity) | BGC0000362_c1 | 3 | CYP121A1; CYP128A1; CYP124A1 |
| Cluster 8 | T1pks | 2089602 | 2142061 | Piericidin_A1_biosynthetic_gene_cluster (50% of genes show similarity) | BGC0000124_c1 |  |  |
| Cluster 9 | Nrps | 2409049 | 2464558 | Glycopeptidolipid_biosynthetic_gene_cluster (20% of genes show similarity) | BGC0000362_c1 | 1 | CYP144A1 |
| Cluster 10 | T3pks-T1pks | 2510307 | 2568434 | Streptomycin_biosynthetic_gene_cluster (12% of genes show similarity) | BGC0000717_c1 | 1 | CYP139A1 |
| Cluster 11 | T1pks | 2664940 | 2711266 | Glycopeptidolipid_biosynthetic_gene_cluster (17% of genes show similarity) | BGC0000362_c1 |  |  |
| Cluster 12 | T3pks | 2847469 | 2888650 | BE-7585A_biosynthetic_gene_cluster (14% of genes show similarity) | BGC0000203_c1 |  |  |
| Cluster 13 | T1pks-Nrps | 3054850 | 3118361 | Glycopeptidolipid_biosynthetic_gene_cluster (10% of genes show similarity) | BGC0000365_c1 |  |  |
| Cluster 14 | Terpene | 3799712 | 3820620 | Hopene_biosynthetic_gene_cluster (15% of genes show similarity) | BGC0000663_c1 |  |  |
| Cluster 15 | T1pks-Nrps | 4223760 | 4309381 | Glycopeptidolipid_biosynthetic_gene_cluster (10% of genes show similarity) | BGC0000365_c1 |  |  |
|  |  |  |  | ***Mycobacterium tuberculosis* KZN 4207** |  |  |  |
| Cluster 1 | Nrps | 88286 | 137669 | - | - |  |  |
| Cluster 2 | T1pks | 465990 | 510198 | - | - |  |  |
| Cluster 3 | Bacteriocin | 886317 | 897114 | - | - |  |  |
| Cluster 4 | T1pks-Nrps | 1093650 | 1188158 | Glycopeptidolipid_biosynthetic_gene_cluster (10% of genes show similarity) | BGC0000365_c1 |  |  |
| Cluster 5 | Other | 1472382 | 1515888 | - | - |  |  |
| Cluster 6 | Nrps | 1715957 | 1775890 | Mycobactin_biosynthetic_gene_cluster (100% of genes show similarity) | BGC0001021_c1 |  |  |
| Cluster 7 | Other | 1848727 | 1889260 | Glycopeptidolipid_biosynthetic_gene_cluster (20% of genes show similarity) | BGC0000362_c1 | 3 | CYP121A1; CYP128A1; CYP124A1 |
| Cluster 8 | T1pks | 2087814 | 2140272 | Reveromycin_biosynthetic_gene_cluster (15% of genes show similarity) | BGC0000135_c1 |  |  |
| Cluster 9 | Nrps | 2405898 | 2461407 | Glycopeptidolipid_biosynthetic_gene_cluster (20% of genes show similarity) | BGC0000362_c1 | 1 | CYP144A1 |
| Cluster 10 | T3pks-T1pks | 2507098 | 2565225 | Streptomycin_biosynthetic_gene_cluster (12% of genes show similarity) | BGC0000717_c1 | 1 | CYP139A1 |
| Cluster 11 | T1pks | 2661730 | 2708056 | Glycopeptidolipid_biosynthetic_gene_cluster (17% of genes show similarity) | BGC0000362_c1 |  |  |
| Cluster 12 | T3pks | 2844371 | 2885552 | BE-7585A_biosynthetic_gene_cluster (14% of genes show similarity) | BGC0000203_c1 |  |  |
| Cluster 13 | T1pks-Nrps | 3051750 | 3115288 | Glycopeptidolipid_biosynthetic_gene_cluster (10% of genes show similarity) | BGC0000365_c1 |  |  |
| Cluster 14 | Terpene | 3795336 | 3816244 | Hopene_biosynthetic_gene_cluster (15% of genes show similarity) | BGC0000663_c1 |  |  |
| Cluster 15 | T1pks-Nrps | 4219495 | 4305116 | Glycopeptidolipid_biosynthetic_gene_cluster (10% of genes show similarity) | BGC0000365_c1 |  |  |
|  |  |  |  | ***Mycobacterium  tuberculosis*RGTB327** |  |  |  |
| Cluster 1 | Nrps | 90060 | 137277 | Glycopeptidolipid_biosynthetic_gene_cluster (7% of genes show similarity) | BGC0000362_c1 |  |  |
| Cluster 2 | Bacteriocin | 887005 | 897802 | Glycopeptidolipid_biosynthetic_gene_cluster (20% of genes show similarity) | BGC0000362_c1 |  |  |
| Cluster 3 | Nrps | 1300846 | 1358039 | Glycopeptidolipid_biosynthetic_gene_cluster (6% of genes show similarity) | BGC0000365_c1 |  |  |
| Cluster 4 | T3pks | 1525759 | 1566940 | BE-7585A_biosynthetic_gene_cluster (14% of genes show similarity) | BGC0000203_c1 |  |  |
| Cluster 5 | T3pks-T1pks | 1850431 | 1907434 | Streptomycin_biosynthetic_gene_cluster (10% of genes show similarity) | BGC0000717_c1 |  |  |
| Cluster 6 | Other | 2520687 | 2561202 | Glycopeptidolipid_biosynthetic_gene_cluster (20% of genes show similarity) | BGC0000362_c1 | 2 | CYP124A1; CYP102B25 |
| Cluster 7 | Other | 2891575 | 2935081 | - | - |  |  |
| Cluster 8 | T1pks-Nrps | 3226347 | 3313899 | Candicidin_biosynthetic_gene_cluster (33% of genes show similarity) | BGC0000034_c1 |  |  |
| Cluster 9 | Terpene | 3773355 | 3794263 | Hopene_biosynthetic_gene_cluster (15% of genes show similarity) | BGC0000663_c1 |  |  |
| Cluster 10 | Nrps | 4232920 | 4290071 | Glycopeptidolipid_biosynthetic_gene_cluster (10% of genes show similarity) | BGC0000365_c1 |  |  |
|  |  |  |  | ***Mycobacterium tuberculosis* CDC1551** |  |  |  |
| Cluster 1 | Nrps | 88146 | 137529 | - | - |  |  |
| Cluster 2 | T1pks | 467180 | 511388 | Glycopeptidolipid_biosynthetic_gene_cluster (25% of genes show similarity) | BGC0000362_c1 |  |  |
| Cluster 3 | Bacteriocin | 886381 | 897178 | - | - |  |  |
| Cluster 4 | T1pks-Nrps | 1293164 | 1356702 | Glycopeptidolipid_biosynthetic_gene_cluster (10% of genes show similarity) | BGC0000365_c1 |  |  |
| Cluster 5 | T3pks | 1524683 | 1565864 | BE-7585A_biosynthetic_gene_cluster (14% of genes show similarity) | BGC0000203_c1 |  |  |
| Cluster 6 | T3pks-T1pks | 1845066 | 1903193 | Streptomycin_biosynthetic_gene_cluster (12% of genes show similarity) | BGC0000717_c1 | 1 | CYP139A1 |
| Cluster 7 | T1pks | 2276864 | 2329319 | Reveromycin_biosynthetic_gene_cluster (15% of genes show similarity) | BGC0000135_c1 |  |  |
| Cluster 8 | Other | 2524087 | 2564956 | - | - | 3 | CYP124A1; CYP128A1; CYP121A1 |
| Cluster 9 | Nrps | 2635031 | 2694964 | Mycobactin_biosynthetic_gene_cluster (100% of genes show similarity) | BGC0001021_c1 |  |  |
| Cluster 10 | Other | 2894015 | 2937521 | - | - |  |  |
| Cluster 11 | T1pks-Nrps | 3219766 | 3314275 | Glycopeptidolipid_biosynthetic_gene_cluster (10% of genes show similarity) | BGC0000365_c1 |  |  |
| Cluster 12 | Terpene | 3794965 | 3815873 | Hopene_biosynthetic_gene_cluster (15% of genes show similarity) | BGC0000663_c1 |  |  |
| Cluster 13 | T1pks-Nrps | 4228268 | 4313889 | Glycopeptidolipid_biosynthetic_gene_cluster (10% of genes show similarity) | BGC0000365_c1 |  |  |
|  |  |  |  | ***Mycobacterium tuberculosis*strains CCDC5079** |  |  |  |
| Cluster 1 | Nrps | 85821 | 135158 | - | - |  |  |
| Cluster 2 | T1pks | 464327 | 507521 | Glycopeptidolipid_biosynthetic_gene_cluster (5% of genes show similarity) | BGC0000362_c1 |  |  |
| Cluster 3 | Bacteriocin | 882693 | 893490 | - | - |  |  |
| Cluster 4 | T1pks-Nrps | 1291321 | 1354813 | Glycopeptidolipid_biosynthetic_gene_cluster (13% of genes show similarity) | BGC0000365_c1 |  |  |
| Cluster 5 | T3pks | 1524046 | 1565227 | BE-7585A_biosynthetic_gene_cluster (14% of genes show similarity) | BGC0000203_c1 |  |  |
| Cluster 6 | T1pks | 1701368 | 1747631 | Glycopeptidolipid_biosynthetic_gene_cluster (20% of genes show similarity) | BGC0000365_c1 |  |  |
| Cluster 7 | T3pks-T1pks | 1844222 | 1902349 | Streptomycin_biosynthetic_gene_cluster (12% of genes show similarity) | BGC0000717_c1 | 1 | CYP139A1 |
| Cluster 8 | T1pks | 2267794 | 2314201 | Hygrocin_biosynthetic_gene_cluster (16% of genes show similarity) | BGC0000075_c1 | 3 | CYP124A1; CYP128A1; CYP121A1 |
| Cluster 9 | Other | 2513369 | 2554229 | - | - | 3 | CYP124A1; CYP128A1; CYP121A1 |
| Cluster 10 | Nrps | 2621067 | 2680998 | Mycobactin_biosynthetic_gene_cluster (90% of genes show similarity) | BGC0001021_c1 |  |  |
| Cluster 11 | Other | 2879665 | 2923171 | - | - |  |  |
| Cluster 12 | T1pks-Nrps | 3200387 | 3294904 | Cremimycin_biosynthetic_gene_cluster (20% of genes show similarity) | BGC0000042_c1 |  |  |
| Cluster 13 | Terpene | 3788074 | 3808982 | Hopene_biosynthetic_gene_cluster (15% of genes show similarity) | BGC0000663_c1 |  |  |
| Cluster 14 | T1pks-Nrps | 4223124 | 4308749 | Glycopeptidolipid_biosynthetic_gene_cluster (10% of genes show similarity) | BGC0000365_c1 |  |  |
|  |  |  |  | ***Mycobacterium tuberculosis* 7199-99** |  |  |  |
| Cluster 1 | Nrps | 88193 | 137576 | - | - |  |  |
| Cluster 2 | T1pks | 469987 | 514195 | Glycopeptidolipid_biosynthetic_gene_cluster (25% of genes show similarity) | BGC0000362_c1 |  |  |
| Cluster 3 | Bacteriocin | 889733 | 900530 | - | - |  |  |
| Cluster 4 | T1pks-Nrps | 1298263 | 1361753 | Glycopeptidolipid_biosynthetic_gene_cluster (10% of genes show similarity) | BGC0000365_c1 |  |  |
| Cluster 5 | T3pks | 1529729 | 1570910 | BE-7585A_biosynthetic_gene_cluster (14% of genes show similarity) | BGC0000203_c1 |  |  |
| Cluster 6 | T1pks | 1707228 | 1753554 | Glycopeptidolipid_biosynthetic_gene_cluster (20% of genes show similarity) | BGC0000365_c1 |  |  |
| Cluster 7 | T3pks-T1pks | 1850116 | 1909601 | Streptomycin_biosynthetic_gene_cluster (12% of genes show similarity) | BGC0000717_c1 | 1 | CYP139A1 |
| Cluster 8 | T1pks | 2283970 | 2336425 | Reveromycin_biosynthetic_gene_cluster (15% of genes show similarity) | BGC0000135_c1 |  |  |
| Cluster 9 | Nrps | 2639653 | 2699586 | Mycobactin_biosynthetic_gene_cluster (100% of genes show similarity) | BGC0001021_c1 |  |  |
| Cluster 10 | Other | 2898468 | 2941974 | - | - |  |  |
| Cluster 11 | T1pks-Nrps | 3223580 | 3318089 | Glycopeptidolipid_biosynthetic_gene_cluster (10% of genes show similarity) | BGC0000365_c1 |  |  |
| Cluster 12 | Terpene | 3801540 | 3822448 | Hopene_biosynthetic_gene_cluster (15% of genes show similarity) | BGC0000663_c1 |  |  |
| Cluster 13 | T1pks-Nrps | 4245655 | 4331276 | Glycopeptidolipid_biosynthetic_gene_cluster (10% of genes show similarity) | BGC0000365_c1 |  |  |
|  |  |  |  | ***Mycobacterium tuberculosis* Beijing/NITR203** |  |  |  |
| Cluster 1 | Nrps | 88147 | 137530 | - |  |  |  |
| Cluster 2 | T1pks | 467006 | 509918 | Rubradirin_biosynthetic_gene_cluster (3% of genes show similarity) | BGC0000141_c1 |  |  |
| Cluster 3 | Bacteriocin | 886390 | 897187 | Glycopeptidolipid_biosynthetic_gene_cluster (20% of genes show similarity) | BGC0000362_c1 |  |  |
| Cluster 4 | T1pks-Nrps | 1293600 | 1357090 | Glycopeptidolipid_biosynthetic_gene_cluster (10% of genes show similarity) | BGC0000365_c1 |  |  |
| Cluster 5 | T3pks | 1524696 | 1565877 | BE-7585A_biosynthetic_gene_cluster (14% of genes show similarity) | BGC0000203_c1 |  |  |
| Cluster 6 | T1pks | 1701947 | 1748273 | Glycopeptidolipid_biosynthetic_gene_cluster (20% of genes show similarity | BGC0000365_c1 |  |  |
| Cluster 7 | T3pks-T1pks | 1854013 | 1912140 | Streptomycin_biosynthetic_gene_cluster (12% of genes show similarity) | BGC0000717_c1 | 1 | CYP139A1 |
| Cluster 8 | Nrps | 1957816 | 2014457 | Glycopeptidolipid_biosynthetic_gene_cluster (20% of genes show similarity) | BGC0000362_c1 | 1 | CYP144A1 |
| Cluster 9 | T1pks | 2274341 | 2326796 | Reveromycin_biosynthetic_gene_cluster (15% of genes show similarity) | BGC0000135_c1 |  |  |
| Cluster 10 | Other | 2526666 | 2567535 | Glycopeptidolipid_biosynthetic_gene_cluster (20% of genes show similarity) | BGC0000362_c1 | 3 | CYP124A1; CYP128A1; CYP121A1 |
| Cluster 11 | Nrps | 2637478 | 2697396 | Mycobactin_biosynthetic_gene_cluster (100% of genes show similarity) | BGC0001021_c1 |  |  |
| Cluster 12 | Other | 2897602 | 2941108 | - |  |  |  |
| Cluster 13 | T1pks-Nrps | 3225133 | 3319641 | Glycopeptidolipid_biosynthetic_gene_cluster (10% of genes show similarity) | BGC0000365_c1 |  |  |
| Cluster 14 | Terpene | 3803713 | 3824621 | Hopene_biosynthetic_gene_cluster (15% of genes show similarity) | BGC0000663_c1 |  |  |
| Cluster 15 | T1pks-Nrps | 4235544 | 4321162 | Glycopeptidolipid_biosynthetic_gene_cluster (13% of genes show similarity) | BGC0000365_c1 |  |  |
|  |  |  |  | ***Mycobacterium tuberculosis*CAS/NITR204** |  |  |  |
| Cluster 1 | T1pks | 465362 | 506675 | Glycopeptidolipid_biosynthetic_gene_cluster (10% of genes show similarity) | BGC0000365_c1 |  |  |
| Cluster 2 | Bacteriocin | 883770 | 894567 | Glycopeptidolipid_biosynthetic_gene_cluster (20% of genes show similarity) | BGC0000362_c1 |  |  |
| Cluster 3 | T1pks-Nrps | 1289949 | 1353397 | Glycopeptidolipid_biosynthetic_gene_cluster (10% of genes show similarity) | BGC0000365_c1 |  |  |
| Cluster 4 | T3pks | 1520441 | 1561622 | BE-7585A_biosynthetic_gene_cluster (9% of genes show similarity) | BGC0000203_c1 |  |  |
| Cluster 5 | T3pks | 1848672 | 1889733 | Streptomycin_biosynthetic_gene_cluster (7% of genes show similarity) | BGC0000717_c1 | 1 | CYP139A1 |
| Cluster 6 | T1pks | 2271732 | 2316264 | Monensin_biosynthetic_gene_cluster (26% of genes show similarity) | BGC0000100_c1 |  |  |
| Cluster 7 | Other | 2519374 | 2560243 | Glycopeptidolipid_biosynthetic_gene_cluster (20% of genes show similarity) | BGC0000362_c1 | 2 | CYP124A1; CYP128A1 |
| Cluster 8 | Nrps | 2633125 | 2684309 | Mycobactin_biosynthetic_gene_cluster (40% of genes show similarity) | BGC0001021_c1 |  |  |
| Cluster 9 | Other | 2888395 | 2931373 | - | - |  |  |
| Cluster 10 | T1pks-Nrps | 3231109 | 3308740 | Epothilone_biosynthetic_gene_cluster (20% of genes show similarity) | BGC0000990_c1 |  |  |
| Cluster 11 | Terpene | 3788576 | 3809484 | Hopene_biosynthetic_gene_cluster (15% of genes show similarity) | BGC0000663_c1 |  |  |
| Cluster 12 | Nrps | 4245982 | 4303122 | Glycopeptidolipid_biosynthetic_gene_cluster (13% of genes show similarity) | BGC0000365_c1 |  |  |
|  |  |  |  | ***Mycobacterium tuberculosis* EAI5** |  |  |  |
| Cluster 1 | Nrps | 88158 | 137541 | - | - |  |  |
| Cluster 2 | T1pks | 466282 | 509950 | - | - |  |  |
| Cluster 3 | Bacteriocin | 885125 | 895922 | - | - |  |  |
| Cluster 4 | T1pks-Nrps | 1292378 | 1355868 | Glycopeptidolipid_biosynthetic_gene_cluster (10% of genes show similarity) | BGC0000365_c1 |  |  |
| Cluster 5 | T3pks | 1522123 | 1563304 | BE-7585A_biosynthetic_gene_cluster (14% of genes show similarity) | BGC0000203_c1 |  |  |
| Cluster 6 | T3pks-T1pks | 1852601 | 1909584 | Streptomycin_biosynthetic_gene_cluster (10% of genes show similarity) | BGC0000717_c1 | 1 | CYP139A1 |
| Cluster 7 | Nrps | 1955266 | 2010597 | - | - | 1 | CYP144A1 |
| Cluster 8 | Nrps | 2626850 | 2686783 | Mycobactin_biosynthetic_gene_cluster (100% of genes show similarity) | BGC0001021_c1 |  |  |
| Cluster 9 | Other | 2885657 | 2929163 | - | - |  |  |
| Cluster 10 | T1pks-Nrps | 3211874 | 3306382 | Glycopeptidolipid_biosynthetic_gene_cluster (6% of genes show similarity) | BGC0000365_c1 |  |  |
| Cluster 11 | Terpene | 3785088 | 3806008 | Hopene_biosynthetic_gene_cluster (15% of genes show similarity) | BGC0000663_c1 |  |  |
| Cluster 12 | T1pks-Nrps | 4215580 | 4301200 | Glycopeptidolipid_biosynthetic_gene_cluster (13% of genes show similarity) | BGC0000365_c1 |  |  |
|  |  |  |  | ***Mycobacterium tuberculosis*EAI5/NITR206** |  |  |  |
| Cluster 1 | Nrps | 88410 | 134359 | - |  |  |  |
| Cluster 2 | T1pks | 467755 | 509074 | Cremimycin_biosynthetic_gene_cluster (17% of genes show similarity) | BGC0000042_c1 |  |  |
| Cluster 3 | Bacteriocin | 888488 | 899285 | Glycopeptidolipid_biosynthetic_gene_cluster (20% of genes show similarity) | BGC0000362_c1 |  |  |
| Cluster 4 | Nrps | 1302990 | 1360183 | Glycopeptidolipid_biosynthetic_gene_cluster (13% of genes show similarity) | BGC0000365_c1 |  |  |
| Cluster 5 | T3pks | 1527541 | 1568029 | BE-7585A_biosynthetic_gene_cluster (11% of genes show similarity) | BGC0000203_c1 |  |  |
| Cluster 6 | T1pks | 1707794 | 1750760 | Glycopeptidolipid_biosynthetic_gene_cluster (20% of genes show similarity) | BGC0000365_c1 |  |  |
| Cluster 7 | T3pks | 1857101 | 1897934 | Streptomycin_biosynthetic_gene_cluster (7% of genes show similarity) | BGC0000717_c1 | 1 | CYP139A1 |
| Cluster 8 | T1pks | 2286023 | 2329235 | Reveromycin_biosynthetic_gene_cluster (15% of genes show similarity) | BGC0000135_c1 |  |  |
| Cluster 9 | Nrps | 2635271 | 2695268 | Mycobactin_biosynthetic_gene_cluster (80% of genes show similarity) | BGC0001021_c1 |  |  |
| Cluster 10 | Other | 3274152 | 3316209 | Piericidin_A1_biosynthetic_gene_cluster (50% of genes show similarity) | BGC0001169_c1 |  |  |
| Cluster 11 | Terpene | 3796074 | 3816985 | Hopene_biosynthetic_gene_cluster (15% of genes show similarity) | BGC0000663_c1 |  |  |
| Cluster 12 | T1pks | 4229489 | 4312569 | Glycopeptidolipid_biosynthetic_gene_cluster (6% of genes show similarity) | BGC0000365_c1 |  |  |
|  |  |  |  | ***Mycobacterium tuberculosis* Erdman=ATCC 35801** |  |  |  |
| Cluster 1 | Nrps | 87332 | 136670 | - |  |  |  |
| Cluster 2 | T1pks | 468741 | 511653 | Glycopeptidolipid_biosynthetic_gene_cluster (15% of genes show similarity) | BGC0000362_c1 |  |  |
| Cluster 3 | Bacteriocin | 885438 | 896235 | Glycopeptidolipid_biosynthetic_gene_cluster (20% of genes show similarity) | BGC0000362_c1 |  |  |
| Cluster 4 | T1pks-Nrps | 1292243 | 1355731 | Glycopeptidolipid_biosynthetic_gene_cluster (13% of genes show similarity) | BGC0000365_c1 |  |  |
| Cluster 5 | T3pks | 1518963 | 1560144 | BE-7585A_biosynthetic_gene_cluster (14% of genes show similarity) | BGC0000203_c1 |  |  |
| Cluster 6 | T1pks | 1694913 | 1741176 | Glycopeptidolipid_biosynthetic_gene_cluster (20% of genes show similarity) | BGC0000365_c1 |  |  |
| Cluster 7 | T3pks-T1pks | 1837801 | 1895928 | Streptomycin_biosynthetic_gene_cluster (12% of genes show similarity) | BGC0000717_c1 | 1 | CYP139A1 |
| Cluster 8 | T1pks | 2270605 | 2323060 | Reveromycin_biosynthetic_gene_cluster (15% of genes show similarity) | BGC0000135_c1 |  |  |
| Cluster 9 | Nrps | 2626627 | 2686560 | Mycobactin_biosynthetic_gene_cluster (100% of genes show similarity) | BGC0001021_c1 |  |  |
| Cluster 10 | Other | 2886112 | 2929618 | - | - |  |  |
| Cluster 11 | T1pks-Nrps | 3214209 | 3308717 | Glycopeptidolipid_biosynthetic_gene_cluster (10% of genes show similarity) | BGC0000365_c1 |  |  |
| Cluster 12 | Terpene | 3785902 | 3806810 | Hopene_biosynthetic_gene_cluster (15% of genes show similarity) | BGC0000663_c1 |  |  |
| Cluster 13 | T1pks-Nrps | 4216853 | 4302474 | Glycopeptidolipid_biosynthetic_gene_cluster (10% of genes show similarity) | BGC0000365_c1 |  |  |
|  |  |  |  | ***Mycobacterium tuberculosis* UT205** |  |  |  |
| Cluster 1 | Nrps | 88255 | 137638 | - | - |  |  |
| Cluster 2 | T1pks | 467045 | 511253 | - | - |  |  |
| Cluster 3 | Bacteriocin | 888533 | 899330 | - | - |  |  |
| Cluster 4 | T1pks-Nrps | 1296390 | 1359880 | Glycopeptidolipid_biosynthetic_gene_cluster (10% of genes show similarity) | BGC0000365_c1 |  |  |
| Cluster 5 | T3pks | 1527789 | 1568970 | BE-7585A_biosynthetic_gene_cluster (14% of genes show similarity) | BGC0000203_c1 |  |  |
| Cluster 6 | T1pks | 1705146 | 1751472 | Glycopeptidolipid_biosynthetic_gene_cluster (20% of genes show similarity) | BGC0000365_c1 |  |  |
| Cluster 7 | T3pks-T1pks | 1857379 | 1915506 | Streptomycin_biosynthetic_gene_cluster (12% of genes show similarity) | BGC0000717_c1 | 1 | CYP139A1 |
| Cluster 8 | Other | 2532570 | 2573439 | - | - | 2 | CYP124A1; CYP128A1 |
| Cluster 9 | Nrps | 2642431 | 2702364 | Mycobactin_biosynthetic_gene_cluster (100% of genes show similarity) | BGC0001021_c1 |  |  |
| Cluster 10 | Other | 2902700 | 2946206 | - | - |  |  |
| Cluster 11 | T1pks-Nrps | 3230570 | 3325079 | Glycopeptidolipid_biosynthetic_gene_cluster (10% of genes show similarity) | BGC0000365_c1 |  |  |
| Cluster 12 | Terpene | 3810044 | 3830952 | Hopene_biosynthetic_gene_cluster (15% of genes show similarity) | BGC0000663_c1 |  |  |
| Cluster 13 | T1pks-Nrps | 4242395 | 4328016 | Glycopeptidolipid_biosynthetic_gene_cluster (10% of genes show similarity) | BGC0000365_c1 |  |  |
|  |  |  |  | ***Mycobacterium canetti* CIPT 140010059** |  |  |  |
| Cluster 1 | Nrps | 91719 | 141102 | - | - |  |  |
| Cluster 2 | T1pks | 473020 | 520252 | Rubradirin_biosynthetic_gene_cluster (3% of genes show similarity) | BGC0000141_c1 |  |  |
| Cluster 3 | Bacteriocin | 894179 | 904976 | - | - |  |  |
| Cluster 4 | Nrps | 1320091 | 1377271 | Glycopeptidolipid_biosynthetic_gene_cluster (13% of genes show similarity) | BGC0000365_c1 |  |  |
| Cluster 5 | T3pks | 1548166 | 1589347 | BE-7585A_biosynthetic_gene_cluster (14% of genes show similarity) | BGC0000203_c1 |  |  |
| Cluster 6 | T1pks | 1726309 | 1780572 | Glycopeptidolipid_biosynthetic_gene_cluster (23% of genes show similarity) | BGC0000365_c1 |  |  |
| Cluster 7 | T3pks-T1pks | 1880898 | 1939026 | Streptomycin_biosynthetic_gene_cluster (12% of genes show similarity) | BGC0000717_c1 | 1 | CYP139A1 |
| Cluster 8 | T1pks | 2324305 | 2376769 | Reveromycin_biosynthetic_gene_cluster (15% of genes show similarity) | BGC0000135_c1 |  |  |
| Cluster 9 | Other | 2582707 | 2623576 | Glycopeptidolipid_biosynthetic_gene_cluster (20% of genes show similarity) | BGC0000362_c1 | 3 | CYP124A1; CYP128A1; CYP121A1 |
| Cluster 10 | Nrps | 2696313 | 2756246 | Mycobactin_biosynthetic_gene_cluster (100% of genes show similarity) | BGC0001021_c1 |  |  |
| Cluster 11 | Other | 2967557 | 3011063 | - | - |  |  |
| Cluster 12 | T1pks-Nrps | 3284106 | 3376575 | Glycopeptidolipid_biosynthetic_gene_cluster (13% of genes show similarity) | BGC0000365_c1 |  |  |
| Cluster 13 | Terpene | 3871405 | 3892313 | Hopene_biosynthetic_gene_cluster (15% of genes show similarity) | BGC0000663_c1 |  |  |
| Cluster 14 | T1pks-Nrps | 4303115 | 4388651 | Glycopeptidolipid_biosynthetic_gene_cluster (10% of genes show similarity) | BGC0000365_c1 |  |  |
|  |  |  |  | ***Mycobacterium canetti* CIPT 140060008** |  |  |  |
| Cluster 1 | Nrps | 93492 | 142875 | - | - |  |  |
| Cluster 2 | T1pks | 472069 | 516271 | - | - |  |  |
| Cluster 3 | Bacteriocin | 880676 | 891473 | - | - |  |  |
| Cluster 4 | T1pks-Nrps | 1289683 | 1353176 | Glycopeptidolipid_biosynthetic_gene_cluster (10% of genes show similarity) | BGC0000365_c1 |  |  |
| Cluster 5 | T3pks | 1525644 | 1566825 | BE-7585A_biosynthetic_gene_cluster (14% of genes show similarity) | BGC0000203_c1 |  |  |
| Cluster 6 | T1pks | 1706894 | 1753157 | Glycopeptidolipid_biosynthetic_gene_cluster (20% of genes show similarity) | BGC0000365_c1 |  |  |
| Cluster 7 | T3pks-T1pks | 1855101 | 1913229 | Streptomycin_biosynthetic_gene_cluster (12% of genes show similarity) | BGC0000717_c1 | 1 | CYP139A1 |
| Cluster 8 | T1pks | 2296179 | 2348634 | Reveromycin_biosynthetic_gene_cluster (15% of genes show similarity) | BGC0000135_c1 |  |  |
| Cluster 9 | Other | 2558631 | 2599491 | Glycopeptidolipid_biosynthetic_gene_cluster (20% of genes show similarity) | BGC0000362_c1 | 2 | CYP124A1; CYP128A1 |
| Cluster 10 | Nrps | 2667576 | 2727509 | Mycobactin_biosynthetic_gene_cluster (100% of genes show similarity) | BGC0001021_c1 |  |  |
| Cluster 11 | Other | 2935556 | 2979062 | - | - |  |  |
| Cluster 12 | T1pks-Nrps | 3248529 | 3340884 | Glycopeptidolipid_biosynthetic_gene_cluster (10% of genes show similarity) | BGC0000365_c1 |  |  |
| Cluster 13 | Terpene | 3831892 | 3852800 | Hopene_biosynthetic_gene_cluster (15% of genes show similarity) | BGC0000663_c1 |  |  |
| Cluster 14 | T1pks-Nrps | 4254850 | 4340471 | Glycopeptidolipid_biosynthetic_gene_cluster (10% of genes show similarity) | BGC0000365_c1 |  |  |
|  |  |  |  | ***Mycobacterium canetti* CIPT 140710010** |  |  |  |
| Cluster 1 | Nrps | 104157 | 153540 | - | - |  |  |
| Cluster 2 | T1pks | 478749 | 522951 | - | - |  |  |
| Cluster 3 | Bacteriocin | 900411 | 911208 | - | - |  |  |
| Cluster 4 | T1pks-Nrps | 1350891 | 1414367 | Glycopeptidolipid_biosynthetic_gene_cluster (10% of genes show similarity) | BGC0000365_c1 |  |  |
| Cluster 5 | T3pks | 1599811 | 1640992 | BE-7585A_biosynthetic_gene_cluster (14% of genes show similarity) | BGC0000203_c1 |  |  |
| Cluster 6 | T1pks | 1780717 | 1826980 | Glycopeptidolipid_biosynthetic_gene_cluster (20% of genes show similarity) | BGC0000365_c1 |  |  |
| Cluster 7 | T3pks-T1pks | 1927571 | 1985699 | Streptomycin_biosynthetic_gene_cluster (12% of genes show similarity) | BGC0000717_c1 | 1 | CYP139A1 |
| Cluster 8 | Nrps | 2039070 | 2098936 | Oxazolomycin_biosynthetic_gene_cluster (6% of genes show similarity) | BGC0001106_c1 | 1 | CYP144A1 |
| Cluster 9 | T1pks | 2391616 | 2444071 | Chartreusin_biosynthetic_gene_cluster (15% of genes show similarity) | BGC0000206_c2 |  |  |
| Cluster 10 | Other | 2642997 | 2683866 | - | - | 3 | CYP124A1; CYP128A; CYP121A1 |
| Cluster 11 | Nrps | 2758943 | 2818861 | Mycobactin_biosynthetic_gene_cluster (100% of genes show similarity) | BGC0001021_c1 |  |  |
| Cluster 12 | Other | 3030055 | 3073561 | - | - |  |  |
| Cluster 13 | T1pks-Nrps | 3349860 | 3445383 | Glycopeptidolipid_biosynthetic_gene_cluster (10% of genes show similarity) | BGC0000365_c1 |  |  |
| Cluster 14 | Terpene | 3918921 | 3939829 | Hopene_biosynthetic_gene_cluster (15% of genes show similarity) | BGC0000663_c1 |  |  |
| Cluster 15 | T1pks-Nrps | 4350509 | 4440905 | Glycopeptidolipid_biosynthetic_gene_cluster (13% of genes show similarity) | BGC0000365_c1 |  |  |
|  |  |  |  | ***Mycobacterium bovis*AF 2122/97** |  |  |  |
| Cluster 1 | Nrps | 88192 | 137575 | - | - |  |  |
| Cluster 2 | T1pks | 468118 | 510958 | Nigericin_biosynthetic_gene_cluster (33% of genes show similarity) | BGC0000114_c1 |  |  |
| Cluster 3 | Bacteriocin | 887302 | 898099 | - | - |  |  |
| Cluster 4 | T1pks-Nrps | 1295003 | 1358493 | Glycopeptidolipid_biosynthetic_gene_cluster (10% of genes show similarity) | BGC0000365_c1 |  |  |
| Cluster 5 | T3pks | 1525912 | 1567093 | BE-7585A_biosynthetic_gene_cluster (14% of genes show similarity) | BGC0000203_c1 |  |  |
| Cluster 6 | T1pks | 1690505 | 1736831 | Glycopeptidolipid_biosynthetic_gene_cluster (20% of genes show similarity) | BGC0000365_c1 |  |  |
| Cluster 7 | T3pks-T1pks | 1843955 | 1902082 | Streptomycin_biosynthetic_gene_cluster (12% of genes show similarity) | BGC0000717_c1 | 1 | CYP139A1 |
| Cluster 8 | Nrps | 1947776 | 1989374 | - | - |  |  |
| Cluster 9 | T1pks | 2262855 | 2315310 | Reveromycin_biosynthetic_gene_cluster (15% of genes show similarity) | BGC0000135_c1 |  |  |
| Cluster 10 | Other | 2510248 | 2551117 | - | - | 3 | CYP124A1; CYP128A1; CYP121A1 |
| Cluster 11 | Nrps | 2610182 | 2670115 | Mycobactin_biosynthetic_gene_cluster (100% of genes show similarity) | BGC0001021_c1 |  |  |
| Cluster 12 | Other | 2869730 | 2913236 | - | - |  |  |
| Cluster 13 | T1pks-Nrps | 3186430 | 3281054 | Glycopeptidolipid_biosynthetic_gene_cluster (6% of genes show similarity) | BGC0000365_c1 |  |  |
| Cluster 14 | Terpene | 3760840 | 3781748 | Hopene_biosynthetic_gene_cluster (15% of genes show similarity) | BGC0000663_c1 |  |  |
| Cluster 15 | T1pks-Nrps | 4176630 | 4262251 | Glycopeptidolipid_biosynthetic_gene_cluster (13% of genes show similarity) | BGC0000365_c1 |  |  |
|  |  |  |  | ***Mycobacterium bovis* BCG Pasteur 1173P2** |  |  |  |
| Cluster 1 | Nrps | 117898 | 167281 | - | - |  |  |
| Cluster 2 | T1pks | 497914 | 540754 | Nigericin_biosynthetic_gene_cluster (33% of genes show similarity) | BGC0000114_c1 |  |  |
| Cluster 3 | Bacteriocin | 916894 | 927691 | - | - |  |  |
| Cluster 4 | T1pks-Nrps | 1325009 | 1386951 | Glycopeptidolipid_biosynthetic_gene_cluster (10% of genes show similarity) | BGC0000365_c1 |  |  |
| Cluster 5 | T3pks | 1549982 | 1591163 | BE-7585A_biosynthetic_gene_cluster (14% of genes show similarity) | BGC0000203_c1 |  |  |
| Cluster 6 | T1pks | 1714424 | 1760750 | Glycopeptidolipid_biosynthetic_gene_cluster (20% of genes show similarity) | BGC0000365_c1 |  |  |
| Cluster 7 | T3pks-T1pks | 1859427 | 1917551 | Streptomycin_biosynthetic_gene_cluster (12% of genes show similarity) | BGC0000717_c1 | 1 | CYP139A1 |
| Cluster 8 | Nrps | 1963129 | 2004727 | - | - |  |  |
| Cluster 9 | T1pks | 2258183 | 2310638 | Reveromycin_biosynthetic_gene_cluster (15% of genes show similarity) | BGC0000135_c1 |  |  |
| Cluster 10 | Other | 2505335 | 2546204 | - | - | 3 | CYP124A1; CYP128A1; CYP121A1 |
| Cluster 11 | Nrps | 2602722 | 2662655 | Mycobactin_biosynthetic_gene_cluster (100% of genes show similarity) | BGC0001021_c1 |  |  |
| Cluster 12 | Other | 2862318 | 2905824 | - | - |  |  |
| Cluster 13 | T1pks-Nrps | 3179434 | 3274115 | Glycopeptidolipid_biosynthetic_gene_cluster (6% of genes show similarity) | BGC0000365_c1 |  |  |
| Cluster 14 | Terpene | 3790354 | 3811262 | Hopene_biosynthetic_gene_cluster (15% of genes show similarity) | BGC0000663_c1 |  |  |
| Cluster 15 | T1pks-Nrps | 4208348 | 4293968 | Glycopeptidolipid_biosynthetic_gene_cluster (10% of genes show similarity) | BGC0000365_c1 |  |  |
|  |  |  |  | ***Mycobacterium bovis* BCG Korea 1168P** |  |  |  |
| Cluster 1 | Nrps | 88231 | 137614 | - | - |  |  |
| Cluster 2 | T1pks | 468247 | 511087 | Nigericin_biosynthetic_gene_cluster (33% of genes show similarity) | BGC0000114_c1 |  |  |
| Cluster 3 | Bacteriocin | 887227 | 898024 | - | - |  |  |
| Cluster 4 | T1pks-Nrps | 1295342 | 1357284 | Glycopeptidolipid_biosynthetic_gene_cluster (10% of genes show similarity) | BGC0000365_c1 |  |  |
| Cluster 5 | T3pks | 1520315 | 1561496 | BE-7585A_biosynthetic_gene_cluster (14% of genes show similarity) | BGC0000203_c1 |  |  |
| Cluster 6 | T1pks | 1684653 | 1730979 | Glycopeptidolipid_biosynthetic_gene_cluster (20% of genes show similarity) | BGC0000365_c1 |  |  |
| Cluster 7 | T3pks-T1pks | 1829654 | 1887778 | Streptomycin_biosynthetic_gene_cluster (12% of genes show similarity) | BGC0000717_c1 | 1 | CYP139A1 |
| Cluster 8 | Nrps | 1933356 | 1974954 | - | - |  |  |
| Cluster 9 | T1pks | 2227828 | 2280283 | Reveromycin_biosynthetic_gene_cluster (15% of genes show similarity) | BGC0000135_c1 |  |  |
| Cluster 10 | Other | 2474980 | 2515849 | - | - | 3 | CYP124A1; CYP128A1; CYP121A1 |
| Cluster 11 | Nrps | 2572367 | 2632300 | Mycobactin_biosynthetic_gene_cluster (100% of genes show similarity) | BGC0001021_c1 |  |  |
| Cluster 12 | Other | 2831966 | 2875472 | - | - |  |  |
| Cluster 13 | T1pks-Nrps | 3149082 | 3243763 | Glycopeptidolipid_biosynthetic_gene_cluster (10% of genes show similarity) | BGC0000362_c1 |  |  |
| Cluster 14 | Terpene | 3795812 | 3816720 | Hopene_biosynthetic_gene_cluster (15% of genes show similarity) | BGC0000663_c1 |  |  |
| Cluster 15 | T1pks-Nrps | 4210537 | 4296157 | Glycopeptidolipid_biosynthetic_gene_cluster (10% of genes show similarity) | BGC0000365_c1 |  |  |
|  |  |  |  | ***Mycobacterium bovis* BCG Mexico** |  |  |  |
| Cluster 1 | Nrps | 88231 | 137614 | - | - |  |  |
| Cluster 2 | T1pks | 468248 | 511088 | Nigericin_biosynthetic_gene_cluster (33% of genes show similarity) | BGC0000114_c1 |  |  |
| Cluster 3 | Bacteriocin | 887173 | 897970 | - | - |  |  |
| Cluster 4 | T1pks-Nrps | 1295288 | 1357230 | Glycopeptidolipid_biosynthetic_gene_cluster (10% of genes show similarity) | BGC0000365_c1 |  |  |
| Cluster 5 | T3pks | 1520261 | 1561442 | BE-7585A_biosynthetic_gene_cluster (14% of genes show similarity) | BGC0000203_c1 |  |  |
| Cluster 6 | T1pks | 1684703 | 1731029 | Glycopeptidolipid_biosynthetic_gene_cluster (20% of genes show similarity) | BGC0000365_c1 |  |  |
| Cluster 7 | T3pks-T1pks | 1829706 | 1887830 | Streptomycin_biosynthetic_gene_cluster (12% of genes show similarity) | BGC0000717_c1 | 1 | CYP139A1 |
| Cluster 8 | Nrps | 1933408 | 1975006 | - | - |  |  |
| Cluster 9 | T1pks | 2237548 | 2290003 | Reveromycin_biosynthetic_gene_cluster (15% of genes show similarity) | BGC0000135_c1 |  |  |
| Cluster 10 | Other | 2484700 | 2525569 | - | - | 3 | CYP124A1; CYP128A1; CYP121A1 |
| Cluster 11 | Nrps | 2582087 | 2642020 | Mycobactin_biosynthetic_gene_cluster (100% of genes show similarity) | BGC0001021_c1 |  |  |
| Cluster 12 | Other | 2841683 | 2885189 | - | - |  |  |
| Cluster 13 | T1pks-Nrps | 3158799 | 3253480 | Glycopeptidolipid_biosynthetic_gene_cluster (6% of genes show similarity) | BGC0000365_c1 |  |  |
| Cluster 14 | Terpene | 3769719 | 3790627 | Hopene_biosynthetic_gene_cluster (15% of genes show similarity) | BGC0000663_c1 |  |  |
| Cluster 15 | T1pks-Nrps | 4187713 | 4272679 | Glycopeptidolipid_biosynthetic_gene_cluster (10% of genes show similarity) | BGC0000365_c1 |  |  |
|  |  |  |  | ***Mycobacterium bovis*BCG Toyko 172** |  |  |  |
| Cluster 1 | Nrps | 88231 | 137614 | - | - |  |  |
| Cluster 2 | T1pks | 468247 | 511087 | Nigericin_biosynthetic_gene_cluster (33% of genes show similarity) | BGC0000114_c1 |  |  |
| Cluster 3 | Bacteriocin | 888611 | 899408 | - | - |  |  |
| Cluster 4 | T1pks-Nrps | 1296347 | 1359837 | Glycopeptidolipid_biosynthetic_gene_cluster (10% of genes show similarity) | BGC0000365_c1 |  |  |
| Cluster 5 | T3pks | 1522869 | 1564050 | BE-7585A_biosynthetic_gene_cluster (14% of genes show similarity) | BGC0000203_c1 |  |  |
| Cluster 6 | T1pks | 1687317 | 1733643 | Glycopeptidolipid_biosynthetic_gene_cluster (20% of genes show similarity) | BGC0000365_c1 |  |  |
| Cluster 7 | T3pks-T1pks | 1832329 | 1890453 | Streptomycin_biosynthetic_gene_cluster (12% of genes show similarity) | BGC0000717_c1 | 1 | CYP139A1 |
| Cluster 8 | Nrps | 1936031 | 1977629 | - | - |  |  |
| Cluster 9 | T1pks | 2250961 | 2303416 | Reveromycin_biosynthetic_gene_cluster (15% of genes show similarity) | BGC0000135_c1 |  |  |
| Cluster 10 | Other | 2498107 | 2538976 | - | - | 3 | CYP124A1; CYP128A1; CYP121A1 |
| Cluster 11 | Nrps | 2595494 | 2655427 | Mycobactin_biosynthetic_gene_cluster (100% of genes show similarity) | BGC0001021_c1 |  |  |
| Cluster 12 | Other | 2855091 | 2898597 | - | - |  |  |
| Cluster 13 | T1pks-Nrps | 3172261 | 3266942 | Glycopeptidolipid_biosynthetic_gene_cluster (6% of genes show similarity) | BGC0000365_c1 |  |  |
| Cluster 14 | Terpene | 3788267 | 3809175 | Hopene_biosynthetic_gene_cluster (15% of genes show similarity) | BGC0000663_c1 |  |  |
| Cluster 15 | T1pks-Nrps | 4205541 | 4291158 | Glycopeptidolipid_biosynthetic_gene_cluster (10% of genes show similarity) | BGC0000365_c1 |  |  |
|  |  |  |  | ***Mycobacterium abscessus* ATCC 19977** |  |  |  |
| Cluster 1 | T1pks | 158481 | 203829 | - | - |  |  |
| Cluster 2 | Other | 487819 | 531847 | - | - |  |  |
| Cluster 3 | Other | 645339 | 689499 | Clavulanic_acid_biosynthetic_gene_cluster (8% of genes show similarity) | BGC0000845_c1 |  |  |
| Cluster 4 | Bacteriocin | 696411 | 707208 | - | - |  |  |
| Cluster 5 | T1pks-Nrps | 907298 | 966146 | Glycopeptidolipid_biosynthetic_gene_cluster (20% of genes show similarity) | BGC0000365_c1 | 1 | CYP1110B1 |
| Cluster 6 | T2pks | 2010675 | 2051931 | - | - | 1 | CYP153A |
| Cluster 7 | Nrps | 2101960 | 2154998 | Nocobactin_NA_biosynthetic_gene_cluster (75% of genes show similarity) | BGC0001027_c1 |  |  |
| Cluster 8 | Ectoine-T1pks-Nrps | 2210723 | 2332581 | Ectoine_biosynthetic_gene_cluster (100% of genes show similarity) | BGC0000853_c1 |  |  |
| Cluster 9 | Ectoine | 2625531 | 2635881 | Clavulanic_acid_biosynthetic_gene_cluster (8% of genes show similarity) | BGC0000845_c1 |  |  |
| Cluster 10 | Other | 2997425 | 3044572 | - | - |  |  |
| Cluster 11 | Bacteriocin | 3071596 | 3082492 | - | - |  |  |
| Cluster 12 | T1pks | 3168420 | 3214662 | Glycopeptidolipid_biosynthetic_gene_cluster (16% of genes show similarity) | BGC0000365_c1 |  |  |
| Cluster 13 | Nrps | 3335855 | 3383528 | - | - | 1 | CYP1128A |
| Cluster 14 | Other | 3398186 | 3441677 | Thiolactomycin_biosynthetic_gene_cluster (100% of genes show similarity) | BGC0001353_c2 |  |  |
| Cluster 15 | T1pks | 3598780 | 3641758 | RK-682_biosynthetic_gene_cluster (36% of genes show similarity) | BGC0000140_c1 |  |  |
| Cluster 16 | Nrps | 4122854 | 4180960 | Glycopeptidolipid_biosynthetic_gene_cluster (94% of genes show similarity) | BGC0000364_c1 |  |  |
| Cluster 17 | Bacteriocin | 4711818 | 4722678 | - | - |  |  |
| Cluster 18 | Nrps | 4729126 | 4847667 | Glycopeptidolipid_biosynthetic_gene_cluster (52% of genes show similarity) | BGC0000363_c1 | 1 | CYP135B |
|  |  |  |  | ***Mycobacterium abscessus*subsp. *bolletti*50594** |  |  |  |
| Cluster 1 | T1pks | 161195 | 206585 | - | - |  |  |
| Cluster 2 | Other | 504696 | 548724 | - | - |  |  |
| Cluster 3 | Other | 661435 | 705595 | Clavulanic_acid_biosynthetic_gene_cluster (8% of genes show similarity) | BGC0000845_c1 |  |  |
| Cluster 4 | Bacteriocin | 712494 | 723291 | Pactamycin_biosynthetic_gene_cluster (3% of genes show similarity) | BGC0000119_c1 |  |  |
| Cluster 5 | T1pks-Nrps | 922521 | 981375 | Glycopeptidolipid_biosynthetic_gene_cluster (20% of genes show similarity) | BGC0000365_c1 |  |  |
| Cluster 6 | T2pks-Nrps | 2020698 | 2103922 | Nocobactin_NA_biosynthetic_gene_cluster (75% of genes show similarity) | BGC0001027_c1 |  |  |
| Cluster 7 | Ectoine-T1pks-Nrps | 2166118 | 2265937 | Ectoine_biosynthetic_gene_cluster (100% of genes show similarity) | BGC0000853_c1 |  |  |
| Cluster 8 | Ectoine | 2588452 | 2598802 | - | - |  |  |
| Cluster 9 | Other | 2932894 | 2980050 | - | - |  |  |
| Cluster 10 | Bacteriocin | 3006899 | 3017795 | - | - |  |  |
| Cluster 11 | T1pks | 3103495 | 3149776 | Glycopeptidolipid_biosynthetic_gene_cluster (13% of genes show similarity) | BGC0000365_c1 |  |  |
| Cluster 12 | Nrps | 3266186 | 3313859 | Lomaiviticin_biosynthetic_gene_cluster (3% of genes show similarity) | BGC0000240_c1 | 1 | CYP1128A |
| Cluster 13 | Other | 3329761 | 3373252 | Thiolactomycin_biosynthetic_gene_cluster (100% of genes show similarity) | BGC0001353_c2 |  |  |
| Cluster 14 | Bacteriocin | 4676174 | 4687034 | - | - |  |  |
| Cluster 15 | Nrps | 4693688 | 4812188 | Glycopeptidolipid_biosynthetic_gene_cluster (52% of genes show similarity) | BGC0000363_c1 | 1 | CYP135B |
|  |  |  |  | ***Mycobacterium abscessus* 47J26** |  |  |  |
| Cluster 1 | T1pks | 158293 | 203647 | - | - |  |  |
| Cluster 2 | Other | 477159 | 521187 | - | - |  |  |
| Cluster 3 | Other | 634852 | 679012 | Clavulanic_acid_biosynthetic_gene_cluster (8% of genes show similarity) | BGC0000845_c1 |  |  |
| Cluster 4 | Bacteriocin | 686966 | 697763 | Pactamycin_biosynthetic_gene_cluster (3% of genes show similarity) | BGC0000119_c1 |  |  |
| Cluster 5 | T1pks-Nrps | 824318 | 883172 | Glycopeptidolipid_biosynthetic_gene_cluster (20% of genes show similarity) | BGC0000365_c1 |  |  |
| Cluster 6 | T2pks-Nrps | 2006372 | 2089393 | Nocobactin_NA_biosynthetic_gene_cluster (75% of genes show similarity) | BGC0001027_c1 |  |  |
| Cluster 7 | Ectoine-T1pks-Nrps | 2150144 | 2250498 | Ectoine_biosynthetic_gene_cluster (100% of genes show similarity) | BGC0000853_c1 |  |  |
| Cluster 8 | Ectoine | 2569896 | 2580150 | Clavulanic_acid_biosynthetic_gene_cluster (8% of genes show similarity) | BGC0000845_c1 |  |  |
| Cluster 9 | Other | 2963708 | 3010807 | - | - |  |  |
| Cluster 10 | Bacteriocin | 3039563 | 3050459 | - | - |  |  |
| Cluster 11 | T1pks | 3133453 | 3179734 | Glycopeptidolipid_biosynthetic_gene_cluster (13% of genes show similarity) | BGC0000365_c1 |  |  |
| Cluster 12 | Nrps | 3295071 | 3342744 | Lomaiviticin_biosynthetic_gene_cluster (3% of genes show similarity) | BGC0000240_c1 | 1 | CYP1128A |
| Cluster 13 | Other | 3356857 | 3400348 | Thiolactomycin_biosynthetic_gene_cluster (100% of genes show similarity) | BGC0001353_c2 |  |  |
| Cluster 14 | Nrps | 4139758 | 4197864 | Glycopeptidolipid_biosynthetic_gene_cluster (94% of genes show similarity) | BGC0000364_c1 |  |  |
| Cluster 15 | Bacteriocin | 4688809 | 4699606 | - | - |  |  |
| Cluster 16 | Nrps | 4706323 | 4826135 | Glycopeptidolipid_biosynthetic_gene_cluster (47% of genes show similarity) | BGC0000364_c1 | 1 | CYP135B |
|  |  |  |  | ***Mycobacterium abscessus* 103** |  |  |  |
| Cluster 1 | T1pks | 158484 | 203832 | - | - |  |  |
| Cluster 2 | Other | 472620 | 516648 | - | - |  |  |
| Cluster 3 | Other | 630140 | 674300 | Clavulanic_acid_biosynthetic_gene_cluster (8% of genes show similarity) | BGC0000845_c1 |  |  |
| Cluster 4 | Bacteriocin | 681212 | 692009 | - | - |  |  |
| Cluster 5 | T1pks-Nrps | 891844 | 950692 | Glycopeptidolipid_biosynthetic_gene_cluster (20% of genes show similarity) | BGC0000365_c1 | 1 | CYP1110B1 |
| Cluster 6 | T2pks | 1995058 | 2036314 | - | - | 1 | CYP153A |
| Cluster 7 | Nrps | 2086343 | 2139381 | Nocobactin_NA_biosynthetic_gene_cluster (75% of genes show similarity) | BGC0001027_c1 |  |  |
| Cluster 8 | Ectoine-T1pks-Nrps | 2195106 | 2316966 | Ectoine_biosynthetic_gene_cluster (100% of genes show similarity) | BGC0000853_c1 |  |  |
| Cluster 9 | Ectoine | 2609951 | 2620205 | Clavulanic_acid_biosynthetic_gene_cluster (8% of genes show similarity) | BGC0000845_c1 |  |  |
| Cluster 10 | Other | 2981734 | 3028881 | - | - |  |  |
| Cluster 11 | Bacteriocin | 3055905 | 3066801 | - | - |  |  |
| Cluster 12 | T1pks | 3152718 | 3198999 | Glycopeptidolipid_biosynthetic_gene_cluster (16% of genes show similarity) | BGC0000365_c1 |  |  |
| Cluster 13 | Nrps | 3320153 | 3367826 | - | - | 1 | CYP1128A |
| Cluster 14 | Other | 3382484 | 3425975 | Thiolactomycin_biosynthetic_gene_cluster (100% of genes show similarity) | BGC0001353_c2 |  |  |
| Cluster 15 | T1pks | 3583078 | 3626056 | RK-682_biosynthetic_gene_cluster (36% of genes show similarity) | BGC0000140_c1 |  |  |
| Cluster 16 | Nrps | 4107149 | 4163992 | Glycopeptidolipid_biosynthetic_gene_cluster (94% of genes show similarity) | BGC0000364_c1 |  |  |
| Cluster 17 | Bacteriocin | 4696087 | 4706884 | - | - |  |  |
| Cluster 18 | Nrps | 4713384 | 4831926 | Glycopeptidolipid_biosynthetic_gene_cluster (52% of genes show similarity) | BGC0000363_c1 | 1 | CYP135B |
|  |  |  |  | ***Mycobacterium abscessus*subsp. *bolletti*MA 1948** |  |  |  |
| Cluster 1 | T1pks | 158459 | 203807 | - | - |  |  |
| Cluster 2 | Other | 487797 | 531825 | - | - |  |  |
| Cluster 3 | Other | 645317 | 689477 | Clavulanic_acid_biosynthetic_gene_cluster (8% of genes show similarity) | BGC0000845_c1 |  |  |
| Cluster 4 | Bacteriocin | 696389 | 707186 | - | - |  |  |
| Cluster 5 | T1pks-Nrps | 907074 | 965922 | Glycopeptidolipid_biosynthetic_gene_cluster (20% of genes show similarity) | BGC0000365_c1 | 1 | CYP1110B1 |
| Cluster 6 | T2pks | 2010031 | 2051287 | - | - | 1 | CYP153A |
| Cluster 7 | Nrps | 2101316 | 2154354 | Nocobactin_NA_biosynthetic_gene_cluster (75% of genes show similarity) | BGC0001027_c1 |  |  |
| Cluster 8 | Ectoine-T1pks-Nrps | 2210079 | 2331923 | Ectoine_biosynthetic_gene_cluster (100% of genes show similarity) | BGC0000853_c1 |  |  |
| Cluster 9 | Ectoine | 2624826 | 2635080 | Clavulanic_acid_biosynthetic_gene_cluster (8% of genes show similarity) | BGC0000845_c1 |  |  |
| Cluster 10 | Other | 2996595 | 3043742 | - | - |  |  |
| Cluster 11 | Bacteriocin | 3070767 | 3081663 | - | - |  |  |
| Cluster 12 | T1pks | 3167591 | 3213872 | Glycopeptidolipid_biosynthetic_gene_cluster (16% of genes show similarity) | BGC0000365_c1 |  |  |
| Cluster 13 | Nrps | 3335032 | 3382705 | - | - | 1 | CYP1128A |
| Cluster 14 | Other | 3397363 | 3440854 | Thiolactomycin_biosynthetic_gene_cluster (100% of genes show similarity) | BGC0001353_c2 |  |  |
| Cluster 15 | T1pks | 3597915 | 3640893 | RK-682_biosynthetic_gene_cluster (36% of genes show similarity) | BGC0000140_c1 |  |  |
| Cluster 16 | Nrps | 4127741 | 4176842 | Glycopeptidolipid_biosynthetic_gene_cluster (94% of genes show similarity) | BGC0000364_c1 |  |  |
| Cluster 17 | Bacteriocin | 4708919 | 4719716 | - | - |  |  |
| Cluster 18 | Nrps | 4726200 | 4844741 | Glycopeptidolipid_biosynthetic_gene_cluster (52% of genes show similarity) | BGC0000363_c1 | 1 | CYP135B |
|  |  |  |  | ***Mycobacterium abscessus* VO6705** |  |  |  |
| Cluster 1 | Nrps | 144035 | 244947 | Glycopeptidolipid_biosynthetic_gene_cluster (52% of genes show similarity) | BGC0000363_c1 | 1 | CYP135B |
| Cluster 2 | Bacteriocin | 269250 | 280047 | - | - |  |  |
| Cluster 3 | Nrps | 770368 | 827211 | Glycopeptidolipid_biosynthetic_gene_cluster (94% of genes show similarity) | BGC0000364_c1 |  |  |
| Cluster 4 | Other | 1483184 | 1526675 | Thiolactomycin_biosynthetic_gene_cluster (100% of genes show similarity) | BGC0001353_c2 |  |  |
| Cluster 5 | Nrps | 1548800 | 1596473 | - | - | 1 | CYP1128A |
| Cluster 6 | T1pks | 1726616 | 1772897 | Glycopeptidolipid_biosynthetic_gene_cluster (13% of genes show similarity) | BGC0000365_c1 |  |  |
| Cluster 7 | Bacteriocin | 1858614 | 1869510 | - | - |  |  |
| Cluster 8 | Other | 1898270 | 1941794 | - | - |  |  |
| Cluster 9 | Ectoine | 2315260 | 2325514 | - | - |  |  |
| Cluster 10 | T1pks-Ectoine-Nrps | 2623115 | 2722933 | Ectoine_biosynthetic_gene_cluster (100% of genes show similarity) | BGC0000853_c1 |  |  |
| Cluster 11 | T2pks-Nrps | 2783626 | 2866813 | Nocobactin_NA_biosynthetic_gene_cluster (75% of genes show similarity) | BGC0001027_c1 |  |  |
| Cluster 12 | T1pks-Nrps | 3801727 | 3860575 | Glycopeptidolipid_biosynthetic_gene_cluster (20% of genes show similarity) | BGC0000365_c1 |  |  |
| Cluster 13 | Bacteriocin | 3997591 | 4008388 | Pactamycin_biosynthetic_gene_cluster (3% of genes show similarity) | BGC0000119_c1 |  |  |
| Cluster 14 | Other | 4172247 | 4216275 | - | - |  |  |
| Cluster 15 | T1pks | 4478252 | 4523642 | - | - |  |  |
|  |  |  |  | ***Mycobacterium avium* 104** |  |  |  |
| Cluster 1 | T1pks | 190988 | 236363 | - | - |  |  |
| Cluster 2 | Bacteriocin | 707909 | 718706 | - | - |  |  |
| Cluster 3 | T3pks-T1pks | 1207060 | 1293496 | Kanamycin_biosynthetic_gene_cluster (2% of genes show similarity) | BGC0000703_c1 |  |  |
| Cluster 4 | T1pks-Nrps | 1718145 | 1777594 | Glycopeptidolipid_biosynthetic_gene_cluster (26% of genes show similarity) | BGC0000365_c1 |  |  |
| Cluster 5 | Nrps | 1966429 | 2033194 | Mycobactin_biosynthetic_gene_cluster (80% of genes show similarity) | BGC0001021_c1 | 1 | CYP187A |
| Cluster 6 | T1pks | 2363595 | 2409891 | - | - | 1 | CYP150A |
| Cluster 7 | T1pks | 2454065 | 2506580 | Herbimycin_biosynthetic_gene_cluster (10% of genes show similarity) | BGC0000074_c1 | 1 | CYP105NSF1 |
| Cluster 8 | Nrps | 3073635 | 3144900 | - | - |  |  |
| Cluster 9 | T3pks-T1pks | 3153360 | 3211590 | Streptomycin_biosynthetic_gene_cluster (14% of genes show similarity) | BGC0000717_c1 | 1 | CYP139A |
| Cluster 10 | Nrps | 3299015 | 3402918 | Glycopeptidolipid_biosynthetic_gene_cluster (69% of genes show similarity) | BGC0000362_c1 |  |  |
| Cluster 11 | Other | 3599656 | 3643177 | - | - |  |  |
| Cluster 12 | Terpene | 4007133 | 4028083 | Isorenieratene_biosynthetic_gene_cluster (71% of genes show similarity) | BGC0000664_c1 |  |  |
| Cluster 13 | Other | 4224404 | 4267655 | Glycopeptidolipid_biosynthetic_gene_cluster (7% of genes show similarity) | BGC0000362_c1 |  |  |
| Cluster 14 | Nrps | 4995861 | 5046139 | - | - |  |  |
|  |  |  |  | ***Mycobacterium avium*subsp. *paratuberculosis*K10** |  |  |  |
| Cluster 1 | T1pks | 208518 | 253842 | - | - |  |  |
| Cluster 2 | Bacteriocin | 648470 | 659267 | - | - |  |  |
| Cluster 3 | Other | 1064778 | 1108299 | - | - |  |  |
| Cluster 4 | Nrps | 1289241 | 1341324 | Glycopeptidolipid_biosynthetic_gene_cluster (35% of genes show similarity) | BGC0000362_c1 |  |  |
| Cluster 5 | T3pks-T1pks | 1452154 | 1504179 | Streptomycin_biosynthetic_gene_cluster (14% of genes show similarity) | BGC0000717_c1 | 1 | CYP139A |
| Cluster 6 | Nrps | 1512373 | 1571527 | Telomycin_biosynthetic_gene_cluster (8% of genes show similarity) | BGC0001406_c1 |  |  |
| Cluster 7 | Nrps | 1834059 | 1888514 | - | - |  |  |
| Cluster 8 | T1pks | 1939801 | 1992313 | Herbimycin_biosynthetic_gene_cluster (10% of genes show similarity) | BGC0000074_c1 | 1 | CYP105NSF1 |
| Cluster 9 | T1pks-Nrps | 2024496 | 2083965 | Glycopeptidolipid_biosynthetic_gene_cluster (13% of genes show similarity) | BGC0000365_c1 | 1 | CYP150A |
| Cluster 10 | Nrps | 2382034 | 2451169 | Mycobactin_biosynthetic_gene_cluster (100% of genes show similarity) | BGC0001021_c1 | 1 | CYP1034A |
| Cluster 11 | T1pks-Nrps | 2459656 | 2519101 | Glycopeptidolipid_biosynthetic_gene_cluster (26% of genes show similarity) | BGC0000365_c1 |  |  |
| Cluster 12 | T1pks | 2913453 | 2955744 | Marinacarboline_biosynthetic_gene_cluster (23% of genes show similarity) | BGC0001137_c1 |  |  |
| Cluster 13 | T3pks | 2955877 | 2996926 | Alkylresorcinol_biosynthetic_gene_cluster (100% of genes show similarity) | BGC0000282_c1 | 1 | CYP187A |
| Cluster 14 | Terpene | 3407499 | 3428230 | Isorenieratene_biosynthetic_gene_cluster (71% of genes show similarity) | BGC0000664_c1 |  |  |
| Cluster 15 | Other | 3624143 | 3667490 | - | - |  |  |
| Cluster 16 | T1pks-Nrps | 4111530 | 4228843 | Glycopeptidolipid_biosynthetic_gene_cluster (26% of genes show similarity) | BGC0000365_c1 |  |  |
|  |  |  |  | ***Mycobacterium avium* subsp. *paratuberculosis*MAP4** |  |  |  |
| Cluster 1 | Nrps | 1 | 99437 | Glycopeptidolipid_biosynthetic_gene_cluster (15% of genes show similarity) | BGC0000362_c1 |  |  |
| Cluster 2 | Other | 543572 | 586823 | - | - |  |  |
| Cluster 3 | Terpene | 782734 | 803684 | Isorenieratene_biosynthetic_gene_cluster (71% of genes show similarity) | BGC0000664_c1 |  |  |
| Cluster 4 | T3pks | 1213604 | 1254653 | Alkylresorcinol_biosynthetic_gene_cluster (100% of genes show similarity) | BGC0000282_c1 | 1 | CYP187A |
| Cluster 5 | T1pks | 1254780 | 1297071 | Marinacarboline_biosynthetic_gene_cluster (23% of genes show similarity) | BGC0001137_c1 |  |  |
| Cluster 6 | T1pks-Nrps | 1691452 | 1750897 | Glycopeptidolipid_biosynthetic_gene_cluster (26% of genes show similarity) | BGC0000365_c1 |  |  |
| Cluster 7 | Nrps | 1757972 | 1827107 | Mycobactin_biosynthetic_gene_cluster (100% of genes show similarity) | BGC0001021_c1 | 1 | CYP1034A |
| Cluster 8 | T1pks-Nrps | 2125298 | 2180594 | Glycopeptidolipid_biosynthetic_gene_cluster (15% of genes show similarity) | BGC0000363_c1 | 1 | CYP150A |
| Cluster 9 | T1pks | 2216949 | 2269461 | Herbimycin_biosynthetic_gene_cluster (10% of genes show similarity) | BGC0000074_c1 | 1 | CYP105NSF1 |
| Cluster 10 | Nrps | 2320747 | 2375201 | - | - |  |  |
| Cluster 11 | Nrps | 2637717 | 2696871 | Telomycin_biosynthetic_gene_cluster (8% of genes show similarity) | BGC0001406_c1 |  |  |
| Cluster 12 | T3pks-T1pks | 2705065 | 2757090 | Streptomycin_biosynthetic_gene_cluster (14% of genes show similarity) | BGC0000717_c1 | 1 | CYP139A |
| Cluster 13 | Nrps | 2846485 | 2919999 | Glycopeptidolipid_biosynthetic_gene_cluster (35% of genes show similarity) | BGC0000362_c1 |  |  |
| Cluster 14 | Other | 3100942 | 3144463 | - | - |  |  |
| Cluster 15 | Bacteriocin | 3549953 | 3560750 | - | - |  |  |
| Cluster 16 | T1pks | 3955383 | 4000758 | - | - |  |  |
| Cluster 17 | T1pks | 4182204 | 4228500 | Glycopeptidolipid_biosynthetic_gene_cluster (15% of genes show similarity) | BGC0000362_c1 | 1 | CYP144A |
|  |  |  |  | ***Mycobacterium intracellulare* ATCC 13950** |  |  |  |
| Cluster 1 | T1pks | 205651 | 250972 | - | - |  |  |
| Cluster 2 | Bacteriocin | 635320 | 646117 | - | - |  |  |
| Cluster 3 | Nrps | 1107358 | 1150645 | Glycopeptidolipid_biosynthetic_gene_cluster (15% of genes show similarity) | BGC0000362_c1 |  |  |
| Cluster 4 | T3pks | 1164439 | 1205461 | Alkylresorcinol_biosynthetic_gene_cluster (100% of genes show similarity) | BGC0000282_c1 | 1 | CYP187A |
| Cluster 5 | T1pks | 1205576 | 1251938 | Marinacarboline_biosynthetic_gene_cluster (23% of genes show similarity) | BGC0001137_c1 |  |  |
| Cluster 6 | T1pks-Nrps | 1873947 | 1932779 | Glycopeptidolipid_biosynthetic_gene_cluster (23% of genes show similarity) | BGC0000365_c1 |  |  |
| Cluster 7 | Nrps | 2038653 | 2111736 | Mycobactin_biosynthetic_gene_cluster (80% of genes show similarity) | BGC0001021_c1 |  |  |
| Cluster 8 | T1pks-Nrps | 2437934 | 2503761 | Glycopeptidolipid_biosynthetic_gene_cluster (6% of genes show similarity) | BGC0000365_c1 | 1 | CYP150A |
| Cluster 9 | T1pks | 2539388 | 2591924 | FD-891_biosynthetic_gene_cluster (50% of genes show similarity) | BGC0000058_c1 | 2 | CYP279A; CYP105U |
| Cluster 10 | Nrps | 2996715 | 3067965 | - | - |  |  |
| Cluster 11 | T3pks-T1pks | 3082075 | 3140357 | Streptomycin_biosynthetic_gene_cluster (14% of genes show similarity) | BGC0000717_c1 |  |  |
| Cluster 12 | Nrps | 3231290 | 3336183 | Glycopeptidolipid_biosynthetic_gene_cluster (33% of genes show similarity) | BGC0000362_c1 |  |  |
| Cluster 13 | Nrps | 3347222 | 3397502 | Glycopeptidolipid_biosynthetic_gene_cluster (25% of genes show similarity) | BGC0000362_c1 |  |  |
| Cluster 14 | Other | 3535808 | 3579332 | - | - |  |  |
| Cluster 15 | Terpene | 3940114 | 3961067 | Isorenieratene_biosynthetic_gene_cluster (71% of genes show similarity) | BGC0000664_c1 |  |  |
| Cluster 16 | Other | 4161097 | 4204345 | - | - | 1 | CYP1122A1 |
| Cluster 17 | Other | 4702833 | 4746399 | Heme_D1_biosynthetic_gene_cluster (11% of genes show similarity) | BGC0000905_c1 |  |  |
| Cluster 18 | Nrps | 4997133 | 5047399 | - | - |  |  |
| Cluster 19 | Other | 5234552 | 5278040 | - | - |  |  |
|  |  |  |  | ***Mycobacterium intracellulare*MOTT-02** |  |  |  |
| Cluster 1 | T1pks | 202276 | 247597 | - | - |  |  |
| Cluster 2 | Bacteriocin | 631683 | 642480 | - | - |  |  |
| Cluster 3 | Nrps | 1104554 | 1147841 | Glycopeptidolipid_biosynthetic_gene_cluster (15% of genes show similarity) | BGC0000362_c1 |  |  |
| Cluster 4 | T3pks | 1165970 | 1207088 | Alkylresorcinol_biosynthetic_gene_cluster (100% of genes show similarity) | BGC0000282_c1 | 2 | CYP187A; CYP150A |
| Cluster 5 | T1pks | 1207102 | 1253464 | Marinacarboline_biosynthetic_gene_cluster (23% of genes show similarity) | BGC0001137_c1 |  |  |
| Cluster 6 | T1pks-Nrps | 1839531 | 1898363 | Glycopeptidolipid_biosynthetic_gene_cluster (23% of genes show similarity) | BGC0000365_c1 |  |  |
| Cluster 7 | Nrps | 2015556 | 2088616 | Mycobactin_biosynthetic_gene_cluster (80% of genes show similarity) | BGC0001021_c1 |  |  |
| Cluster 8 | T1pks-Nrps | 2442610 | 2508437 | Glycopeptidolipid_biosynthetic_gene_cluster (6% of genes show similarity) | BGC0000365_c1 | 1 | CYP150A |
| Cluster 9 | T1pks | 2544113 | 2596649 | FD-891_biosynthetic_gene_cluster (50% of genes show similarity) | BGC0000058_c1 | 2 | CYP279A; CYP105U |
| Cluster 10 | Nrps | 3004717 | 3075967 | - | - |  |  |
| Cluster 11 | T3pks-T1pks | 3086027 | 3144309 | Streptomycin_biosynthetic_gene_cluster (14% of genes show similarity) | BGC0000717_c1 |  |  |
| Cluster 12 | Nrps | 3235279 | 3340169 | Glycopeptidolipid_biosynthetic_gene_cluster (33% of genes show similarity) | BGC0000362_c1 |  |  |
| Cluster 13 | Nrps | 3351211 | 3401491 | Glycopeptidolipid_biosynthetic_gene_cluster (25% of genes show similarity) | BGC0000362_c1 |  |  |
| Cluster 14 | Other | 3539706 | 3583230 | - | - |  |  |
| Cluster 15 | Terpene | 3933994 | 3954947 | Isorenieratene_biosynthetic_gene_cluster (71% of genes show similarity) | BGC0000664_c1 |  |  |
| Cluster 16 | Other | 4160267 | 4203515 | - | - |  |  |
| Cluster 17 | Other | 4727192 | 4770758 | Heme_D1_biosynthetic_gene_cluster (11% of genes show similarity) | BGC0000905_c1 | 1 | CYP1128A |
| Cluster 18 | Nrps | 5004974 | 5055243 | - | - |  |  |
| Cluster 19 | Other | 5242614 | 5286102 | - | - |  |  |
|  |  |  |  | ***Mycobacterium intracellulare* MOTT-64** |  |  |  |
| Cluster 1 | T1pks | 204915 | 250227 | - | - |  |  |
| Cluster 2 | Bacteriocin | 632658 | 643455 | - | - |  |  |
| Cluster 3 | T3pks-T1pks | 1130404 | 1217633 | Kanamycin_biosynthetic_gene_cluster (2% of genes show similarity) | BGC0000703_c1 | 1 | CYP187A |
| Cluster 4 | T1pks-Nrps | 1775427 | 1834259 | Glycopeptidolipid_biosynthetic_gene_cluster (23% of genes show similarity) | BGC0000365_c1 |  |  |
| Cluster 5 | Nrps | 1931020 | 2004097 | Mycobactin_biosynthetic_gene_cluster (80% of genes show similarity) | BGC0001021_c1 |  |  |
| Cluster 6 | T1pks | 2329063 | 2375413 | Glycopeptidolipid_biosynthetic_gene_cluster (6% of genes show similarity) | BGC0000365_c1 | 1 | CYP150A |
| Cluster 7 | T1pks | 2411044 | 2463577 | Geldanamycin_biosynthetic_gene_cluster (13% of genes show similarity) | BGC0000066_c1 | 2 | CYP279A; CYP105U |
| Cluster 8 | Nrps | 2868444 | 2939694 | - | - |  |  |
| Cluster 9 | T3pks-T1pks | 2948713 | 3006995 | Streptomycin_biosynthetic_gene_cluster (14% of genes show similarity) | BGC0000717_c1 |  |  |
| Cluster 10 | Nrps | 3144860 | 3202786 | Glycopeptidolipid_biosynthetic_gene_cluster (46% of genes show similarity) | BGC0000362_c1 |  |  |
| Cluster 11 | Other | 3371314 | 3414838 | - | - |  |  |
| Cluster 12 | Terpene | 3763092 | 3784045 | Isorenieratene_biosynthetic_gene_cluster (57% of genes show similarity) | BGC0000664_c1 |  |  |
| Cluster 13 | Other | 3975840 | 4019088 | - | - |  |  |
| Cluster 14 | Other | 4511862 | 4555428 | Heme_D1_biosynthetic_gene_cluster (11% of genes show similarity) | BGC0000905_c1 |  |  |
| Cluster 15 | Nrps | 4780724 | 4830977 | - | - |  |  |
| Cluster 16 | Other | 5017137 | 5060703 | - | - |  |  |
|  |  |  |  | ***Mycobacterium intracellulare* MOTT-36Y** |  |  |  |
| Cluster 1 | T1pks | 199992 | 245304 | - | - |  |  |
| Cluster 2 | Bacteriocin | 629625 | 640422 | - | - |  |  |
| Cluster 3 | Nrps | 1105073 | 1148354 | Glycopeptidolipid_biosynthetic_gene_cluster (15% of genes show similarity) | BGC0000362_c1 |  |  |
| Cluster 4 | T3pks | 1164196 | 1205314 | Alkylresorcinol_biosynthetic_gene_cluster (100% of genes show similarity) | BGC0000282_c1 | 1 | CYP187A |
| Cluster 5 | T1pks | 1205322 | 1251708 | Marinacarboline_biosynthetic_gene_cluster (23% of genes show similarity) | BGC0001137_c1 |  |  |
| Cluster 6 | T1pks-Nrps | 1612829 | 1671661 | Glycopeptidolipid_biosynthetic_gene_cluster (23% of genes show similarity) | BGC0000365_c1 |  |  |
| Cluster 7 | Nrps | 1969691 | 2040739 | Mycobactin_biosynthetic_gene_cluster (80% of genes show similarity) | BGC0001021_c1 |  |  |
| Cluster 8 | T1pks-Nrps | 2450171 | 2516232 | Griseoviridin_/_viridogrisein_biosynthetic_gene_cluster (5% of genes show similarity) | BGC0000459_c1 | 1 | CYP150A |
| Cluster 9 | T1pks | 2554583 | 2607128 | FD-891_biosynthetic_gene_cluster (50% of genes show similarity) | BGC0000058_c1 | 2 | CYP279A; CYP105U |
| Cluster 10 | Nrps | 3026559 | 3097719 | - | - |  |  |
| Cluster 11 | Nrps | 3120372 | 3169978 | Nocobactin_NA_biosynthetic_gene_cluster (62% of genes show similarity) | BGC0001027_c1 |  |  |
| Cluster 12 | T3pks-T1pks | 3194941 | 3253223 | Streptomycin_biosynthetic_gene_cluster (14% of genes show similarity) | BGC0000717_c1 |  |  |
| Cluster 13 | Nrps | 3341126 | 3445965 | Glycopeptidolipid_biosynthetic_gene_cluster (30% of genes show similarity) | BGC0000362_c1 |  |  |
| Cluster 14 | Nrps | 3454194 | 3504474 | Glycopeptidolipid_biosynthetic_gene_cluster (25% of genes show similarity) | BGC0000362_c1 |  |  |
| Cluster 15 | Other | 3686359 | 3729883 | - | - |  |  |
| Cluster 16 | Terpene | 4095204 | 4116157 | Isorenieratene_biosynthetic_gene_cluster (71% of genes show similarity) | BGC0000664_c1 |  |  |
| Cluster 17 | Other | 4317879 | 4361127 | - | - |  |  |
| Cluster 18 | Other | 4928567 | 4972133 | Heme_D1_biosynthetic_gene_cluster (11% of genes show similarity) | BGC0000905_c1 |  |  |
| Cluster 19 | Nrps | 5199528 | 5249784 | - | - |  |  |
| Cluster 20 | Other | 5437412 | 5480900 | - | - |  |  |
|  |  |  |  | ***Mycobacterium indicus pranii*MTCC 9506** |  |  |  |
| Cluster 1 | T1pks | 381606 | 426918 | - | - |  |  |
| Cluster 2 | Bacteriocin | 810927 | 821724 | - | - |  |  |
| Cluster 3 | Nrps | 1267209 | 1310496 | Glycopeptidolipid_biosynthetic_gene_cluster (15% of genes show similarity) | BGC0000362_c1 |  |  |
| Cluster 4 | T3pks | 1328632 | 1369750 | Alkylresorcinol_biosynthetic_gene_cluster (100% of genes show similarity) | BGC0000282_c1 | 1 | CYP1123A1 |
| Cluster 5 | T1pks | 1371370 | 1417732 | Marinacarboline_biosynthetic_gene_cluster (23% of genes show similarity) | BGC0001137_c1 |  |  |
| Cluster 6 | T1pks-Nrps | 1838180 | 1897014 | Glycopeptidolipid_biosynthetic_gene_cluster (23% of genes show similarity) | BGC0000365_c1 |  |  |
| Cluster 7 | Nrps | 2013424 | 2086499 | Mycobactin_biosynthetic_gene_cluster (80% of genes show similarity) | BGC0001021_c1 |  |  |
| Cluster 8 | T1pks-Nrps | 2378278 | 2444351 | Glycopeptidolipid_biosynthetic_gene_cluster (6% of genes show similarity) | BGC0000365_c1 |  |  |
| Cluster 9 | T1pks | 2479850 | 2532386 | FD-891_biosynthetic_gene_cluster (50% of genes show similarity) | BGC0000058_c1 |  |  |
| Cluster 10 | Nrps | 2964807 | 3036045 | - | - | 1 | CYP126NSF1 |
| Cluster 11 | Nrps | 3096521 | 3146118 | Nocobactin_NA_biosynthetic_gene_cluster (62% of genes show similarity) | BGC0001027_c1 |  |  |
| Cluster 12 | T3pks-T1pks | 3177808 | 3236093 | Streptomycin_biosynthetic_gene_cluster (14% of genes show similarity) | BGC0000717_c1 |  |  |
| Cluster 13 | Nrps | 3330916 | 3435746 | Glycopeptidolipid_biosynthetic_gene_cluster (33% of genes show similarity) | BGC0000362_c1 |  |  |
| Cluster 14 | Nrps | 3445034 | 3495314 | Glycopeptidolipid_biosynthetic_gene_cluster (25% of genes show similarity) | BGC0000362_c1 |  |  |
| Cluster 15 | Other | 3673617 | 3717141 | - | - |  |  |
| Cluster 16 | Terpene | 4089867 | 4110820 | Isorenieratene_biosynthetic_gene_cluster (71% of genes show similarity) | BGC0000664_c1 |  |  |
| Cluster 17 | Other | 4316039 | 4359287 | - | - |  |  |
| Cluster 18 | Other | 4913374 | 4956940 | Heme_D1_biosynthetic_gene_cluster (11% of genes show similarity) | BGC0000905_c1 |  |  |
| Cluster 19 | Nrps | 5182202 | 5232471 | - | - |  |  |
| Cluster 20 | Other | 5423381 | 5466869 | - | - |  |  |
|  |  |  |  | ***Mycobacterium leprae* Br4923** |  |  |  |
| Cluster 1 | T1pks | 106545 | 151899 | - | - |  |  |
| Cluster 2 | T1pks | 160054 | 220598 | Meridamycin_biosynthetic_gene_cluster (15% of genes show similarity) | BGC0001011_c1 |  |  |
| Cluster 3 | Other | 564390 | 607956 | - | - |  |  |
| Cluster 4 | T1pks-Nrps | 1434324 | 1489339 | Glycopeptidolipid_biosynthetic_gene_cluster (13% of genes show similarity) | BGC0000365_c1 |  |  |
| Cluster 5 | T1pks | 2774826 | 2841607 | Glycopeptidolipid_biosynthetic_gene_cluster (13% of genes show similarity) | BGC0000365_c1 |  |  |
|  |  |  |  | ***Mycobacterium leprae* TN** |  |  |  |
| Cluster 1 | T1pks | 106518 | 151872 | - | - |  |  |
| Cluster 2 | T1pks | 160027 | 220571 | Meridamycin_biosynthetic_gene_cluster (15% of genes show similarity) | BGC0001011_c1 |  |  |
| Cluster 3 | Other | 564381 | 607947 | - | - |  |  |
| Cluster 4 | T1pks-Nrps | 1434299 | 1489314 | Glycopeptidolipid_biosynthetic_gene_cluster (13% of genes show similarity) | BGC0000365_c1 |  |  |
| Cluster 5 | T1pks | 2774928 | 2841709 | Glycopeptidolipid_biosynthetic_gene_cluster (13% of genes show similarity) | BGC0000365_c1 |  |  |
|  |  |  |  | ***Mycobacterium* sp. JDM601** |  |  |  |
| Cluster 1 | T1pks-Nrps | 321014 | 379784 | Glycopeptidolipid_biosynthetic_gene_cluster (30% of genes show similarity) | BGC0000365_c1 |  |  |
| Cluster 2 | Nrps | 744617 | 787744 | - | - |  |  |
| Cluster 3 | T3pks | 846764 | 887831 | Alkylresorcinol_biosynthetic_gene_cluster (100% of genes show similarity) | BGC0000282_c1 |  |  |
| Cluster 4 | T1pks-Nrps | 963349 | 1054885 | Glycopeptidolipid_biosynthetic_gene_cluster (16% of genes show similarity) | BGC0000365_c1 |  |  |
| Cluster 5 | Bacteriocin | 1154086 | 1164883 | - | - |  |  |
| Cluster 6 | Bacteriocin | 1202427 | 1213302 | - | - |  |  |
| Cluster 7 | Other | 2438176 | 2480758 | - | - |  |  |
| Cluster 8 | Other | 2752165 | 2799298 | - | - |  |  |
| Cluster 9 | T1pks-Nrps | 2955531 | 3005017 | Glycopeptidolipid_biosynthetic_gene_cluster (16% of genes show similarity) | BGC0000365_c1 | 1 | CYP135B |
| Cluster 10 | Nrps | 3091566 | 3160817 | Glycopeptidolipid_biosynthetic_gene_cluster (52% of genes show similarity) | BGC0000363_c1 |  |  |
| Cluster 11 | Ectoine | 3681298 | 3691690 | Ectoine_biosynthetic_gene_cluster (75% of genes show similarity) | BGC0000853_c1 |  |  |
| Cluster 12 | T1pks-Nrps | 3989573 | 4057345 | Mycobactin_biosynthetic_gene_cluster (90% of genes show similarity) | BGC0001021_c1 |  |  |
| Cluster 13 | Other | 4192488 | 4236717 | - | - |  |  |
| Cluster 14 | Other | 5626427 | 5670359 | - | - |  |  |
| Cluster 15 | Otherks | 5736804 | 5777820 | - | - |  |  |
| Cluster 16 | T1pks | 5971186 | 6016612 | - | - |  |  |
|  |  |  |  | ***Mycobacterium liflandi* 128FXT** |  |  |  |
| Cluster 1 | Nrps | 53996 | 113069 | - | - |  |  |
| Cluster 2 | Lantipeptide | 1028670 | 1051960 | Prodigiosin_biosynthetic_gene_cluster (12% of genes show similarity) | BGC0000257_c1 |  |  |
| Cluster 3 | T1pks-Nrps | 2025342 | 2118939 | Glycopeptidolipid_biosynthetic_gene_cluster (6% of genes show similarity) | BGC0000365_c1 |  |  |
| Cluster 4 | Other | 2191790 | 2234579 | Herboxidiene_biosynthetic_gene_cluster (8% of genes show similarity) | BGC0001065_c1 |  |  |
| Cluster 5 | Nrps | 2519702 | 2575129 | - | - | 1 | CYP143A3 |
| Cluster 6 | Nrps | 2698186 | 2742700 | - | - | 2 | CYP140A5; CYP125A6 |
| Cluster 7 | T1pks | 2975658 | 3028221 | Reveromycin_biosynthetic_gene_cluster (15% of genes show similarity) | BGC0000135_c1 |  |  |
| Cluster 8 | Other | 3088621 | 3132163 | - | - | 1 | CYP147G |
| Cluster 9 | T3pks | 3174471 | 3215652 | BE-7585A_biosynthetic_gene_cluster (16% of genes show similarity) | BGC0000203_c1 |  |  |
| Cluster 10 | Other | 3237240 | 3280764 | - | - |  |  |
| Cluster 11 | Nrps | 3293349 | 3348506 | Calcium-dependent_antibiotic_biosynthetic_gene_cluster (12% of genes show similarity) | BGC0000315_c1 |  |  |
| Cluster 12 | T3pks-T1pks | 3362786 | 3421021 | Streptomycin_biosynthetic_gene_cluster (8% of genes show similarity) | BGC0000717_c1 | 1 | CYP139A3 |
| Cluster 13 | T1pks-Nrps | 3513352 | 3591569 | - | - |  |  |
| Cluster 14 | Terpene | 3732104 | 3753120 | Merochlorin_biosynthetic_gene_cluster (9% of genes show similarity) | BGC0001083_c1 |  |  |
| Cluster 15 | Nrps | 4247597 | 4310181 | Nocobactin_NA_biosynthetic_gene_cluster (87% of genes show similarity) | BGC0001027_c1 |  |  |
| Cluster 16 | T3pks | 4894395 | 4935558 | Alkylresorcinol_biosynthetic_gene_cluster (100% of genes show similarity) | BGC0000282_c1 |  |  |
| Cluster 17 | Terpene | 5448989 | 5469948 | Carotenoid_biosynthetic_gene_cluster (18% of genes show similarity) | BGC0000633_c1 |  |  |
| Cluster 18 | Bacteriocin | 5544946 | 5555752 | - | - |  |  |
| Cluster 19 | Other | 5971253 | 6015179 | - | - |  |  |
| Cluster 20 | T1pks | 6046854 | 6092211 | - | - |  |  |
|  |  |  |  | ***Mycobacterium ulcerans* Agy99** |  |  |  |
| Cluster 1 | Terpene | 376470 | 397429 | Carotenoid_biosynthetic_gene_cluster (14% of genes show similarity) | BGC0000636_c1 |  |  |
| Cluster 2 | Bacteriocin | 413436 | 424242 | - | - |  |  |
| Cluster 3 | Nrps | 1633725 | 1675233 | - | - |  |  |
| Cluster 4 | T3pks-T1pks | 1797861 | 1842169 | Streptomycin_biosynthetic_gene_cluster (6% of genes show similarity) | BGC0000717_c1 |  |  |
| Cluster 5 | Other | 1878764 | 1922288 | Glycopeptidolipid_biosynthetic_gene_cluster (10% of genes show similarity) | BGC0000363_c1 |  |  |
| Cluster 6 | T3pks | 1942027 | 1983208 | BE-7585A_biosynthetic_gene_cluster (16% of genes show similarity) | BGC0000203_c1 |  |  |
| Cluster 7 | T1pks-Nrps | 2194258 | 2286509 | Glycopeptidolipid_biosynthetic_gene_cluster (6% of genes show similarity) | BGC0000365_c1 |  |  |
| Cluster 8 | T1pks | 2501288 | 2553863 | Reveromycin_biosynthetic_gene_cluster (15% of genes show similarity) | BGC0000135_c1 |  |  |
| Cluster 9 | Nrps | 2920775 | 2970209 | Sch47554_/_Sch47555_biosynthetic_gene_cluster (3% of genes show similarity) | BGC0000268_c1 |  |  |
| Cluster 10 | Nrps | 4009686 | 4072249 | Nocobactin_NA_biosynthetic_gene_cluster (87% of genes show similarity) | BGC0001027_c1 |  |  |
| Cluster 11 | Other | 4813556 | 4857482 | - | - |  |  |
| Cluster 12 | T1pks | 5505568 | 5550919 | - | - |  |  |
|  |  |  |  | ***Mycobacterium marinum*** |  |  |  |
| Cluster 1 | T1pks-Nrps | 61110 | 142230 | Borrelidin_biosynthetic_gene_cluster (13% of genes show similarity) | BGC0000031_c1 |  |  |
| Cluster 2 | Amglyccycl | 622806 | 644011 | Acarbose_biosynthetic_gene_cluster (7% of genes show similarity) | BGC0000691_c1 |  |  |
| Cluster 3 | T1pks | 770757 | 818013 | Divergolide_biosynthetic_gene_cluster (17% of genes show similarity) | BGC0001119_c1 |  |  |
| Cluster 4 | Nrps | 937309 | 1003509 | Yatakemycin_biosynthetic_gene_cluster (6% of genes show similarity) | BGC0000466_c1 | 1 | CYP185A4 |
| Cluster 5 | Lantipeptide | 1048397 | 1077803 | Prodigiosin_biosynthetic_gene_cluster (12% of genes show similarity) | BGC0000257_c1 |  |  |
| Cluster 6 | T1pks-Nrps | 1315964 | 1396552 | Meridamycin_biosynthetic_gene_cluster (5% of genes show similarity) | BGC0001011_c1 |  |  |
| Cluster 7 | Nrps | 1447040 | 1496513 | Sch47554_/_Sch47555_biosynthetic_gene_cluster (3% of genes show similarity) | BGC0000268_c1 |  |  |
| Cluster 8 | T1pks-Nrps | 1976632 | 2069138 | Maklamicin_biosynthetic_gene_cluster (15% of genes show similarity) | BGC0001288_c1 |  |  |
| Cluster 9 | Other | 2145225 | 2188281 | Herboxidiene_biosynthetic_gene_cluster (10% of genes show similarity) | BGC0001065_c1 |  |  |
| Cluster 10 | Other | 2414473 | 2457997 | - | - |  |  |
| Cluster 11 | T3pks | 2479648 | 2520829 | BE-7585A_biosynthetic_gene_cluster (16% of genes show similarity) | BGC0000203_c1 |  |  |
| Cluster 12 | T1pks-Nrps | 2653622 | 2725353 | Glycopeptidolipid_biosynthetic_gene_cluster (16% of genes show similarity) | BGC0000365_c1 |  |  |
| Cluster 13 | T3pks-T1pks | 2834948 | 2893181 | Streptomycin_biosynthetic_gene_cluster (8% of genes show similarity) | BGC0000717_c1 | 1 | CYP139A3 |
| Cluster 14 | Nrps | 2909898 | 2958315 | Calcium-dependent_antibiotic_biosynthetic_gene_cluster (12% of genes show similarity) | BGC0000315_c1 |  |  |
| Cluster 15 | Nrps | 3039481 | 3094347 | - | - | 1 | CYP143A3 |
| Cluster 16 | Nrps | 3212598 | 3257064 | - | - | 2 | CYP140A5; CYP125A6 |
| Cluster 17 | Other | 3386607 | 3430149 | - | - | 1 | CYP147G1 |
| Cluster 18 | T1pks | 3490898 | 3543461 | Reveromycin_biosynthetic_gene_cluster (15% of genes show similarity) | BGC0000135_c1 |  |  |
| Cluster 19 | T1pks-Nrps | 3586300 | 3656515 | FD-891_biosynthetic_gene_cluster (62% of genes show similarity) | BGC0000058_c1 |  |  |
| Cluster 20 | Nrps | 3674228 | 3734879 | Glycopeptidolipid_biosynthetic_gene_cluster (21% of genes show similarity) | BGC0000364_c1 |  |  |
| Cluster 21 | Terpene | 3750419 | 3771435 | Merochlorin_biosynthetic_gene_cluster (9% of genes show similarity) | BGC0001083_c1 |  |  |
| Cluster 22 | Nrps | 3799489 | 3888779 | Glycopeptidolipid_biosynthetic_gene_cluster (12% of genes show similarity) | BGC0000362_c1 |  |  |
| Cluster 23 | Nrps | 4272219 | 4321743 | Salinomycin_biosynthetic_gene_cluster (6% of genes show similarity) | BGC0000144_c1 |  |  |
| Cluster 24 | Nrps | 4343500 | 4406063 | Nocobactin_NA_biosynthetic_gene_cluster (87% of genes show similarity) | BGC0001027_c1 |  |  |
| Cluster 25 | T3pks | 4991265 | 5032314 | Alkylresorcinol_biosynthetic_gene_cluster (100% of genes show similarity) | BGC0000282_c1 | 2 | CYP187A; CYP187A |
| Cluster 26 | Terpene | 5563644 | 5584603 | Carotenoid_biosynthetic_gene_cluster (18% of genes show similarity) | BGC0000633_c1 |  |  |
| Cluster 27 | Bacteriocin | 5637840 | 5648646 | - | - |  |  |
| Cluster 28 | Other | 6060204 | 6104130 | - | - | 1 | CYP164A3 |
| Cluster 29 | T1pks | 6166568 | 6211916 | - | - |  |  |
|  |  |  |  | ***Mycobacterium massiliense*** |  |  |  |
| Cluster 1 | T1pks | 186478 | 231814 | - | - |  |  |
| Cluster 2 | Bacteriocin | 618241 | 629038 | - | - |  |  |
| Cluster 3 | Nrps | 1076477 | 1119758 | Glycopeptidolipid_biosynthetic_gene_cluster (15% of genes show similarity) | BGC0000362_c1 |  |  |
| Cluster 4 | T3pks | 1131990 | 1173072 | Alkylresorcinol_biosynthetic_gene_cluster (100% of genes show similarity) | BGC0000282_c1 | 1 | CYP187A |
| Cluster 5 | T1pks | 1174122 | 1220460 | Marinacarboline_biosynthetic_gene_cluster (23% of genes show similarity) | BGC0001137_c1 |  |  |
| Cluster 6 | T1pks-Nrps | 1595844 | 1654671 | Glycopeptidolipid_biosynthetic_gene_cluster (23% of genes show similarity) | BGC0000365_c1 |  |  |
| Cluster 7 | Nrps | 1774929 | 1849514 | Mycobactin_biosynthetic_gene_cluster (80% of genes show similarity) | BGC0001021_c1 |  |  |
| Cluster 8 | T1pks-Nrps | 2235796 | 2301900 | Glycopeptidolipid_biosynthetic_gene_cluster (6% of genes show similarity) | BGC0000365_c1 | 1 | CYP150A |
| Cluster 9 | T1pks | 2339988 | 2392539 | FD-891_biosynthetic_gene_cluster (50% of genes show similarity) | BGC0000058_c1 | 3 | CYP279A; CYP279A; CYP105U |
| Cluster 10 | Other | 2686540 | 2730067 | - | - | 2 | CYP105Q; CYP189A |
| Cluster 11 | Nrps | 2779613 | 2850947 | - | - |  |  |
| Cluster 12 | T3pks-T1pks | 2875790 | 2934084 | Streptomycin_biosynthetic_gene_cluster (14% of genes show similarity) | BGC0000717_c1 |  |  |
| Cluster 13 | Nrps | 3044916 | 3128206 | Glycopeptidolipid_biosynthetic_gene_cluster (33% of genes show similarity) | BGC0000362_c1 |  |  |
| Cluster 14 | Other | 3358669 | 3402181 | - | - |  |  |
| Cluster 15 | Terpene | 3792921 | 3813874 | Isorenieratene_biosynthetic_gene_cluster (71% of genes show similarity) | BGC0000664_c1 |  |  |
| Cluster 16 | Nrps | 4014408 | 4076495 | Borrelidin_biosynthetic_gene_cluster (4% of genes show similarity) | BGC0000031_c1 |  |  |
| Cluster 17 | Nrps | 4131350 | 4182830 | Galbonolides_biosynthetic_gene_cluster (6% of genes show similarity) | BGC0000065_c1 |  |  |
| Cluster 18 | Nrps | 4839148 | 4892609 | - | - |  |  |
| Cluster 19 | Other | 5083791 | 5127279 | Glycopeptidolipid_biosynthetic_gene_cluster (15% of genes show similarity) | BGC0000364_c1 |  |  |
|  |  |  |  | ***Mycobacterium kansassi* ATCC 12478** |  |  |  |
| Cluster 1 | Other | 144113 | 187649 | Glycopeptidolipid_biosynthetic_gene_cluster (12% of genes show similarity) | BGC0000362_c1 |  |  |
| Cluster 2 | Nrps | 200094 | 245025 | - | - |  |  |
| Cluster 3 | Bacteriocin | 269265 | 280125 | - | - |  |  |
| Cluster 4 | Nrps | 769230 | 827336 | Glycopeptidolipid_biosynthetic_gene_cluster (94% of genes show similarity) | BGC0000364_c1 |  |  |
| Cluster 5 | Other | 1483355 | 1526846 | Thiolactomycin_biosynthetic_gene_cluster (100% of genes show similarity) | BGC0001353_c2 |  |  |
| Cluster 6 | Nrps | 1548971 | 1596644 | - | - | 1 | CYP1128A |
| Cluster 7 | T1pks | 1726826 | 1773068 | Glycopeptidolipid_biosynthetic_gene_cluster (13% of genes show similarity) | BGC0000365_c1 |  |  |
| Cluster 8 | Bacteriocin | 1858783 | 1869679 | - | - |  |  |
| Cluster 9 | Other | 1898439 | 1941963 | - | - |  |  |
| Cluster 10 | Ectoine | 2315434 | 2325784 | - | - |  |  |
| Cluster 11 | T1pks-Ectoine-Nrps | 2623296 | 2723115 | Ectoine_biosynthetic_gene_cluster (100% of genes show similarity) | BGC0000853_c1 |  |  |
| Cluster 12 | T2pks-Nrps | 2783808 | 2866995 | Nocobactin_NA_biosynthetic_gene_cluster (75% of genes show similarity) | BGC0001027_c1 |  |  |
| Cluster 13 | T1pks-Nrps | 3802034 | 3860882 | Glycopeptidolipid_biosynthetic_gene_cluster (20% of genes show similarity) | BGC0000365_c1 |  |  |
| Cluster 14 | Bacteriocin | 3998170 | 4008967 | Pactamycin_biosynthetic_gene_cluster (3% of genes show similarity) | BGC0000119_c1 |  |  |
| Cluster 15 | Other | 4172826 | 4216854 | - | - |  |  |
| Cluster 16 | T1pks | 4478946 | 4524336 | - | - |  |  |
|  |  |  |  | ***Mycobacterium* sp. JLS** |  |  |  |
| Cluster 1 | T1pks-Nrps | 236908 | 294426 | Glycopeptidolipid_biosynthetic_gene_cluster (23% of genes show similarity) | BGC0000365_c1 |  |  |
| Cluster 2 | T3pks | 1143068 | 1184120 | Alkylresorcinol_biosynthetic_gene_cluster (100% of genes show similarity) | BGC0000282_c1 |  |  |
| Cluster 3 | Other | 2046311 | 2088803 | - | - |  |  |
| Cluster 4 | Other | 2387869 | 2431393 | - | - |  |  |
| Cluster 5 | T3pks | 2459971 | 2501071 | Alkylresorcinol_biosynthetic_gene_cluster (66% of genes show similarity) | BGC0000282_c1 |  |  |
| Cluster 6 | T1pks-Nrps | 2952303 | 3010907 | Maklamicin_biosynthetic_gene_cluster (4% of genes show similarity) | BGC0001288_c1 |  |  |
| Cluster 7 | T1pks-Nrps | 3247657 | 3304169 | Glycopeptidolipid_biosynthetic_gene_cluster (20% of genes show similarity) | BGC0000365_c1 |  |  |
| Cluster 8 | T1pks-Nrps | 3628609 | 3696583 | Mycobactin_biosynthetic_gene_cluster (80% of genes show similarity) | BGC0001021_c1 |  |  |
| Cluster 9 | Ectoine | 4648250 | 4658648 | Ectoine_biosynthetic_gene_cluster (75% of genes show similarity) | BGC0000853_c1 |  |  |
| Cluster 10 | Nrps | 5097915 | 5151556 | Arsenopolyketides_biosynthetic_gene_cluster (12% of genes show similarity) | BGC0001283_c1 |  |  |
| Cluster 11 | Other | 5394805 | 5438740 | Phosphonoglycans_biosynthetic_gene_cluster (3% of genes show similarity) | BGC0000806_c1 |  |  |
| Cluster 12 | T1pks | 5627176 | 5672701 | - | - |  |  |
| Cluster 13 | Terpene | 5708418 | 5729368 | Isorenieratene_biosynthetic_gene_cluster (71% of genes show similarity) | BGC0000664_c1 |  |  |
|  |  |  |  | ***Mycobacterium* sp. KMS** |  |  |  |
| Cluster 1 | T1pks-Nrps | 259328 | 316819 | Glycopeptidolipid_biosynthetic_gene_cluster (23% of genes show similarity) | BGC0000365_c1 |  |  |
| Cluster 2 | T3pks | 1139543 | 1180595 | Alkylresorcinol_biosynthetic_gene_cluster (100% of genes show similarity) | BGC0000282_c1 |  |  |
| Cluster 3 | Other | 2125389 | 2167881 | - | - |  |  |
| Cluster 4 | Other | 2404377 | 2447901 | - | - |  |  |
| Cluster 5 | T3pks | 2476640 | 2517560 | Alkylresorcinol_biosynthetic_gene_cluster (66% of genes show similarity) | BGC0000282_c1 |  |  |
| Cluster 6 | T1pks-Nrps | 2980304 | 3039499 | Maklamicin_biosynthetic_gene_cluster (4% of genes show similarity) | BGC0001288_c1 |  |  |
| Cluster 7 | T1pks-Nrps | 3292694 | 3358542 | Glycopeptidolipid_biosynthetic_gene_cluster (20% of genes show similarity) | BGC0000365_c1 |  |  |
| Cluster 8 | T1pks-Nrps | 3683064 | 3748128 | Mycobactin_biosynthetic_gene_cluster (80% of genes show similarity) | BGC0001021_c1 |  |  |
| Cluster 9 | Ectoine | 4479778 | 4490167 | Ectoine_biosynthetic_gene_cluster (75% of genes show similarity) | BGC0000853_c1 |  |  |
| Cluster 10 | Other | 5080400 | 5124335 | Phosphonoglycans_biosynthetic_gene_cluster (3% of genes show similarity) | BGC0000806_c1 |  |  |
| Cluster 11 | T1pks | 5321914 | 5367466 | - | - |  |  |
| Cluster 12 | Terpene | 5403154 | 5424104 | Isorenieratene_biosynthetic_gene_cluster (71% of genes show similarity) | BGC0000664_c1 |  |  |
|  |  |  |  | ***Mycobacterium* sp. MCS** |  |  |  |
| Cluster 1 | T1pks-Nrps | 251988 | 309479 | Glycopeptidolipid_biosynthetic_gene_cluster (23% of genes show similarity) | BGC0000365_c1 |  |  |
| Cluster 2 | T3pks | 1135234 | 1176286 | Alkylresorcinol_biosynthetic_gene_cluster (100% of genes show similarity) | BGC0000282_c1 |  |  |
| Cluster 3 | Other | 2106405 | 2148897 | - | - |  |  |
| Cluster 4 | Other | 2385393 | 2428917 | - | - |  |  |
| Cluster 5 | T3pks | 2457656 | 2498576 | Alkylresorcinol_biosynthetic_gene_cluster (66% of genes show similarity) | BGC0000282_c1 |  |  |
| Cluster 6 | T1pks-Nrps | 2962416 | 3021458 | Maklamicin_biosynthetic_gene_cluster (4% of genes show similarity) | BGC0001288_c1 |  |  |
| Cluster 7 | T1pks-Nrps | 3264412 | 3330260 | Glycopeptidolipid_biosynthetic_gene_cluster (20% of genes show similarity) | BGC0000365_c1 |  |  |
| Cluster 8 | T1pks-Nrps | 3654782 | 3719846 | Mycobactin_biosynthetic_gene_cluster (80% of genes show similarity) | BGC0001021_c1 |  |  |
| Cluster 9 | Ectoine | 4451114 | 4461503 | Ectoine_biosynthetic_gene_cluster (75% of genes show similarity) | BGC0000853_c1 |  |  |
| Cluster 10 | Other | 5042742 | 5086677 | Phosphonoglycans_biosynthetic_gene_cluster (3% of genes show similarity) | BGC0000806_c1 |  |  |
| Cluster 11 | T1pks | 5284292 | 5329844 | - | - |  |  |
| Cluster 12 | Terpene | 5365532 | 5386482 | Isorenieratene_biosynthetic_gene_cluster (71% of genes show similarity | BGC0000664_c1 |  |  |
|  |  |  |  | ***Mycobacterium vanbaalenii*PYR-1** |  |  |  |
| Cluster 1 | T1pks-Nrps | 273453 | 330979 | Glycopeptidolipid_biosynthetic_gene_cluster (26% of genes show similarity) | BGC0000365_c1 |  |  |
| Cluster 2 | T1pks-Nrps | 1007476 | 1069625 | Galbonolides_biosynthetic_gene_cluster (6% of genes show similarity) | BGC0000065_c1 |  |  |
| Cluster 3 | T3pks | 1255771 | 1296844 | Alkylresorcinol_biosynthetic_gene_cluster (100% of genes show similarity) | BGC0000282_c1 |  |  |
| Cluster 4 | Terpene | 1662600 | 1683547 | Isorenieratene_biosynthetic_gene_cluster (57% of genes show similarity) | BGC0000664_c1 |  |  |
| Cluster 5 | Other | 2325146 | 2367734 | - | - |  |  |
| Cluster 6 | Other | 2696581 | 2740069 | - | - |  |  |
| Cluster 7 | Other | 2808426 | 2852319 | BE-7585A_biosynthetic_gene_cluster (23% of genes show similarity) | BGC0000203_c1 |  |  |
| Cluster 8 | Linaridin | 2937292 | 2957792 | - | - |  |  |
| Cluster 9 | T1pks-Nrps | 3277119 | 3344407 | Microsclerodermins_biosynthetic_gene_cluster (18% of genes show similarity) | BGC0001019_c1 | 1 | CYP138C1 |
| Cluster 10 | T1pks-Nrps | 4074903 | 4141538 | Mycobactin_biosynthetic_gene_cluster (80% of genes show similarity) | BGC0001021_c1 |  |  |
| Cluster 11 | Bacteriocin | 5481316 | 5492113 | - | - |  |  |
| Cluster 12 | Ectoine | 5641882 | 5652274 | Ectoine_biosynthetic_gene_cluster (75% of genes show similarity) | BGC0000853_c1 |  |  |
| Cluster 13 | Other | 5742762 | 5786718 | Phosphonoglycans_biosynthetic_gene_cluster (3% of genes show similarity) | BGC0000806_c1 |  |  |
| Cluster 14 | T1pks | 6002514 | 6048036 | - | - |  |  |
| Cluster 15 | Terpene | 6292612 | 6313811 | - | - |  |  |
|  |  |  |  | ***Mycobacterium smegmatis* MC2 155** |  |  |  |
| Cluster 1 | Nrps | 1 | 61623 | Albachelin_biosynthetic_gene_cluster (60% of genes show similarity) | BGC0000714_c1 |  |  |
| Cluster 2 | T1pks-Nrps | 427899 | 509098 | Glycopeptidolipid_biosynthetic_gene_cluster (96% of genes show similarity) | BGC0001211_c1 |  |  |
| Cluster 3 | T3pks | 873684 | 914772 | Alkylresorcinol_biosynthetic_gene_cluster (100% of genes show similarity) | BGC0000365_c1 | 1 | CYP185A1 |
| Cluster 4 | Bacteriocin | 2227832 | 2238686 | - | BGC0000282_c1 |  |  |
| Cluster 5 | Terpene | 2416068 | 2437015 | Isorenieratene_biosynthetic_gene_cluster (57% of genes show similarity) | - |  |  |
| Cluster 6 | Other | 2531374 | 2573953 | - | BGC0000664_c1 |  |  |
| Cluster 7 | Other | 2995829 | 3039335 | - | - |  |  |
| Cluster 8 | Nrps | 3739540 | 3805988 | Glycopeptidolipid_biosynthetic_gene_cluster (13% of genes show similarity) | - |  |  |
| Cluster 9 | Ectoine | 3966007 | 3976399 | Ectoine_biosynthetic_gene_cluster (75% of genes show similarity) | BGC0000365_c1 |  |  |
| Cluster 10 | Nrps | 4565368 | 4625560 | Mycobactin_biosynthetic_gene_cluster (90% of genes show similarity) | BGC0000853_c1 |  |  |
| Cluster 11 | T1pks-Nrps | 4799250 | 4851664 | Glycopeptidolipid_biosynthetic_gene_cluster (19% of genes show similarity) | BGC0001021_c1 |  |  |
| Cluster 12 | Nrps | 5027070 | 5080900 | Arginomycin_biosynthetic_gene_cluster (13% of genes show similarity) | BGC0000769_c1 |  |  |
| Cluster 13 | Other | 5801365 | 5844886 | - | BGC0000883_c1 |  |  |
| Cluster 14 | Bacteriocin | 5892680 | 5903477 | - | - |  |  |
| Cluster 15 | Other | 5982575 | 6025196 | - | - |  |  |
| Cluster 16 | Other | 6180052 | 6223987 | Glycopeptidolipid_biosynthetic_gene_cluster (6% of genes show similarity) | - |  |  |
| Cluster 17 | T1pks | 6436780 | 6482305 | - | BGC0000365_c1 |  |  |
| Cluster 18 | T1pks | 6791076 | 6836550 | Sisomicin_biosynthetic_gene_cluster (5% of genes show similarity) | - |  |  |
|  |  |  |  | ***Mycobacterium chubuense* NBB4** |  |  |  |
| Cluster 1 | T1pks-Nrps | 213443 | 270925 | Glycopeptidolipid_biosynthetic_gene_cluster (23% of genes show similarity) | BGC0000365_c1 |  |  |
| Cluster 2 | T3pks | 840785 | 881870 | Alkylresorcinol_biosynthetic_gene_cluster (100% of genes show similarity) | BGC0000282_c1 |  |  |
| Cluster 3 | Terpene | 1177480 | 1198424 | Carotenoid_biosynthetic_gene_cluster (21% of genes show similarity) | BGC0000636_c1 |  |  |
| Cluster 4 | Arylpolyene | 2017291 | 2058448 | - | - | 3 | CYP187A; CYP150A; CYP189A |
| Cluster 5 | Other | 2344446 | 2387940 | - | - |  |  |
| Cluster 6 | Ectoine | 2388530 | 2398922 | Ectoine_biosynthetic_gene_cluster (75% of genes show similarity) | BGC0000853_c1 |  |  |
| Cluster 7 | T1pks-Nrps | 2837828 | 2905289 | Avilamycin_A_biosynthetic_gene_cluster (5% of genes show similarity) | BGC0000026_c1 | 1 | CYP138C |
| Cluster 8 | Other | 3386862 | 3429642 | U-68204_biosynthetic_gene_cluster (14% of genes show similarity) | BGC0001354_c1 |  |  |
| Cluster 9 | Nrps | 3659238 | 3730463 | Glycopeptidolipid_biosynthetic_gene_cluster (13% of genes show similarity) | BGC0000365_c1 |  |  |
| Cluster 10 | T1pks | 4550517 | 4596045 | - | - | 1 | CYP292A |
| Cluster 11 | Bacteriocin | 4717275 | 4728072 | Pactamycin_biosynthetic_gene_cluster (3% of genes show similarity) | BGC0000119_c1 |  |  |
| Cluster 12 | Other | 4958728 | 5002657 | Phosphonoglycans_biosynthetic_gene_cluster (3% of genes show similarity) | BGC0000806_c1 |  |  |
| Cluster 13 | T1pks | 5216614 | 5262085 | - | - |  |  |
|  |  |  |  | ***Mycobacterium  gilvum*PYR-GCK** |  |  |  |
| Cluster 1 | T1pks-Nrps | 380671 | 438247 | Glycopeptidolipid_biosynthetic_gene_cluster (26% of genes show similarity) | BGC0000365_c1 |  |  |
| Cluster 2 | Terpene | 983162 | 1004367 | - | - |  |  |
| Cluster 3 | T1pks | 1194968 | 1240304 | - | - |  |  |
| Cluster 4 | Other | 1443556 | 1487530 | Phosphonoglycans_biosynthetic_gene_cluster (3% of genes show similarity) | BGC0000806_c1 |  |  |
| Cluster 5 | Bacteriocin | 1691217 | 1702014 | Pactamycin_biosynthetic_gene_cluster (3% of genes show similarity) | BGC0000119_c1 |  |  |
| Cluster 6 | Terpene | 1906811 | 1927755 | Isorenieratene_biosynthetic_gene_cluster (71% of genes show similarity) | BGC0000664_c1 | 1 | CYP188A |
| Cluster 7 | T1pks-Nrps | 2796226 | 2861142 | Mycobactin_biosynthetic_gene_cluster (80% of genes show similarity) | BGC0001021_c1 |  |  |
| Cluster 8 | Other | 2995862 | 3038351 | - | - |  |  |
| Cluster 9 | T1pks-Nrps | 3562186 | 3629559 | Avilamycin_A_biosynthetic_gene_cluster (5% of genes show similarity) | BGC0000026_c1 | 1 | CYP138C2 |
| Cluster 10 | T3pks | 4225912 | 4266985 | Herboxidiene_biosynthetic_gene_cluster (6% of genes show similarity) | BGC0001065_c1 |  |  |
| Cluster 11 | Other | 4374244 | 4416826 | - | - | 3 | CYP189A; CYP150A; CYP187A |
| Cluster 12 | Ectoine | 5143127 | 5153519 | Ectoine_biosynthetic_gene_cluster (75% of genes show similarity) | BGC0000853_c1 |  |  |
| Cluster 13 | Other | 5282888 | 5323730 | - | - |  |  |
| Cluster 14 | T3pks | 5449695 | 5490777 | Alkylresorcinol_biosynthetic_gene_cluster (100% of genes show similarity) | BGC0000282_c1 |  |  |
|  |  |  |  | ***Mycobacterium gilvum* Spyr1** |  |  |  |
| Cluster 1 | T1pks-Nrps | 339200 | 396776 | Glycopeptidolipid_biosynthetic_gene_cluster (26% of genes show similarity) | BGC0000365_c1 |  |  |
| Cluster 2 | Bacteriocin | 1026345 | 1037142 | Pactamycin_biosynthetic_gene_cluster (3% of genes show similarity) | BGC0000119_c1 |  |  |
| Cluster 3 | Terpene | 1258378 | 1279322 | Isorenieratene_biosynthetic_gene_cluster (71% of genes show similarity) | BGC0000664_c1 | 1 | CYP188A |
| Cluster 4 | T1pks-Nrps | 2157546 | 2222588 | Mycobactin_biosynthetic_gene_cluster (80% of genes show similarity) | BGC0001021_c1 |  |  |
| Cluster 5 | T1pks-Nrps | 2775797 | 2843206 | Avilamycin_A_biosynthetic_gene_cluster (5% of genes show similarity) | BGC0000026_c1 | 1 | CYP138C2 |
| Cluster 6 | T3pks | 3435596 | 3476669 | Herboxidiene_biosynthetic_gene_cluster (6% of genes show similarity) | BGC0001065_c1 |  |  |
| Cluster 7 | Other | 3582553 | 3625135 | - | - | 3 | CYP189A; CYP150A; CYP187A |
| Cluster 8 | Ectoine | 4395958 | 4406350 | Ectoine_biosynthetic_gene_cluster (75% of genes show similarity) | BGC0000853_c1 |  |  |
| Cluster 9 | Other | 4540020 | 4580862 | - | - |  |  |
| Cluster 10 | T3pks | 4706085 | 4747167 | Alkylresorcinol_biosynthetic_gene_cluster (100% of genes show similarity) | BGC0000282_c1 |  |  |
| Cluster 11 | Other | 4928470 | 4972444 | Phosphonoglycans_biosynthetic_gene_cluster (3% of genes show similarity) | BGC0000806_c1 |  |  |
| Cluster 12 | T1pks | 5182179 | 5227515 | - | - |  |  |
| Cluster 13 | Terpene | 5411716 | 5432921 | - | - |  |  |
|  |  |  |  | ***Mycobacterium smegmatis*JS623** |  |  |  |
| Cluster 1 | T1pks-Nrps | 195961 | 253457 | Glycopeptidolipid_biosynthetic_gene_cluster (26% of genes show similarity) | BGC0000365_c1 |  |  |
| Cluster 2 | Nrps | 346982 | 404899 | Glycopeptidolipid_biosynthetic_gene_cluster (78% of genes show similarity) | BGC0000364_c1 |  |  |
| Cluster 3 | Other | 627984 | 671520 | - | - |  |  |
| Cluster 4 | T3pks | 724354 | 765415 | Alkylresorcinol_biosynthetic_gene_cluster (100% of genes show similarity) | BGC0000282_c1 | 1 | CYP135B |
| Cluster 5 | T3pks | 1146215 | 1187333 | - | - |  |  |
| Cluster 6 | Other | 2284486 | 2326996 | - | - | 2 | CYP187A; CYP150A |
| Cluster 7 | Other | 2640333 | 2683839 | - | - |  |  |
| Cluster 8 | Nrps | 3391916 | 3433310 | Glycopeptidolipid_biosynthetic_gene_cluster (7% of genes show similarity) | BGC0000362_c1 |  |  |
| Cluster 9 | T1pks | 4162220 | 4206377 | Meilingmycin_biosynthetic_gene_cluster (2% of genes show similarity) | BGC0000093_c1 |  |  |
| Cluster 10 | T3pks | 4294384 | 4335520 | Griseoviridin_/_viridogrisein_biosynthetic_gene_cluster (5% of genes show similarity) | BGC0000459_c1 |  |  |
| Cluster 11 | Terpene | 4848846 | 4869796 | Isorenieratene_biosynthetic_gene_cluster (71% of genes show similarity) | BGC0000664_c1 |  |  |
| Cluster 12 | T1pks-Nrps | 5451816 | 5505010 | - | - | 1 | CYP136NSF2 |
| Cluster 13 | Bacteriocin | 5598281 | 5609078 | - | - |  |  |
| Cluster 14 | T1pks-Nrps | 5785277 | 5864758 | Chartreusin_biosynthetic_gene_cluster (15% of genes show similarity) | BGC0000206_c2 |  |  |
| Cluster 15 | Other | 5892270 | 5936181 | Phosphonoglycans_biosynthetic_gene_cluster (3% of genes show similarity) | BGC0000806_c1 |  |  |
| Cluster 16 | T1pks | 6130226 | 6175661 | - | - |  |  |
|  |  |  |  | ***Mycobacterium rhodesiae*** |  |  |  |
| Cluster 1 | T1pks | 48296 | 93830 | Galbonolides_biosynthetic_gene_cluster (6% of genes show similarity) | BGC0000065_c1 | 2 | CYP189A; CYP102NSF1 |
| Cluster 2 | T1pks-Nrps | 249686 | 327946 | Glycopeptidolipid_biosynthetic_gene_cluster (33% of genes show similarity) | BGC0000362_c1 | 1 | CYP1121A1 |
| Cluster 3 | Arylpolyene | 486822 | 528012 | - | - |  |  |
| Cluster 4 | Nrps | 1009235 | 1052347 | - | - |  |  |
| Cluster 5 | Lantipeptide | 1503784 | 1532987 | - | - |  |  |
| Cluster 6 | Terpene | 1776658 | 1797842 | - | - |  |  |
| Cluster 7 | T1pks | 1951592 | 1997159 | - | - |  |  |
| Cluster 8 | Other | 2181020 | 2224934 | Phosphonoglycans_biosynthetic_gene_cluster (3% of genes show similarity) | BGC0000806_c1 |  |  |
| Cluster 9 | Nrps | 2272026 | 2324007 | - | - | 1 | CYP125A |
| Cluster 10 | Bacteriocin | 2535310 | 2546107 | - | - |  |  |
| Cluster 11 | Terpene | 3638976 | 3659923 | Isorenieratene_biosynthetic_gene_cluster (71% of genes show similarity) | BGC0000664_c1 |  |  |
| Cluster 12 | T1pks-Nrps | 4300696 | 4361762 | Mycobactin_biosynthetic_gene_cluster (60% of genes show similarity) | BGC0001021_c1 |  |  |
| Cluster 13 | Nrps | 4719612 | 4799700 | Glycopeptidolipid_biosynthetic_gene_cluster (10% of genes show similarity) | BGC0000365_c1 | 1 | CYP189A |
| Cluster 14 | Arylpolyene | 4940071 | 4981234 | Glycopeptidolipid_biosynthetic_gene_cluster (5% of genes show similarity) | BGC0000362_c1 | 1 | CYP124A |
| Cluster 15 | Nrps | 5038727 | 5113037 | Pentalenolactone_biosynthetic_gene_cluster (23% of genes show similarity) | BGC0000678_c1 |  |  |
| Cluster 16 | Other | 5543496 | 5586996 | - | - |  |  |
| Cluster 17 | Other | 5848025 | 5890520 | - | - |  |  |
| Cluster 18 | Arylpolyene | 6144777 | 6185937 | - | - | 1 | CYP144A |
|  |  |  |  | ***Mycobacterium neoaurum* VKM Ac-18150** |  |  |  |
| Cluster 1 | T1pks | 62335 | 107761 | - | - |  |  |
| Cluster 2 | Other | 154612 | 198061 | Herboxidiene_biosynthetic_gene_cluster (3% of genes show similarity) | BGC0001065_c1 |  |  |
| Cluster 3 | Terpene | 561323 | 582507 | - | - |  |  |
| Cluster 4 | T1pks-Nrps | 632798 | 691946 | Glycopeptidolipid_biosynthetic_gene_cluster (33% of genes show similarity) | BGC0000365_c1 |  |  |
| Cluster 5 | Other | 2319764 | 2362295 | - | - |  |  |
| Cluster 6 | Other | 2706443 | 2750177 | BE-7585A_biosynthetic_gene_cluster (9% of genes show similarity) | BGC0000203_c1 |  |  |
| Cluster 7 | T1pks-Nrps | 2819680 | 2872045 | Griseobactin_biosynthetic_gene_cluster (11% of genes show similarity) | BGC0000368_c1 |  |  |
| Cluster 8 | Ectoine | 3002251 | 3012649 | Ectoine_biosynthetic_gene_cluster (100% of genes show similarity) | BGC0000853_c1 |  |  |
| Cluster 9 | T1pks | 3678348 | 3723822 | Laspartomycin_biosynthetic_gene_cluster (9% of genes show similarity) | BGC0000379_c1 |  |  |
| Cluster 10 | T1pks-Nrps | 3912925 | 3978393 | Mycobactin_biosynthetic_gene_cluster (80% of genes show similarity) | BGC0001021_c1 |  |  |
| Cluster 11 | Terpene | 4742141 | 4810153 | Isorenieratene_biosynthetic_gene_cluster (71% of genes show similarity) | BGC0000664_c1 |  |  |
| Cluster 12 | Bacteriocin | 4977471 | 4988268 | - | - |  |  |
| Cluster 13 | Other | 5022054 | 5065536 | Rifamycin_biosynthetic_gene_cluster (3% of genes show similarity) | BGC0000136_c1 | 1 | CYP161NSF1 |
| Cluster 14 | T1pks | 5094761 | 5139101 | - | - |  |  |
| Cluster 15 | Other | 5274956 | 5318840 | Galbonolides_biosynthetic_gene_cluster (10% of genes show similarity) | BGC0000065_c1 |  |  |
