# Supplementary material for: Similarities, variations, and evolution of cytochrome P450s in Streptomyces versus Mycobacterium
Source: Sci Rep. 2019 Mar 8;9:3962. doi: 10.1038/s41598-019-40646-y (PMC6408508; doi:10.1038/s41598-019-40646-y)
Supplement: Supplementary file 4 — Dataset 3 [file 41598_2019_40646_MOESM4_ESM.pdf]

Similarities, variations, and evolution of cytochrome P450s in *Streptomyces* versus *Mycobacterium*

Louisa Moshoeshoe Senate[1@], Martin Phalane Tjatji[1@], Kayla Pillay[2@], Wanping Chen[3@], Ntokozo Minenhle Zondo[2], Puleng Rosinah Syed[4], Fanele Cabangile Mnguni[2], Zinhle Edith Chiliza[2], Hans Denis Bamal[1], Rajshekhar Karpoormath[4], Thandeka Khoza[5], Samson Sitheni Mashele[1], Jonathan Michael Blackburn[6], Jae-Hyuk Yu[7,8], David R Nelson[9*], Khajamohiddin Syed[2*]

[1] Unit for Drug Discovery Research, Department of Health Sciences, Faculty of Health and Environmental Sciences, Central University of Technology, Bloemfontein 9300, Free State, South Africa

[2] Department of Biochemistry and Microbiology, Faculty of Science and Agriculture, University of Zululand, KwaDlangezwa 3886, KwaZulu-Natal, South Africa

[3] College of Food Science and Technology, Huazhong Agricultural University, Wuhan, Hubei Province, China

[4] Department of Pharmaceutical Chemistry, College of Health Sciences, University of KwaZulu-Natal, Durban 4000, KwaZulu-Natal, South Africa

[5] Department of Biochemistry, School of Life Sciences, University of KwaZulu-Natal (Pietermaritzburg campus), Scottsville, 3209, KwaZulu-Natal, South Africa

[6] Institute of Infectious Disease & Molecular Medicine; Department of Integrative Biomedical Sciences, Faculty of Health Sciences, University of Cape Town, Cape Town 7925, South Africa

[7] Department of Bacteriology, University of Wisconsin-Madison, 3155 MSB, 1550 Linden Drive, Madison WI 53706, USA

[8] Department of Systems Biotechnology, Konkuk University, Seoul, Republic of Korea.

[9] Department of Microbiology, Immunology and Biochemistry, University of Tennessee Health Science Center, Memphis, TN 38163, USA

@ Authors contributed equally to the work

* Corresponding authors' email:

drnelson1@gmail.com & khajamohiddinsyed@gmail.com

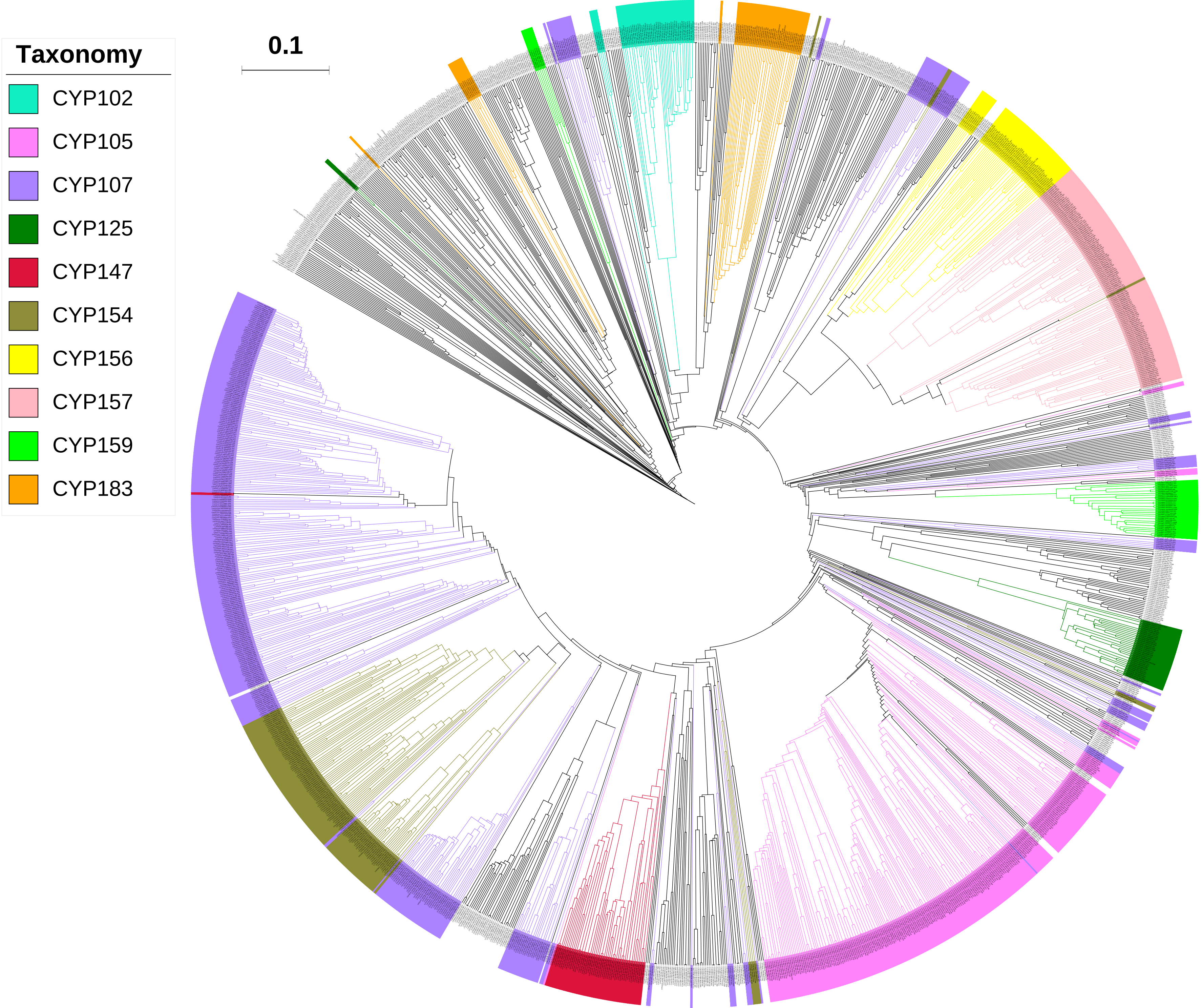
Taxonomy
CYP102
CYP105
CYP107
CYP125
CYP147
CYP154
CYP156
CYP157
CYP159
CYP183
0.1
