# Supplementary material for: Similarities, variations, and evolution of cytochrome P450s in Streptomyces versus Mycobacterium
Source: Sci Rep. 2019 Mar 8;9:3962. doi: 10.1038/s41598-019-40646-y (PMC6408508; doi:10.1038/s41598-019-40646-y)
Supplement: Supplementary file 5 — Dataset 4 [file 41598_2019_40646_MOESM5_ESM.docx]

**Similarities, variations, and evolution of cytochrome P450s in *Streptomyces* versus *Mycobacterium***

Louisa Moshoeshoe Senate^1@^, Martin Phalane Tjatji^1@^, Kayla Pillay^2@^, Wanping Chen^3@^, Ntokozo Minenhle Zondo^2^, Puleng Rosinah Syed^4^, Fanele Cabangile Mnguni^2^, Zinhle Edith Chiliza^2^, Hans Denis Bamal^1^, Rajshekhar Karpoormath^4^, Thandeka Khoza^5^, Samson Sitheni Mashele^1^, Jonathan Michael Blackburn^6^, Jae-Hyuk Yu^7,8^, David R Nelson^9*^, Khajamohiddin Syed^2*^

^1^ Unit for Drug Discovery Research, Department of Health Sciences, Faculty of Health and Environmental Sciences, Central University of Technology, Bloemfontein 9300, Free State, South Africa

^2^Department of Biochemistry and Microbiology, Faculty of Science and Agriculture, University of Zululand, KwaDlangezwa 3886, KwaZulu-Natal, South Africa

^3^ College of Food Science and Technology, Huazhong Agricultural University, Wuhan, Hubei Province, China

^4^ Department of Pharmaceutical Chemistry, College of Health Sciences, University of KwaZulu-Natal, Durban 4000, KwaZulu-Natal, South Africa

^5^Department of Biochemistry, School of Life Sciences, University of KwaZulu-Natal (Pietermaritzburg campus), Scottsville, 3209, KwaZulu-Natal, South Africa

^6^Institute of Infectious Disease & Molecular Medicine; Department of Integrative Biomedical Sciences, Faculty of Health Sciences, University of Cape Town, Cape Town 7925, South Africa

^7^Department of Bacteriology, University of Wisconsin-Madison, 3155 MSB, 1550 Linden Drive, Madison WI 53706, USA

^8^ Department of Systems Biotechnology, Konkuk University, Seoul, Republic or Korea

^9^ Department of Microbiology, Immunology and Biochemistry, University of Tennessee Health Science Center, Memphis, TN 38163, USA

 @ Authors contributed equally to the work

* Corresponding authors’ email:

[drnelson1@gmail.com](mailto:drnelson1@gmail.com) & [khajamohiddinsyed@gmail.com](mailto:khajamohiddinsyed@gmail.com)

**Table S1. General information on *Streptomyces* species used in the study.**

| **Species name** | **Specie characteristic or well-known for** | **References** |
| --- | --- | --- |
| *Streptomyces coelicolor* | It is involved in breaking down organic material in the soil and responsible for producing a majority of the antibiotics in use today, as well as some immunosuppressant and anti-tumor agents. It has also been used in genetic engineering studies. | 1 |
| *Streptomyces avermitilis* | It produces certain secondary metabolites, namely avermectin, which is a potent agent against a wide array of nematodes and arthropod parasites. | 2, 3 |
| *Streptomyces griseus* | It produces streptomycin, a broad-spectrum antibiotic, which has been used for the treatment of various diseases such as tuberculosis and the plague caused by *Yersinia pestis*. The organism has also been reported to contain a gene cluster that includes fredericamycin, an anticancer drug. | 4, 5, 6 |
| *Streptomyces globisporus* | It produces antitumor angucyclines landomycin A and landomycin E and enediyne antitumor antibiotic C-1027. | 7 |
| *Streptomyces scabiei* | It is a plant pathogen causing corky lesions to form on tuber and root crops as well as decreasing the growth of seedlings. Along with other closely related species it causes potato diseases. It can produce several related toxins, which are mainly responsible for its pathogenicity and moreover, *S. scabiei* produces toxins called thaxtomins, which cause hypertrophy and cell death in host plant tissues and are essential for pathogenicity. | 8 |
| *Streptomyces* sp. *Sirex* AA-E | It is an aerobic microbe that is a prominent member of a bacterial/fungal symbiotic community associated with the invasive pinewood-boring wasp Sirex noctilio. *Streptomyces* sp. *Sirex* AA-E has shown that it secretes a full suite of endo- and exo-cellulases, hemicellulases, pectinases and polysaccharide monooxygenases when grown on biomass. | 9 |
| *Streptomyces violaceusniger* | It produces antibiotics that inhibit the growth of other bacteria. | 10 |
| *Streptomyces cattleya* NRRL 8057 = DSM 46488 | It produces the antibiotics thienamycin and cephamycin C and is one of the rare bacteria known to synthesize fluorinated metabolites. | 11 |
| *Streptomyces pratensis* | It shows chemotaxonomic characteristics and also produces antibacterial compounds. | 12 |
| *Streptomyces bingchenggensis* | It is a soil bacterium that produces milbemycins, a family of macrolide antibiotics that are commercially important in crop protection and veterinary medicine. This species has a huge number of protein-coding genes and a huge number of transport proteins, which form systems that are important for metabolism and also enable their adaptation to complex environments. | 13 |
| *Streptomyces hygroscopicus* subsp. *jinggangensis* 5008 | It produces jingangmycin, aminocyclitol antibiotic, which has been proven to be identical to validamycin. Jingangmycin has been widely used as a prime control reagent against sheath blight disease of rice plants and damping-off of cucumber seedlings in China and many other Eastern Asian countries. Meanwhile, its transformed product valienamine is a pharmaceutically important precursor for the synthesis of voglibose, a highly effective drug for insulin-independent diabetes. | 14 |
| *Streptomyces hygroscopicus* subsp. *jinggangensis* TL01 | It produces antimycin A, which is widely used as a pesticide in the catfish farming industry and also has potent killing activity against insects, nematodes and fungi. | 15 |
| *Streptomyces venezuelae* | It produces chloromyeetin and jadomycin. | 16 |
| *Streptomyces davawensis* | It produces the riboflavin analogs roseoflavin and 8-demethyl-8-amino-riboflavin. Both compounds show antimicrobial activity against Gram-positive bacteria such as *Bacillus subtilis,* but also against Gram-negative bacteria if uptake systems for flavins/flavin analogs are present. | 17 |
| *Streptomyces albus* J1074 | It is a widely used host for heterogenous expression of bioactive natural products. | 18 |
| *Streptomyces albus* DSM 41398 | It produces salinomycin. Salinomycin has selective activity against cancer. However, it has been widely used as veterinary medicine in animal husbandry as food additive and growth promoter for years. | 19 |
| *Streptomyces* sp. PAMC 26508 | This is an endosymbiotic bacterium isolated from the Antarctic lichen *Cladonia borealis*. | 20 |
| *Streptomyces fulvissimus* | It produces the ionophore antibiotic valinomycin. Secretes an antibacterial protein inhibitory to *Micrococcus luteus*, *Bacillus subtillis*, *Bacillus cereus*, and methicillin-resistant *Staphylococcus aureas* strains. | 21 |
| *Streptomyces collinus* | It produces ansatrienin A2, ansatrienin A3, ansatrienin B, napthomycin A, collinomycine, toromycin, streptocollin, kirromycin (elfamycin-family) and rubromycine. Causes common scab of potato. | 22 |
| *Streptomyces rapamycinicus* | It produces produce rapamycin (also known as sirolimus) and azalomucin C, a macrodiolide antibiotic used as anthelmintic in animal fodder. Used as an immunosuppressant in renal transplant and for the prevention of restenosis after stent insertion for the treatment of coronary heart disease. | 23 |
| *Streptomyces albulus* NK660 | It produces ε-Poly-L-lysine (ε-PL). ε-PL exhibits antimicrobial activity against a wide spectrum of microorganisms. Exhibits antiphage activity, and ε -PL has been used as a food preservative. | 24 |
| *Streptomyces albulus* ZPM | This species produces the homopolymer antibiotic, e-poly-lysine (e-PL). | 25 |
| *Streptomyces lividans* | Used as a host for the synthesis and secretion of homologous and heterologous proteins of industrial interest. | 26 |
| *Streptomyces glaucescens* | It produces tetracenomycin C, tetracenomycin D and tetracenomycin E. Functions to steer chemical outcome of cyclization. | 27, 28 |
| *Streptomyces vietnamensis* | It forms a white aerial mycelium and a reddish brown substrate mycelium. It shows antibacterial activity against other bacterial species such as *Escherichia coli*, *Pseudomonas aeruginosa*, *Candida albicans*, *Penicillium citrinum* and *B. subtilis.* | 29, 30 |
| *Streptomyces* sp. 769 | It produces cytotoxic and antibacterial secondary metabolites. | 31 |
| *Streptomyces cyaneogriseus* | It is a thermotolerant Streptomyces that produces nemadectin, which is a commercially important antibiotic. This is a macrolide antibiotic widely used as a biopesticide. It also produces chitosanase enzyme, which allows it to break down chitosan as a sole carbon source. | 32 |
| *Streptomyces* sp. A02 | This species produces antifungal metabolites. It is a producer of commercially important polyene macrocylic antibiotic natamycin and potential biocontrol agent to several plant fungal diseases. | 33 |
| *Streptomyces xiamenensis* | It produces an anti-fibrotic benzopyran called xiamenmycin, a compound used for treating fibrotic diseases such as idiopathic pulmonary fibrosis, liver cirrhosis, progressive kidney disease, systemic sclerosis and cardiovascular fibrosis. | 34 |
| *Streptomyces* sp. Mg1 | A streptomyces producing a macrolide antibiotic called chalcomycin A, which takes part in inhibition of *B. subtilis* cell growth in combination by lysis and degradation of *B. subtilis* colonies. | 35, 36 |
| *Streptomyces* sp. CNQ-509 | It contains 29 putative gene clusters used for biosynthesis of secondary metabolites, which are involved in the formation of meroterpenoid molecules. It produces a variety of terpenoid compounds. | 37 |
| *Streptomyces ambofaciens* | It produces two antibiotics, namely spiramycin, which is a polyketide derivative, and congocidin (netropsin). Reports have indicated that it comprises 23 gene clusters, which produce other secondary compounds such as kinamycins, antimycins and stambomycins. | 38-41 |
| *Streptomyces pristinaespiralis* | This streptomyces produces two antibiotics, namely cyclohexadepsiptide pristinamycin (PI) and polyunsaturated macrolactone pristinamycin II (PII) co-produced at a ratio of 30:70. The PI plays a vital role in synergistic antibacterial activity of a wide range of gram-positive bacteria such as methicillin-resistant *Staphylococci*, drug-resistant *Streptococcus pneumonia* and vancomycin-resistant *Enterococcus faecium* and Gram negative bacteria such as *Haemophilus* sp. | 42-44 |
| *Streptomyces* sp. CFMR 7 | This species was discovered in rubber plantations in Penang Malaysia. It is has been found to degrade rubber. | 45, 46 |
| *Streptomyces* sp. CdTB01 | It was isolated from soil contaminated with heavy metals. It can tolerate high concentrations of heavy metals such as cadmium. | 13 |
| *Streptomyces reticuli* | It produces mycelia-associated cellulose, which can degrade crystalline cellulose to cellobiose. | 47, 48 |
| *Streptomyces* sp. 4F | Found in the soil in China, it is a fast grower and moderately thermophilic, i.e it grows at temperatures between 30^o^C and 50^o^C. It is therefore used for antibiotic expression and DNA assembly. | 49, 50 |
| *Streptomyces leeuwenhoekii* | It was isolated from the hyper-arid high-altitude Atacama Desert in Chile. It produces novel polyketide antibiotics such as the chaxamycins and chaxalactins. Chaxamycins A-D are ansamycin-type polyketides with potential antibacterial activity against MRSA. It is involved in anti-proliferative activity, which results from the inhibition of the ATPase activity of the human Hsp90 protein. | 51 |
| *Streptomyces rubrolavendulae* | It has the potential to produce an anti-methicillin resistant *Staphyloccocus aureus* compound. | 52 |
| *Streptomyces parvulus* | This streptomyces produces a polypeptide antibiotic called Actinomycin D, which is a potential anti-microbial agent against streptomycin-resistant pathogenic bacteria. | 53, 54 |
| *Streptomyces lydicus* | It produces actithiazic acid, streptolydigin, lydimycin, natamycin and 1-deoxygalactonojirimycin. It has the ability to be used as an agent against fungal plant pathogens. It has also been reported that the species *S. lydicus* WYEC108 is a strong antagonist against various fungal plant root-rot and white-rot pathogens and a number of wood decay fungi. | 55-58 |
| *Streptomyces clavuligerus* | It produces more than 20 secondary metabolites, which include clavulanic acid that is a beta-lactam antibiotic. Clavulanic acid is a common antibiotic used in combination with *β*-lactam antibiotics (for example, Augmentin) to treat infections caused by *β*-lactamase-producing pathogens. It also synthesizes the *β*‑lactam antibiotic cephamycin C and numerous antifungal compounds with a clavam structure. | 59-61 |
| *Streptomyces griseochromogenes* | It produces blasticidin A, B, C, S. Blasticidin S is a potent antifungal and cytotoxic peptidyl nucleoside antibiotic. It also produces pentalenene and cytomycin. | 62, 63 |
| *Streptomyces lincolnensis* | It produces lincomycin, which is an anti-bacterial antibiotic. It also produces valienol, a C-7 cyclitol similar in structure to valienamine. | 64 |
| *Streptomyces noursei* | It produces nystatin fungicidin, a polyene antifungal medication. | 65 |

**Table S2. List of *Streptomyces* species used in the study.** Species names, codes, the respective genome database links and genome IDs (from NCBI) were listed in the table.

| **Species name** | **Species code** | **Database link** | **Genome ID** |
| --- | --- | --- | --- |
| *Streptomyces coelicolor* | sco | https://img.jgi.doe.gov/cgi-bin/m/main.cgi?section=TaxonDetail&page=taxonDetail&taxon_oid=637000305 | [NC_003888](http://www.genome.jp/dbget-bin/www_bget?refseq+NC_003888) |
| *Streptomyces avermitilis* MA-4680 | sma | https://img.jgi.doe.gov/cgi-bin/m/main.cgi?section=TaxonDetail&page=taxonDetail&taxon_oid=637000304 | [BA000030](https://www.ncbi.nlm.nih.gov/nuccore/BA000030) |
| *Streptomyces griseus_*NBRC_13350 | sgr | https://img.jgi.doe.gov/cgi-bin/m/main.cgi?section=TaxonDetail&page=iprGeneList&ext_accession=IPR001128&taxon_oid=641522653 | [AP009493](https://www.ncbi.nlm.nih.gov/nuccore/AP009493) |
| *Streptomyces globisporus* | sgb | https://img.jgi.doe.gov/cgi-bin/m/main.cgi?section=GeneDetail&page=genePageMainFaa&gene_oid=2668573307 | [CP013738](https://www.ncbi.nlm.nih.gov/nuccore/CP013738) |
| *Streptomyces scabiei* 87.22 | scb | https://img.jgi.doe.gov/cgi-bin/m/main.cgi?section=TaxonDetail&page=iprGeneList&ext_accession=IPR001128&taxon_oid=646564576 | [FN554889](https://www.ncbi.nlm.nih.gov/nuccore/FN554889) |
| *Streptomyces sp. Sirex* AA-E | ssx | https://img.jgi.doe.gov/cgi-bin/m/main.cgi?section=TaxonDetail&page=iprGeneList&ext_accession=IPR001128&taxon_oid=2523533511 | [CP002993](https://www.ncbi.nlm.nih.gov/nuccore/CP002993) |
| *Streptomyces violaceusniger* Tu 4113 | svl | https://img.jgi.doe.gov/cgi-bin/m/main.cgi?section=TaxonDetail&page=iprGeneList&ext_accession=IPR001128&taxon_oid=648276750 | [CP002994](https://www.ncbi.nlm.nih.gov/nuccore/CP002994) |
| *Streptomyces cattleya* NRRL 8057 | sct | https://img.jgi.doe.gov/cgi-bin/m/main.cgi?section=TaxonDetail&page=taxonDetail&taxon_oid=2511231113 | [FQ859185](https://www.ncbi.nlm.nih.gov/nuccore/FQ859185) |
| *Streptomyces cattleya* NRRL 8058 = DSM 46488 | scy | https://img.jgi.doe.gov/cgi-bin/m/main.cgi?section=TaxonDetail&page=taxonDetail&taxon_oid=2511231200 | [CP003219](https://www.ncbi.nlm.nih.gov/nuccore/CP003219) |
| *Streptomyces pratensis/flavogriseus* IAF 45 | sfa | <http://www.genome.jp/kegg-bin/show_organism?org=sfa> | [CP002475](https://www.ncbi.nlm.nih.gov/nuccore/CP002475) |
| *Streptomyces bingchenggensis* | sbh | https://img.jgi.doe.gov/cgi-bin/m/main.cgi?section=TaxonDetail&page=taxonDetail&taxon_oid=646862346 | [CP002047](https://www.ncbi.nlm.nih.gov/nuccore/CP002047) |
| *Streptomyces hygroscopicus subsp. jinggangensis* 5008 | shy | <http://www.genome.jp/kegg-bin/show_organism?org=shy> | [CP003275](https://www.ncbi.nlm.nih.gov/nuccore/CP003275) |
| *Streptomyces hygroscopicus subsp. jinggangensis* TL01 | sho | <http://www.genome.jp/kegg-bin/show_organism?org=sho> | [CP003720](https://www.ncbi.nlm.nih.gov/nuccore/CP003720) |
| *Streptomyces venezuelae* | sve | https://img.jgi.doe.gov/cgi-bin/m/main.cgi?section=TaxonDetail&page=iprGeneList&ext_accession=IPR001128&taxon_oid=2718218231 | [FR845719](https://www.ncbi.nlm.nih.gov/nuccore/FR845719) |
| *Streptomyces davawensis* | sdv | https://img.jgi.doe.gov/cgi-bin/m/main.cgi?section=TaxonDetail&page=taxonDetail&taxon_oid=2561511188 | [HE971709](https://www.ncbi.nlm.nih.gov/nuccore/HE971709) |
| *Streptomyces albus* J1074 | salb | https://img.jgi.doe.gov/cgi-bin/m/main.cgi?section=TaxonDetail&page=taxonDetail&taxon_oid=2541047081 | [CP004370](https://www.ncbi.nlm.nih.gov/nuccore/CP004370) |
| *Streptomyces albus* DSM 41398 | sals | https://img.jgi.doe.gov/cgi-bin/m/main.cgi?section=TaxonDetail&page=taxonDetail&taxon_oid=2639762818 | [CP010519](https://www.ncbi.nlm.nih.gov/nuccore/CP010519) |
| *Streptomyces* sp. PAMC 26508 | strp | https://img.jgi.doe.gov/cgi-bin/m/main.cgi?section=TaxonDetail&page=iprGeneList&ext_accession=IPR001128&taxon_oid=2561511190 | [CP003990](https://www.ncbi.nlm.nih.gov/nuccore/CP003990) |
| *Streptomyces fulvissimus* | sfi | https://img.jgi.doe.gov/cgi-bin/m/main.cgi?section=TaxonDetail&page=taxonDetail&taxon_oid=2554235391 | [CP005080](https://www.ncbi.nlm.nih.gov/nuccore/CP005080) |
| *Streptomyces collinus* | sci | https://img.jgi.doe.gov/cgi-bin/m/main.cgi?section=TaxonDetail&page=taxonDetail&taxon_oid=2554235367 | [CP006259](https://www.ncbi.nlm.nih.gov/nuccore/CP006259) |
| *Streptomyces rapamycinicus* | src | https://img.jgi.doe.gov/cgi-bin/m/main.cgi?section=TaxonDetail&page=iprGeneList&ext_accession=IPR001128&taxon_oid=2563366593 | [CP006567](https://www.ncbi.nlm.nih.gov/nuccore/CP006567) |
| *Streptomyces albulus* NK660 | salu | https://img.jgi.doe.gov/cgi-bin/m/main.cgi?section=TaxonDetail&page=taxonDetail&taxon_oid=2579778836 | [CP007574](https://www.ncbi.nlm.nih.gov/nuccore/CP007574) |
| *Streptomyces albus* ZPM | sall | https://img.jgi.doe.gov/cgi-bin/m/main.cgi?section=TaxonDetail&page=taxonDetail&taxon_oid=2639762896 | [CP006871](https://www.ncbi.nlm.nih.gov/nuccore/CP006871) |
| *Streptomyces lividans* | slv | https://img.jgi.doe.gov/cgi-bin/m/main.cgi?section=TaxonDetail&page=iprGeneList&ext_accession=IPR001128&taxon_oid=2597490034 | [CP009124](https://www.ncbi.nlm.nih.gov/nuccore/CP009124) |
| *Streptomyces glaucescens* | sgu | https://img.jgi.doe.gov/cgi-bin/m/main.cgi?section=TaxonDetail&page=taxonDetail&taxon_oid=2627854123 | [CP009438](https://www.ncbi.nlm.nih.gov/nuccore/CP009438) |
| *Streptomyces vietnamensis* | svt | https://img.jgi.doe.gov/cgi-bin/m/main.cgi?section=TaxonDetail&page=iprGeneList&ext_accession=IPR001128&taxon_oid=2627853829 | [CP010407](https://www.ncbi.nlm.nih.gov/nuccore/CP010407) |
| *Streptomyces* sp. 769 | stre | https://img.jgi.doe.gov/cgi-bin/m/main.cgi?section=TaxonDetail&page=iprGeneList&ext_accession=IPR001128&taxon_oid=2627853736 | [CP003987](https://www.ncbi.nlm.nih.gov/nuccore/CP003987) |
| *Streptomyces cyaneogriseus* | scw | https://img.jgi.doe.gov/cgi-bin/m/main.cgi?section=TaxonDetail&page=taxonDetail&taxon_oid=2648501341 | [CP010849](https://www.ncbi.nlm.nih.gov/nuccore/CP010849) |
| *Streptomyces lydicus* A02 | sld | https://img.jgi.doe.gov/cgi-bin/m/main.cgi?section=TaxonDetail&page=iprGeneList&ext_accession=IPR001128&taxon_oid=2651869762 | [CP007699](https://www.ncbi.nlm.nih.gov/nuccore/CP007699) |
| *Streptomyces xiamenensis* 318 | sxi | https://img.jgi.doe.gov/cgi-bin/m/main.cgi?section=TaxonDetail&page=iprGeneList&ext_accession=IPR001128&taxon_oid=2630968921 | [CP009922](https://www.ncbi.nlm.nih.gov/nuccore/CP009922) |
| *Streptomyces* sp. Mg1 | strm | https://img.jgi.doe.gov/cgi-bin/m/main.cgi?section=TaxonDetail&page=iprGeneList&ext_accession=IPR001128&taxon_oid=2579778547 | [CP011664](https://www.ncbi.nlm.nih.gov/nuccore/CP011664) |
| *Streptomyces* sp. CNQ-509 | strc | https://img.jgi.doe.gov/cgi-bin/m/main.cgi?section=TaxonDetail&page=iprGeneList&ext_accession=IPR001128&taxon_oid=2654587513 | [CP011492](https://www.ncbi.nlm.nih.gov/nuccore/CP011492) |
| *Streptomyces ambofaciens* | samb | https://img.jgi.doe.gov/cgi-bin/m/main.cgi?section=TaxonDetail&page=taxonDetail&taxon_oid=2654588008 | [CP012382](https://www.ncbi.nlm.nih.gov/nuccore/CP012382) |
| *Streptomyces pristinaespiralis* HCCB 10218 | spri | <http://www.genome.jp/kegg-bin/show_organism?org=spri> | [CP011340](https://www.ncbi.nlm.nih.gov/nuccore/CP011340) |
| *Streptomyces* sp. CFMR 7 | scz | https://img.jgi.doe.gov/cgi-bin/m/main.cgi?section=TaxonDetail&page=iprGeneList&ext_accession=IPR001128&taxon_oid=2654587638 | [CP011522](https://www.ncbi.nlm.nih.gov/nuccore/CP011522) |
| *Streptomyces* sp. CdTB01 | scx | https://img.jgi.doe.gov/cgi-bin/m/main.cgi?section=TaxonDetail&page=iprGeneList&ext_accession=IPR001128&taxon_oid=2687453384 | [CP013743](https://www.ncbi.nlm.nih.gov/nuccore/CP013743) |
| *Streptomyces reticuli* | srw | https://img.jgi.doe.gov/cgi-bin/m/main.cgi?section=TaxonDetail&page=iprGeneList&ext_accession=IPR001128&taxon_oid=2687453570 | [LN997842](https://www.ncbi.nlm.nih.gov/nuccore/LN997842) |
| *Streptomyces* sp. 4F | strf | https://img.jgi.doe.gov/cgi-bin/m/main.cgi?section=TaxonDetail&page=iprGeneList&ext_accession=IPR001128&taxon_oid=2687453197 | [CP013142](https://www.ncbi.nlm.nih.gov/nuccore/CP013142) |
| *Streptomyces leeuwenhoekii* C34(2013) | sle | https://img.jgi.doe.gov/cgi-bin/m/main.cgi?section=TaxonDetail&page=iprGeneList&ext_accession=IPR001128&taxon_oid=2582581028 | [LN831790](https://www.ncbi.nlm.nih.gov/nuccore/LN831790) |
| *Streptomyces rubrolavendulae* | srn | https://img.jgi.doe.gov/cgi-bin/m/main.cgi?section=TaxonDetail&page=iprGeneList&ext_accession=IPR001128&taxon_oid=2765235980 | [CP017316](https://www.ncbi.nlm.nih.gov/nuccore/CP017316) |
| *Streptomyces parvulus* | spav | https://img.jgi.doe.gov/cgi-bin/m/main.cgi?section=TaxonDetail&page=iprGeneList&ext_accession=IPR001128&taxon_oid=2718218336 | [CP015866](https://www.ncbi.nlm.nih.gov/nuccore/CP015866) |
| *Streptomyces lydicus* 103 | slc | https://img.jgi.doe.gov/cgi-bin/m/main.cgi?section=TaxonDetail&page=iprGeneList&ext_accession=IPR001128&taxon_oid=2718217986 | [CP017157](https://www.ncbi.nlm.nih.gov/nuccore/CP017157) |
| *Streptomyces* sp. SAT1 | strt | https://img.jgi.doe.gov/cgi-bin/m/main.cgi?section=TaxonDetail&page=iprGeneList&ext_accession=IPR001128&taxon_oid=2718218381 | [CP015849](https://www.ncbi.nlm.nih.gov/nuccore/CP015849) |
| *Streptomyces clavuligerus* | sclf | https://img.jgi.doe.gov/cgi-bin/m/main.cgi?section=TaxonDetail&page=taxonDetail&taxon_oid=647533233 | [CP016559](https://www.ncbi.nlm.nih.gov/nuccore/CP016559) |
| *Streptomyces griseochromogenes* | sgs | https://img.jgi.doe.gov/cgi-bin/m/main.cgi?section=TaxonDetail&page=iprGeneList&ext_accession=IPR001128&taxon_oid=2684622604 | [CP016279](https://www.ncbi.nlm.nih.gov/nuccore/CP016279) |
| *Streptomyces* sp. S10(2016) | stsi | https://img.jgi.doe.gov/cgi-bin/m/main.cgi?section=TaxonDetail&page=iprGeneList&ext_accession=IPR001128&taxon_oid=2687453302 | [CP015098](https://www.ncbi.nlm.nih.gov/nuccore/CP015098) |
| *Streptomyces lincolnensis* | sls | <http://www.genome.jp/kegg-bin/show_organism?org=sls> | [CP016438](https://www.ncbi.nlm.nih.gov/nuccore/CP016438) |
| *Streptomyces noursei* | snr | https://img.jgi.doe.gov/cgi-bin/m/main.cgi?section=TaxonDetail&page=iprGeneList&ext_accession=IPR001128&taxon_oid=2602042089 | [CP011533](https://www.ncbi.nlm.nih.gov/nuccore/CP011533) |

**Table S3. Information on mycobacterial species used in the study.** Species names, codes, and genome IDs (from NCBI) were listed in the table.

| Species name | Species code | Genome ID |
| --- | --- | --- |
| *Mycobacterium africanum* GM041182 | MAF | FR878060 |
| *Mycobacterium tuberculosis* C | MTO | CP002992 |
| *Mycobacterium tuberculosis* F11 | MTF | CP000717 |
| *Mycobacterium tuberculosis* H37Ra | MRA | CP000611 |
| *Mycobacterium tuberculosis* H37Rv | MTU | NC_000962 |
| *Mycobacterium tuberculosis* Haarlem | MTUL | CP001664 |
| *Mycobacterium tuberculosis* KZN 1435 | MTB | CP001658 |
| *Mycobacterium tuberculosis* KZN 605 | MTZ | CP001976 |
| *Mycobacterium tuberculosis* KZN 4207 | MTK | CP001662 |
| *Mycobacterium tuberculosis* RGTB327 | MTG | CP003233 |
| *Mycobacterium tuberculosis* CDC1551 | MTC | AE000516 |
| *Mycobacterium tuberculosis* strains CCDC5079 | MTE | CP001641 |
| *Mycobacterium tuberculosis* 7199-99 | MTUB | HE663067 |
| *Mycobacterium tuberculosis* Beijing/NITR203 | MTJ | CP005082 |
| *Mycobacterium tuberculosis* CAS/NITR204 | MTUC | CP005386 |
| *Mycobacterium tuberculosis* EAI5 | MTX | CP006578 |
| *Mycobacterium tuberculosis* EAI5/NITR206 | MTUH | CP004886 |
| *Mycobacterium tuberculosis* Erdman=ATCC 35801 | MTN | AP012340 |
| *Mycobacterium tuberculosis* UT205 | MTD | HE608151 |
| *Mycobacterium canetti* CIPT 140010059 | MCE | HE572590 |
| *Mycobacterium canetti* CIPT 140060008 | MCQ | FO203507 |
| *Mycobacterium canetti* CIPT 140710010 | MCX | FO203509 |
| *Mycobacterium bovis* AF 2122/97 | MBO | NC_002945 |
| *Mycobacterium bovis* BCG Pasteur 1173P2 | MBB | AM408590 |
| *Mycobacterium bovis* BCG Korea 1168P | MBK | CP003900 |
| *Mycobacterium bovis* BCG Mexico | MBM | CP002095 |
| *Mycobacterium bovis* BCG Toyko 172 | MBT | AP010918 |
| *Mycobacterium abscessus* ATCC 19977 | MAB | NC_010397 |
| *Mycobacterium abscessus* subsp.*bolletti* 50594 | MABB | CP004374 |
| *Mycobacterium abscessus* 47J26 | MABL | AP014547 |
| *Mycobacterium abscessus* 103 | MAZ | CP009408 |
| *Mycobacterium abscessus* subsp.*bolletti* MA 1948 | MAY | CP009408 |
| *Mycobacterium abscessus* VO6705 | MYS | CP009615 |
| *Mycobacterium Avium* 104 | MAV | CP000479 |
| *Mycobacterium Avium* subsp.*paratuberculosis* K10 | MPA | AE016958 |
| *Mycobacterium avium* subsp.*paratuberculosis* MAP4 | MAO | CP005928 |
| *Mycobacterium intracellulare* ATCC 13950 | MIA | CP003322 |
| *Mycobacterium intracellulare* MOTT-02 | MIT | CP003323 |
| *Mycobacterium intracellulare* MOTT-64 | MIE | CP009499 |
| *Mycobacterium intracellulare* MOTT-36Y | MMM | CP003491 |
| *Mycobacterium indicus pranii* MTCC 9506 | MID | CP002275 |
| *Mycobacterium* sp.JDM601 | MYV | CP009914 |
| *Mycobacterium liflandi* 128FXT | MLI | CP003899 |
| *Mycobacterium marinum* | MMAE | HG917972 |
| *Mycobacterium massiliense* | MMAL | CP023147 |
| *Mycobacterium kansassi* ATCC 12478 | MMV | CP003699 |
| *Mycobacterium vanbaalenii* PYR-1 | MVA | CP000511 |
| *Mycobacterium smegmatis* MC2 155 | MSB | CP009494 |
| *Mycobacterium chubuense* NBB4 | MCB | CP003053 |
| *Mycobacterium gilvum* PYR-GCK | MGI | CP000656 |
| *Mycobacterium gilvum* Spyr1 | MSP | CP002385 |
| *Mycobacterium smegmatis* JS623 | MSA | CP003078 |
| *Mycobacterium rhodesiae* | MRH | CP003169 |
| *Mycobacterium neoaurum* VKM Ac-18150 | MNE | CP006936 |

**Table S4. P450 family and subfamily level comparative analysis of P450s in 48 *Streptomyces* species.** Open reading frames (ORFs) for each species were obtained from the genome database listed in Table S2. The percentage of P450s was calculated considering ORFs as 100%.

| **Species name** | **Species code** | **No. of P450s** | **No. of P450 families** | **No. of P450 subfamilies** | **ORFs** | **% of P450s** |
| --- | --- | --- | --- | --- | --- | --- |
| *Streptomyces albulus* ZPM | sall | 69 | 27 | 52 | 8191 | 0.8 |
| *Streptomyces albulus* NK660 | salu | 64 | 27 | 50 | 8086 | 0.8 |
| *Streptomyces noursei* | snr | 64 | 26 | 52 | 8691 | 0.7 |
| *Streptomyces violaceusniger* | svl | 51 | 16 | 42 | 8985 | 0.6 |
| *Streptomyces bingchenggensis* | sbh | 50 | 26 | 44 | 10022 | 0.5 |
| *Streptomyces rapamycinicus* | src | 63 | 23 | 56 | 10002 | 0.6 |
| *Streptomyces cattleya* NRRL 8057 = DSM 46488 | scy | 42 | 21 | 39 | 7569 | 0.6 |
| *Streptomyces* sp. 769 | stre | 59 | 24 | 49 | 9553 | 0.6 |
| *Streptomyces hygroscopicus* subsp. *jinggangensis* 5008 | shy | 38 | 18 | 33 | 9108 | 0.4 |
| *Streptomyces cattleya* NRRL 8057 = DSM 46488 | sct | 41 | 20 | 38 | 7475 | 0.5 |
| *Streptomyces hygroscopicus* subsp. *jinggangensis* TL01 | sho | 37 | 18 | 33 | 8878 | 0.4 |
| *Streptomyces avermitilis* | sma | 53 | 24 | 45 | 7676 | 0.7 |
| *Streptomyces collinus* | sci | 34 | 16 | 27 | 7113 | 0.5 |
| *Streptomyces lydicus* | sld | 40 | 19 | 35 | 8888 | 0.5 |
| *Streptomyces* sp. Mg1 | strm | 37 | 21 | 36 | 7117 | 0.5 |
| *Streptomyces leeuwenhoekii* | sle | 36 | 17 | 34 | 7005 | 0.5 |
| *Streptomyces pratensis* | sfa | 29 | 16 | 26 | 6572 | 0.4 |
| *Streptomyces reticuli* | srw | 47 | 26 | 43 | 8090 | 0.6 |
| *Streptomyces griseus* | sgr | 28 | 13 | 24 | 7136 | 0.4 |
| *Streptomyces* sp. PAMC 26508 | strp | 29 | 16 | 26 | 7073 | 0.4 |
| *Streptomyces* sp. SirexAA-E | ssx | 24 | 10 | 22 | 6357 | 0.4 |
| *Streptomyces davawensis* | sdv | 32 | 19 | 30 | 8616 | 0.4 |
| *Streptomyces cyaneogriseus* | scw | 33 | 16 | 30 | 5830 | 0.6 |
| *Streptomyces lincolnensis* | sls | 24 | 15 | 23 | 8590 | 0.3 |
| *Streptomyces pristinaespiralis* | spri | 23 | 12 | 18 | 7352 | 0.3 |
| *Streptomyces venezuelae* | sve | 23 | 16 | 21 | 7453 | 0.3 |
| *Streptomyces* sp. CFMR 7 | scz | 24 | 13 | 20 | 6716 | 0.4 |
| *Streptomyces vietnamensis* | svt | 30 | 20 | 29 | 7356 | 0.4 |
| *Streptomyces xiamenensis* | sxi | 20 | 12 | 19 | 5484 | 0.4 |
| *Streptomyces coelicolor* | sco | 18 | 10 | 17 | 8152 | 0.2 |
| *Streptomyces albus* J1074 | salb | 18 | 9 | 18 | 5832 | 0.3 |
| *Streptomyces ambofaciens* | samb*^#^* | 19 | 10 | 18 | 7793 | 0.2 |
| *Streptomyces lividans* | slv | 20 | 10 | 18 | 7360 | 0.3 |
| *Streptomyces scabiei* | scb | 30 | 16 | 30 | 8746 | 0.3 |
| *Streptomyces glaucescens* | sgu | 18 | 11 | 17 | 6567 | 0.3 |
| *Streptomyces albus* DSM 41398 | sals | 25 | 13 | 24 | 7330 | 0.3 |
| *Streptomyces fulvissimus* | sfi | 19 | 10 | 16 | 6925 | 0.3 |
| *Streptomyces* sp. CNQ-509 | strc | 16 | 11 | 16 | 6407 | 0.2 |
| *Streptomyces rubrolavendulae* | srn | 20 | 12 | 19 | 5425 | 0.4 |
| *Streptomyces clavuligerus* | sclf | 65 | 30 | 58 | 5981 | 1.1 |
| *Streptomyces griseochromogenes* | sgs | 46 | 24 | 40 | 9064 | 0.5 |
| *Streptomyces* sp. S10(2016) | stsi | 20 | 15 | 20 | 7661 | 0.3 |
| *Streptomyces globisporus* | sgb | 23 | 13 | 19 | 6654 | 0.3 |
| *Streptomyces* sp. CdTB01^#^ | scx | 26 | 17 | 25 | 8743 | 0.3 |
| *Streptomyces parvulus* | spav | 25 | 15 | 25 | 6714 | 0.4 |
| *Streptomyces lydicus* | slc | 32 | 13 | 29 | 6872 | 0.5 |
| *Streptomyces* sp. SAT1 | strt | 25 | 15 | 22 | 6110 | 0.4 |
| *Streptomyces* sp. 4F | strf | 16 | 11 | 15 | 6792 | 0.2 |

#, Two and one false positives P450 fragments were identified in *Streptomyces ambofaciens* ATCC 23877 and *Streptomyces* sp. CdTB01, respectively. These P450 fragments were not taken into final count.

**Table S5: New P450 families and new P450 subfamilies identified in *Streptomyces* species.**

| **New families** | | **Total number** |
| --- | --- | --- |
| CYP1035-CYP1039; CYP1047; CYP1048; CYP1053-CYP1059; CYP1060-CYP1066; CYP1189-CYP1194; CYP1196-CYP1199; CYP1251; CYP1274; CYP1416-CYP1420; CYP1422-CYP1424; CYP154; CYP155-CYP159; CYP161; CYP1618; CYP170; CYP171; CYP178-CYP184; CYP2045; CYP208; CYP2108; CYP211; CYP2189; CYP2238; CYP246; CYP282; CYP283 | | 66 |
| **New subfamilies (144)** | | |
| **P450 family** | **Total number of new subfamilies** | **Name of the subfamily** |
| CYP102 | 2 | B, G |
| CYP105 | 38 | AT, AU, AV, AW, AX, AY, AZ, B, BA, BB, BC, BD, BE, BF, BG, BH, BQ, BR, BS, BT, BU, BV, BW, D, DB, DC, DD, DE, DF, DG, H, M, N, P, Q, R, U, Z |
| CYP107 | 62 | AD, AK, AL, AM, BK, BW, BY, BZ, CD-CH, CJ, CK-CN, CP-CW, DU, DW, EA-EH, EJ, EK-EN, EP-ER, KW-KZ, LA-LG, P, T-Y |
| CYP112 | 1 | B |
| CYP113 | 4 | G, J, Y, Z |
| CYP1222 | 1 | D |
| CYP1223 | 1 | B |
| CYP123 | 1 | D |
| CYP1240 | 1 | B |
| CYP135 | 2 | D, G |
| CYP136 | 2 | E, F |
| CYP143 | 1 | C |
| CYP145 | 3 | B, C, D |
| CYP147 | 2 | B, K |
| CYP152 | 1 | D |
| CYP166 | 1 | C |
| CYP186 | 1 | D |
| CYP251 | 3 | E, F, G |
| CYP1005 | 1 | B |
| CYP1278 | 1 | B |
| CYP1339 | 1 | B |
| CYP1341 | 1 | E |
| CYP162 | 1 | C |
| CYP163 | 4 | C, D, G, H, |
| CYP1995 | 2 | B, C |
| CYP199 | 1 | R |
| CYP274 | 2 | B, C |
| CYP285 | 2 | B, D |
| CYP298 | 1 | B |

**Table S6: Comparative analysis of P450 family members in *Streptomyces* species.** The percentage contribution of each P450 family is calculated as percentage contribution to the total number of P450s (1625 P450s) identified in 48 *Streptomyces* species.

| **P450 family** | **Number of P450s** | **% contribution to total number of P450s** |
| --- | --- | --- |
| CYP107 | 381 | 23.4 |
| CYP105 | 225 | 13.8 |
| CYP157 | 123 | 7.6 |
| CYP154 | 119 | 7.3 |
| CYP156 | 56 | 3.4 |
| CYP147 | 52 | 3.2 |
| CYP183 | 48 | 3.0 |
| CYP102 | 45 | 2.8 |
| CYP159 | 37 | 2.3 |
| CYP125 | 33 | 2.0 |
| CYP158 | 31 | 1.9 |
| CYP163 | 28 | 1.7 |
| CYP180 | 27 | 1.7 |
| CYP113 | 22 | 1.4 |
| CYP170 | 21 | 1.3 |
| CYP1035 | 20 | 1.2 |
| CYP161 | 15 | 0.9 |
| CYP184 | 14 | 0.9 |
| CYP124 | 13 | 0.8 |
| CYP1047 | 11 | 0.7 |
| CYP152 | 11 | 0.7 |
| CYP1038 | 10 | 0.6 |
| CYP1199 | 10 | 0.6 |
| CYP155 | 10 | 0.6 |
| CYP285 | 10 | 0.6 |
| CYP1005 | 8 | 0.5 |
| CYP182 | 8 | 0.5 |
| CYP1060 | 7 | 0.4 |
| CYP251 | 7 | 0.4 |
| CYP1043 | 6 | 0.4 |
| CYP1064 | 6 | 0.4 |
| CYP1240 | 6 | 0.4 |
| CYP1046 | 5 | 0.3 |
| CYP108 | 5 | 0.3 |
| CYP121 | 5 | 0.3 |
| CYP162 | 5 | 0.3 |
| CYP1029 | 4 | 0.2 |
| CYP1189 | 4 | 0.2 |
| CYP145 | 4 | 0.2 |
| CYP165 | 4 | 0.2 |
| CYP166 | 4 | 0.2 |
| CYP179 | 4 | 0.2 |
| CYP208 | 4 | 0.2 |
| CYP245 | 4 | 0.2 |
| CYP247 | 4 | 0.2 |
| CYP1031 | 3 | 0.2 |
| CYP1037 | 3 | 0.2 |
| CYP1278 | 3 | 0.2 |
| CYP134 | 3 | 0.2 |
| CYP1419 | 3 | 0.2 |
| CYP1423 | 3 | 0.2 |
| CYP171 | 3 | 0.2 |
| CYP178 | 3 | 0.2 |
| CYP1013 | 2 | 0.1 |
| CYP1036 | 2 | 0.1 |
| CYP1048 | 2 | 0.1 |
| CYP1061 | 2 | 0.1 |
| CYP1062 | 2 | 0.1 |
| CYP116 | 2 | 0.1 |
| CYP1190 | 2 | 0.1 |
| CYP1191 | 2 | 0.1 |
| CYP1192 | 2 | 0.1 |
| CYP1197 | 2 | 0.1 |
| CYP1198 | 2 | 0.1 |
| CYP1251 | 2 | 0.1 |
| CYP130 | 2 | 0.1 |
| CYP1341 | 2 | 0.1 |
| CYP135 | 2 | 0.1 |
| CYP136 | 2 | 0.1 |
| CYP140 | 2 | 0.1 |
| CYP1416 | 2 | 0.1 |
| CYP1417 | 2 | 0.1 |
| CYP1420 | 2 | 0.1 |
| CYP143 | 2 | 0.1 |
| CYP1453 | 2 | 0.1 |
| CYP1530 | 2 | 0.1 |
| CYP1618 | 2 | 0.1 |
| CYP181 | 2 | 0.1 |
| CYP186 | 2 | 0.1 |
| CYP194 | 2 | 0.1 |
| CYP1995 | 2 | 0.1 |
| CYP199 | 2 | 0.1 |
| CYP2027 | 2 | 0.1 |
| CYP211 | 2 | 0.1 |
| CYP244 | 2 | 0.1 |
| CYP253 | 2 | 0.1 |
| CYP274 | 2 | 0.1 |
| CYP1039 | 1 | 0.1 |
| CYP1041 | 1 | 0.1 |
| CYP1042 | 1 | 0.1 |
| CYP1053 | 1 | 0.1 |
| CYP1054 | 1 | 0.1 |
| CYP1055 | 1 | 0.1 |
| CYP1056 | 1 | 0.1 |
| CYP1057 | 1 | 0.1 |
| CYP1058 | 1 | 0.1 |
| CYP1059 | 1 | 0.1 |
| CYP1063 | 1 | 0.1 |
| CYP1065 | 1 | 0.1 |
| CYP1066 | 1 | 0.1 |
| CYP1113 | 1 | 0.1 |
| CYP112 | 1 | 0.1 |
| CYP1151 | 1 | 0.1 |
| CYP1193 | 1 | 0.1 |
| CYP1194 | 1 | 0.1 |
| CYP1196 | 1 | 0.1 |
| CYP1207 | 1 | 0.1 |
| CYP1215 | 1 | 0.1 |
| CYP1222 | 1 | 0.1 |
| CYP1223 | 1 | 0.1 |
| CYP122 | 1 | 0.1 |
| CYP123 | 1 | 0.1 |
| CYP1248 | 1 | 0.1 |
| CYP1274 | 1 | 0.1 |
| CYP1339 | 1 | 0.1 |
| CYP1373 | 1 | 0.1 |
| CYP1385 | 1 | 0.1 |
| CYP1392 | 1 | 0.1 |
| CYP1418 | 1 | 0.1 |
| CYP1422 | 1 | 0.1 |
| CYP1424 | 1 | 0.1 |
| CYP1457 | 1 | 0.1 |
| CYP1459 | 1 | 0.1 |
| CYP1469 | 1 | 0.1 |
| CYP146 | 1 | 0.1 |
| CYP1509 | 1 | 0.1 |
| CYP151 | 1 | 0.1 |
| CYP1694 | 1 | 0.1 |
| CYP1722 | 1 | 0.1 |
| CYP177 | 1 | 0.1 |
| CYP1813 | 1 | 0.1 |
| CYP2045 | 1 | 0.1 |
| CYP206 | 1 | 0.1 |
| CYP2108 | 1 | 0.1 |
| CYP2189 | 1 | 0.1 |
| CYP2238 | 1 | 0.1 |
| CYP2266 | 1 | 0.1 |
| CYP228 | 1 | 0.1 |
| CYP2340 | 1 | 0.1 |
| CYP246 | 1 | 0.1 |
| CYP268 | 1 | 0.1 |
| CYP282 | 1 | 0.1 |
| CYP283 | 1 | 0.1 |
| CYP298 | 1 | 0.1 |

**Table S7: Comparative analysis of secondary metabolite BGCs between the genera *Streptomyces* and *Mycobacterium*.**

| **Secondary metabolite BGC** | **Number of clusters** | |
| --- | --- | --- |
|  | ***Streptomyces*** | ***Mycobacterium*** |
| Nrps | 117 | 177 |
| Other | 94 | 159 |
| T1pks | 94 | 149 |
| T1pks-Nrps | 53 | 146 |
| Bacteriocin | 76 | 71 |
| Terpene | 229 | 60 |
| T3pks | 41 | 58 |
| T3pks-T1pks | 0 | 38 |
| Ectoine | 50 | 15 |
| Ectoine-T1pks-Nrps | 0 | 5 |
| T2pks | 41 | 4 |
| T2pks-nrps | 2 | 4 |
| Arylpolyene | 3 | 4 |
| Lantipeptide | 87 | 3 |
| Amglyccycl | 7 | 2 |
| T1pks-Ectoine-Nrps | 0 | 1 |
| otherKs | 8 | 1 |
| linaridin | 3 | 1 |
| Siderophore | 121 | Not identified |
| Butyrolactone | 58 | Not identified |
| Melanin | 40 | Not identified |
| Lassopeptide | 35 | Not identified |
| Thiopeptide | 21 | Not identified |
| T1pks-Otherks | 13 | Not identified |
| Indole | 9 | Not identified |
| Bacteriocin-Lantipeptide | 8 | Not identified |
| Transatpks-T1pks-Nrps | 8 | Not identified |
| Bacteriocin-Nrps | 8 | Not identified |
| T3pks-Nrps | 7 | Not identified |
| Terpene-Nrps | 7 | Not identified |
| Lantipeptide-Nrps | 5 | Not identified |
| Melanin-Nrps | 5 | Not identified |
| T2pks-Terpene | 5 | Not identified |
| Ladderane | 5 | Not identified |
| Terpene-T1pks | 5 | Not identified |
| Transatpks-Nrps | 5 | Not identified |
| Nrps-T1pks-Otherks | 4 | Not identified |
| Lantipeptide-T1pks-Nrps | 4 | Not identified |
| T1pks-Butyrolactone-Nrps | 4 | Not identified |
| T2pks-Otherks | 4 | Not identified |
| Lantipeptide-Terpene | 4 | Not identified |
| T2pks-Butryolactone | 4 | Not identified |
| Melanin-Terpene | 4 | Not identified |
| T3pks-Terpene-Nrps | 3 | Not identified |
| T2pks-T1pks-Otherks | 3 | Not identified |
| Ladderane-Arylpolyene-Nrps | 3 | Not identified |
| Arylpolyene-Ladderane | 3 | Not identified |
| Bacteriocin-T1pks-Nrps | 3 | Not identified |
| T1pks-Siderophore | 3 | Not identified |
| Blactam | 3 | Not identified |
| Otherks-Nrps | 3 | Not identified |
| Lantipeptide-Terpene-Nrps | 3 | Not identified |
| T3pks-T1pks-Nrps | 3 | Not identified |
| Nucleoside | 3 | Not identified |
| T1pks-Butyrolactone-otherks | 2 | Not identified |
| Butyrolactone-Otherks | 2 | Not identified |
| T1pks-Linaridin | 2 | Not identified |
| Hserlactone | 2 | Not identified |
| T1pks-Lassopeptide | 2 | Not identified |
| T3pks-Terpene | 2 | Not identified |
| Nrps-Otherks | 2 | Not identified |
| Blactam-T1pks-Nrps | 2 | Not identified |
| Phenazine-Nrps | 2 | Not identified |
| T2pks-Ladderane-Nrps | 2 | Not identified |
| T2pks-T1pks | 2 | Not identified |
| Bacteriocin-Terpene | 2 | Not identified |
| Lassopeptide-Nrps | 2 | Not identified |
| Lantipeptide-Lassopeptide | 2 | Not identified |
| T2pks-Oligosaccharide-Nucleoside-Nrps | 2 | Not identified |
| Butyrolactone-T1pks-Nrps | 2 | Not identified |
| Terpene-T3pks-T1pks-Nrps | 2 | Not identified |
| T2pks-Oligosaccharide-Nrps-Otherks | 2 | Not identified |
| Terpene-T1pks-Nrps | 2 | Not identified |
| Bacteriocin-T1pks | 2 | Not identified |
| Phosphoglycolipid | 2 | Not identified |
| Thiopeptide-Bacteriocin | 2 | Not identified |
| T1pks-Butyrolactone | 1 | Not identified |
| T1pks-Terpene | 1 | Not identified |
| Transatpks-T1pks-Otherks-Nrps | 1 | Not identified |
| Bacteriocin-Bottromycin | 1 | Not identified |
| Butyrolactone-T1pks-Otherks | 1 | Not identified |
| Indole-T1pks | 1 | Not identified |
| T2pks-Butyrolactone-Nrps | 1 | Not identified |
| Bacteriocin-Lantipeptide-T1pks-Otherks-Nrps | 1 | Not identified |
| Ectoine-Terpene | 1 | Not identified |
| Transatpks-Otherks-Nrps | 1 | Not identified |
| Bacteriocin-Lantipeptide-T1pks | 1 | Not identified |
| Nrps-Furan-T1pks-Otherks | 1 | Not identified |
| Siderophore-Nrps | 1 | Not identified |
| T2pks-Oligosaccharide-Otherks | 1 | Not identified |
| Transatpks-Terpene-Nrps | 1 | Not identified |
| Ladderane-Nrps | 1 | Not identified |
| Butyrolactone-Terpene | 1 | Not identified |
| Bacteriocin-Oligosaccharide | 1 | Not identified |
| Terpene-T3pks-Cyanobactin-Nrps | 1 | Not identified |
| Phosphonate | 1 | Not identified |
| Arylpolyene-Nrps | 1 | Not identified |
| Melanin-Arylpolyene-Ladderane | 1 | Not identified |
| T1pks-Phenazine | 1 | Not identified |
| Terpene-Ectoine | 1 | Not identified |
| Bacteriocin-Otherks | 1 | Not identified |
| Nrps-Transatpks-T1pks-Otherks | 1 | Not identified |
| Thiopeptide-T2pks-Otherks-Nrps | 1 | Not identified |
| Nrps-Ladderane | 1 | Not identified |
| Lantipeptide-Lassopeptide-Nrps | 1 | Not identified |
| T1pks-Arylpolyene-Ladderane | 1 | Not identified |
| Bacteriocin-Nrps-Lantipeptide-T1pks-Otherks | 1 | Not identified |
| Nrps-Arylpolyene-Ladderane | 1 | Not identified |
| T3pks-Otherks-Butyrolactone-Nrps | 1 | Not identified |
| Otherks-Butyrolactone-Nrps | 1 | Not identified |
| Butyrolactone-T1pks | 1 | Not identified |
| T2pks-Lantipeptide-Terpene | 1 | Not identified |
| T1pks-Arylpolyene | 1 | Not identified |
| Bacteriocin-Butyrolactone | 1 | Not identified |
| Thiopeptide-T1pks | 1 | Not identified |
| T3pks-Fused-Nrps | 1 | Not identified |
| Transatpks | 1 | Not identified |
| Cyanobactin-T1pks-Nrps | 1 | Not identified |
| T2pks-Oligosaccharide | 1 | Not identified |
| Lantipeptide-T1pks | 1 | Not identified |
| Lantipeptide-Linaridin | 1 | Not identified |
| Linaridin-T1pks-Lassopeptide-Nrps | 1 | Not identified |
| Terpene-Otherks | 1 | Not identified |
| T2pks-T3pks | 1 | Not identified |
| T1pks-Transatpks-Terpene | 1 | Not identified |
| Fused | 1 | Not identified |
| T3pks-Terpene-Otherks | 1 | Not identified |
| Phenazine | 1 | Not identified |
| T1pks-Terpene-Nrps | 1 | Not identified |
| Oligosaccharide-T1pks-Nrps | 1 | Not identified |
| Oligosaccharide-Ectoine-T2pks-Nrps-T1pks-Otherks | 1 | Not identified |
| Thiopeptide-Nrps | 1 | Not identified |
| Thiopeptide-T2pks | 1 | Not identified |
| Ectoine-Nrps | 1 | Not identified |
| T2pks-Ectoine-Otherks | 1 | Not identified |
| Thiopeptide-T1pks-Nrps | 1 | Not identified |
| Ectoine-Butyrolactone | 1 | Not identified |
| Transatpks-T1pks-Otherks-Nrps | 1 | Not identified |
| Bacteriocin-Lassopeptide-Nrps | 1 | Not identified |
| T2pks-T3pks-Otherks | 1 | Not identified |
| Ectoine-T1pks-Otherks | 1 | Not identified |
| Butyrolactone-Amglyccycl-T1pks-Nrps | 1 | Not identified |
| Nrps-T2pks-Otherks-T1pks-Phenazine | 1 | Not identified |
| Nrps-Transatpks-Terpene-Otherks | 1 | Not identified |
| Butyrolactone-Amglyccycl | 1 | Not identified |
| T3pks-Butyrolactone | 1 | Not identified |
| Nucleoside-Lassopeptide-Nrps | 1 | Not identified |
| Phosphonate-Nrps | 1 | Not identified |
| Thiopeptide-Terpene | 1 | Not identified |
| Bacteriocin-Terpene-Nrps | 1 | Not identified |
| Indole-Terpene-Nrps | 1 | Not identified |
| Blactam-Nrps | 1 | Not identified |
| Phosphonate-Bacteriocin | 1 | Not identified |
| Siderophore-T1pks-Nrps | 1 | Not identified |
| Phosphonate-Terpene | 1 | Not identified |
| Lantipeptide-T1pks-Otherks | 1 | Not identified |
| Phosphonate-T3pks-Nrps-Ladderane | 1 | Not identified |
| Indole-T3pks | 1 | Not identified |
| Amglyccycl-Butyrolactone | 1 | Not identified |
| Bacteriocin-T2pks | 1 | Not identified |
| Terpene-Butyrolactone | 1 | Not identified |
| Nrps-Siderophore | 1 | Not identified |

**Table S8. Comparative analysis of secondary metabolite biosynthetic gene clusters and P450s that are part of these clusters in *Streptomyces* species.**

| **Species code** | **Cluster name** | **Number of P450s** | **P450 name** |
| --- | --- | --- | --- |
| sco | T3pks | 1 | CYP158A2 |
|  | Terpene | 1 | CYP170A1 |
|  | T3pks-Terpene-Nrps | 1 | CYP105N1 |
| sma | T1pks | 2 | CYP105D6; CYP105P1 |
|  | Nrps | 1 | CYP147B1 |
|  | Nrps-T1pks-Otherks | 2 | CYP178A1; CYP178A3P |
|  | T1pks | 1 | CYP171A1 |
|  | Terpene | 1 | CYP180A1 |
|  | T2pks-T1pks-Otherks | 2 | CYP107Y1; CYP181A1 |
|  | T1pks | 2 | CYP107W1; CYP105B23 |
|  | Terpene | 1 | CYP183A1 |
|  | Terpene | 1 | CYP170A2 |
|  | Butyrolactone-Otherks | 2 | CYP107V1; CYP107U2 |
|  | T3pks | 1 | CYP158A3 |
|  | T1pks | 1 | CYP105R1 |
| sgr | T1pks-Nrps | 1 | CYP105D1 |
|  | Melanin | 1 | CYP124G2 |
|  | T1pks-Nrps | 3 | CYP162C1; CYP208A1; CYP154M2 |
|  | T1pks-Nrps | 1 | CYP107BX5 |
|  | Nrps | 1 | CYP107BY1 |
|  | Ladderane-Arylpolyene-Nrps | 2 | CYP163B5; CYP107BZ1 |
|  | T3pks | 1 | CYP107F4 |
|  | Transatpks-T1pks-Otherks-Nrps | 1 | CYP107CA2 |
| sgb | T3pks | 1 | CYP107F4 |
|  | Arylpolyene-Ladderane | 1 | CYP1373A2 |
|  | Bacteriocin-T1pks-Nrps | 1 | CYP107BX10 |
|  | Melanin | 1 | CYP124G14 |
|  | T1pks-Nrps | 1 | CYP105D30 |
| scb | Terpene | 1 | CYP154A4 |
|  | Lantipeptide-Nrps | 4 | CYP246A1; CYP1048A1; CYP156D1; CYP154L1 |
|  | T1pks-Nrps | 1 | CYP107AM1 |
|  | Bacteriocin-Bottromycin | 1 | CYP283A1 |
|  | Butyrolactone-T1pks-Otherks | 1 | CYP107AL1 |
|  | Terpene | 1 | CYP157C5 |
|  | Indole-T1pks | 1 | CYP156B2 |
|  | T1pks | 1 | CYP107AK1 |
| ssx | Melanin | 1 | CYP124G3 |
|  | Nrps | 1 | CYP105N1 |
|  | T2pks-T1pks-Otherks | 2 | CYP181A1; CYP107Y1 |
|  | T1pks | 2 | CYP105AZ1; CYP105AZ2 |
|  | T1pks-Nrps | 1 | CYP107BX4 |
|  | T2pks-Butyrolactone-Nrps | 1 | CYP105A4 |
| svl | Terpene | 2 | CYP107BW1; CYP1013A2 |
|  | Nrps | 2 | CYP162A3; CYP107CK1 |
|  | T1pks | 1 | CYP124B3 |
|  | T1pks | 2 | CYP107BW1; CYP105AX1 |
|  | Otherks | 2 | CYP183F2; CYP105AV1 |
|  | Bacteriocin-Lantipeptide-T1pks-Otherks-Nrps | 4 | CYP105AQ2; CYP155A5; CYP107E9; CYP105AN3 |
|  | T1pks | 1 | CYP107AD1 |
|  | Lantipeptide-T1pks-Nrps | 1 | CYP105AY1 |
|  | T1pks | 1 | CYP105U1 |
|  | Terpene | 2 | CYP156C9; CYP125A20 |
|  | Indole | 2 | CYP107U9; CYP156B6 |
|  | Ladderane-Arylpolyene-Nrps | 3 | CYP163B4; CYP107CF1; CYP107CE1 |
|  | Terpene | 1 | CYP147F5 |
| sct | T1pks | 2 | CYP107AS; CYP107CR1 |
|  | Lantipeptide | 1 | CYP107AE6 |
|  | T1pks-Nrps | 1 | CYP184A4 |
|  | Transatpks-T1pks-Nrps | 2 | CYP107CS1; CYP107W2 |
|  | T3pks-Terpene | 1 | CYP158A13 |
|  | Lantipeptide | 1 | CYP105AA10 |
|  | T1pks-Butyrolactone-Nrps | 2 | CYP107CT1; CYP105B25 |
| scy | T1pks | 3 | CYP107AS; CYP1274A1; CYP107CR1 |
|  | Lantipeptide | 1 | CYP107AE6 |
|  | T1pks-Nrps | 1 | CYP184A4 |
|  | Transatpks-T1pks-Nrps | 2 | CYP107CS1; CYP107W2 |
|  | T3pks-Terpene | 1 | CYP158A13 |
|  | Lantipeptide | 1 | CYP105AA10 |
|  | T1pks-Butyrolactone-Nrps | 2 | CYP107CT1; CYP105B25 |
| sfa | Blactam-T1pks-Nrps | 2 | CYP247A3; CYP107BX5 |
|  | T1pks | 2 | CYP105AZ2; CYP105AZ1 |
|  | T1pks-Nrps | 3 | CYP1029A2; CYP1423A2; CYP285A2 |
|  | Terpene | 1 | CYP157K1 |
|  | Melanin | 1 | CYP124G4 |
|  | Nrps | 2 | CYP1035A4; CYP156B9 |
| sbh | Bacteriocin-T1pks-Nrps | 2 | CYP183C1; CYP183D1 |
|  | Terpene | 1 | CYP183E1 |
|  | Bacteriocin-Lantipeptide-T1pks | 1 | CYP1039A1 |
|  | Transatpks-T1pks-Nrps | 3 | CYP105H6; CYP107BK1; CYP171A2 |
|  | Nrps | 2 | CYP107BM3; CYP157B13 |
|  | T1pks-Nrps | 1 | CYP113G1 |
|  | Other | 1 | CYP154P1 |
|  | T1pks-Nrps | 1 | CYP1037A1 |
|  | Otherks | 1 | CYP268A4 |
|  | T1pks | 1 | CYP124B2 |
|  | Otherks-Nrps | 1 | CYP163C1 |
|  | Transatpks-Terpene-Nrps | 2 | CYP161C1; CYP183A2 |
| shy | T1pks | 1 | CYP105B22 |
|  | Terpene | 1 | CYP107X1 |
|  | Nrps | 1 | CYP163B6 |
|  | Nrps | 1 | CYP285B1 |
|  | T1pks | 2 | CYP105AZ2; CYP105AZ1 |
|  | T3pks | 1 | CYP158A14 |
|  | Terpene | 1 | CYP170A10 |
|  | Terpene | 1 | CYP180A6 |
|  | Bacteriocin-Nrps | 1 | CYP113K3 |
| sho | T1pks | 1 | CYP105B22 |
|  | Terpene | 1 | CYP107X1 |
|  | Nrps | 1 | CYP163B6 |
|  | Nrps | 1 | CYP285B1 |
|  | T1pks | 2 | CYP105AZ2; CYP105AZ1 |
|  | T3pks | 1 | CYP158A14 |
|  | Terpene | 1 | CYP170A10 |
|  | Terpene | 1 | CYP180A6 |
|  | Bacteriocin-Nrps | 1 | CYP113K3 |
| sve | Lantipeptide-Terpene | 1 | CYP157C14 |
|  | Indole | 1 | CYP245A3 |
|  | Other | 1 | CYP121A2 |
|  | T3pks | 1 | CYP158A5 |
|  | Other | 2 | CYP105AC2; CYP180A5 |
|  | Ladderane-Nrps | 4 | CYP1056A1; CYP107CL1; CYP162A4; CYP163B5 |
| sdv | T1pks-Nrps | 1 | CYP105BA1 |
|  | Other | 1 | CYP1005B2 |
|  | Bacteriocin-Lantipeptide | 1 | CYP179B1 |
|  | Terpene | 1 | CYP183K1 |
|  | Lantipeptide-T1pks-Nrps | 1 | CYP179A3 |
|  | Butyrolactone-Terpene | 1 | CYP180A6 |
|  | Bacteriocin-Oligosaccharide | 2 | CYP107CN1; CYP107CP1 |
|  | Terpene-Nrps | 1 | CYP170A9 |
|  | T1pks-Nrps | 3 | CYP113J1; CYP113J2; CYP162A5 |
|  | Otherks-Nrps | 2 | CYP125A22; CYP163C2 |
|  | T3pks | 1 | CYP158A7 |
|  | Terpene-T3pks-Cyanobactin-Nrps | 2 | CYP1041A2; CYP1058A1 |
| salb | T1pks-Nrps | 1 | CYP107BX2 |
|  | Otherks | 1 | CYP1420A1 |
|  | Nrps | 1 | CYP146A3 |
|  | Terpene | 1 | CYP170B5 |
|  | Lantipeptide | 1 | CYP154A1 |
|  | T3pks | 1 | CYP107F4 |
|  | Lantipeptide-T1pks-Nrps | 1 | CYP105H3 |
| sals | Nrps | 1 | CYP105BK3 |
|  | T1pks-Butyrolactone-Nrps | 1 | CYP107EJ1 |
|  | T1pks-Otherks | 2 | CYP113Y1; CYP105DB1 |
|  | T1pks | 1 | CYP107DU1 |
|  | Terpene | 1 | CYP170B5 |
|  | Arylpolyene-Nrps | 1 | CYP107T3 |
|  | Arylpolyene | 1 | CYP107KW1 |
|  | T2pks-Otherks | 1 | CYP1193A1 |
|  | T1pks-Nrps | 1 | CYP1194A1 |
| strp | Nrps | 2 | CYP156B9; CYP1035A4 |
|  | Melanin | 1 | CYP124G4 |
|  | Terpene | 1 | CYP157K1 |
|  | T1pks-Nrps | 3 | CYP285A2, CYP1423A2, CYP1029A2 |
|  | T1pks | 2 | CYP105AZ1; CYP105AZ2 |
|  | Bacteriocin-Otherks | 1 | CYP1057A1 |
|  | Blactam-T1pks-Nrps | 2 | CYP107BX5; CYP247A3 |
| sfi | T3pks | 1 | CYP107F4 |
|  | T1pks-Nrps | 1 | CYP107BX3 |
|  | Melanin | 1 | CYP124G2 |
| sci | Transatpks-T1pks-Nrps | 2 | CYP107CQ1; CYP105AJ2 |
|  | Bacteriocin | 2 | CYP1059A1; CYP105B21 |
|  | T3pks | 1 | CYP158A2 |
|  | Terpene | 1 | CYP170A10 |
|  | T1pks | 2 | CYP105BC1; CYP105AH2 |
|  | Terpene | 1 | CYP180A6 |
|  | Terpene | 1 | CYP183A4 |
|  | Bacteriocin-Nrps | 1 | CYP113K3 |
|  | Transatpks-T1pks-Nrps | 2 | CYP105AJ2; CYP107CQ1 |
| src | T1pks | 1 | CYP105AX1 |
|  | T1pks | 1 | CYP124B3 |
|  | T1pks-Nrps | 3 | CYP161D1; CYP105AT1; CYP107L12 |
|  | Terpene | 1 | CYP1013A2 |
|  | T1pks-Nrps | 1 | CYP105AU1 |
|  | Terpene | 1 | CYP147F5 |
|  | Nrps-Arylpolyene-Ladderane | 3 | CYP107CE1; CYP107CF1; CYP163B4 |
|  | Indole | 2 | CYP156B6; CYP107U9 |
|  | Terpene | 2 | CYP125A20; CYP156C8 |
|  | T1pks | 3 | CYP194B3; CYP194B4; CYP107BS2 |
|  | T1pks-Nrps | 3 | CYP105AW1; CYP122A4; CYP107G2 |
|  | Otherks | 1 | CYP183F1 |
|  | T1pks-Arylpolyene-Ladderane | 1 | CYP107B3 |
|  | T1pks-Nrps | 1 | CYP105AY1 |
|  | T1pks | 1 | CYP107AD1 |
|  | Bacteriocin-Nrps-Lantipeptide-T1pks-Otherks | 3 | CYP105AN3; CYP107E9; CYP155A5 |
|  | Terpene | 1 | CYP105AV1 |
|  | T1pks | 1 | CYP107CD1 |
| salu | Butyrolactone | 1 | CYP107L43 |
|  | Other | 1 | CYP1190A1 |
|  | Other | 1 | CYP1192A1 |
|  | T1pks-Nrps | 1 | CYP1191A1 |
|  | Nrps | 1 | CYP107EB1 |
|  | Transatpks-T1pks-Nrps | 2 | CYP163B9; CYP105AA13 |
|  | T1pks | 2 | CYP105H9; CYP161A7 |
|  | Transatpks-Nrps | 3 | CYP107B6; CYP1189A1; CYP1189A2 |
|  | T3pks-Otherks-Butyrolactone-Nrps | 2 | CYP107F9; CYP163C3 |
|  | T2pks-Oligosaccharide-Nucleoside-Nrps | 2 | CYP113D6; CYP157C28 |
|  | Butyrolactone | 1 | CYP107AE9 |
|  | Lantipeptide | 1 | CYP251G1 |
|  | Terpene-T1pks | 2 | CYP161A6; CYP105H1 |
| sall | Nrps | 3 | CYP107EL1; CYP163B8; CYP107EA2 |
|  | Butyrolactone | 1 | CYP107L43 |
|  | Other | 1 | CYP1190A1 |
|  | Other | 3 | CYP147F21; CYP1060A2; CYP1192A1 |
|  | T1pks | 1 | CYP1191A1 |
|  | Transatpks-T1pks-Nrps | 2 | CYP163B9; CYP105AA13 |
|  | T1pks | 1 | CYP105H9 |
|  | Transatpks-Nrps | 2 | CYP1189A1; CYP1189A2 |
|  | T3pks | 1 | CYP107F9 |
|  | Otherks-Butyrolactone-Nrps | 1 | CYP163C3 |
|  | T2pks-Oligosaccharide-Nucleoside-Nrps | 2 | CYP113D6; CYP157C28 |
|  | Butyrolactone | 1 | CYP107AE9 |
|  | Lantipeptide | 1 | CYP251G1 |
|  | T1pks | 2 | CYP161A6; CYP105H1 |
|  | Nrps | 3 | CYP107EA2; CYP163B8; CYP107EL1 |
| slv | T3pks-Terpene-Nrps | 1 | CYP105N1 |
|  | Terpene | 1 | CYP170A1 |
|  | T3pks | 1 | CYP158A2 |
| sgu | Other | 1 | CYP178B1 |
|  | Nrps | 1 | CYP163B10 |
|  | Terpene | 1 | CYP170A22 |
|  | Terpene | 1 | CYP180A9 |
|  | T2pks | 1 | CYP107AH4 |
|  | Terpene | 1 | CYP157K3 |
|  | Nrps | 1 | CYP113K5 |
| svt | Other | 1 | CYP121A2 |
|  | T2pks-Nrps | 3 | CYP1029A3; CYP1423A1; CYP285A5 |
|  | Lantipeptide | 1 | CYP107LD1 |
|  | T2pks-Lantipeptide-Terpene | 2 | CYP154C11; CYP157A19 |
| stre | Butyrolactone-T1pks-Nrps | 1 | CYP105BA3 |
|  | T1pks-Arylpolyene | 3 | CYP107EM1; CYP1198A1; CYP105BV1 |
|  | Terpene | 1 | CYP157C31 |
|  | T1pks | 2 | CYP105H1; CYP161A6 |
|  | Butyrolactone | 1 | CYP107AE10 |
|  | T3pks | 1 | CYP107F9 |
|  | T1pks | 3 | CYP105DE1; CYP105BS1; CYP1197A1 |
|  | Thiopeptide-T1pks | 1 | CYP184A8 |
|  | Nrps | 1 | CYP1196A1 |
|  | Transatpks-Nrps | 2 | CYP107CA2; CYP154D14 |
|  | Lantipeptide | 1 | CYP107L42 |
|  | Lantipeptide-Nrps | 2 | CYP163B; CYP1278B1 |
|  | Terpene-T1pks | 1 | CYP105H8 |
|  | T1pks-Butyrolactone-Nrps | 1 | CYP105BA3 |
| scw | T1pks | 2 | CYP107L35; CYP105BT1 |
|  | Terpene | 1 | CYP170A17 |
|  | T1pks | 1 | CYP107W3 |
|  | Siderophore | 1 | CYP105AC8 |
|  | Terpene | 2 | CYP161C4; CYP183A4 |
|  | Terpene | 1 | CYP157K4 |
|  | T3pks-Fused-Nrps | 1 | CYP105AC17 |
| sld | T2pks | 1 | CYP105BW1 |
|  | Transatpks | 2 | CYP147K2; CYP1038A7 |
|  | Terpene | 1 | CYP147F28P |
|  | Butyrolactone | 1 | CYP107AE8 |
|  | Other | 1 | CYP1469A2 |
|  | Terpene | 1 | CYP157C29 |
|  | T3pks-Nrps | 2 | CYP107F8; CYP105D25 |
|  | Terpene | 1 | CYP157C42 |
|  | T2pks-Oligosaccharide | 1 | CYP113D5 |
|  | Lassopeptide-Nrps | 2 | CYP1278B2; CYP163B11 |
|  | Other | 1 | CYP121A3 |
|  | T1pks | 2 | CYP161A5; CYP105H3 |
|  | T1pks | 1 | CYP186D1 |
| sxi | Terpene | 1 | CYP157C32 |
|  | T1pks-Nrps | 2 | CYP1029A4; CYP285A6 |
|  | Lantipeptide-Linaridin | 1 | CYP1223B1 |
|  | Linaridin-T1pks-Lassopeptide-Nrps | 1 | CYP107LF1 |
|  | Terpene | 1 | CYP183W1 |
|  | T3pks-Terpene-Nrps | 1 | CYP107F11 |
| strm | Terpene-Nrps | 1 | CYP154Q2 |
|  | Lantipeptide-Terpene | 2 | CYP157C26; CYP134A3 |
|  | Other | 1 | CYP105DF1 |
|  | Thiopeptide | 2 | CYP2238A1; CYP1048A3 |
|  | Terpene-Otherks | 2 | CYP154D15; CYP157C27 |
|  | T1pks | 2 | CYP105L3; CYP1995C1 |
|  | Terpene | 1 | CYP251F1 |
| strc | T1pks-Transatpks-Terpene | 1 | CYP105AC15 |
|  | Nrps | 2 | CYP107LC1; CYP105BK2 |
|  | T2pks | 1 | CYP1064A5 |
|  | Fused | 1 | CYP1341E2 |
|  | T3pks | 2 | CYP157A20; CYP154C13 |
|  | T3pks-Terpene-Otherks | 1 | CYP123D1 |
|  | T1pks-Nrps | 1 | CYP105BA2 |
|  | T3pks-Nrps | 2 | CYP165E2; CYP165B8 |
|  | Terpene | 1 | CYP157C32 |
| samb | T2pks-Butyrolactone | 1 | CYP154K2 |
|  | Indole | 1 | CYP156B15 |
|  | Terpene | 1 | CYP170A1 |
|  | Oligosaccharide-T1pks-Nrps | 2 | CYP107EP1; CYP113B4 |
|  | Terpene | 1 | CYP157K5 |
|  | T1pks | 2 | CYP107EF1, CYP107EP1 |
|  | T2pks-Butyrolactone | 1 | CYP154K2 |
| spri | Oligosaccharide-Ectoine-T2pks-Nrps-T1pks-Otherks | 3 | CYP154A22; CYP107EH1; CYP113C2 |
|  | Melanin | 1 | CYP124G6 |
|  | T2pks-Oligosaccharide-Nrps-Otherks | 3 | CYP154B6; CYP113C2, CYP107EH1 |
|  | T1pks-Ectoine-Otherks | 1 | CYP154A22 |
| scz | Melanin | 1 | CYP124G2 |
|  | Bacteriocin-T1pks-Nrps | 1 | CYP107BX7 |
|  | Nrps | 1 | CYP154A18 |
|  | T3pks | 1 | CYP107F4 |
|  | Arylpolyene | 1 | CYP105D20 |
| scx | Indole | 1 | CYP145C3 |
|  | Terpene | 1 | CYP180A29 |
|  | Terpene | 1 | CYP170A19 |
|  | Terpene | 2 | CYP157C39; CYP183B5 |
| srw | T1pks | 1 | CYP166A3 |
|  | Bacteriocin-Nrps | 1 | CYP113K6 |
|  | Other | 1 | CYP162B2 |
|  | Other | 2 | CYP121A5; CYP154U7 |
|  | T2pks-T3pks-Otherks | 1 | CYP158A14 |
|  | Nrps | 2 | CYP107LB1; CYP105BK2 |
|  | Butyrolactone-Amglyccycl-T1pks-Nrps | 1 | CYP1037A2 |
|  | Terpene | 1 | CYP170A23 |
|  | Nrps | 1 | CYP2045A1 |
|  | Terpene | 1 | CYP180A28 |
| strf | Nrps-T2pks-Otherks-T1pks-Phenazine | 1 | CYP105DD1 |
|  | Terpene | 1 | CYP170A16 |
|  | T2pks-Otherks | 1 | CYP105DD1 |
| sle | T3pks-T1pks-Nrps | 3 | CYP107L35; CYP105BT1; CYP154B4 |
|  | Terpene | 1 | CYP157K4 |
|  | Lassopeptide | 1 | CYP102B20 |
|  | Nrps | 1 | CYP113K3 |
|  | Nrps-Transatpks-Terpene-Otherks | 2 | CYP107EG1; CYP105BR1 |
|  | Terpene-T1pks | 3 | CYP107Q3; CYP105D28; CYP166A2 |
|  | T1pks | 3 | CYP154Z1; CYP1416A1; CYP2266A2 |
|  | Terpene | 2 | CYP183A4; CYP161C4 |
|  | T1pks-Siderophore | 2 | CYP107L33; CYP105AC8 |
|  | T3pks | 1 | CYP107F10 |
|  | Terpene | 1 | CYP170A17 |
|  | T1pks | 1 | CYP1418A1 |
|  | T1pks | 1 | CYP1031A3 |
|  | Other | 1 | CYP107AM11 |
| srn | Terpene | 3 | CYP251A3; CYP157C21; CYP158A20 |
|  | Indole | 2 | CYP245A6; CYP244A3 |
|  | Nrps | 2 | CYP159A23; CYP157B32 |
|  | Thiopeptide | 2 | CYP183H3; CYP183G3 |
|  | Terpene | 1 | CYP251F2 |
|  | Lantipeptide-Nrps | 1 | CYP1207A10 |
|  | T3pks-Butyrolactone | 1 | CYP158A19 |
|  | Nrps | 2 | CYP107E12; CYP285D1 |
| spav | Indole | 2 | CYP156B15; CYP183J3 |
|  | Terpene | 1 | CYP170A1 |
|  | Terpene | 2 | CYP157K6; CYP105D33 |
| slc | T1pks | 1 | CYP147F26 |
|  | Terpene-T1pks-Nrps | 2 | CYP107FH2; CYP107B26 |
|  | T1pks-Nrps | 1 | CYP163G1 |
|  | Thiopeptide | 1 | CYP147F27 |
|  | Butyrolactone | 1 | CYP107AE12 |
|  | Melanin-Nrps | 1 | CYP107FV4 |
|  | Other | 1 | CYP1005B7 |
|  | Nucleoside-Lassopeptide-Nrps | 1 | CYP157C36 |
|  | T3pks-Nrps | 1 | CYP107F15 |
| strt | Phosphonate-Nrps | 1 | CYP1618A1 |
|  | Thiopeptide-Terpene | 1 | CYP180B6 |
|  | Butyrolactone | 1 | CYP105B73 |
|  | Terpene | 1 | CYP183X4 |
|  | T3pks | 1 | CYP158A25 |
|  | Other | 1 | CYP107P31 |
|  | Terpene | 1 | CYP170A18 |
|  | Bacteriocin-T1pks | 1 | CYP158A24 |
|  | Phosphoglycolipid | 1 | CYP107E32 |
|  | Bacteriocin-Terpene-Nrps | 2 | CYP1722A3; CYP1618A2 |
| sclf | Terpene-T1pks-Nrps | 3 | CYP105BG1; CYP163B7; CYP251E1 |
|  | T3pks | 1 | CYP107F7 |
|  | Indole-Terpene-Nrps | 2 | CYP107NSF1; CYP107BY2 |
|  | T1pks-Nrps | 1 | CYP136E1 |
|  | Blactam-Nrps | 1 | CYP105M1 |
|  | Melanin | 1 | CYP124G5 |
|  | T1pks-Butyrolactone-Otherks | 1 | CYP107AL2 |
|  | T1pks | 1 | CYP154A14 |
| sgs | Thiopeptide-Bacteriocin | 1 | CYP113V2 |
|  | Terpene | 1 | CYP170A10 |
|  | T1pks-Nrps | 1 | CYP247A3_ortholog |
|  | T1pks | 1 | CYP105BC2 |
|  | Terpene | 1 | CYP180A26 |
|  | Lantipeptide-T1pks-Nrps | 2 | CYP208A9; CYP107BK3 |
|  | Nrps | 1 | CYP107KX1 |
|  | Other | 2 | CYP121A4; CYP154U6 |
|  | Terpene | 1 | CYP183X2 |
|  | Phosphonate-T3pks-Nrps-Ladderane | 1 | CYP158A21 |
| stsi | Butyrolactone | 1 | CYP154K3 |
|  | Terpene | 1 | CYP170A15 |
|  | Nrps | 2 | CYP163B15; CYP113Z1 |
| sls | Ladderane | 1 | CYP105B41 |
|  | Nrps | 1 | CYP163A5 |
|  | Nrps | 1 | CYP1424A1 |
|  | T3pks | 1 | CYP158A15 |
|  | Terpene | 2 | CYP183Y1; CYP157C22 |
|  | Terpene | 1 | CYP170A20 |
|  | Nrps-Siderophore | 1 | CYP107L32 |
|  | T2pks | 1 | CYP105B41 |
| snr | T1pks | 5 | CYP105BV1; CYP1198A1; CYP107EM1; CYP1060A2; CYP147F29 |
|  | Terpene | 1 | CYP157C31 |
|  | T1pks | 2 | CYP105H1; CYP161A1 |
|  | Butyrolactone | 1 | CYP107AE10 |
|  | Other | 1 | CYP1248A3 |
|  | T1pks | 1 | CYP107EQ1 |
|  | T1pks | 3 | CYP107KZ1; CYP1420A1; CYP107A3 |
|  | T3pks | 1 | CYP107F9 |
|  | Thiopeptide-Bacteriocin | 1 | CYP113D4 |
|  | T1pks | 1 | CYP105AB15 |
|  | Transatpks-Nrps | 3 | CYP107CA2; CYP154D14; CYP105AC16 |
|  | Terpene | 1 | CYP105A7 |
|  | Lantipeptide-Nrps | 2 | CYP163B; CYP1278B1 |
|  | Terpene-T1pks | 1 | CYP105H8 |

**Table S9. Comparative analysis of secondary metabolite biosynthetic gene clusters and P450s that are part of these clusters in mycobacterial species.**

| Species code | Cluster name | Number of P450s | P450 name |
| --- | --- | --- | --- |
| MAF | T3pks-T1pks | 1 | CYP139A1 |
|  | Other | 3 | CYP124A1; CYP128A1; CYP121A1 |
| MTO | T3pks-T1pks | 1 | CYP139A1 |
|  | Nrps | 1 | CYP144A1 |
|  | Other | 3 | CYP124A1; CYP128A1; CYP121A1 |
| MTF | T3pks-T1pks | 1 | CYP139A1 |
|  | Other | 3 | CYP124A1; CYP128A1; CYP121A1 |
| MRA | T3pks-T1pks | 1 | CYP139A1 |
|  | T1pks | 1 | CYP139A1 |
|  | Other | 2 | CYP124A1; CYP128A1 |
| MTU | T3pks-T1pks | 1 | CYP139A1 |
|  | Nrps | 1 | CYP144A1 |
|  | Other | 2 | CYP124A1; CYP128A1 |
| MTUL | T3pks-T1pks | 1 | CYP139A1 |
| MTB | Other | 3 | CYP121A1; CYP128A1; CYP124A1 |
|  | Nrps | 1 | CYP144A1 |
|  | T3pks-T1pks | 1 | CYP139A1 |
| MTZ | Other | 3 | CYP121A1; CYP128A1; CYP124A1 |
|  | Nrps | 1 | CYP144A1 |
|  | T3pks-T1pks | 1 | CYP139A1 |
| MTK | Other | 3 | CYP121A1; CYP128A1; CYP124A1 |
|  | Nrps | 1 | CYP144A1 |
|  | T3pks-T1pks | 1 | CYP139A1 |
| MTG | Other | 2 | CYP124A1; CYP102B25 |
| MTC | T3pks-T1pks | 1 | CYP139A1 |
|  | Other | 3 | CYP124A1; CYP128A1; CYP121A1 |
| MTE | T3pks-T1pks | 1 | CYP139A1 |
|  | T1pks | 3 | CYP124A1; CYP128A1; CYP121A1 |
|  | Other | 3 | CYP124A1; CYP128A1; CYP121A1 |
| MTUB | T3pks-T1pks | 1 | CYP139A1 |
| MTJ | T3pks-T1pks | 1 | CYP139A1 |
|  | Nrps | 1 | CYP144A1 |
|  | Other | 3 | CYP124A1; CYP128A1; CYP121A1 |
| MTUC | T3pks | 1 | CYP139A1 |
|  | Other | 2 | CYP124A1; CYP128A1 |
| MTX | T3pks-T1pks | 1 | CYP139A1 |
|  | Nrps | 1 | CYP144A1 |
| MTUH | T3pks | 1 | CYP139A1 |
| MTN | T3pks-T1pks | 1 | CYP139A1 |
| MTD | T3pks-T1pks | 1 | CYP139A1 |
|  | Other | 2 | CYP124A1; CYP128A1 |
| MCE | T3pks-T1pks | 1 | CYP139A1 |
|  | Other | 3 | CYP124A1; CYP128A1; CYP121A1 |
| MCQ | T3pks-T1pks | 1 | CYP139A1 |
|  | Other | 2 | CYP124A1; CYP128A1 |
| MCX | T3pks-T1pks | 1 | CYP139A1 |
|  | Nrps | 1 | CYP144A1 |
|  | Other | 3 | CYP124A1; CYP128A1; CYP121A1 |
| MBO | T3pks-T1pks | 1 | CYP139A1 |
|  | Other | 3 | CYP124A1; CYP128A1; CYP121A1 |
| MBB | T3pks-T1pks | 1 | CYP139A1 |
|  | Other | 3 | CYP124A1; CYP128A1; CYP121A1 |
| MBK | T3pks-T1pks | 1 | CYP139A1 |
|  | Other | 3 | CYP124A1; CYP128A1; CYP121A1 |
| MBM | T3pks-T1pks | 1 | CYP139A1 |
|  | Other | 3 | CYP124A1; CYP128A1; CYP121A1 |
| MBT | T3pks-T1pks | 1 | CYP139A1 |
|  | Other | 3 | CYP124A1; CYP128A1; CYP121A1 |
| MAB | T1pks-Nrps | 1 | CYP1110B1 |
|  | T2pks | 1 | CYP153A |
|  | Nrps | 1 | CYP1128A |
|  | Nrps | 1 | CYP135B |
| MABB | Nrps | 1 | CYP1128A |
|  | Nrps | 1 | CYP135B |
| MABL | Nrps | 1 | CYP1128A |
|  | Nrps | 1 | CYP135B |
| MAZ | T1pks-Nrps | 1 | CYP1110B1 |
|  | T2pks | 1 | CYP153A |
|  | Nrps | 1 | CYP1128A |
|  | Nrps | 1 | CYP135B |
| MAY | T1pks-Nrps | 1 | CYP1110B1 |
|  | T2pks | 1 | CYP153A |
|  | Nrps | 1 | CYP1128A |
|  | Nrps | 1 | CYP135B |
| MYS | Nrps | 1 | CYP135B |
|  | Nrps | 1 | CYP1128A |
| MAV | Nrps | 1 | CYP187A |
|  | T1pks | 1 | CYP150A |
|  | T1pks | 1 | CYP105NSF1 |
|  | T3pks-T1pks | 1 | CYP139A |
| MPA | T3pks-T1pks | 1 | CYP139A |
|  | T1pks | 1 | CYP105NSF1 |
|  | T1pks-Nrps | 1 | CYP150A |
|  | Nrps | 1 | CYP1034A |
|  | T3pks | 1 | CYP187A |
| MAO | T3pks | 1 | CYP187A |
|  | Nrps | 1 | CYP1034A |
|  | T1pks-Nrps | 1 | CYP150A |
|  | T1pks | 1 | CYP105NSF1 |
|  | T3pks-T1pks | 1 | CYP139A |
|  | T1pks | 1 | CYP144A |
| MIA | T3pks | 1 | CYP187A |
|  | T1pks-Nrps | 1 | CYP150A |
|  | T1pks | 2 | CYP279A; CYP105U |
|  | Other | 1 | CYP1122A1 |
| MIT | T3pks | 2 | CYP187A; CYP150A |
|  | T1pks-Nrps | 1 | CYP150A |
|  | T1pks | 2 | CYP279A; CYP105U |
|  | Other | 1 | CYP1128A |
| MIE | T3pks-T1pks | 1 | CYP187A |
|  | T1pks | 1 | CYP150A |
|  | T1pks | 2 | CYP279A; CYP105U |
| MMM | T3pks | 1 | CYP187A |
|  | T1pks-Nrps | 1 | CYP150A |
|  | T1pks | 2 | CYP279A; CYP105U |
| MID | T3pks | 1 | CYP1123A1 |
|  | Nrps | 1 | CYP126NSF1 |
| MYV | T1pks-Nrps | 1 | CYP135B |
| MLI | Nrps | 1 | CYP143A3 |
|  | Nrps | 2 | CYP140A5; CYP125A6 |
|  | Other | 1 | CYP147G |
|  | T3pks-T1pks | 1 | CYP139A3 |
| MMAE | Nrps | 1 | CYP185A4 |
|  | T3pks-T1pks | 1 | CYP139A3 |
|  | Nrps | 1 | CYP143A3 |
|  | Nrps | 2 | CYP140A5; CYP125A6 |
|  | Other | 1 | CYP147G1 |
|  | T3pks | 2 | CYP187A; CYP187A |
|  | Other | 1 | CYP164A3 |
| MMAL | T3pks | 1 | CYP187A |
|  | T1pks-Nrps | 1 | CYP150A |
|  | T1pks | 3 | CYP279A; CYP279A; CYP105U |
|  | Other | 2 | CYP105Q; CYP189A |
| MMV | Nrps | 1 | CYP1128A |
| MVA | T1pks-Nrps | 1 | CYP138C1 |
| MSB | T3pks | 1 | CYP185A1 |
| MCB | Arylpolyene | 3 | CYP187A; CYP150A; CYP189A |
|  | T1pks-Nrps | 1 | CYP138C |
|  | T1pks | 1 | CYP292A |
| MGI | Terpene | 1 | CYP188A |
|  | T1pks-Nrps | 1 | CYP138C2 |
|  | T1pks-Nrps | 3 | CYP189A; CYP150A; CYP187A |
| MSP | Terpene | 1 | CYP188A |
|  | T1pks-Nrps | 1 | CYP138C2 |
|  | Other | 3 | CYP189A; CYP150A; CYP187A |
| MSA | T3pks | 1 | CYP135B |
|  | Other | 2 | CYP187A; CYP150A |
|  | T1pks-Nrps | 1 | CYP136NSF2 |
| MRH | T1pks | 2 | CYP189A; CYP102NSF1 |
|  | T1pks-Nrps | 1 | CYP1121A1 |
|  | Nrps | 1 | CYP125A |
|  | Nrps | 1 | CYP189A |
|  | Arylpolyene | 1 | CYP124A |
|  | Arylpolyene | 1 | CYP144A |
| MNE | Other | 1 | CYP161NSF1 |

**Table S10. Comparative analysis of P450s that are part of secondary metabolite biosynthetic gene clusters between the genera *Streptomyces* and *Mycobacterium*.** The P450 families commonly found in both genera are highlighted in bold font.

| ***Streptomyces*** | | ***Mycobacterium*** | |
| --- | --- | --- | --- |
| P450 family | No. of P450s | P450 family | No. of P450s |
| CYP107 | 127 | CYP139 | 32 |
| **CYP105** | **95** | **CYP124** | **24** |
| CYP157 | 27 | CYP128 | 22 |
| CYP154 | 23 | **CYP121** | **17** |
| CYP163 | 22 | CYP187 | 14 |
| CYP170 | 21 | CYP150 | 13 |
| CYP113 | 19 | CYP144 | 10 |
| CYP183 | 19 | **CYP105** | **9** |
| CYP158 | 17 | CYP1128 | 8 |
| **CYP124** | **12** | CYP135 | 8 |
| CYP180 | 11 | CYP189 | 6 |
| CYP156 | 10 | CYP279 | 6 |
| **CYP161** | **10** | CYP138 | 4 |
| **CYP147** | **9** | CYP1110 | 3 |
| CYP285 | 7 | **CYP125** | **3** |
| CYP251 | 6 | CYP153 | 3 |
| **CYP121** | **5** | **CYP102** | **2** |
| CYP162 | 5 | CYP1034 | 2 |
| CYP1029 | 4 | CYP140 | 2 |
| CYP1189 | 4 | CYP143 | 2 |
| **CYP125** | **3** | **CYP147** | **2** |
| CYP1278 | 3 | CYP185 | 2 |
| CYP1423 | 3 | CYP188 | 2 |
| CYP178 | 3 | CYP1121 | 1 |
| CYP184 | 3 | CYP1122 | 1 |
| CYP247 | 3 | CYP126 | 1 |
| CYP1005 | 2 | **CYP136** | **1** |
| CYP1013 | 2 | **CYP161** | **1** |
| CYP1035 | 2 | CYP164 | 1 |
| CYP1037 | 2 | CYP292 | 1 |
| CYP1048 | 2 | CYP1123 | 1 |
| CYP1060 | 2 |  |  |
| CYP1190 | 2 |  |  |
| CYP1191 | 2 |  |  |
| CYP1192 | 2 |  |  |
| CYP1198 | 2 |  |  |
| CYP1420 | 2 |  |  |
| CYP155 | 2 |  |  |
| CYP1618 | 2 |  |  |
| CYP165 | 2 |  |  |
| CYP166 | 2 |  |  |
| CYP171 | 2 |  |  |
| CYP179 | 2 |  |  |
| CYP181 | 2 |  |  |
| CYP194 | 2 |  |  |
| CYP208 | 2 |  |  |
| CYP245 | 2 |  |  |
| **CYP102** | **1** |  |  |
| CYP1031 | 1 |  |  |
| CYP1038 | 1 |  |  |
| CYP1039 | 1 |  |  |
| CYP1041 | 1 |  |  |
| CYP1056 | 1 |  |  |
| CYP1057 | 1 |  |  |
| CYP1058 | 1 |  |  |
| CYP1059 | 1 |  |  |
| CYP1064 | 1 |  |  |
| CYP1193 | 1 |  |  |
| CYP1194 | 1 |  |  |
| CYP1196 | 1 |  |  |
| CYP1197 | 1 |  |  |
| CYP1207 | 1 |  |  |
| CYP1223 | 1 |  |  |
| CYP122 | 1 |  |  |
| CYP123 | 1 |  |  |
| CYP1248 | 1 |  |  |
| CYP1274 | 1 |  |  |
| CYP1341 | 1 |  |  |
| CYP134 | 1 |  |  |
| **CYP136** | **1** |  |  |
| CYP1373 | 1 |  |  |
| CYP1416 | 1 |  |  |
| CYP1418 | 1 |  |  |
| CYP1424 | 1 |  |  |
| CYP145 | 1 |  |  |
| CYP1469 | 1 |  |  |
| CYP146 | 1 |  |  |
| CYP159 | 1 |  |  |
| CYP1722 | 1 |  |  |
| CYP186 | 1 |  |  |
| CYP1995 | 1 |  |  |
| CYP2045 | 1 |  |  |
| CYP2238 | 1 |  |  |
| CYP2266 | 1 |  |  |
| CYP244 | 1 |  |  |
| CYP246 | 1 |  |  |
| CYP268 | 1 |  |  |
| CYP283 | 1 |  |  |

**Table S11. Functional analysis of P450s.** Functional analysis of P450s is presented at family level. Functional analysis of specific family members of the *Streptomyces* species is presented in Table S12.

| **P450 family** | **General function** | **References** |
| --- | --- | --- |
| **Functional analysis of Streptomyces P450s** | | |
| CYP102 | Fatty acid hydroxylation | 66 |
| CYP105 | Biotransformation or degradation of xenobiotics, and biosynthesis of secondary metabolites | 67, 68 |
| CYP107 | Biosynthesis of secondary metabolites | 69 |
| CYP113 | Biosynthesis of secondary metabolites | 70 |
| CYP122 | Metabolism of terpenoids and polyketides | 71 |
| CYP158 | Biosynthesis of secondary metabolites (flaviolin) | 72, 73 |
| CYP154 | Production of secondary metabolites and hydroxylation of steroids | 74, 75, 76 |
| CYP170 | Biosynthesis of secondary metabolites | 77, 78, 79 |
| CYP1048 & CYP264 | Production of phytotoxins | 80, 81 |
| CYP161 | Biosynthesis of secondary metabolites | 82 |
| CYP151 & CYP248 | Biosynthesis of secondary metabolites | 83 |
| CYP163 | Biosynthesis of secondary metabolites | 84 |
| CYP129 & CYP131 | Biosynthesis of secondary metabolites | 85-87 |
| CYP162 | Biosynthesis of secondary metabolites | 88 |
| CYP245 & CYP244 | Biosynthesis of secondary metabolites | 89, 90 |
| CYP183 | Biosynthesis of secondary metabolites | 91 |
| **Functional analysis of P450s from other microorganisms** | | |
| CYP121 | Synthesis of mycocyclosin, a natural product | 92 |
| CYP125 | Cholesterol oxidation | 93, 94 |
| CYP124 | Cholesterol and lipids hydroxylation | 95 |
| CYP152 | Alkene production | 96 |
| CYP108 | Terpineol hydroxylation | 97 |
| CYP116 | Degradation of toxic compounds (herbicides and alkyl aryl ethers) | 98, 99 |
| CYP140 | Mycolactone toxin synthesis | 100 |
| CYP165& CYP146 | Secondary metabolite production (vancomycin biosynthesis) | 101, 102 |
| CYP199 | Oxidation of benzoic acid derivatives | 103, 104 |
| CYP268 | Hydroxylation of long chain branched acetate and ketone | 105 |

**Table S12: Functional analysis of P450s.** Functional analysis of P450s was presented at P450 subfamily level with respect to *Streptomyces* species.

| **Species name** | **CYP P450** | **Function** | **References** |
| --- | --- | --- | --- |
| *Streptomyces coelicolor* A3(2) | CYP170A1 | - Catalyzes the oxidation of epi-isozizaene to an epimeric mix of 5-albaflavenol | 79 |
|  | CYP102B1 | - Fatty acid hydroxylase | 66 |
|  | CYP105N1 | - Oxidase in coelibactin siderophore biosynthesis - Monooxygenase involved in coelibactin synthesis | 106, 107 |
|  | CYP107U1 | - Putative steroid oxidase with role in sporulation and antibiotic synthesis | 108 |
|  | CYP154A1 | - Di-pentaenone cyclization - Involved in polyketide metabolism | 75, 76 |
|  | CPY158A1  CYP158A2 | - C–C coupling in flaviolin polymerization | 72, 73 |
|  | CYP170A1 | - Two-step allylic oxidation of epi-isozizaene to albaflavenone in albaflavenone biosynthesis | 79 |
|  | CYP154C1 | - 12- and 14-carbon macrolactone monooxygenase   e.g., narbomycin hydroxylase | 74 |
| *Streptomyces avermitilis* | CYP102D1  C1-CYP105D6  C26-CYP105P1 | - Fatty acid hydroxylase | 109; 110 |
|  | CYP105D6 | - C1-hydroxylation of filipin | 109 |
|  | CYP105D7 | - Filipin hydroxylase - 1-deoxypentalenic acid hydroxylase | 78 |
|  | CYP105P1 | - Filipin hydroxylation | 109 |
|  | CYP170A2 | - 2-step allylic oxidation of epi-isozizaene to albaflavenone | 111 |
|  | CYP171A1 | - C6 and C8a avermectin algycone hydroxylation | 112 |
|  | CYP183A | - Pentalene hydroxylase | 91 |
| *Streptomyces scabiei* | CYP1048A1 | - Direct nitration of L-tryptophan with NO, O2, redox partners, and NADPH - Plays a novel catalytic role in the biosynthesis of a cyclic dipeptide phytotoxin - Involved in the production of the plant toxin thaxtomin, responsible for potato common scab | 80, 113-115 |
|  | CYP246A1 | - Thaxtomin phenylalanyl di-hydroxylase in thaxtomin A biosynthesis | 81 |
| *Streptomyces griseolus* | CYP105A1 | - Catalyze highly selective oxidations of diterpenoids - Vitamin D3 hydroxylase involved in the conversion of vitamin D3 to its active form 1α,25-hydroxy vitamin D3 | 116-118 |
|  | CYP154C3 | - Catalyzes monooxygenation reactions of a range of steroids | 119 |
| *Streptomyces venezuelae* | CYP107L1 | - Involved in ring decoration of macrolide antibiotics - Catalyzes regioselective C-12 hydroxylation of narbomycin (the final step of pikromycin biosynthesis) - 12- and 14- carbon macrolactone, e.g narbomycin and YC-17 hydroxylation | 69 |
| *Streptomyces natalensis* | CYP161A2 (PimD) | - 4,5-Desepoxypimaricin epoxidase in pimaricin biosynthesis | 82 |
| *Streptomyces thioluteus* | CYP151A (AurH) | - Oxidation and ring formation to convert deoxyaureothin to aureothin | 83 |
|  | CYP248A1 | - Aureothin synthase | 83 |
| *Streptomyces himastatinicus*  *ATCC 53653* | CYP107B (HmtN) | - ʸ-Hydroxylation of an unusual pipera-zic acid (Pip) motif in himastatin biosynthesis | 70 |
| *Streptomyces himastatinicus*  *ATCC 53653* | HmtT | - Regio- and stereospecific C2/C3 epoxidation of L-tryptophan indole ring and subsequent cyclization forming hexahydropyrroloindole in himastatin biosynthesis | 70 |
| *Streptomyces sp. Acta 2897* | CYP163B3 (P450 Sky) | - 3 successive β-hydroxylations of separate PCP-bound L-amino acid precursors in skyllamycin biosynthesis | 84, 120 |
| *Streptomyces nodosus* | CYP161A3 (AmphL)  CYP105H4 (AmphN) | - Polyketide oxidative tailoring reactions | 121, 122 |
| *Streptomyces thermotolerans* | CYP107C1 | - C12-C13 epoxidation of carbomycin B to make carbomycin C | 123 |
| *Streptomyces bikiniensis* | ChmH1 | - C20 methyl macrolide hydroxylation | 124 |
| *Streptomyces clavuligerus* | CYP105M1 (orf10) | - Possible clavaminic acid derivative epoxidase | 125 |
| *Streptomyces sp* strain C5 | CYP129A2 (dox A)  CYP131A2 (dnrQ) | - C10,C13, C14 anthracycline glycine DNR precursor hydroxylations and likely aglycone core oxidation | 85-87 |
| *Streptomyces graminofaciens* | GfsF | - C8-9 macrolide epoxidation then C10 hydroxylation | 126, 127 |
| *Streptomyces tsukubaensis* | CYP122A4 (FkbD) | - 4-Electron C-9 FK506 precursor oxidation | 71 |
| *Streptomyces pulveraceus* | FosK | - C18 fostriecin hydroxylation | 128, 129 |
| *Streptomyces himastatinicus* | CYP107B (HmtN) and HmtT and HmtS | - Piperazic acid (Pip) motif ʸ-hydroxylation and C2/C3L-tryptophan epoxidation cyclization to hexahydropyrroloindole and biaryl aromatic coupling of depsipeptide monomers | 130-132 |
| *Streptomyces tendae* | CYP162A1 | - Histidine β-hydroxyation to form nikkomycins X and I | 88, 133 |
| *Streptomyces spheroids* | CYP163A1 (NovI) | - PCP-loaded tyrosine β-hydroxylation | 134, 135 |
| *Streptomyces peucetius* | CYP105F2 | - Oleandomycin tailoring hydroxylation | 136, 137 |
|  | CYP107A1 | - Catalyzes the H2O2-mediated dealkylation of 7-ethoxycoumarin | 138 |
| *Streptomyces hygroscopicus* | CYP107G1 (rapN)  CYP122A2 (rapJ)  CYP122A3 | - C9, C26, C27 and C32 rapamycin macrolactone hydroxylation | 89, 90 |
| *Streptomyces sp tp-a0274* | CYP245A1 (StaP) and CYP244 A1 (StaN) | - Aryl-aryl coupling of chromopyrrolic acid and C-N linkage of staurosporine aglycone | 89, 90 |
| *Streptomyces griseochromogenes* | TauI/TmcR | - C5 tautomycetin oxygenation | 139, 140 |
| *Streptomyces fradiae* | CYP105L1 (TylH1,orf7), CYP113B1 (TylI), CYP154B1 | - Likely C23 methyl lactone ring oxidase (CYP105L1) and C20 methyl O-mycaminosyl-tylactone hydroxylation (CYP113B1) | 141, 142 |
| *Streptomyces sp. 307-9* | TamI | - C10 oxidation of tirandamycin C to E, then C11-12 epoxidation C18 hydroxylation | 143, 144 |
| *Streptomyces albus* | CYP170B1 | - Produces albaflavenone from *epi*-isozizaene | 77 |

**REFERENCES**

1. Bilyk, B., Weber, S., Myronovskyi, M., Bilyk, O., Petzke, L. & Luzhetskyy, A. *In vivo* random mutagenesis of streptomycetes using mariner-based transposon *Himar1*. *Appl. Microbiol. Biotechnol.* **97**, 351-359 (2013).
2. Witt, D. & Stackebrandt, E., 1990. Unification of the genera *Streptoverticillum* and *Streptomyces*, and amendation of *Streptomyces Waksman* and *Henrici* 1943, 339^AL^. *Syst. Appl. Microbiol.* **13(4),** 361-371 (1990).
3. Bentley, S. D. *et al*. Complete genome sequence of the model actinomycete *Streptomyces coelicolor* A3(2). *Nature* **417(6885),** 141 (2002).
4. Distler, J., Mansouri, K., Mayer, G., Stockmann, M. & Piepersberg, W. Streptomycin biosynthesis and its regulation in Streptomycetes. *Gene* **115,** 105-111 (1992).
5. Ohnishi, Y. *et al.* Genome sequence of the streptomycin-producing microorganism *Streptomyces griseus* IFO 13350. *J. bacterial.* **190,** 4050-4060 (2008).
6. Wendt-Pienkowski, E. *et al.* Cloning, sequencing, analysis, and heterologous expression of the fredericamycin biosynthetic gene cluster from *Streptomyces griseus*. *J. Am. Chem. Soc.* **127(47),** 16442-16452 (2005).
7. Rebets, Y. *et al.* Production of landomycins in *Streptomyces globisporus* 1912 and *S. cyanogenus* S136 is regulated by genes encoding putative transcriptional activators. *FEMS Microbiol. Lett.* **222(1),** 149-153 (2003).
8. Beattie, G. A. Plant-associated bacteria: survey, molecular phylogeny, genomics and recent advances. In *Plant-associated bacteria*. 1-56. (Springer, Dordrecht, 2007).
9. Takasuka, T. E., Book, A. J., Lewin, G. R., Currie, C. R. & Fox, B. G. Aerobic deconstruction of cellulosic biomass by an insect-associated Streptomyces. *Sci. Rep.* **3,** 1030 (2013).
10. Gunnelius, L., Kurkela, J., Hakkila, K., Koskinen, S., Parikainen, M. & Tyystjärvi, T. The ω subunit of RNA polymerase is essential for thermal acclimation of the cyanobacterium *Synechocystis* sp. PCC 6803. *PLoS ONE* 9(11), e112599 (2014).
11. Núñez, L. E., Méndez, C., Braña, A. F., Blanco, G. & Salas, J. A. The biosynthetic gene cluster for the β-lactam carbapenem thienamycin in *Streptomyces cattleya*. *Chemistry & Biology* **10,** 301-311 (2003).
12. Rong, X. *et al.* Classification of Streptomyces phylogroup pratensis (Doroghazi and Buckley, 2010) based on genetic and phenotypic evidence, and proposal of *Streptomyces pratensis* sp. nov. *Syst. Appl. Microbiol.* **36,** 401-407 (2013).
13. Zhou, G. *et al*. Complete genome sequence of the Streptomyces sp. strain CdTB01, a bacterium tolerant to cadmium. *J. Biotechnol.* **229,** 42-43 (2016).
14. Matsumoto, S., Kouchi, M., Fukui, H. & Ueda, Y. Phylogenetic analyses of the subgenus Eurosa using the ITS nrDNA sequence. In *XXV International Horticultural Congress, Part 11: Application of Biotechnology and Molecular Biology and Breeding-Gene* **521**, 193-202 (1998).
15. Baskaran, S. *et al*. Architecture and dynamics of the autophagic phosphatidylinositol 3-kinase complex. *Elife* **3,** (2014).
16. Pullan, S. T., Chandra, G., Bibb, M. J. & Merrick, M. Genome-wide analysis of the role of GlnR in *Streptomyces venezuelae* provides new insights into global nitrogen regulation in actinomycetes. *BMC Genomics* **12,** 175 (2011).
17. Jankowitsch, F. *et al.* Genome sequence of the bacterium Streptomyces davawensis JCM 4913 and heterologous production of the unique antibiotic roseoflavin. *J. Bacteriol.* **194(24),** 6818-6827 (2012).
18. Zaburannyi, N., Rabyk, M., Ostash, B., Fedorenko, V. & Luzhetskyy, A. Insights into naturally minimised *Streptomyces albus* J1074 genome. *BMC Genomics* **15(1),** 97 (2014).
19. Lu, Q., Yu, Y., Ma, Q., Chen, B. & Zhang, H. 2D Transition‐Metal‐Dichalcogenide‐Nanosheet‐Based composites for photocatalytic and electrocatalytic hydrogen evolution reactions. *Advanced Materials* **28(10),** 1917-1933 (2016).
20. Shin, B., Gunawan, O., Zhu, Y., Bojarczuk, N. A., Chey, S. J. & Guha, S. Thin film solar cell with 8.4% power conversion efficiency using an earth‐abundant Cu_2_ZnSnS_4_ absorber. *Prog. Photovolt.: Research and Applications* **21,** 72-76 (2013).
21. Myronovskyi, M., Tokovenko, B., Manderscheid, N., Petzke, L. & Luzhetskyy, A. Complete genome sequence of *Streptomyces fulvissimus*. *J. Biotechnol.* **168,** 117-118 (2013).
22. Rückert, C. *et al*. Complete genome sequence of the kirromycin producer *Streptomyces collinus* Tü 365 consisting of a linear chromosome and two linear plasmids. *J. Biotechnol.* **168,** 739-740 (2013).
23. Baranasic, D. *et al*. Draft genome sequence of *Streptomyces rapamycinicus* strain NRRL 5491, the producer of the immunosuppressant rapamycin. *Genome announc.* **1***,* e00581-13 (2013).
24. Gu, Y. *et al*. Genome sequence of the ε-poly-L-lysine-producing strain *Streptomyces albulus* NK660, isolated from soil in Gutian, Fujian Province, China. *Genome announc.* **2,** e00532-14 (2014).
25. Wang, L., Gao, C., Tang, N., Hu, S. & Wu, Q. Identification of genetic variations associated with epsilon-poly-lysine biosynthesis in *Streptomyces albulus* ZPM by genome sequencing. *Sci. Rep.* **5,** 9201 (2015).
26. Rückert, C. *et al*. Complete genome sequence of *Streptomyces lividans* TK24. *J. Biotechnol.* **199,** 21-22 (2015).
27. Van Broekhoven, A., Shapiro, F. & Anné, J. eds. *Novel frontiers in the production of compounds for biomedical use,* 1. (Springer Science & Business Media, 2001).
28. Thompson, T. B., Katayama, K., Watanabe, K., Hutchinson, C. R. & Rayment, I. Structural and functional analysis of tetracenomycin F2 cyclase from *Streptomyces glaucescens* a type II polyketide cyclase. *J. Biol. Chem.* **279(36),** 37956-37963 (2004).
29. Deng, J., Ren, P., Deng, D. & Bao, X. Enhanced electron penetration through an ultrathin graphene layer for highly efficient catalysis of the hydrogen evolution reaction. *Angew. Chem. Int. Ed. Engl.* **54,** 2100-2104 (2015).
30. Zhu, H. H., Guo, J., Yao, Q., Yang, S. Z., Deng, M. R., Hanh, V. T. & Ryan, M. J. *Streptomyces vietnamensis* sp. nov., a streptomycete with violet–blue diffusible pigment isolated from soil in Vietnam. *Int. J. Syst. Evol. Microbiol.* **57(8),** 1770-1774 (2007).
31. Song, Y., Liu, G., Li, J., Huang, H., Zhang, X., Zhang, H. & Ju, J. Cytotoxic and antibacterial angucycline- and prodigiosin-analogues from the deep-sea derived *Streptomyces* sp. SCSIO 11594. *Mar. Drugs* **13,** 1304-1316 (2015).
32. Wang, H. *et al*. Complete genome sequence of *Streptomyces cyaneogriseus* ssp. noncyanogenus, the thermotolerant producer of commercial antibiotics nemadectin. *J. Biotechnol.* **204,** 1-2 (2015).
33. Wu, H., Li, J., Dong, D., Liu, T., Zhang, T., Zhang, D. & Liu, W. Heterologous coexpression of *Vitreoscilla* hemoglobin and *Bacillus megaterium* glucanase in *Streptomyces lydicus* A02 enhanced its production of antifungal metabolites. *Enzyme Microb. Technol.* **81,** 80-87 (2015).
34. Xu, J., Wang, Y., Xie, S. J., Xu, J., Xiao, J. & Ruan, J. S. *Streptomyces xiamenensis* sp. nov., isolated from mangrove sediment. *Int. J. Syst. Evol. Microbiol.* **59,** 472-476 (2009).
35. Barger, S. R., Hoefler, B. C., Cubillos-Ruiz, A., Russell, W. K., Russell, D. H. & Straight, P. D. Imaging secondary metabolism of *Streptomyces* sp. Mg1 during cellular lysis and colony degradation of competing *Bacillus subtilis*. *Antonie Van Leeuwenhoek* **102**, 435-445 (2012).
36. Hoefler, B. C., Konganti, K. & Straight, P. D. *De novo* assembly of the *Streptomyces* sp. strain Mg1 genome using PacBio single-molecule sequencing. *Genome announc.* **1(4),** e00535-13 (2013).
37. Leipoldt, F., Zeyhle, P., Kulik, A., Kalinowski, J., Heide, L. & Kaysser, L. Diversity of ABBA prenyltransferases in marine *Streptomyces* sp. CNQ-509: promiscuous Enzymes for the biosynthesis of mixed terpenoid compounds. *PLoS ONE* **10,** e0143237 (2015).
38. Karray, F. *et al*. Organization of the biosynthetic gene cluster for the macrolide antibiotic spiramycin in *Streptomyces ambofaciens*. *Microbiology* **153,** 4111-4122 (2007).
39. Juguet, M. *et al*. An iterative nonribosomal peptide synthetase assembles the pyrrole-amide antibiotic congocidine in *Streptomyces ambofaciens*. *Chem. Biol.* **16,** 421-431 (2009).
40. Aigle, B. *et al*. Genome mining of *Streptomyces ambofaciens*. *J. Ind. Microbiol. Biotechnol.* **41,** 251-263 (2014).
41. Thibessard, A. *et al*. Complete genome sequence of *Streptomyces ambofaciens* ATCC 23877, the spiramycin producer. *J. Biotechnol.* **214**, 117-118 (2015).
42. Qadri, H., Ueno, Y., Mostafa, A. & Halim, M. *In vitro* activity of quinupristin/dalfopristin, RP59500, against gram-positive clinical isolates. *Chemotherapy* **43,** 94-99 (1997).
43. Mast, Y., Weber, T., Gölz, M., Ort‐Winklbauer, R., Gondran, A., Wohlleben, W. & Schinko, E. Characterization of the ‘pristinamycin supercluster’ of *Streptomyces pristinaespiralis.* *Microb. Biotechnol.* **4,** 192-206 (2011).
44. Tian, J. *et al.* The complete genome sequence of a high pristinamycin-producing strain *Streptomyces pristinaespiralis* HCCB10218. *J. Biotechnol.* **214,** 45-46 (2015).
45. Nanthini, J. *et al*. Complete genome sequence of *Streptomyces* sp. strain CFMR 7, a natural rubber degrading actinomycete isolated from Penang, Malaysia. *J. Biotechnol.* **214,** 47-48 (2015).
46. Nanthini, J. & Sudesh, K. Biodegradation of natural rubber and natural rubber products by *Streptomyces* sp. Strain CFMR 7. *J. Polym. Env.* **25(3),** 606-616 (2017).
47. Schrempf, H., & S. Walter. The cellulolytic system of *Streptomyces reticuli*. *Int. J. Biol. Macromol.* **17(6),** 353-355 (1995).
48. Wibberg, D., Al-Dilaimi, A., Busche, T., Wedderhoff, I., Schrempf, H., Kalinowski, J. & de Orué Lucana, D. O. Complete genome sequence of *Streptomyces reticuli*, an efficient degrader of crystalline cellulose. *J. Biotechnol.* **222,** 13-14 (2016).
49. Chen, W. & Qin, Z. Development of a gene cloning system in a fast-growing and moderately thermophilic Streptomyces species and heterologous expression of Streptomyces antibiotic biosynthetic gene clusters. *BMC Microbiol.* **11(1),** 243 (2011).
50. Chen, W. H., Qin, Z. J., Wang, J., & Zhao, G. P. The MASTER (methylation-assisted tailorable ends rational) ligation method for seamless DNA assembly. *Nucleic Acids Res.* **41,** e93-e93 (2013).
51. Gomez-Escribano, J. P., Castro, J. F., Razmilic, V., Chandra, G., Andrews, B., Asenjo, J. A. & Bibb, M. J. The *Streptomyces leeuwenhoekii* genome: *de novo* sequencing and assembly in single contigs of the chromosome, circular plasmid pSLE1 and linear plasmid pSLE2. *BMC genomics* **16,** 485 (2015).
52. Kannan, R. R., Iniyan, A. M. & Vincent, S. G. P. Production of a compound against methicillin resistant *Staphylococcus aureus* (MRSA) from *Streptomyces rubrolavendulae* ICN3 & its evaluation in zebrafish embryos. *Indian J. Med. Res.* **139,** 913 (2014).
53. Shetty, P. R., Buddana, S. K., Tatipamula, V. B., Naga, Y. V. V. & Ahmad, J. Production of polypeptide antibiotic from *Streptomyces parvulus* and its antibacterial activity. *Braz. J. Microbiol.* **45,** 303-312 (2014).
54. Nishizawa, T., Miura, T., Harada, C., Guo, Y., Narisawa, K., Ohta, H., Takahashi, H. & Shirai, M. Complete genome sequence of *Streptomyces parvulus* 2297, integrating site-specifically with actinophage R4. *Genome Announc.* **4,** e00875-16 (2016).
55. Yuan, W. M. & Crawford, D. L. Characterization of *Streptomyces lydicus* WYEC108 as a potential biocontrol agent against fungal root and seed rots. *Appli. Environ. Microbiol.* **61,** 3119-3128 (1995).
56. Gomez, C., Olano, C., Mendez, C. & Salas, J. A. Three pathway-specific regulators control streptolydigin biosynthesis in *Streptomyces lydicus*. *Microbiology* **158,** 2504-2514 (2012).
57. Atta, H. M., El-Sayed, A. S., El-Desoukey, M. A., Hassan, M. & El-Gazar, M. Biochemical studies on the Natamycin antibiotic produced by *Streptomyces lydicus*: Fermentation, extraction and biological activities. *J. Saudi Chem. Soc.* **19**, 360-371 (2015).
58. Jia, N., Ding, M. Z., Luo, H., Gao, F. & Yuan, Y. J*.* Complete genome sequencing and antibiotics biosynthesis pathways analysis of *Streptomyces lydicus* 103. *Sci. Rep.* **7,** 44786 (2017)
59. De la Fuente, A., Lorenzana, L. M., Martín, J. F. & Liras, P. Mutants of *Streptomyces clavuligerus* with disruptions in different genes for clavulanic acid biosynthesis produce large amounts of holomycin: possible cross-regulation of two unrelated secondary metabolic pathways. *J. Bacteriol.* **184**, 6559-6565 (2002).
60. Paradkar, A. Clavulanic acid production by *Streptomyces clavuligerus*: biogenesis, regulation and strain improvement. *J. Antibiot.* **66,** 411-420 (2013).
61. Cao, G., Zhong, C., Zong, G., Fu, J., Liu, Z., Zhang, G. & Qin, R. Complete genome sequence of *Streptomyces clavuligerus* F613-1, an industrial producer of clavulanic acid. *Genome Announc.* **4,** e01020-16 (2016).
62. Cone, M. C., Yin, X., Grochowski, L. L., Parker, M. R. & Zabriskie, T. M. The blasticidin S biosynthesis gene cluster from *Streptomyces griseochromogenes*: sequence analysis, organization, and initial characterization. *Chembiochem* **4,** 821-828 (2003).
63. Wu, L., Chen, G. & Feng, G. Complete genome sequence of *Streptomyces griseochromogenes* ATCC 14511 T, a producer of nucleoside compounds and diverse secondary metabolites. *J. Biotechnol.* **249,** 16-19 (2017).
64. Peschke, U., Schmidt, H., Zhang, H. Z. & Piepersberg, W. Molecular characterization of the lincomycin-production gene cluster of *Streptomyces lincolnensis* 78-11. *Mol. Microbiol.* **16,** 1137–1156 (1995).
65. Brown, R., Hazen, E. L. & Mason, A. Effect of fungicidin (nystatin) in mice injected with lethal mixtures of Aureomycin and *Candida albicans*. *Science* **117,** 609-610 (1953).
66. Lamb, D.C. *et al*. *Streptomyces coelicolor* A3(2) CYP102 protein, a novel fatty acid hydroxylase encoded as a heme domain without an N-terminal redox partner. *Appl. Environ. Microbiol.* **76,** 1975-1980 (2010).
67. Moody, S. C. & Loveridge, E. J. CYP105—diverse structures, functions and roles in an intriguing family of enzymes in Streptomyces. *J. Appl. Microbiol.* **117,** 1549-1563, (2014).
68. Li, Z. Z., Li, X. F., Yang, W., Dong, X., Yu, J., Zhu, S. L., Li, M., Xie, L. & Tong, W. Y. Identification and functional analysis of cytochrome P450 complement in *Streptomyces virginiae* IBL14. *BMC Genomics* **14(1),** p.130. (2013).
69. Sherman, D. H., Li, S., Yermalitskaya, L. V., Kim, Y., Smith, J. A., Waterman, M. R. & Podust, L. M. The structural basis for substrate anchoring, active site selectivity, and product formation by P450 PikC from *Streptomyces* *venezuelae*. *J. Biol. Chem.* **281,** 26289–26297 (2006).
70. Zhang, H., Chen, J., Wang, H., Xie, Y., Ju, J., Yan, Y. & Zhang, H. Structural analysis of HmtT and HmtN involved in the tailoring steps of himastatin biosynthesis. *FEBS Lett.* **587,** 1675-1680 (2013).
71. Chen, D., Zhang, Q., Zhang, Q., Cen, P., Xu, Z. & Liu, W. Improvement of FK506 production in *Streptomyces* *tsukubaensis* by genetic enhancement of the supply of unusual polyketide extender units via utilization of two distinct site-specific recombination systems. *Appl. Environ. Microbiol.* **78,** 5093-5103 (2012).
72. Zhao, B. *et al*. Different binding modes of two flaviolin substrate molecules in cytochrome P450 158A1 (CYP158A1) compared to CYP158A2. Biochemistry **46**, 8725–8733 (2007).
73. Zhao, B., Bellamine, A., Lei, L. & Waterman, M. R. The role of Ile87 of CYP158A2 in oxidative coupling reaction. *Arch. Biochem. Biophys.* **518,** 127-132 (2012).
74. Podust, L. M., Kim, Y., Arase, M., Neely, B. A., Beck, B. J., Bach, H., Sherman, D. H., Lamb, D. C., Kelly, S. L. & Waterman, M. R. The 1.92- Å structure of *Streptomyces coelicolor* A3(2) CYP154C1 a new monooxygenase that functionalizes macrolide ring systems. *J. Biol. Chem.* **278,** 12214-12221 (2003).
75. Podust, L. M., Bach, H., Kim, Y., Lamb, D. C., Arase, M., Sherman, D. H., Kelly, S. L. & Waterman, M. R. Comparison of the 1.85 Å, structure of CYP154A1 from *Streptomyces coelicolor* A3(2) with the closely related CYP154C1 and CYPs from antibiotic biosynthetic pathways. *Protein Sci.* **13,** 255–268 (2004).
76. Cheng, Q., Lamb, D. C., Kelly, S. L., Lei, L. & Guengerich, F. P. Cyclization of a cellular dipentaenone by *Streptomyces coelicolor* cytochrome P450 154A1 without oxidation/reduction. *J. Am. Chem. Soc.* **132,** 15173-15175 (2010).
77. Moody, S. C., Zhao, B., Lei, L., Nelson, D. R., Mullins, J. G., Waterman, M. R., Kelly, S. L. & Lamb, D. C. Investigating conservation of the albaflavenone biosynthetic pathway and CYP170 bifunctionality in streptomycetes. *FEBS J.* **279,** 1640–1649 (2012).
78. Takamatsu, S., Xu, L. H., Fushinobu, S., Shoun, H., Komatsu, M., Cane, D. E. & Ikeda, H. Pentalenic acid is a shunt metabolite in the biosynthesis of the pentalenolactone family of metabolites: hydroxylation of 1-deoxypentalenic acid mediated by CYP105D7 (SAV_7469) of *Streptomyces avermitilis*. *J. Antibiot.* **64,** 65–71 (2011).
79. Zhao, B., Lei, L., Vassylyev, D. G., Lin, X., Cane, D. E., Kelly, S. L., Yuan, H., Lamb, D. C. & Waterman, M. R. Crystal structure of albaflavenone monooxygenase containing a moonlighting terpene synthase active site. *J. Biol. Chem.* **284,** 36711–36719 (2009).
80. Yu, F., Li, M., Xu, C., Wang, Z., Zhou, H., Yang, M., Chen, Y., Tang, L. & He, J. Structural insights into the mechanism for recognizing substrate of the cytochrome P450 enzyme TxtE. *PLoS ONE* **8,** e81526 (2013).
81. Healy, F. G., Krasnoff, S. B., Wach, M., Gibson, D. M. & Loria, R. Involvement of a cytochrome P450 monooxygenase in thaxtomin—a biosynthesis by *Streptomyces acidiscabies*. *J. Bacteriol.* **184,** 2019– 2029 (2002).
82. Kelly, A. K., McGee, M., Crews, D. H., Sweeney, T., Boland, T. M. & Kenny, D. A. Repeatability of feed efficiency, carcass ultrasound, feeding behavior, and blood metabolic variables in finishing heifers divergently selected for residual feed intake 1. *J. Anim. Sci.* **88,** 3214-3225 (2010).
83. Zocher, G., Richter, M. E., Mueller, U. & Hertweck, C. Structural fine-tuning of a multifunctional cytochrome P450 monooxygenase. *J. Am. Chem. Soc.* **133,** 2292–2302 (2011).
84. Haslinger, K., Brieke, C., Uhlmann, S., Sieverling, L., Süssmuth, R. D. & Cryle, M. J. The structure of a transient complex of a nonribosomal peptide synthetase and a cytochrome P450 monooxygenase. *Angew. Chem. Int. Ed. Engl.* **53(32),** 8518-8522 (2014).
85. Jaffrezou, J. P. *et al*. Daunorubicin-induced apoptosis: myelin hydrolysis. *EMBO J.* **15,** 2417–2424 (1996).
86. Dickens, M. L., Priestley, N. D. & Strohl, W. R. *In vivo* and *in vitro* bioconversion of epsilon-rhodomycinone glycoside to doxorubicin: functions of DauP, DauK, and DoxA. *J. Bacteriol.* **179,** 2641-2650 (1997).
87. Walczak, R. J., Dickens, M. L., Priestley, N. D. & Strohl, W. R. Purification, properties, and characterization of recombinant *Streptomyces sp* strain C5 DoxA, a cytochrome P-450 catalyzing multiple steps in doxorubicin biosynthesis. *J. Bacteriol.* **181,** 298–304 (1999).
88. Xie, Z., Niu, G., Li, R., Liu, G. & Tan, H. Identification and characterization of SanH and SanI involved in the hydroxylation of pyridyl residue during nikkomycin biosynthesis in *Streptomyces* *ansochromogenes*. *Curr. Microbiol*. **55,** 537–542 (2007).
89. Huang, S., Bjornsti, M. A. & Houghton P. J. Rapamycins: mechanism of action and cellular resistance. Cancer Biol. Ther. **2,** 222–232 (2003).
90. Molnár, I., Aparicio, J. F., Haydock, S. F., Khaw, L. E., Schwecke, T., König, A., Staunton, J. & Leadlay, P. F. Organisation of the biosynthetic gene cluster for rapamycin in *Streptomyces hygroscopicus*: analysis of genes flanking the polyketide synthase. *Gene* **169,** 1–7 (1996).
91. Quaderer, R., Omura, S., Ikeda, H., & Cane, D. E. Pentalenolactone biosynthesis. Molecular cloning and assignment of biochemical function to PtII, a cytochrome P450 of Streptomyces avermitilis. *J. Am. Chem. Soc.* **128,** 13036–13037 (2006).
92. Belin, P. *et al*. Identification and structural basis of the reaction catalyzed by CYP121, an essential cytochrome P450 in *Mycobacterium tuberculosis*. *Proc. Nat. Acad. Sci.* **106(18),** 7426-7431 (2009).
93. McLean, K. J., Lafite, P., Levy, C., Cheesman, M. R., Mast, N., Pikuleva, I. A., Leys, D. & Munro, A. W. The structure of *Mycobacterium tuberculosis* CYP125 molecular basis for cholesterol binding in a P450 needed for host infection. *J. Biol. Chem.* **284,** 35524-35533 (2009).
94. Ouellet, H. *et al*. *Mycobacterium tuberculosis* CYP125A1, a steroid C27 monooxygenase that detoxifies intracellularly generated cholest‐4‐en‐3‐one. *Mol. Microbiol.* **77,** 730-742 (2010).
95. Johnston, J. B., Kells, P. M., Podust, L. M. & de Montellano, P. R. O. Biochemical and structural characterization of CYP124: A methyl-branched lipid ω-hydroxylase from *Mycobacterium tuberculosis*. *Proc. Nat. Acad. Sci.* **106,** 20687-20692 (2009).
96. Belcher, J. *et al*. Structure and biochemical properties of the alkene producing cytochrome P450 OleTJE (CYP152L1) from the *Jeotgalicoccus* sp. 8456 bacterium. *J. Biol. Chem.* **289,** 6535-6550 (2014).
97. Hasemann, C. A., Ravichandran, K. G., Peterson, J. A. & Deisenhofer, J. Crystal structure and refinement of cytochrome P450terp at 2·3 Å resolution. *J. Mol. Biol.* **236,** 1169-1185 (1994).
98. Warman, A. J. *et al.* Characterization of *Cupriavidus metallidurans* CYP116B1-a thiocarbamate herbicide oxygenating P450-phthalate dioxygenase reductase fusion protein. *FEBS J.* **279,** 1675–1693 (2012).
99. Çelik, A., Roberts, G. A., White, J. H., Chapman, S. K., Turner, N. J. & Flitsch, S. L. Probing the substrate specificity of the catalytically self-sufficient cytochrome P450RhF from a *Rhodococcus sp*. *Chem. Commun.* **43,** 4492-4494 (2006).
100. Mve-Obiang, A. *et al*. A newly discovered mycobacterial pathogen isolated from laboratory colonies of Xenopus species with lethal infections produces a novel form of mycolactone, the *Mycobacterium ulcerans* macrolide toxin. *Infect. Immun.* **73,** 3307-3312 (2005).
101. Pylypenko, O., Vitali, F., Zerbe, K., Robinson, J. A. & Schlichting, I. Crystal structure of OxyC, a cytochrome P450 implicated in an oxidative C–C coupling reaction during vancomycin biosynthesis. *J. Biol. Chem.* ***278*,** 46727-46733 (2003).
102. Cryle, M. J., Meinhart, A. & Schlichting, I. Structural characterization of OxyD, a cytochrome P450 involved in beta-hydroxytyrosine formation in vancomycin biosynthesis. *J. Biol. Chem.* **285,** 24562– 24574 (2010).
103. Bell, S. G., Xu, F., Forward, I., Bartlam, M., Rao, Z. & Wong, L. L. Crystal structure of CYP199A2, a para-substituted benzoic acid oxidizing cytochrome P450 from *Rhodopseudomonas palustris*. *J. Mol. Biol.* **383,** 561–574 (2008).
104. Bell, S. G., Yang, W., Tan, A. B., Zhou, R., Johnson, E. O., Zhang, A., Zhou, W., Rao, Z. & Wong, L. L. The crystal structures of 4-methoxybenzoate bound CYP199A2 and CYP199A4: structural changes on substrate binding and the identification of an anion binding site. *Dalton Trans.* **41,** 8703–8714 (2012).
105. Child, S. A., Naumann, E. F., Bruning, J. B. & Bell, S. G. Structural and functional characterisation of the cytochrome P450 enzyme CYP268A2 from *Mycobacterium marinum*. *Biochem. J.*, BCJ20170946 (2018).
106. Lim, Y. R., Hong, M. K., Kim, J. K., Doan, T. T. N., Kim, D. H., Yun, C. H., Chun, Y. J., Kang, L. W. & Kim, D. Crystal structure of cytochrome P450 CYP105N1 from *Streptomyces coelicolor*, an oxidase in the coelibactin siderophore biosynthetic pathway. *Arch. Biochem. Biophys.* **528**, 111–117 (2012).
107. Zhao, B., Moody, S. C., Hider, R. C., Lei, L., Kelly, S. L., Waterman, M. R. & Lamb, D. C. Structural analysis of cytochrome P450 105N1 involved in the biosynthesis of the zincophore, coelibactin. *Int. J. Mol. Sci.***13,** 8500-8513 (2012).
108. Tian, Z., Cheng, Q., Yoshimoto, F. K., Lei, L., Lamb, D. C. & Guengerich, F. P. Cytochrome P450 107U1 is required for sporulation and antibiotic production in *Streptomyces coelicolor*. *Arch. Biochem. Biophys.* **530,** 101–107 (2013).
109. Xu, L. H., Fushinobu, S., Takamatsu, S., Wakagi, T., Ikeda, H. & Shoun, H. Regio- & stereospecificity of filipin hydroxylation sites revealed by crystal structures of cytochrome P450 105P1 and 105D6 from *Streptomyces avermitilis*. *J. Biol. Chem.* **285,** 16844–16853 (2010).
110. Choi, K. Y., Jung, E., Jung, D. H., Pandey, B. P., Yun, H., Park, H. Y., Kazlauskas, R. J. & Kim, B. G. Cloning, expression and characterization of CYP102D1, a self-sufficient P450 monooxygenase from *Streptomyces avermitilis*. *FEBS J.* **279,** 1650–1662 (2012).
111. Takamatsu, S., Lin, X., Nara, A., Komatsu, M., Cane, D. E. & Ikeda, H. Characterization of a silent sesquiterpenoid biosynthetic pathway in Streptomyces avermitilis controlling epi-isozizaene albaflavenone biosynthesis and isolation of a new oxidized epi-isozizaene metabolite. *Microb. Biotechnol.* **4,** 184–191 (2011).
112. Lamb, D. C., Zhao, B., Guengerich, F. P., Kelly, S. L. & Waterman, M. R.. *Genomics of Streptomyces cytochrome P450.* 233-253. (Caister Academic Press: Norfolk, UK, 2011)
113. Lawrence, C. H., Clark, M. C. & King, R. R. Induction of common scab symptoms in aseptically cultured potato-tubers by the vivotoxin, thaxtomin. *Phytopathology* **80,** 606–608 (1990).
114. Healy, F. G., Wach, M., Krasnoff, S. B., Gibson, D. M. & Loria, R. The txtAB genes of the plant pathogen *Streptomyces acidiscabies* encode a peptide synthetase required for phytotoxin thaxtomin—a production and pathogenicity. *Mol. Microbiol.* **38,** 794–804 (2000).
115. Challis, D., Yu, J., Evani, U. S., Jackson, A. R., Paithankar, S., Coarfa, C., Milosavljevic, A., Gibbs, R. A. & Yu, F. An integrative variant analysis suite for whole exome next-generation sequencing data. *BMC bioinformatics* **13(1),** 8 (2012).
116. Sugimoto, H. Crystal structure of *Streptomyces* *griseolus* CYP105A1 catalyzing vitamin D hydroxylation. *Vitamins* **82,** 497-502, (2008).
117. Hayashi, K., Sugimoto, H., Shinkyo, R., Yamada, M., Ikeda, S., Ikushiro, S., Kamakura, M., Shiro, Y. & Sakaki, T. Structure-based design of a highly active vitamin D hydroxylase from *Streptomyces griseolus* CYP105A1. *Biochemistry* **47,** 11964–11972 (2008).
118. Janocha, S., Zapp, J., Hutter, M., Kleser, M., Bohlmann, J. & Bernhardt, R. Resin acid conversion with CYP105A1: an enzyme with potential for the production of pharmaceutically relevant diterpenoids. *ChemBioBhem* **14,** 467–473 (2013).
119. Makino, T., Katsuyama, Y., Otomatsu, T., Misawa, N. & Ohnishi, Y. Regio- and stereospecific hydroxylation of various steroids at the 16 alpha position of the D ring by the *Streptomyces griseus* cytochrome P450 cyp154c3. *Appl. Environ. Microbiol.* **80,** 1371–1379 (2014).
120. Uhlmann, S., Süssmuth, R. D. & Cryle, M. J. Cytochrome P450 (sky) interacts directly with the nonribosomal peptide synthetase to generate three amino acid precursors in skyllamycin biosynthesis. *ACS Chem. Biol.* **8,** 2586–2596 (2013).
121. Caffrey, P., Lynch, S., Flood, E., Finnan, S. & Oliynyk, M. Amphotericin biosynthesis in *Streptomyces* *nodosus*: deductions from analysis of polyketide synthase and late genes. *Chem. Biol.* **8,** 713-723 (2001).
122. Agarwal, P. K., Agarwal, P., Reddy, M. K. & Sopory, S. K. Role of DREB transcription factors in abiotic and biotic stress tolerance in plants. *Plant Cell Rep.* **25,** 1263-1274 (2006).
123. Ashy, M. A., Abd-El-Galil, M. K. & Abou-Zeid, A. Z. A. Carbomycin a macrolide antibiotic. *Zentralbl. Bakteriol. Naturwiss.* **135,** 541-551 (1980).
124. Ward, S. L., Hu, Z., Schirmer, A., Reid, R., Revill, W. P., Reeves, C. D., Petrakovsky, O. V., Dong, S. D. & Katz, L. Chalcomycin biosynthesis gene cluster from *Streptomyces bikiniensis*: novel features of an unusual ketolide produced through expression of the Chm polyketide synthase in *Streptomyces fradiae*. *Antimicrob. Agents Chemother.* **48,** 4703-4712 (2004).
125. Reading, C. & Cole, M. Clavulanic acid-beta-lactamase- inhibiting beta-lactam from *Streptomyces clavuligerus*. *Antimicrob. Agents Chemother.* **11,** 852–857 (1977).
126. Kudo, F., Motegi. A., Mizoue, K. & Eguchi, T. Cloning and characterization of the biosynthetic gene cluster of 16-membered macrolide antibiotic FD-891: involvement of a dual functional cytochrome P450 monooxygenase catalyzing epoxidation and hydroxylation. *ChemBioChem* **11,** 1798–1798 (2010).
127. Kataoka, T., Yamada, A., Bando, M., Honma, T., Mizoue, K.& Nagai, K. FD-891, a structural analogue of concanamycin-A that does not affect vacuolar acidification or perforin activity, yet potently prevents cytotoxic T-lymphocyte-mediated cytotoxicity through the blockage of conjugate formation. *Immunology* **100,** 170–177 (2000).
128. Liu, Z., Zhang, W., Huang, F., Feng, H., Shu, W., Xu, X. & Chen, Y. High throughput capture of circulating tumor cells using an integrated microfluidic system. *Biosens. Bioelectron.* **47,** 113-119 (2013).
129. Kong, R., Liu, X., Su, C., Ma, C., Qiu, R. & Tang, L. Elucidation of the biosynthetic gene cluster and the post-PKS modification mechanism for fostriecin in *Streptomyces pulveraceus*. *Chem. Biol.* **20,** 45-54 (2013).
130. Leet, J. E., Schroeder, D. R., Golik, J., Matson, J. A., Doyle, T. W., Lam, K. S., Hill, S. E., Lee, M. S., Whitney, J. L. & Krishnan, B. S. Himastatin, a new antitumor antibiotic from *Streptomyces hygroscopicus*. *J. Antibiot.* **49,** 299–311 (1996).
131. Ma, J., Wang, Z., Huang, H., Luo, M., Zuo, D., Wang, B., Sun, A., Cheng, Y. Q., Zhang, C. & Ju, J. Biosynthesis of himastatin: assembly line and characterization of three cytochrome P450 enzymes involved in the post-tailoring oxidative steps. *Angew Chem. Int. Ed.* **50,** 7797–7802 (2011).
132. Zheng, R., Chen, Z., Cai, C., Wang, X., Huang, Y., Xiao, B. & Sun, G. Effect of biochars from rice husk, bran, and straw on heavy metal uptake by pot-grown wheat seedling in a historically contaminated soil. *BioResources* **8,** 5965-5982 (2013).
133. Lauer, B., Russwurm, R., Schwarz, W., Kalmanczhelyi, A., Bruntner, C., Rosemeier, A. & Bormann, C. Molecular characterization of co-transcribed genes from Streptomyces tendae Tu901 involved in the biosynthesis of the peptidyl moiety and assembly of the peptidyl nucleoside antibiotic nikkomycin. *Mol. Gen. Genet.* **264,** 662–673 (2001).
134. Steffensky, M., Muhlenweg, A., Wang, Z. X., Li, S. M., & Heide, L. Identification of the novobiocin biosynthetic gene cluster of *Streptomyces spheroids* NCIB 11891. Antimicrob. Agents Chemother. **44,** 1214–1222 (2000).
135. Chen, H. W. & Walsh C. T. Coumarin formation in novobiocin biosynthesis: beta-hydroxylation of the aminoacyl enzyme tyrosyl-S-NovH by a cytochrome P450 NovI. *Chem. Biol.* **8,** 301–312 (2001).
136. Rodriguez, A. M., Olano, C., Mendez, C., Hutchinson, C. R., & Salas J. A cytochrome P450-like gene possibly involved in oleandomycin biosynthesis by streptomyces-antibioticus. *FEMS Microbiol. Lett.* **127,** 117–120 (1995).
137. Shrestha, P., Oh T. J., Liou, K. & Sohng, J. K. Cytochrome P450 (cyp105f2) from *Streptomyces peucetius* and its activity with oleandomycin. *Appl. Microbiol. Biotechnol.* **79,** 555–562 (2008).
138. Niraula, N. P., Kanth, B. K., Sohng, J. K., & Oh, T. J. Hydrogen peroxide-mediated dealkylation of 7-ethoxycoumarin by cytochrome P450 (CYP107AJ1) from *Streptomyces peucetius* ATCC27952. *Enzyme Microb. Technol.* **48,** 181–186 (2011).
139. Wang, F., Kong, R., Liu, B., Zhao, J., Qiu, R. & Tang, L. Functional characterization of the genes tauO, tauK, and tauI in the biosynthesis of tautomycetin. *J. Microbiol.* **50,** 770–776 (2012).
140. Kim, D., Nah, J. H., Choi, S. S., Shin, H. S., Sherman, D. H. & Kim, E. S. Biological activities of an engineered tautomycetin analogue *via* disruption of tmcR-encoding hydroxylase in *Streptomyces* sp. CK4412. J. Ind. Microbiol. Biotechnol. **39,** 1563–1568 (2012).
141. Merson-Davies L. A. & Cundliffe, E. Analysis of five tylosin biosynthetic genes from the tyllBA region of the *Streptomyces fradiae* genome. *Mol. Microbiol.* **13,** 349–355, (1994).
142. Fouces, R., Mellado, E., Diez, B., & Barredo, J. L. The tylosin biosynthetic cluster from Streptomyces fradiae: genetic organization of the left region. *Microbiology* **145**, 855–868 (1999).
143. Carlson, J. C., Fortman, J. L., Anzai, Y., Li, S., Burr, D. A., & Sherman, D. H., Identification of the tirandamycin biosynthetic gene cluster from *Streptomyces* sp 307–309. *ChemBioChem* **11,** 564–572 (2010).
144. Carlson, J. C., Li, S., Gunatilleke, S. S., Anzai, Y., Burr, D. A., Podust, L. M. & Sherman, D. H. Tirandamycin biosynthesis is mediated by co-dependent oxidative enzymes. *Nat. Chem.* **3,** 628–633 (2011).
